# Preparative‐Scale Biocatalytic Oxygenation of *N*‐Heterocycles with a Lyophilized Peroxygenase Catalyst

**DOI:** 10.1002/anie.202214759

**Published:** 2022-12-22

**Authors:** Balázs Pogrányi, Tamara Mielke, Alba Díaz‐Rodríguez, Jared Cartwright, William P. Unsworth, Gideon Grogan

**Affiliations:** ^1^ Department of Chemistry University of York Heslington York YO10 5DD UK; ^2^ GSK Medicines Research Centre Gunnels Wood Road Stevenage Hertfordshire, SG1 2NY UK; ^3^ Department of Biology University of York Heslington York YO10 5DD UK

**Keywords:** Biocatalysis, Cytochromes P450, Oxygenation, Peroxygenase, Unspecific Peroxygenase (UPO)

## Abstract

A lyophilized preparation of an unspecific peroxygenase variant from *Agrocybe aegerita* (r*Aae*UPO‐PaDa‐I‐H) is a highly effective catalyst for the oxygenation of a diverse range of *N*‐heterocyclic compounds. Scalable biocatalytic oxygenations (27 preparative examples, ca. 100 mg scale) have been developed across a wide range of substrates, including alkyl pyridines, bicyclic *N*‐heterocycles and indoles. H_2_O_2_ is the only stoichiometric oxidant needed, without auxiliary electron transport proteins, which is key to the practicality of the method. Reaction outcomes can be altered depending on whether hydrogen peroxide was delivered by syringe pump or through in situ generation using an alcohol oxidase from *Pichia pastoris* (*Pp*AOX) and methanol as a co‐substrate. Good synthetic yields (up to 84 %), regioselectivity and enantioselectivity (up to 99 % *ee*) were observed in some cases, highlighting the promise of UPOs as practical, versatile and scalable oxygenation biocatalysts.

## Graphical abstract

A lyophilized preparation of an unspecific peroxygenase variant from *Agrocybe aegerita* (r*Aae*UPO‐PaDa‐I‐H) is a highly effective catalyst for the oxygenation of a diverse range of *N*‐heterocyclic compounds. Scalable, high yielding and enantioselective biocatalytic oxygenations have been developed, including 27 preparative examples (ca. 100 mg scale) across a wide range of substrates, including alkyl pyridines, bicyclic *N*‐heterocycles and indoles.

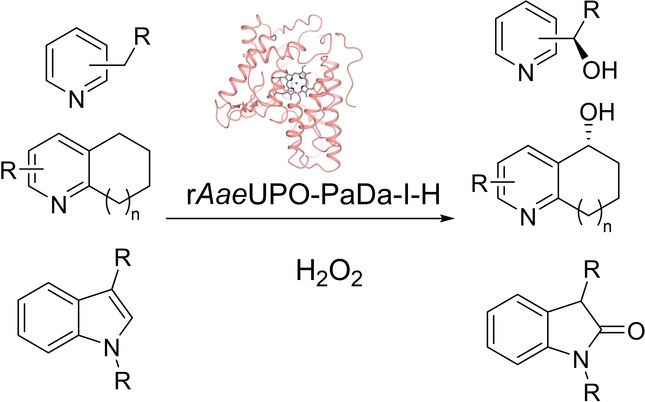

## Introduction

The oxygenation of non‐activated carbon atoms remains one of the most important reactions in synthetic chemistry, for the synthesis of bulk chemical precursors and the preparation of late‐stage oxygenated intermediates and drug metabolites.[^1^, ^2^] As toxic reagents and/or harsh reaction conditions often needed when using abiotic oxidants, oxygenase enzymes are of significant interest, particularly for the functionalization of complex molecules with high regio‐ and enantioselectivity.[^3^, ^4^, ^5^, ^6^] Cytochromes P450 (P450s) are known to catalyze the selective oxygenation reactions of a range of substrates[^7^, ^8^, ^9^] including bulk chemicals such as alkanes^[10]^ and aromatics,^[11]^ as well more complex molecules like steroids,^[12]^ terpenes^[13]^ and pharmaceuticals.^[14]^

Despite considerable research on recombinant P450s expressed in heterologous hosts, their use in preparative scale oxygenation is limited, with only a few industrial examples having been developed.[^15^, ^16^, ^17^] This is due to challenges associated with the heterologous expression of P450s (particularly eukaryotic P450s), poor turnover rates, low stability, and the mode of electron transport to the active site heme in P450s, which requires a nicotinamide cofactor (NAD(P)/H) and auxiliary redox proteins (e.g. cytochrome P450 reductases and ferredoxin reductases/ferredoxins).

The development of alternative enzyme systems for hydroxylation that avoid these disadvantages is therefore important. In 2004, Hofrichter and co‐workers reported the discovery of heme‐dependent unspecific peroxygenases (UPOs) in fungal species, such as the enzyme from *Agrocybe aegerita* (*Aae*UPO).[^18^, ^19^, ^20^] This new class of oxygenase enzymes can catalyze the oxygenation of a range of unactivated carbon atoms in alkanes,^[21]^ alkenes^[22]^ aromatics^[20]^ and drug molecules,[^23^, ^24^] but crucially, *at the expense only of the hydrogen peroxide as the stoichiometric oxidant, without the need for NADPH or auxiliary electron transport proteins*. Recently developed heterologous systems for the expression of UPOs[^25^, ^26^, ^27^, ^28^] have facilitated the application of both *Aae*UPO and other enzymes to the oxygenation of a number of substrates, including a 115 mmol‐scale production of butanol from butane in a bioreactor.^[29]^ These practically simpler hydroxylation biocatalysts can be considered much more suitable than P450s for scalable applications.

**Scheme 1:**
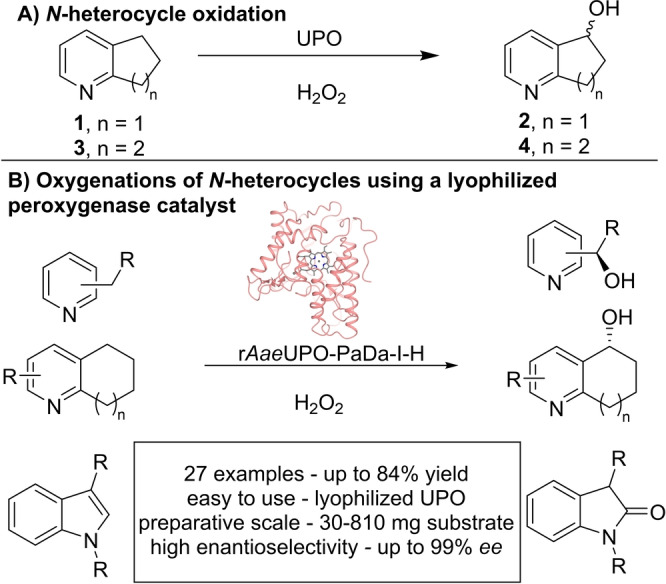
A) Exemplar biocatalytic hydroxylation of *N*‐heterocycles **1** and **3**. B) Oxygenation of *N*‐heterocycles using a lyophilized peroxygenase catalyst.

This study is focused on exploring the oxygenation of *N*‐heterocyclic compounds (Scheme 1). *N*‐heterocycles feature heavily in pharmaceutical compounds, and have been the target of biocatalytic oxygenations by various alternative systems.[^30^, ^31^, ^32^, ^33^] The oxygenation of *N*‐heterocycles, like pyridine derivatives **1** and **3**, were identified as reactions of interest (Scheme 1A), and hence their oxygenation was explored using *Aae*UPO, in addition to a much wider array of *N*‐heterocyclic systems including alkyl pyridines, bicyclic *N*‐heterocycles and indoles (Scheme 1B). The results show that, in contrast to cell‐free P450‐based systems, *Aae*UPO can be readily applied to the hundreds‐of‐milligrams scale synthesis of oxygenated *N*‐heterocyclic intermediates, with remarkable promiscuity, when applied as an easy‐to‐use cell free lyophilized powder. Significantly, the method was used successfully in 27 preparative scale examples (30–810 mg). As expected, reaction yields and selectivities varied depending on the substrate; nonetheless, with moderate‐to‐good isolated yields generally observed (up to 84 %), and high regioselectivity and enantioselectivity (up to 99 % *ee*) in several cases, *Aae*UPO clearly has much promise as a practical, versatile and scalable oxygenation biocatalyst.

## Results and Discussion

Prior to this study, the expression of useful amounts of *Aae*UPO in *E. coli* had not been reported. Therefore, recombinant production of the enzyme was carried out using the yeast host *Pichia pastoris*. The gene encoding the 9‐point mutant of *Aae*UPO, described by Alcalde and co‐workers[^25^, ^26^] was cloned into a modified pPICZα vector and equipped with a C‐terminal histidine tag to give a variant r*Aae*UPO‐Pada‐I‐H as described previously.^[34]^ This mutant sequence was chosen as the wild‐type *Aae*UPO was reported not to express well in *P. pastoris*.[^25^, ^26^] 19 L of fermentation in *Pichia* resulted in culture supernatant that was concentrated and lyophilized to give 138 g of powdered enzyme.

### Oxygenation of Alkylpyridines

**Scheme 2:**
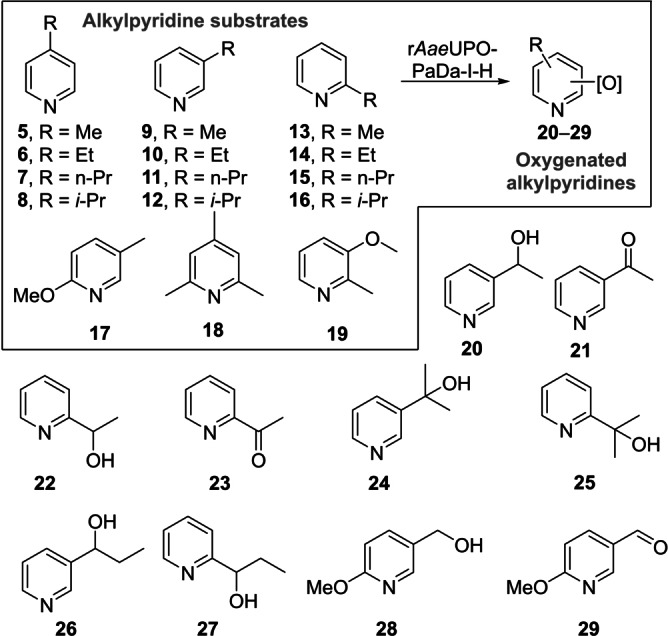
Alkylpyridine substrates used and products identified in a first screen using r*Aae*UPO‐PaDa‐I‐H.

r*Aae*UPO‐Pada‐I‐H was then used in a first screen with a range of alkylpyridines. Thus, alkylpyridines **5** to **16** (10 mM in 5 mL of 50 mM KPi buffer pH=7.0) were reacted with the enzyme (Scheme 2) with slow addition of two equivalents of hydrogen peroxide by syringe pump at a rate of 0.08 eq h^−1^ (Supporting Information Section 4 Method 1). 4‐Alkylpyridines **5**–**8** were not transformed by r*Aae*UPO‐Pada‐I‐H, which we propose may be because of coordination of the unhindered *N*‐atom in these substrates to the heme iron of the enzyme. Similarly, 3‐picoline **9**, 2‐picoline **13**, 2,4,6‐trimethylpyridine **18** and 3‐methoxy‐2‐picoline **19** were not transformed. Interestingly the behaviour with **9** contrasts with that reported for wild‐type *Aae*UPO, in which a mixture of products with oxygenation at both the the methyl group and the N atom was observed^35]^ although the substrate concentration was in that case was much lower (500 μM). However, alkylpyridines with more shielded *N*‐atoms gave more acceptable conversions. For example, 3‐ethylpyridine **10**, was converted into a 9 : 1 mixture of the (*R*)‐enantiomer of alcohol **20** with >98 % *ee* and the corresponding ketone **21**, in 98 % conversion overall. 2‐Ethylpyridine **14** was also transformed, albeit with only 3 % conversion into alcohol **22** under these conditions. 3‐Isopropyl pyridine **12** and 2‐isopropyl pyridine **16** were transformed into tertiary alcohols **24** and **25** with 17 % and 3 % conversions respectively. 3‐*n*‐Propylpyridine **11** and the 2‐*n*‐propyl isomer **15** were converted to benzylic alcohols **26** (4 %) and **27** (15 %). Finally, 2‐methoxy 5‐methyl pyridine **17** was transformed into alcohol **28** and aldehyde **29** with 30 % and 70 % conversion respectively. The enantioselectivity of most of the transformations that afforded chiral products was not recorded at this point, although as is shown later in the manuscript, the enantioselectivity was very high in all cases measured.

*Aae*UPO is known to be deactivated by hydrogen peroxide^[36]^ and in our hands r*Aae*UPO‐Pada‐I‐H lost 50 % of activity even at 1 mM H_2_O_2_, measured using the veratryl alcohol peroxygenase assay (Supporting Information, Sections 5 and 6; Figure S1).^[23]^ In an attempt therefore to reduce the peroxide stress on the UPO, a system for in situ generation of peroxide was evaluated, using alcohol oxidase from *Pichia pastoris* (*Pp*AOX) and methanol, based on the method previously described by Hollmann and co‐workers.^[36]^ Three different amounts of *Pp*AOX (0.3, 1.0 and 3.0 U mL^−1^) were tested for each substrate (Supporting Information Section 4, Method 2), as it was observed that different substrates required different amounts of *Pp*AOX for high conversions. *Pp*AOX demonstrated no activity towards these or other substrates when applied without r*Aae*UPO‐PaDa‐I‐H. The best results showed that superior conversions could be achieved in many cases using in situ peroxide generation with selectivity conserved. In this manner, 3‐isopropyl pyridine **12** was now transformed into **24** with 80 % conversion and 2‐ethylpyridine **14** was hydroxylated to form the (*R*)‐benzyl alcohol **22** and ketone **23** with 36 % and 29 % conversion respectively.

**Figure 1:**
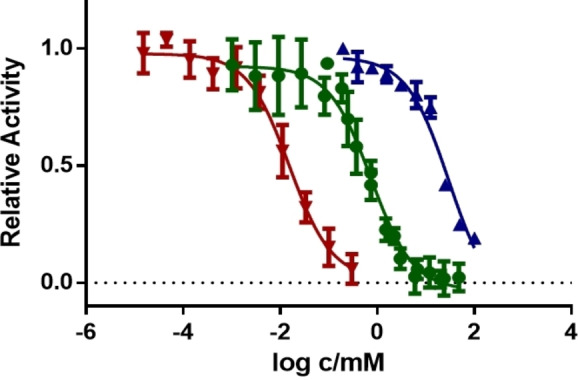
Inhibition of r*Aae*UPO‐PaDa‐I‐H by 2‐ethylpyridine (**14**, blue), 3‐ethylpyridine **10** (green) and 4‐ethylpyridine (**6**, red). IC_50_ **14=**29.5 mM, **10=**0.7 mM, **6=**0.016 mM.

The low conversion with 4‐alkylated substrates e.g. 4‐ethylpyridine **6** compared to their 2‐ and 3‐ethyl isomers, such as **14** and **10**, was investigated by comparing the inhibition of r*Aae*UPO‐Pada‐I‐H with these compounds using a competition‐based format of the 5‐nitro‐1,3‐benzodioxole assay for UPOs described previously (Figure 1, see Supporting Information Section 7 for further details).^[37]^ In this way, IC_50_ values for each of substrates **14**, **10** and **6** were obtained (Supporting Information Section 7, Table S1). The very low value of 0.016 mM recorded for **6** is reflective of the strong binding of the available nitrogen in this substrate to the heme iron, militating against productive binding for oxygenation.

### Oxygenation of Bicyclic Substrates

**Scheme 3:**
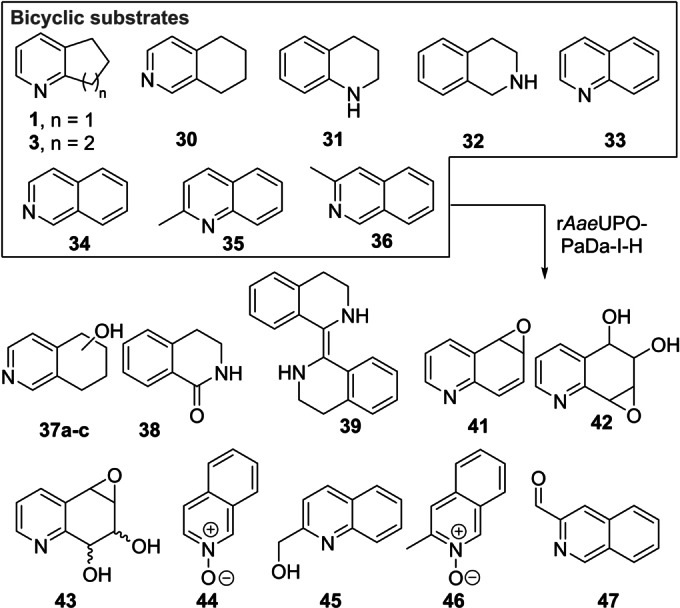
Bicyclic substrates used and products identified in a first screen using r*Aae*UPO‐PaDa‐I‐H.

The screening approach was extended to the r*Aae*UPO‐Pada‐I‐H catalyzed biotransformation of bicyclic compounds such as **1**, **3** and a range of bicyclic *N*‐heterocycles **30**–**36** (Scheme 3) using either syringe pump addition of hydrogen peroxide or in situ generation using *Pp*AOX (Supporting Information Section 8).

Products were identified by GC‐MS analysis and compared with authentic standards. Scale‐up of several of the reactions later (see below) also allowed additional verification of product identities by various characterisation methods. First, 2,3‐cyclohexenopyridine **3** was transformed into 5‐hydroxy product **4** with 75 % conversion using slow addition of H_2_O_2_. 5,6,7,8‐tetrahydroisoquinoline **30** was transformed into a mixture of three regioisomeric alcohols (**37 a**–**c**, approximately 1 : 1 : 1 ratio) with a combined conversion of 64 %, also using slow addition of H_2_O_2_. Lower selectivity was revealed in the transformation of 1,2,3,4‐tetrahydro(iso)quinoline derivatives **31** and **32**, with several unstable products (e.g. **38** and **39**) formed with either mode of peroxide delivery, which were later found to lead to the formation of oligomers upon scale‐up. Quinoline **33** was oxidized at various positions in its benzenoid ring, yielding epoxide **41**, its ring‐opened derivative **42** and dihydroxy epoxide **43** with 81 % combined conversion with slow addition of H_2_O_2_. The same conditions also saw isoquinoline **34** converted into *N*‐oxide **44** with high selectivity. In contrast, quinaldine (2‐methylquinoline) **35** was oxidized on the aromatic ring to form a phenolic product (unknown regioisomer, 32 % conversion) and at the benzylic position, to form **45** with 10 % conversion. Oxidation of 3‐methylisoquinoline **36** yielded *N*‐oxide **46** as the major product (82 %) and aldehyde **47** as a minor product (8 %), each again using syringe pump addition of peroxide. The formation of *N*‐oxides from both **34** and **36** is likely indicative of the greater availability of the nitrogen atom for oxidation in isoquinolines.

### Transformation of Indoles

**Scheme 4:**
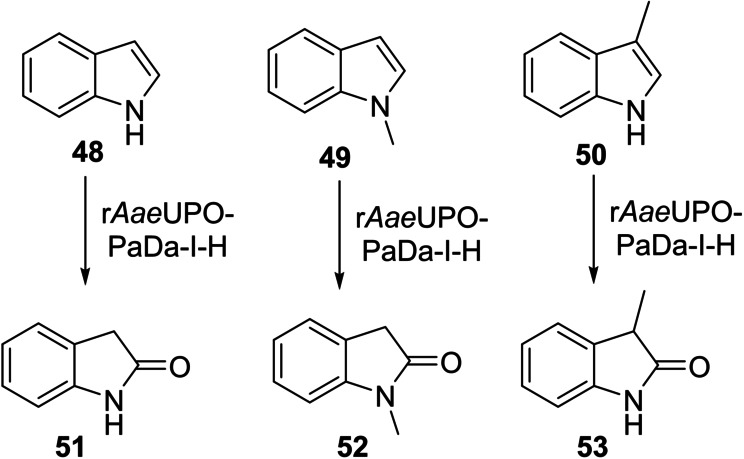
Indole substrates used and products identified in a first screen using r*Aae*UPO‐PaDa‐I‐H.

Three indole derivatives **48**, **49** and **50** were also tested, and each was converted smoothly into 2‐oxindole products; **51**, **52** and **53** were formed in 100 %, 85 % and 85 % conversions respectively, using slow addition of H_2_O_2_ in the case of **48** and **50**, and *Pp*AOX in the case of **49** (Scheme 4).

### Scaleable Oxygenations Using rAaeUPO‐Pada‐I‐H

**Scheme 5:**
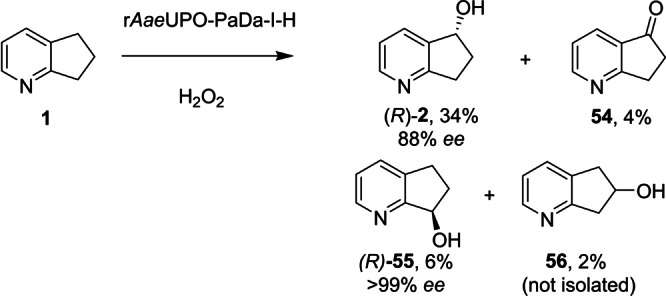
Oxidation of pyrindan **1** by r*Aae*UPO‐Pada‐I‐H on a 300 mg scale.

To optimize the r*Aae*UPO‐Pada‐I‐H/*Pp*AOX system for scaleable *N‐*heterocycle oxygenation, a design of experiments (DoE) approach was applied to the transformation of pyrindan **1** into chiral alcohol **2** (Supporting Information Section 9). Notably, **2** is a key intermediate in the synthesis of patented Ghrelin *O*‐acetyltransferase inhibitors,^[38]^ which underlines the utility of *Aae*UPO in the production of useful chiral feedstock molecules. As input factors, the substrate concentration (5–50 mM), the temperature (20–40 °C), the r*Aae*UPO‐H loading (1–10 U mL^−1^), and the r*Aae*UPO‐H to *Pp*AOX ratio (20 : 1–5 : 1) were chosen. Ten reactions were run using the factors described (Table S2) containing two centre‐points for reproducibility and eight factorial points, with a combination of factors. As responses, the GC‐based conversion to the desired hydroxylated product 6,7‐dihydro‐5*H*‐cyclopenta[*b*]pyridin‐5‐ol **2** and the GC‐based conversion to by‐products (**54**–**56**) were chosen. The results for the ten runs are shown in Table S3. Analysis of the DoE experiments suggested that the best results for obtaining **2** would be obtained at a 40 mM concentration of **1** at 20 °C using 10 U mL^−1^ UPO at a 5 : 1 ratio to *Pp*AOX (2 U mL^−1^). Informed by this result, a scale‐up reaction using 40 mM of **1** on a 300 mg scale was performed using 10 U mL^−1^ r*Aae*UPO‐PaDa‐I‐H at a 5 : 1 ratio with *Pp*AOx, at 20 °C. After 115 h, GC analysis suggested that products **2**, **54** and **55** were formed with 63 %, 4 % and 17 % conversion, as well as a small amount (2 %) of the product **56**, with hydroxylation in the 6‐position (Scheme 5). Following purification using flash chromatography (*R*)‐**2** was isolated in 34 % yield and with 88 % *ee*.

Following the success of this approach, the r*Aae*UPO‐PaDa‐I‐H/*Pp*AOX system was then applied to other preparative scale reactions, starting with the oxygenation of alkyl pyridines **10**, **14**, and **15** on scales ranging between 100–810 mg of substrate (Table 1, and Supporting Information Section 10). All yields quoted refer to isolated material following column chromatography. It was not always possible to fully separate the products in cases where mixtures of products were obtained, with full details of the isolated materials for all preparative scale reactions included in the Supporting Information.

**Table 1:** Preparative biotransformation of simple alkylpyridine substrates using r*Aae*UPO‐PaDa‐I‐H.

Substrate	Scale/ time	H_2_O_2_ method	Products Isolated Yield; *ee*
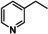	100 mg 10 mM 24 h	**A**	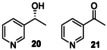
**10**			(*R*)‐**20**, 35 %; 98 % *ee* **21**, 50 %
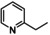	810 mg 20 mM 24 h	**A**	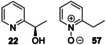
**14**			(*R*)‐**22**, 19 %; >99 % *ee* **57**, 16 %
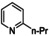	120 mg 20 mM 24 h	**A**	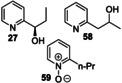
**15**			(*R*)‐**27**, 32 %; >99 % *ee* **58**, 14 % n.d.; **59**, 51 %

**A**: 50 mM KPi (pH=7.0), 10 % v/v MeCN, 400 mM MeOH, *Pp*AOX H_2_O_2_ generation; **B**: 5 mM KPi (pH=7.0), 10 % v/v MeCN, H_2_O_2_ slow addition; see Supporting Information Section 9 for details of both methods; n.d.=*ee* not determined.

First, 3‐ethyl pyridine **10** was tested, and it was transformed into the (*R*)‐alcohol **20** in 35 % isolated yield (>99 % *ee*), along with ketone **21** (50 %), resulting from further oxidation. In the same way, 2‐ethylpyridine **14** was converted into (*R*)‐alcohol **22** and the *N‐*oxide **57** in 19 % (>99 % *ee*) and 16 % isolated yield respectively. 2‐*n*‐Propylpyridine **15** was converted into a mixture of regioisomeric alcohols (**27** in 32 % yield, >99 % *ee* and **58** in 14 % yield), but the major product was the *N*‐oxide **59** (51 %).

Next, a range of disubstituted alkyl pyridines was tested, in order to explore substituent and electronic effects (Table 2). 2‐Methoxy‐5‐methylpyridine **17** was converted into primary alcohol **28** and aldehyde **29** with 31 % and 47 % isolated yields respectively. These results confirm that the biocatalyzed oxygenation method can be applied to methyl pyridines as well as ethyl pyridines. Pyridine **60**, which contains both an ethyl and methyl group, was oxygenated primarily on the ethyl benzylic position to form **61 a** in 73 % yield, with a low (3 %) yield of **61 b**. Dimethyl pyridine **62** showed clear selectivity for oxygenation on the *meta*‐ rather than *ortho*‐methyl group, affording oxidised products **63 a**–**c** in 84 % combined yield. 3,5‐Dimethyl pyridine **64** afforded a mixture of two oxidised products **65 a**,**b**.

**Table 2:** Preparative biotransformation of disubstituted alkylpyridine substrates using r*Aae*UPO‐PaDa‐I‐H.

Substrate	Scale/ time	H_2_O_2_ method	Products Isolated Yield; *ee*
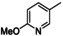	115 mg 10 mM 30 h	**B**	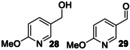
**17**			**28**, 31 %; **29**, 47 %
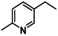	30 mg 10 mM 13 h	**B**	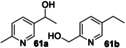
**60**			<**61 a**, 73 %, n.d; **61 b**, 3 %
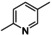	40 mg 10 mM 13 h	**B**	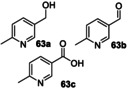
**62**			**63 a**, 48 %; **63 b**, 20 %; **63 c**, 16 %
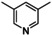	33 mg 10 mM 13 h	**B**	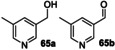
**64**			**65 a**, 24 %; **65 b**, 13 %
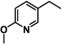	30 mg 10 mM 13 h	**B**	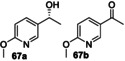
**66**			(*R*)**‐67 a**, 36 %; >99 % *ee*; **67 b**, 49 %
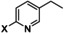	30 mg 10 mM 13 h	**B**	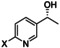
**68**, X=Cl **69**, X=CN **70**, X=CONH_2_ **71**, X=SO_2_Me			(*+*)‐**72**, X=Cl, 73 %; n.d. (*R*)‐**73**,^[a]^ X=CN, 84 %; >99 % *ee* (*R*)‐**74**, X=CONH_2_, 54 %; >99 % *ee* (*R*)**‐75**, X=SO_2_Me, 71 %; 92 % *ee*

**A**: 50 mM KPi (pH=7.0), 10 % v/v MeCN, 400 mM MeOH, *Pp*AOX H_2_O_2_ generation; **B**: 5 mM KPi (pH=7.0), 10 % v/v MeCN, H_2_O_2_ slow addition; see Supporting Information Section 9 for details of both methods; n.d.=*ee* not determined.^[a]^ 5 h reaction time.

2,5‐Disubstituted pyridines were the most effective substrates tested: for example, electron rich substrate **66** affording alcohol **67 a** in high *ee*, alongside over‐oxidised ketone product **67 b** in good combined yield. Electron deficient 2,5‐disubstituted pyridines also proved to be effective substrates, with ethyl pyridines **68**–**71** all being selectively converted into secondary alcohols **72**–**75** in good isolated yields (54–84 %) and with excellent *ee* (up to >99 %) in all cases measured.

**Scheme 6:**
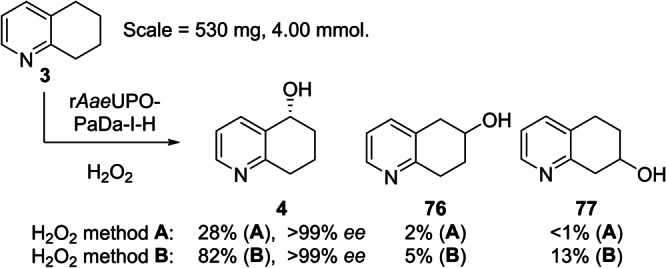
Oxidation of 2,3‐cyclohexenopyridine **3** by r*Aae*UPO‐Pada‐I‐H using H_2_O_2_ generation methods **A** and **B**. Isolated yields following column chromatography. 24 h (**A**) and 25 h (**B**) reaction times.

The preparative oxygenation of various bicyclic *N‐*heterocycles was then examined on scales ranging from 30–530 mg. In these cases, a relatively high amount of *Pp*AOX was generally required to reach synthetically useful yields when using this H_2_O_2_ generation mode (H_2_O_2_ method **A**). This prompted us, in most instances, to perform reactions with slow H_2_O_2_ addition (H_2_O_2_ method **B**), with superior products yields usually observed using this method; for example, substrate **3** was hydroxylated predominantly at the 5‐position to give (*R*)‐alcohol **4** (>99 % ee) in both cases, but in either 28 % or 82 % isolated yield using *Pp*AOX or slow H_2_O_2_ addition respectively. Interestingly, the identity of the side products formed was different depending which peroxide source was used, as the 6‐hydroxy product **76** was formed (although in only 2 % yield) when using *Pp*AOX, whereas the 7‐hydroxy compound **77** was formed (in 13 % isolated yield) when slow addition of H_2_O_2_ was employed (Scheme 6).

A range of other bicyclic *N‐*heterocycles was also examined in preparative scale reactions (Table 3). In the case of tetrahydroisoquinoline **30**, the enzyme was selective for oxygenation to form alcohol products, albeit with relatively low regioselectivity; benzylic isomers **37 a** and **37 c** and the 7‐hydroxy compound **37 b** were recovered in similar yields of 12 %, 22 % and 15 % respectively. Oxidation of quinoline **33** gave the epoxide **41** and the dihydroxy epoxide **42** in 60 % and 21 % isolated yields respectively. The oxidation of isoquinoline **34** on the same scale

**Table 3:** Preparative biotransformation of bicyclic *N*‐heterocycles using r*Aae*UPO‐PaDa‐I‐H.

Substrate	Scale/ time	H_2_O_2_ method	Products Isolated Yield
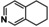	270 mg 10 mM 25 h	**B**	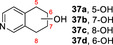
**30**			**37 a**, 12 %; **37 b**, 15 % **37 c**, 22 %; **37 d**, 5 %
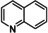	130 mg 10 mM 25 h	**B**	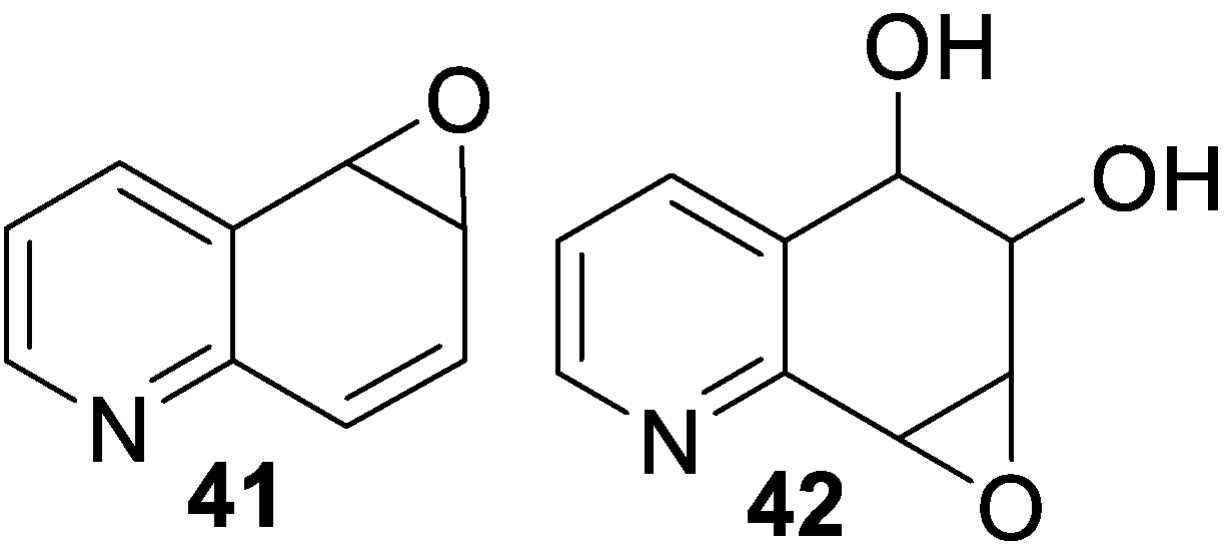
**33**			**41**, 60 %; **42**, 21 %
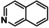	130 mg 10 mM 25 h	**B**	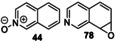
**34**			**44**, 34 %; **78**, 20 %
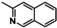	43 mg 10 mM 25 h	**B**	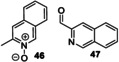
**36**			**46**, 59 %; **47**, 12 %
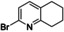	30 mg 10 mM 25 h	**B**	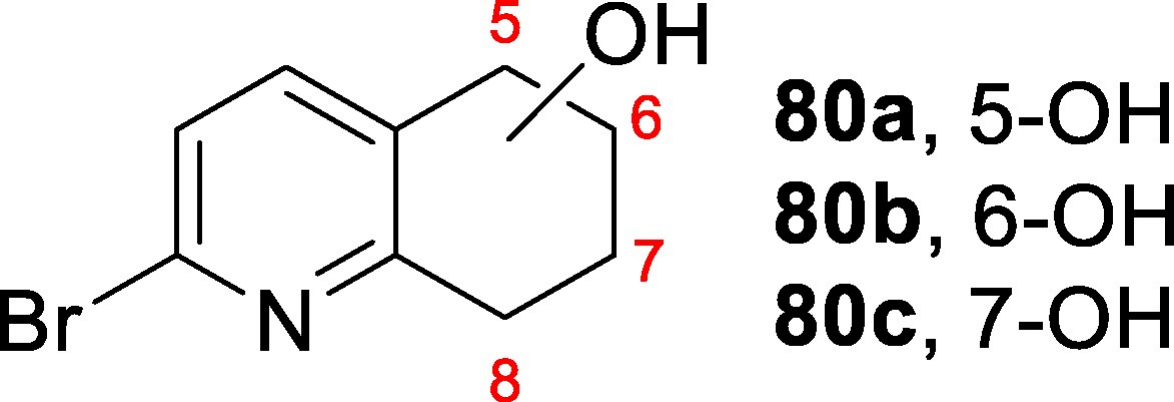
**79**			**80 a**, 53 %; **80 b**/**80 c**, 11 %/9 %
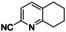	33 mg 10 mM 25 h	**B**	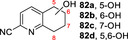
**81**			**82 a**, 24 %; **82 b**, 31 % **82 c**, 16 %; **82 d**, 7 %
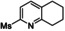	130 mg 10 mM 25 h	**B**	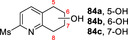
**83**			**84 a**, 43 %; **84 b**, 14 %; **84 c**, 4 %
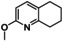	130 mg 10 mM 25 h	**B**	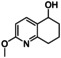
**85**			**86**, 44 %
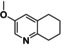	30 mg 10 mM 25 h	**B**	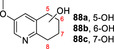
**87**			**88 a**, 19 %; **88 b**, 40 % **88 c**, 17 %
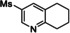	30 mg 10 mM 25 h	**B**	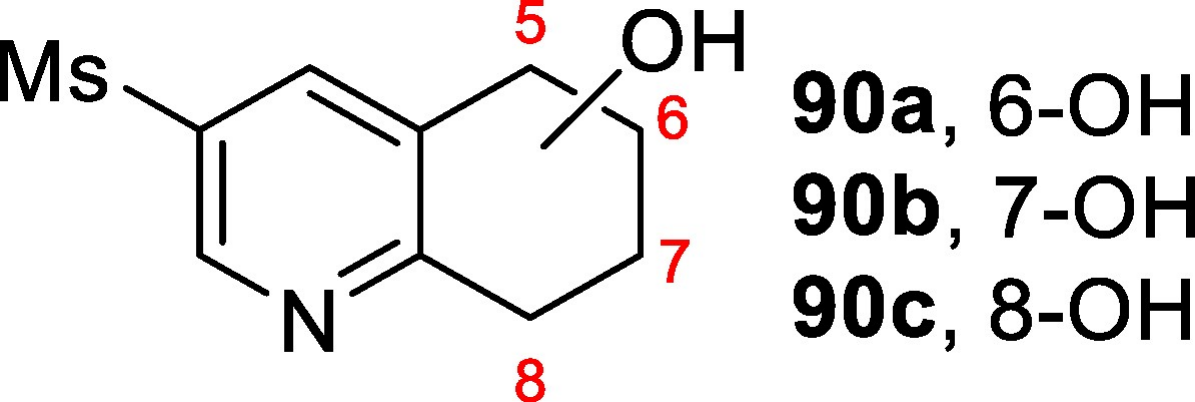
**89**			**90 a**, 42 %; **90 b**, 17 % **90 c**, 15 %

**A**: 50 mM KPi (pH=7.0), 10 % v/v MeCN, 400 mM MeOH, *Pp*AOX H_2_O_2_ generation; **B**: 5 mM KPi (pH=7.0), 10 % v/v MeCN, H_2_O_2_ slow addition; see Supporting Information Section 9 for details of both methods. *ee* was not determined in this series. Ms=CH_3_SO_2_.

gave the *N*‐oxide **44** and dihydroxy compound **78** in 34 % and 20 % yields, respectively. These results contrast with the hydroxylation of quinoline **34** by P450_BM3_ variants, which furnished only aromatic hydroxylation products.^[33]^ *N*‐oxide formation was also the major reaction pathway when methyl‐substituted isoquinoline **36** was tested, with *N*‐oxide **46** and aldehyde **47** obtained in 59 % and 12 % yield respectively.

Attention then turned to electronically diverse tetrahydroquinoline substrates. Substrates **79**, **81** and **83** (which all contain electron poor 2‐substitutents) were selectively oxygenated (with no apparent over‐oxidation to ketones), but with modest regioselectivity, forming alcohol products (**80 a**–**c**, **82 a**–**d**, **84 a**–**c**). In contrast, the analogous methoxy‐substituted substrate **85** was hydroxylated with complete regioselectivity, affording alcohol **86** as a single regioisomer. Comparable regioselectivity was not conserved when switching to 3‐substituted substrates **87** and **89**, with regioisomeric mixtures **88 a–c** and **90 a–c** formed, although the selectivity for oxygenation to form alcohol over ketone products was again high.

Finally, preparative scale indole oxygenation reactions were performed (Table 4). Indoles **48** and **49** were converted into 2‐oxindole derivatives **51** and **52** in 60 % and 75 % yield respectively. In addition, 3‐methyl‐2‐oxindole **50** was oxidized to 2‐oxindole **53** in 50 % yield, but also formed 3‐hydroxy derivative **63** in 29 % yield. This chiral alcohol product was found to be racemic, which may indicate that oxidation proceeds through epoxidation of the enol tautomer of **53** (i.e. a 2‐hydroxyindole), followed by epoxide opening to form a cation at the indole 3‐position and trapping by water, thus eroding any enantioselectivity imparted during the epoxidation.

**Table 4:** Preparative biotransformation of indoles using r*Aae*UPO‐PaDa‐I‐H.

Substrate	Scale/ time	H_2_O_2_ method	Products Isolated Yield
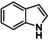	60 mg 10 mM 13 h	**B**	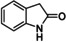
**48**			**51**, 60 %
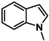	50 mg 10 mM 20 h	**A**	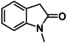
**49**			**52**, 75 %
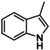	130 mg 10 mM 25 h	**B**	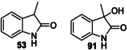
**50**			**53**, 50 %; **91**, 29 % (racemic)

**A**: 50 mM KPi (pH=7.0), 10 % v/v MeCN, 400 mM MeOH, *Pp*AOX H_2_O_2_ generation; **B**: 5 mM KPi (pH=7.0), 10 % v/v MeCN, H_2_O_2_ slow addition; see Supporting Information Section 9 for details of both methods.

## Conclusion

In summary, a lyophilized r*Aae*UPO‐PaDa‐I‐H has been shown to be an effective and promiscuous biocatalyst for the practical oxygenation of a wide range on *N*‐heterocyclic substrates, showcased by 27 preparative scale biotransformations (30–810 mg). Various reactivity/selectivity trends have been identified, isolated yields are moderate‐to‐good in most cases, and enantioselectivity is generally excellent.

These oxygenation reactions offers several advantages in terms of selectivity and sustainability over conventional chemical methods. When comparing r*Aae*UPO‐PaDa‐I‐H with established P450 oxygenations, it is true that P450s can offer superior selectivity in some cases, especially where mutant libraries give scope for improvements.[^39^, ^40^] However, this must be considered against the requirement for the nicotinamide cofactor and electron transport proteins when using P450s. The results in this report certainly suggest that UPOs offer major advantages with respect to scalability, and furthermore it is anticipated that selectivity may be improved through reaction engineering as we learn more about this enzyme class; e.g. by optimising the mode and rate of hydrogen peroxide delivery (as demonstrated herein), as well as through mutational engineering of the UPOs, which has been used to improve selectivity and performance in related biotransformations.[^29^, ^41^] The simplicity of the reaction systems overall is another advantage, as is our ability to generate large amounts of a robust, easy‐to‐use lyophilised biocatalyst. Taken together, these features all point towards an extremely promising future for UPO catalysis for use in the scalable, biocatalytic oxygenation of industrially relevant molecules.

## Conflict of interest

The authors declare no conflict of interest.

1

## Supporting information

As a service to our authors and readers, this journal provides supporting information supplied by the authors. Such materials are peer reviewed and may be re‐organized for online delivery, but are not copy‐edited or typeset. Technical support issues arising from supporting information (other than missing files) should be addressed to the authors.

Supporting Information

## Data Availability

The data that support the findings of this study are available from the corresponding author upon reasonable request.
