# Supplementary material for: Preparative‐Scale Biocatalytic Oxygenation of N‐Heterocycles with a Lyophilized Peroxygenase Catalyst
Source: Angew Chem Int Ed Engl. 2022 Dec 22;62(5):e202214759. doi: 10.1002/anie.202214759 (PMC10107140; doi:10.1002/anie.202214759)
Supplement: Supplementary file 1 — Supporting Information [file ANIE-62-0-s001.pdf]

Angewandte Chemie
Eine Zeitschrift der Gesellschaft Deutscher Chemiker

Supporting Information

Preparative-Scale Biocatalytic Oxygenation of *N*-Heterocycles with a Lyophilized Peroxygenase Catalyst

B. Pogrányi, T. Mielke, A. Díaz-Rodríguez, J. Cartwright, W. P. Unsworth, G. Grogan**

Table of Contents

1) Abbreviations	3
2) Materials and methods	4
3) Production of r*Aae*UPO-PaDa-I-H	5
4) Screening of alkylpyridines for r*Aae*UPO-PaDa-I-H-mediated oxidations	6
5) Biochemical Assays	7
6) Inactivation of r*Aae*UPO-PaDa-I-H by hydrogen peroxide	8
7) Inhibition studies using alkylpyridines	9
8) Screening of bicyclic *N*-heterocycles for r*Aae*UPO-PaDa-I-H-mediated oxidations	10
9) DoE experiments for the optimisation of hydroxylation of 6,7-dihydro-5*H*-cyclopenta[*b*]pyridine	11–14
10) Large-scale biotransformations of alkylpyridines by r*Aae*UPOPaDa-I-H	15
11) Organic Synthesis and Spectroscopic Characterization	16–64
12) Representative Gas Chromatograms of Substrate Oxidations	65–71
13) Chiral Chromatograms	72–82
14) ^{1}H and ^{13}C NMR Spectra	83–272
15) References	273–276

Section 1. Abbreviations

AcOH – Acetic acid, AcCl – Acetyl chloride, Ar, - referring to the atoms of any aromatic moiety, C.I. – Confidence interval, DAD – Diode array detector, DCM – Dichloromethane, DIPA – Diisopropylamine, DIPEA – *N*,*N*-Diisopropylethylamine, DMF –Dimethylformamide, DMSO – Dimethyl-sulphoxide, EtOAc – Ethyl-acetate, Et_2O – Diethyl-ether, EtOH – Ethanol, eq. – Equivalent, FA – Formic acid, FID – Flame ionization detector, IC_{50} – Half maximal inhibitory concentration (substrate concentration, which - under the conditions employed - inhibits the enzyme by 50%), IPA – Isopropanol, KPi buffer - Potassium phosphate buffer, MeCN – Acetonitrile, MeOH – Methanol, *m*-CPBA – meta-Chloroperoxybenzoic acid, m.p. – Melting point, NBD – 5-nitro-1,3-benzodioxole, *n*-Hex – *n*-Hexane, PAGE – Polyacrylamide gel electrophoresis, Ph – referring to the atoms of the 'phenyl moiety', PpAox – Alcohol oxidase from *Pichia pastoris* (corresponding to AOX1 gene), p-TsOH – *p*-toluenesulphonic acid, r*Aae*UPO – Recombinant UPO mutant[1] expressed in *Pichia pastoris* host, R_f – Retardation factor, rt – Room temperature, SDS – Sodium dodecyl sulphate, TEA – Triethylamine, THF – Tetrahydrofuran, TLC – Thin layer chromatography, upH_2O – ultrapure water, VA – Veratryl alcohol.

Section 2. Materials and Methods

General Techniques and Equipment

Residual solvents were removed under high vacuum. Column chromatography was performed on silica gel (VWR International, 40-63 μM mesh particle size). Analytical thin layer chromatography (TLC) was performed on Merck silica gel 60 F_{254} coated aluminium plates, the compounds were visualized under UV (wavelength 254 nm). Preparative thin layer chromatography was performed on silica gel coated glass plates, the compounds were visualized under UV (wavelength 254 nm). A Razel A 99 syringe pump was used for the slow addition of solutions. Melting points were measured on a Gallenkamp Melting Point Measuring Apparatus in capillary tubes and data are reported uncorrected. NMR measurements were taken on Jeol ECS and Bruker AV and AM spectrometers. All spectra were acquired at room temperature, unless otherwise noted. The ^{1}H and ^{13}C chemical shifts are stated on a δ internal scale in parts per million (ppm) units. Residual solvent was used as the standard. Coupling constants are reported in Hz. MS analysis was performed on a Bruker compact® time of flight mass spectrometer (ESI and APCI), GC-MS spectra were acquired on an Agilent 5975C MSD system equipped with a 60 m DB-5™ column. For GC-FID analysis, an Agilent 7890B equipped with a 30 m DB-5MS™ column was used, *ee* values were measured on the same instrument using chiral columns, as described. HPLC separations were performed on a Shimadzu Prominence and Agilent 1200 series apparatus; achiral separations were performed using a Phenomenex 4 µm Polar-RP 80 Å column (250 x 4.6 mm). UV-VIS spectra were collected on an Agilent Cary® 100 UV-VIS spectrophotometer. Infrared spectra were acquired on a Perkin Elmer Spectrum Two FT-IR Spectrometer. Optical rotation was measured on a Bellingham & Stanley ADP450 Polarimeter.

Materials

All chemicals were purchased from Sigma Aldrich (Irvine, UK), Alfa Aesar (Heysham, UK), Tokyo Chemical Industry (Oxford, UK) or Fluorochem (Hadfield, UK) and used without further purification, GPR-grade and HPLC-grade solvents were bought from VWR International (Lutterworth, UK). Anhydrous solvents were collected from a solvent tower, purchased, or dried using standard methods. Ultrapure water was produced by a Milli-Q® Integral Water Purification System.

Section 3. Production of r*Aae*UPO-PaDa-I-H

The cloning and expression of the r*Aae*UPO-PaDa-I-H and its preparation from fermentations of *Pichia pastoris* have been described previously.[1] The activity of this lyophilizate was 0.97 U mg^{-1}, as measured by the 5-nitro-1,3-benzodioxole (NBD) assay.

Section 4. Screening of alkylpyridines for r*Aae*UPO-PaDa-I-H-mediated oxidations

Method 1: *Slow addition of aqueous H_2O_2 solution*

Reactions were carried out in 10 mL round bottom flasks. The 8.8 U mL^{-1} r*Aae*UPO-PaDa-I-H solution was prepared in 2.5 mL 100 mM pH = 7.0 KPi buffer (to a final concentration of 4.4 U mL^{-1}) and it was diluted by the addition of 1 mL deionised water, followed by the addition of 500 µL of substrate solution in MeCN. The flask was sealed by a septum and the reaction was stirred at 400 rpm at rt. 0.5 mL substrate solution was added from 100 mM MeCN stock solution (final concentration 10 mM) and 1 mL 50 mM peroxide solution (10 mM final concentration, 1.0 eq) was added dropwise using a syringe pump over 25 h. Reactions were usually finished after the addition time. The reaction medium was extracted with 5 mL EtOAc three times, the combined organic phases were dried over anhydrous $MgSO_4$ and evaporated *in vacuo*. Conversions achieved this way for the various alkylpyridines were measured by GC-FID and are reported in the manuscript.

Method 2: *PpAOx-mediated H_2O_2 generation*

The reactions were carried out in 1 mL total volume in 6 mL glass vials. 9.1 mg lyophilized expression supernatant - containing 4.4 U r*Aae*UPO-PaDa-I-H - were dissolved in 500 µL 100 mM pH = 7.0 KPi buffer and diluted by the addition of 359 µL deionised water. Stock solutions of the commercially available *Pp*AOx were prepared by dilution with 100 mM pH = 7.0 KPi buffer and 25 µL was added to each reaction (to reach 0.3, 1, and 3 U mL^{-1} final activities, respectively), followed by the addition of 100 µL 100 mM MeCN stock solution of the substrate (10 mM final concentration). Reactions were started by the addition of 16.2 µL MeOH (400 mM final concentration). Reaction vessels were shaken at 700 rpm at rt for 12-24 h, based on the progression of the reaction. Reaction media were extracted with 2 x 2 mL of EtOAc, the organic phases were combined and dried over $MgSO_4$. Analyses were performed using GC-FID. Conversions are reported in the manuscript.

Section 5. Biochemical Assays

5-Nitro-1,3-benzodioxole (NBD) Assay

NBD assays were performed by dissolving the lyophilized supernatant of rAaeUPO-PaDa-I-H in 500 µL 100 mM KPi buffer (pH = 7.0), which was subsequently diluted to 880 µL volume by the addition of upH_2O. 100 µL of 10 mM MeCN solution of NBD was added (to a final concentration of 1 mM), followed by the addition of 20 µL 100 mM aqueous H_2O_2 solution (final concentration of 2 mM). The absorbance was measured at 420 nm and relative activities were calculated from the initial linear part of the absorbance-time curve.

Veratryl Alcohol (VA) Assay

The lyophilized supernatant of r*Aae*UPO-PaDa-I-H was dissolved in 500 µL 100 mM KPi buffer (pH = 7.0), then 380 µL upH_2O was added. 100 µL 10 mM MeCN stock solution of veratryl alcohol was added to the mixture (final concentration 1 mM) and the reaction was started by the addition of 20 µL 100 mM H_2O_2 in H_2O (final concentration 2 mM). The reaction kinetics were followed by measuring the absorbance at 310 nm. The relative activities could be calculated from the initial reaction rate using veratryl aldehyde's attenuation coefficient. The assay has been found to perform well above ~0.1 U mL^{-1} rAaeUPO-PaDa-I-H level.

Section 6. Inactivation of r*Aae*UPO-PaDa-I-H by hydrogen peroxide.

The sensitivity of r*Aae*UPO-PaDa-I-H to H_2O_2 is highlighted by **Figure S1**.

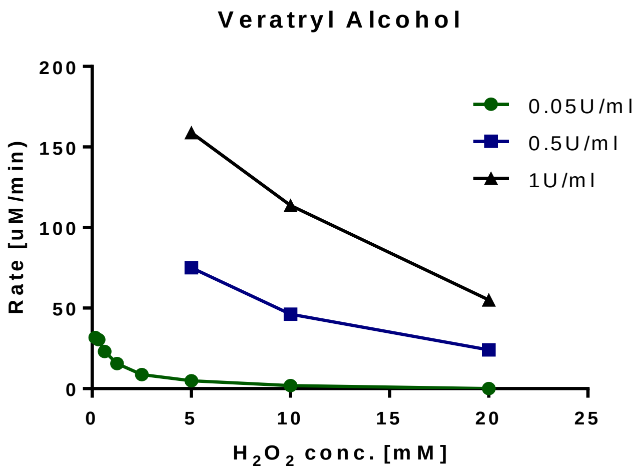

Figure S1. Inactivation of r*Aae*UPO-PaDa-I-H by hydrogen peroxide using veratryl alcohol as a substrate. The assay was performed and monitored as described in **Section 5.**

Section 7. Inhibition studies using alkylpyridines

Method

The NBD assay was validated in a stopped assay format, as this made the accurate termination of the reactions prior to readout possible. The measurements were performed in 96 well plates. 60 µL of r*Aae*UPO-PaDa-I-H stock solution in 50 mM pH = 7.0 KPi buffer (1.3 U mL^{-1} final concentration) and 7.5 µL 6 mM NBD MeCN solution (0.3 mM final concentration) were added to each well, followed by the addition of 7.5 µL competing substrate solution in MeCN. The reactions were started by the addition of 75 µL 4 mM H_2O_2 solution (2 mM final concentration). Reactions were stopped after 10, 20 and 30 s by the addition of 150 µL 10 mM NaN_3 solution in Milli-Q water. Water was used instead of H_2O_2 solution and MeCN instead of substrate solution in the case of negative and positive controls, respectively. Plate readings were carried out within 30 min. Measurements were performed in three technical replicates, n = 3. Binding affinities (Table S1) were calculated using the Cheng-Prusoff equation, assuming competitive binding of NBD and the substrate in question.[2] K_d stands for dissociation constant.

Table S1. Binding affinities of alkyl pyridine substrates with r*Aae*IUPO-PaDa-I-H.

Substrate	IC_{50} (mM)	95% C.I.	K_d (mM)
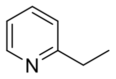	**29.5 mM**	**18.7 – 46.5**	**15.8 mM**
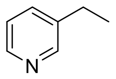	**0.70 mM**	**0.58 – 0.86**	**0.39 mM**
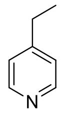	**0.016 mM**	**0.012 – 0.020 mM**	**0.008 mM**

Section 8. Screening of bicyclic *N*-heterocycles for r*Aae*UPO-PaDa-I-H-mediated oxidations

Method 1: *Slow* H_2O_2 *addition*

An identical method was used to that described in **Section 4**. Results of the screen are reported in the manuscript.

Method 2: *PpAOx-mediated* H_2O_2 *generation*

The reactions were carried out in an identical manner to the one described in **Section 4**. Results of the screen are reported in the manuscript.

The methods used for chromatographic separation are described under SI **Section 12**.

Section 9. DoE experiments for the optimisation of hydroxylation of 6,7-dihydro-5*H*-cyclopenta[*b*]pyridine 1

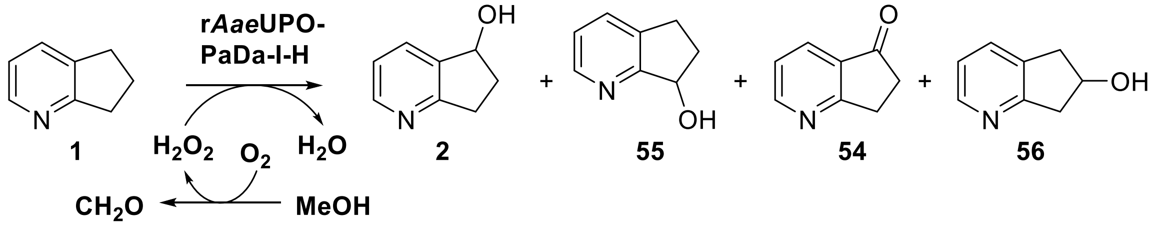

Scheme S1 r*Aae*UPO-PaDa-I-H catalyzed conversion of 6,7-dihydro-5*H*-cyclopenta[b]pyridine **1** in the presence of *Pp*AOx and MeOH.

To examine the factors influencing the oxidation of 6,7-dihydro-5*H*-cyclopenta[*b*]pyridine **1** by r*Aae*UPO-PaDa-I-H employing *Pp*AOx for the generation of H_2O_2 *in situ*, a design of experiments (DoE) approach was used. The Design Expert software was used to analyse the results. The UPO concentration, UPO/*Pp*AOx ratio, substrate loading, and the temperature were chosen as factors, whilst the conversion to product and by-products as responses.

Integrity10 reaction station (Electrothermal, Stone, UK) was used for preparing and running the reactions in 10 mL total volume. The experimental conditions are given in **Table S2**. All reactions were run in 50 mM KP_i buffer at pH 7.0 (from 100 mM stock) in the presence of 200 mM MeOH and 10% MeCN. Glass tubes were charged with a stirrer bar and the reactor block containing these vessels was heated to the desired temperature prior to the addition of MeOH to initiate the reactions. Samples were taken by diluting reaction media with MeCN to yield uniform concentrations (i.e. samples with 50 mM substrate were diluted 10 times and 5 mM samples were not diluted at all).

Table S2. Factors and space types used throughout the experiments, f stands for factorial and c for centre, n = 10.

			Factor 1	Factor 2	Factor 3	Factor 4	
**Run**	**Std.**	**Space Type**	**Substrate Conc.**	**T**	**UPO Level**	**UPO:*Pp*AOx**	***Pp*AOx Level**
1	3	f	5 mM	40 °C	1 U mL^{-1}	20	0.05 U mL-1
2	4	f	50 mM	40 °C	1 U mL^{-1}	5	0.2 U mL^{-1}
3	6	f	50 mM	20 °C	10 U mL^{-1}	5	2 U mL^{-1}
4	10	c	27.5 mM	30 °C	5.5 U mL^{-1}	12.5	0.44 U mL^{-1}
5	1	f	5 mM	20 °C	1 U mL^{-1}	5	0.2 U mL^{-1}
6	5	f	5 mM	20 °C	10 U mL^{-1}	20	0.5 U mL^{-1}
7	2	f	50 mM	20 °C	1 U mL^{-1}	20	0.05 U mL^{-1}
8	8	f	50 mM	40 °C	10 U mL^{-1}	20	0.5 U mL^{-1}
9	7	f	5 mM	40 °C	10 U mL^{-1}	5	2 U mL^{-1}
10	9	c	27.5 mM	30 °C	5.5 U mL^{-1}	12.5	0.44 U mL^{-1}

After 20 h the reaction mixture was extracted with ethyl acetate (2 x 10 mL), the combined organic phases dried over anhydrous $MgSO_4$, filtered and evaporated *in vacuo*. Results are collected in **Table S3**.

Table S3: Responses of the 10 DoE runs, with the GC-derived yields for the desired 5-hydroxyl **2** and the non-desired 5-oxo **54**, and 6- **56**, and 7-hydroxyl **55** side products.

	Response 1	Response 2	Response 3	Response 4	Response 5
Run	Conversion (GC)		Isolated crude	Extracted Conversion	
	to **2**	To **54-56**	**2**, **54-56**	to **2**	to **54-56**
1	24%	6%	74%	78%	16%
2	1%	0%	41%	2%	0%
3	35%	8%	41%	42%	11%
4	26%	6%	29%	45%	9%
5	73%	22%	100%	72%	24%
6	66%	34%	100%	73%	20%
7	7%	1%	49%	10%	2%
8	2%	0%	34%	2%	0%
9	73%	25%	74%	70%	27%
10	29%	7%	31%	55%	13%

The contribution of the effects and cross-effects of the factors listed in **Table S2** were ranked by the software using a least square estimation. In all cases the biggest influence on the responses was the substrate concentration (likely either due to substrate inhibition, or by affecting phase separation and precipitation). The factor ranked next was the UPO concentration, which unsurprisingly increased the amount of both the desired **2** product and of the side products. The selectivity between the 5- and 7-positions (**2**, **55**) remained similar, the amount of the 5-oxo **54** compound increased, as a result of the higher enzyme concentration, however. Further significant factors determined in the half-normal plots were the temperature, the UPO to *Pp*AOx ratio, and cross-effects between various factors (see **Figure S2A-B**).

Using the factor-response relationship data collected, an overlay plot was created by plotting the enzyme ratio (UPO/*Pp*Aox) against substrate concentration. The adjustment of temperature and overall UPO loading then allowed the identification of a window with conditions that satisfied the criteria that the conversion after extraction be greater than 50 % and the by-product formation after extraction be less than 15 % (**Figure S2C**).

Based on the results thus obtained, 40 mM substrate concentration, 20 °C as temperature, 10 U mL^{-1} r*Aae*UPO-PaDa-I-H and 2 U mL^{-1} *Pp*AOx concentrations were deemed to be the best conditions.

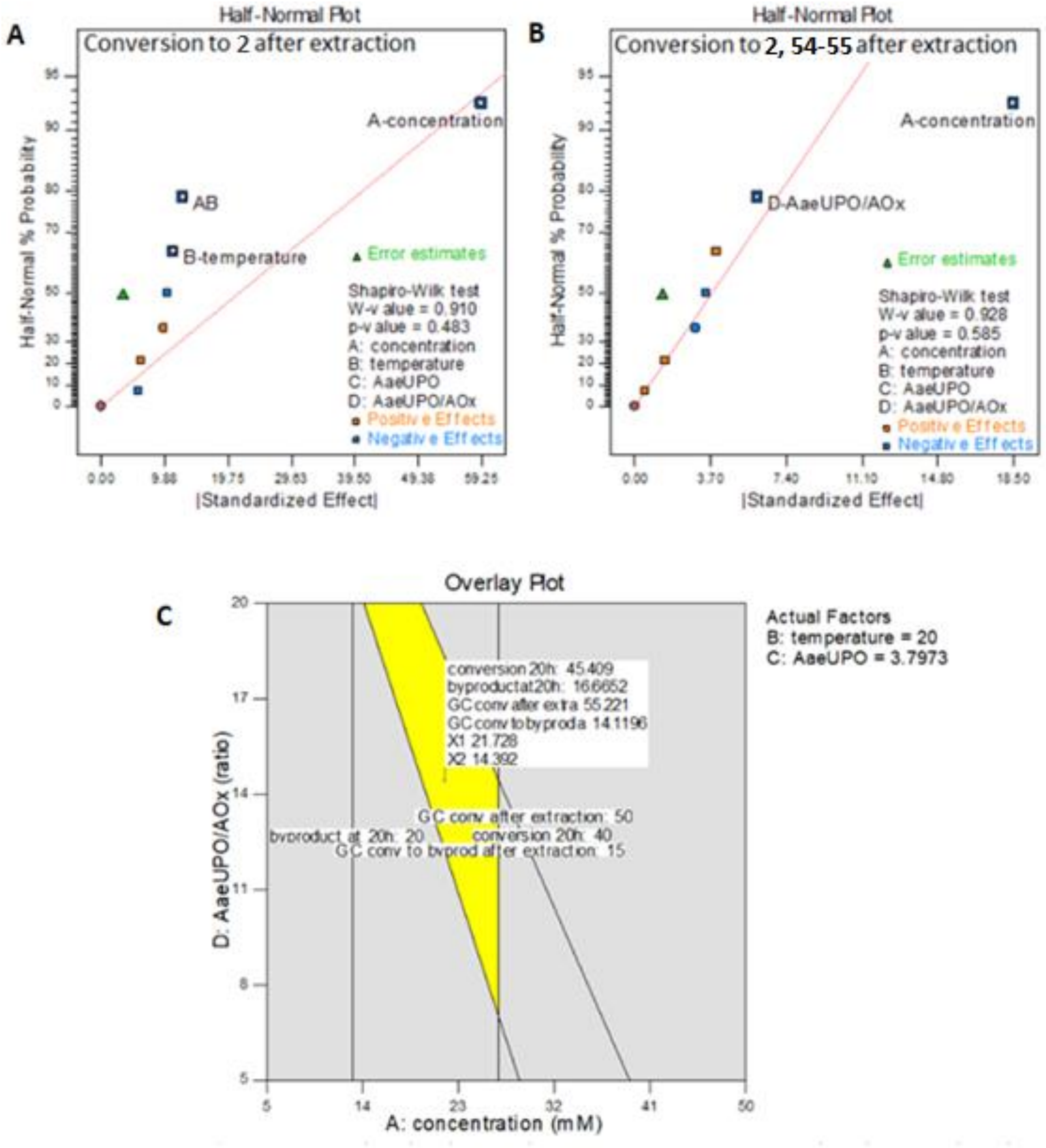

Figure S2 A-B: Half-Normal plots representing the influences of the different factors on the conversion of the stating material to the its 5-hydroxy derivative **2** after extraction (**A**), and the total conversion for by-products (**B**). **Figure S2C** Overlay plot adjusted to optimize the yield of **2** versus that of the side-products.

Section 10. Large-scale biotransformations of alkylpyridines by r*Aae*UPOPaDa-I-H

Method A: *In situ* H_2O_2 *generation using PpAOX*

After optimal reaction conditions had been identified, scale-up trials were performed and conversion was found to be robust, provided that the headspace was at least twice of the reaction volume, and the reactions were performed in glass vessels. Scale-up reactions could be performed both in round bottom and conical flasks, the former employing magnetic stirring at 500 rpm, whilst the latter was shaken at 200 rpm in an orbital shaker (see individual experiments). Scale-up was attempted stepwise, increasing the reaction volume threefold during every step. The conditions were usually robust enough to give similar yields to small scale experiments up to 50 mL reaction volume.

Method B: H_2O_2 *Slow addition using syringe pump*
Slow addition reactions were performed in round-bottomed flasks in every case with magnetic stirring at 500 rpm. The molar addition rate of H_2O_2 had to be rigorously kept at the same level than in the case of the small-scale reactions to achieve identical conversions and yields. The conditions used and the scale of the reactions are reported in the manuscript.

Section 11. Synthesis and Spectroscopic Characterization

Synthesis of Authentic Standards

6,7-dihydro-5H-cyclopenta[b]pyridin-5-one **54**

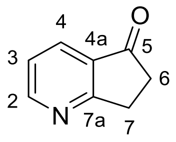

2,3-Cyclopentenopyridine 1 (980 µL, 1.00 g, 8.39 mmol, 1.00 eq.) was dissolved in distilled H_2O (50.0 mL) and $MnSO_4 \cdot H_2O$ (14.0 mg, 0.084 mmol, 0.010 eq.) was added. Aqueous *tert*-butylhydroperoxide solution was added (70 % m/m, 6.21 mL, 45.3 mmol, 5.40 eq.) to the solution at rt and the reaction mixture was stirred at this temperature for 20 h. The colour of the reaction turned brown during this time. Additional aqueous *tert*-butylhydroperoxide solution was added (70 % m/m, 10.0 mL, 72.9 mmol, 8.69 eq.) and the reaction was stirred at rt for an additional 36 h. The reaction was cooled to 0 °C and quenched by the addition of solid $Na_2S_2O_5$ (16.0 g, 83.9 mmol, 10.0 eq.). The precipitate was then filtered and the filtrate was extracted with EtOAc (3 x 100 mL). The unified organic phase was washed with brine (30 mL), dried over anhydrous $MgSO_4$, filtered and evaporated under reduced pressure to yield the crude material as brown oil. The crude product was taken into the next step without further purification. Yield: 551 mg (49%). R_f = 0.11 (eluent: *n*-Hex:EtOAc = 7:3).

^{1}H NMR (400 MHz, $CDCl_3$) δ 8.81 (dd, *J* = 4.8, 1.5 Hz, 1H, 2-H_{Ar}), 8.03 (dd, *J* = 7.7, 1.5 Hz, 1H, 4-H_{Ar}), 7.33 (dd, *J* = 7.7, 4.8 Hz, 1H, 3-H_{Ar}), 3.34 - 3.24 (m, 2H, 7-H_α, 7-H_β), 2.87 - 2.77 (m, 2H, 6-H_α, 6-H_β). ^{13}C NMR (101 MHz, $CDCl_3$) δ 205.2 (5-C), 174.5 (7a-C_{Ar}), 155.9 (2-C_{Ar}), 132.1 (4-C_{Ar}), 130.5 (4a-C_{Ar}), 122.7 (3-C_{Ar}), 35.9 (6-C), 28.9 (7-C). HRMS (ESI, m/z) m/z calculated for C_8H_7NO [M+H$^+$] 134.0605, found 134.0600. The data match those reported in the literature.[3]

*(+/-)-6,7-Dihydro-5H-cyclopenta[b]pyridin-5-ol rac-***2**

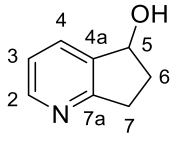

6,7-Dihydro-5*H*-cyclopenta[*b*]pyridin-5-one 54 (10.0 mg, 80.0 µmol, 1.00 eq.) was dissolved in MeOH (1 mL) and $NaBH_4$ (4.30 mg, 130 µmol, 1.50 eq.) was added in 3 portions to the mixture at rt. The reaction was stirred at rt for 3 h, and it was poured onto water (20 mL) and the aqueous phase was extracted with EtOAc (3 x 10 mL). The unified organic phases were washed with brine, dried over anhydrous $MgSO_4$, filtered, and evaporated *in vacuo*. The crude product was purified by column chromatography (eluent: DCM/MeOH = 9:1) to yield the title compound as pale-yellow oil. R_f = 0.28 (eluent: DCM/MeOH = 9:1). Yield: 8.0 mg (79%).

^{1}H NMR (400 MHz, $CDCl_3$) δ 8.62–8.31 (m, 1H, 2-H_{Ar}), 7.71 (d, *J* = 7.6 Hz, 1H, 4 - H_{Ar}), 7.15 (dd, *J* = 7.6, 2.5 Hz, 1H, 3-H_{Ar}), 5.29 (t, *J* = 6.5 Hz, 1H, 5-H), 3.16 (ddd, *J* = 16.8, 8.9, 4.4 Hz, 1H, 7-H_α), 2.94 (dt, *J* = 16.8, 7.9 Hz, 1H, 7-H_β), 2.66 - 2.50 (m, 1H, 6-H_α), 2.07 - 1.91 (m, 2H, 6-H_β, -O*H*). ^{13}C NMR (101 MHz, $CDCl_3$) δ 164.3 (7a-C_{Ar}), 149.9 (2-C_{Ar}), 137.9 (4a-C_{Ar}), 132.6

(4-C_{Ar}), 121.9 (3-C_{Ar}), 74.6 (5-C), 34.5 (6-C), 31.9 (7-C). HRMS (ESI, m/z) m/z calculated for C_8H_9NO [M+H$^+$] 136.0757, found 136.0762.

*6,7-Dihydro-5*H-*cyclopenta[*b*]pyridine 1-oxide* **S1**

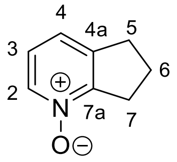

2,3-Cyclopentenopyridine (250 µL, 250 mg, 2.10 mmol, 1.00 eq.) was dissolved in DCM (5.0 mL) and *m*-CPBA (400 mg, 2.31 mmol, 1.10 eq.) was added to the reaction in one portion. After 3 hours, additional *m*-CPBA was added (140 mg, 0.81 mmol, 0.39 eq.) in one portion and the reaction was stirred at room temperature for 24 h. The reaction was poured onto aqueous saturated $NaHCO_3$ solution (10 mL) and it was extracted with 70/30 v/v % mixture of $CHCl_3$ and IPA (3 x 20 mL). The unified organic phase was dried over anhydrous $MgSO_4$, filtered and evaporated under reduced pressure to yield the crude material as white solid (400 mg). The crude contained *m*-chlorobenzoic acid, but it was taken into the next step without further purification. R_f = 0.08 (eluent: EtOAc:*n*-Hex = 9:1).

^{1}H NMR (400 MHz, $CDCl_3$) δ 8.08 (d, *J* = 6.3 Hz, 1H, 2-H_{Ar}), 7.14 (d, *J* = 7.6 Hz, 1H, 4-H_{Ar}), 7.07 (dd, *J* = 7.6, 6.3 Hz, 1H, 3-H_{Ar}), 3.18 (t, *J* = 7.7 Hz, 2H, 5-H_α, 5-H_β), 3.01 (t, *J* = 7.6 Hz, 2H, 7-H_α, 7-H_β), 2.17 (p, *J* = 7.6 Hz, 2H, 6-H_α, 6-H_β).). ^{13}C NMR (101 MHz, $CDCl_3$) δ 153.5 (7a-C_{Ar}), 142.5 (2-C_{Ar}), 137.5 (4a-C_{Ar}), 124.0 (3-C_{Ar}), 123.5 (4-C_{Ar}), 31.7 (5-C_{Ar}), 29.7 (7-C_{Ar}), 22.1 (6-C_{Ar}). The data match those reported in the literature.[4]

*6,7-Dihydro-5*H-*cyclopenta[b]pyridin-7-yl trifluoromethanesulfonate* **S2**

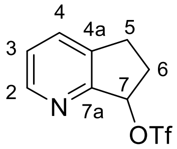

Crude **S1** (274 mg, 2.03 mmol (according to ^{1}H NMR), 1.00 eq.) was dissolved in DCM (15.0 mL) and Tf_2O (706 µL, 1.06 g, 5.08 mmol, 2.50 eq.) was added to the solution dropwise over 20 min at 0 °C. The reaction was stirred at room temperature overnight. The reaction media was neutralised by the addition of aqueous saturated $NaHCO_3$ solution and the layers were separated. The aqueous layer was further extracted with EtOAc (3 x 30 mL). The unified organic phase was dried over anhydrous $MgSO_4$, filtered and evaporated *in vacuo* to yield the crude material as brown oil (301 mg). The crude was taken into the next step without further purification.

^{1}H NMR (400 MHz, $CDCl_3$) δ 8.53 (dd, *J* = 4.7, 1.3 Hz, 1H, 2-H_{Ar}), 7.65 (dd, *J* = 7.8, 1.3 Hz, 1H, 4-H_{Ar}), 7.28 – 7.22 (dd, 7.8, 4.7 Hz, 1H, 3-H_{Ar}), 6.35 (dd, *J* = 7.5, 4.2 Hz, 1H, 7-H_{Ar}), 3.21 – 3.11 (m, 1H, 5-H_α), 3.04 (d, *J* = 7.6 Hz, 1H, 5-H_β), 2.72 – 2.62 (m, 1H, 6-H_α), 2.22 – 2.15 (m, 1H, 6-H_β). ^{13}C NMR (101 MHz, $CDCl_3$) δ 158.2 (7a-C_{Ar}), 149.3 (2-C_{Ar}), 138.1 (4a-C_{Ar}), 133.6 (4-C_{Ar}), 124.3 (3-C_{Ar}), 81.2 (7-C_{Ar}), 30.1 (5-C_{Ar}), 28.0 (6-C_{Ar}).

*(+/-)-6,7-dihydro-5*H*-cyclopenta[b]pyridin-7-ol rac*-**55**

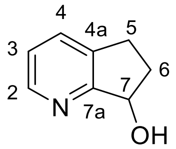

Crude **S2** (301 mg) was dissolved in DCM (10 mL) and stirred with aqueous LiOH solution (20 mL, 1.0 M) for 48 h at rt. The volatiles were removed under reduced pressure and the aqueous phase was extracted with EtOAc (3 x 30 mL). The unified organic phase was dried over anhydrous $MgSO_4$, filtered and evaporated *in vacuo* to yield the crude as brown oil (155 mg). The crude was purified by column chromatography (eluent: EtOAc) to yield the product as pale yellow solid. R_f = 0.18 (eluent: EtOAc). Yield: 77 mg (27% calculated for the three steps).

^{1}H NMR (400 MHz, $CDCl_3$) δ 8.42 (dd, *J* = 4.9, 1.4 Hz, 1H, 2-H_{Ar}), 7.57 (dd, *J* = 7.7, 1.3 Hz, 1H, 4-H_{Ar}), 7.14 (dd, *J* = 7.7, 4.9 Hz, 1H, 3-H_{Ar}), 5.26 (dd, *J* = 7.5, 6.0 Hz, 1H, 7-H), 3.11 – 2.98 (m, 1H, 5-H_α), 2.86 – 2.74 (m, 1H, 5-H_β), 2.55 (dddd, *J* = 13.3, 8.4, 7.5, 4.2 Hz, 1H, 6-H_α), 2.07 (dddd, *J* = 13.3, 8.9, 7.1, 6.0 Hz, 1H, 6-H_β). ^{13}C NMR (101 MHz, $CDCl_3$) δ 165.1 (7a-C_{Ar}), 147.9 (2-C_{Ar}), 136.7 (4a-C_{Ar}), 133.6 (4-C_{Ar}), 122.9 (3-C_{Ar}), 74.4 (7-C), 32.9 (6-C), 27.6 (5-C). HRMS (ESI, m/z) m/z calculated for C_8H_9NO [M+H$^+$] 136.0757, found 136.0761. The data match those reported in the literature.[4]

(+/-)-5,6,7,8-Tetrahydroquinoline-5-ol rac-**4**

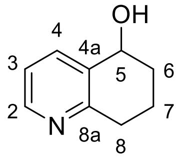

5,6,7,8-Tetrahydroquinoline-5-one (500 mg, 3.39 mmol, 1.00 eq.) was dissolved in MeOH (10 mL) and $NaBH_4$ (190 mg, 5.10 mmol, 1.50 eq.) was added to the mixture at rt. The reaction was stirred at rt for 1 h. The reaction mixture was then poured onto saturated $NaHCO_3$ solution (60 mL) and the aqueous phase was extracted with EtOAc (3 x 30 mL). The combined organic phase was dried over anhydrous $MgSO_4$, filtered, and evaporated under *vacuo*. The crude product was used directly without further purification. The product was obtained as a transparent oil, which slowly crystallized at rt. R_f = 0.11 (eluent: n-Hex/EtOAc = 1:2). m.p.: 84–85 °C. Yield: 0.42 g (83%).

^{1}H NMR (400 MHz, $CDCl_3$) δ 8.39 (dd, *J* = 4.6, 1.3 Hz, 1H, 2-H_{Ar}), 7.77 (dd, *J* = 7.6, 1.3 Hz, 1H, 4-H_{Ar}), 7.13 (dd, *J* = 7.6, 4.6 Hz, 1H, 3-H_{Ar}), 4.84–4.78 (m, 1H, 5-H), 3.04 - 2.79 (m, 2H, 8-H_α, 8-H_β), 2.50 (s, 1H, -CH-O*H*), 2.13–1.97 (m, 2H, 6-H_α, 7-H_α), 1.92–1.77 (m, 2H, 6-H_β, 7-H_β). ^{13}C NMR (101 MHz, $CDCl_3$) δ 157.2 (8a-C_{Ar}), 148.6 (2-C_{Ar}), 136.5 (4-C_{Ar}), 134.6 (4a-C_{Ar}), 121.6 (3-C_{Ar}), 68.0 (5-C), 32.3 (6-C), 32.3 (8-C), 18.9 (7-C). HRMS (ESI, m/z) m/z calculated for $C_9H_{11}NO$ [M+H$^+$] 150.0919, found 150.0911. The data match those reported in the literature.[5]

3-Propylpyridine **11**

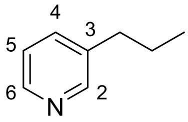

Mg turnings (1.80 g, 74.4 mmol, 6.00 eq.) were added to a round bottomed flask and activated with catalytic amount of I_2 and the turnings were then suspended in dry THF (35 mL) under Ar atmosphere. 1-Bromopropane (9.81 g, 7.21 mL, 79.8 mmol, 6.50 eq.) was added dropwise to the mixture and the reaction was stirred for an additional 5 min at rt. Meanwhile, 3-bromopyridine (1.96 g, 1.19 mL, 12.4 mmol, 1.00 eq.) and Fe(acac)$_3$ (330 mg, 0.930 mmol, 0.0750 eq.) were dissolved in dry THF (50 mL) in a separate flask and the solution was cooled to –20 °C. The freshly prepared propyl-magnesium bromide solution was then added dropwise to the –20 °C cooled reaction mixture over 15 min. The reaction was stirred at this temperature for another 30 min, during which time the colour of the solution changed from red to black. The reaction was quenched with 0.5 M aqueous citric acid solution (100 mL, 50 mmol), followed by neutralization with 0.5 M Na_2CO_3 solution (100 mL). The reaction medium was extracted with EtOAc (3 x 100 mL), the combined organic phase was dried over anhydrous $MgSO_4$, filtered, and evaporated i*n vacuo*. The crude material was purified by column chromatography (eluent: *n*-Hex/EtOAc = 1:1) to yield 3-propylpyridine as a brown oil. R_f = 0.56 (eluent: *n*-Hex/EtOAc = 1:1). Yield: 0.90 g (60%).

^{1}H NMR (400 MHz, $CDCl_3$) δ 8.43 (d, *J* = 1.7 Hz, 1H, 2-H_{Ar}), 8.43 (dd, *J* = 4.6, 1.7 Hz, 1H, 6-H_{Ar}), 7.48 (dt, *J* = 7.7, 1.7 Hz, 1H, 4-H_{Ar}), 7.19 (dd, *J* = 7.7, 4.6 Hz, 1H, 5-H_{Ar}), 2.58 (t, *J* = 7.3 Hz, 2H, Py-C*H*$_2$-), 1.64 (sext, *J* = 7.3 Hz, 2H, -CH_2-C*H*$_2$-CH_3), 0.94 (t, *J* = 7.3 Hz, 3H, -CH_2-CH_2-C*H*$_3$). ^{13}C NMR (101 MHz, $CDCl_3$) δ 150.2 (6-C_{Ar}), 147.4 (2-C_{Ar}), 137.8 (3-C_{Ar}), 136.0 (4-C_{Ar}), 123.3 (5-C_{Ar}), 35.2 (Py-*C*H_2-), 24.4 (-*C*H_2-CH_3), 13.8 (-CH_2-*C*H_3). HRMS (ESI, m/z) m/z calculated for $C_8H_{11}N$ [$M+H^+$] 122.0970, found 122.0959. The data match those reported in the literature.[6]

3-Isopropylpyridine **12**

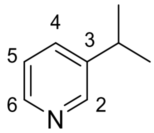

3-Bromopyridine (1.00 g, 0.62 mL, 6.3 mmol, 1.0 eq.) and Fe(acac)$_3$ (0.170 g, 0.470 mmol, 0.074 eq.) were dissolved in dry THF and the solution was subsequently cooled to –20 °C. 2 M Isopropyl-magnesium bromide THF solution (5.38 mL, 10.8 mmol, 1.70 eq.) was added to the solution dropwise over 15 min. The reaction was stirred at this temperature for another 30 min, during which time the colour of the solution changed from red to black. The reaction was quenched with 30 mL 0.5 M citric acid solution (15 mmol), followed by neutralization with 30 mL 0.5 M Na_2CO_3 solution. The reaction medium was extracted with EtOAc (3 x 100 mL), the combined organic phase was dried over anhydrous $MgSO_4$, filtered, and evaporated in *vacuo*. The crude material was purified by column chromatography (eluent: *n*-Hex/EtOAc = 1:1) to yield 3-isopropylpyridine as a yellow oil. R_f = 0.53 (eluent: *n*-Hex/EtOAc = 1:1). Yield: 0.38 g (50%).

^{1}H NMR (400 MHz, $CDCl_3$) δ 8.49 (d, *J* = 2.4 Hz, 1H, 2-H_{Ar}), 8.43 (dd, *J* = 4.5, 2.4 Hz, 1H, 6-H_{Ar}), 7.53 (dt, *J* = 8.0, 2.4 Hz, 1H, 4-H_{Ar}), 7.21 (dd, *J* = 8.0, 4.5 Hz, 1H, 5-H_{Ar}), 2.93 (sept, *J* = 7.9 Hz, 1H, -C*H*-), 1.28 (s, 3H, -C*H*$_3$), 1.26 (s, 3H, -C*H*$_3$). ^{13}C NMR (101 MHz, $CDCl_3$) δ 148.8 (6-C_{Ar}), 147.5 (2-C_{Ar}), 143.8 (3-C_{Ar}), 133.8 (5-C_{Ar}), 123.5 (4-C_{Ar}), 31.9 (-*C*H-), 23.8 (-*C*H3). HRMS (ESI, m/z) m/z calculated for $C_8H_{11}N$ [M+H$^+$] 122.0970, 122.0963 found. The data match those reported in the literature.[7]

(+/-)-1-(3-Pyridyl)-ethanol rac-**20**

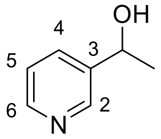

3-Acetylpyridine **21** (55.0 mg, 0.450 mmol, 1.00 eq.) was dissolved in MeOH (5 mL) and $NaBH_4$ (25.8 mg, 0.680 mmol, 1.50 eq.) was added to the mixture at rt. The reaction was stirred at this temperature overnight. The reaction mixture was poured onto water (20 mL) and the aqueous phase was extracted with EtOAc (3 x 10 mL). The unified organic phase was dried over anhydrous $MgSO_4$, filtered, and evaporated under *vacuo*. The crude product was used directly as a standard. The product is a pale yellow oil. R_f = 0.39 (eluent: EtOAc/*n*-Hex = 4:1). Yield: 52.6 mg (95%).

^{1}H NMR (400 MHz, $CDCl_3$) δ 8.48 (s, 1H, 2-H_{Ar}), 8.40 (d, *J* = 4.8 Hz, 1H, 6-H_{Ar}), 7.75 (d, *J* = 8.2 Hz, 1H, 4-H_{Ar}), 7.29 (dd, *J* = 8.2, 4.8 Hz, 1H, 5-H_{Ar}), 4.93 (q, *J* = 6.7 Hz, 1H, -C*H*(OH)-CH_3), 3.60 (broad s, 1H, -O*H*), 1.51 (d, *J* = 6.9 Hz, 3H, -C*H*$_3$). ^{13}C NMR (101 MHz, $CDCl_3$) δ 148.3 (2-C_{Ar}), 147.2 (6-C_{Ar}), 141.7 (3-C_{Ar}), 133.6 (4-C_{Ar}), 123.7 (5-C_{Ar}), 67.7 (-*C*H(OH)-), 25.3 (-*C*H$_3$). HRMS (ESI, m/z) m/z calculated for C_7H_9NO [M+H$^+$] 124.0762, found 124.0756. The data match those reported in the literature.[8]

(+/-)-1-(2-Pyridyl)-ethanol rac-**22**

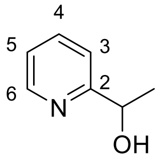

2-Acetylpyridine **23** (100 mg, 0.830 mmol, 1.00 eq.) was dissolved in MeOH (3 mL) and $NaBH_4$ (46.8 mg, 1.24 mmol, 1.50 eq.) was added in 3 portions to the mixture at rt. After 1h, the reaction mixture was poured onto deionised water (20 mL) and the aqueous phase was extracted with EtOAc (3x10 mL). The combined organic phase was washed with brine, dried over anhydrous $MgSO_4$, filtered, and evaporated *in vacuo*. The crude product was purified by column chromatography (eluent: EtOAc). The product is a dense, transparent oil. R_f = 0.15 (eluent: *n*-Hex/EtOAc = 5:1, 0.5 % TEA). Yield: 97 mg (95%).

^{1}H NMR (400 MHz, $CDCl_3$) δ 8.54 (d, *J* = 4.9 Hz, 1H, 6-H_{Ar}), 7.69 (t, *J* = 7.7 Hz, 1H, 4-H_{Ar}), 7.27 (d, *J* = 7.7 Hz, 1H, 3-H_{Ar}), 7.20 (dd, *J* = 7.7, 4.9 Hz, 1H, 5-H_{Ar}), 4.89 (qd, *J* = 6.5, 4.1 Hz, 1H, -C*H*-OH), 4.28 (d, *J* = 4.1 Hz, 1H, -O*H*), 1.51 (d, *J* = 6.5 Hz, 3H, -C*H*$_3$). ^{13}C NMR (101 MHz, δ) 148.4 (6-C_{Ar}), 137.0 (4-C_{Ar}), 122.5 (5-C_{Ar}), 120.1 (3-C_{Ar}), 69.0 (-*C*H-OH), 24.5 (-*C*H$_3$). HRMS (ESI, m/z) m/z calculated for C_7H_9NO [M+H$^+$] 124.0762, found 124.0756. The data match those reported in the literature.[9]

2-(3-Pyridyl)-2-propanol **24**

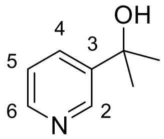

3-Acetylpyridine **21** (1.21 g, 10.0 mmol, 1.00 eq.) was dissolved in dry Et_2O (25 mL) and 3.0 M ethereal MeMgBr solution (3.60 mL, 11.0 mmol, 1.10 eq.) was added to the mixture dropwise at 0 °C, over 20 min. The reaction was left to warm to rt and stirred at this temperature for 1 h. H_2O (25 mL) was added, followed by 1.5 mL of concentrated HCl solution. The layers were separated and the aqueous layer was extracted with EtOAc (3x20 mL). The unified organic phase was dried over anhydrous $MgSO_4$, filtered and evaporated *in vacuo*. The yielded yellow oil (0.89 g) was purified by column chromatography (eluent: *n*-Hex:EtOAc = 7:3 (2 % TEA)). The product is pale yellow oil. R_f = 0.18 (eluent: *n*-Hex:EtOAc = 7:3 (2% TEA)). Yield: 495 mg (36%).

^{1}H NMR (400 MHz, MeOH-d_4) δ 8.67 (d, *J* = 2.3 Hz, 1H, 2-H_{Ar}), 8.38 (dd, *J* = 4.8, 1.5 Hz, 1H, 6-H_{Ar}), 7.93 (ddd, *J* = 8.1, 2.3, 1.5 Hz, 1H, 4-H_{Ar}), 7.38 (dd, *J* = 8.1, 4.8 Hz, 1H, 5-H_{Ar}), 1.54 (s, 6H, -C*H*$_3$).^{13}C NMR (101 MHz, MeOH-d_4) δ 147.9 (6-C_{Ar}), 147.1 (2-C_{Ar}), 145.6 (3-C_{Ar}), 134.9 (4-C_{Ar}), 124.8 (5-C_{Ar}), 31.7 (2 x *C*H_3). HRMS (ESI, m/z) m/z calculated for $C_8H_{11}NO$ [M+H$^+$] 138.0913, found 138.0914. The data match those reported in the literature.[10]

2-(3-Pyridyl)-2-propanol **25**

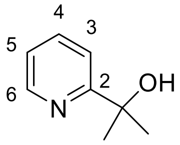

2-Acetylpyridine **23** (1.21 g, 10.0 mmol, 1.00 eq.) was dissolved in dry Et_2O (25 mL) and 3.0 M ethereal MeMgBr solution (3.60 mL, 11.0 mmol, 1.10 eq.) was added to the solution dropwise at 0 °C over 20 min. The reaction was left to warm to rt and stirred at this temperature for 1 h. Deionised water (25 mL) was added, followed by concentrated HCl solution (1.5 mL). The layers were separated and the organic layer was extracted with EtOAc (3 x 20 mL). The unified organic phase was dried over anhydrous $MgSO_4$, filtered and evaporated *in vacuo*. The yielded transparent oil (1.06 g) was dissolved in *n*-Hex (3 mL) and the solution was cooled to –20 °C. White crystals formed, which were filtered on sintered glass funnel and washed with copious amounts of cold *n*-Hex. This yielded white crystalline material which was used without further purification. Yield: 721 mg (53%).

^{1}H NMR (400 MHz, MeOH-d_4) δ 8.44 (d, *J* = 4.7 Hz, 1H, 6-H_{Ar}), 7.77 (t, *J* = 7.7 Hz, 1H, 4-H_{Ar}), 7.67 (d, *J* = 7.7 Hz, 1H, 3-H_{Ar}), 7.22 (dd, *J* = 7.7, 4.7 Hz, 1H, 5-H_{Ar}), 1.53 (s, 3H, -C*H*$_3$), 1.51 (s, 3H, -C*H*$_3$).^{13}C NMR (101 MHz, MeOH-d_4) δ 168.6 (2-C_{Ar}), 148.9 (6-C_{Ar}), 138.5 (4-C_{Ar}), 123.1 (5-C_{Ar}), 120.2 (3-C_{Ar}), 30.6 (-*C*H_3). HRMS (ESI, m/z) m/z calculated for $C_8H_{11}NO$ [M+Na$^+$] 160.0733, found 160.0733. The data match those reported in the literature.[11]

*(+/-)-1-(2-Pyridyl)-propan-1-ol rac-***27**

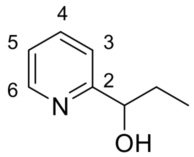

2-Bromopyridine (1.00 g, 6.33 mmol, 1.00 eq) was dissolved in dry Et_2O (30 mL) under Ar atmosphere and the solution was cooled to –78 °C. n-BuLi solution (1.6 M in hexanes, 3.96 mL, 6.33 mmol, 1.00 eq) was added dropwise to the solution at this temperature, followed by 45 min of stirring. Propionaldehyde (0.46 mL, 0.37 g, 6.33 mmol, 1.00 eq) was added in dry Et_2O (4.36 mL) to the reaction media dropwise and the mixture was stirred at rt for 15 min. The reaction media was quenched with deionised water (20 mL), the phases were separated and the aqueous phase was extracted with EtOAc (3 x 15 mL). The unified organic phase was dried over anhydrous $MgSO_4$, filtered and evaporated *in vacuo*. The crude material was purified by column chromatography (eluent: EtOAc/n-Hex = 7:3). The compound was yielded as dense yellow oil. R_f = 0.44 (eluent: EtOAc/n-Hex = 7:3). Yield: 547 mg (62%).

^1H NMR (400 MHz, $CDCl_3$) δ 8.53 (dd, *J* = 4.9, 1.5 Hz, 1H, 6-H_{Ar}), 7.67 (td, *J* = 7.5, 1.5 Hz, 2H, 4-H_{Ar}), 7.25 (d, *J* = 7.5 Hz, 1H, 3-H_{Ar}), 7.19 (dd, *J* = 7.5, 4.9 Hz, 1H, 5-H_{Ar}), 4.69 (dd, *J* = 7.2, 4.8 Hz, 1H, -C*H*-OH), 4.25 (s, 1H, -O*H*), 1.94 - 1.82 (m, 1H, -*H*CH-CH_3), 1.71 (dp, *J* = 14.5, 7.2 Hz, 1H, -HC*H*-CH_3), 0.94 (t, *J* = 7.2 Hz, 3H, -C*H*$_3$). ^{13}C NMR (101 MHz, $CDCl_3$) δ 162.1 (2-C_{Ar}), 148.3 (6-C_{Ar}), 136.7 (4-C_{Ar}), 122.4 (3-C_{Ar}), 120.5 (5-C_{Ar}), 73.9 (-*C*H-OH), 31.4 (-*C*H_2-), 9.5 (-*C*H_3). HRMS (ESI, m/z) m/z calculated for $C_8H_{11}NO$ [$M+Na^+$] 160.0738, found 160.0730 The data match those reported in the literature.[12]

5,6,7,8-Tetrahydroisoquinoline-5-ol **37a**, *5,6,7,8-Tetrahydroisoquinoline-8-ol* **37c**

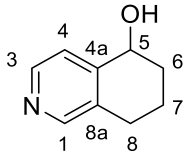

37a

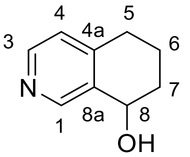

37c

5,6,7,8-Tetrahydroisoquinoline **3** (500 mg, 481 µL, 3.75 mmol, 1.00 eq.) was dissolved in the mixture of AcOH (0.29 mL) and H_2O (16.8 mL), followed by the addition of $KMnO_4$ (1.34 g, 8.48 mmol, 2.26 eq.) over 10 min at rt (ice bath was used to keep the temperature at rt). The slurry was left stirring for 1 h, then the reaction mixture was filtered on a sintered glass funnel. The filtrate was extracted with DCM (3x10 mL) and the combined organic phase was dried over $MgSO_4$, filtered, and evaporated in *vacuo*. The regioisomeric ketones (5- and 8-oxo products) could not be separated by column chromatography, thus the mixture of ketones was reduced to a mixture of alcohols according to the following protocol.

The mixture of the two ketones (194 mg) was dissolved in MeOH (15 mL) and $NaBH_4$ (78.0 mg, 2.06 mmol, 1.50 eq.) was added at rt in three portions. After 30 min, the reaction mixture was poured onto saturated $NaHCO_3$ solution (25 mL) and the aqueous phase was extracted with EtOAc (3x15 mL). The unified organic phase was dried over anhydrous $MgSO_4$, filtered, and evaporated under *vacuo*. Column chromatography (eluent: Et_2O/acetone = 4:1 (1% TEA)) afforded **37a** and **37c**. Compound **37a** is yellow oil. R_f = 0.50 (Et_2O/acetone = 2:1).

Yield: 17 mg (3.0%). Compound **37c** is deep brown oil. R_f = 0.37 (eluent: Et_2O:acetone = 2:1). Yield: 52 mg (9.0%). Mixed column fractions were not collected.

Data for compound **37a**: ^1H NMR (400 MHz, $CDCl_3$) δ 8.32 (d, *J* = 4.9 Hz, 1H, 3-H_{Ar}), 8.27 (s, 1H, 1-H_{Ar}), 7.38 (d, *J* = 4.9 Hz, 1H, 4-H_{Ar}), 4.71 (t, *J* = 5.9 Hz, 1H, 5-H), 2.64 - 2.80 (m, 2H, 7-H_α, 7-H_β), 2.12 - 1.88 (m, 2H, 8-H_α, 9-H_α), 1.64 - 1.80 (m, 2H, 8-H_β, 9-H_β), 1.41 (s, 1H, -O*H*). ^{13}C NMR (101 MHz, $CDCl_3$) δ 150.1 (1-C_{Ar}), 148.1 (4a-C_{Ar}), 147.1 (3-C_{Ar}), 132.5 (8a-C_{Ar}), 122.5 (4-C_{Ar}), 67.4 (5-C), 32.2 (6-C), 26.1 (8-C), 19.3 (7-C). HRMS (ESI, m/z) m/z calculated for C9H11NO [M+H$^+$] 150.0919, found 150.0912. The data match those reported in the literature.[13]

Data for compound **37c**: ^1H NMR (400 MHz, $CDCl_3$) δ 8.63 (s, 1H, 1-H_{Ar}), 8.36 (d, *J* = 5.1 Hz, 1H, 3-H_{Ar}), 7.01 (d, *J* = 5.1 Hz, 1H, 4-H_{Ar}), 4.87 (d, *J* = 4.9 Hz, 1H, 8-H), 2.98 - 2.57 (m, 2H, 5-H_α, 5-H_β), 2.09 - 1.73 (m, 4H, 6-H_α, 6-H_β, 7-H_α, 7-H_β). ^{13}C NMR (101 MHz, $CDCl_3$) δ 150.6 (1-C_{Ar}), 148.1 (3-C_{Ar}), 146.5 (5a-C_{Ar}), 135.0 (8a-C_{Ar}), 123.8 (4-C_{Ar}), 65.8 (8-C_{Ar}), 31.1 (7-C_{Ar}), 28.6 (5-C_{Ar}), 18.1 (6-C_{Ar}). HRMS (ESI, m/z) m/z calculated for C9H11NO [M+H$^+$] 150.0919, found 150.0912. The data match those reported in the literature.[14]

2-Methylquinoline-N-oxide **S3**

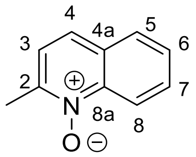

The material was synthesized according to the literature protocol.[15] Quinaldine **35** (470 µL, 500 mg, 3.50 mmol, 1.00 eq.) was dissolved in DCM (23.0 mL) and *m*CPBA was added in 3 portions over 15 min to the solution at 0 °C. The reaction was stirred at rt for 72 h. After this, saturated $NaHCO_3$ solution (23.0 mL) was added to the reaction mixture and the phases were separated. The aqueous phase was extracted with DCM (3 x 20 mL) and the unified organic phase was washed with brine (1 x 15 mL). The organic phase was dried over anhydrous $MgSO_4$, filtered and evaporated *in vacuo* to yield the crude material as brown oil. The crude was used in the next step without further purification. R_f = 0.45 (eluent: DCM:MeOH = 20:1). Yield: 482 mg (95%).

^1H NMR (400 MHz, $CDCl_3$) δ 8.75 (d, *J* = 8.7 Hz, 1H, 4-H_{Ar}), 7.80 (dd, *J* = 8.2, 1.2 Hz, 1H, 5-H_{Ar}), 7.72 (ddd, *J* = 8.5, 7.6, 1.2 Hz, 1H, 7-H_{Ar}), 7.62 (d, *J* = 8.5 Hz, 1H, 8-H_{Ar}), 7.56 (dd, *J* = 8.2, 7.6 Hz, 1H, 6-H_{Ar}), 7.28 (d, *J* = 8.7 Hz, 1H, 3-H_{Ar}), 2.69 (s, 3H, -C*H*$_3$). ^{13}C NMR (101 MHz, $CDCl_3$) δ 145.9 (2-C_{Ar}), 141.6 (8a-C_{Ar}), 130.3 (7-C_{Ar}), 129.3 (4a-C_{Ar}), 128.0 (5-C_{Ar}), 127.8 (6-C_{Ar}), 125.3 (8-C_{Ar}), 123.1 (3-C_{Ar}), 119.6 (4-C_{Ar}), 18.9 (-*C*H_3). HRMS (ESI, m/z) m/z calculated for $C_{10}H_9NO$ [M+H$^+$] 160.757, found 160.0755. The data match those published in the literature.[15]

Quinoline-2-carbaldehyde **S4**

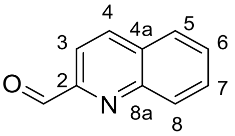

2-Methylquinoline-*N*-oxide **S3** (641 mg, 4.08 mmol, 1.00 eq.) was dissolved in dry DCM (5.0 mL) and Tf_2O (1.71 mL, 2.88 g, 10.2 mmol, 2.50 eq.) was added to the solution dropwise at rt. The temperature of the reaction media increased to reflux temperature, as a result of exothermic reaction as the reagent was added. The reaction was stirred at rt for 1 h and concentrated K_2CO_3 solution (10.0 mL) was added to the reaction. The reaction was stirred at rt overnight. Instead of the expected carbinol, quinoline-2-carbaldehyde **S4** was formed in the reaction. The crude was purified by column chromatography (eluent: *n*-Hex:EtOAc = 9:1). R_f = 0.41 (eluent: *n*-Hex:EtOAc = 9:1) to yield **S4** as yellow solid. Yield: 158 mg (25%).

^{1}H NMR (400 MHz, $CDCl_3$) δ 10.23 (s, 1H, -C*H*O), 8.31 (d, *J* = 8.4 Hz, 1H, 4-H_{Ar}), 8.25 (d, *J* = 8.4 Hz, 1H, 5-H_{Ar}), 8.03 (d, *J* = 8.4 Hz, 1H, 3-H_{Ar}), 7.90 (dd, *J* = 8.2, 1.3 Hz, 1H, 8-H_{Ar}), 7.83 (dd, *J* = 8.4, 1.3 Hz, 1H, 6-H_{Ar}), 7.72 - 7.65 (m, 1H, 7-H_{Ar}). ^{13}C NMR (101 MHz, $CDCl_3$) δ 193.9 (-*C*HO), 152.7 (2-C_{Ar}), 148.1 (8a-C_{Ar}), 137.6 (4-C_{Ar}), 130.7 (5-C_{Ar}), 130.6 (6-C_{Ar}), 130.2 (4a-C_{Ar}), 129.4 (7-C_{Ar}), 128.01 (8-C_{Ar}), 117.2 (3-C_{Ar}). HRMS (ESI, m/z) m/z calculated for $C_{10}H_7NO$ [$M+H^+$] 158.0600, found 158.601. The data match those published in the literature.[16]

2-Hydroxymethylquinoline **45**

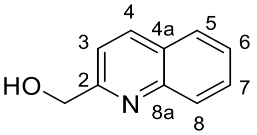

Quinoline-2-carbaldehyde **S4** (135 mg, 0.956 mmol, 1.00 eq.) was dissolved in MeOH (5.0 mL) and $NaBH_4$ (43.0 mg, 1.15 mmol, 1.20 eq.) was added in one portion at rt. The reaction was stirred at this temperature for 1 day. The reaction was poured onto saturated $NaHCO_3$ solution (15.0 mL) and the resulting suspension was extracted with EtOAc (3 x 10 mL). The unified organic phase was dried over anhydrous $MgSO_4$, filtered and evaporated *in vacuo* to yield the crude material as pale red oil. The crude material was used without further purification. R_f = 0.07 (eluent: *n*-Hex:EtOAc = 8:2). Yield: 126 mg (92%).

^{1}H NMR (400 MHz, $CDCl_3$) δ 8.17 (d, *J* = 8.4 Hz, 1H, 4-H_{Ar}), 8.11 (d, *J* = 8.4 Hz, 1H, 5-H_{Ar}), 7.84 (d, *J* = 7.8 Hz, 1H, 8-H_{Ar}), 7.73 (t, *J* = 8.5 Hz, 1H, 6-H_{Ar}), 7.56 (dd, *J* = 8.7, 7.8 Hz, 1H, 7-H_{Ar}), 7.31 (d, *J* = 8.4 Hz, 1H, 3-H_{Ar}), 4.94 (s, 2H, -C*H*$_2$O). ^{13}C NMR (101 MHz, $CDCl_3$) δ 159.1 (8a-C_{Ar}), 146.5 (2-C_{Ar}), 137.4 (4-C_{Ar}), 130.2 (5-C_{Ar}), 128.5 (8-C_{Ar}), 127.9 (7-C_{Ar}), 127.7 (4a-C_{ar}), 126.7 (6-C_{Ar}), 118.6 (3-C_{Ar}) 64.1 (-*C*H_2OH). The data match those published in the literature.[15]

2-Methoxy-5-bromopyridine **S5**

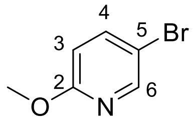

2,5-Dibromopyiridine (1.00 g, 4.22 mmol, 1.00 eq.) was dissolved in dry, degassed DMF (13.0 mL) under a N_2 atmosphere. In a separate flask, sodium metal (310 mg, 13.6 mmol, 3.23 eq.) was added carefully to MeOH portionwise (3.46 mL, 2.74 g, 85.5 mmol, 20.3 eq.). Once all of the sodium metal had reacted, the NaOMe solution was transferred to the reaction mixture via a canula. The resulting mixture was heated at 80 °C for 1 h and then cooled to rt. The reaction was diluted with Et_2O (33 mL) and it was washed with deionised H_2O (4 x 10 mL).

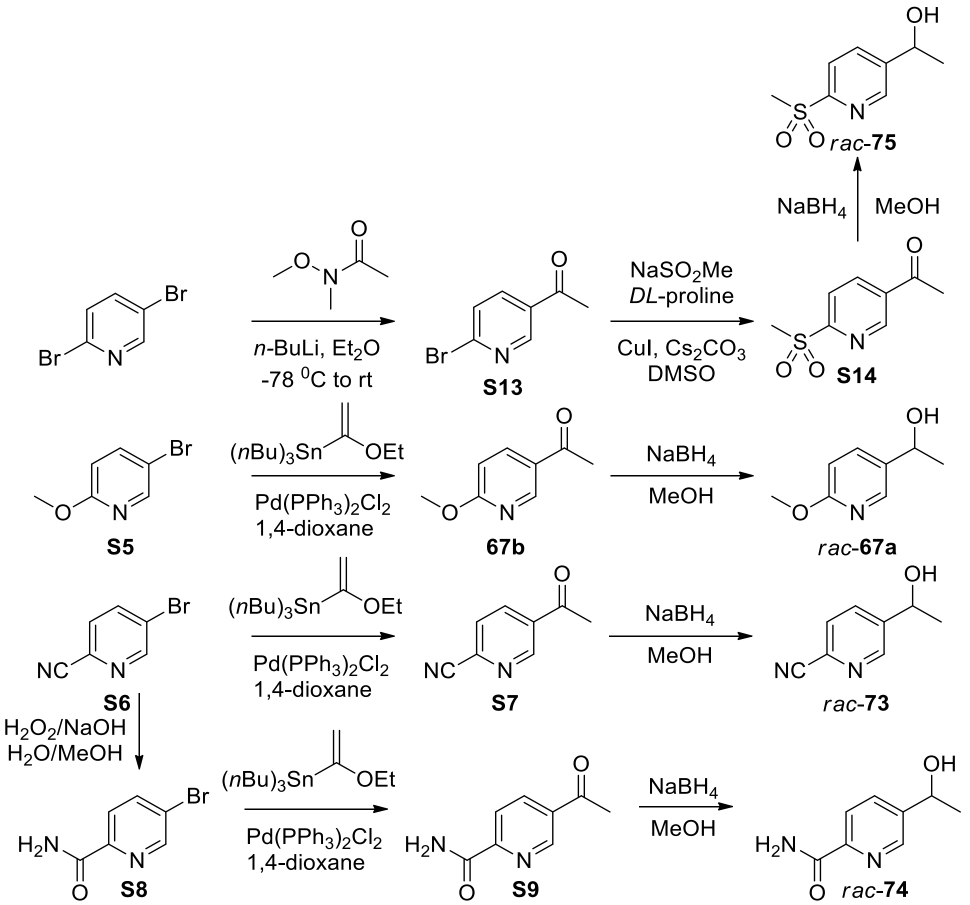

Scheme S2. Syntheses of *para*-substituted 5-ethylpyridines.

The unified aqueous phase was basified (pH = 10), the precipitate was filtered, and the filtrate was extracted with Et_2O (2 x 20 mL). The organic phases were combined, dried over anhydrous $MgSO_4$, filtered and evaporated under reduced pressure. The yielded crude material (yellow oil) was purified by column chromatography (eluent: *n*-Hex:EtOAc = 20:1) and **S5** was yielded as pale yellow oil. R_f = 0.42 (eluent: *n*-Hex:EtOAc = 20:1). Yield: 371 mg (47%).

^{1}H NMR (400 MHz, $CDCl_3$) δ 8.19 (d, *J* = 2.5 Hz, 1H, 6-H_{Ar}), 7.63 (dd, *J* = 8.6, 2.5 Hz, 1H, 4-H_{Ar}), 6.66 (d, *J* = 8.6 Hz, 1H, 3-H_{Ar}), 3.90 (s, 3H, OC*H*$_3$). ^{13}C NMR (101 MHz, $CDCl_3$) δ 163.1 (2-C_{Ar}), 147.6 (6-C_{Ar}), 141.2 (4-C_{Ar}), 112.7 (3-C_{Ar}), 111.8 (5-C_{Ar}), 53.9 (O*C*H_3). HRMS (APCI,

m/z) m/z calculated for $C_6H_6{}^{79}BrNO$ [M+H$^+$] calculated 187.9706, found 187.9709. The data match those published in the literature.[17]

2-Methoxy-5-ethylpyridine **66**

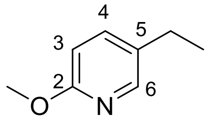

2-Methoxy-5-bromopyridine (400 mg, 2.13 mmol, 1.00 eq.), anhydrous K_2CO_3 (1.18 g, 8.52 mmol, 4.00 eq.), and Pd(dppf)Cl_2·DCM (87.4 mg, 0.107 mmol, 5.00 mol%) were suspended in dry, degassed DMF (5.0 mL). Next, triethylborane solution (1.0 M in THF, 2.82 mL, 2.82 mmol, 1.30 eq.) was added to the solution dropwise at rt over 20 min. The

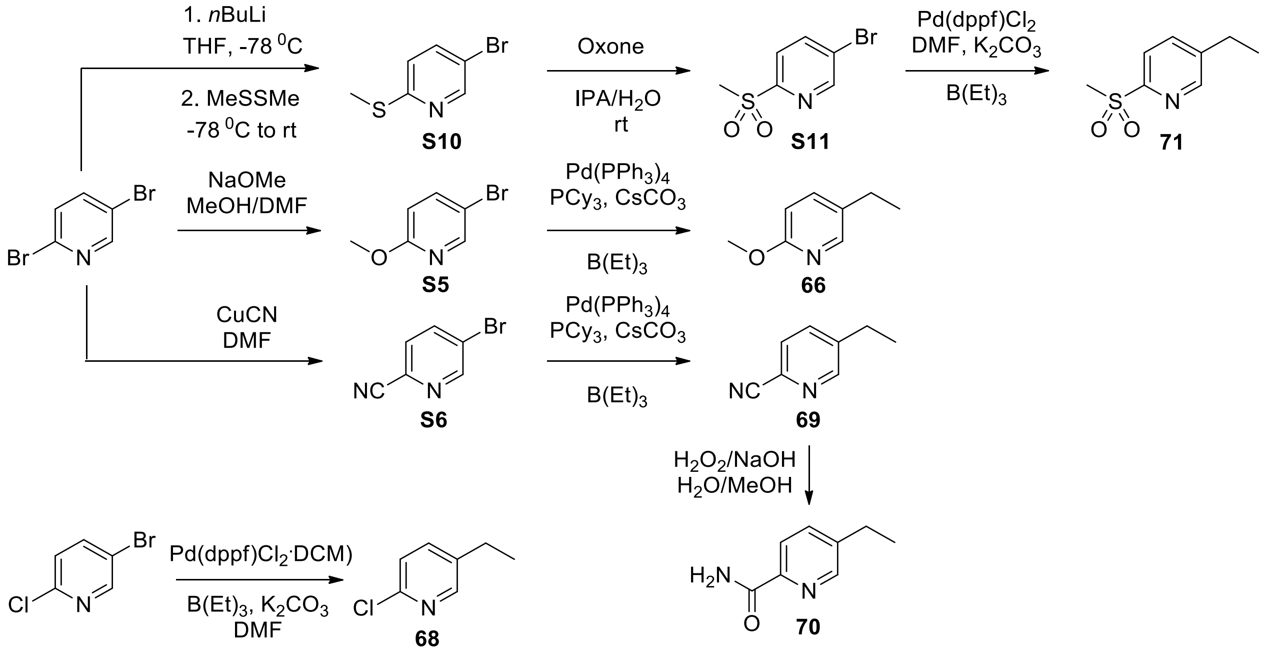

Scheme S3. Syntheses of *para*-substituted (pyridin-3-yl)-ethanol authentic standards.

colour of the reaction turned brown as a result. The reaction was heated at 85 °C for 4 h and it was left to cool to rt. The reaction mixture was diluted with deionised H_2O (20 mL) and Et_2O (20 mL). The layers were separated and the aqueous phase was extracted with Et_2O (2 x 20 mL). The combined organic phase was washed with brine (3 x 10 mL), dried over anhydrous $MgSO_4$, filtered and evaporated *in vacuo* to yield the crude material as brown oil. The crude (370 mg) was purified by column chromatography (eluent: *n*-Hex:EtOAc = 9:1) to yield the title compound **66** as yellow oil. R_f = 0.31 (eluent: *n*-Hex:EtOAc = 9:1). Yield: 278 mg (95%).

^{1}H NMR (400 MHz, $CDCl_3$) δ 7.98 (d, *J* = 2.5 Hz, 1H, 6-H$_{Ar}$), 7.41 (dd, *J* = 8.3, 2.5 Hz, 1H, 4-H$_{Ar}$), 6.68 (d, *J* = 8.3 Hz, 1H, 3-H$_{Ar}$), 3.91 (s, 3H, OC*H*$_3$), 2.56 (q, *J* = 7.6 Hz, 2H, C*H*$_2$), 1.21 (t, *J* = 7.6 Hz, 2H, -CH$_2$-C*H*$_3$). ^{13}C NMR (101 MHz, $CDCl_3$) δ 162.7 (2-C$_{Ar}$), 145.6 (6-C$_{Ar}$), 138.7 (4-C$_{Ar}$), 132.1 (5-C$_{Ar}$), 110.5 (3-C$_{Ar}$), 53.4 (O*C*H$_3$), 25.3 (*C*H$_2$), 15.8 (CH$_2$*C*H$_3$). HRMS (ESI, m/z) m/z calculated for $C_8H_{12}NO$ [M+H$^+$] 138.0913, found 138.0915. The data match those published in the literature.[18]

1-(6-Methoxypyridin-3-yl)-ethan-1-one **67b**

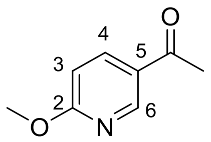

The compound was synthesized according to the literature.[19] 2-Methoxy-5-bromopyridine **S5** (100 mg, 0.550 mmol, 1.00 eq.) and $Pd(PPh_3)_2Cl_2$ (39.0 mg, 0.0550 mmol, 0.100 eq.) were dissolved in dry, degassed 1,4-dioxane (10.0 mL). Tributyl-(1-ethoxyvinyl)-stannane (223 µL, 238 mg, 0.660 mmol, 1.20 eq.) was added to the solution dropwise and the reaction was refluxed overnight. 1.0 M aqueous HCl solution (10.0 mL) was added to the reaction and the mixture was stirred at rt for 3 h. The layers were separated and the aqueous layer was neutralised with saturated $NaHCO_3$ solution. The aqueous phase was extracted with EtOAc (3 x 30 mL). The unified organic phase was washed with brine (20 mL), dried over anhydrous $MgSO_4$, filtered and evaporated *in vacuo*. The yielded crude material (green oil) was purified by column chromatography (eluent: *n*-Hex:EtOAc = 20:1). The product was obtained as a yellow oil. R_f = 0.26 (eluent: *n*-Hex:EtOAc = 20:1). Yield: 72 mg (87%).

^{1}H NMR (400 MHz, $CDCl_3$) δ 8.78 (d, *J* = 1.8 Hz, 1H, 6-H_{Ar}), 8.14 (dd, *J* = 8.7, 1.8 Hz, 1H, 4-H_{Ar}), 6.79 (d, *J* = 8.7 Hz, 1H, 3-H_{Ar}), 4.01 (s, 3H, OC*H*$_3$), 2.57 (s, 3H, CO-C*H*$_3$). ^{13}C NMR (101 MHz, $CDCl_3$) δ 195.9 (*C*O), 167.0 (2-C_{Ar}), 149.6 (6-C_{Ar}), 138.3 (4-C_{Ar}), 119.3 (5-C_{Ar}), 111.3 (3-C_{Ar}), 54.2 (O*C*H$_3$), 26.5 (CO-*C*H$_3$). HRMS (ESI, m/z) m/z calculated for $C_8H_9NO_2$ [M+H$^+$] 152.0712, found 152.0707. The data match those published in the literature.[19]

*(+/-)-1-(6-Methoxypyridin-3-yl)-ethanol rac-***67a**

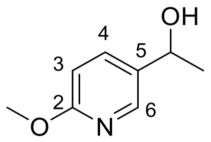

1-(6-Methoxypyridin-3-yl)-ethan-1-one **67b** (50.0 mg, 0.331 mmol, 1.00 eq.) was dissolved in MeOH (5.0 mL) and $NaBH_4$ (15.0 mg, 0.397 mmol, 1.20 eq.) was added in one portion at 0 °C. The reaction was stirred at rt for 3 h. The reaction media was poured onto saturated $NaHCO_3$ solution (20 mL) and the aqueous phase was extracted with EtOAc (3 x 30 mL). The unified organic phase was washed with brine (20 mL), dried over anhydrous $MgSO_4$, filtered, and evaporated under reduced pressure to yield the crude (50 mg) as cloudy oil. The crude was purified by column chromatography (eluent: *n*-Hex:EtOAc = 7:3) to yield the product *rac*-**67a** as transparent oil. R_f = 0.19 (eluent: *n*-Hex:EtOAc = 7:3). Yield: 28 mg (55%).

^{1}H NMR (400 MHz, $CDCl_3$) δ 8.07 (d, *J* = 2.5 Hz, 1H, 6-H_{Ar}), 7.62 (dd, *J* = 8.6, 2.5 Hz, 1H, 4-H_{Ar}), 6.72 (d, *J* = 8.6 Hz, 1H, 3-H_{Ar}), 4.86 (q, *J* = 6.5 Hz, 1H, -C*H*(OH)-), 3.90 (s, 3H, OC*H*$_3$), 1.47 (d, *J* = 6.4 Hz, 3H, CH(OH)-C*H*$_3$). ^{13}C NMR (101 MHz, $CDCl_3$) δ 163.9 (2-C_{Ar}), 144.3 (6-C_{Ar}), 136.5 (4-C_{Ar}), 133.9 (5-C_{Ar}), 111.0 (3-C_{Ar}), 67.9 (-*C*H(OH)-), 53.6 (O*C*H$_3$), 25.0 (-CH(OH)-*C*H$_3$). HRMS (ESI, m/z) m/z calculated for $C_8H_{11}NO_2$ [M+H$^+$] 154.0863, found 154.0858. The data match those published in the literature.[20]

2-Chloro-5-ethylpyridine **68**

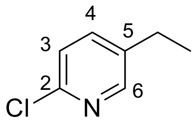

2-Chloro-5-bromopyridine (500 mg, 2.60 mmol, 1.00 eq.), anhydrous K_2CO_3 (1.44 g, 10.4 mmol, 4.00 eq.), and Pd(dppf)Cl_2*DCM (106 mg, 0.130 mmol, 5.00 mol%) were dissolved in dry, degassed DMF (10.0 mL). Triethylborane solution (1.0 M in THF, 3.38 mL, 3.38 mmol, 1.30 eq.) was added to the reaction dropwise at rt. The reaction was heated at 50 °C for 72 h. The reaction was poured onto deionised H_2O (50 mL) and Et_2O was added (50 mL). The layers were separated, and the aqueous layer was extracted with Et_2O (3 x 50 mL). The combined organic phase was washed with 5% aqueous LiCl solution (3 x 30 mL) and brine (1 x 30 mL). The organic phase was dried over anhydrous $MgSO_4$, filtered, and evaporated under reduced pressure to yield the crude material as brown, dense oil. The crude (226 mg) was purified by column chromatography (eluent: *n*-Hex:EtOAc = 7:3). The purified material contained minor amounts (10%) of the undesired 2-ethyl-5-bromopyridine. The purified material is pale yellow oil. R_f = 0.20 (eluent: *n*-Hex:EtOAc = 20:1). Yield: 95 mg (59% calculated for 90% purity).

^{1}H NMR (400 MHz, $CDCl_3$) δ 8.22 (d, *J* = 2.8 Hz, 1H, 6-C_{Ar}), 7.47 (dd, *J* = 8.0, 2.6 Hz, 1H, 4-C_{Ar}), 7.23 (d, *J* = 8.0 Hz, 1H, 3-C_{Ar}), 2.63 (q, *J* = 7.7 Hz, 2H, -C*H*$_2$-), 1.24 (t, *J* = 7.7 Hz, 3H, -C*H*$_3$). ^{13}C NMR (101 MHz, $CDCl_3$) δ 161.7 (2-C_{Ar}), 149.3 (6-C_{Ar}), 138.4 (4-C_{Ar}), 124.0 (3-C_{Ar}), 118.1 (5-C_{Ar}), 25.5 (-*C*H_2-), 15.3 (-*C*H_3). HRMS (ESI, m/z) m/z calculated for C_7H_9ClN [M+H$^+$] 142.0418, found 142.0413. IR (film): ν_{max}/cm^{-1} 2969 (C-H_{Ar}), 2934 (C-H_{Ar}), 1462 (C-N), 1108, 735.

2-Cyano-5-bromopyridine **S6**

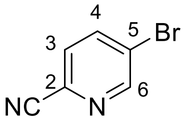

The compound was synthesized according to the literature.[21] 2,5-Dibromopyiridine (2.00 g, 8.44 mmol, 1.00 eq.), CuCN (0.65 g, 7.23 mmol, 0.85 eq.), and KCN (0.47 g, 7.23 mmol, 0.85 eq.) were suspended in dry, degassed DMF (20.0 mL) under N_2 atmosphere and the mixture was heated at 150 °C for 12 h. After this, the reaction media was diluted with Et_2O (80.0 mL) and the organic phase was washed with distilled water (4 x 15 mL). The aqueous phase was basified with 1 M NaOH solution (pH = 10) and extracted with EtOAc (3 x 20 mL). The unified organic phase was washed with brine (2 x 15 mL), dried over anhydrous $MgSO_4$, filtered and evaporated *in vacuo*. The crude material was purified by column chromatography (eluent: *n*-Hex:EtOAc = 9:1) to yield **S6** as white solid. R_f = 0.39 (eluent: *n*-Hex:EtOAc = 9:1). Yield: 315 mg (22%).

^{1}H NMR (400 MHz, $CDCl_3$) δ 8.78 (dd, *J* = 2.3, 0.8 Hz, 1H, 6-H_{Ar}), 7.99 (dd, *J* = 8.3, 2.3 Hz, 1H, 4-H_{Ar}), 7.59 (dd, *J* = 8.3, 0.8 Hz, 1H, 3-H_{Ar}). ^{13}C NMR (101 MHz, $CDCl_3$) δ 152.8 (6-C), 139.9 (4-C), 132.2 (2-C), 129.4 (3-C), 125.3 (5-C), 116.7 (-*C*N). HRMS (APCI, m/z) m/z calculated for $C_7H_3{}^{79}BrN_2$ [M+H$^+$] 182.9552, found 182.9545. The data match those published in the literature.[22]

2-Cyano-5-ethylpyridine **69**

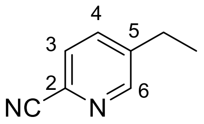

2-Cyano-5-bromopyridine **S6** (340 mg, 2.01 mmol, 1.00 eq.), anhydrous K_2CO_3 (1.11 g, 8.05 mmol, 4.00 eq.), and Pd(dppf)Cl_2·DCM (82.0 mg, 0.101 mmol, 5.00 mol%) were suspended in dry, degassed DMF (5.0 mL). The triethylborane solution (1.0 M in THF, 2.62 mL, 2.62 mmol, 1.30 eq.) was added to the solution dropwise at rt over 20 min. The colour of the reaction turned brown, as a result. The reaction was heated at 85 °C for 4 h and it was left to cool to rt. The reaction mixture was diluted with deionised H_2O (20 mL) and Et_2O (20 mL). The layers were separated and the aqueous phase was extracted with Et_2O (2 x 20 mL). The combined organic phase was washed with brine (3 x 10 mL), dried over anhydrous $MgSO_4$, filtered and evaporated *in vacuo* to yield the crude material as brown oil. The crude (326 mg) was purified by column chromatography (eluent: *n*-Hex:EtOAc = 7:3) to yield the title compound **69** as yellow oil. R_f = 0.41 (eluent: *n*-Hex:EtOAc = 7:3). Yield: 246 mg (93%).

^{1}H NMR (400 MHz, $CDCl_3$) δ ^{13}C NMR (101 MHz, $CDCl_3$) δ 8.56 (s, 1H, 6-H_{Ar}), 7.69 - 7.58 (m, 2H, 3-H_{Ar}, 4-H_{Ar}), 2.74 (q, *J* = 7.6 Hz, 2H, -C*H*$_2$-), 1.29 (t, *J* = 7.6 Hz, 3H, -C*H*$_3$). ^{13}C NMR (101 MHz, $CDCl_3$) δ 151.3 (6-C_{Ar}), 143.7 (5-C_{Ar}), 136.1 (4-C_{Ar}), 131.4 (2-C_{Ar}), 128.3 (3-C_{Ar}), 117.6 (-*C*N), 26.4 (-*C*H_2-), 14.9 (-*C*H_3). HRMS (ESI, m/z) m/z calculated for $C_8H_8N_2$ [M+Na$^+$] 155.0580, found 155.0578. The data match those published in the literature.[23]

5-Acetyl-2-pyridinecarbonitrile **S7**

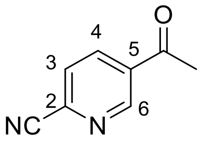

2-Cyano-5-bromopyridine **S6** (200 mg, 1.18 mmol, 1.00 eq.) and Pd(PPh_3)$_2$$Cl_2$ (83.0 mg, 0.118 mmol, 0.100 eq.) were dissolved in dry, degassed 1,4-dioxane. The tributyl-(1-ethoxyvinyl)-stannane (440 µL, 470 mg, 1.30 mmol, 1.10 eq.) was added to the solution dropwise and the reaction mixture was heated for 4 h at reflux temperature. After this, 1.0 M aqueous HCl solution (10.0 mL) was added to the reaction and the mixture was stirred at rt for 3 h. The reaction was neutralised with saturated $NaHCO_3$, the layers were separated and the aqueous phase was extracted with EtOAc (3 x 50 mL). The combined organic phase was washed with brine (25.0 mL), dried over anhydrous $MgSO_4$, filtered and evaporated *in vacuo* to yield the crude material as brown oil. The crude was purified by column chromatography (eluent: *n*-Hex:EtOAc = 7:3). R_f = 0.14 (eluent: *n*-Hex:EtOAc = 7:3). Yield: 169 mg (98%).

^{1}H NMR (400 MHz, $CDCl_3$) δ 9.22 (d, *J* = 2.1 Hz, 1H, 6-H_{Ar}), 8.36 (dd, *J* = 8.1, 2.1 Hz, 1H, 4-H_{Ar}), 7.83 (d, *J* = 8.1 Hz, 1H, 3-H_{Ar}), 2.69 (s, 3H, -CO-C*H*$_3$). ^{13}C NMR (101 MHz, $CDCl_3$) δ 195.3 (-*C*O-), 150.8 (6-C_{Ar}), 137.0 (2-C_{Ar}), 136.7 (4-C_{Ar}), 133.9 (5-C_{Ar}), 128.5 (3-C_{Ar}), 116.6 (-*C*N), 27.1 (-CO-*C*H_3). HRMS (ESI, m/z) m/z calculated for $C_8H_6N_2O$ [M+H$^+$] 147.0553, found 147.0557. The data match those published in the literature.[24]

*(+/-)-5-(1-Hydroxyethyl)-pyridine-2-carbonitrile rac-***73**

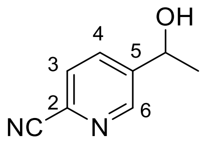

5-Acetyl-2-pyridinecarbonitrile **S7** (50.0 mg, 0.342 mmol, 1.00 eq.) was dissolved in MeOH (5.0 mL) and $NaBH_4$ (16.0 mg, 0.411 mmol, 1.20 eq.) was added in one portion at rt. The reaction was stirred at this temperature overnight and the reaction mixture was poured onto saturated $NaHCO_3$ solution (20 mL). The aqueous phase was extracted with EtOAc (3 x 20 mL), dried over anhydrous $MgSO_4$, filtered, and evaporated under reduced pressure to yield the crude material as transparent oil. The crude was used without further purification. R_f = 0.19 (eluent: *n*-Hex:EtOAc = 7:3). Yield: 49 mg (96%).

^{1}H NMR (400 MHz, $CDCl_3$) δ 8.66 (d, *J* = 2.2 Hz, 1H, 6-H_{Ar}), 7.88 (dd, *J* = 8.0, 2.2 Hz, 1H, 4-H_{Ar}), 7.66 (d, *J* = 8.0 Hz, 1H, 3-H_{Ar}), 5.03 (q, *J* = 6.5 Hz, 1H, -C*H*(OH)-), 1.52 (d, *J* = 6.5 Hz, 3H, -C*H*$_3$). ^{13}C NMR (101 MHz, $CDCl_3$) δ 149.0 (6-C_{Ar}), 145.5 (2-C_{Ar}), 134.2 (4-C_{Ar}), 132.3 (5-C_{Ar}), 128.5 (3-C_{Ar}), 117.3 (-*C*N), 67.5 (-*C*(OH)-), 25.4 (-*C*H_3). HRMS (ESI, m/z) m/z calculated for $C_8H_8N_2O$ [M+H$^+$] 149.0709, found 149.0701. The data match those published in the literature.[25]

p-Bromobenzamide **S8**

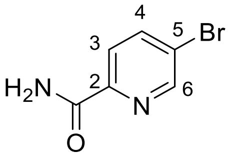

2-Cyano-5-bromopyridine **S6** (100 mg, 0.592 mmol, 1.00 eq) was dissolved in MeOH (0.50 mL) and 3.0 M NaOH solution was added (0.60 mL) and white precipitate was formed, as a result. 30% H_2O_2 (156 µL, 5.09 mmol, 11.6 eq.) was added to the suspension dropwise and reaction was stirred at rt overnight. The precipitate was filtered on sintered glass funnel and the cake was washed with copious amounts of water and cold Et_2O (10.0 mL). The crude material (white microcrystalline solid) was used without further purification. R_f = 0.59 (eluent: *n*-Hex:EtOAc = 7:3). Yield: 107 mg (90%).

^{1}H NMR (400 MHz, $(CD_3)_2SO$) δ 8.74 (d, *J* = 2.4 Hz, 1H, 6-H_{Ar}), 8.22 (dd, *J* = 8.4, 2.4 Hz, 1H, 4-H_{Ar}), 8.13 (s, 1H, -HN*H*), 7.95 (d, *J* = 8.4 Hz, 1H, 3-H_{Ar}), 7.72 (s, 1H, -*H*NH). ^{13}C NMR (101 MHz, $(CD_3)_2SO$) δ 165.3 (*C*O-NH_2), 149.2 (6-C_{Ar}), 149.1 (2-C_{Ar}), 140.4 (4-C_{Ar}), 123.9 (3-C_{Ar}), 123.4 (5-C_{Ar}).

5-Ethylpicolinamide **70**

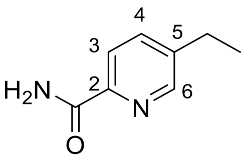

2-Cyano-5-ethylpyridine **69** (100 mg, 0.757 mmol, 1.00 eq.) was dissolved in MeOH (0.50 mL) and 3.0 M aqueous NaOH solution was added to the solution (0.60 mL). White precipitate

formed, as a result. To the suspension was added 30% H_2O_2 solution (200 µL, 74.0 mg, 2.18 mmol, 2.88 eq.) dropwise at rt and the mixture was stirred at rt for 30 min. The precipitate was filtered and the cake was washed with copious amounts of deionised H_2O. The crude material was dried under vacuum and was used without further purification. R_f = 0.16 (eluent:*n*-Hex:EtOAc = 7:3). Yield: 70 mg (62%).

^{1}H NMR (400 MHz, $CDCl_3$) δ 8.40 (d, *J* = 2.2 Hz, 1H, 6-H_{Ar}), 8.12 (d, *J* = 7.9 Hz, 1H, 3-H_{Ar}), 7.82 (broad s, 1H, -N*H*$_2$), 7.67 (dd, *J* = 7.9, 2.3 Hz, 1H, 4-H_{Ar}), 5.82 (s, 1H, -N*H'*$_2$), 2.72 (q, *J* = 7.7 Hz, 2H, -C*H*$_2$-CH_3), 1.28 (t, *J* = 7.6 Hz, 2H). ^{13}C NMR (101 MHz, $CDCl_3$) δ 167.2 (-*C*O-), 148.3 (6-C_{Ar}), 147.4 (2-C_{Ar}), 142.9 (5-C_{Ar}), 136.6 (4-C_{Ar}), 122.4 (3-C_{Ar}), 26.2 (-*C*H_2-CH_3), 15.2 (-CH_2-*C*H_3). HRMS (ESI, m/z) m/z calculated for $C_8H_{10}N_2O$ [M+H$^+$] 151.0871, found 151.0870.

5-Acetylpicolinamide **S9**

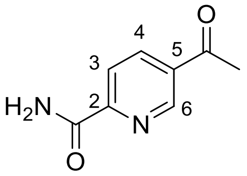

p-Bromobenzamide **S8** (200 mg, 1.18 mmol, 1.00 eq.) and $Pd(PPh_3)_2Cl_2$ (82.8 mg, 0.118 mmol, 0.10 eq.) were dissolved in dry, degassed 1,4-dioxane (15.0 mL) and tributyl(1-ethoxyvinyl)-stannane (478 µL, 511 mg, 1.42 mmol, 1.20 eq.) was added to the solution dropwise at rt. The reaction was then refluxed for 4 h. After this, 1.0 M aqueous HCl solution (10.0 mL) was added to the reaction and the mixture was stirred at rt for 3 h. The mixture was neutralised with saturated $NaHCO_3$ solution, the layers were separated, and the aqueous phase was extracted with EtOAc (3 x 50 mL). The unified organic phase was dried over anhydrous $MgSO_4$, filtered and evaporated *in vacuo.* The crude material (brown oil) was purified by column chromatography (eluent: *n*-Hex:EtOAc = 7:3). The title compound **S9** was yielded as yellow oil. R_f = 0.39 (eluent: *n*-Hex:EtOAc = 7:3). Yield: 168 mg (97%).

^{1}H NMR (400 MHz, $CDCl_3$) δ 9.22 (d, *J* = 2.1 Hz, 1H, 6-H_{Ar}), 8.36 (dd, *J* = 8.0, 2.1 Hz, 1H, 4-H_{Ar}), 7.83 (d, *J* = 8.0 Hz, 1H, 3-H_{Ar}), 2.69 (s, 3H, -C*H*$_3$). ^{13}C NMR (101 MHz, $CDCl_3$) δ 195.3 (-*C*O-CH_3), 150.8 (6-C_{Ar}), 137.0 (2-C_{Ar}), 136.6 (4-C_{Ar}), 133.9 (5-C_{Ar}), 128.5 (3-C_{Ar}), 116.6 (-*C*N), 27.1 (-*C*H_3). HRMS (ESI, m/z) m/z calculated for $C_8H_6N_2O$ [M+H$^+$] 147.0557, found 147.0553. The data match those published in the literature.[24]

(+/-)-5-(1-Hydroxyethyl)pyridine-2-carboxamide rac-**74**

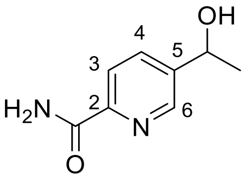

5-Acetyl-2-pyridinecarbonitrile **S7** (20.0 mg, 0.122 mmol, 1.00 eq.) was dissolved in MeOH (5.0 mL) and $NaBH_4$ (5.40 mg, 0.146 mmol, 1.20 eq.) was added to the solution in one portion at 0 °C. The reaction was stirred at rt overnight. The reaction was poured onto saturated $NaHCO_3$ solution (20 mL) and extracted with EtOAc (3 x 20 mL), dried over anhydrous $MgSO_4$, filtered and evaporated under reduced pressure to yield the crude material as white microcrystalline solid. The crude material was used without further purification. R_f = 0.31 (eluent: *n*-Hex:EtOAc = 7:3). Yield: 19.3 mg (87%).

^1H NMR (400 MHz, MeOH-d_4) δ 8.63 (s, 1H, 6-H_{Ar}), 8.06 (d, J = 8.2 Hz, 1H, 3-H_{Ar}), 7.93 (dd, J = 8.2, 2.2 Hz, 1H, 4-H_{Ar}), 4.95 (q, J = 6.8 Hz, 1H, -CH(OH)-), 1.47 (d, J = 6.8 Hz, 3H, -CH_3). ^{13}C NMR (101 MHz, MeOH-d_4) δ 169.4 (-CO-NH_2), 149.7 (2-C_{Ar}), 147.8 (6-C_{Ar}), 146.6 (5-C_{Ar}), 135.7 (4-C_{Ar}), 123.1 (3-C_{Ar}), 68.3 (-CH(OH)-), 25.3 (-CH_3). HRMS (ESI, m/z) m/z calculated for $C_8H_{10}N_2O_2$ [M+Na^+] 189.0634, found 189.0632.

5-Bromo-2-(methylsulfanyl)-pyridine **S10**

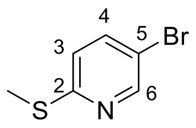

The compound was synthesized according a literature method.[26] 2,5-Dibromopyiridine (2.00 g, 8.44 mmol, 1.00 eq.) was dissolved in dry toluene (100 mL) under N_2 atmosphere. *n*-BuLi solution (2.50 M in hexanes, 4.05 mL, 10.1 mmol, 1.20 eq.) was added to the reaction dropwise at –78 °C. The reaction was stirred for 2 h at this temperature and dimethyldisulfide (0.97 mL, 1.03 g, 11.0 mmol, 1.30 eq.) was added to the mixture dropwise over 20 min. The reaction was left to warm to room temperature and stirred overnight. The reaction was poured onto 1.0 M aqueous NaOH solution (50 mL), the layers were separated, and the aqueous layer was extracted with EtOAc (3 x 30 mL). The unified organic phase was dried over anhydrous $MgSO_4$, filtered and evaporated under reduced pressure to yield the crude material as a dense, yellow oil. The compound was used without further purification. R_f = 0.74 (eluent: *n*-Hex:EtOAc = 10:1). Yield: 1.50 g (87%).

^1H NMR (400 MHz, $CDCl_3$) δ 8.48 (d, J = 2.4 Hz, 1H, 6-H_{Ar}), 7.58 (dd, J = 8.6, 2.4 Hz 1H, 4-H_{Ar}), 7.08 (d, J = 8.6 Hz, 1H, 3-H_{Ar}), 2.54 (s, 3H, -S-CH_3). ^{13}C NMR (101 MHz, $CDCl_3$) δ 158.9 (2-C_{Ar}), 150.4 (6-C_{Ar}), 138.4 (4-C_{Ar}), 122.8 (3-C_{Ar}), 115.9 (5-C_{Ar}), 13.6 (-S-$C$$H_3$). HRMS (APCI, m/z) m/z calculated for $C_6H_6{}^{79}BrNS$ 203.9477, found 203.9478. The data match those published in the literature.[27]

5-Bromo-2-methylsulfonylpyridine **S11**

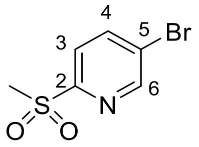

5-Bromo-2-(methylsulfanyl)-pyridine **S10** (190 mg, 0.931 mmol, 1.00 eq) was dissolved in a mixture of *i*-PrOH (12.7 mL) and deionised H_2O (6.3 mL). This was followed by the addition of oxone (326 mg, 2.14 mmol, 2.30 eq.) in a single portion at rt. The reaction was stirred at 60 °C for 48 h. After this time, further oxone (300 mg, 1.97 mmol, 2.12 eq.) was added the reaction was stirred at 60 °C overnight. The volatiles were removed under reduced pressure and the residual material was diluted with EtOAc (40 mL), the layers were separated and the organic phase was washed with deionised H_2O (2 x 15 mL), and brine (15 mL). The organic layer was dried over $MgSO_4$, filtered, and evaporated under reduced pressure to yield the crude material as a pale brown solid. The material was used without further purification. R_f = 0.32 (eluent: *n*-Hex:EtOAc = 7:3). Yield: 207 mg (95%).

^1H NMR (400 MHz, $CDCl_3$) δ 8.78 (d, J = 2.3 Hz, 1H, 6-H_{Ar}), 8.11 (dd, J = 8.3, 2.3 Hz, 1H, 4-H_{Ar}), 7.98 (d, J = 8.3 Hz, 1H, 3-H_{Ar}), 3.23 (s, 3H, -SO_2CH_3). ^{13}C NMR (101 MHz, $CDCl_3$) δ

156.6 (2-C_{Ar}), 151.4 (6-C_{Ar}), 141.0 (4-C_{Ar}), 125.5 (5-C_{Ar}), 122.6 (3-C_{Ar}), 40.2 (-SO_2CH_3). HRMS (ESI, m/z) m/z calculated for $C_6H_6{}^{79}BrNO_2S$ [M+H$^+$] 235.9375, found 235.9373. The data match those published in the literature.[28]

5-Ethyl-2-methylsulfonylpyridine **71**

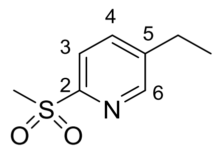

5-Bromo-2-methylsulfonylpyiridine **S11** (150 mg, 0.675 mmol, 1.00 eq.), Pd(dppf)Cl_2*DCM (22.6 mg, 0.0338 mmol, 5 mol%) and K_2CO_3 (370 mg, 2.70 mmol, 4.00 eq.) were suspended in degassed and dry DMF (3.0 mL). Triethylborane solution (1.0 M in THF, 0.88 mL, 0.88 mmol, 1.3 eq.) was added to the reaction dropwise at rt over 10 min. The reaction turned brown, as a result. The reaction was stirred at 85 °C for 24 h. After this, the reaction was diluted with deionised H_2O (10 mL) and Et_2O (10 mL). The layers were separated and the aqueous phase was extracted with Et_2O (3 x 20 mL Et_2O). The combined organic phase was washed with 5% LiCl solution (3 x 15 mL) and brine (1 x 10 mL). The organic phase was dried over anhydrous $MgSO_4$, filtered, and evaporated *in vacuo* to yield the crude material as brown oil. The crude material was purified by column chromatography (eluent: *n*-Hex:EtOAc = 7:3) to yield the product as a yellow oil. R_f = 0.29 (eluent:*n*-Hex:EtOAc = 7:3). Yield: 116 mg (93%).

^{1}H NMR (400 MHz, $CDCl_3$) δ 8.57 (d, *J* = 2.0 Hz, 1H, 6-H_{Ar}), 8.01 (d, *J* = 7.9 Hz, 1H, 3-H_{Ar}), 7.77 (dd, *J* = 7.9, 2.0 Hz, 1H, 4-H_{Ar}), 3.22 (s, 3H, -SO_2-CH_3), 2.78 (q, *J* = 7.6 Hz, 2H, -CH_2-), 1.31 (t, *J* = 7.6 Hz, 3H, -CH_3). ^{13}C NMR (101 MHz, $CDCl_3$) δ 155.7 (2-C_{Ar}), 150.1 (2-C_{Ar}), 144.2 (5-C_{Ar}), 137.3 (4-C_{Ar}), 121.2 (3-C_{Ar}), 40.4 (-SO_2CH_3), 26.3 (-CH_2-), 15.1 (-CH_3). HRMS (ESI, m/z) m/z calculated for $C_8H_8NO_2S$ [M+H$^+$] 186.0583, found 186.0582. IR (neat film): v_{max}/cm^{-1} 2929 (CH_{Ar}), 1303 (SO), 1158, 1102.

N-Methoxy-N-methylacetamide **S12**

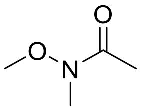

N-Methoxy-*N*-methylamine hydrochloride (15.0 g, 154 mmol, 1.00 eq.) was suspended in dry DCM (300 mL) under N_2 atmosphere in a two-neck round bottom flask equipped with a thermometer. TEA (42.9 mL, 31.1 g, 308 mmol, 2.00 eq.) was added dropwise over 10 min at 0 °C. After this, acetyl chloride (10.9 mL, 12.1 g, 154 mmol, 1.00 eq.) was added to the solution dropwise. Special care was taken to keep the temperature below 4 °C during this step. The reaction was stirred at rt for 16 h and quenched by the addition of saturated $NaHCO_3$ solution (225 mL). The layers were separated and the organic phase was washed with 1.0 M aqueous HCl solution (50 mL) and brine (50 mL). The organic phase was dried over anhydrous $MgSO_4$, filtered and evaporated under reduced pressure to yield the crude material (10.2 g) as yellow oil. The crude material was purified by vacuum distillation (77–80 °C at 53 mbar) to yield the pure title compound as colourless oil. Yield: 7.96 g (60%).

^{1}H NMR (400 MHz, $CDCl_3$) δ 3.67 (s, 3H, OCH_3), 3.16 (s, 3H, NCH_3), 2.11 (s, 3H, $COCH_3$). 13C NMR (101 MHz, $CDCl_3$) δ 172.2 (*C*O), 61.2 (OCH_3), 32.1 ($COCH_3$), 19.9 (NCH_3). The data match those published in the literature.[29]

1-(6-Bromopyridin-3-yl)-ethan-1-one **S13**

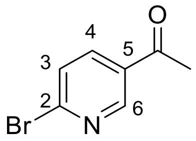

2,5-Dibromopyridine (5.00 g, 21.1 mmol 1.00 eq.) was dissolved in dry Et_2O (80 mL) in flame-dried 250 mL round bottom flask under dry N_2 atmosphere. *n*-BuLi solution (2.50 M, 9.29 mL, 23.2 mmol, 1.10 eq.) was added dropwise to the solution over 20 min at –78 °C. The reaction was stirred at this temperature for 30 min and the solution of *N*-methoxy-*N*-methylacetamide **S12** (2.70 mL, 2.61 g, 25.3 mmol, 1.20 mmol) in dry Et_2O (5.0 mL) was added dropwise to the solution over 20 min at this temperature. The reaction was left to warm to rt and stirred at this temperature for 1 h. The reaction was poured onto saturated NH_4Cl solution (150 mL) and the phases were separated. The aqueous layer was further extracted with EtOAc (3 x 100 mL). The crude material (3.56 g) was yielded as yellow oil. The crude material was recrystallised from EtOAc (15 mL) to yield pure **S13** as pale, yellow needles. R_f = 0.39 (eluent: *n*-Hex:EtOAc = 8:2). Yield: 3.28 g (78%).

^{1}H NMR (400 MHz, $CDCl_3$) δ 8.89 (d, *J* = 2.5 Hz, 1H, 6-H_{Ar}), 8.07 (dd, *J* = 8.3, 2.5 Hz, 1H, 4-H_{Ar}), 7.61 (d, *J* = 8.3 Hz, 1H, 3-H_{Ar}), 2.62 (s, 3H, -CO-C*H*$_3$). ^{13}C NMR (101 MHz, $CDCl_3$) δ 195.8 (-*C*O-), 150.6 (6-C_{Ar}), 147.1 (2-C_{Ar}), 137.8 (4-C_{Ar}), 131.6 (5-C_{Ar}), 128.6 (3-C_{Ar}), 26.9 (-*C*H$_3$). HRMS (ESI, m/z) m/z calculated for $C_7H_8{}^{79}BrNO$ [M+H$^+$] 199.9706, found 199.9701. The data match those published in the literature.[30]

1-(6-Methylsulfonylpyridin-3-yl)-ethanone **S14**

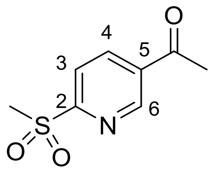

1-(6-Bromopyridin-3-yl)-ethan-1-one **S13** (200 mg, 1.00 mmol, 1.00 eq.), sodium methylsulfinate (240 mg (85% purity), 2.00 mmol, 2.00 eq.), DL-proline (35 mg, 0.30 mmol, 0.30 eq.), CuI (57 mg, 0.30 mmol, 0.30 eq.), and Cs_2CO_3 (98 mg, 0.30 mmol, 0.30 eq.) were measured into a flame-dried 25 mL round bottom flask under N_2 atmosphere and dry DMSO (4.0 mL) was added. The mixture was heated at 140 °C for 4 h and poured into deionised H_2O (40 mL). The precipitate was filtered on a sintered glass funnel and the cake was washed with EtOAc and deionised water. The phases of the filtrate were separated and the aqueous phase was extracted with EtOAc (3 x 30 mL). The unified organic phase was washed with brine (20 mL), dried over anhydrous $MgSO_4$, filtered, and evaporated *in vacuo* to yield the crude material as brown oil. The crude was used without further purification. R_f = 0.28 (eluent: *n*-Hex:EtOAc = 1:1). Yield: 84 mg (42%).

^{1}H NMR (400 MHz, $CDCl_3$) δ 9.22 (d, *J* = 2.2 Hz, 1H, 6-H_{Ar}), 8.48 (dd, *J* = 8.2, 2.4 Hz, 1H, 4-H_{Ar}), 8.20 (d, *J* = 8.2 Hz, 1H, 3-H_{Ar}), 3.28 (s, 3H, -SO_2-C*H*$_3$), 2.70 (s, 3H, -CO-C*H*$_3$). ^{13}C NMR (101 MHz, $CDCl_3$) δ 195.3 (-*C*O-), 161.1 (2-C_{Ar}), 150.1 (6-C_{Ar}), 138.2 (4-C_{Ar}), 134.8 (5-C_{Ar}), 121.1 (3-C_{Ar}), 40.0 (-SO_2-*C*H$_3$), 27.2 (-CO-*C*H$_3$). HRMS (ESI, m/z) m/z calculated for $C_8H_9NO_3S$ [M+H$^+$] 200.0376, found 200.375. IR (film) ν_{max}/cm^{-1} 3009 (-CH_{Ar}), 2927 (-CH_{Ar}), 1692 (-C=O), 1596 (C-N), 1303 (S=O), 1162 (S=O), 1103, 958, 761.

*(+/-)-1-(6-Methylsulfonylpyridin-3-yl)-ethanol rac-***75**

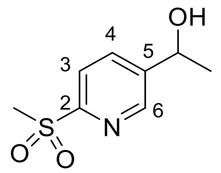

1-(6-Methylsulfonylpyridin-3-yl)-ethan-1-one **S14** (68.0 mg, 0.341 mmol, 1.00 eq.) was dissolved in MeOH (3.0 mL) and $NaBH_4$ (12.9 mg, 0.341 mmol, 1.00 eq.) was added in one portion at 0 °C. The reaction was stirred at this temperature for 1 h and the solution was poured onto saturated $NaHCO_3$ solution (10 mL). The solution was then extracted with EtOAc (3 x 10 mL) and the unified organic phase was dried over anhydrous $MgSO_4$, filtered and evaporated under reduced pressure. The crude material (yellow oil, 45 mg) was purified by column chromatography (eluent: EtOAc:*n*-Hex = 7:3) to yield pure title compound *rac*-**75** as pale yellow oil. R_f = 0.31 (eluent: EtOAc:*n*-Hex = 7:3). Yield: 31 mg (45%).

^{1}H NMR (400 MHz, $CDCl_3$) δ 8.66 (s, 1H, 6-H_{Ar}), 7.99 - 7.94 (m, 2H, 3-H_{Ar}, 4-H_{Ar}), 5.04 (q, *J* = 6.7 Hz, 1H, -C*H*(OH)-), 3.18 (s, 3H, -SO_2-C*H*$_3$), 1.52 (d, *J* = 6.7 Hz, 3H, -CH(OH)-C*H*$_3$). ^{13}C NMR (101 MHz, $CDCl_3$) δ 156.5 (2-C_{Ar}), 148.1 (6-C_{Ar}), 146.0 (4-C_{Ar}), 135.3 (5-C_{Ar}), 121.1 (3-C_{Ar}), 67.5 (-*C*H(OH)-), 40.4 (-SO_2-*C*H_3), 25.5 (-CH(OH)-C*H*$_3$). HRMS (ESI, m/z) m/z calculated for $C_8H_{11}NO_3S$ [M+H$^+$] 202.0532, found 202.0538.

2-Ethylpyridine-N-oxide **57**

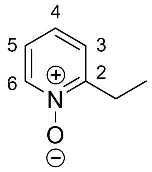

Ethylpridine (62.0 mg, 0.580 mmol, 1.00 eq.) was dissolved in $CHCl_3$ (30 mL) and *m*CPBA (143 mg (~70 % peroxybenzoic acid content), 0.580 mmol, 1.00 eq.) were added to the solution at 0℃. The reaction was stirred at rt for 24 h and the reaction mixture was washed with 0.1 M NaOH (80 mL) solution, followed by water (2x50 mL). The aqueous phase was extracted with EtOAc (3x30 mL), but it still contained *N*-oxide according to TLC. The unified organic phase was dried over $MgSO_4$, filtered, and the crude was purified by column chromatography ($CHCl_3$/MeOH = 19:1). The product is a yellow oil. R_f = 0.42 (eluent $CHCl_3$/MeOH = 19:1). Yield: 37 mg (52%).

^{1}H NMR (400 MHz, $CDCl_3$) δ 8.26 (d, *J* = 6.4 Hz, 1H, 6-H_{Ar}), 7.25 – 7.20 (m, 2H, 3-H_{Ar}, 5-H_{Ar}), 7.17–7.11 (m, 1H, 4-H_{Ar}), 2.96 (q, *J* = 7.5 Hz, 2H, -C*H*$_2$-CH_3), 1.32 (t, *J* = 7.5 Hz, 3H, -CH_2-C*H*$_3$). ^{13}C NMR (101 MHz, $CDCl_3$) δ 154.0 (2-C_{Ar}), 139.7 (6-C_{Ar}), 125.9 (C_{Ar}), 124.6 (C_{Ar}), 123.4 (C_{Ar}), 23.8 (-*C*H_2-CH_3), 10.7 (-CH_2-*C*H_3). HRMS (ESI, m/z) m/z calculated for C_7H_9NO [M+H$^+$] 124.0762, found 124.0756. The data match those reported in the literature.[31]

2-Propylpyridine-1-oxide **59**

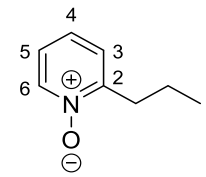

2-Propylpyridine (15.0 mg, 0.120 mmol, 1.00 eq) was dissolved in DCM (2 mL) and *m*-CPBA (23.0 mg, 0.130 mmol, 1.10 eq) was added in three portions at rt, every 30 min. The suspension was stirred overnight at rt. The reaction medium was quenched by the addition of aqueous $Na_2S_2O_5$ (5.00 mL, 114 mg, 0.600 mmol, 5.00 eq) solution to the suspension. After 2 h of stirring, the phases were separated and the aqueous phase was extracted with EtOAc (3x10 mL). The combined organic phase was dried over $MgSO_4$, filtered and evaporated *in vacuo*. The crude material was purified by column chromatography (eluent: EtOAc/MeOH = 7:3) to yield the product as a yellow solid. $R_f = 0.47$ (eluent: EtOAc/MeOH = 7:3). Yield: 13.9 mg (94%). HRMS (ESI, m/z) m/z calculated for $C_8H_{11}NO$ $[M+H^+]$ 138.0919, found .138.0911.

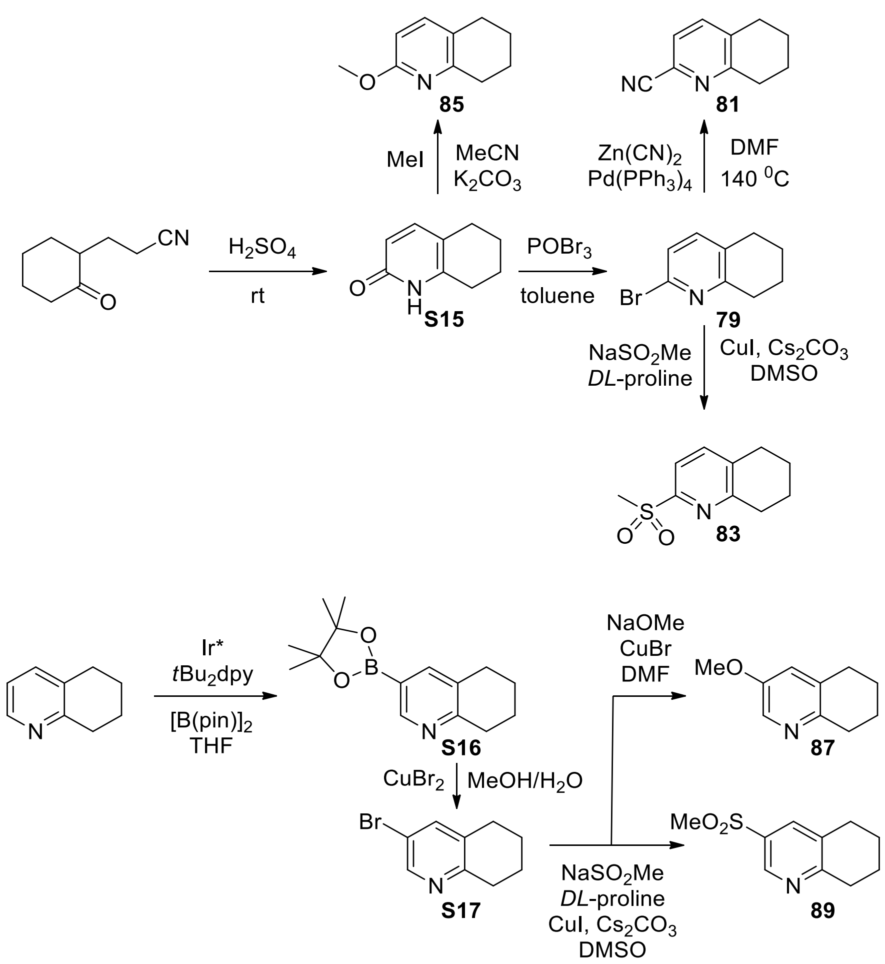

Scheme S4. Syntheses of 2- and 3-substituted 5,6,7,8-tetrahydroquinolines.

^{1}H NMR (400 MHz, MeOH-d$_4$) δ 8.28 (d, *J* = 6.4 Hz, 1H, 6-H_{Ar}), 7.51 (d, *J* = 7.5 Hz, 1H, 3-H_{Ar}), 7.48 (td, *J* = 7.3, 2.3 Hz, 1H, 5-H_{Ar}), 7.36 (td, *J* =7.4 Hz, 2.5 Hz, 1H, 4-H_{Ar}), 2.93 – 2.80 (t, *J* = 7.5 Hz ,2H, -C*H*$_2$-CH$_2$-), 1.73 (sext, *J* = 7.5 Hz, 2H, -CH$_2$-C*H*$_2$-), 0.98 (t, *J* = 7.4 Hz, 3H, -

CH_3). ^{13}C NMR (101 MHz, MeOH-d_4) δ 154.1 (2-C_{Ar}), 132.3 (6-C_{Ar}), 130.8 (5-C_{Ar}), 127.9 (4-C_{Ar}), 125.6 (3-C_{Ar}), 33.4 (-CH_2-CH_2-), 20.8 (-CH_2-CH_2-), 14.1 (-CH_3). The data match those reported in the literature.[32]

5,6,7,8-Tetrahydro-1H-quinolin-2-one **S15**

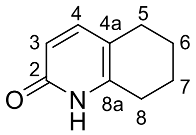

The compound was prepared according to literature protocol.[33] 2-Cyanoethylcyclohexanone (1.47 mL, 1.50 g, 10.0 mmol, 1.00 eq.) was added to concentrated H_2SO_4 (10.0 mL) dropwise at 0 °C. The yielded brown solution was stirred at rt for 18 h. After this, the solution was poured onto 50 g crushed ice it was neutralised with saturated ammonia solution. The mixture was extracted with DCM (3 x 20 mL). The combined organic phase was dried over anhydrous $MgSO_4$, filtered, and evaporated under reduced pressure. The yielded yellow solid was used without further purification. R_f = 0.21 (eluent: *n*-Hex:EtOAc = 7:3 (1% AcOH)). Yield: 1.19 g (80%).

^1H NMR (400 MHz, $CDCl_3$) δ 13.1 (s, 1H, -O*H*), 7.18 (d, *J* = 9.1 Hz, 1H, 4-H_{Ar}), 6.36 (d, *J* = 9.1 Hz, 1H, 3-H_{Ar}), 2.68 (t, *J* = 6.1 Hz, 2H, 5-H_α, 5-H_β), 2.47 (t, *J* = 5.8 Hz, 2H, 8-H_α, 8-H_β), 1.82 – 1.67 (m, 4H, 6-H_α, 6-H_β, 7-H_α, 7-H_β). ^{13}C NMR (101 MHz, $CDCl_3$) δ 165.1 (-*C*O-), 143.9 (3-C_{Ar}), 143.3 (8a-C_{Ar}), 116.9 (4-C_{Ar}), 114.7 (4a-C_{Ar}), 26.8 (5-C), 26.2 (8-C), 22.6 (6-C), 21.7 (7-C). HRMS (ESI, m/z) m/z calculated for $C_9H_{11}NO$ [M+H$^+$] 150.0913, found 150.0914. The data match those published in the literature.[33]

2-Bromo-5,6,7,8-tetrahydroquinoline **79**

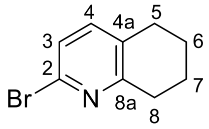

5,6,7,8-Tetrahydro-1*H*-quinolin-2-one **S15** (1.20 g, 8.04 mmol, 1.00 eq.) was dissolved in dry toluene (30.0 mL) in a dry flask equipped with a reflux condenser under N_2 atmosphere and a solution of $POBr_3$ (4.61 g, 16.1 mmol, 2.00 eq.) was added to the reaction in dry toluene (15.0 mL). The solution was refluxed for 2 h. The reaction mixture was cooled to 0 °C and the reaction media was basified by the dropwise addition of 2.0 M aqueous NaOH solution (30 mL) at this temperature. The layers were separated, the organic layer was dried over anhydrous $MgSO_4$, filtered and evaporated *in vacuo* to yield the crude as amorphous brown material. The crude material was purified by column chromatography (eluent: *n*-Hex:EtOAc = 20:1) to yield **79** as pale yellow oil. R_f = 0.21 (eluent: *n*-Hex:EtOAc = 20:1). Yield: 855 mg (50%).

^1H NMR (400 MHz, $CDCl_3$) δ 7.22–7.17 (m, 2H, 3-H_{Ar}, 4-H_{Ar}), 2.89 (t, *J* = 6.5 Hz, 2H, 5-H_α, 5-H_β), 2.70 (t, *J* = 6.4 Hz, 2H, 8-H_α, 8-H_β), 1.89–1.83 (m, 1H), 1.82–1.70 (m, 3H). ^{13}C NMR (101 MHz, $CDCl_3$) δ 159.1 (8a-C_{Ar}), 139.6 (4-C_{Ar}), 138.3 (2-C_{Ar}), 131.7 (4a-C_{Ar}), 125.2 (3-C_{Ar}), 32.3 (8-C), 28.3 (5-C), 22.8 (7-C), 22.4 (6-C). HRMS (ESI, m/z) m/z calculated for $C_9H_{10}N^{79}Br$ [M+H$^+$] 212.0075, found 212.0069. The data match those published in the literature.[34]

5,6,7,8-Tetrahydroquinoline-2-carbonitrile **81**

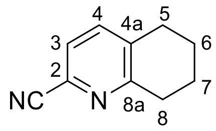

2-Bromo-5,6,7,8-tetrahydroquinoline **79** (200 mg, 0.943 mmol, 1.00 eq.), $Zn(CN)_2$ (220 mg, 1.89 mmol, 2.00 eq.) and $Pd(PPh_3)_4$ (55.0 mg, 0.0470 mmol, 5 mol%) were added to a flame-dried flask under N_2 atmosphere and dry, before degassed DMF (5.0 mL) was added. The mixture was stirred at rt for 15 min and then it was heated at 140 °C for 12 h. The reaction was poured onto ice (15 mL) and the mixture was extracted with EtOAc (3 x 30 mL). The unified organic phase was washed with basic brine solution (pH = 13.0, 3 x 30 mL), the organic phase was dried over anhydrous $MgSO_4$, filtered, and evaporated *in vacuo* to yield the crude material as brown oil. The crude (195 mg) was purified by column chromatography (eluent: *n*-Hex:EtOAc = 9:1) to yield pure **81** as white solid. R_f = 0.23 (eluent: *n*-Hex:EtOAc = 7:3). Yield: 52 mg (35%).

^{1}H NMR (400 MHz, MeOH-d_4) δ 7.65 (d, *J* = 8.2 Hz, 1H, 4-H_{Ar}), 7.56 (d, *J* = 8.2 Hz, 1H, 3-H_{Ar}), 2.90 (t, *J* = 6.9 Hz, 2H, 2 x 8-H), 2.87 (t, *J* = 6.9 Hz, 2 x 5-H), 1.95 - 1.89 (m, 2H, 7-$H_α$, 7-$H_β$), 1.87 - 1.81 (m, 2H, 6-$H_α$, 6-$H_β$). ^{13}C NMR (101 MHz, MeOH-d_4) δ 161.1 (8a-C_{Ar}), 139.4 (4-C_{Ar}), 139.2 (2-C_{Ar}), 131.1 (4a-C_{Ar}), 127.2 (3-C_{Ar}), 118.5 (-*C*N), 33.1 (8-C), 29.8 (5-C), 23.5 (7-C), 23.1 (6-C). HRMS (ESI, m/z) m/z calculated for $C_{10}H_{10}N_2$ [M+H$^+$] 159.0917, found 159.0920. The data match those published in the literature.[35]

2-(Methylsulfonyl)-5,6,7,8-tetrahydroquinoline **83**

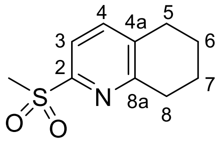

2-Bromo-5,6,7,8-tetrahydroquinoline **79** (200 mg, 0.943 mmol, 1.00 eq.), sodium methylsulfinate (190 mg, 1.89 mmol, 2.00 eq.), Cs_2CO_3 (91.3 mg, 0.280 mmol, 0.300 eq.), *DL*-proline (33.0 mg, 0.280 mmol, 0.300 eq.), CuI (53.3 mg, 0.280 mmol, 0.300 eq.) were measured into a flame-dried flask under an Ar atmosphere and dry, degassed DMSO was added (5.0 mL). After 5 min stirring at rt, the reaction was heated to 140 °C and was stirred at this temperature for 4 h. The reaction was diluted with deionised H_2O (30 mL) and EtOAc (30 mL) and the layers were separated. The aqueous phase was further extracted with EtOAc (3 x 40 mL), the combined organic phase was washed with basified brine (pH = 13.0, 3 x 20 mL), dried over anhydrous $MgSO_4$, filtered, and evaporated *in vacuo* to yield the crude as red oil. The crude material (176 mg) was purified by column chromatography (eluent: *n*-Hex:EtOAc = 7:3) to yield the product **83** as dark yellow oil. R_f = 0.25 (eluent: *n*-Hex:EtOAc = 7:3). Yield: 152 mg (82%).

^{1}H NMR (400 MHz, $CDCl_3$) δ 7.80 (d, *J* = 8.0 Hz, 1H, 4-H_{Ar}), 7.59 (d, *J* = 8.0 Hz, 1H, 3-H_{Ar}), 2.99 (t, *J* = 6.4 Hz, 2H, 5-$H_α$, 5-$H_β$), 2.86 (t, *J* = 6.3 Hz, 2H, 8-$H_α$, 8-$H_β$), 1.99 - 1.87 (m, 2H, 6-$H_α$, 6-$H_β$), 1.91 - 1.79 (m, 2H, 7-$H_α$, 7-$H_β$). ^{13}C NMR (101 MHz, $CDCl_3$) δ 159.2 (8a-C_{Ar}), 154.8 (2-C_{Ar}), 138.4 (4-C_{Ar}), 137.6 (4a-C_{Ar}), 118.3 (3-$_{Ar}$), 40.3 (-SO_2-*C*H_3), 32.6 (5-C), 29.1 (8-C), 22.7 (6-C), 22.3 (7-C). HRMS (ESI, m/z) m/z calculated for $C_{10}H_{13}NSO_2$ [M+H$^+$] 212.0740, found 212.0738.

2-Methoxy-5,6,7,8-Tetrahydroquinoline **85**

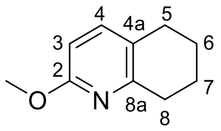

5,6,7,8-Tetrahydro-1*H*-quinolin-2-one **S15** (200 mg, 1.34 mmol, 1.00 eq.) and K_2CO_3 (1.12 g, 8.04 mmol, 6.00 eq.) were suspended in dry MeCN (5.0 mL) under N_2 atmosphere and MeI (0.25 mL, 0.57 g, 4.02 mmol, 3.0 eq.) was added. The reaction was heated at 40 °C for 36 h. The volatiles were removed under reduced pressure, the residue was dissolved in deionised H_2O (10.0 mL) and EtOAc (10.0 mL). The layers were separated, and the aqueous layer was further extracted with EtOAc (3 x 20 mL). The unified aqueous phase was washed with brine (15.0 mL), dried over anhydrous $MgSO_4$, filtered, and evaporated *in vacuo*. The crude (brown solid) was purified by column chromatography (eluent: *n*-Hex:EtOAc = 1:1) to yield **85** as a pale yellow solid. R_f = 0.53 (eluent: *n*-Hex:EtOAc = 1:1). Yield: 162 mg (75%).

^{1}H NMR (400 MHz, $CDCl_3$) δ 7.07 (d, *J* = 9.1 Hz, 1H, 4-H_{Ar}), 6.45 (d, *J* = 9.1 Hz, 1H, 3-H_{Ar}), 3.49 (s, 3H, -C*H*$_3$), 2.62 (t, *J* = 6.3 Hz, 2H, 8-H_α, 8-H_β), 2.50 (t, *J* = 6.2 Hz, 2H, 5-H_α, 5-H_β), 1.88 - 1.79 (m, 2H, 7-H_α, 7-H_β), 1.74 - 1.64 (m, 2H, 6-H_α, 6-H_β). ^{13}C NMR (101 MHz, $CDCl_3$) δ 163.4 (2-C_{Ar}), 143.5 (8a-C_{Ar}), 141.4 (4-C_{Ar}), 117.3 (3-C_{Ar}), 114.9 (4a-C_{Ar}), 30.4 (-*C*H_3), 27.5 (5/8-C), 27.5 (5/8-C) 22.6 (6-C), 21.8 (7-C). HRMS (ESI, m/z) m/z calculated for $C_{10}H_{13}NO$ [M+H$^+$] 164.1070, found 164.1069. The data match those published in the literature.[36]

3-(4,4,5,5-Tetramethyl-1,3,2-dioxaborolan-2-yl)-5,6,7,8-tetrahydroquinoline **S16**

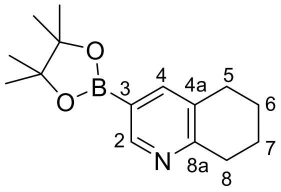

The compound was prepared according to literature protocol.[37] $[Ir(COD)(OMe)]_2$ (59.6 mg, 90.0 µmol, 3 mol%), $[B(pin)]_2$ (0.726 g, 3.00 mmol, 1.00 eq.), and 4,4'-bis(*tert*-butyl)-2-2'-dipyridyl (42.3 mg, 180 µmol, 6 mol%) were dissolved in dry THF under N_2 atmosphere and the solution was refluxed for 15 min. 5,6,7,8-Tetrahydroquinoline **3** (388 µL, 400 mg, 3.00 mmol, 1.00 eq.) was added to the solution at this temperature. The reaction mixture was refluxed overnight. The volatiles were removed and the crude material (dense brown oil) was used without further purification. R_f = 0.09 (eluent: EtOAc:MeOH = 9:1).

^{1}H NMR (400 MHz, $CDCl_3$) δ 8.67 (d, *J* = 1.5 Hz, 1H, 2-H_{Ar}), 7.74 (d, *J* = 1.5 Hz, 1H, 4-H_{Ar}), 2.93 (t, *J* = 6.3 Hz, 2H, 8-H_α, 8-H_β), 2.75 (t, *J* = 6.2 Hz, 2H, 5-H_α, 5-H_β), 1.92 - 1.85 (m, 2H, 6-H_α, 6-H_β), 1.82 - 1.77 (m, 2H, 7-H_α, 7-H_β), 1.34 (s, 12H, 4 x C*H*$_3$). ^{13}C NMR (101 MHz, $CDCl_3$) δ 160.3 (8a-C_{Ar}), 152.6 (2-C_{Ar}), 143.3 (4-C_{Ar}), 131.7 (4a-C_{Ar}), 84.1 (-O-*C*-*C*-O-), 32.8 (8-C), 28.7 (5-C), 25.0 (4 x *C*H_3), 23.1 (6-C), 22.83 (7-C). The data match those published in the literature.[37]

3-Bromo-5,6,7,8-tetrahydroquinoline **S17**

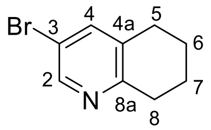

The crude boronic ester **S16** (200 mg) was dissolved in MeOH (6.0 mL) and $CuBr_2$ (1.17 g, 5.25 mmol) was added in deionised H_2O (6.0 mL). The mixture was heated at 75 °C for 6 h. Saturated aqueous NH_3 solution (6.0 mL) was added to the reaction mixture and the aqueous mixture was extracted with Et_2O (3 x 30 mL). The unified organic phase was washed with brine (10 mL) and dried over anhydrous $MgSO_4$, filtered and evaporated *in vacuo* to yield the crude as brown oil (461 mg). The crude was purified by column chromatography (eluent: *n*-Hex:EtOAc = 20:1) to yield **S17** as pale yellow oil. R_f = 0.12 (eluent: *n*-Hex:EtOAc = 20:1). Yield: 271 mg (85% calculated for two steps from 5,6,7,8-tetrahydroquinoline).

^{1}H NMR (400 MHz, $CDCl_3$) δ 8.39 (d, *J* = 2.3 Hz, 1H, 2-H_{Ar}), 7.49 (d, *J* = 2.3 Hz, 1H, 4-H_{Ar}), 2.86 (t, *J* = 6.4 Hz, 2H, 8-H_α, 8-H_β), 2.75 (t, *J* = 6.3 Hz, 2H, 5-H_α, 5-H_β), 1.93 - 1.85 (m, 2H, 7-H_α, 7-H_β), 1.83 – 1.75 (m, 2H, 6-H_α, 6-H_β). ^{13}C NMR (101 MHz, $CDCl_3$) δ 156.1 (8a-C_{Ar}), 147.8 (2-C_{Ar}), 139.1 (4-C_{Ar}), 134.3 (4a-C_{Ar}), 117.4 (3-C_{Ar}), 32.1 (8-C), 28.8 (5-C), 22.9 (7-C), 22.4 (6-C). HRMS (ESI, m/z) m/z calculated for $C_9H_{10}{}^{79}BrN$ [M+H$^+$] 212.0069, found 212.0069. The data match those published in the literature.[37]

3-Methoxy-5,6,7,8-tetrahydroquinoline **87**

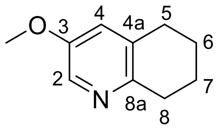

3-Bromo-5,6,7,8-tetrahydroquinoline **S17** (200 mg, 0.944 mmol, 1.00 eq.) and CuBr (271 mg, 1.89 mmol, 2.00 eq.) were dissolved in a dry, degassed DMF (14.0 mL) in a flame-dried flask. In a separate flame-dried flask, sodium metal (1.13 g, 49.1 mmol, 52.0 eq.) was reacted with dry MeOH (10.0 mL). Once completed, the fresh sodium methylate solution was added to the reaction mixture at rt. The reaction was then heated at 105 °C for 4 h. The reaction was cooled and poured onto 150 mL ice-cold water. The aqueous phase was extracted with EtOAc (4 x 50 mL). The unified organic phase was washed with brine (3 x 30 mL), dried over $MgSO_4$, filtered and evaporated *in vacuo*. The residual DMF was removed by azeotroping with *n*-heptane. The crude material (brown oil, 233 mg) was purified by column chromatography (eluent: *n*-Hex:EtOAc = 7:3) to yield pure **87** as yellow oil. R_f = 0.14 (eluent: *n*-Hex:EtOAc = 7:3). Yield: 126 mg (82%).

^{1}H NMR (400 MHz, $CDCl_3$) δ 8.07 (d, *J* = 2.8 Hz, 1H, 2-H_{Ar}), 6.90 (d, *J* = 2.9 Hz, 1H, 4-H_{Ar}), 3.81 (s, 3H, -OC*H*$_3$), 2.86 (t, *J* = 6.3 Hz, 2H, 5-H_α, 5-H_β), 2.75 (t, *J* = 6.3 Hz, 2H, 8-H_α, 8-H_β), 1.91 - 1.83 (m, 2H, 6-H_α, 6-H_β), 1.82 - 1.75 (m, 2H, 7-H_α, 7-H_β). ^{13}C NMR (101 MHz, $CDCl_3$) δ 153.9 (3-C_{Ar}), 149.5 (2-C_{Ar}), 134.3 (8a-C_{Ar}), 132.8 (4a-C_{Ar}), 121.5 (4-C_{Ar}), 55.7 (-O*C*H_3), 31.7 (5-C), 29.2 (8-C), 23.4 (6-C), 22.8 (7-C). HRMS (ESI, m/z) m/z calculated for $C_9H_{13}NO$ [M+H$^+$] 164.1070, found 164.1069. The data match those published in the literature.[38]

3-(Methylsulfonyl)-5,6,7,8-tetrahydroquinoline **89**

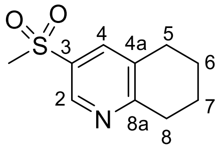

3-Bromo-5,6,7,8-tetrahydroquinoline **S17** (150 mg, 0.750 mmol, 1.00 eq.), sodium methylsulfinate (153 mg, 1.50 mmol, 2.00 eq.), Cs_2CO_3 (75.0 mg, 0.230 mmol, 0.300 eq.), *DL*-proline (27.0 mg, 0.230 mmol, 0.300 eq.), and CuI (44.0 mg, 0.230 mmol, 0.300 eq.) were suspended in dry, degassed DMSO (4.0 mL). The reaction mixture was heated at 140 °C for 3 h. The reaction mixture was diluted with deionised H_2O (30 mL). The precipitate was filtered on a sintered-glass funnel and the cake was washed with EtOAc (40 mL). The layers of the filtrate were separated, and the aqueous phase was extracted with EtOAc (3 x 30 mL), the combined organic phase was dried over anhydrous $MgSO_4$, filtered, and evaporated under reduced pressure to yield the crude material (124 mg) as pale, yellow solid. The crude was purified by column chromatography (eluent: *n*-Hex:EtOAc = 7:3) to yield the product **89** as pale, yellow solid. R_f = 0.30 (eluent: *n*-Hex:EtOAc = 7:3). Yield: 102 mg (39%).

^{1}H NMR (400 MHz, $CDCl_3$) δ 8.86 (d, *J* = 2.3 Hz, 1H, 2-H_{Ar}), 7.87 (d, *J* = 2.2 Hz, 1H, 4-H_{Ar}), 3.08 (s, 3H, -SO_2-C*H*$_3$), 3.01 (t, *J* = 6.5 Hz, 2H, 8-H_α, 8-H_β), 2.87 (t, *J* = 6.3 Hz, 2H, 5-H_α, 5-H_β), 1.99 - 1.90 (m, 2H, 7-H_α, 7-H_β), 1.89 - 1.82 (m, 2H, 6-H_α, 6-H_β). ^{13}C NMR (101 MHz, $CDCl_3$) δ 163.9 (8a-C_{Ar}), 145.4 (2-C_{Ar}), 135.6 (4-C_{Ar}), 134.2 (3-C_{Ar}), 133.4 (4a-C_{Ar}), 45.1 (-SO_2-*C*H_3), 33.0 (8-C), 28.9 (5-C), 22.6 (7-C), 22.3 (6-C). HRMS (ESI, m/z) m/z calculated for $C_9H_{13}NO_2S$ [M+H$^+$] 212.0740, found 212.0746. IR (film): v_{max}/cm^{-1} 2939 (-CH_{Ar}), 1305 (S=O), 1134, 763.

(+/-)-3-Hydroxy-3-methylindolin-2-one rac-**91**

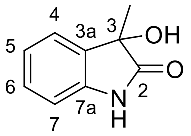

3-Methyl-2-oxindole (50.0 mg, 0.340 mmol, 1.00 eq) and potassium tert-butylate (38.0 mg, 0.340 mmol, 1.00 eq) were suspended in dry toluene (2.0 mL) under O_2 atmosphere. The suspension was stirred at rt for 24 h. The solvent was removed under reduced pressure and the crude material was purified by column chromatography (eluent: EtOAc/n-Hex = 7:3). The product was yielded as a glass-like solid. R_f = 0.59 (eluent: EtOAc/n-Hex = 7:3). Yield: 47.0 mg (85%).

^{1}H NMR (400 MHz, $CDCl_3$) δ 7.31 (d, *J* = 7.3 Hz, 1H, 4-H_{Ar}), 7.20 (dd, *J* = 8.2, 7.2 Hz, 1H, 6-H_{Ar}), 7.01 (t, *J* = 7.6 Hz, 1H, 5-H_{Ar}), 6.86 (d, *J* = 8.1 Hz, 1H, 7-H_{Ar}), 3.32 (s, 1H, -N*H*), 1.47 (s, 3H, -C*H*$_3$). ^{13}C NMR (101 MHz, $CDCl_3$) δ 182.6 (-*C*=O), 142.0 (7a-C_{Ar}), 134.4 (3a-C_{Ar}), 130.4 (6-C_{Ar}), 124.5 (4-C_{Ar}), 123.8 (5-C_{Ar}), 111.2 (7-C_{Ar}), 74.7 (-*C*-OH), 24.7 (-*C*H_3). HRMS (ESI, m/z) m/z calculated for $C_9H_{10}NO_2$ [M+Na$^+$] 186.0531, found .186.0521. The NMR spectra agree with those reported in the literature.[39]

Biocatalytically synthesized compounds.

(–)-6,7-Dihydro-5H-cyclopenta[b]pyridine-5-ol (R)-**2**, 6,7-Dihydro-5H-cyclopenta[b]pyridin-5-one **54**, (+)-6,7-Dihydro-5H-cyclopenta[b]pyridine-7-ol (R)-**55**

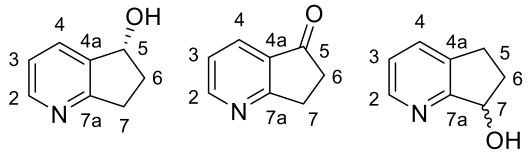

2 **54** **55**

2,3-Cyclopentenopyridine (980 µL, 1.00 g, 8.40 mmol) was dissolved in MeCN (16.4 mL) and added to a solution of lyophilized r*Aae*UPO expression supernatant (1680 U, 2.29 g) in 84 mL 100 mM KPi buffer (pH = 7.0) diluted with 66.2 mL deionised water in a three neck round bottom flask equipped with a water condenser. 1.37 mL MeOH was added, followed by 333 U of *Pp*AOx enzyme (97 µL in sucrose solution). Air was slowly bubbled through the solution during the course of the reaction (for setup see **Figure S**). The reaction was followed with gas chromatography. After 3 days, the reaction media was extracted with EtOAc (4 x 200 mL). The unified organic phase was washed with 50 mL brine, dried over $MgSO_4$, filtered and evaporated *in vacuo*. The yielded crude material was purified by column chromatography (DCM:MeOH = 20:1) and three fractions were collected containing **2**, **53** and **54**.

Data for (*R*)-**2**: Isolated as a brown oil. R_f = 0.19 (eluent: DCM:MeOH = 20:1). Yield: 34%. *ee* = 88%, measured by chiral HPLC (Daicel Chiralpak(R) AD-H column), see ESI **Section 13** for details and chromatograms.

^{1}H NMR (400 MHz, $CDCl_3$) δ 8.24 (d, *J* = 4.9 Hz, 1H, 2-H_{Ar}), 7.64 (d, *J* = 7.5 Hz, 1H, 4-H_{Ar}), 7.05 (dd, *J* = 7.5, 4.9 Hz, 1H, 3-H_{Ar}), 5.19 (t, *J* = 6.6 Hz, 1H, 5-H), 4.86 (s, 1H, -O*H*), 2.99 (ddd, *J* = 16.5, 8.2, 4.2 Hz, 1H, 7-H_α), 2.78 (dt, *J* = 16.5, 8.2 Hz, 1H, 7-H_β), 2.51–2.41 (m, 1H, 6-H_α), 1.92 (dtd, *J* = 13.6, 8.2, 6.6 Hz, 1H, 6-H_β). ^{13}C NMR (101 MHz, $CDCl_3$) δ 163.8 (7a-C_{Ar}), 148.8 (2-C_{Ar}), 138.9 (4a-C_{Ar}), 132.9 (4-C_{Ar}), 121.7 (3-C_{Ar}), 73.7 (5-C), 34.2 (7-C), 31.5 (6-C). $[\alpha]_D^{23}$ = –25.7 ($CHCl_3$, c = 2.32, n = 3). The NMR data match those of the authentic standard (*vide supra*).

Data for **54**: Isolated as a red oil. R_f = 0.41 (eluent: DCM:MeOH = 20:1). Yield: 4%.

^{1}H NMR (400 MHz, $CDCl_3$) δ 8.80 (dd, *J* = 4.9, 1.8 Hz, 1H, 2-H_{Ar}), 8.02 (dd, *J* = 7.8, 1.8 Hz, 1H, 4-H_{Ar}), 7.32 (dd, *J* = 7.8, 4.9 Hz, 1H, 3-H_{Ar}), 3.32 - 3.22 (m, 2H, 7-H_α, 7-H_β), 2.81 - 2.74 (m, 2H, 6-H_α, 6-H_β). ^{13}C NMR (101 MHz, $CDCl_3$) δ 205.1 (5-C), 174.5 (7a-C_{Ar}), 155.9 (2-C_{Ar}), 132.2 (4a-C_{Ar}), 130.5 (4-C_{Ar}), 122.6 (3-C_{Ar}), 35.9 (7-C_{Ar}), 28.8 (6-C_{Ar}). The NMR data match those of the authentic standard (*vide supra*).

Data for *(R)*-**55**: Isolated as a pale yellow amorphous material. R_f = 0.26 (eluent: DCM:MeOH = 20:1). Yield: 6%. *ee* = >99%, measured by chiral HPLC (Daicel Chiralpak(R) AD-H column), see ESI Section 13 for details and chromatograms.

^{1}H NMR (400 MHz, $CDCl_3$) δ 8.40 (d, *J* = 5.0 Hz, 1H, 2-H_{Ar}), 7.57 (d, *J* = 7.6 Hz, 1H, 4-H_{Ar}), 7.13 (dd, *J* = 7.6, 5.0 Hz, 1H, 3-H_{Ar}), 5.25 (t, *J* = 6.7 Hz, 1H, 5-H), 3.05 (ddd, *J* = 16.1, 8.9, 4.1 Hz, 1H, 7-H_α), 2.82 (dt, *J* = 16.1, 7.8 Hz, 1H, 7-H_β), 2.61 - 2.46 (m, 1H, 6-H_α), 2.16 - 1.96 (m, 1H, 6-H_β). ^{13}C NMR (101 MHz, $CDCl_3$) δ 165.1 (7a-C_{Ar}), 147.8 (2-C_{Ar}), 136.6 (4a-C_{Ar}), 133.6

(4-C_{Ar}), 122.8 (3-C_{Ar}), 74.4 (5-C), 32.9 (7-C), 27.6 (6-C). HRMS (ESI, m/z) m/z calculated for C_8H_9NO [M+Na^+] 158.0576, found 158.0576. $[\alpha]_D^{23}$ = 59.6 ($CHCl_3$, c = 0.95, n = 3).

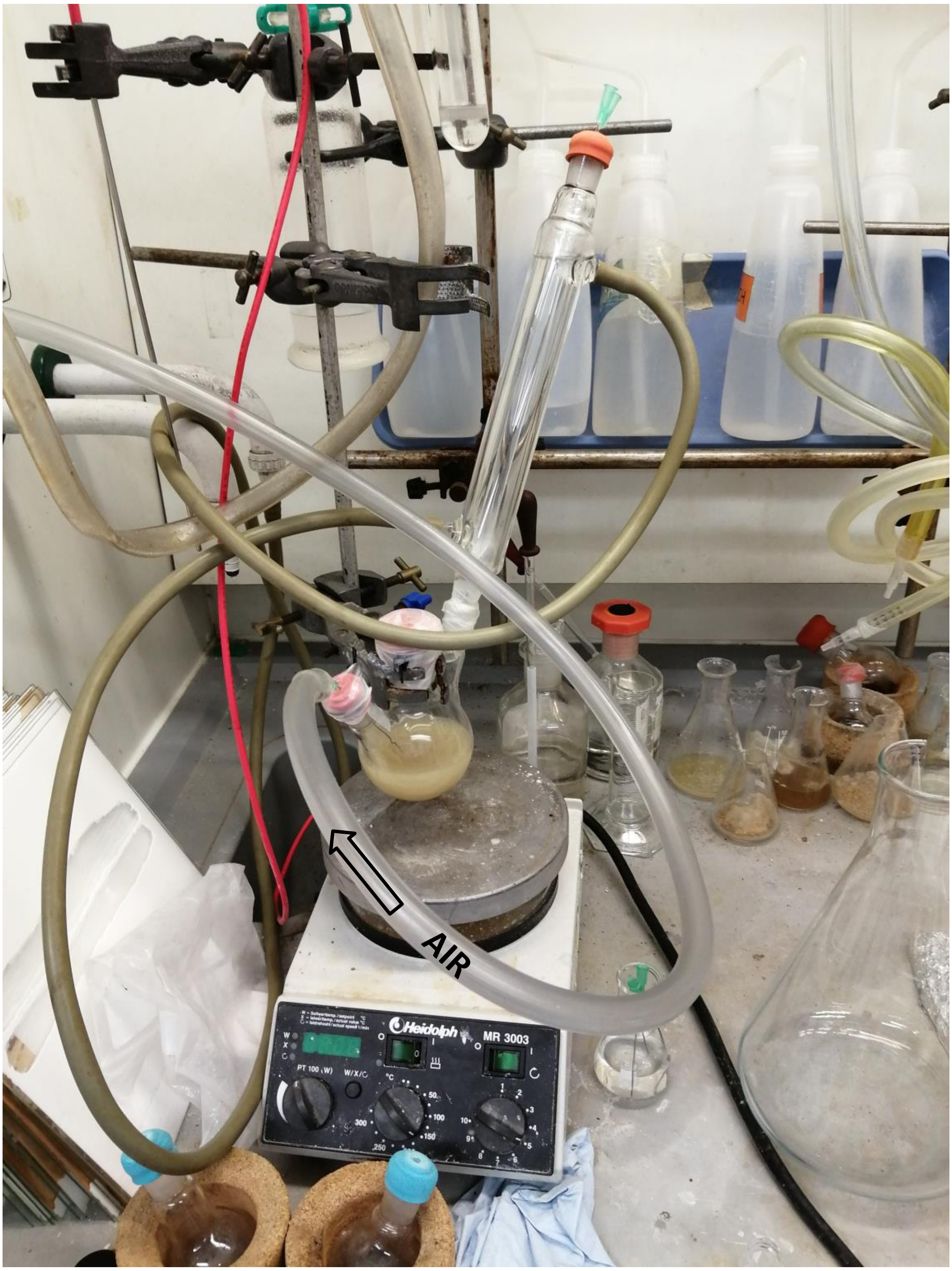

Figure S3. Setup used for the transformation of 2,3-cyclopentenopyridine using *in situ* H_2O_2 generation. Air was constantly bubbled through the reaction media during the course of the reaction at a slow rate. An air condenser was used to minimize the loss of the volatile starting material.

(R)-1-(Pyridine-3-yl)-ethan-1-ol **20**, *3-Acetylpyridine* **21**

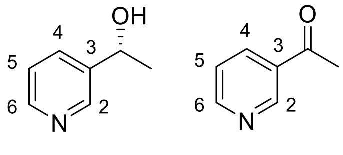

Lyophilized r*Aae*UPO expression supernatant containing (422.7 mg, 409 U) was dissolved in a round bottomed flask in 100 mM pH = 7.0 KPi buffer (46.5 mL) and the solution was diluted with deionised water (33 mL). *Pp*AOx stock solution (26.8 µL in sucrose, 92 U) was diluted with pH = 7.0 100 mM KPi buffer (2.3 mL) and added to the reaction mixture (1 U mL^{-1} final concentration), followed by the addition of 3-ethylpyridine (100 mg, 0.930 mmol, 1.00 eq) in MeCN (9.3 mL). The reaction was initiated by the addition of 1.50 mL MeOH (1.19 g, 37.1 mmol, 400 mM). The solution was stirred for 24 h at rt. The reaction medium was extracted with EtOAc (3 x 100 mL), the combined organic phase was dried over anhydrous $MgSO_4$, filtered and evaporated *in vacuo*. The crude material was purified by column chromatography (eluent: EtOAc/n-Hex = 3:1) to yield (*R*)-**20** (pale yellow oil, R_f = 0.39 (eluent: EtOAc/n-Hex = 4:1), yield: 40 mg (35%)) and **21** (pale yellow oil, yield: 56 mg (50%).) as products.

Data for (*R*)-**20**: *ee* = 98%, measured by chiral GC (Supelco β-DEX^{TM} capillary column), see ESI **Section 13** for details and chromatograms.

^{1}H NMR (400 MHz, $CDCl_3$) δ 8.44 (d, *J* = 1.8 Hz, 1H, 2-H_{Ar}), 8.36 (dd, *J* = 4.2, 1.8 Hz, 1H, 6-H_{Ar}), 7.71 (dt, *J* = 7.8, 1.8 Hz, 1H, 4-H_{Ar}), 7.24 (dt, *J* = 7.8, 4.2 Hz, 1H, 5-H_{Ar}), 4.89 (q, *J* = 6.6 Hz, 1H, -C*H*(OH)-), 3.58 (broad s, 1H, -O*H*), 1.47 (d, *J* = 6.6 Hz, 3H, C*H*3).^{13}C NMR (101 MHz, $CDCl_3$) δ 148.2 (2/6-C_{Ar}), 147.2 (2/6-C_{Ar}), 141.7 (3-C_{Ar}), 133.6 (4-C_{Ar}), 123.7 (5-C_{Ar}), 67.7 (-*C*H(OH)-), 25.3 (*C*H_3). HRMS (ESI, m/z) m/z calculated for C_7H_9NO [M+H$^+$] 124.0762, found 124.0764. The data match those reported in the literature.[8]

Data for **21**: ^{1}H NMR (400 MHz, $CDCl_3$) δ 9.17 (d, *J* = 2.3 Hz, 1H, 2-H_{Ar}), 8.79 (dd, *J* = 4.6 Hz, 2.2 Hz, 1H, 6-H_{Ar}), 8.24 (dt, *J* = 7.9, 2.2 Hz, 1H, 4-H_{Ar}), 7.43 (dd, *J* = 7.9, 4.6 Hz, 1H, 5-H_{Ar}), 2.65 (s, 3H, -C*H*3). ^{13}C NMR (101 MHz, $CDCl_3$) δ 196.9 (*C*O), 153.7 (2-C_{Ar}), 150.1 (6-C_{Ar}), 135.7 (4-C_{Ar}), 132.4 (3-C_{Ar}), 128.8 (5-C_{Ar}), 26.9 (*C*H_3). HRMS (ESI, m/z) m/z calculated for C_7H_7NO [M+H$^+$] 122.0606, found 122.0601. The data match those reported in the literature.[40]

(R)-1-(Pyridine-2-yl)ethanol (*R*)-**22**, *2-Ethylpyridine-1-oxide* **57**

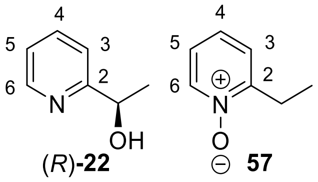

Lyophilized r*Aae*UPO expression supernatant (1.73 g, 1670 U) was dissolved in a round bottomed flask in pH = 7.0 KPi buffer (100 mM, 190 mL) and the solution was diluted with deionised water (135 mL). *Pp*AOx stock solution (166 µL, 570 U) was diluted with pH = 7.0 KPi buffer (100 mM, 9.5 mL) and added to the mixture (1.5 U mL^{-1} final concentration). 2-Ethylpyridine (810 mg, 7.60 mmol, 1.00 eq) was added in MeCN (38 mL). The reaction was initiated by the addition of 6.1 mL MeOH (400 mM final concentration). The reaction was

stirred at rt for 24h and then it was extracted with EtOAc (3 x 400 mL). The unified organic phase was dried over anhydrous $MgSO_4$, filtered, and evaporated *in vacuo*. The crude material was purified by column chromatography (eluent: EtOAc) to yield (*R*)-**22** (dense yellow oil, R_f = 0.15 (eluent: n-Hex/EtOAc = 5:1 (0.5 % TEA)), yield: 178 mg (19%).) and **57** (white solid, R_f = 0.42 (eluent $CHCl_3$/MeOH = 19:1), yield: 150 mg (16%).).

Data for (*R*)-**22**: *ee* = >99%, measured by chiral GC (Supelco β-DEX^{TM} capillary column), see ESI **Section 13** for details and chromatograms.

^{1}H NMR (400 MHz, $CDCl_3$) δ 8.53 (d, *J* = 4.7 Hz, 1H, 6-H_{Ar}), 7.69 (td, *J* = 7.9, 1.3 Hz, 1H, 4-H_{Ar}), 7.27 (d, *J* = 7.9 Hz, 1H, 3-H_{Ar}), 7.20 (dd, *J* = 7.9, 4.7 Hz, 1H, 5-H_{Ar}), 4.89 (q, *J* = 6.6 Hz, 1H, -C*H*-OH), 1.50 (s, 3H, -C*H*$_3$). ^{13}C NMR (101 MHz, $CDCl_3$) δ 163.1 (2-C_{Ar}), 148.2 (6-C_{Ar}), 136.9 (4-C_{Ar}), 122.4 (3-C_{Ar}), 119.9 (5-C_{Ar}), 68.9 (-*C*H-OH), 24.4 (-*C*H_3). HRMS (ESI, m/z) m/z calculated for C_7H_9NO [M+H$^+$] 124.0762, found 124.0755. The data match those reported in the literature.[9]

Data for **57**: ^{1}H NMR (400 MHz, $CDCl_3$) δ 8.26 (d, J = 6.5 Hz, 1H, 6-H_{Ar}), 7.25 - 7.22 (m, 2H, 3-H_{Ar}, 5-H_{Ar}), 7.16 - 7.11 (m, 1H, 4-H_{Ar}), 2.95 (q, *J* = 7.5 Hz, 2H, -C*H*$_2$-), 1.31 (td, *J* = 7.5, 1.6 Hz). ^{13}C NMR (101 MHz, $CDCl_3$) δ 153.9 (2-C_{Ar}), 139.6 (6-C_{Ar}), 126.0 (5-C_{Ar}), 124.6 (4-C_{Ar}), 123.4 (3-C_{Ar}), 23.80 (-C*H*$_2$-), 10.7 (-C*H*$_3$). HRMS (ESI, m/z) m/z calculated for C_7H_9NO [M+H$^+$] 124.0762, found 124.0757. The data match those reported in the literature.[41]

(R)-1-(2-Pyridyl)propan-1-ol (*R*)-**27**, *(2-Pyridyl)propan-2-ol* **58**, *2-Propylpyridine-1-oxide* **59**

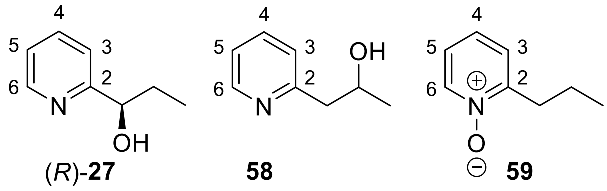

(*R*)-**27** **58** **59**

Lyophilized r*Aae*UPO expression supernatant (227 mg, 220 U) was dissolved in a conical flask in pH = 7.0 KPi buffer (100 mM, 25 mL) and the solution was diluted with deionised water (18 mL). *Pp*AOx stock solution (3.64 µL in sucrose solution, 12.5 U) was diluted with pH = 7.0 KPi buffer (100 mM, 1.25 mL) and the solution was added to the reaction (0.25 U mL^{-1} final concentration). This was followed by the addition of 2-propylpyridine **15** (121 mg, 1.00 mmol, 1.00 eq) in MeCN (5.0 mL). The reaction was initiated by the addition of MeOH (0.81 mL, 400 mM final concentration. The solution was shaken in an orbital shaker at 200 rpm at rt for 24 h. The reaction media was extracted with EtOAc (3 x 50 mL), the organic phase was dried over anhydrous $MgSO_4$ and evaporated *in vacuo*. The crude material was purified by column chromatography (EtOAc to EtOAc/MeOH = 8:2) to yield (*R*)-**27** (dense pale yellow oil, R_f = 0.49 (eluent: EtOAc), yield: 44 mg (32%)), **58** (white solid, R_f = 0.35 (eluent: EtOAc), yield: 19 mg (14%) and **59** (white solid, R_f = 0.10 (eluent: EtOAc), yield: 70 mg (51%).).

Data for (*R*)-**27**: *ee* = >99%, measured by chiral HPLC (Daicel Chiralcel$^{(R)}$ OD-H column), see ESI **Section 13** for details and chromatograms.

^{1}H NMR (400 MHz, $CDCl_3$) δ 8.51 (d, *J* = 5.2 Hz, 1H, 6-H_{Ar}), 7.66 (t, *J* = 7.7 Hz, 1H, 4-H_{Ar}), 7.25 (d, *J* = 7.7 Hz, 1H, 3-H_{Ar}), 7.18 (dd, *J* = 7.7, 5.2 Hz, 1H, 5-H_{Ar}), 4.75 - 4.62 (m, 1H, -C*H*(OH)-), 4.28 (s, 1H, -O*H*), 1.96 - 1.80 (m, 1H, -HC*H*-) 1.79 - 1.62 (m, 1H, -*H*CH), 0.93 (t, *J* = 7.4 Hz, 3H, -C*H*$_3$). ^{13}C NMR (101 MHz, $CDCl_3$) δ 162.1 (2-C_{Ar}), 148.3 (6-C_{Ar}), 136.8 (4-C_{Ar}), 122.3 (3/5-C_{Ar}), 120.5 (3/5-C_{Ar}), 73.9 (-*C*H(OH)-), 31.4 (-*C*H_2-), 9.5 (-*C*H_3). HRMS (ESI,

m/z) m/z calculated for $C_8H_{11}NO$ [M+H$^+$] 138.0919, found 138.0920. The data match those reported in the literature.[42]

Data for **58**: ^{1}H NMR (400 MHz, $CDCl_3$) δ 8.48 (d, *J* = 5.5 Hz, 1H, 6-H$_{Ar}$), 7.61 (t, *J* = 7.6 Hz, 1H, 4-H$_{Ar}$), 7.22 - 7.12 (m, 1H, 5-H$_{Ar}$), 7.12 (d, *J* = 7.7 Hz, 1H, 3-H$_{Ar}$), 5.16 (s, 1H, -O*H*), 4.26 – 4.18 (m, 1H, -C*H*(OH)-), 2.99 - 2.77 (m, 2H, -C*H*$_2$-), 1.27 (d, *J* = 6.2 Hz, 3H, -C*H*$_3$). ^{13}C NMR (101 MHz, $CDCl_3$) δ 160.4 (2-C$_{Ar}$), 148.7 (6-C$_{Ar}$), 136.9 (4-C$_{Ar}$), 123.8 (3-C$_{Ar}$), 121.6 (5-C$_{Ar}$), 67.3 (-*C*H-OH), 45.0 (-*C*H$_2$-), 23.1 (-*C*H$_3$). HRMS (ESI, m/z) m/z calculated for $C_8H_{11}NO$ [M+H$^+$] 138.0919, found 138.0921.

Data for **59**: ^{1}H NMR (400 MHz, $CDCl_3$) δ 8.26 (d, *J* = 6.5 Hz, 1H, 4-H$_{Ar}$), 7.24–7.18 (m, 2H, 3/5 - H$_{Ar}$, 6-H$_{Ar}$), 7.16 - 7.09 (m, 1H, 3/5-H$_{Ar}$), 2.90 (t, *J* = 7.5 Hz, 2H, -C*H*$_2$-CH$_2$-), 1.76 (sext, *J* = 7.5 Hz, 2H, -CH$_2$-C*H*$_2$-), 1.02 (t, *J* = 7.5 Hz, 3H, C*H*$_3$). ^{13}C NMR (101 MHz, $CDCl_3$) δ 152.7 (2-C$_{Ar}$), 139.8 (6-C$_{Ar}$), 125.8 (5-C$_{Ar}$), 125.5 (3-C$_{Ar}$), 123.4 (4-C$_{Ar}$), 32.5 (-*C*H$_2$-CH$_2$-), 19.5 (-CH$_2$-*C*H$_2$-), 14.0 (-*C*H$_3$). HRMS (ESI, m/z) m/z calculated for $C_8H_{11}NO$ [M+H$^+$] 138.0919, found 138.0914. The data match those reported in the literature.[32]

(6-Methoxypyridine-3-yl)-methanol **28**, *6-Methoxy-3-pyridinecarboxaldehyde* **29**

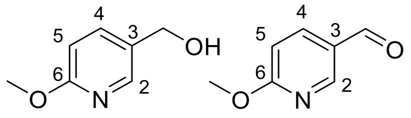

28 **29**

Lyophilized r*Aae*UPO expression supernatant (151 mg, 146 U) was dissolved in pH = 7.0 KPi buffer (100 mM, 41.5 mL) and the solution was diluted with deionised water (16.6 mL). 2-methoxy-5-methylpyridine (100 mg, 0.830 mmol, 1.00 eq.) was added in MeCN (8.3 mL). 30% aqueous H_2O_2 solution (132 µL, 1.30 mmol, 1.56 eq.) was diluted in deionised H_2O (13.5 mL) and the solution was added dropwise to the reaction over 30 h at rt. The reaction was stopped by mixing the reaction media with 30 mL EtOAc for 2 h. The phases were separated and the aqueous phase was extracted with EtOAc (2x30 mL). To aid phase separation, the mixture was filtered through a celite pad multiple times. The unified organic layer was washed with 50 mL brine, dried over anhydrous $MgSO_4$, filtered, and evaporated under reduced pressure. The crude material was separated by column chromatography (eluent: EtOAc/n-Hex = 2:1) to yield **28** (pale yellow oil, R_f = 0.11 (eluent: EtOAc/n-Hex = 2:1), yield: 36 mg (31%)) and **29** (yellow needles, R_f = 0.42 (eluent: EtOAc/n-Hex = 2:1), m.p. 42 °C, yield: 53 mg (47%).).

Data for **28**: ^{1}H NMR (400 MHz, $CDCl_3$) δ 8.11 (d, *J* = 2.4 Hz, 1H, 6-H$_{Ar}$), 7.62 (dd, *J* = 8.5, 2.4 Hz, 1H, 4-H$_{Ar}$), 6.75 (d, *J* = 8.5 Hz, 1H, 3-H$_{Ar}$), 4.62 (s, 2H, -C*H*$_2$-OH), 3.93 (s, 3H, -OC*H*$_3$). ^{13}C NMR (101 MHz, $CDCl_3$) δ 164.1 (6-C$_{Ar}$), 145.9 (2-C$_{Ar}$), 138.6 (4-C$_{Ar}$), 129.1 (3-C$_{Ar}$), 111.1 (5-C$_{Ar}$), 62.7 (-*C*H$_2$-OH), 53.7 (-O*C*H$_3$). HRMS (ESI, m/z) m/z calculated for $C_7H_9NO_2$ [M+H$^+$] 140.0712, found 140.0705. The data match those reported in the literature.[43]

Data for **29**: ^{1}H NMR (400 MHz, $CDCl_3$) δ 9.96 (s, 1H, -C*H*=O), 8.64 (d, *J* = 2.4 Hz, 1H, 2-H$_{Ar}$), 8.07 (dd, *J* = 9.1, 2.4 Hz, 1H, 4-H$_{ar}$), 6.85 (d, *J* = 9.1 Hz, 1H, 5-H$_{Ar}$), 4.03 (s, 3H, -OC*H*$_3$). ^{13}C NMR (101 MHz, $CDCl_3$) δ 189.7 (-*C*H=O), 167.8 (6-C$_{Ar}$), 153.1 (2-C$_{Ar}$), 137.6 (3-C$_{Ar}$), 126.8 (4-C$_{Ar}$), 112.3 (5-C$_{Ar}$), 54.5 (-O*C*H$_3$). HRMS (ESI, m/z) m/z calculated for $C_7H_7NO_2$ [M+H$^+$] 138.0555, found 138.0549. The data match those reported in the literature.[44]

3,4-Dihydroisoquinoline-1(2H)-one **38**, *Isoquinoline-N-oxide* **44**, *3,4-Dihydroisoquinoline* **S19**, *Tetrahydrobiisoquinylidene* **39**

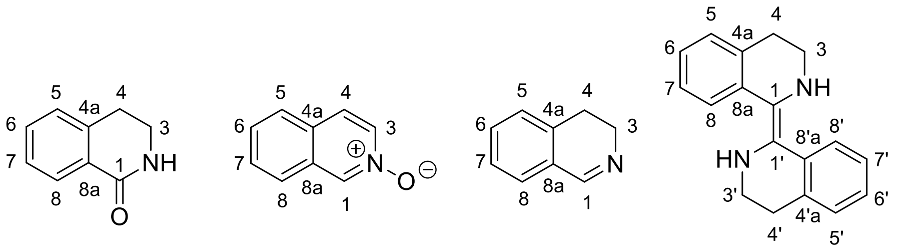

38 **44** **S19** **39**

Lyophilized r*Aae*UPO expression supernatant (114 mg ,110 U) was dissolved in pH = 7.0 KPi buffer (100 mM, 25 mL) in a 100 mL round bottom flask. The solution was diluted with deionised water (10 mL) and 1,2,3,4-tetrahydroisoquinoline (190 µL, 200 mg, 1.50 mmol, 1.00 eq) was added to the solution in MeCN (5 mL). 30% H_2O_2 solution (153 µL, 51.0 mg, 1.50 mmol, 1.00 eq.) was diluted in deionised H_2O (5.0 mL) and the solution was added to the reaction dropwise over 25 h at rt. The reaction was stirred at this temperature for a further 12 h and then the reaction media was extracted with EtOAc (3 x 100 mL), followed by *n*-BuOH (1 x 50 mL). The combined organic phase was dried over anhydrous $MgSO_4$ and evaporated under reduced pressure. The crude material (228 mg) was purified by column chromatography (EtOAc/MeOH = 9:1 to EtOAc/MeOH = 8:2). Some of the products were unstable on silica gel. Aside from the recovered starting material (109 mg, 55% recovered material) and minor amounts of oligomers, five fractions were collected, containing **38** (brown oil, R_f = 0.67 (eluent: EtOAc/MeOH = 9:1), yield: 9.9 mg (4.5 %)), **44** (brown oil, R_f = 0.14 (eluent: EtOAc/MeOH = 9:1), yield: 5.2 mg (2.4%)), **S19** (yellow oil, R_f = 0.57 (eluent: EtOAc/MeOH = 9:1), yield: 15 mg (7.6%)), **39** (yellow oil, R_f = 0.26 (eluent: EtOAc/MeOH = 9:1), yield: 15 mg (7.6%)).

Data for **38**: ^{1}H NMR (400 MHz, $CDCl_3$) δ 8.05 (dd, *J* = 7.7, 1.2 Hz, 1H, 8-H_{Ar}), 7.44 (td, *J* = 7.6, 1.2 Hz, 1H, 6-H_{Ar}), 7.34 (t, *J* = 7.6 Hz, 1H, 7-H_{Ar}), 7.21 (d, *J* = 7.7 Hz, 1H, 5-H_{Ar}), 7.00 (s, 1H, -N*H*-), 3.56 (td, *J* = 6.6, 2.9 Hz, 2H, 3-H_α, 3-H_β), 2.99 (t, *J* = 6.6 Hz, 2H, 4-H_α, 4-H_β). ^{13}C NMR (101 MHz, $CDCl_3$) δ 166.8 (1-C_{Ar}), 139.0 (8a-C_{Ar}), 132.3 (6-C_{Ar}), 128.9 (4a-C_{Ar}), 128.1 (8-C_{Ar}), 127.4 (5/7-C_{Ar}), 127.2 (5/7-C_{Ar}), 40.3 (3-C_{Ar}), 28.3 (4-C_{Ar}). HRMS (ESI, m/z) m/z calculated for C_9H_8NO [M+H$^+$] 146.0606, found 146.0607.[45]

Data for **44**: ^{1}H NMR (400 MHz, $CDCl_3$) δ 8.79 (s, 1H, 1-H_{Ar}), 8.14 (dd, *J* = 6.9, 2.0 Hz, 1H, 2-H_{Ar}), 7.82 - 7.71 (m, 2H, 5-H_{Ar}, 8-H_{Ar}), 7.67 (d, *J* = 7.3 Hz, 1H, 4-H_{Ar}), 7.64 – 7.56 (m, 2H, 6-H_{Ar}, 7-H_{Ar}). ^{13}C NMR (101 MHz, $CDCl_3$) δ 136.8 (1-C_{Ar}), 136.5 (2-C_{Ar}), 129.7 (6/7-C_{Ar}), 129.6 (4a/8a-C_{Ar}), 129.4 (6/7-C_{Ar}), 129.2 (4a/8a-C_{Ar}), 126.8 (5/8-C_{Ar}), 125.3 (5/8-C_{Ar}), 124.5 (4-C_{Ar}). HRMS (ESI, m/z) m/z calculated for C_9H_7NO [M+H$^+$] 145.0528, found 145.0530. The data match those reported in the literature.[46]

Data for **S19**: ^{1}H NMR (400 MHz, $CDCl_3$) δ 8.30 (broad s, 1H, 1-H_{Ar}), 7.33 (td, *J* = 7.0, 2.8 Hz, 1H, 6/7-H_{Ar}), 7.27 - 7.24 (m, 2H, 5-H_{Ar}), 7.13 (d, *J* = 7.5 Hz, 1H, 8-H_{Ar}), 3.72 (t, *J* = 7.6 Hz, 2H, 3-H_α, 3-H_β), 2.73 (t, *J* = 7.6 Hz, 2H, 4-H_α, 4-H_β). ^{13}C NMR (101 MHz, $CDCl_3$) δ 160.7 (1-C_{Ar}), 136.3 (8-C_{Ar}), 131.4 (6/7-C_{Ar}), 127.5 (6/7-C_{Ar}), 127.2 (5-C_{Ar}), 46.9 (3-C_{Ar}), 25.0 (4-C_{Ar}). HRMS (ESI, m/z) m/z calculated for C_9H_9N [M+H$^+$] 132.0808, found 132.0806.[47]

Data for **39**: ^{1}H NMR (400 MHz, $CDCl_3$) δ 7.77 (s, 2H, -N*H*-, -N*H'*-), 7.27 (td, *J* = 7.5, 2.3 Hz, 2H, 6/7-H_{Ar}, 6'/7'-H_{Ar}), 7.25 (td, *J* = 7.5, 2.3 Hz, 2H, 6/7H_{Ar}, 6'/7'-H'_{Ar}), 7.20 (dd, *J* = 7.4, 2.2 Hz, 2H, 5/8-H_{Ar}, 5'/8'-H_{Ar}), 7.12 (dd, *J* = 7.4, 2.2 Hz, 2H, 5/8-H_{Ar}, 5'/8'-H_{Ar}), 4.09 (t, *J* = 7.8 Hz, 4H, 4-H_α, 4-H_β, 4'-H_α, 4'-H_β), 3.16 (t, *J* = 7.8 Hz, 3-H_α, 3-H_β, 3'-H_α, 3'-H_β). ^{13}C NMR (101 MHz, $CDCl_3$) δ 134.8 (4a-C_{Ar}, 4'a-C_{Ar}), 130.2 (8a-C_{Ar}, 8'a-C_{Ar}), 129.7 (5-C_{Ar}, 5'-C_{Ar}), 128.2 (1-C, 1'-C), 127.7 (7-C_{Ar}, 7'-C_{Ar}), 127.3 (8-C_{Ar}, 8'-C_{Ar}), 125.7 (6-C_{Ar}, 6'-C_{Ar}), 57.8 (3-C, 3'-C), 27.7 (4-C, 4'-C). HRMS (ESI, m/z) no molecular ion found under ESI, or APCI conditions.

1-(6-Methylpyridin-3-yl)-ethanol **61a**, *(5-Ethylpyridin-2-yl)-methanol* **61b**

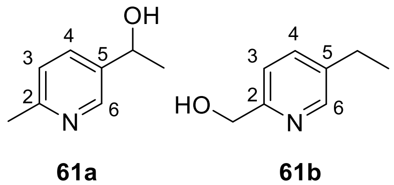

61a **61b**

2-Methyl-5-ethylpyridine **60** (32.6 µL, 30.0 mg, 0.248 mmol, 1.00 eq.) was dissolved in MeCN (2.48 mL) and the solution was added to the solution of lyophilised r*Aae*UPO expression supernatant (113 mg, 101 U) in pH = 7.0 KPi buffer (100 mM, 21.3 mL). 30% H_2O_2 solution (25.3 µL, 8.43 mg, 0.248 mmol, 1.00 eq.) was diluted in deionised H_2O (1.0 mL) and it was added to the reaction media dropwise over 13 h at rt. The reaction media was extracted with EtOAc (4 x 20 mL), the unified organic phase was washed with brine (10 mL), dried over anhydrous $MgSO_4$, filtered, and evaporated under reduced pressure to yield the crude as colourless oil. Preparative thin-layer chromatography afforded **61a** as white solid (R_f = 0.13 (eluent: EtOAc:*n*-Hex = 7:3), yield: 25 mg (73%)). **61b** was also separated as a minor constituent (yellow oil, R_f = 0.33 (eluent: EtOAc:*n*-Hex = 7:3), yield: <1 mg (<3%)).

Data for **61a**: ^{1}H NMR (400 MHz, $CDCl_3$) δ 8.34 (d, *J* = 2.4 Hz, 1H, 6-H_{Ar}), 7.61 (dd, *J* = 8.0, 2.4 Hz, 1H, 4-H_{Ar}), 7.10 (d, *J* = 8.0 Hz, 1H, 3-H_{Ar}), 4.88 (q, *J* = 6.6 Hz, 1H, -C*H*(OH)-), 2.49 (s, 3H, -CH(OH)-C*H*$_3$), 1.47 (d, *J* = 6.2 Hz, 3H, Py-C*H*$_3$). ^{13}C NMR (101 MHz, $CDCl_3$) δ 157.3 (2-C_{Ar}), 146.6 (6-C_{Ar}), 138.5 (5-C_{Ar}), 133.9 (4-C_{Ar}), 123.3 (3-C_{Ar}), 67.8 (-C*H*(OH)-), 25.2 (-CH(OH)-*C*H_3), 23.9 (Py-*C*H_3). HRMS (ESI, m/z) m/z calculated for $C_8H_{11}NO$ [M+H$^+$] 138.0913, found 138.0914. IR (film): v_{max}/cm^{-1} 3230 (O-H), 2972 (C-H_{Ar}), 2925 (C-H_{Ar}), 1493 (C-N), 1097 (C-O), 1076 (C-O), 901, 834, 737. The data match those published in the literature.[48]

Data for **61b**: HRMS (ESI, m/z) m/z calculated for $C_8H_{11}NO$ [M+H$^+$] 138.0913, found 138.0914. IR (film): v_{max}/cm^{-1} 3290 (O-H), 2924 (C-H_{Ar}), 2854 (C-H_{Ar}), 1456 (C-N), 1150, 800.

(6-Methylpyridin-3-yl)-methanol **63a**, *6-Methylnicotinaldehyde* **63b**, 6-Methylnicotinic acid **63c**

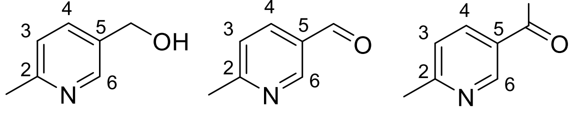

63a **63b** **63c**

2,5-Dimethylpyridine **62** (40.0 mg, 0.373 mmol, 1.00 eq.) was dissolved in MeCN (3.73 mL) and the solution was added to a solution of lyophilised r*Aae*UPO expression supernatant in a mixture of pH = 7.0 KPi buffer (18.7 mL) and deionised H_2O (13.9 mL). 30% aqueous H_2O_2

(38.1 µL, 12.6 mg, 0.373 mmol, 1.00 eq.) was diluted in deionised H_2O (1.0 mL) and the solution was added to the reaction mixture dropwise over 13 h at rt. The reaction was extracted with EtOAc (3 x 50 mL) and the unified organic phase was dried over anhydrous $MgSO_4$, filtered, and evaporated *in vacuo* to yield the crude material as transparent oil (19 mg). The crude material contained starting material, **63a**, **63b**, and the acid **63c** in 48%, 20% and 16%, yield respectively (^{1}H NMR relative amounts). The crude was separated by column chromatography (*n*-Hex:EtOAc = 1:1) to yield two fractions. The major fraction contained (6-Methylpyridin-3-yl)-methanol **63a** (R_f = 0.05 (eluent: *n*-Hex:EtOAc = 1:1), 4.3 mg (9.4%)), whilst the minor fraction contained 6-methylnicotinaldehyde **63b** (R_f = 0.34 (eluent: *n*-Hex:EtOAc = 1:1), 3.5 mg (7.7%)).

Data for **63a**: ^{1}H NMR (400 MHz, $CDCl_3$) δ 8.56 (s, 1H, 6-H_{Ar}), 7.75 (dd, *J* = 8.0, 2.1 Hz, 1H, 4-H_{Ar}), 7.26 (d, *J* = 8.0 Hz, 1H, 3-H_{Ar}), 4.74 (s, 2H, -C*H*$_2$OH), 2.67 (s, 3H, Py-C*H*$_3$). ^{13}C NMR (101 MHz, $CDCl_3$) δ 156.5 (2-C_{Ar}), 146.0 (6-C_{Ar}), 137.4 (4-C_{Ar}), 134.9 (5-C_{Ar}), 124.2 (3-C_{Ar}), 62.3 (-*C*H_2OH), 23.1 (Py-*C*H_3). HRMS (ESI, m/z) m/z calculated for C_7H_9NO [M+H$^+$] 124.0757, found 124.0756. The data match those reported in the literature.[49]

Data for **63b**: ^{1}H NMR (400 MHz, $CDCl_3$) δ 10.07 (s, 1H, -C*H*O), 8.96 (s, 1H, 6-H_{Ar}), 8.09 (dd, *J* = 8.3, 2.2 Hz, 1H, 4-H_{Ar}), 7.35 (d, *J* = 8.3 Hz, 1H, 3-H_{Ar}), 2.68 (s, 3H, Py-C*H*$_3$). HRMS (ESI, m/z) m/z calculated for C_7H_7NO [M+H$^+$] 122.0600, found 122.0598. The data match those reported in the literature.[50]

The acid was not isolated from the column, but it was observed in the crude ^{1}H NMR spectrum and its spectroscopic features agreed with those described for 6-methylnicotinic acid **63c** in the literature.[51]

(5-Methylpyridin-3-yl)-methanol **65a**, *5-Methylnicotinaldehyde* **65b**

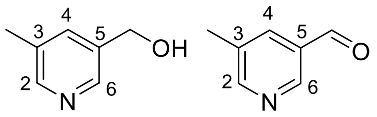

65a **65b**

3,5-Dimethylpyridine **64** (35.1 µL, 33.0 mg, 0.307 mmol, 1.00 eq.) was dissolved in MeCN (3.07 mL) and the solution was added to a solution of lyophilised r*Aae*UPO expression supernatant in a mixture of pH = 7.0 KPi buffer (15.4 mL) and deionised H_2O (11.3 mL). 30% aqueous H_2O_2 (31.3 µL, 10.4 mg, 0.307 mmol, 1.00 eq.) was diluted in deionised H_2O (1.0 mL) and the solution was added to the reaction mixture dropwise over 13 h at rt. The reaction was extracted with EtOAc (3 x 30 mL), and the unified organic phase was dried over anhydrous $MgSO_4$, filtered, and evaporated *in vacuo* to yield the crude material as transparent oil (18 mg). The crude material contained starting material 3,5-dimethylpyridine, alcohol **65a**, and aldehyde **65b**, in a 1:1.8:1 ratio (^{1}H NMR relative amounts). This corresponds to 24% and 13% yields for the alcohol and the aldehyde, respectively (calculated according based on the mass of the crude material).

Data for **65a**: ^{1}H NMR (400 MHz, $CDCl_3$) δ 8.40 - 8.27 (m, 2H, 2-H_{Ar}, 6-H_{Ar}), 7.54 (s, 1H, 4-H_{Ar}), 4.67 (s, 2H, -C*H*$_2$OH), 3.84 (s, 1H, -O*H*), 2.31 (s, 3H, Py-C*H*$_3$). ^{13}C NMR (101 MHz, $CDCl_3$) δ 148.9 (2/6-C_{Ar}), 145.4 (2/6-C_{Ar}), 135.8 (4-C_{Ar}), 134.2, 131.2, 62.3 (-*C*H_2-OH), 18.4

(Py-CH_3). HRMS (ESI, m/z) m/z calculated for C_7H_9NO [M+H$^+$] 124.0757, found 124.0755. The data match those reported in the literature.[52]

Data for **65b**: ^1H NMR (400 MHz, $CDCl_3$) δ 10.06 (s, 1H, -C*H*O), 8.84 (d, *J* = 1.7 Hz, 2H, 1H, 6-H_{Ar}), 8.64 (d, *J* = 2.3 Hz, 1H, 2-H_{Ar}), 7.95 (dd, *J* = 2.3, 1.7 Hz, 1H, 4-H_{Ar}), 2.42 (s, 3H, Py-C*H*$_3$). ^{13}C NMR (101 MHz, $CDCl_3$) δ 191.1 (-*C*HO), 155.4 (2-C_{Ar}), 149.6 (6-C_{Ar}), 134.2, 131.2, 135.9 (4-C_{Ar}), 18.4 (Py-*C*H$_3$). HRMS (ESI, m/z) calculated for C_7H_7NO [M+H$^+$] 122.0600, found 122.0601. The data match those reported in the literature.[53]

1-(6-Methoxypyridin-3-yl)-ethan-1-one **67b**, *(+)-1-(6-Methoxypyridin-3-yl)-ethanol* (*R*)-**67a**

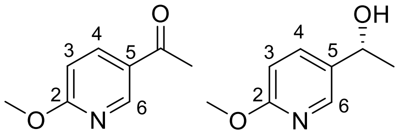

67b (*R*)-**67a**

Lyophilized r*Aae*UPO expression supernatant (100 mg, 90.0U) was dissolved in pH = 7.0 KPi buffer (100 mM, 18.7 mL) and 2-methoxy-5-ethylpyridine **66** (30.0 mg, 0.219 mmol, 1.00 eq.) was added to the reaction media in MeCN (2.19 mL). 30% aqueous H_2O_2 solution (22.3 µL, 7.44 mg, 0.219 mmol, 1.00 eq.) was diluted in deionised H_2O (1.0 mL) and the solution was added to the reaction dropwise over 13 h at rt. The aqueous phase was basified with 1.0 M NaOH solution (to pH = 10) and extracted with EtOAc (3 x 30 mL). The combined organic phase was washed with brine (20 mL) dried over anhydrous $MgSO_4$, filtered and evaporated *in vacuo* to yield the crude material as yellow, amorphous material. The crude (31 mg) was purified by column chromatography (eluent: *n*-Hex:EtOAc = 7:3) to yield two fractions. The major fraction contained ketone **67b** (R_f = 0.62 (eluent: *n*-Hex:EtOAc = 7:3), yield: 16.0 mg (49%)), whilst the minor fraction contained the alcohol (*R*)-**67a** (R_f = 0.24 (eluent: *n*-Hex:EtOAc = 7:3), yield: 12.3 mg (36%)).

Data for **67b**: ^1H NMR (400 MHz, $CDCl_3$) δ 8.78 (d, *J* = 2.4 Hz, 1H, 6-H_{Ar}), 8.14 (dd, *J* = 8.7, 2.4 Hz, 1H, 4-H_{Ar}), 6.79 (d, *J* = 8.7 Hz, 1H, 3-H_{Ar}), 4.01 (s, 3H, -O-C*H*$_3$), 2.57 (s, 3H, -CO-C*H*$_3$). ^{13}C NMR (101 MHz, $CDCl_3$) δ 195.9 (-*C*O-), 167.0 (2-C_{Ar}), 149.6 (6-C_{Ar}), 138.3 (4-C_{Ar}), 127.2 (5-C_{Ar}), 111.3 (3-C_{Ar}), 54.2 (-O-*C*H$_3$), 26.5 (-CO-*C*H$_3$). HRMS (APCI, m/z) m/z calculated for $C_8H_9NO_2$ [M+H$^+$] 152.0706, found 152.0700. The data match those published in the literature.[19]

Data for (*R*)-**67a**: *ee* = >99%, measured by chiral HPLC (Chiralcel(R) OJ-H), see ESI section 13 for details and chromatograms. $[\alpha]_D^{20}$ = 35.8 ($CHCl_3$, c = 0.82, n = 3).

^1H NMR (400 MHz, $CDCl_3$) δ 8.11 (d, *J* = 2.5 Hz, 1H, 6-H_{Ar}), 7.63 (dd, *J* = 8.5, 2.5 Hz, 1H, 4-H_{Ar}), 6.74 (d, *J* = 8.5 Hz, 1H, 3-H_{Ar}), 4.88 (q, *J* = 6.4 Hz, 1H, -C*H*(OH)-), 3.92 (s, 3H, -O-C*H*$_3$), 1.49 (d, *J* = 6.4 Hz, 3H, -CH(OH)-C*H*$_3$). ^{13}C NMR (101 MHz, $CDCl_3$) δ 163.9 (2-C_{Ar}), 144.2 (6-C_{Ar}), 136.5 (4-C_{Ar}), 133.8 (5-C_{Ar}), 111.1 (3-C_{Ar}), 68.0 (-*C*H(OH)-), 53.62 (-O-*C*H$_3$), 25.0 (-CH(OH)-*C*H$_3$). HRMS (APCI, m/z) m/z calculated for $C_8H_{11}NO_2$ [M+H$^+$] 154.0863, found 154.0863. The data match those published in the literature.[20]

*(+)-1-(6-Chloropyridin-3-yl)-ethanol (+)-***72**, **S18**

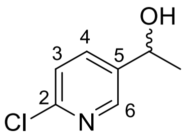

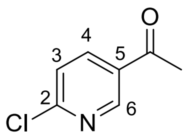

(+)-**72** **S20**

2-Chloro-5-ethylpyridine **68** (30 mg, 2.12 mmol, 1.00 eq.) was dissolved in MeCN (1.99 mL) and the solution was added to the solution of lyophilised r*Aae*UPO expression supernatant (90.9 mg, 81.8 U) in pH = 7.0 KPi buffer (100 mM, 16.9 mL). 30% H_2O_2 solution (20.3 µL, 7.51 mg, 2.20 mmol, 1.05 eq.) was diluted in deionised water (1.0 mL) and the solution was added to the reaction dropwise over 13 h at rt. The reaction media was extracted with EtOAc (3 x 20 mL) and the unified organic phase was washed with brine (10 mL), dried over anhydrous $MgSO_4$, filtered, and evaporated *in vacuo*. The crude material (yellow oil, 29 mg) was purified by column chromatography (eluent: *n*-Hex:EtOAc = 7:3) and pure (*R*)-**72** (pale yellow oil, R_f = 0.21 (eluent: *n*-Hex:EtOAc = 7:3), yield: 23 mg (73%)) and **S18** (brown oil, R_f = 0.59 (eluent: *n*-Hex:EtOAc = 7:3), yield: 2.0 mg (6.5%)) were yielded as products.

Data for (*R*)-**72**: $[\alpha]_{\mathrm{D}}^{20}$ = 28.7 ($CHCl_3$, c = 2.35, n = 3).

^{1}H NMR (400 MHz, $CDCl_3$) δ 8.33 (d, *J* = 2.7 Hz, 1H, 6-H_{Ar}), 7.70 (dd, *J* = 8.5, 2.7 Hz, 1H, 4-H_{Ar}), 7.30 (d, *J* = 8.5 Hz, 1H, 3-H_{Ar}), 4.94 (q, *J* = 6.7 Hz, 1H, -C*H*(OH)-), 1.50 (d, *J* = 6.7 Hz, 3H, -C*H*$_3$). ^{13}C NMR (101 MHz, $CDCl_3$) δ 150.4 (2-C_{Ar}), 147.3 (6-C_{Ar}), 140.2 (5-C_{Ar}), 136.4 (4-C_{Ar}), 124.3 (3-C_{Ar}), 67.5 (-C*H*(OH)-), 25.4 (-C*H*$_3$). HRMS (ESI, m/z) m/z calculated for C_7H_8ClNO [M+H$^+$] 158.0367, found 158.0369. IR (film): ν_{max}/cm^{-1} 3328 (O-H), 2974 (C-H_{Ar}), 2927 (C-H_{Ar}), 1458 (C-N), 1101 (C-O), 699, 796, 741. The data match those published in the literature.[54]

For **S18**: ^{1}H NMR (400 MHz, $CDCl_3$) δ 8.94 (d, *J* = 2.5 Hz, 1H, 6-H_{Ar}), 8.20 (dd, *J* = 8.3, 2.5 Hz, 1H, 4-H_{Ar}), 7.45 (d, *J* = 8.3 Hz, 1H, 3-H_{Ar}), 2.63 (s, 3H, -CO-C*H*$_3$). HRMS (ESI, m/z) m/z calculated for C_7H_6ClNO [M+H$^+$] 156.0211, found 156.0215. IR (film): ν_{max}/cm^{-1} 2923 (C-H_{Ar}), 2853 (C-H_{Ar}), 1735 (C=O), 1458 (C-N), 1261, 1091, 1019, 799. The data match those published in the literature.[30]

(+)-1-(6-Cyanopyridin-3-yl)-ethanol (*R*)-**73**

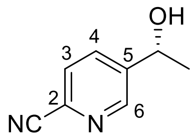

2-Cyano-5-ethylpyridine **69** (30.0 mg, 0.227 mmol, 1.00 eq.) was dissolved in MeCN (2.27 mL) and added to the solution of lyophilised r*Aae*UPO expression supernatant (103 mg, 92.7 U) in pH = 7.0 KPi buffer (100 mM, 19.4 mL). 30% H_2O_2 (23.2 µL, 7.70 mg, 0.227 mmol, 1.00 eq.) was diluted in deionised H_2O (1.0 mL) and added to the reaction dropwise over 5 h at rt. The reaction media was extracted with EtOAc (3 x 30 mL), the unified organic phase was washed with brine (10 mL), dried over anhydrous $MgSO_4$, filtered, and evaporated *in vacuo*. The crude material is purple oil. The crude material (40 mg) was purified by column chromatography (eluent: *n*-Hex:EtOAc = 7:3) to yield **73** as transparent oil. R_f = 0.36 (eluent: *n*-Hex:EtOAc = 7:3). Yield: 28.0 mg (84%). *ee* = >99%, measured by chiral HPLC (Daicel

Chiralpak(R) AD-H), see ESI **Section 13** for details and chromatograms. $[\alpha]_D^{20}$ = 38.2 ($CHCl_3$, c = 0.85, n = 3).

^{1}H NMR (400 MHz, $CDCl_3$) δ 8.70 (d, *J* = 2.1 Hz, 1H, 6-H_{Ar}), 7.89 (dd, *J* = 8.0, 2.1 Hz, 1H, 4-H_{Ar}), 7.68 (d, *J* = 8.0 Hz, 1H, 3-H_{Ar}), 5.05 (q, *J* = 6.5 Hz, 1H, -C*H*(OH)-), 2.36 (broad s, 1H, -O*H*), 1.54 (d, *J* = 6.5 Hz, 3H, -C*H*$_3$). ^{13}C NMR (101 MHz, $CDCl_3$) δ 149.0 (6-C_{Ar}), 145.1 (2-C_{Ar}), 134.0 (4-C_{Ar}), 132.7 (5-C_{Ar}), 128.5 (3-C_{Ar}), 117.4 (-*C*N), 67.7 (-C*H*(OH)-), 25.6 (-C*H*$_3$). HRMS (ESI, m/z) m/z calculated for $C_8H_8N_2O$ [M+Na$^+$] 171.0529, 171.0529.

(+)-5-(1-Hydroxyethyl)pyridine-2-carboxamide (*R*)-**74**

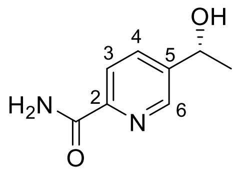

Lyophilized r*Aae*UPO expression supernatant was dissolved in pH = 7.0 KPi buffer (100 mM, 17.0 mL) and 5-ethylpicolinamide **70** (30.0 mg, 0.200 mmol, 1.00 eq.) was added in MeCN (2.0 mL). 30% H_2O_2 (20.4 µL, 6.80 mg, 0.200 mmol, 1.00 eq.) was diluted with deionised H_2O (1.0 mL) and added to the solution dropwise over 13 h at rt. The aqueous phase was extracted with EtOAc (3 x 20 mL), the unified organic phase was washed with brine (15 mL), dried over anhydrous $MgSO_4$, filtered, and evaporated under reduced pressure to yield the crude material as white solid. The crude was purified by column chromatography (eluent: *n*-Hex:EtOAc = 7:3). The title compound **74** was yielded as a white solid. R_f = 0.21 (eluent: EtOAc:*n*-Hex = 7:3). Yield: 18.0 mg (54%). *ee* = >99%, measured by chiral HPLC (Chiralcel(R) OJ-H column), see ESI **Section 13** for details and chromatograms. $[\alpha]_D^{20}$ = 34.5 (MeOH, c = 0.75, n = 3).

^{1}H NMR (400 MHz, $CDCl_3$) δ 8.61 (d, *J* = 2.2 Hz, 1H, 6-H_{Ar}), 8.05 (d, *J* = 8.2 Hz, 1H, 3-H_{Ar}), 7.91 (dd, *J* = 8.2, 2.2 Hz, 1H, 4-H_{Ar}), 4.94 (q, *J* = 6.3 Hz, 1H, -C*H*(OH)-), 1.46 (d, *J* = 6.3 Hz, 3H, -C*H*$_3$). ^{13}C NMR (101 MHz, $CDCl_3$) δ 169.3 (-*C*O-), 149.9 (2-C_{Ar}), 147.7 (6-C_{Ar}), 146.5 (5-C_{Ar}), 135.6 (4-C_{Ar}), 123.0 (3-C_{Ar}), 68.4 (-*C*H(OH)-), 25.4 (-*C*H$_3$). HRMS (ESI, m/z) m/z calculated for $C_8H_{10}N_2O_2$ [M+Na$^+$] 189.0634, found 189.0637. IR (film): ν_{max}/cm^{-1} 3369 (-OH), 2926 (-CH_{Ar}), 1677 (-C=O), 1301, 1160. 1098.

(+)-1-(6-Methylsulfinylpyridin-3-yl)-ethanol (*R*)-**75**

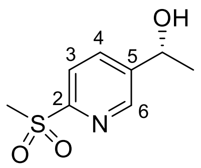

Lyophilized r*Aae*UPO expression supernatant (74 mg, 66U) was dissolved in pH = 7.0 KPi buffer (100 mM, 13.6 mL) and 5-ethyl-2-(methanesulfonyl)-pyridine **71** (30.0 mg, 0.162 mmol, 1.00 eq.) was added to the reaction media in MeCN (1.62 mL). 30% aqueous H_2O_2 solution (16.5 µL, 5.51 mg, 0.162 mmol, 1.00 eq.) was diluted in deionised H_2O (1.0 mL) and the solution was added to the reaction dropwise over 13 h at rt. The aqueous phase was basified with 1.0 M NaOH solution and extracted with EtOAc (3 x 30 mL). To aid phase separation, the mixture was filtered through a celite pad. The combined organic phase was dried over

anhydrous $MgSO_4$, filtered and evaporated *in vacuo* to yield the crude material as brown oil. The crude (36 mg) was purified by column chromatography (eluent: *n*-Hex:EtOAc = 7:3) to yield the product **75** as yellow oil. R_f = 0.22 (eluent: *n*-Hex:EtOAc = 7:3). Yield: 23 mg (71%). *ee* = 92%, measured by chiral HPLC (Daicel Chiralpak(R) AD-H column), see ESI **Section 13** for details and chromatograms. $[\alpha]_D^{20}$ = 34.5 (MeOH, c = 0.75, n = 3).

^{1}H NMR (400 MHz, MeOH-d$_4$) δ 8.69 (d, *J* = 2.1 Hz, 1H, 6-H_{Ar}), 8.02 (d, *J* = 8.3 Hz, 1H, 3-H_{Ar}), 7.97 (dd, *J* = 8.3, 2.1 Hz, 1H, 4-H_{Ar}), 5.07 (td, *J* = 6.5, 3.4 Hz, 1H, -C*H*(OH)-), 3.21 (s, 3H, -SO_2-C*H*$_3$), 2.41 (d, *J* = 3.4 Hz, 1H, -O*H*), 1.55 (d, *J* = 6.8 Hz, 3H, -CH(OH)-C*H*$_3$). ^{13}C NMR (101 MHz, $CDCl_3$) δ 156.8 (2-C_{Ar}), 148.0 (6-C_{Ar}), 145.8 (5-C_{Ar}), 135.3 (4-C_{Ar}), 121.2 (3-C_{Ar}), 67.7 (-*C*H(OH)-), 40.4 (-SO_2-*C*H$_3$), 25.6 (-CH(OH)-*C*H$_3$). HRMS (ESI, m/z) m/z calculated for $C_8H_{11}NO_3S$ [M+H$^+$] 202.0532, found 202.0536.

(-)-*5,6,7,8-Tetrahydroquinolin-5-ol* (*R*)-**4**, *5,6,7,8-Tetrahydroquinolin-6-ol* **76** - *In Situ Peroxide Generation*

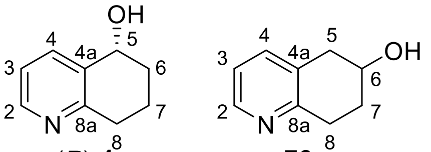

(*R*)-**4** **76**

Lyophilized r*Aae*UPO expression supernatant (364 mg, 352 U) was dissolved in a round bottomed flask in pH = 7.0 KPi buffer (100 mM, 100 mL) and the solution was diluted with deionised (72 mL) water. *Pp*AOx stock solution (58 µL in sucrose, 200 U) was diluted with pH = 7.0 KPi buffer (100 mM, 5 mL) and the solution was added to the reaction (1 U mL^{-1} final concentration). After this, 5,6,7,8-tetrahydroquinoline **3** (530 mg, 4.00 mmol, 1.00 eq) was added in MeCN (20 mL). The reaction was initiated by the addition of MeOH (3.2 mL, 400 mM final concentration) to the solution, which was left to stir for 24 h. The reaction medium was extracted with EtOAc (3 x 200 mL). The unified organic phase was dried over anhydrous $MgSO_4$, filtered, and evaporated *in vacuo*. The crude material was purified by column chromatography (eluent: EtOAc:MeOH=8:2) to yield two fractions. The major fraction (dense brown oil, R_f = 0.49 (eluent: EtOAc:acetone=7:3)) contained pure (*R*)-**4** (167 mg, 28%), the minor fraction (5 mg, brown oil, R_f = 0.38 (eluent: EtOAc:MeOH=8:2)) contained an 8:2 ratio of isomers **76** and **77**, which represents a 2% of **76** and 0.4% yield of **77**, calculated according to ^{1}H NMR ratio.

Data for (*R*)-**4**: *ee* = >99%, measured by chiral HPLC (Daicel Chiralpak(R) AD-H column), see ESI **Section 13** for details and chromatograms. $[\alpha]_D^{22}$ = -31.2 (MeOH, c = 2.81, n = 3).

^{1}H NMR (400 MHz, $CDCl_3$) δ 8.39 (dd, *J* = 4.8, 1.8 Hz, 1H, 2-H_{Ar}), 7.77 (dd, *J* = 7.5, 1.8 Hz, 1H, 4-H_{Ar}), 7.13 (dd, *J* = 7.5, 4.8 Hz, 1H, 3-H_{Ar}), 4.83 - 4.77 (m, 1H, 5-H), 3.02 - 2.82 (m, 2H, 8-H_α, 8-H_β), 2.56 (s, 1H, -O*H*), 2.11 - 1.97 (m, 2H, 6-H_β, 7-H_β), 1.92 - 1.78 (m, 2H, 6-H_α, 7-H_α). ^{13}C NMR (101 MHz, $CDCl_3$) δ 157.2 (8a-C_{Ar}), 148.6 (2-C_{Ar}), 136.5 (4a-C_{Ar}), 134.6 (4-C_{Ar}), 121.6 (3-C_{Ar}), 68.0 (5-C), 32.3 (6-C), 32.3 (8-C), 18.9 (7-C). HRMS (ESI, m/z) m/z calculated for $C_9H_{11}NO$ [M+H$^+$] 150.0919, found 150.0912. The data match those reported in the literature.[55]

Data for **76**: ^{1}H NMR (400 MHz, $CDCl_3$) δ 8.36 (d, *J* = 4.8 Hz, 1H, 2-H_{Ar}), 7.37 (d, *J* = 7.5 Hz, 1H, 4-H_{Ar}), 7.05 (dd, *J* = 7.5, 4.8 Hz, 1H, 3-H_{Ar}), 4.26-4.18 (m, 1H, 6-H), 3.32 – 2.88 (m, 3H, 5-H_α, 8-H_α, 8-H_β), 2.81 (dd, *J* = 16.4, 7.3 Hz, 1H, 5-H_β), 2.15 – 1.85 (m, 2H, 7-H_α, 7-H_β). ^{13}C NMR (101 MHz, $CDCl_3$) δ 156.1 (8a-C_{Ar}), 147.2 (2-C_{Ar}), 137.7 (4-C_{Ar}), 121.4 (3-C_{Ar}), 66.4 (6-

C), 37.5 (5-C), 31.2 (8-C), 29.8 (7-C). HRMS (ESI, m/z) m/z calculated for $C_9H_{11}NO$ [M+H$^+$] 150.0919, found 150.0912. The data match those reported in the literature.[56]

Data for **77**: see next page *(slow H_2O_2 addition method)*

(-)-*5,6,7,8-Tetrahydroquinolin-5-ol* (*R*)-**4**, *5,6,7,8-Tetrahydroquinolin-7-ol* **77** *- Slow H_2O_2 Addition*

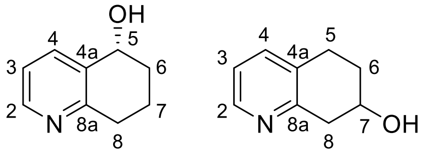

(*R*)-**4** **77**

Lyophilized r*Aae*UPO expression supernatant (273 mg, 264 U) was dissolved in pH = 7.0 KPi buffer (100 mM, 100 mL) and the solution was diluted with deionised water (40 mL). 5,6,7,8-tetrahydroquinoline **30** (266 mg, 2.00 mmol, 1.00 eq.) was added in MeCN (20 mL). 30% aqueous H_2O_2 solution (408 µL, 136 mg, 4.00 mmol, 2.00 eq.) was diluted in deionised H_2O (40 mL) and the solution was added to the mixture dropwise over 25 h at rt. To aid phase separation and extraction, the reaction medium was saturated with NaCl and it was extracted with EtOAc (3 x 200 mL). The combined organic phase was dried over anhydrous $MgSO_4$, filtered, and evaporated *in vacuo*. The crude material was separated by column chromatography (eluent: EtOAc/acetone = 7:3 to EtOAc/MeOH = 8:2) to yield two fractions. The major fraction (dense, brown oil, R_f = 0.49 (eluent: EtOAc/acetone = 7:3)) contained pure (*R*)-**4** (244 mg, 82%), the minor fraction (brown oil, R_f = 0.22 (eluent: EtOAc/acetone = 7:3)) contained 7:3 ratio of isomers **77** and **76**, which represents a 13% of **77** and 5% yield of **76**, calculated according to ^{1}H NMR ratio.

Data for (*R*)-**4**: The data is identical to the one described above.

Data for **77**:^{1}H NMR (400 MHz, $CDCl_3$) δ 8.37 (d, *J* = 4.9 Hz, 1H, 2-H_{Ar}), 7.40 (d, *J* = 7.6 Hz, 1H, 4-H_{Ar}), 7.06 (dd, *J* = 7.6, 4.9 Hz, 1H, 3-H_{Ar}), 4.34 - 4.27 (m, 1H, 7-H), 3.26 (dd, *J* = 17.3, 4.9 Hz, 1H, 8-H_α), 3.18 - 2.90 (m, 2H, 8-H_β, 6-H_α), 2.84 - 2.73 (m, 1H, 5-H_α), 2.15 - 1.78 (m, 2H, 5-H_β, 6-H_β). ^{13}C NMR (101 MHz, $CDCl_3$) δ 154.8 (8a-C_{Ar}), 147.3 (2-C_{Ar}), 137.6 (4a-C_{Ar}), 136.6 (4-C_{Ar}), 121.5 (3-C_{Ar}), 66.8 (7-C), 41.5 (8-C), 30.6 (6-C), 25.4 (5-C). HRMS (ESI, m/z) m/z calculated for $C_9H_{11}NO$ [M+H$^+$] 150.0919, found 150.0912.

Tetrahydroisoquinolin-5-ol **37a**, *5,6,7,8-Tetrahydroisoquinolin-7-ol* **37b**, *5,6,7,8-Tetrahydroisoquinolin-8-ol* **37c**, *5,6,7,8-Tetrahydroisoquinolin-8-ol* **37c** and *5,6,7,8- 5,6,7,8-Tetrahydroisoquinolin-6-ol* **37d**

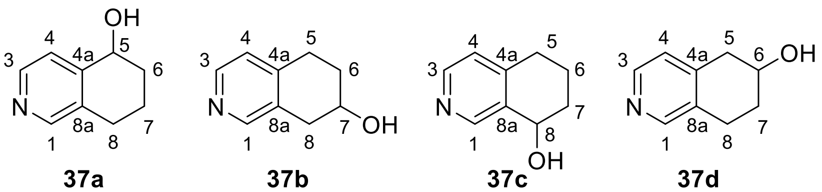

37a **37b** **37c** **37d**

Lyophilized *rAae*UPO expression supernatant (682 mg, 660 U) was dissolved in pH = 7.0 KPi buffer (100 mM, 75 mL) and the solution was diluted with deionised H_2O (42 mL). 5,6,7,8-tetrahydroisoquinoline **30** (200 mg, 1.50 mmol, 1.00 eq.) was added in MeCN (15 mL). 30% aqeous H_2O_2 solution (153 µL, 51.0 mg, 1.50 mmol, 1.00 eq.) was diluted in deionised H_2O

(9 mL) and the solution was added to the mixture dropwise over 25 h at rt. To aid phase separation and extraction, the reaction was saturated with NaCl. The reaction media was then extracted with EtOAc (3 x 150 mL) and *n*-BuOH (1 x 150 mL). The unified organic phase was dried over anhydrous $MgSO_4$, filtered, and evaporated *in vacuo*. The crude material was separated by two rounds of column chromatography (eluent: $CHCl_3$/TEA = 20:1 (1 % MeOH), followed by $CHCl_3$/TEA = 9:1). Two major product fractions were obtained. The major fraction (90 mg, deep red oil, R_f = 0.11 (eluent: $CHCl_3$:MeOH = 9:1)) contained an 10:7 ratio of isomers **37c** and **37b**, which represents a 22% yield of **37c** and 15% yield of **37b**, calculated according to their ^{1}H NMR ratio. Another fraction (26 mg, brown oil, R_f = 0.20 (eluent: $CHCl_3$/TEA = 9:1)) was also obtained containing a 7:3 ratio of isomers **37a** and **37d**, which represents a 12% of **37a** and 5% yield of **37d**, calculated according to ^{1}H NMR ratio.

Data for **37a:** ^{1}H NMR (400 MHz, MeOH-d_4) δ 8.05 (s, 1H, 1-H_{Ar}), 8.00 (dd, *J* = 6.6, 1.9 Hz, 1H, 3-H_{Ar}), 7.23 (d, *J* = 6.6 Hz, 1H, 4-H_{Ar}), 4.64 (t, *J* = 6.0 Hz, 1H, 5-H) 2.83-2.65 (m, 2H, 8-H_α, 8-H_β), 2.13 – 1.92 (m, 1H, 6-H_α), 1.85 – 1.69 (m, 3H, 6-H_β, 7-H_α, 7-H_β). ^{13}C NMR (101 MHz, MeOH-d_4) 150.2 (1-C_{Ar}), 147.0 (3-C_{Ar}), 143.4 (4a-C_{Ar}), 139.7 (8a-C_{Ar}), 128.1 (4-C_{Ar}), 67.9 (5-H), 32.8, 29.0, 27.2, 26.9, 23.0, 22.7, 20.4 HRMS (ESI, m/z) m/z calculated for $C_7H_{11}NO$ [M+H$^+$] 150.0919, found 150.0913 (mixture of **37a** and **37d**).

Data for **37b:** ^{1}H NMR (400 MHz, MeOH-d_4) δ 8.17 (s, 1H, 1-H_{Ar}), 8.13 (d, *J* = 5.0 Hz, 1H, 3-H_{Ar}), 7.09 (d, *J* = 5.0 Hz, 1H, 4-H_{Ar}), 4.11-4.01 (m, 1H, 7-H), 3.15 – 2.88 (m, 1H, 8-H_α), 2.85 – 2.59 (m, 4H, 5-H', 5-H_β, 8-H_β), 2.02-1.89 (m, 1H, 6-H_α), 1.88 – 1.62 (m, 1H, 6-H_β). ^{13}C NMR (101 MHz, MeOH-d_4) 150.7 (1-C_{Ar}), 148.1 (4a-C_{Ar}), 146.7 (3-C_{Ar}), 133.0 (8a-C_{Ar}), 125.1 (4-C_{Ar}), 66.5 (-*C*H-OH), 35.6 (Ar-*C*'H_2-), 31.0 (Ar-C''H_2-), 27.0 (-CH(OH)-*C*H_2-CH_2-).

Data for **37c:** ^{1}H NMR (400 MHz, MeOH-d_4) δ 8.48 (s, 1H, 1-H_{Ar}), 8.20 (d, *J* = 5.6 Hz, 1H, 3-H_{Ar}), 7.10 (d, *J* = 5.6 Hz, 1H, 4-H_{Ar}), 4.73 (t, *J* = 4.9 Hz, 1H, 8-H), 3.05 – 2.88 (m, 1H, 5-H'), 2.85 – 2.59 (m, 1H, 5-H''), 2.01-1.88 (m, 2H, 6-H', 7-H'), 1.88 – 1.62 (m, 2H, 6-H'', 7-H''). ^{13}C NMR (101 MHz, $CDCl_3$) δ 150.7 (1-C_{Ar}), 149.1 (4a-C_{Ar}), 147.8 (3-C_{Ar}), 137.2 (8a-C_{Ar}), 125.3 (4-C_{Ar}), 66.3 (8-C), 32.8 (7-C), 29.5 (5-C), 19.3 (6-C). HRMS (ESI, m/z) m/z calculated for $C_9H_{11}NO$ [M+H$^+$] 150.0919, found 150.0914 (mixture of **37a** and **37b**).

Data for **37d**: ^{1}H NMR (400 MHz, MeOH-d_4) δ 7.46 (s, 1H, 1-H_{Ar}), 7.46 (d, *J* = 5.1 Hz, 1H, 3-H_{Ar}), 7.13 (d, *J* = 5.1 Hz, 1H, 4-H_{Ar}), 4.12-4.05 (m, 1H, -C*H*(OH)-), 3.06-2.90 (m, 2H, Ar-CH_2-CH(OH)-), 2.80-2.60 (m, 2H, Ar-CH_2-), 2.10-1.94 (m, 1H, Ar-CH_2-*H*CH-), 1.83-1.73 (m, 1H, Ar-CH_2-HC*H*-).^{13}C NMR (101 MHz, MeOH-d_4) 151.0 (1-C_{Ar}), 138.5, 137.1, 124.1, 66.3 (6-C), 32.8, 29.0, 27.2, 26.9, 23.0, 22.7, 20.4.

5,6-Epoxy-5,6-dihydroquinoline **41**, *6,7-Epoxy-5,6-trans-dihydroxy-5,6,7,8-tetrahydroquinoline* **43**

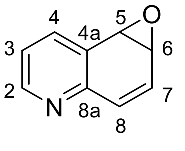

41

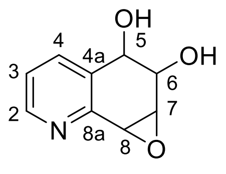

43

Lyophilized r*Aae*UPO expression supernatant (159 mg, 131 U) was dissolved in pH = 7.0 KPi buffer (100 mM, 15 mL) and the mixture was diluted with deionised water (6.0 mL). Quinoline **33** (35.5 µL, 38.7 mg, 300 µmol, 1.00 eq.) was added to the reaction in MeCN (3.0 mL). 30%

H_2O_2 (30.6 µL, 10.2 mg, 300 µmol, 1.00 eq.) was diluted with deionised H_2O (6.0 mL) and the solution was added to the reaction medium over 25 h at rt. The aqueous phase was saturated with NaCl and extracted with EtOAc (4 x 40 mL). The crude material (51 mg) was purified by column chromatography (eluent: DCM:MeOH = 20:1). Two fractions were collected. The major fraction contained pure epoxide **41** (brown oil, R_f = 0.56 (eluent: DCM:MeOH = 20:1), yield: 26 mg (60%)) and the minor fraction contained pure **43** (red solid, R_f = 0.29 (eluent: DCM:MeOH = 20:1), yield: 11 mg (21%)).

Data for **41**: ^{1}H NMR (400 MHz, $CDCl_3$) δ 8.63 (d, *J* = 4.8 Hz, 1H, 2-H_{Ar}), 7.92 (d, *J* = 8.0 Hz, 1H, 4-H_{Ar}), 7.28-7.22 (m, 1H, 3-H_{Ar}), 6.98 (dd, *J* = 9.9 Hz, 1.5 Hz, 1H, 8-H), 6.74 (dd, *J* = 9.9, 3.8 Hz, 1H, 7-H), 4.51 (d, *J* = 3.8 Hz, 1H, 5-H), 4.17 (td, *J* = 3.8, 1.5 Hz, 1H, 6-H). HRMS (ESI, m/z) m/z calculated for C_9H_7NO [M+H$^+$] 146.0606, found 146.0597. The spectroscopic data match those previously reported.[57]

Data for **43**: ^{1}H NMR (400 MHz, MeOH-d$_4$) δ 8.47 (dd, *J* = 5.2, 1.5 Hz, 1H, 2-H_{Ar}), 7.80 (dd, *J* = 7.7, 1.5 Hz, 1H, 4-H_{Ar}), 7.44 (dd, *J* = 7.7, 5.2 Hz, 1H, 3-H_{Ar}), 4.55 (dd, *J* = 2.9, 2.0 Hz, 1H, 6-H), 4.37 (t, *J* = 2.9 Hz, 1H, 5-H), 4.07 (d, *J* = 3.8 Hz, 1H, 8-H), 3.88 (dt, *J* = 3.8, 2.1 Hz, 1H, 7-H). ^{13}C NMR (101 MHz, MeOH-d$_4$) δ 153.9 (8a-C_{Ar}), 149.7 (2-C_{Ar}), 140.9 (4-C_{Ar}), 133.5 (5a-C_{Ar}), 125.8 (3-C_{Ar}), 73.1 (8-C), 68.2 (7-C), 58.7 (6/7-C), 53.7 (6/7-C). HRMS (ESI, m/z) m/z calculated for $C_9H_7NO_3$ [M+H$^+$] 180.0661, found 180.0652.

Isoquinoline-2-oxide **44**, *7,8-Epoxy-7,8-dihydroisoquinoline* **78**

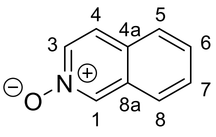

44

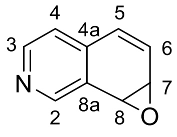

78

Lyophilized r*Aae*UPO expression supernatant (0.91 g, 881 U) was dissolved in pH = 7.0 KPi buffer (100 mM, 50 mL) in a 250 mL round bottom flask. The solution was diluted with deionised water (27.5 mL), followed by the addition of isoquinoline **33** (117 µL, 129 mg, 1.00 mM, 1.00 eq) in MeCN (10 mL). 30% H_2O_2 (102 µL, 34.0 mg, 1.00 mM, 1.00 eq.) solution was diluted with deionised water (12.5 mL) and the solution was added to the suspension dropwise over 25 h at rt. The aqueous phase was extracted with EtOAc (4 x 100 mL) and the unified organic phase was dried over $MgSO_4$, filtered, and evaporated *in vacuo*. The crude product was purified by column chromatography (DCM (3% MeOH)). Aside from the recovered starting material (70 mg, 0.54 mmol, 54%), 2 fractions were collected. The major fraction contained pure *N*-oxide **44** (red oil, R_f = 0.39 (DCM:MeOH = 20:1), yield: 50 mg (34%)), whilst the minor fraction contained epoxide **78** (brown oil, R_f = 0.51 (DCM (3 % MeOH), yield: 8 mg (5.5%)).

Data for **44**: ^{1}H NMR (400 MHz, MeOH-d$_4$) δ 9.00 (s, 1H, 1-H_{Ar}), 8.18 (dd, *J* = 6.8, 2.2 Hz, 1H, 3-H_{Ar}), 7.98 (dd, *J* = 7.0, 2.3 Hz, 1H, 3-H_{Ar}), 7.96 (dt, *J* = 7.2, 2.3 Hz, 2H, 5-H_{Ar}, 8-H_{Ar}), 7.74 (t, *J* = 7.2 Hz, 1H, 7-H_{Ar}), 7.73 (t, *J* = 7.2 Hz, 1H, 6-H_{Ar}). ^{13}C NMR (101 MHz, MeOH-d$_4$) 136.8, 136.5, 129.7, 129.6, 129.4, 129.2, 126.8, 125.3, 124.5. HRMS (ESI, m/z) m/z calculated for C_9H_7NO [M+H$^+$] 146.0606, found 146.0602. The data match those reported in the literature.[58]

Data for **78**: ^{1}H NMR (400 MHz, $CDCl_3$) δ 8.83 (s, 1H, 1-H_{Ar}), 8.61 (d, *J* = 5.2 Hz, 1H, 3-H_{Ar}), 7.18 (d, *J* = 5.2 Hz, 1H, 4-H_{Ar}), 6.74 (dd, *J* = 10.1, 1.7 Hz, 1H, 5-H), 6.70 (dd, *J* = 10.1, 3.8 Hz,

1H, 6-H), 4.53 (d, J = 3.8 Hz, 1H, 8-H), 4.15 (td, J = 3.8, 1.7 Hz, 1H). HRMS (ESI, m/z) m/z calculated for C_9H_7NO [M+H$^+$] 146.0606, found 146.0602.The data match those reported in the literature.[59]

3-Methylisoquinoline-N-oxide **46**, *Isoquinoline-3-carbaldehyde* **47**

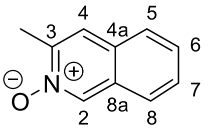

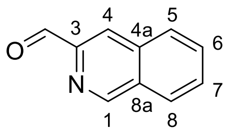

46 **47**

Lyophilized r*Aae*UPO expression supernatant (136 mg, 132 U) was dissolved in pH = 7.0 KPi buffer (100 mM, 15 mL). The solution was diluted with deionised water (6.0 mL), followed by the addition of 3-methylisoquinoline (43 mg, 0.30 mM, 1.00 eq) in MeCN (3.0 mL). 30% H_2O_2 (34.0 µL, 11.3 mg, 0.30 mM, 1.00 eq.) solution was diluted with deionised water (6.0 mL) and the solution was added to the suspension dropwise over 25 h at rt. The aqueous phase was extracted with EtOAc (4 x 40 mL) and the unified organic phase was dried over $MgSO_4$, filtered, and evaporated *in vacuo*. The crude product was purified by column chromatography (EtOAc:MeOH = 9:1). Two fractions were collected: the major fraction contained pure *N*-oxide **46** (pale brown solid, R_f = 0.20 (eluent: EtOAc:MeOH = 9:1), yield: 28 mg (59%)) and the minor fraction contained pure aldehyde **47** (yellow oil, R_f = 0.91 (eluent: EtOAc:MeOH = 9:1), yield: 5 mg (12%)).

Data for **46**: ^{1}H NMR (400 MHz, $CDCl_3$) δ 8.84 (s, 1H, 1-H_{Ar}), 7.72 - 7.66 (m, 2H, 2 x H_{Ar}), 7.63 (s, 1H, 4-H_{Ar}), 7.55 - 7.50 (m, 2H, 2 x H_{Ar}), 2.63 (s, 3H, -CH_3). ^{13}C NMR (101 MHz, $CDCl_3$) δ 146.0 (3-C_{Ar}), 136.5 (1-C_{Ar}), 129.3 (4a-C_{Ar}), 128.9 (5-C_{Ar}), 128.6 (8a-C_{Ar}), 128.4 (8-C_{Ar}), 125.6 (6/7-C_{Ar}), 124.6 (6/7-C_{Ar}), 123.2 (4-C_{Ar}), 17.8 (-CH_3). HRMS (ESI, m/z) m/z calculated for $C_{10}H_9NO$ [M+H$^+$] 160.0757, found 160.0753. The data match those reported in the literature.[60]

Data for **47**: ^{1}H NMR (400 MHz, $CDCl_3$) δ 10.28 (s, 1H, -C*H*O), 9.39 (s, 1H, 1-H_{Ar}), 8.41 (s, 1H, 4-H_{Ar}), 8.09 (dd, J = 7.0, 2.0 Hz, 1H, 5/8-H_{Ar}), 8.05 (dd, J = 7.3, 1.7 Hz, 1H, 5/8-H_{Ar}), 7.85 - 7.78 (m, 2H, 6-H_{Ar}, 7-H_{Ar}). ^{13}C NMR (101 MHz, $CDCl_3$) δ 193.5 (-*C*HO), 153.4 (1-C_{Ar}), 131.6, 130.7, 130.3, 128.0, 121.9 (4-C_{Ar}). HRMS (ESI, m/z) m/z calculated for $C_{10}H_7NO$ [M+H$^+$] 158.0600, found 158.0599. The data match those reported in the literature.[61]

(-)-2-Bromo-5,6,7,8-tetrahydroquinolin-5-ol **80a**, *2-Bromo-5,6,7,8-tetrahydroquinolin-6-ol* **80b**, *2-Bromo-5,6,7,8-tetrahydroquinolin-7-ol* **80c**

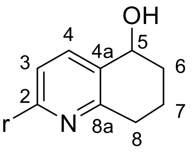

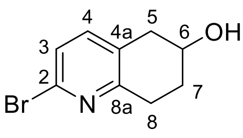

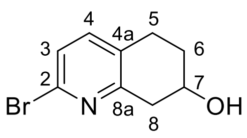

80a **80b** **80c**

2-Bromo-5,6,7,8-tetrahydroquinoline **79** (30.0 mg, 0.141 mmol, 1.00 eq.) was dissolved in MeCN (1.41 mL) and added to a solution of lyophilised r*Aae*UPO supernatant (128 mg, 115 U) in a mixture of pH = 7.0 KPi buffer (100 mM, 7.05 mL) and deionised H_2O (4.64 mL) resulting in a heterogenous mixture. 30% H_2O_2 (14.4 µL, 4.81 mg, 0.141 mmol, 1.00 eq.) was diluted in deionised H_2O (1.0 mL) and the solution was added to the reaction dropwise over 25 h at rt.

The resulting suspension was extracted with EtOAc (3 x 20 mL), the combined organic phase was washed with brine (10 mL), dried over $MgSO_4$, filtered, and evaporated *in vacuo* to yield the crude material as brown oil. The crude (28 mg) was separated by preparative thin-layer chromatography (eluent: *n*-Hex:EtOAc = 1:1) to yield two fractions. The major fraction contained pure 2-bromo-5,6,7,8-tetrahydroquinolin-5-ol **80a** (transparent oil, R_f = 0.50 (eluent: *n*-Hex:EtOAc = 1:1), yield: 17 mg (53%)), whilst the minor fraction (yellow oil, R_f = 0.30 (eluent: *n*-Hex:EtOAc = 1:1), 6.0 mg) contained a mixture of 2-bromo-5,6,7,8-tetrahydroquinolin-6-ol **80b** and 2-bromo-5,6,7,8-tetrahydroquinolin-7-ol **80c** in a 1:0.66 ratio (identity of the major isomer is unclear), which corresponds to 11% and 9% yields, for the two isomers.

Data for 5-ol **80a**: $[\alpha]_D^{22}$ = –43.6 ($CHCl_3$, c = 0.79, n = 3).

^{1}H NMR (400 MHz, MeOH-d_4) δ 7.70 (d, *J* = 8.1 Hz, 1H, 4-H_{Ar}), 7.38 (d, *J* = 8.1 Hz, 1H, 3-H_{Ar}), 4.68 (dd, *J* = 6.6, 4.0 Hz, 1H, 5-H), 2.91 - 2.74 (m, 2H, 8-H_α, 8-H_β), 2.0 - 1.95 (m, 2H, 6-H_α, 7-H_α), 1.85 - 1.71 (m, 2H, 6-H_β, 7-H_β). ^{13}C NMR (101 MHz, MeOH-d_4) δ 160.0 (8a-C_{Ar}), 141.0 (4-C_{Ar}), 140.7 (2-C_{Ar}), 136.0 (4a-C_{Ar}), 127.0 (3-C_{Ar}), 67.8 (5-H), 32.7 (6/8-C), 32.6 (6/8-C), 19.5 (7-C). HRMS (ESI, m/z) m/z calculated for $C_9H_{10}NOBr$ [M+H$^+$] 228.0024, found 228.0021. IR (film): v_{max}/cm^{-1} 3338 (O-H), 2943 (C-H_{Ar}), 1574 (C=N), 1436 (C=N), 1105 (C-O), 841.

Data for mixture of **80b** and **80c**: $[\alpha]_D^{22}$ = 14.3 ($CHCl_3$, c = 0.29, n = 3).

^{1}H NMR (400 MHz, MeOH-d_4) δ 7.42 - 7.35 (m, 1H, 4-$H_{Ar,}$ 4-H'$_{Ar}$), 7.30 (d, *J* = 7.7 Hz, 1H, 3-$H_{Ar,}$ 3-H'$_{Ar}$), 4.20 - 4.13 (m, 1H, 6/7-H), 4.13 - 4.05 (m, 1H, 6/7-H), 3.13 - 2.65 (m, 4H, 2 x 5-H, 2 x 5-H'$_{Ar}$, 2 x 8-H, 2 x 8-H'), 2.07 - 1.75 (m, 2H, 2 x 6/7-H, 2 x 6/7-H'). ^{13}C NMR (101 MHz, MeOH-d_4) δ 140.6 (4-C_{Ar}), 139.6 (4-C'$_{Ar}$), 131.4 (2-C_{Ar}), 129.8 (2-C'$_{Ar}$), 125.4 (3-C_{Ar}), 125.4 (3-C'$_{Ar}$) 122.6 (4a-C_{Ar}), 122.6 (4a-C'$_{Ar}$), 65.2 (6/7-C), 64.7 (6/7-C), 39.9, 35.85, 29.74, 29.43, 28.36, 23.98. HRMS (ESI, m/z) m/z calculated for $C_9H_{10}NOBr$ [M+H$^+$] 228.0024, found 228.0017. IR (film): v_{max}/cm^{-1} 3349 (O-H), 2933 (C-H_{Ar}), 1573 (C=N), 1440 (C=N), 1192 (C-O), 1095 (C-O), 833.

(-)-5-hydroxy-5,6,7,8-tetrahydroquinoline-2-carbonitrile **82a**, *6-Hydroxy-5,6,7,8-tetrahydroquinoline-2-carbonitrile* **82b**, *7-hydroxy-5,6,7,8-tetrahydroquinoline-2-carbonitrile* **82c**, *(+)-5,6-dihydroxy-5,6,7,8-tetrahydroquinoline-2-carbonitrile* **82d**

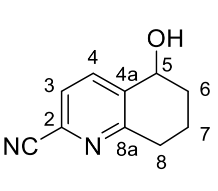

82a

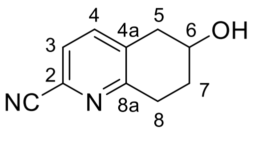

82b

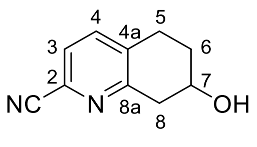

82c

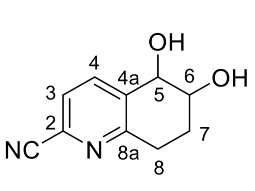

82d

Lyophilised r*Aae*UPO expression supernatant (190 mg, 171 U) was dissolved in a mixture of pH = 7.0 KPi buffer (100 mM, 10.5 mL) and deionised H_2O (7.4 mL). 2-Cyano-5-6-7-8-tetrahydroquinoline **81** (33.1 mg, 0.209 mmol, 1.00 eq.) was added to the mixture in MeCN (2.10 mL). 30% aqueous H_2O_2 (21.3 µL, 7.09 mg, 0.209 mmol, 1.00 eq.) was diluted with deionised H_2O (1.0 mL) and added to the reaction mixture dropwise over 25 h at rt. The pH of the reaction media was adjusted to 12 with the addition of 3.0 M NaOH solution and it was extracted with EtOAc (4 x 50 mL). The unified organic phase was washed with brine (15 mL), dried over anhydrous $MgSO_4$, filtered, and evaporated *in vacuo* to yield the crude material as

pale brown amorphous solid. The crude (32 mg) was purified by preparative thin-layer chromatography (eluent: *n*-Hex:EtOAc = 1:1) to yield three fractions. The major fraction (pale yellow amorphous material, R_f = 0.23 (eluent: *n*-Hex:EtOAc = 1:1), 17.5 mg), contained a mixture of β-alcohols 6-ol **82b** and 7-ol **82c** in 2:1 ratio (corresponding to 31% and 16% yield based on the ^{1}H NMR ratio). A second fraction contained pure 5-ol **82a** (yellow oil, R_f = 0.35 (eluent: *n*-Hex:EtOAc = 1:1), yield: 8.6 mg (24%)). The smallest fraction contained pure 5,6-diol **82d** (R_f = 0.58 (eluent: *n*-Hex:EtOAc = 1:1), yield: 2.5 mg (7%)).

Data for **82a**: $[\alpha]_{\mathrm{D}}^{21}$ = –63.2 ($CHCl_3$, c = 0.125, n = 3).

^{1}H NMR (400 MHz, MeOH-d_4) δ 8.02 (d, *J* = 8.2 Hz, 1H, 4-H_{Ar}), 7.69 (d, *J* = 7.7 Hz, 1H, 3-H_{Ar}), 4.77 (dd, *J* = 7.5, 4.4 Hz, 1H, 5-H), 3.00 – 2.84 (m, 2H, 8-$H_α$, 8-$H_β$), 2.13 - 2.04 (m, 2H, 6-$H_α$, 7-$H_α$), 1.91 - 1.75 (m, 2H, 6-$H_β$, 7-$H_β$). ^{13}C NMR (101 MHz, MeOH-d_4) δ 160.9 (8a-C_{Ar}), 141.4 (2-C_{Ar}), 138.7 (4-C_{Ar}), 132.6 (4a-C_{Ar}), 127.6 (3-C_{Ar}), 118.3 (-CN), 68.2 (5-C), 32.8 (8-C), 32.5 (6-C), 19.6 (7-C). HRMS (ESI, m/z) m/z calculated for $C_{10}H_{10}N_2O$ [M+H$^+$] 175.0866, found 175.0864. IR (film): v_{max}/cm^{-1} 3351 (O-H), 2945 (C-H), 2236 (-CN), 1570 (C=N), 1449 (C-N), 1421, 1066 (C-O), 1050, 998, 851.

Data for **82b**: $[\alpha]_{\mathrm{D}}^{24}$ = –5.3 (mixture of **82b** and **82c**, $CHCl_3$, c = 0.65, n = 3).

^{1}H NMR (400 MHz, MeOH-d_4) δ 7.66 (dd, *J* = 7.8 Hz, 1H, 4-H_{Ar}), 7.58 (d, *J* = 7.8 Hz, 1H, 3-H_{Ar}), 4.27 - 4.16 (m, 1H, 6-H), 3.14 (dd, *J* = 7.4, 4.4 Hz, 1H, 8-$H_α$), 3.10 (dd, *J* = 7.4, 4.4 Hz, 1H, 5-$H_α$), 2.90 - 2.80 (m, 2H, 5-$H_β$, 8-$H_β$), 2.12 - 1.94 (m, 1H,7-$H_α$) 1.87 (td, *J* = 14.4, 7.3 Hz, 1H, 7-$H_β$). ^{13}C NMR (101 MHz, MeOH-d_4) δ 159.1 (8a-C_{Ar}), 138.9 (4-C_{Ar}), 138.3 (2-C_{Ar}), 131.6 (4a-C_{Ar}), 127.4 (3-C_{Ar}), 118.4 (-CN), 64.9 (6-C), 41.5 (5-C), 30.4 (7-C), 26.0 (8-C). HRMS (ESI, m/z) m/z calculated for $C_{10}H_{10}N_2O$ [M+H$^+$] 175.0866, found 175.0866. IR (film): v_{max}/cm^{-1} 3359 (O-H), 2931 (C-H), 2236 (CN), 1570 (C=N), 1452 (C-N), 1054 (C-O), 841.

Data for **82c**: ^{1}H NMR (400 MHz, MeOH-d_4) δ 7.66 (d, *J* = 7.8 Hz, 1H, 4-H_{Ar}), 7.58 (d, *J* = 7.8 Hz, 1H, 3-H_{Ar}), 4.18 – 4.13 (m, 7-H), 3.10 - 3.00 (m, 2H, 5-$H_α$, 8-$H_α$), 2.95 - 2.80 (5-$H_β$, 8-$H_β$), 2.11 - 1.95 (m, 2H, 6-$H_α$, 6-$H_β$). ^{13}C NMR (101 MHz, MeOH-d_4) δ 160.3 (8a-C_{Ar}), 139.9 (4-C_{Ar}), 136.9 (2-C_{Ar}), 131.4 (4a-C_{Ar}), 127.3 (3-C_{Ar}), 118.4 (-CN), 65.7 (7-H), 38.0 (8-C), 30.9 (6-C), 29.7 (5-C).

Data for (+)-**82d**: $[\alpha]_{D}^{24}$ = 7.7 ($CHCl_3$, c = 0.08, n = 3).

^{1}H NMR (600 MHz, MeOH-d_4) δ 8.07 (d, *J* = 7.7 Hz, 1H, 4-H_{Ar}), 7.75 (dd, *J* = 7.7 Hz, 1H, 3-H_{Ar}), 4.02 (d, *J* = 4.0 Hz, 1H, 5-H), 3.84 (dd, *J* = 4.0, 2.7 Hz, 1H, 6-H), 2.87 - 2.80 (m, 2H, 8-$H_α$, 8-$H_β$), 2.57 - 2.51 (m, 1H, 7-$H_α$), 1.96 - 1.89 (m, 1H, 7-$H_β$). ^{13}C NMR (151 MHz, MeOH-d_4) δ 159.1 (8a-C_{Ar}), 138.0 (4-C_{Ar}), 133.5 (4a-C_{Ar}), 131.6 (2-C_{Ar}), 126.5 (3-C_{Ar}), 54.8 (6-C), 50.4 (5-C), 26.8 (8-C), 20.5 (7-C). HRMS (ESI, m/z) m/z calculated for $C_{10}H_8N_2O$ (loss of water) [M+H$^+$] 173.0709, found 173.0720. IR (film): v_{max}/cm^{-1} 3362 (O-H), 2925 (C-H), 2235 (-CN), 1574 (C=N), 1453 (C-N), 1063, 839.

(-)-2-(Methylsulfonyl)-5,6,7,8-tetrahydroquinolin-5-ol **84a**, *2-(Methylsulfonyl)-5,6,7,8-tetrahydroquinolin-6-ol* **84b**, *2-(Methylsulfonyl)-5,6,7,8-tetrahydroquinolin-7-ol* **84c**

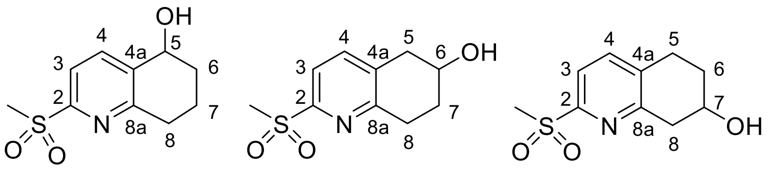

84a **84b** **84c**

2-(Methylsulfonyl)-5,6,7,8-tetrahydroquinolin **83** (50.0 mg, 0.237 mmol, 1.00 eq.) was dissolved in MeCN (2.37 mL) and added to a solution of lyophilised r*Aae*UPO expression supernatant (216 mg, 194 U) in a mixture of pH = 7.0 KPi buffer (100 mM, 11.9 mL) and deionised H_2O (8.48 mL). 30% aqueous H_2O_2 solution was diluted in deionised H_2O (1.0 mL) and the solution was added dropwise to the reaction mixture over 25 h at rt. The reaction media was extracted with EtOAc (4 x 20 mL), the combined organic phase was washed with brine (15 mL), dried over anhydrous $MgSO_4$, filtered, and evaporated under reduced pressure to yield the crude material as brown oil. The crude (61 mg) was purified by preparative thin-layer chromatography (eluent: *n*-Hex:EtOAc = 1:1) to yield two fractions containing productss, and a third containing recovered starting material (35%, yellow oil). The major product fraction (yellow oil, R_f = 0.18 (eluent: *n*-Hex:EtOAc = 1:1), yield: 23 mg (43%)) contained pure **84a**. The minor fraction (yellow oil, R_f = 0.07 (eluent: *n*-Hex:EtOAc = 1:1), 9.7 mg) as a mixture of 6-ol **84b** and 7-ol **84c** in a 4:1 ratio (based on ^{1}H NMR), which corresponds to 14% and 4% yields respectively.

Data for **84a**: $[\alpha]_D^{23}$ = –52.8 ($CHCl_3$, c = 0.37, n = 3).

^{1}H NMR (400 MHz, MeOH-d_4) δ 8.13 (d, *J* = 7.9 Hz, 1H, 4-H_{Ar}), 7.90 (d, *J* = 7.7 Hz, 1H, 3-H_{Ar}), 4.81 (dd, *J* = 7.5, 3.9 Hz, 1H, 5-H), 3.20 (s, 3H, -SO_2-C*H*$_3$), 3.02 - 2.94 (m, 2H, 8-H_α, 8-H_β), 2.17 - 2.05 (m, 2H, 6-H_α, 7-H_α), 1.95 - 1.79 (m, 2H, 6-H_β, 7-H_β). ^{13}C NMR (101 MHz, MeOH-d_4) δ 160.1 (2/8a-C_{Ar}), 157.3 (2/8a-C_{Ar}), 141.4 (4a-C_{Ar}), 139.6 (4-C_{Ar}), 119.7 (3-C_{Ar}), 68.2 (5-C), 40.5 (-SO_2-*C*H_3), 33.0 (6-C), 32.6 (8-C), 19.7 (7-C). HRMS (ESI, m/z) m/z calculated for $C_{10}H_{13}NO_3S$ [M+H$^+$] 228.0689, found 228.0686. IR (film): v_{max}/cm^{-1} 3487 (O-H), 2941 (C-H_{Ar}), 1303 (S=O), 1153 (C-O), 1126 (C-O), 960.4, 758.

Data for **84b**: $[\alpha]_D^{23}$ = 0.93 (mixture of **84b** and **84c**, $CHCl_3$, c = 0.65, n = 3).

^{1}H NMR (400 MHz, MeOH-d_4) δ 7.78 (d, *J* = 6.0 Hz, 1H, 4-H_{Ar}), 7.78 (d, *J* = 6.0 Hz, 1H, 3-H_{Ar}), 4.27 - 4.21 (m, 2H, 5-H), 3.24 – 3.17 (m, 1H, 5-H_α), 3.16 (s, 3H, -SO_2-C*H*$_3$), 3.13 - 2.97 (m, 1H, 5-H_β), 2.95 - 2.81 (m, 2H, 8-H_α, 8-H_β), 2.07 - 1.96 (m, 1H, 7-H_α), 1.92 – 1.81 (m, 1H, 7-H_β). ^{13}C NMR (101 MHz, MeOH-d_4) δ 158.3 (2/8a-C_{Ar}), 156.4 (2/8a-C_{Ar}), 139.6 (4-C_{Ar}), 138.4 (4a-C_{Ar}), 119.7 (3-C_{Ar}), 66.4 (6-H), 41.7 (5-C), 40.6 (-SO_2-*C*H_3), 30.6 (7-C), 26.0 (8-C). HRMS (ESI, m/z) m/z calculated for $C_{10}H_{13}NO_3S$ [M+H$^+$] 228.0689, found 228.0680. IR (film): v_{max}/cm^{-1} 3401 (O-H), 2930 (C-H_{Ar}), 1444 (C=N), 1303 (S=O), 1154 (C-O), 1125, 960, 767.

Data for 7-ol **84c**: ^{1}H NMR (400 MHz, MeOH-d_4) δ 7.80 (d, *J* = 4.5 Hz, 1H, 4-H_{Ar}), 7.75 (d, *J* = 4.5 Hz, 1H, 3-H_{Ar}), 4.22 - 4.12 (m, 1H, 7-H). 3.24 – 3.17 (m, 1H, 8-H_α), 3.16 (s, 3H, -SO_2-C*H*$_3$), 3.13 - 2.97 (m, 1H, 8-H_β), 2.95–2.81 (m, 2H, 5-H_α, 5-H_β), 2.07 - 1.96 (m, 2H, 6-H_α, 6-H_β). ^{13}C NMR (101 MHz, MeOH-d_4) δ 158.3 (2/8a-C_{Ar}), 156.3 (2/8a-C_{Ar}), 140.7 (4-C_{Ar}), 138.4 (4a-C_{Ar}), 119.6 (3-C_{Ar}), 65.9 (7-C), 40.6 (-SO_2-*C*H_3), 38.0 (8-C), 31.2 (5-C), 29.9 (6-C).

(+)-2-Methoxy-5,6,7,8-tetrahydroquinolin-5-ol **86**

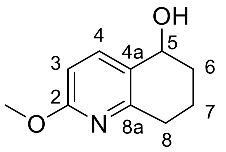

Lyophilised r*Aae*UPO expression supernatant (278 mg, 250 U) was dissolved in pH = 7.0 KPi buffer (100 mM, 15.3 mL) and the solution was diluted with deionised H_2O (11.3 mL). 2-Methoxy-5,6,7,8-tetrahydroquinoline **85** (50.0 mg, 0.306 mmol, 1.00 eq.) was dissolved in MeCN (3.06 mL) and added to the reaction mixture. 30% aqueous H_2O_2 (31.3 µL, 10.0 mg, 0.306 mmol, 1.00 eq.) was diluted with deionised water (1.00 mL) and added to the reaction media dropwise over 25 h at rt. The reaction mixture was extracted with EtOAc (4 x 30 mL), the unified organic phase was washed with brine (15 mL), dried over anhydrous $MgSO_4$, filtered, and evaporated *in vacuo* to yield the crude material as yellow solid. The crude (33 mg) was purified by column chromatography (EtOAc:MeOH = 40:1) to yield pure **86** as white solid. R_f = 0.08 (eluent: EtOAc:MeOH = 40:1). Yield: 24.4 mg (44%). $[\alpha]_D^{20}$ = 3.33 ($CHCl_3$, c = 0.12, n = 3).

^1H NMR (400 MHz, MeOH-d_4) δ 7.53 (d, *J* = 9.3 Hz, 1H, 4-H_{Ar}), 6.44 (d, *J* = 9.3 Hz, 1H, 3-H_{Ar}), 4.55 (dd, *J* = 4.2 Hz, 4.2 Hz 1H, 5-H), 3.50 (s, 3H, -OC*H*3), 2.78 (dt, *J* = 18.1, 5.3 Hz, 1H, 8-H_α), 2.68 - 2.59 (m, 1H, 8-H_β), 2.08 - 1.94 (m, 1H, 7-H_α), 1.87 - 1.75 (m, 3H, 6-H_α, 6-H_β, 7-H_β). ^{13}C NMR (101 MHz, MeOH-d_4) δ 165.4 (2-C_{Ar}), 147.7 (8a-C_{Ar}), 142.9 (4-C_{Ar}), 120.2 (4a-C_{Ar}), 117.2 (3-C_{Ar}), 67.0 (5-C), 31.6 (-OCH_3), 31.1 (6-C), 28.3 (8-C), 18.5 (7-C). HRMS (ESI, m/z) m/z calculated for $C_{10}H_{13}NO_2$ [M+Na$^+$] 202.0838, found 202.0840. IR v_{max}/cm^{-1} 3361 (OH), 2923 (C-H_{Ar}), 2853 (C-H), 1652, 1378, 1106, 832.

(-)-3-Methoxy-5,6,7,8-tetrahydroquinolin-5-ol **88a**, *3-Methoxy-5,6,7,8-tetrahydroquinolin-6-ol* **88b**, *3-Methoxy-5,6,7,8-tetrahydroquinolin-7-ol* **88c**

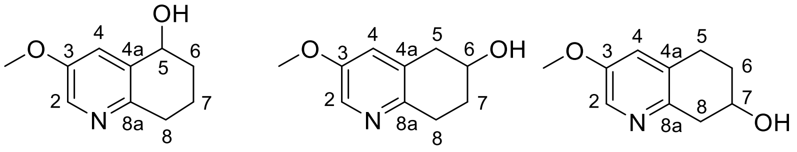

88a **88b** **88c**

3-Methoxy-5,6,7,8-tetrahydroquinoline **87** (30.0 mg, 0.184 mmol, 1.00 eq.) was dissolved in MeCN (1.84 mL) and the solution was added to a solution of lyophilised r*Aae*UPO expression supernatant (167 mg, 150 U) in a mixture of pH = 7.0 KPi buffer (100 mM, 9.20 mL) and deionised H_2O (6.36 mL). 30% H_2O_2 aqueous solution (18.8 µL, 6.25 mg, 0.164 mmol, 1.00 eq.) was diluted with deionised H_2O (1.0 mL) and the solution was added to the reaction mixture over 25 h, at rt. The pH of the reaction mixture was adjusted to pH 13 by the addition of 1.0 M NaOH solution and it was extracted with EtOAc (4 x 30 mL). The unified organic phase was washed with brine (15 mL), dried over anhydrous $MgSO_4$, filtered, and evaporated under reduced pressure to yield the crude material as brown oil. The crude material (40 mg) was purified by preparative thin-layer chromatography (eluent: *n*-Hex:EtOAc = 1:1). Aside from a small amount of recovered starting material (3.0 mg (10%)), two fractions were collected. The major fraction (white amorphous material, R_f = 0.13 (eluent: *n*-Hex:EtOAc = 1:1), 18.9 mg) contained an inseparable mixture of β-alcohols **88b** and **88c** in a

7:3 ratio (according to ^{1}H NMR), which corresponds to 40% and 17% yields, respectively. The minor fraction (transparent amorphous material, R_f = 0.31 (eluent: *n*-Hex:EtOAc = 1:1), yield: 6.3 mg (19%)) contained pure 5-ol **88a**.

Data for 5-ol **88a**: $[\alpha]_D^{21}$ = –53.2 ($CHCl_3$, c = 0.083, n = 3).

^{1}H NMR (500 MHz, $CDCl_3$, T = 233K) δ 8.08 (d, *J* = 2.7 Hz, 1H, 2-H_{Ar}), 6.94 (d, *J* = 2.7 Hz, 1H, 4-H_{Ar}), 4.94 (s, 1H, -O*H*), 4.77 (t, *J* = 6.5 Hz, 1H, 5-H), 3.82 (s, 3H, -OC*H*$_3$), 2.83 (dt, *J* = 16.9, 6.6 Hz, 1H, 8-H_α), 2.75 (dt, *J* = 16.9, 5.7 Hz, 1H, 8-H_β), 2.25 - 2.12 (m, 1H, 6-H_α), 2.04 - 1.95 (m, 1H, 7-H_α), 1.91 – 1.82 (m, 1H, 6-H_β), 1.83 - 1.74 (m, 1H, 7-H_β). ^{13}C NMR (125 MHz, $CDCl_3$, T = 233 K) δ 154.6 (8a-C_{Ar}), 149.8 (3-C_{Ar}), 134.5 (2-C_{Ar}), 132.7 (4a-C_{Ar}), 120.7 (4-C_{Ar}), 68.1 (5-C), 55.7 (-O*C*H$_3$), 30.8 (6-C), 28.8 (8-C), 19.1 (7-C). HRMS (ESI, m/z) calculated for $C_{10}H_{14}NO_2$ [M+H$^+$] 180.1019, found 180.1021. IR (film): v_{max}/cm^{-1} 3258 (O-H), 2937 (C-H), 1592, 1470 (C=N), 1310 (C-N), 1283, 1071 (C-O), 1034, 878.

Data for **88b**: $[\alpha]_D^{21}$ = 14.3 (mixture of **88b** and **88c**, $CHCl_3$, c = 0.29, n = 3).

^{1}H NMR (400 MHz, MeOH-d_4) δ 7.94 (d, *J* = 3.0 Hz, 1H, 2-H_{Ar}), 7.12 (d, *J* = 3.0 Hz, 1H), 4.19 - 4.08 (m, 1H, 6-H), 3.80 (s, 3H, -OC*H*$_3$), 3.06 (dd, *J* = 16.8, 4.9 Hz, 1H, 5-H_α), 3.01 - 2.91 (m, 1H, 8-H_α), 2.85 – 2.78 (m, 1H, 8-H_β), 2.74 (dd, *J* = 16.8, 7.0 Hz, 1H, 5-H_β), 2.06 - 1.93 (m, 1H, 7-H_α), 1.85 - 1.72 (m, 1H, 7-H_β). ^{13}C NMR (101 MHz, MeOH-d_4) δ 156.0 (8a-C_{Ar}), 147.6 (3-C_{Ar}), 135.4 (2-C_{Ar}), 133.9 (4a-C_{Ar}), 122.4 (4-C_{Ar}), 67.2 (6-C), 56.1 (-O*C*H$_3$), 40.6 (5-C), 31.2 (8-C), 26.4 (7-C). HRMS (ESI, m/z) calculated for $C_{10}H_{14}NO_2$ [M+H$^+$] 180.1019, found 180.1021. IR (film): v_{max}/cm^{-1} 3271 (O-H), 2934 (C-H), 1595, 1470 (C=N), 1282 (C-N), 1244, 1037 (C-O), 728.

Data for 7-ol **88c**: ^{1}H NMR (400 MHz, MeOH-d_4) δ 7.93 (d, *J* = 2.8 Hz, 1H, 2-H_{Ar}), 7.11 (d, *J* = 2.8 Hz, 1H, 4-H_{Ar}), 4.10 - 4.05 (m, 1H, 7-H), 3.80 (s, 3H, -OC*H*$_3$), 3.01 - 2.92 (m, 2H, 5-H_α, 8-H_α), 2.85 – 2.73 (m, 2H, 5-H_β, 8-H_β), 2.04 - 1.93 (m, 1H, 6-H_α), 1.92 - 1.83 (m, 1H, 6-H_β). ^{13}C NMR (101 MHz, MeOH-d_4) δ 156.0 (8a-C_{Ar}), 148.8 (3-C_{Ar}), 135.2 (2-C_{Ar}), 132.5 (4a-C_{Ar}), 123.9 (4-C_{Ar}), 66.5 (7-H), 56.1 (-O*C*H$_3$), 38.1 (5-C), 31.8 (6-C), 29.0 (8-C).

3-(Methylsulfonyl)-5,6,7,8-tetrahydroquinolin-6-ol **90a**, *3-(Methylsulfonyl)-5,6,7,8-tetrahydroquinolin-7-ol* **90b**, *(+)-3-(Methylsulfonyl)-5,6,7,8-tetrahydroquinolin-8-ol* **90c**

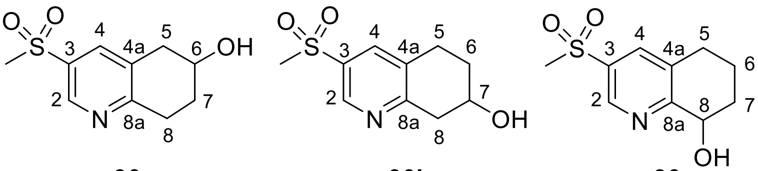

90a **90b** **90c**

Lyophilised r*Aae*UPO expression supernatant (129 mg, 116 U) was dissolved in pH = 7.0 KPi buffer (100 mM, 7.10 mL) and the solution was diluted with deionised H_2O (4.68 mL). 3-(methylsulfonyl)-5,6,7,8-tetrahydroquinoline **89** (30.0 mg, 0.142 mmol, 1.00 eq.) was dissolved in MeCN (1.42 mL) and the solution was added to the enzyme solution. 30% H_2O_2 solution (14.5 µL, 4.80 mg, 0.142 mmol, 1.00 eq.) was diluted and it was added to the reaction mixture dropwise over 25 h at rt. The reaction mixture was extracted with EtOAc (4 x 20 mL), the unified organic phase was washed with brine (20 mL), dried over anhydrous $MgSO_4$, filtered and evaporated *in vacuo* to yield the crude as pale green solid (31 mg). The crude was separated by column chromatography (eluent: DCM:MeOH = 20:1) and two fractions were

yielded. The major fraction (19 mg, pale yellow solid, R_f = 0.22 (eluent: DCM:MeOH = 20:1)) contained a mixture of regioisomers in a in 5:2 ratio 6-ol **90a** and 7-ol **90b** (based on ^{1}H NMR ratio), corresponding to yields of 42% for **90a** and 17% for **90b**. The minor fraction contained pure 8-ol **90c** (white solid, R_f = 0.50 (eluent: DCM:MeOH = 20:1), yield: 4.9 mg (15%)).

Data for **90a**: ^{1}H NMR (400 MHz, MeOH-d$_4$) δ 8.76 (d, *J* = 2.5 Hz, 1H, 2-H$_{Ar}$), 8.04 (d, *J* = 2.5 Hz, 1H, 4-H$_{Ar}$), 4.22 - 4.16 (m, 1H, 6-H), 3.28 - 3.16 (m, 1H, 5-H$_α$), 3.15 (s, 3H, -SO2-C*H*$_3$), 3.13 - 3.09 (m, 1H, 8-H$_α$), 3.06 – 2.82 (m, 2H, 5-H$_β$, 8-H$_β$), 2.09 - 1.84 (m, 2H, 7-H$_α$, 7-H$_β$). ^{13}C NMR (101 MHz, MeOH-d$_4$) δ 162.2 (8a-C$_{Ar}$), 144.7 (2-C$_{Ar}$), 136.8 (4-C$_{Ar}$), 135.7 (3-C$_{Ar}$), 131.7 (4a-C$_{Ar}$), 64.3 (6-C), 43.3 (5-C), 36.4 (8-C), 29.4, 29.2, 28.7. HRMS (ESI, m/z) m/z calculated for $C_{10}H_{13}NO_2S$ [M+H$^+$] 228.0689, found 228.0694.

Data for 7-ol **90b**: $[\alpha]_D^{22}$ = 19.5 ($CHCl_3$, c = 0.17, n = 3).

^{1}H NMR (400 MHz, MeOH-d$_4$) δ 8.77 (d, *J* = 2.8 Hz, 1H, 2-H$_{Ar}$), 8.04 (d, *J* = 2.8 Hz, 1H, 4-H$_{Ar}$), 4.29 – 4.22 (m, 1H, 7-H), 3.13 - 3.09 (m, 1H, 8-H$_α$), 3.15 (s, 3H, -SO$_2$-C*H*$_3$), 3.06 – 2.82 (m, 3H, 8-H$_β$, 5-H$_α$, 5-H$_β$), 2.09 - 1.84 (m, 2H, 6-H$_α$, 6-H$_β$). ^{13}C NMR (101 MHz, MeOH-d$_4$) δ 161.0 (8a-C$_{Ar}$), 144.8 (2-C$_{Ar}$), 135.1 (3-C$_{Ar}$), 135.1 (4-C$_{Ar}$), 133.2 (4a-C$_{Ar}$), 64.9 (7-C), 40.5 (8-C), 29.4, 29.2, 28.7.

Data for 8-ol **90c**: ^{1}H NMR (400 MHz, MeOH-d$_4$) δ 8.87 (d, *J* = 2.2 Hz, 1H, 2-H$_{Ar}$), 8.07 (d, *J* = 2.2 Hz, 1H, 4-H$_{Ar}$), 4.74 (dd, *J* = 5.4, 5.4 Hz, 1H, 8-H), 3.16 (s, 3H, -SO$_2$-C*H*$_3$), 2.95 (ddd, *J* = 17.0, 5.9 Hz, 1H, 5-H$_α$), 2.85 (ddd, *J* = 17.0, 5.9 Hz, 1H, 5-H$_β$), 2.14 - 1.93 (m, 3H, 7-H$_α$, 7-H$_β$, 6-H$_α$), 1.87 - 1.75 (m, 1H, 6-H$_β$). ^{13}C NMR (101 MHz, MeOH-d$_4$) δ 163.9 (8a-C$_{Ar}$), 146.4 (2-C$_{Ar}$), 137.7 (4-C$_{Ar}$), 137.4 (4a-C$_{Ar}$), 135.2 (3-C$_{Ar}$), 69.5 (8-C), 44.5 (-SO$_2$-*C*H$_3$), 32.2 (7-C), 29.4 (5-C), 17.8 (6-C). HRMS (ESI, m/z) m/z calculated for $C_{10}H_{13}NO_2S$ [M+Na$^+$] 250.0508, found 250.0516. IR (film): v_{max}/cm^{-1} 3375 (O-H), 2925 (C-H), 1310 (S=O), 1140, 764.

2-Oxindole **51**

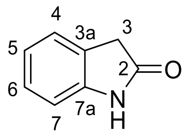

51

Lyophilized *rAae*UPO expression supernatant (68 mg, 66 U) was dissolved in pH = 7.0 KPi buffer (100 mM, 25 mL) and the solution was diluted with deionised water (10 mL). Indole **48** (59.0 mg, 0.500 mmol, 1.00 eq.) was dissolved in MeCN (5.0 mL) and the solution was added to the reaction mixture. 30% H_2O_2 solution (51.1 µL, 17.0 mg, 0.500 mmol, 1.00 eq.) was diluted in deionised H_2O (10.0 mL) and added to the mixture dropwise over 13 h at rt. The reaction was stirred for another 20 h and it was extracted with EtOAc (4 x 50 mL). To aid phase separation and extraction, the aqueous phase was saturated with NaCl. The unified organic phase was dried over anhydrous $MgSO_4$, filtered, and evaporated under reduced pressure. The crude material was separated by column chromatography (eluent: EtOAc:n-Hex = 6:4), 2-oxindole **51** was yielded as brown solid. R_f = 0.60 (eluent: EtOAc/n-Hex = 6:4). Yield: 40 mg (60%).

^{1}H NMR (400 MHz, $CDCl_3$) δ 9.49 (broad s, 1H, -N*H*), 7.21 (d, *J* = 7.3 Hz, 1H, 7-H$_{Ar}$), 7.21 (t, *J* = 7.3 Hz, 1H, 5-H$_{Ar}$), 7.01 (t, *J* = 7.4 Hz, 1H, 6-H$_{Ar}$), 6.91 (d, *J* = 8.1 Hz, 1H, 4-H$_{Ar}$), 3.54 (s,

2H, 3-H_α, 3-H_β). ^{13}C NMR (101 MHz, $CDCl_3$) δ 178.5 (2-C), 142.8 (7a-C_{Ar}), 128.02 (5-C_{Ar}), 125.4 (3a-C_{Ar}), 124.6 (7-C_{Ar}), 122.4 (6-C_{Ar}), 110.0 (4-C_{Ar}), 31.7 (2-C). HRMS (ESI, m/z) m/z calculated for C_8H_7NO [M+Na$^+$] 156.0420, found 156.0418. The data match those reported in the literature.[62]

1-Methylindolin-2-one **52**

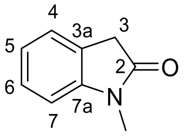

52

Lyophilized *rAae*UPO expression supernatant (46 mg, 44 U) was dissolved in pH = 7.0 KPi buffer (100 mM, 10 mL) in a 150 mL conical flask and the solution was diluted with deionised water (8.2 mL). This was followed by the addition of 1-methylindole **49** (52.5 mg, 0.400 mM, 1.00 eq) in MeCN (2.0 mL). *Pp*AOx stock solution (5.83 µL in sucrose, 20U) was prepared in pH = 7.0 KPi buffer (100 mM, 0.5 mL) and added to the reaction mixture. The reaction was started by the addition of MeOH (0.32 mL, 400 mM final concentration). The flask was shaken at 200 rpm at rt for 20 h and the reaction media was extracted with EtOAc (4 x 20 mL) and *n*-BuOH (1 x 20 mL) The unified organic phase was dried over anhydrous $MgSO_4$, filtered, and evaporated *in vacuo*. The crude product was purified by column chromatography (DCM/MeOH = 19:1) to yield the product as brown oil. R_f = 0.80 (eluent: DCM/MeOH = 19:1). Yield: 44 mg (75%).

^{1}H NMR (400 MHz, $CDCl_3$) δ 7.26 (t, *J* = 7.6 Hz, 1H, 5-H_{Ar}), 7.22 (d, *J* = 7.5 Hz, 1H, 4-H_{Ar}), 7.02 (t, *J* = 7.7 Hz, 1H, 6-H_{Ar}), 6.80 (d, *J* = 7.8 Hz, 1H, 7-H_{Ar}), 3.49 (s, 2H, 3-H_α, 3-H_β), 3.18 (s, 3H, -C*H*$_3$). ^{13}C NMR (101 MHz, $CDCl_3$) δ 175.2 (2-C), 145.3 (7a-C_{Ar}), 128.0 (6-C_{Ar}), 124.6 (3a-C_{Ar}), 124.4 (4-C_{Ar}), 122.4 (5-C_{Ar}), 108.2 (7-C_{Ar}), 35.8 (3-C), 26.2 (-*C*H_3). HRMS (ESI, m/z) m/z calculated for C_9H_9NO [M+Na$^+$] 170.0576, found 170.078. The data match those reported in the literature.[63]

3-Methylindolin-2-one **53**, *3-Hydroxy-3-Methyloxindole* **91**

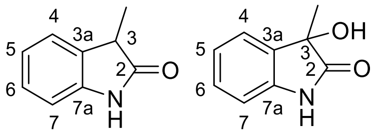

53 **91**

Lyophilized r*Aae*UPO supernatant (227 mg, 220 U) was dissolved in pH = 7.0 KPi buffer (100 mM, 50 mL) and the solution was diluted with deionised water (20 mL). Scatole **50** (131 mg, 1.00 mmol, 1.00 eq.) was dissolved in MeCN (20 mL) and the solution was added to the reaction mixture. 30% H_2O_2 solution (102 µL, 34.0 mg, 1.00 mmol, 1.00 eq.) was diluted in deionised H_2O (20.0 mL) and added to the reaction dropwise over 25 h to the reaction at rt. The reaction media was saturated with NaCl and extracted with EtOAc (3 x 50 mL) and *n*-BuOH (1 x 50 mL). The organic phases were unified, dried over anhydrous $MgSO_4$, filtered, and evaporated under reduced pressure. The crude material was separated by column chromatography (eluent: EtOAc:n-Hex = 7:3 to 1:1), two fractions were collected. The major fraction contained 3-methylindolin-2-one **53** (yellow microcrystalline solid, R_f = 0.70 (eluent:

EtOAc:*n*-Hex = 1:1), yield: 74 mg (50%)), the minor fraction contained 3-hydroxy-3-methyloxindole **91** (brown oil, R_f = 0.42 (eluent: EtOAc:*n*-Hex = 1:1), yield: 32 mg (20%)).

Data for **53**: ^{1}H NMR (400 MHz, $CDCl_3$) δ 9.52 (s, 1H, -N*H*), 7.21 (d, *J* = 7.6 Hz, 1H, 7-H_{Ar}), 7.21 (t, *J* = 7.6 Hz, 1H, 5-H_{Ar}), 7.03 (t, *J* = 7.6 Hz, 1H, 6-H_{Ar}), 6.94 (d, *J* = 7.6 Hz, 1H, 4-H_{Ar}), 3.48 (q, *J* = 7.7 Hz, 1H, 3-H), 1.51 (d, *J* = 7.6 Hz, 3H, -CH_3). ^{13}C NMR (101 MHz, $CDCl_3$) δ 182.1 (2-C), 141.5 (7a-C_{Ar}), 131.4 (3a-C_{Ar}), 128.0 (5-C_{Ar}), 123.8 (7-C_{Ar}), 122.4 (6-C_{Ar}), 110.0 (4-C_{Ar}), 41.3 (3-C), 15.3 (-*C*H_3). HRMS (ESI, m/z) m/z calculated for C_9H_9NO [M+Na^+] 170.0576, found 170.0578. The data match those reported in the literature.[64]

Data for **91**: ^{1}H NMR (400 MHz, $CDCl_3$) δ 8.75 (s, 1H, -N*H*), 7.37 (d, *J* = 7.4 Hz, 1H, 7-H_{Ar}), 7.22 (t, *J* = 7.6 Hz, 1H, 5-H_{Ar}), 7.05 (t, *J* = 7.4 Hz, 1H, 6-H_{Ar}), 6.86 (d, *J* = 7.8 Hz, 1H, 4-H_{Ar}), 3.88 (s, 1H, -O*H*), 1.59 (s, 3H, -CH_3). ^{13}C NMR (101 MHz, $CDCl_3$) δ 181.4 (2-C), 140.0 (7a-C_{Ar}), 132.1 (3^a-C_{Ar}), 129.7 (6-C_{Ar}), 123.9 (4-C_{Ar}), 123.3 (5-C_{Ar}), 110.7 (7-C_{Ar}), 74.2 (-*C*-OH), 24.7 (-*C*H_3). HRMS (ESI, m/z) m/z calculated for $C_9H_9NO_2$ [M+Na^+] 186.0525, found 186.0523. The data match those reported in the literature.[65]

Section 12. Representative Gas Chromatograms of Substrate Oxidations

Representative GC-FID and HPLC-DAD spectra are listed, starting material peaks are highlighted in each case. Compounds identified by the use of authentic standards are also shown on the spectra.

Separation methods

Achiral GC-FID Method

Samples were injected from EtOAc solution. 1 µL injection, 40:1 split ratio, inlet temp.: 250 °C, FID temp.: 300 °C, unless noted otherwise.

Substituted pyridines and alkylbenzenes: 100 °C pre-run temperature for 2 min; gradient heating to 130 °C (10 °C min^{-1}) and between 130 and 220 °C (40 °C min^{-1}). *Bicyclic substrates*: 120 °C pre-run temperature for 2 min, 15 °C min^{-1} to 220 °C, 220 °C post-run temperature for 5 min. *Design of experiment GC method*: 25:1 split ratio, linear gradient between 70 °C and 320 °C (25 °C min^{-1}).

Achiral HPLC-DAD Method

Samples were prepared by diluting the aqueous reaction mixture with MeOH in a 9:1 ratio. The samples were centrifuged at 13.000 x *g* for 30 min. 1 µL of supernatant was directly injected. Flow rate: 0.4 mL min^{-1}, eluent A: 20 mM KPi buffer (pH = 7.0), eluent B: MeCN. Gradient elution from 10 % to 50 % eluent B over 35 min, then isocratic elution at 50 % eluent B for 5 min.

3-Ethylpyridine **10**

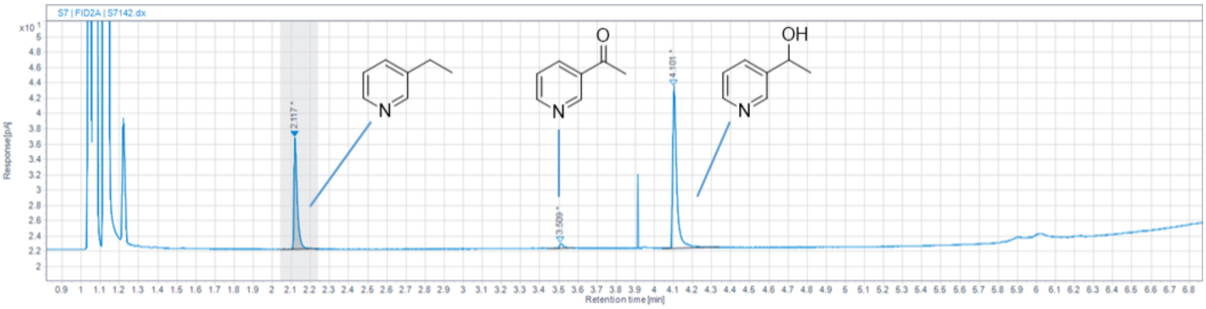

Separation method: GC.

2-Ethylpyridine **14**

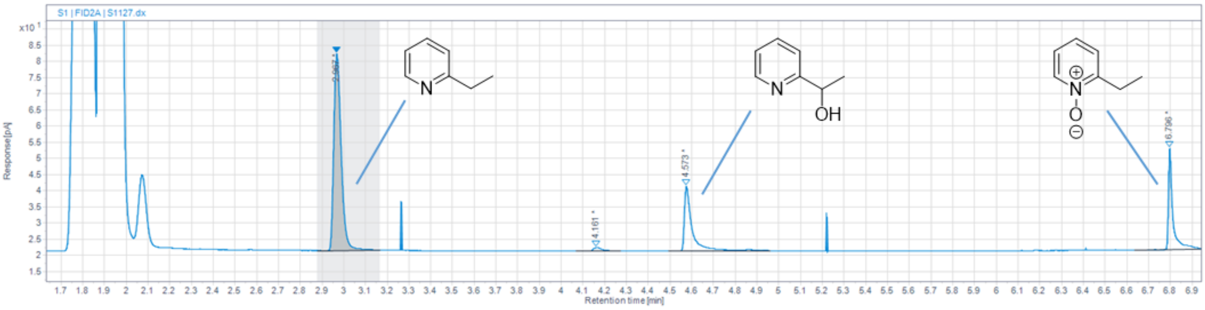

Separation method: GC.

3-Isopropylpyridine **12**

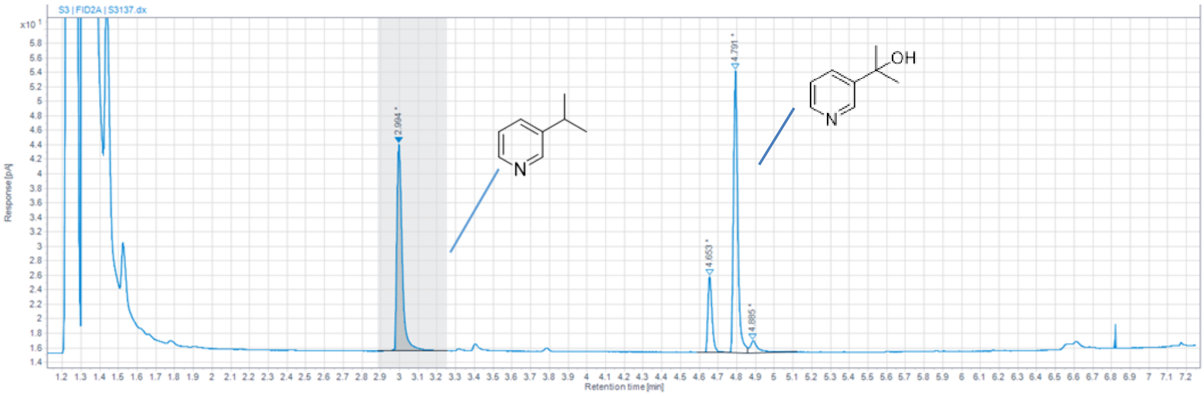

Separation method: GC.

2-Isopropylpyridine **16**

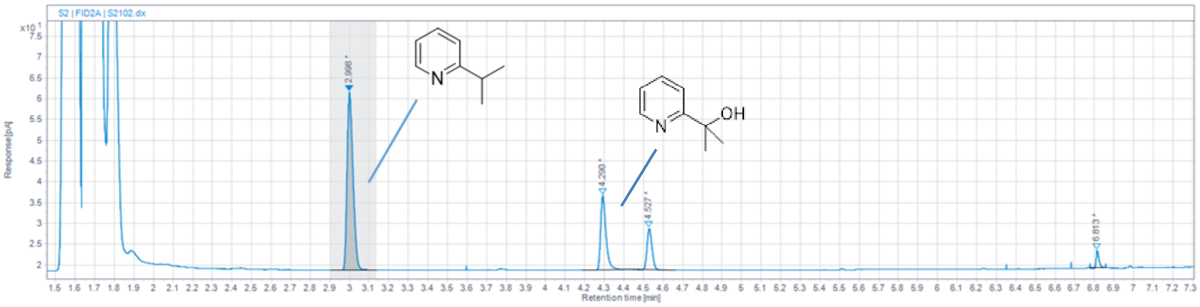

Separation method: GC.

3-Propylpyridine **11**

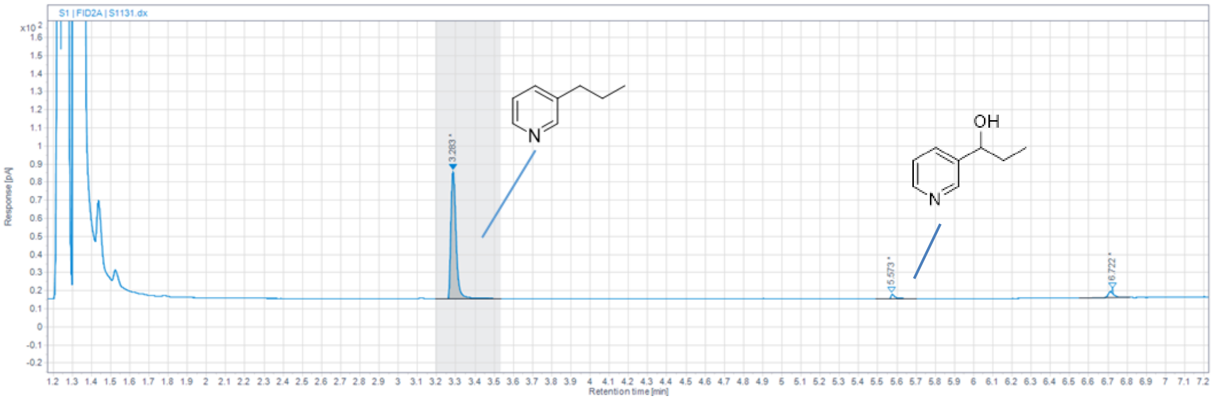

Separation method: GC.

*2-n-Propylpyridine **15***

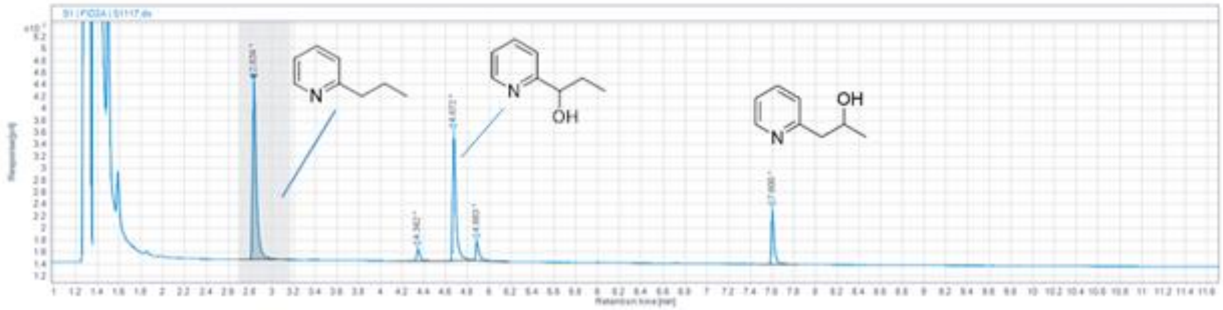

Separation method: GC.

2-Methoxy-5-methylpyridine **17**

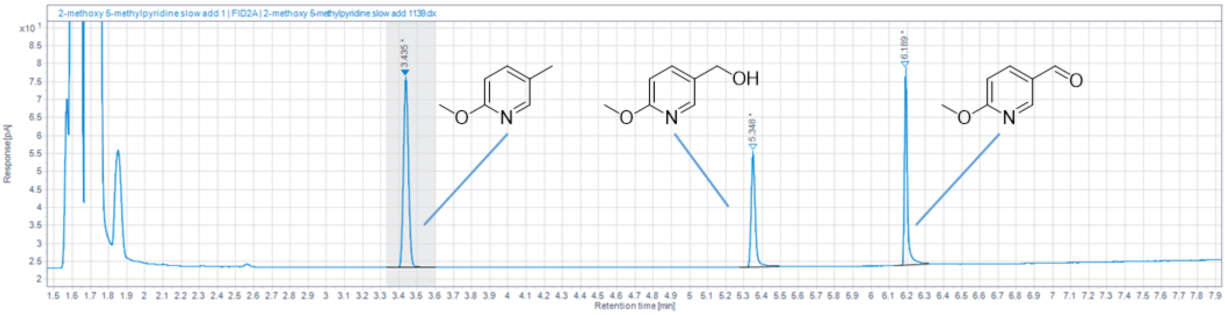

Separation method: GC.

2,3-Cyclopentenopyridine **1**

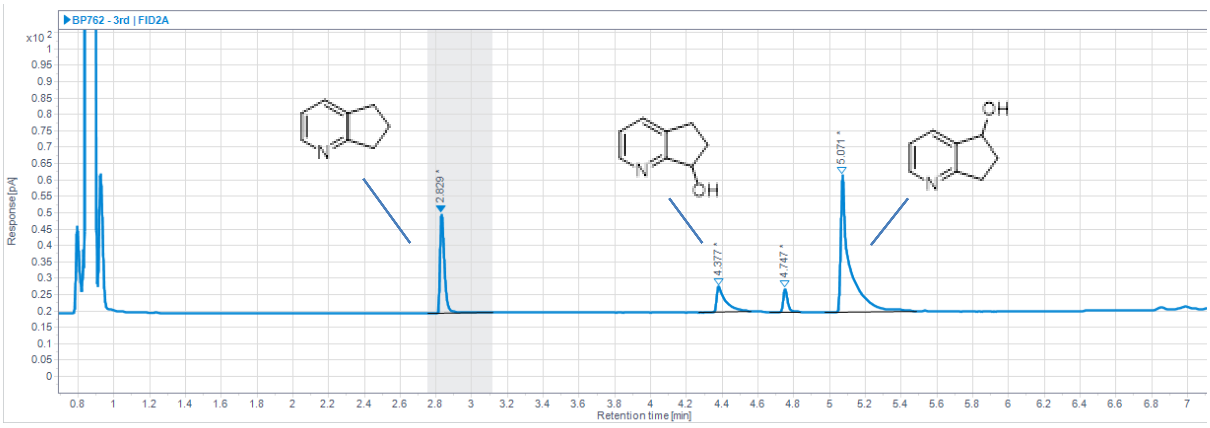

Separation method: GC.

5,6,7,8-Tetrahydroquinoline **3**

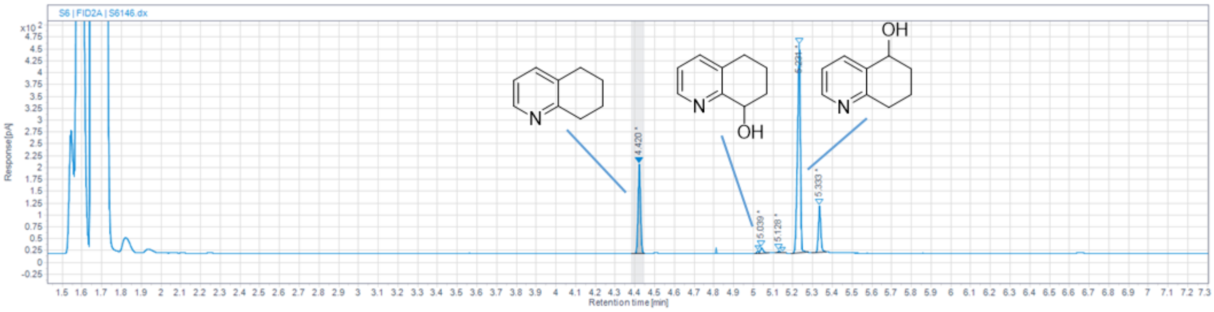

Separation method: GC.

5,6,7,8-Tetrahydroisoquinoline **30**

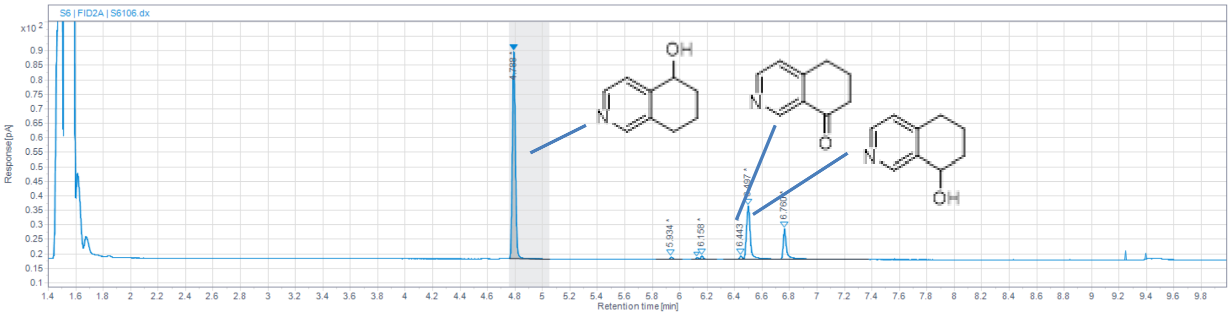

Separation method: GC.

1,2,3,4-Tetrahydroquinoline **31**

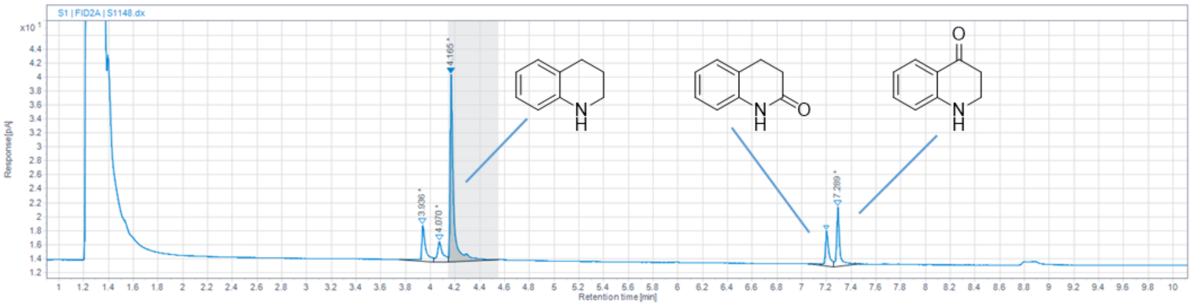

Separation method: GC.

1,2,3,4-Tetrahydroisoquinoline **32**

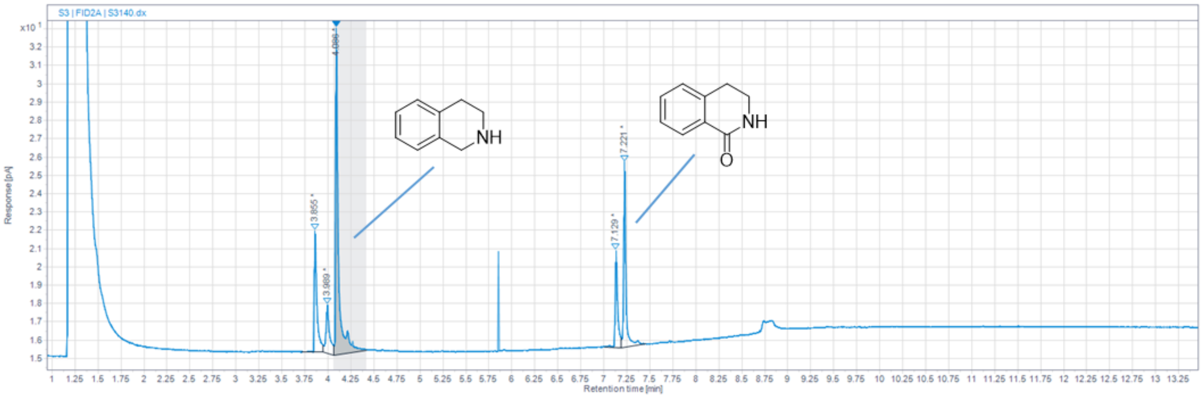

Separation method: GC.

Quinoline **33**

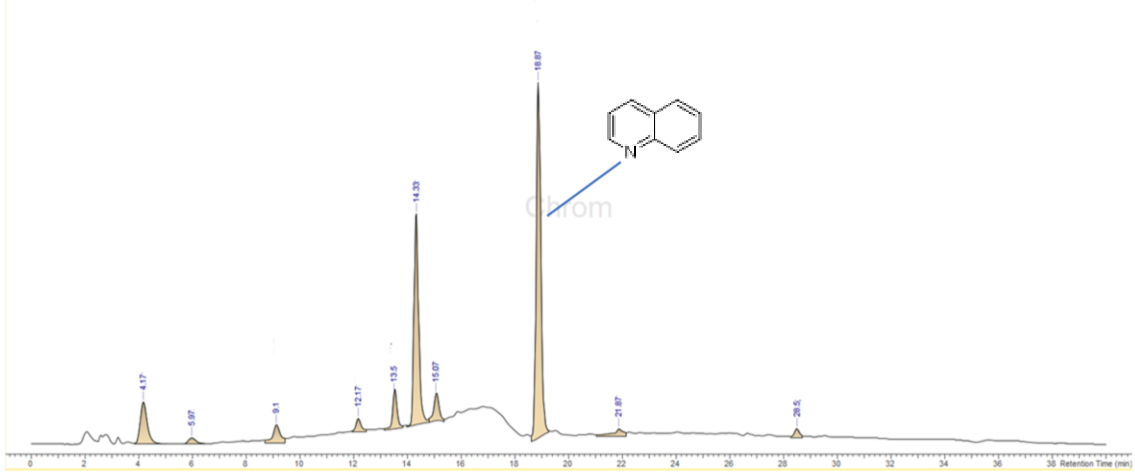

Separation method: HPLC (detection at 254 nm).

Quinaldine **35**

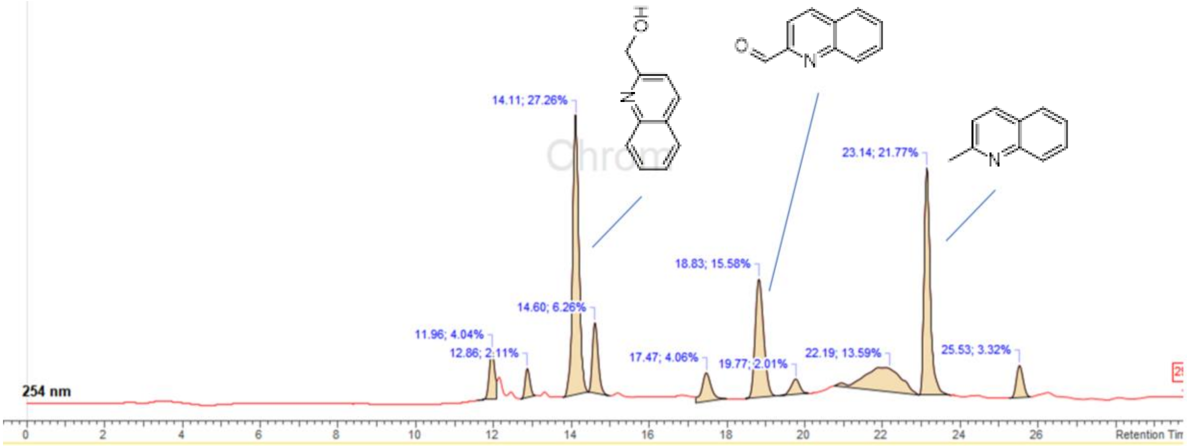

Separation method: HPLC (detection at 254 nm).

3-Methylisoquinoline **36**

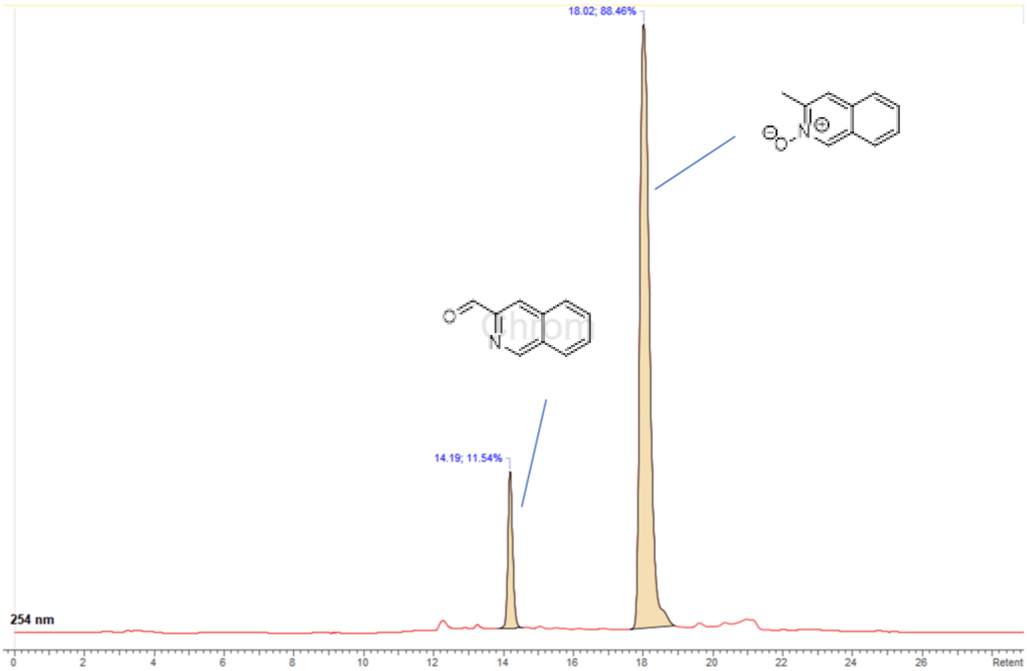

Separation method: HPLC (detection at 254 nm).

Indole **48**

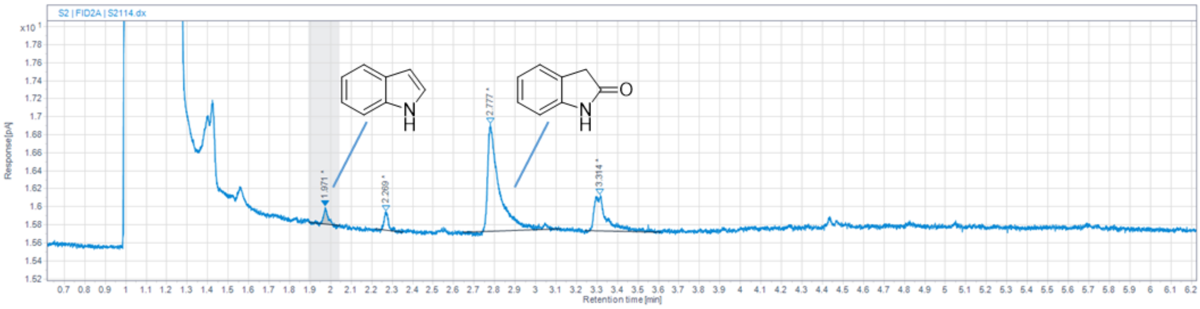

Separation method: GC

1-Methylindole **49**

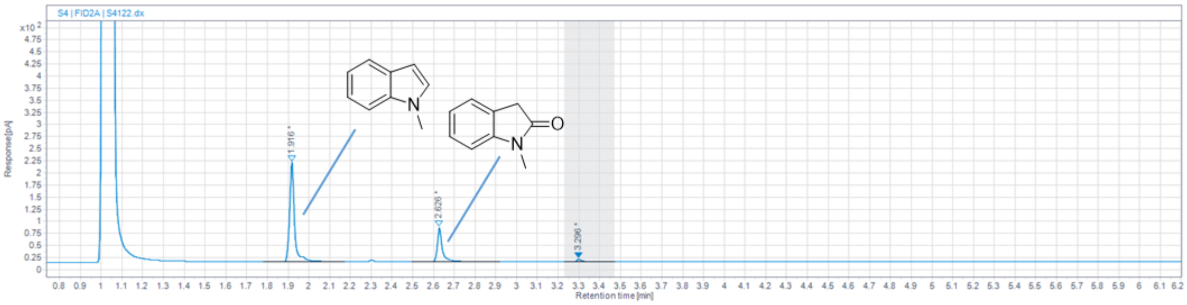

Separation method: GC.

3-Methylindole **50**

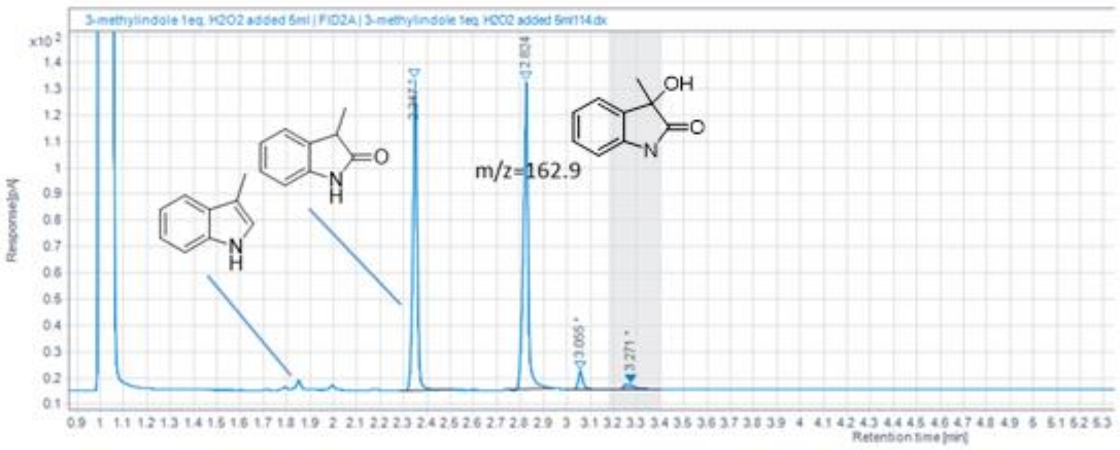

Separation method: GC.

Section 13. Chiral Chromatograms

(R)-1-(Pyridine-3-yl)-ethanol (*R*)-**20**

Method: Separation was performed on a Supelco β-DEX^{TM} 120 capillary column (30 m x 0.25 mm, d_f = 0.25 µm). Samples were injected from EtOAc solution. 0.2 µL injection, 40:1 split ratio, inlet temp.: 250 °C, FID temp.: 300 °C, isotherm 130 °C column temperature, flow speed: 42.53 cm/s.

Stereoisomers were identified according to literature.[66]

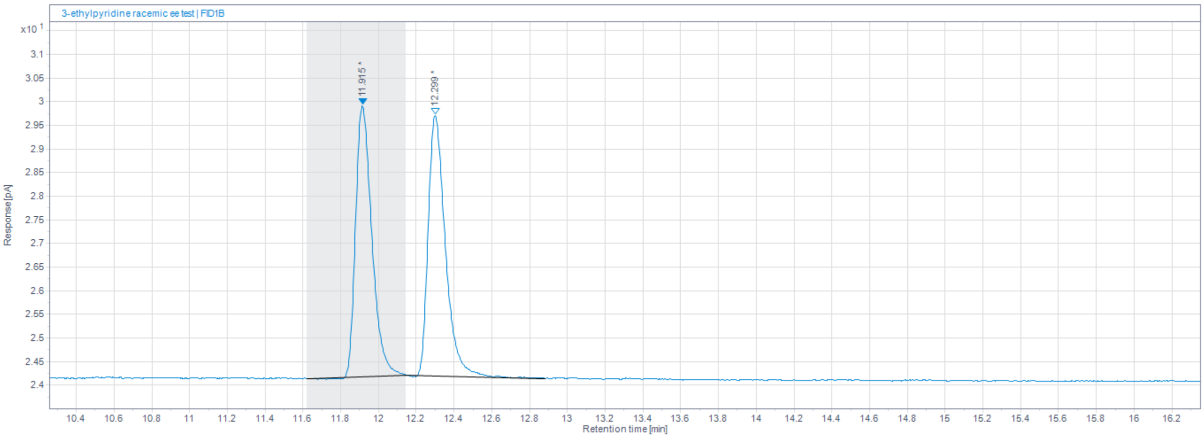

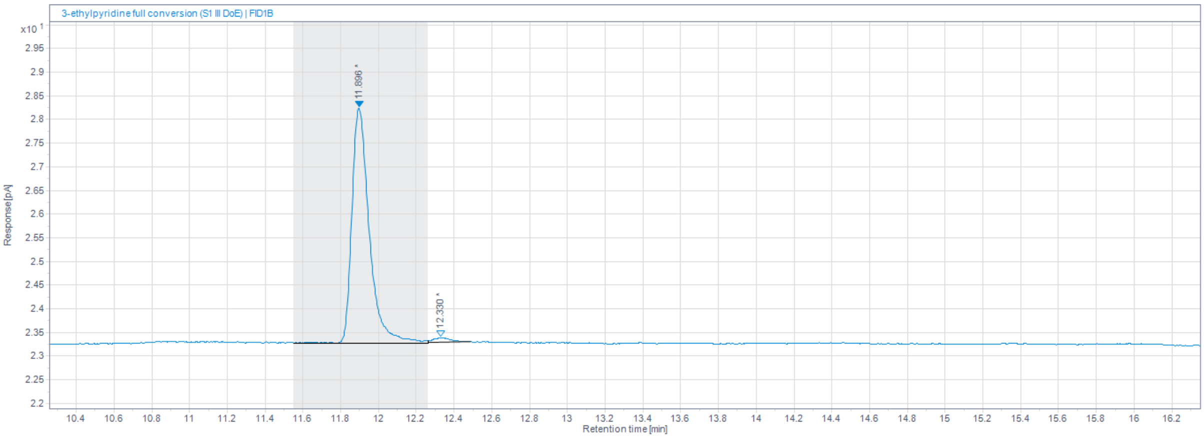

Retention time	Compound	%
**11.9 min**	**(*R*)-1-(pyridine-3-yl)ethanol**	**98.1**
**12.3 min**	**(*S*)-1-(pyridine-3-yl)ethanol**	**1.9**

(R)-1-(Pyridine-2-yl)-ethanol (*R*)-**22**

Method: Separation was performed on a Supelco β-DEX^{TM} 120 capillary column (30 m x 0.25 mm, d_f = 0.25 µm). 0.2 µL injection from EtOAc solution (1 mg/mL), 40:1 split ratio, inlet temp.: 250 °C, FID temp.: 300 °C, column temp.: 90 °C, flow speed: 40.75 cm/s.

Stereoisomers were identified according to literature.[66]

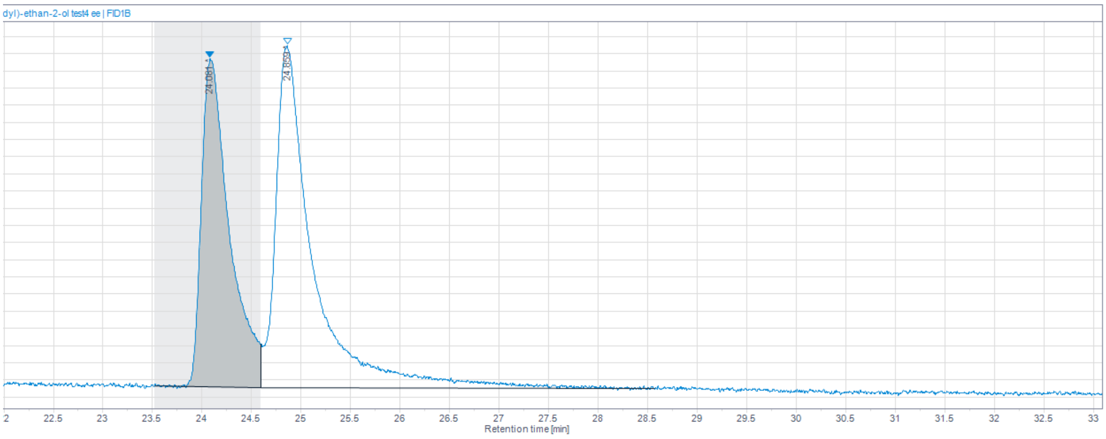

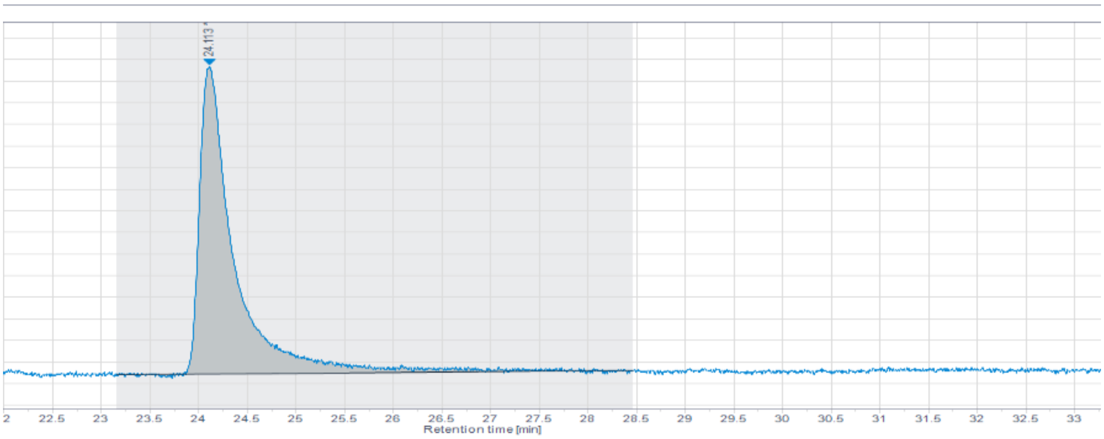

Retention time	Compound	%
**24.1**	**(*R*)-1-(pyridine-2-yl)ethanol**	**n.d.**
**24.9**	**(*S*)-1-(pyridine-2-yl)ethanol**	**>99**

(R)-1-(Pyridine-2-yl)-propanol (*R*)-**27**

Method: Separation was performed on a Daicel Chiralcel(R) OD-H HPLC column (250 x 4.60 mm, 5 µm). 10 µL injection from MeOH solution (1 mg/mL), 25 °C column temperature, 0.3 mL/min flow, eluent: IPA/n-hexane 5/95, detection: 254 nm.

Stereoisomers were identified according to literature.[67]

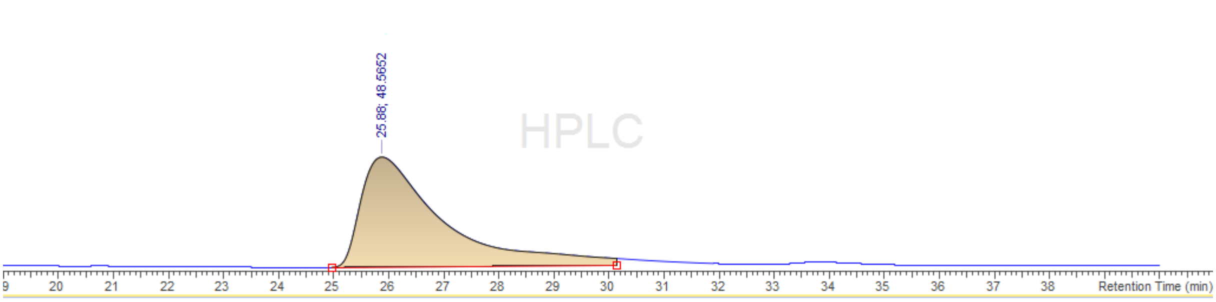

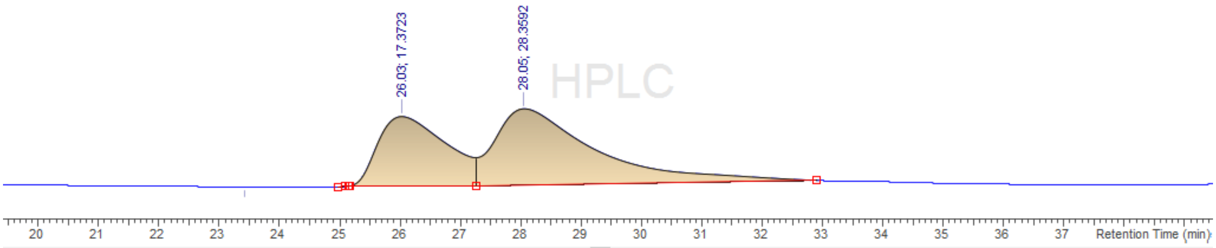

Retention time	Compound	%
**25.9**	**(*R*)-1-(pyridine-2-yl)propanol**	**>99**
**28.1**	**(*S*)-1-(pyridine-2-yl)propanol**	**n.d.**

*(-)-6,7-Dihydro-5H-cyclopenta[b]pyridin-5-ol (R)-***2**

Method: Separation was performed on a Daicel Chiralpak[(R)] AD-H HPLC column (150 x 4.60 mm, 5 µm). 10 µL injection from *n*-Hex/IPA 80/20 solution (1 mg/mL), 25 °C column temperature, 1.0 mL/min flow, eluent: IPA/n-hexane 20/80, detection: 230 nm.

The absolute stereochemistry was assigned compared to the available (*S*)-enantiomer.

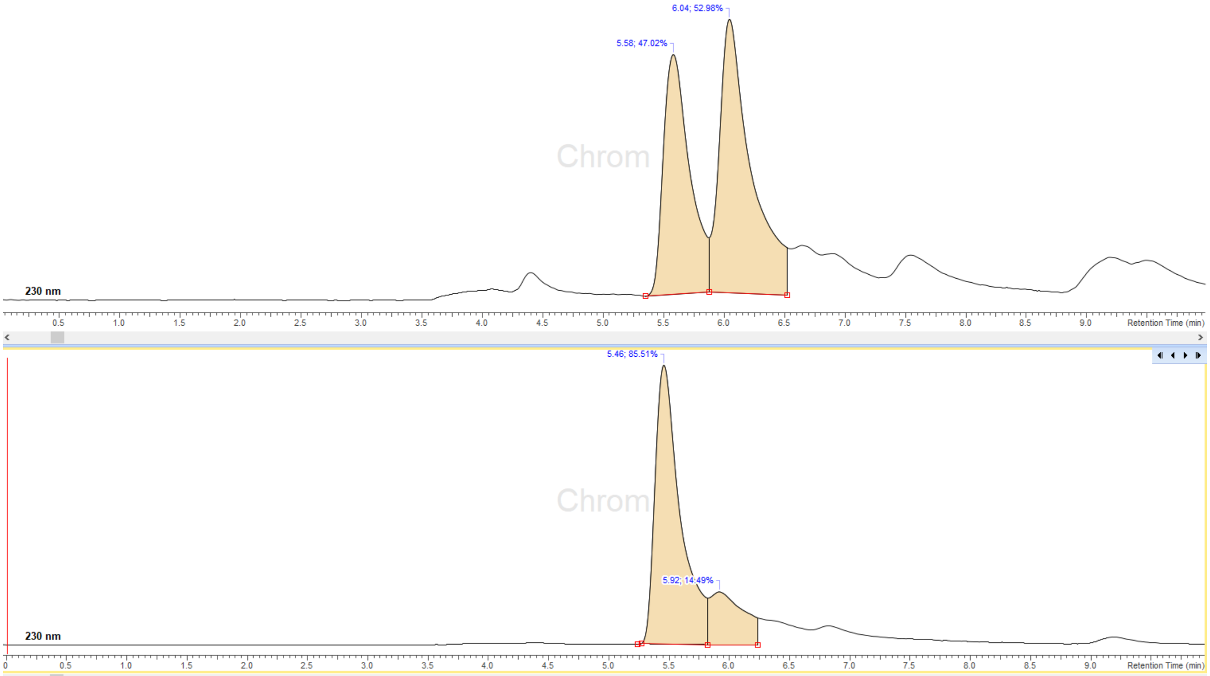

Retention time	Compound	%
**5.58**	**(*R*)-6,7-dihydro-5*H*-cyclopenta[*b*]pyridin-5-ol**	**88.1**
**6.04**	**(*S*)-6,7-dihydro-5*H*-cyclopenta[*b*]pyridin-5-ol**	**11.8**

*(+)-6,7-dihydro-5*H*-cyclopenta[*b*]pyridin-7-ol (R)*-**55**

Method: Separation was performed on a Daicel Chiralpak(R) AD-H HPLC column (150 x 4.60 mm, 5 µm). 10 µL injection from *n*-Hex/IPA 90/10 solution (1 mg/mL), 25 °C column temperature, 0.75 mL/min flow, eluent: *n*-Hex/IPA 95/5, detection: 230 nm.

The absolute stereochemistry was assigned according to the known optical rotation of *(R)*-**55**.[68]

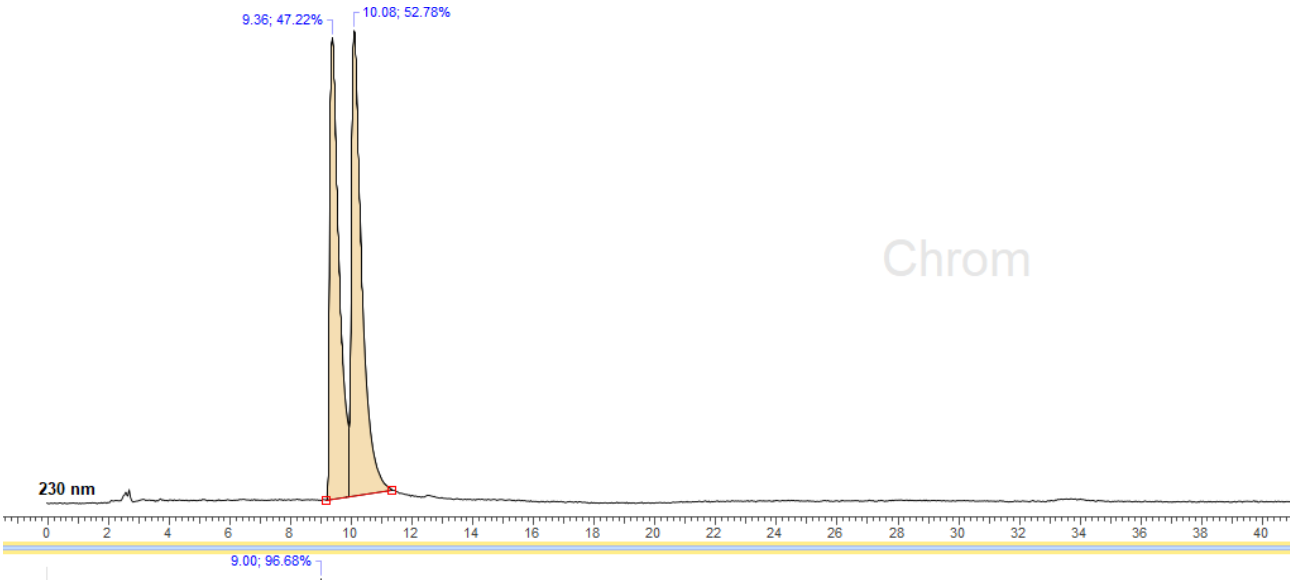

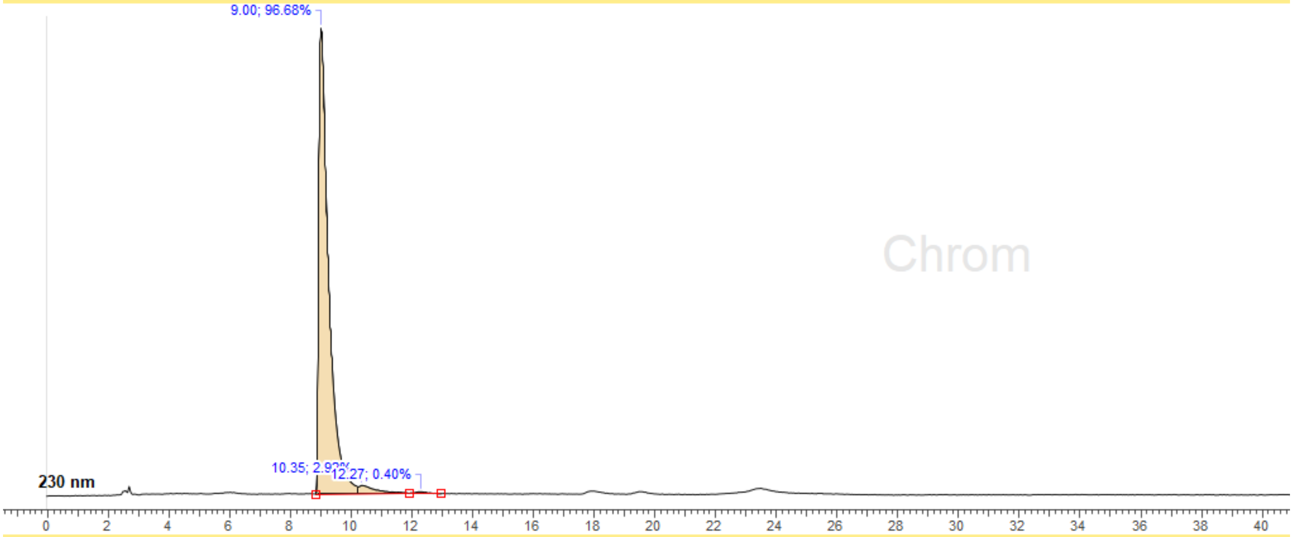

(+)-1-(6-Methoxypyridin-3-yl)-ethan-1-ol (*R*)-**67a**

Method: Separation was performed on a Daicel Chiralcel(R) OJ-H HPLC column (250 x 4.60 mm, 5 µm). 10 µL injection from 90/10 *n*-Hex/IPA solution (1 mg/mL), 25 °C column temperature, 0.5 mL/min flow, eluent: IPA/n-hexane 10/90, detection: 230 nm.

Absolute stereochemistry was assigned according to the known optical rotation of the (*R*)-enantiomer.[69]

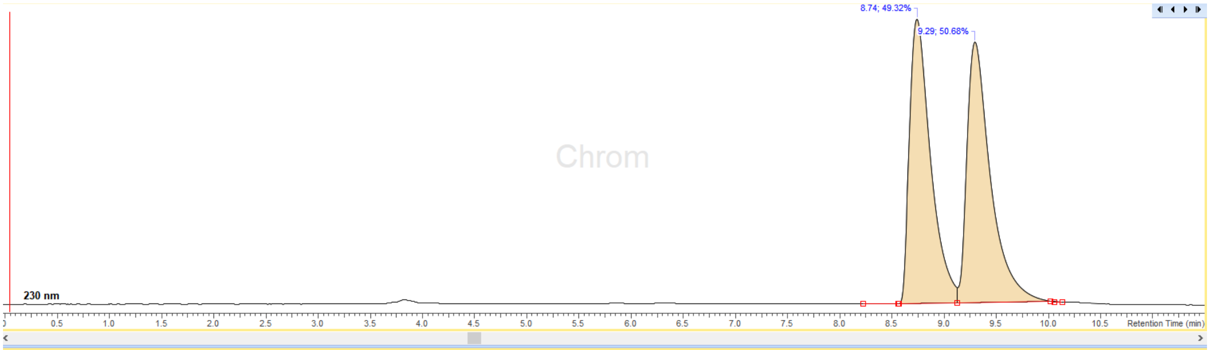

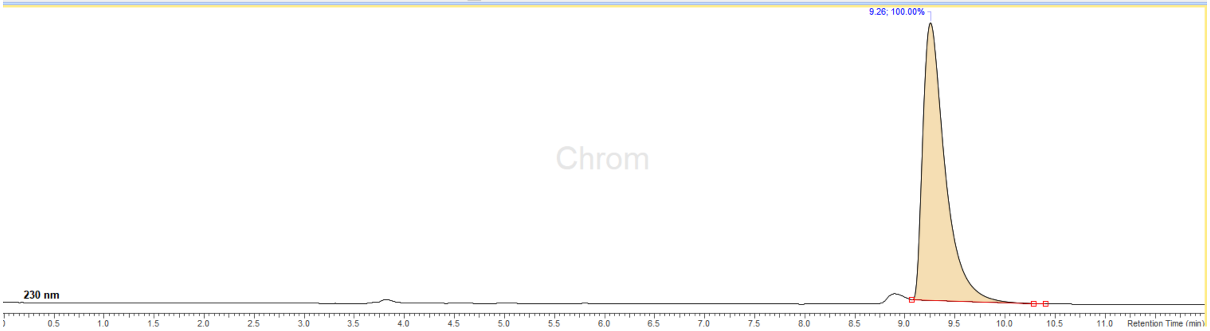

Retention time	Compound	%
**8.74**	**(*R*)-1-(6-Methoxypyridin-3-yl)-ethan-1-ol**	**>99**
**9.29**	**(*S*)-1-(6-Methoxypyridin-3-yl)-ethan-1-ol**	**n.d.**

(+)-5-(1-Hydroxyethyl)-picolinonitrile (*R*)-**73**

Method: Separation was performed on a Daicel Chiralpak[(R)] AD-H HPLC column (150 x 4.60 mm, 5 µm). 10 µL injection from 90/10 *n*-Hex/IPA solution (1 mg/mL), 25 °C column temperature, 0.9 mL/min flow, eluent: IPA/n-hexane 10/90, detection: 230 nm.

Absolute stereochemistry was assigned by analogy with the retention times of known 2-substituted (R)- and (S)-(pyridin-3-yl)-ethan-1-ols (*vide infra*).

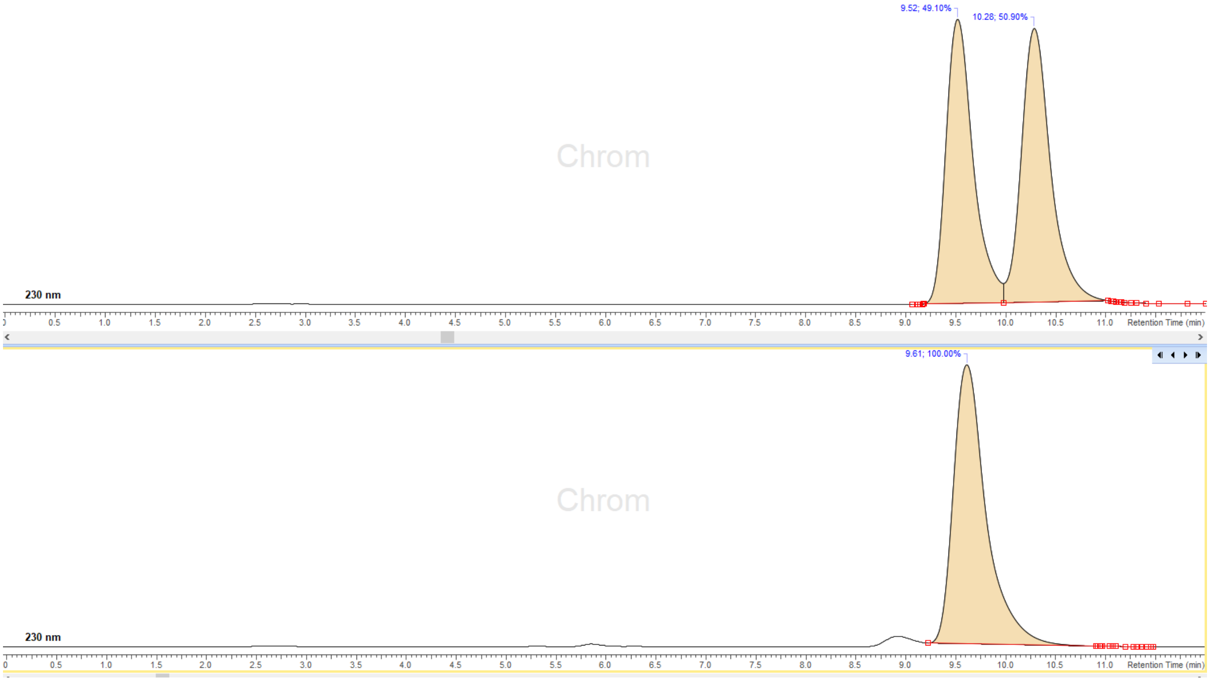

Retention time	Compound	%
**9.52**	**(+)-1-(6-Methoxypyridin-3-yl)-ethan-1-ol**	**>99**
**10.3**	**(-)-1-(6-Methoxypyridin-3-yl)-ethan-1-ol**	**n.d.**

(+)-5-(1-Hydroxyethyl)-picolinamide (*R*)-**74**

Method: Separation was performed on a Daicel Chiralcel(R) OJ-H HPLC column (250 x 4.60 mm, 5 µm). 10 µL injection from 90/10 *n*-Hex/IPA solution (1 mg/mL), 25 °C column temperature, 0.75 mL/min flow, eluent: IPA/n-hexane 20/80, detection: 230 nm.

Absolute stereochemistry was assigned by analogy with the retention times of known 2-substituted (R)- and (S)-(pyridin-3-yl)-ethan-1-ols (*vide infra*).

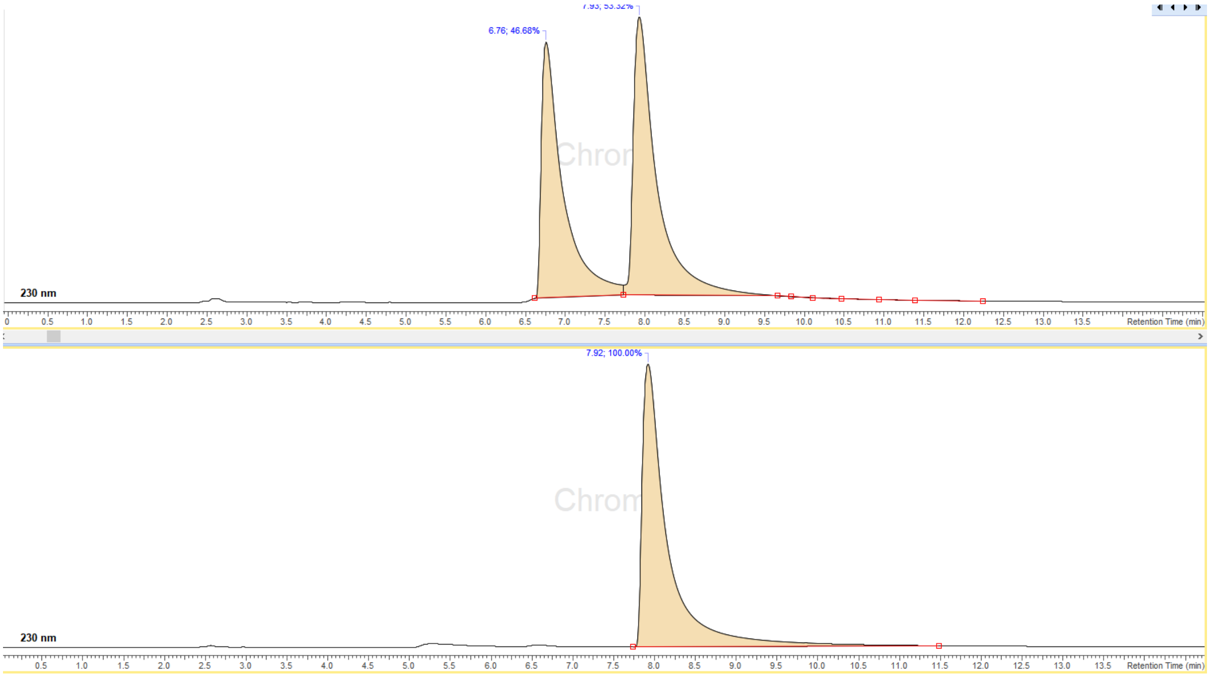

Retention time	Compound	%
**6.76**	**(-)-5-(1-Hydroxyethyl)-picolinamide**	**n.d.**
**7.93**	**(+)-5-(1-Hydroxyethyl)-picolinamide**	**>99.9**

(+)-1-(6-(Methylsulfonyl)pyridin-3-yl)-ethan-1-ol (*R*)**-75**

Method: Separation was performed on a Daicel AD-H HPLC column (250 x 4.60 mm, 5 µm). 10 µL injection from 90/10 *n*-Hex/IPA solution (1 mg/mL), 25 °C column temperature, 0.75 mL/min flow, eluent: IPA/n-hexane 20/80, detection: 230 nm.

Absolute stereochemistry was assigned by analogy with the retention times of known 2-substituted (R)- and (S)-(pyridin-3-yl)-ethan-1-ols (*vide infra*).

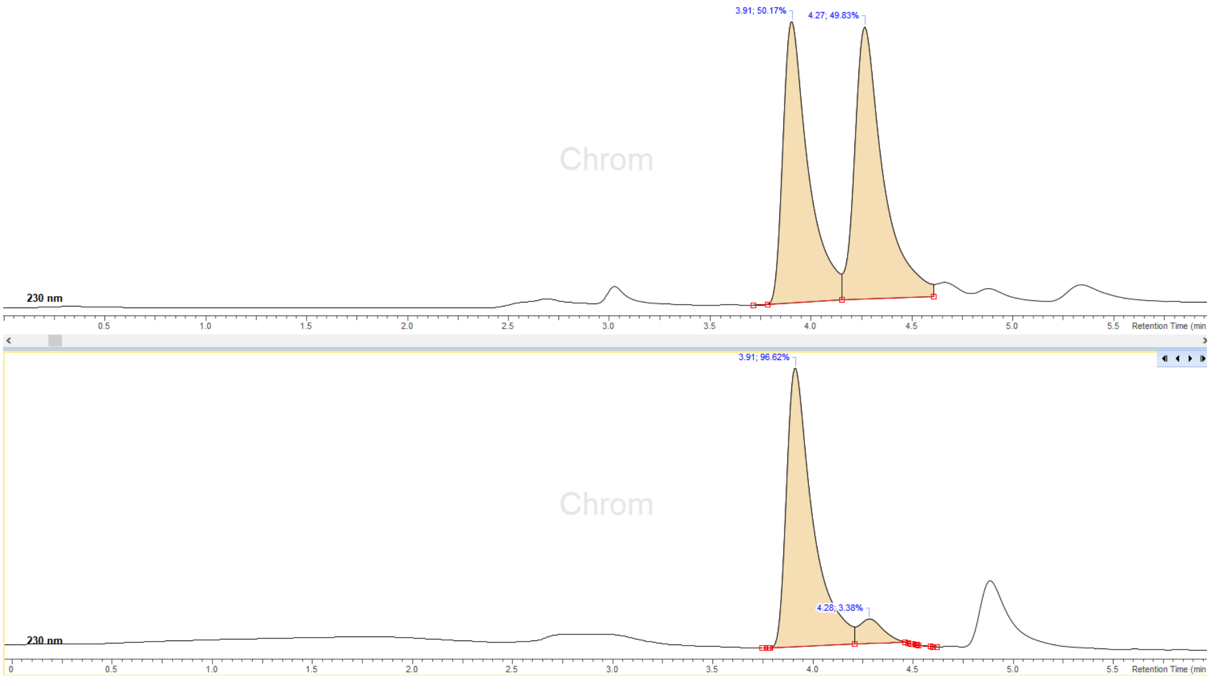

Retention time	Compound	%
**3.91**	**(+)-1-(6-(Methylsulfonyl)pyridin-3-yl)ethan-1-ol**	**92.3**
**4.27**	**(-)-1-(6-(Methylsulfonyl)pyridin-3-yl)ethan-1-ol**	**7.69**

(-)-5-hydroxy-5,6,7,8-tetrahydroquinoline (*R*)-**4**

Method: Separation was performed on a Daicel Chiralpak(R) AD-H HPLC column (150 x 4.60 mm, 5 µm). 10 µL injection from 90/10 *n*-Hex/IPA solution (1 mg/mL), 25 °C column temperature, 1.0 mL/min flow, eluent: IPA/n-hexane 5/95, detection: 230 nm.

Absolute stereochemistry was assigned according to the known optical rotation of the (*R*)-enantiomer.[70]

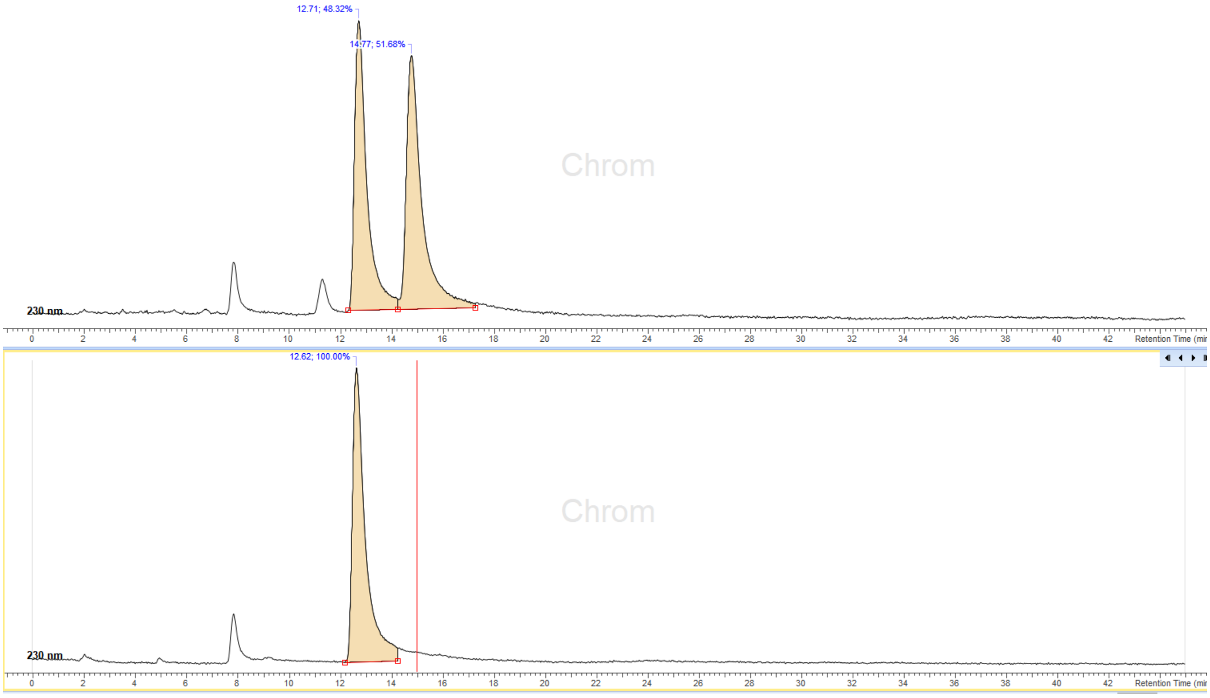

Retention time	Compound	%
**12.7**	**(*R*)-5-hydroxy-5,6,7,8-tetrahydroquinoline**	**>99**
**14.8**	**(*S*)-5-hydroxy-5,6,7,8-tetrahydroquinoline**	**n.d.**

3-Hydroxy-3-Methyloxindole **91**

Method: Separation was performed on a Daicel Chiralcel[(R)] OD-H HPLC column (150 mm x 4.60 mm, 5.0 µm). Samples were injected from 85/15 *n*-Hex/IPA solution. 10.0 µL injection volume, 25 °C column temperature, 1.0 mL/min flow, eluent: IPA/*n*-Hex 15/85, detection: 220 nm.

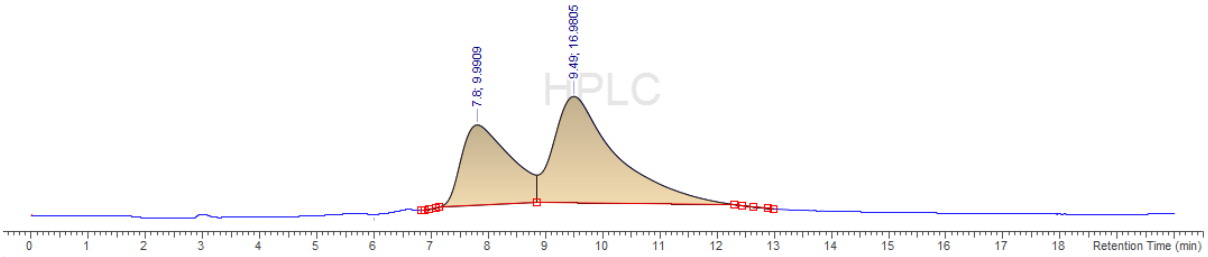

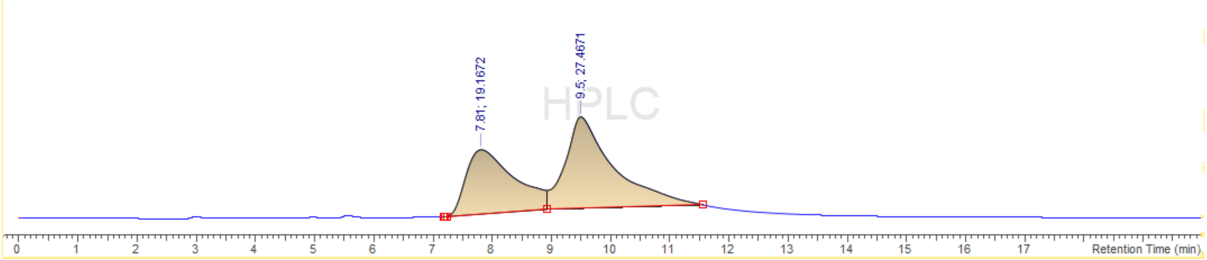

Retention time	Compound	%
**7.80**	**(*?*)-3-Hydroxy-3-Methyloxindole**	**50.0**
**9.49**	**(*?*)-3-Hydroxy-3-Methyloxindole**	**50.0**

14) ^{1}H and ^{13}C NMR Spectra

NMR Synthesis of Authentic Standards and Starting Materials

*6,7-Dihydro-5*H*-cyclopenta[*b*]pyridine 1-oxide* **S1**

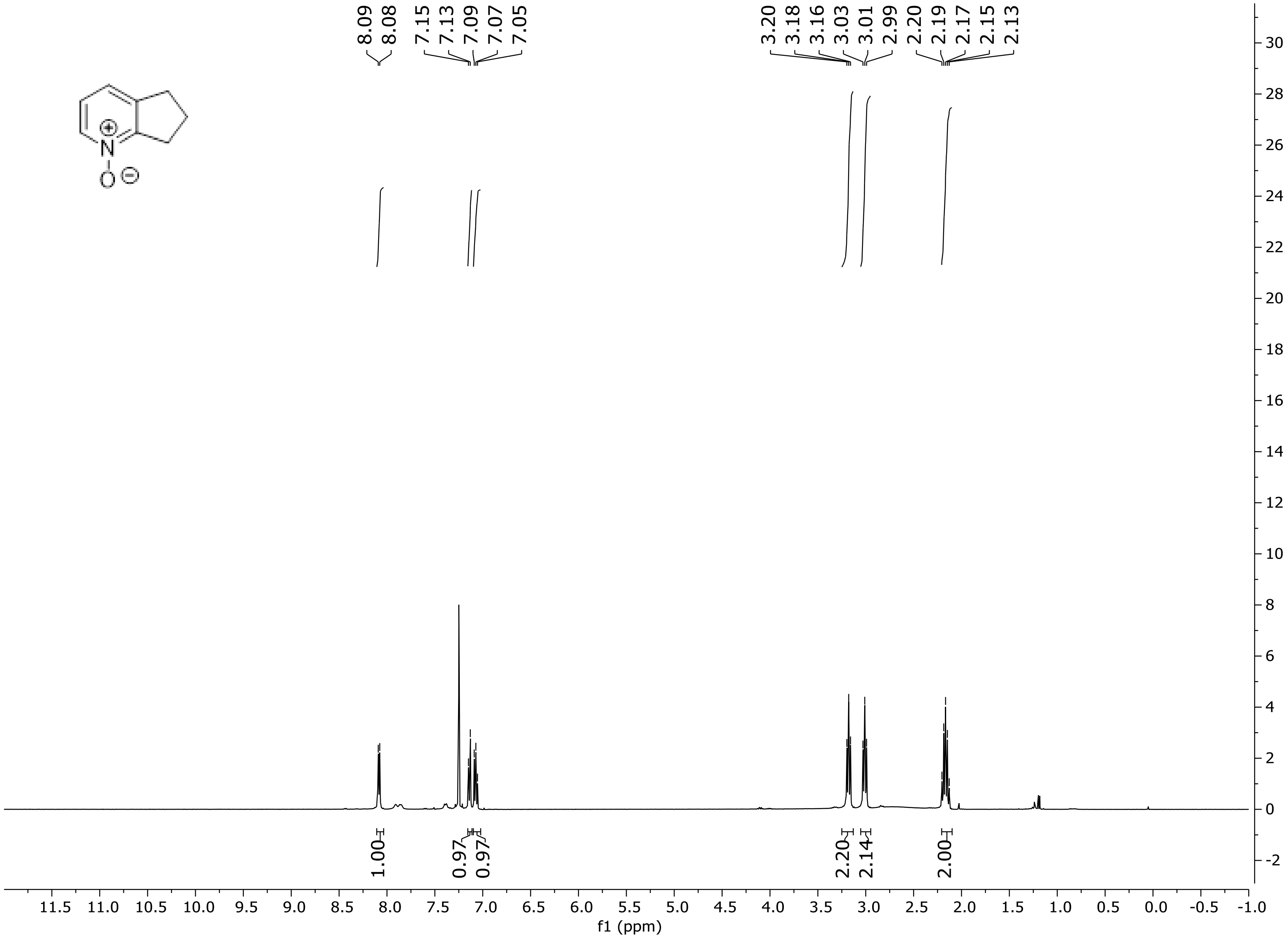

Figure S2. ^{1}H NMR (400 MHz, $CDCl_3$, 298K) of .**S1**.

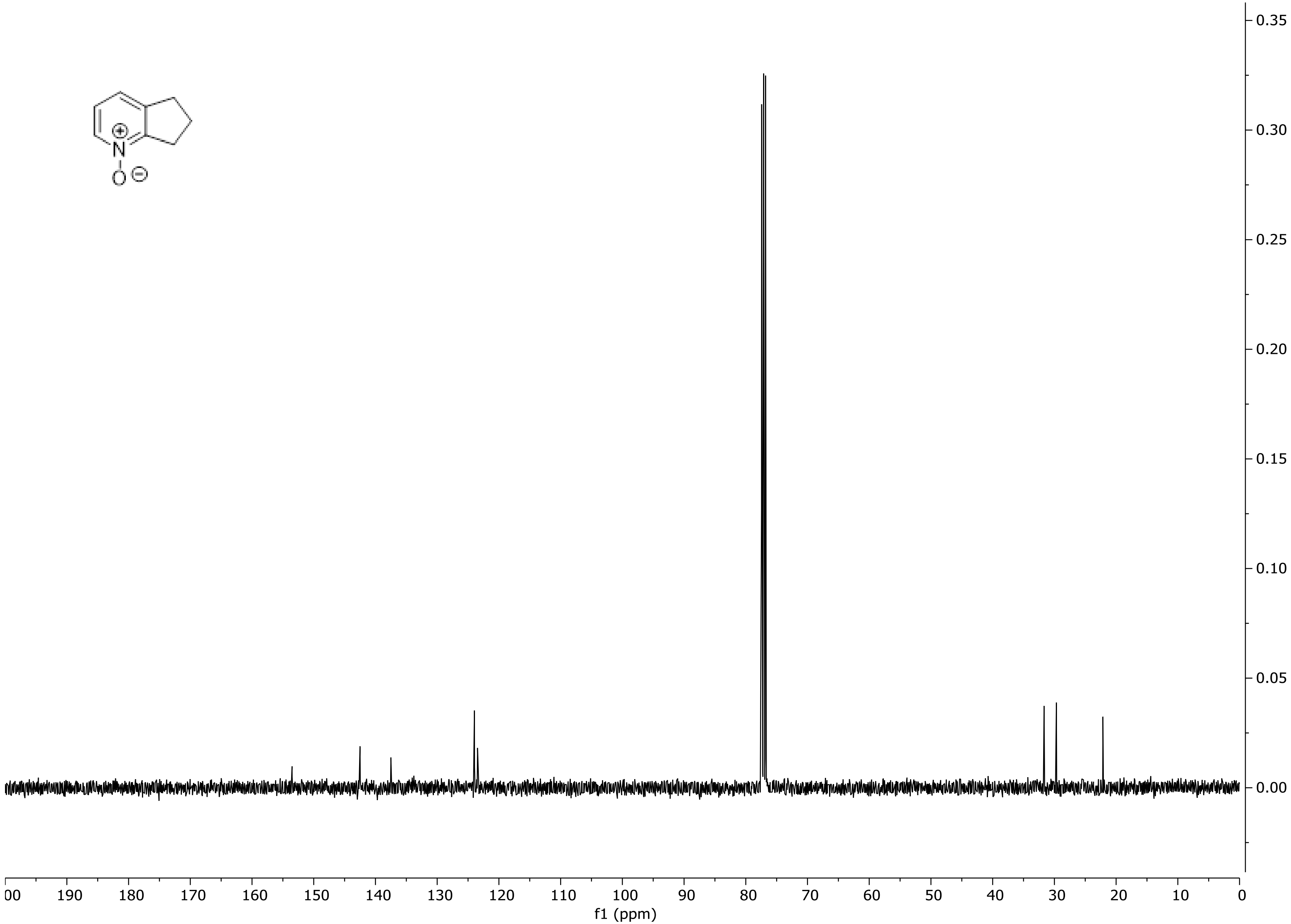

Figure S3. ^{13}C NMR (101 MHz, $CDCl_3$, 298K) of .**S1**.

*6,7-Dihydro-5*H*-cyclopenta[b]pyridin-7-yl trifluoromethanesulfonate* **S2**

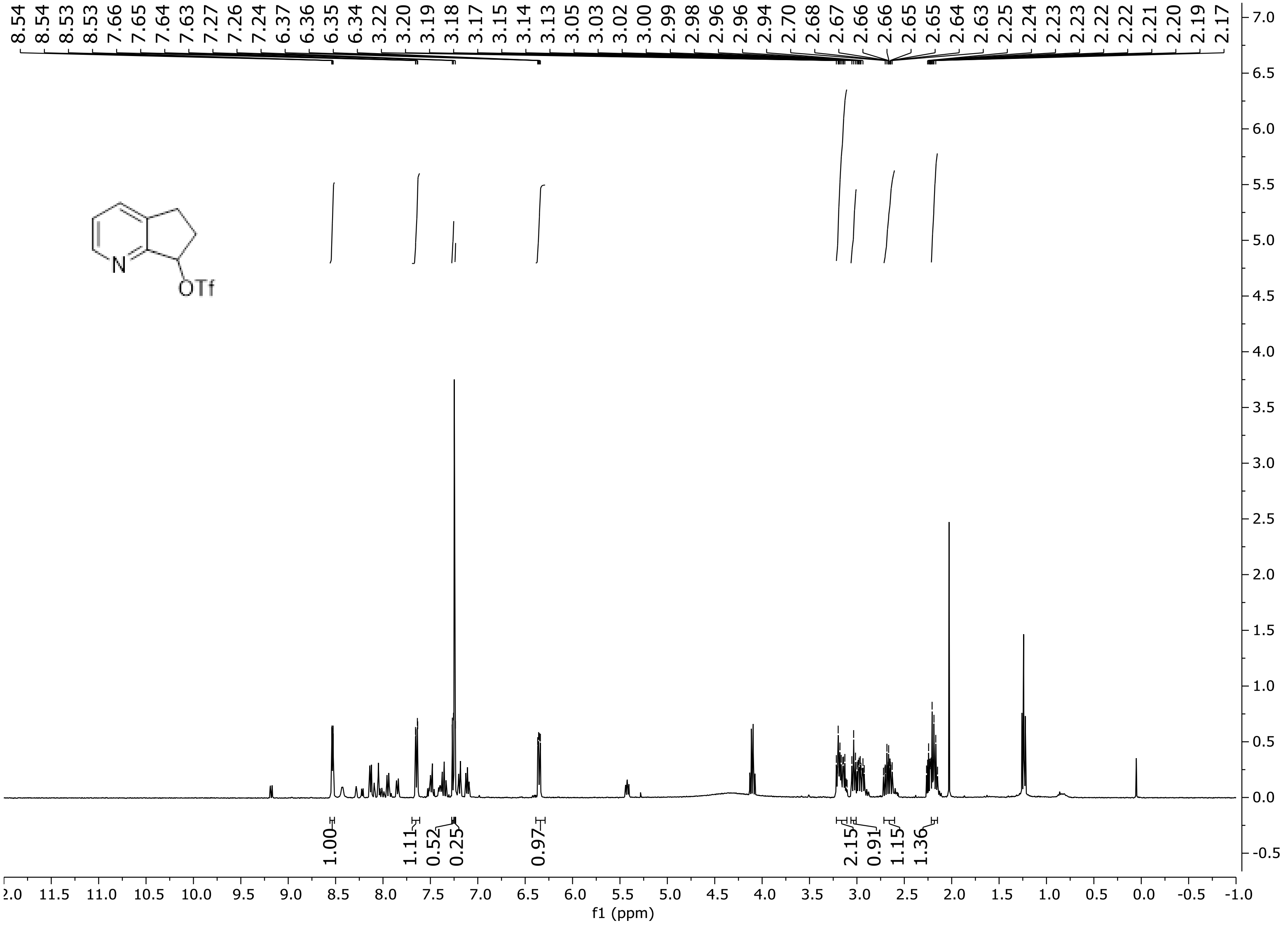

Figure S4. ^{1}H NMR (400 MHz, $CDCl_3$, 298K) of .**S2**.

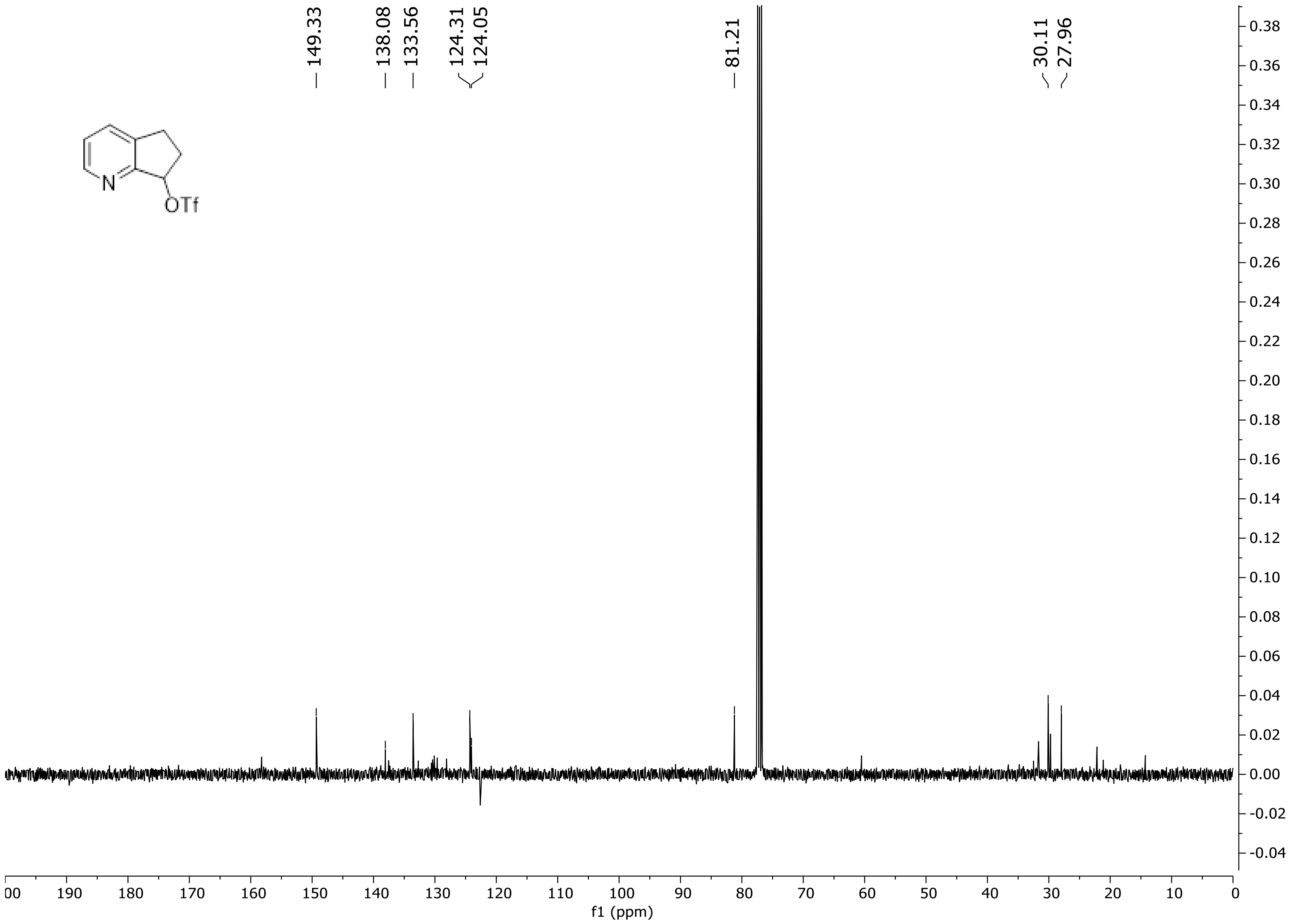

Figure S5. ^{13}C NMR (101 MHz, $CDCl_3$, 298K) of .**S2**.

*(+/-)-6,7-dihydro-5*H*-cyclopenta[b]pyridin-7-ol rac-***55**

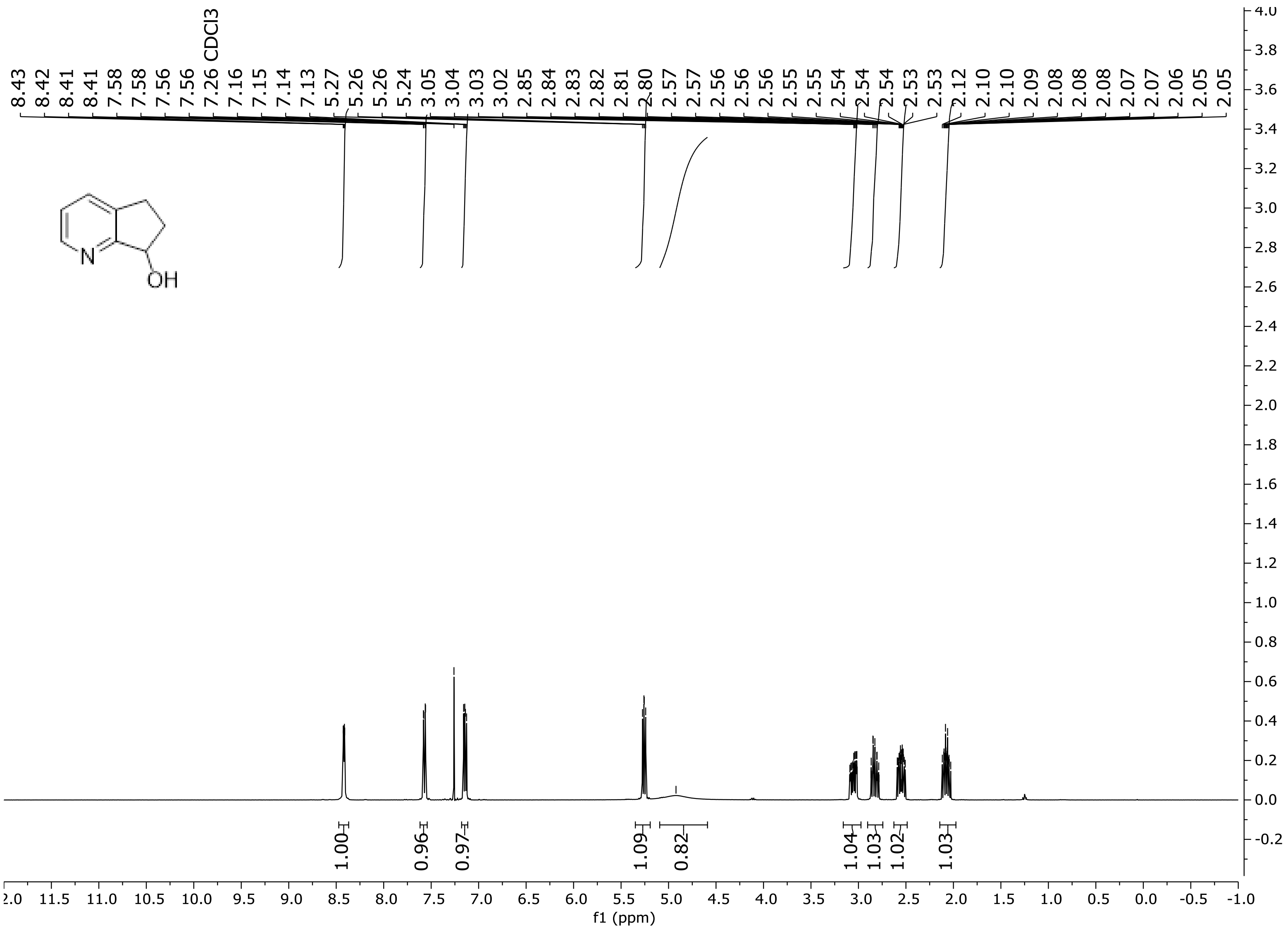

Figure S6. ^{1}H NMR (400 MHz, $CDCl_3$, 298K) of .*rac*-**55**.

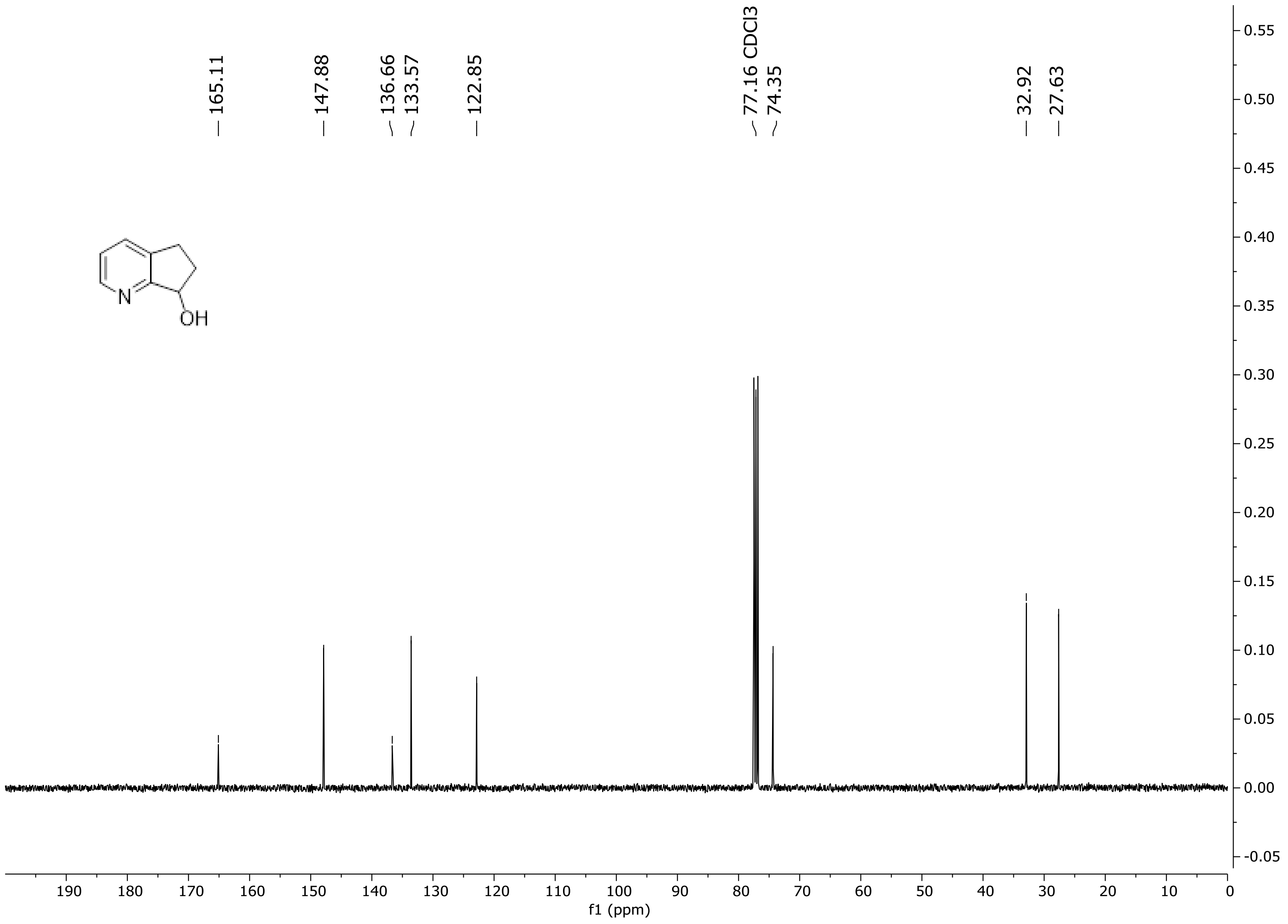

Figure S7. ^{13}C NMR (101 MHz, $CDCl_3$, 298K) of .*rac*-**55**.

3-Propylpyridine **11**

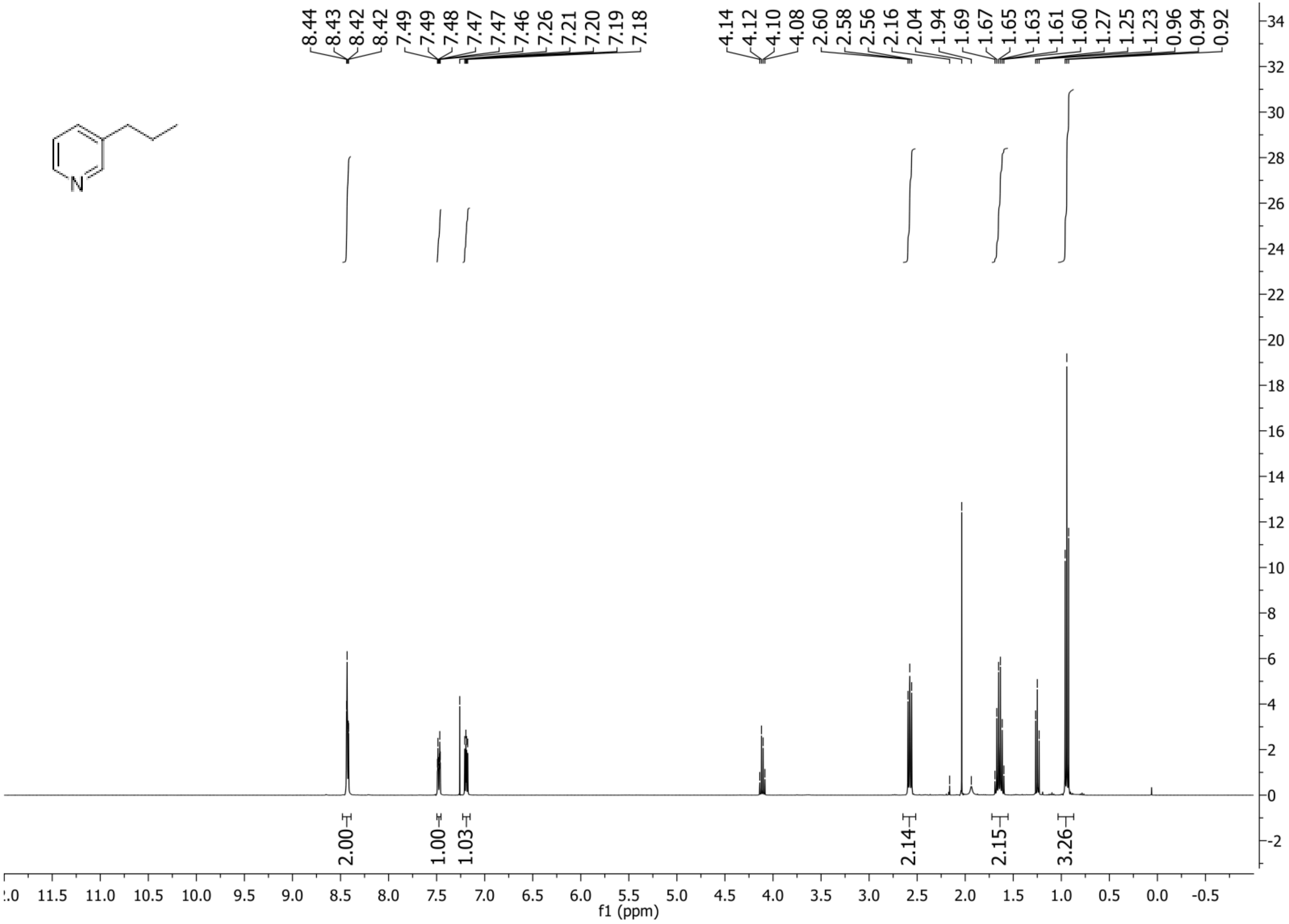

Figure S8. ^{1}H NMR (400 MHz, $CDCl_3$, 298K) of .**11**.

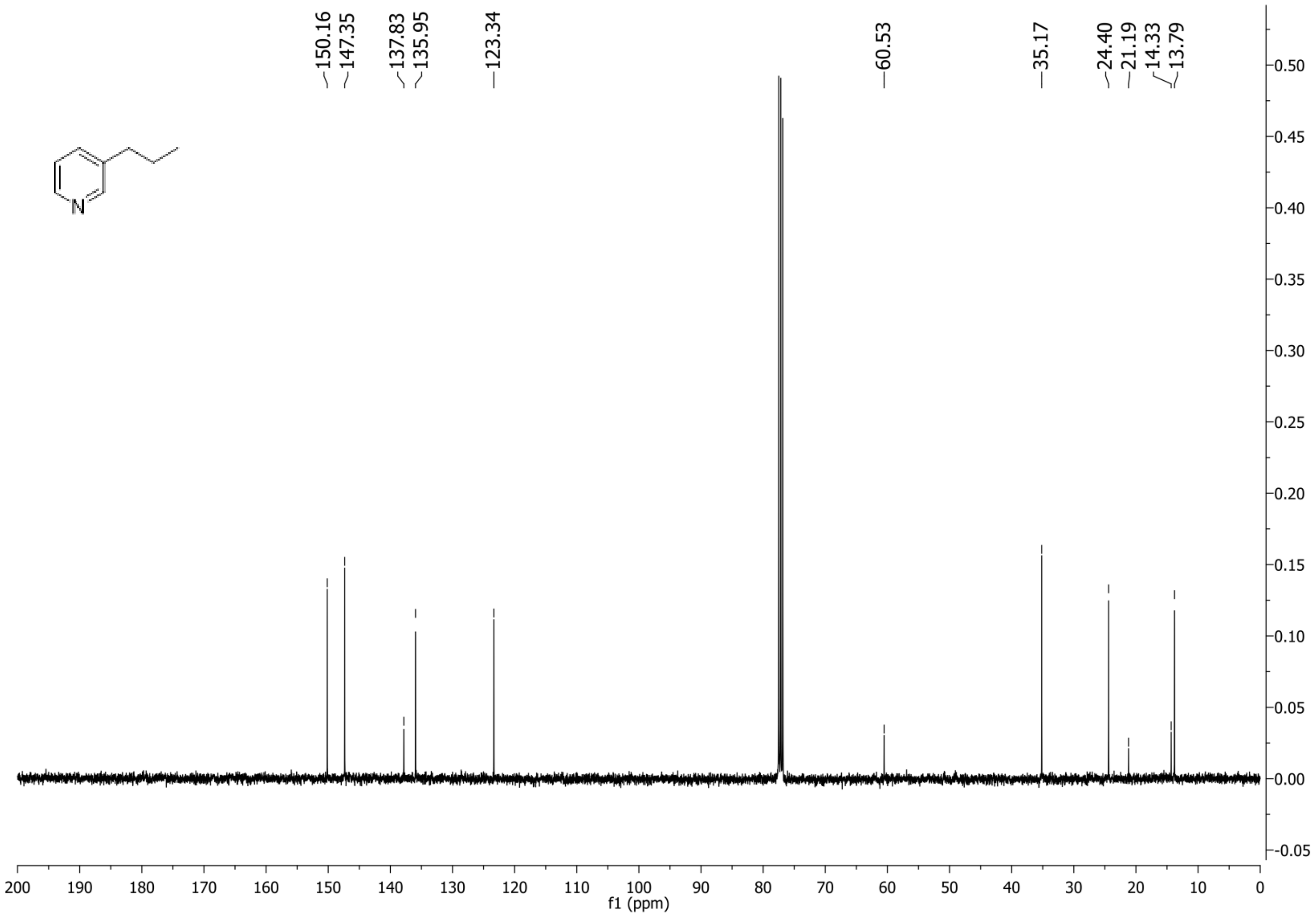

Figure S9. ^{13}C NMR (101 MHz, $CDCl_3$, 298K) of .**11**.

3-Isopropylpyridine **12**

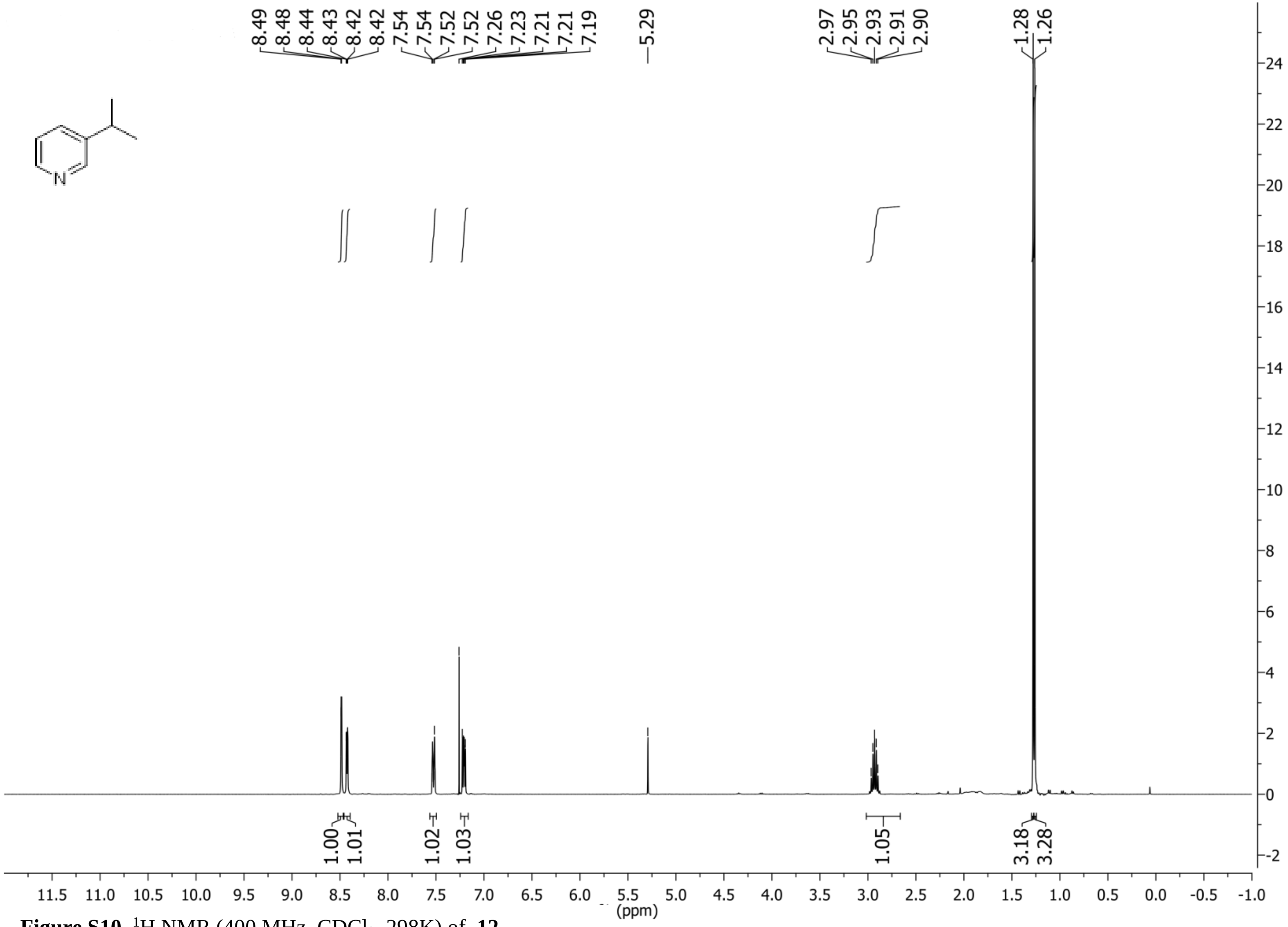

Figure S10. ^{1}H NMR (400 MHz, $CDCl_3$, 298K) of .**12**.

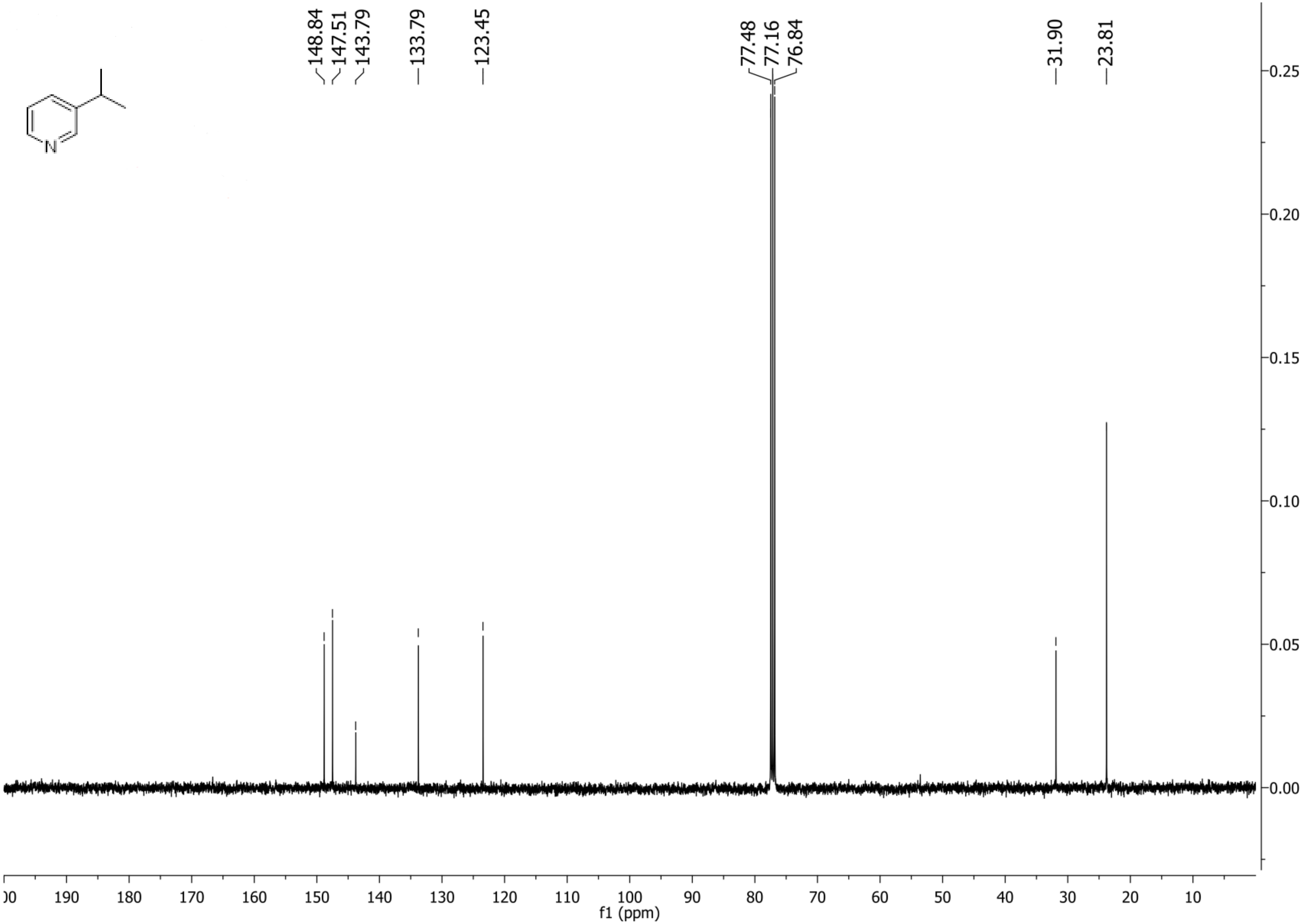

Figure S11. ^{1}H NMR (400 MHz, $CDCl_3$, 298K) of .**12**.

*(+/-)-1-(pyridine-3-yl)-ethan-1-ol rac-***20**

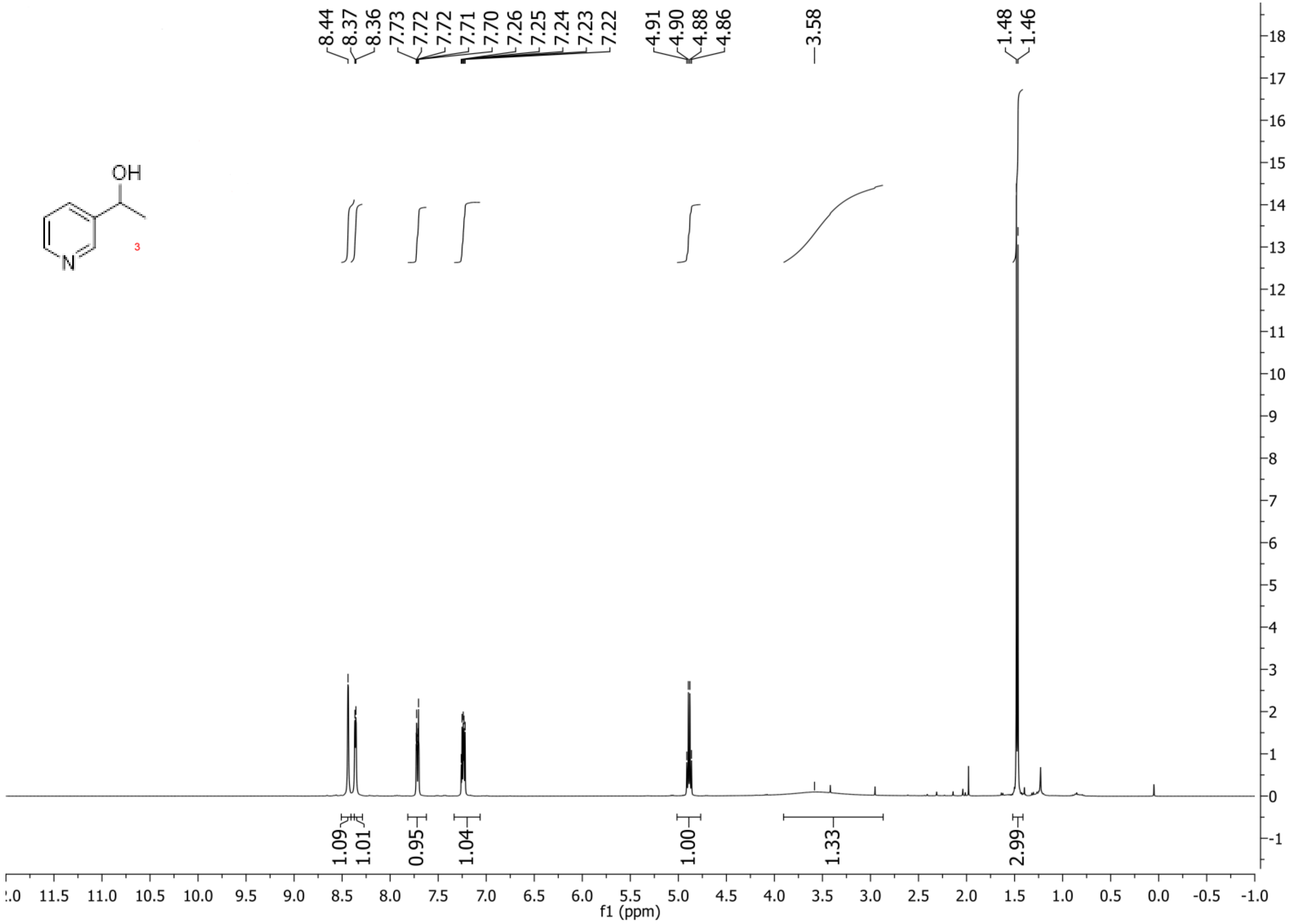

Figure S12. ^{1}H NMR (400 MHz, $CDCl_3$, 298K) of .**20**.

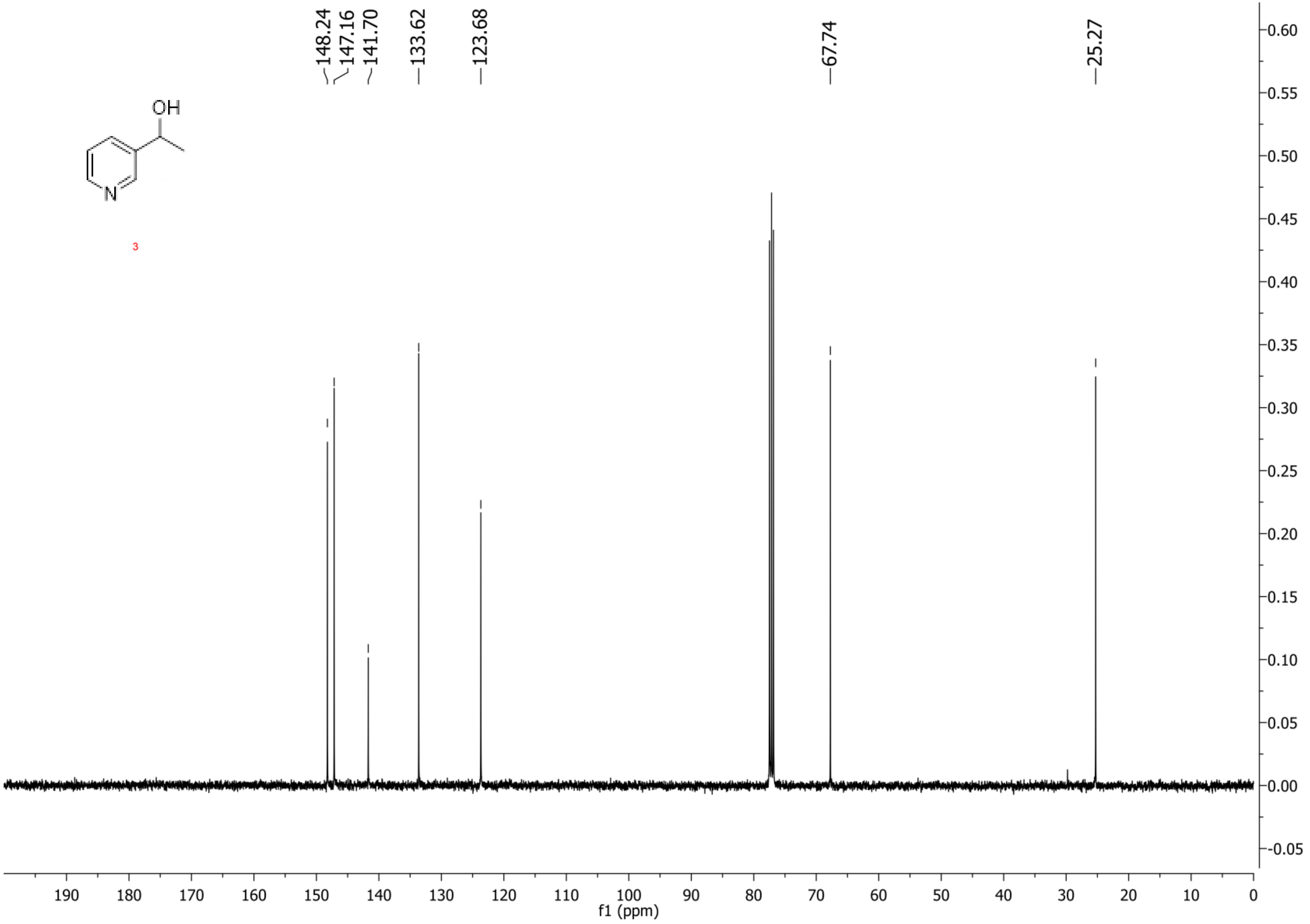

Figure S13. ^{13}C NMR (101 MHz, $CDCl_3$, 298K) of .**20**.

*(+/-)-(Pyridine-2-yl)-ethan-1-ol rac-***22**

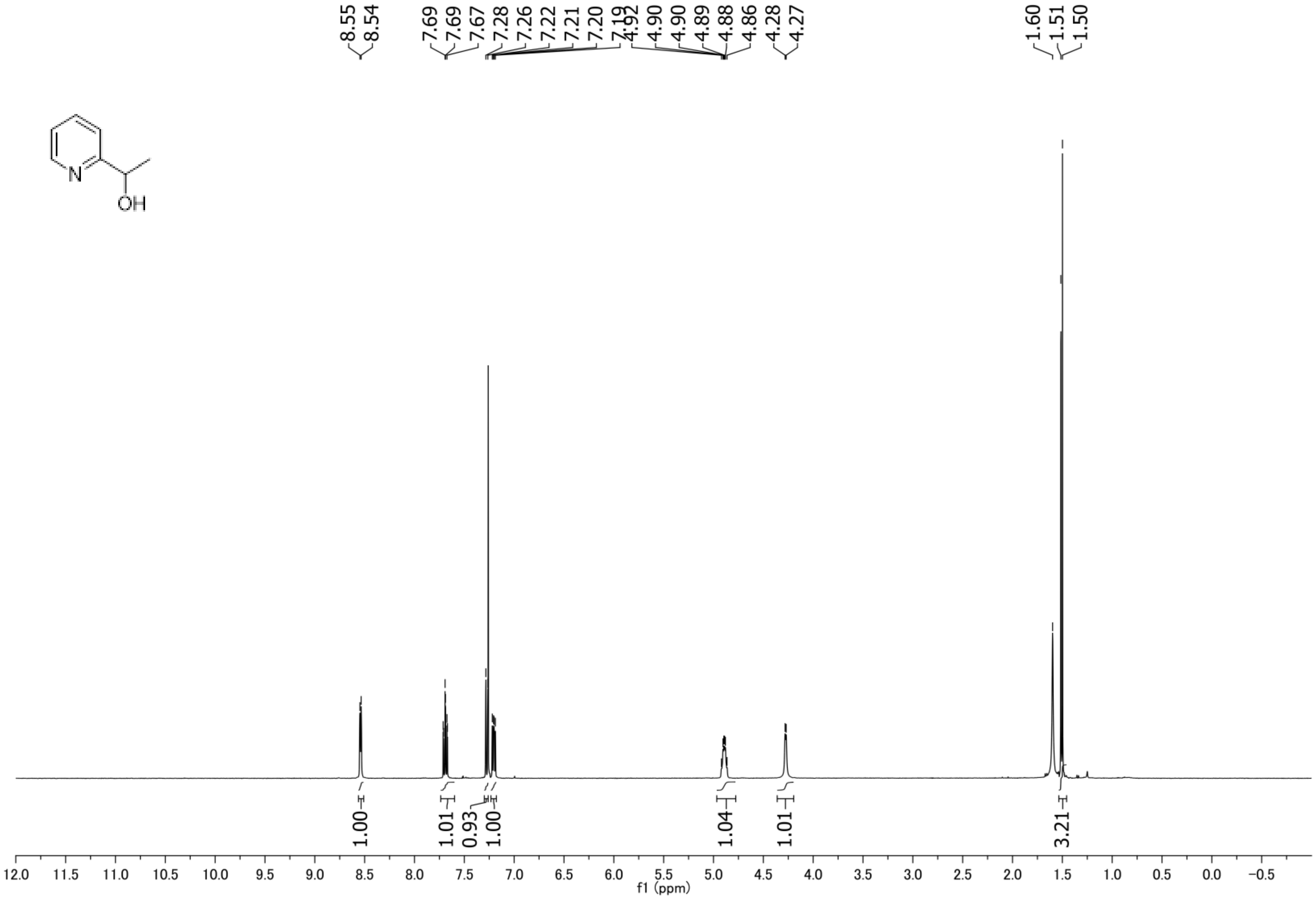

Figure S14. ^{1}H NMR (400 MHz, $CDCl_3$, 298K) of .*rac*-**22**.

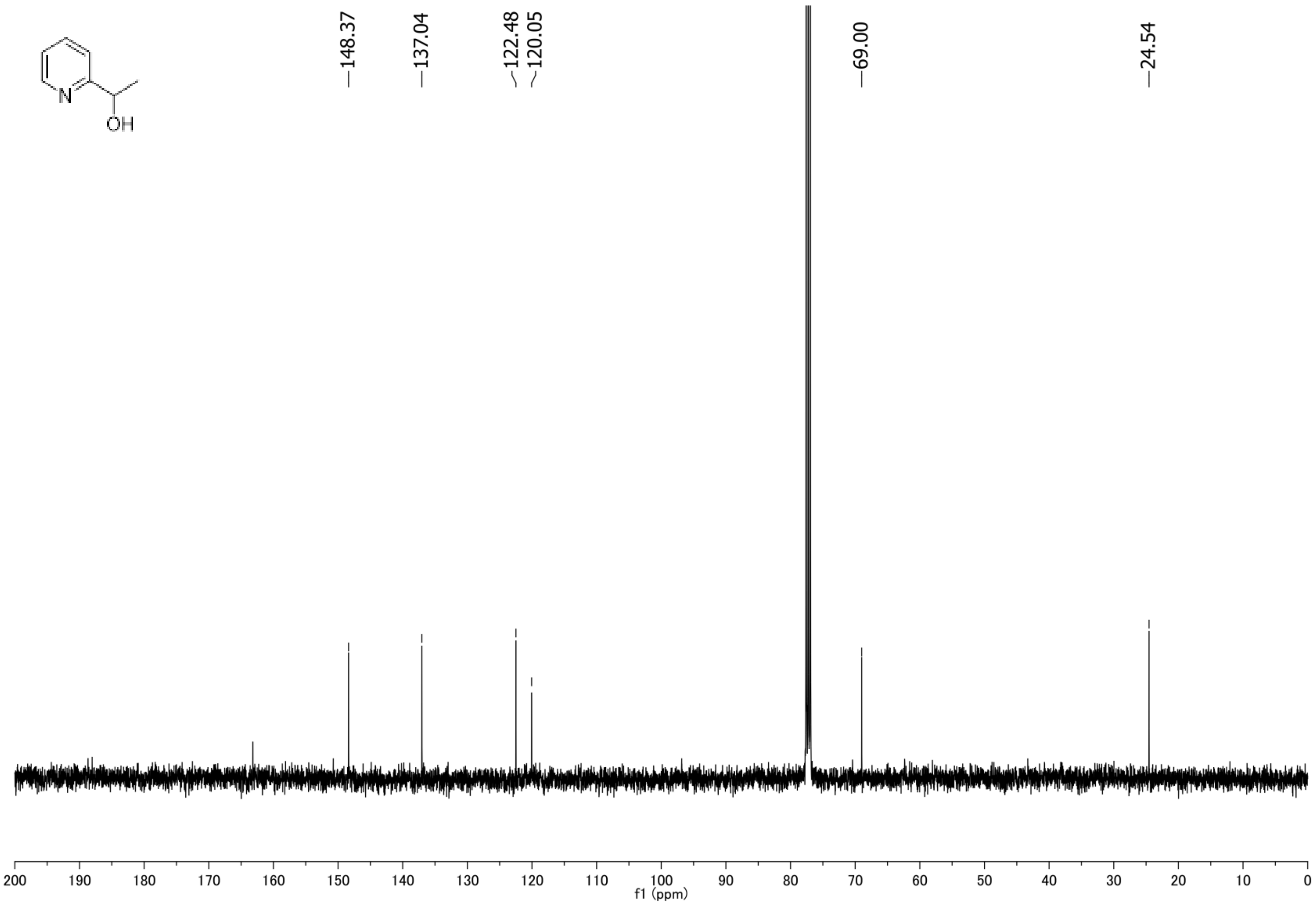

Figure S15. ^{13}C NMR (101 MHz, $CDCl_3$, 298K) of .*rac*-**22**.

2-(Pyridin-3-yl)-propan-2-ol **24**

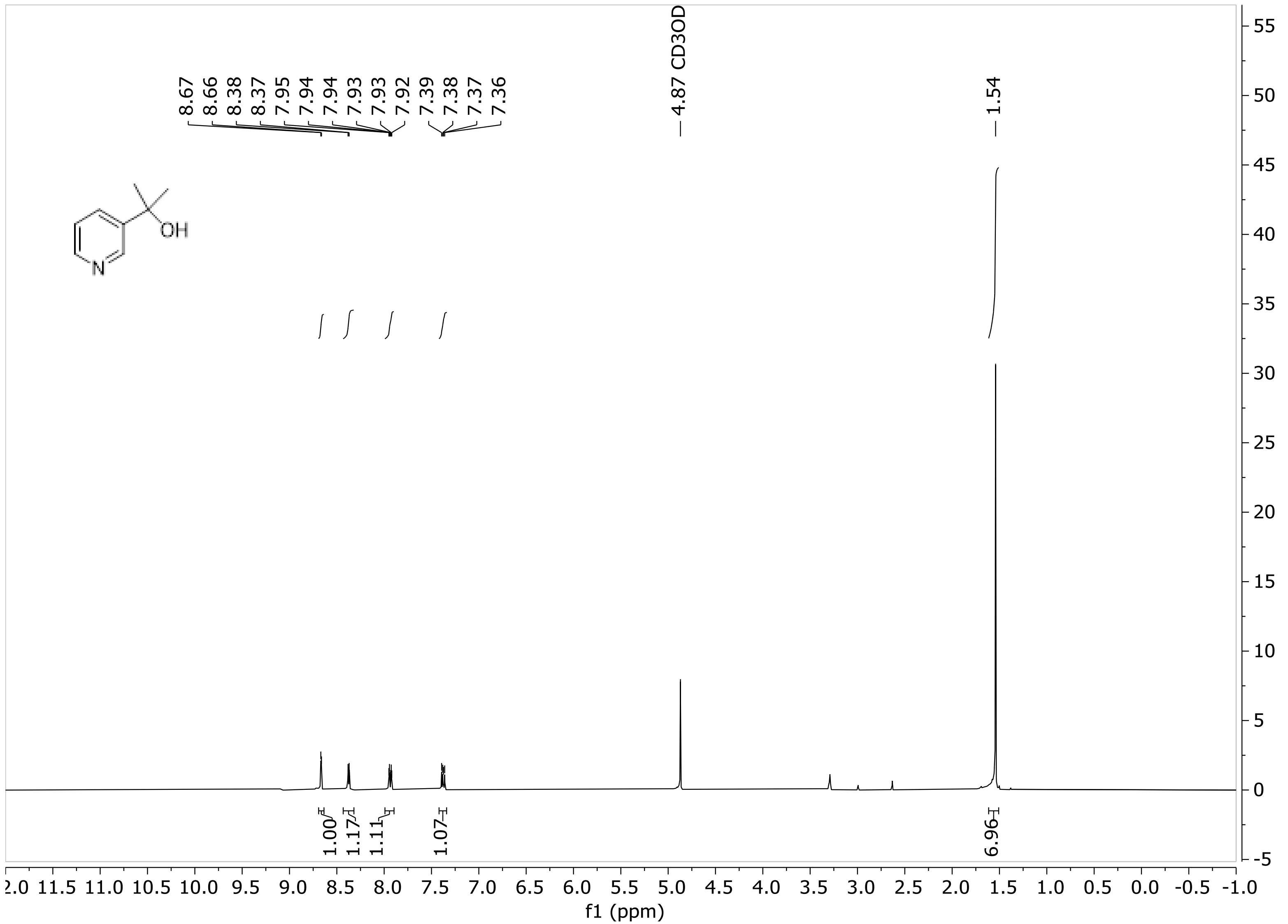

Figure S16. ^{1}H NMR (400 MHz, MeOH-d_4, 298K) of .**24**.

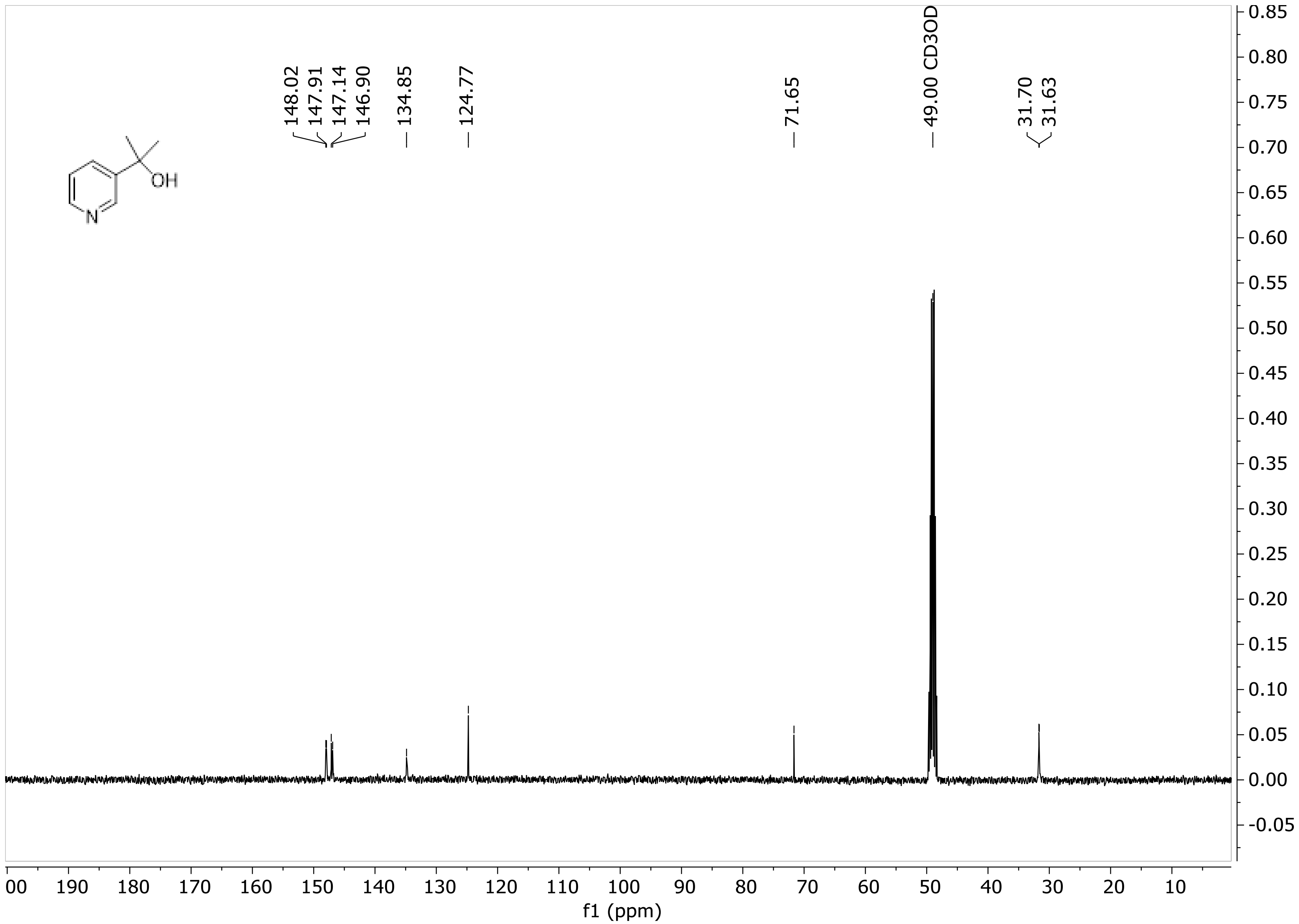

Figure S17. ^{13}C NMR (101 MHz, MeOH-d_4, 298K) of .**24**.

2-(Pyridin-2-yl)-propan-2-ol **25**

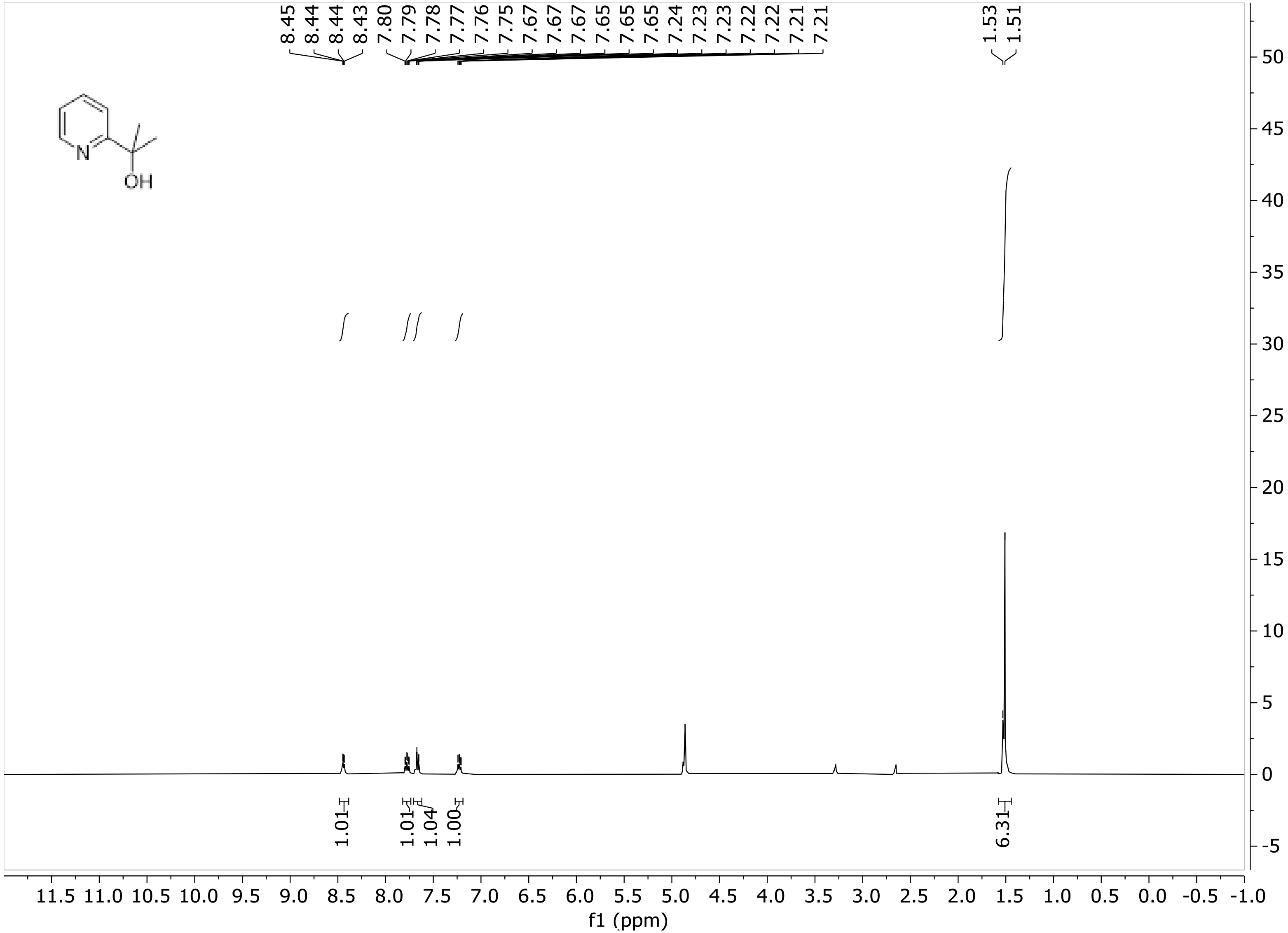

Figure S18. ^{1}H NMR (400 MHz, MeOH-d_3, 298K) of .**25**.

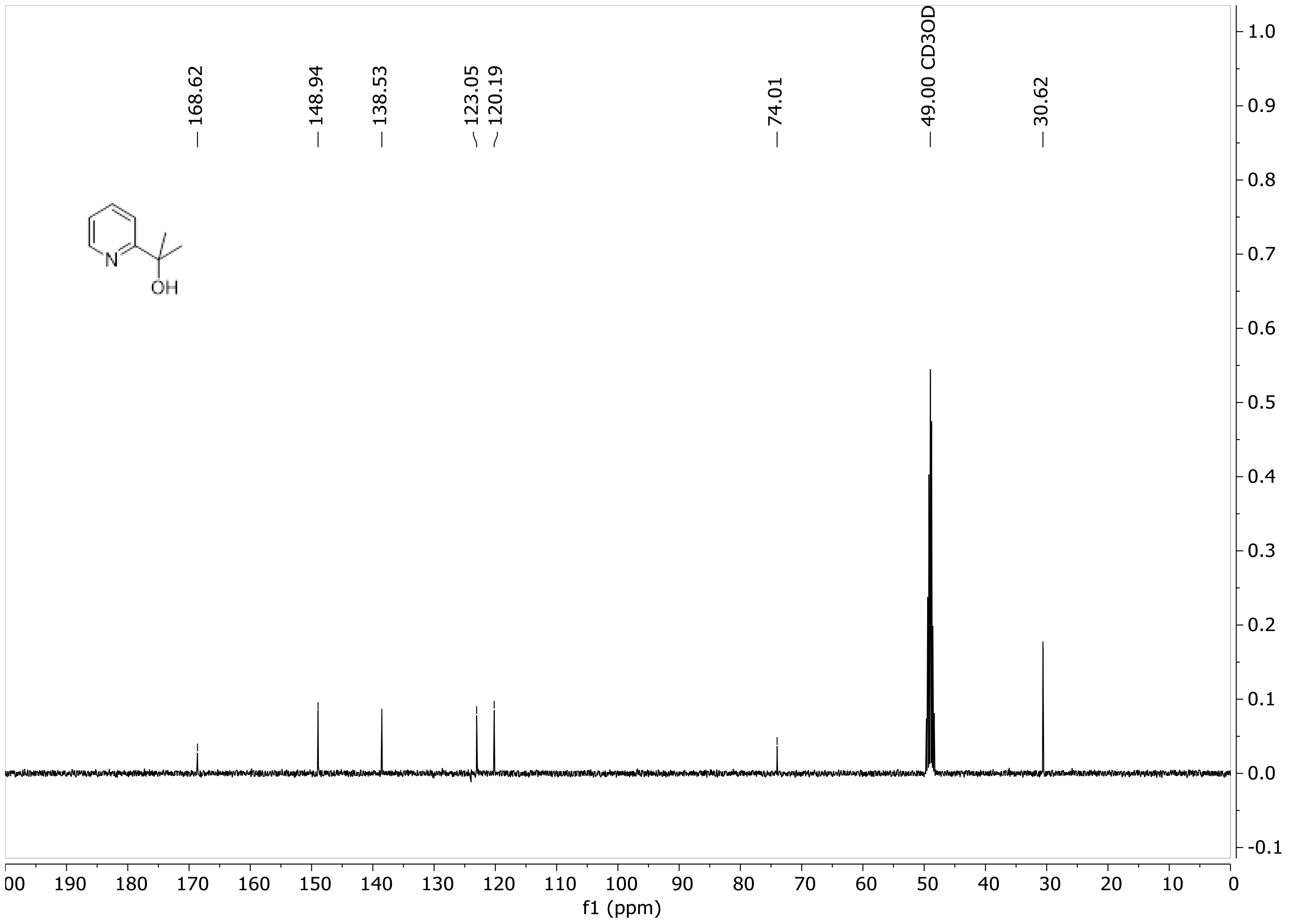

Figure S19. ^{13}C NMR (101 MHz, MeOH-d_3, 298K) of .**25**.

*(+/-)-1-(Pyridine-2-yl)-propan-1-ol rac-***27**

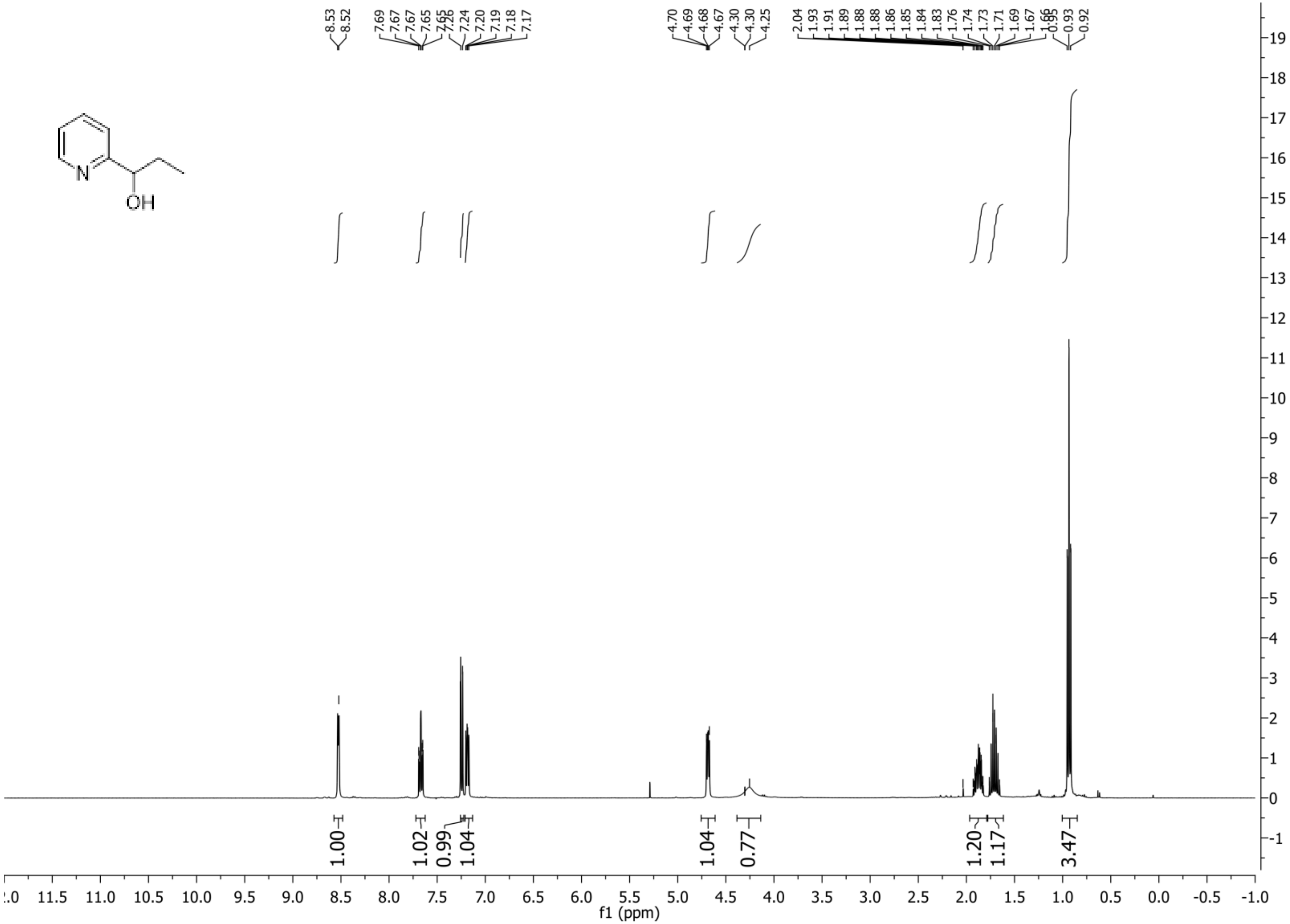

Figure S20. ^{1}H NMR (400 MHz, $CDCl_3$, 298K) of .*rac*-**27**.

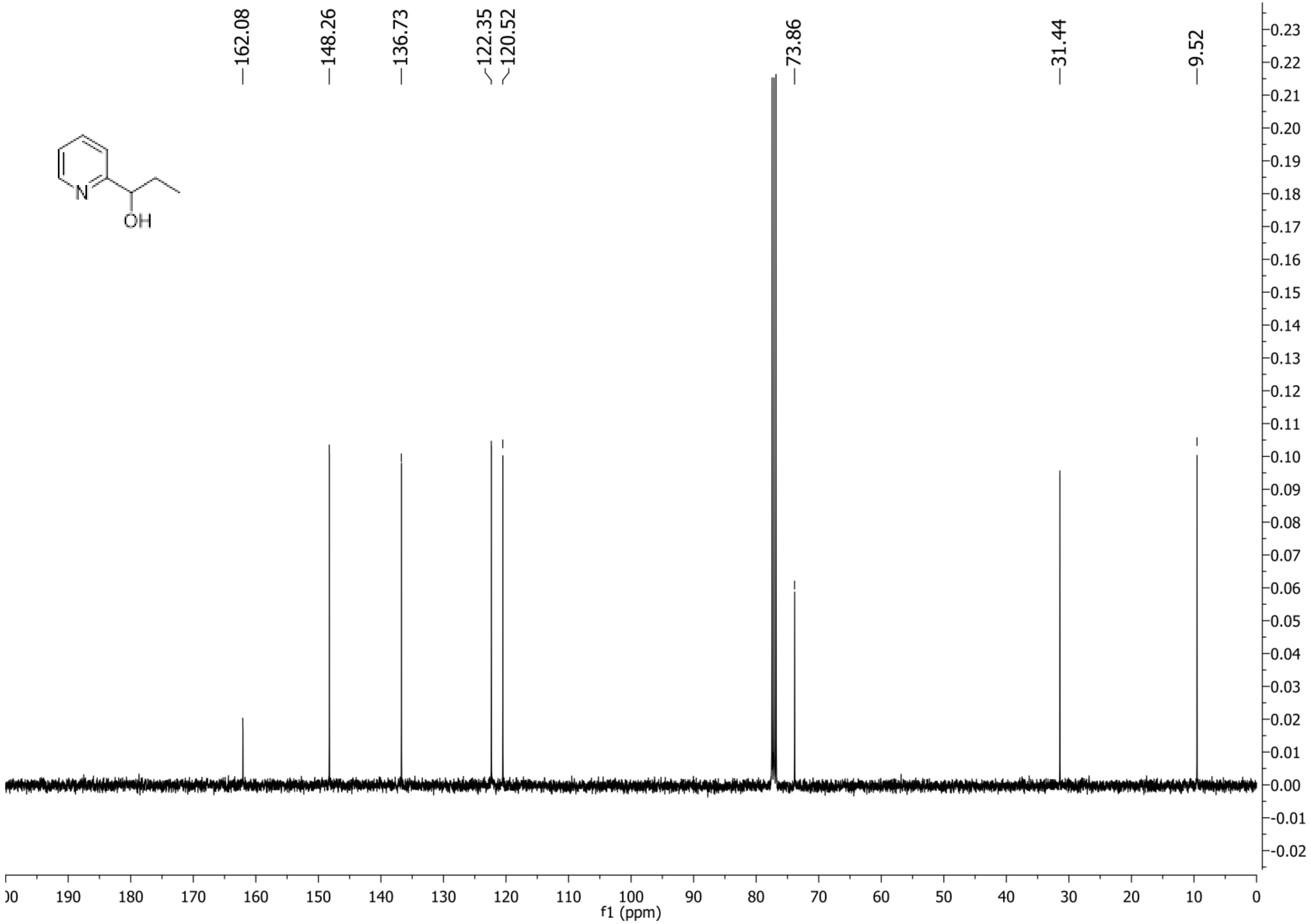

Figure S21. ^{13}C NMR (101 MHz, $CDCl_3$, 298K) of .*rac*-**27**.

2-Methylquinoline 1-oxide **S3**

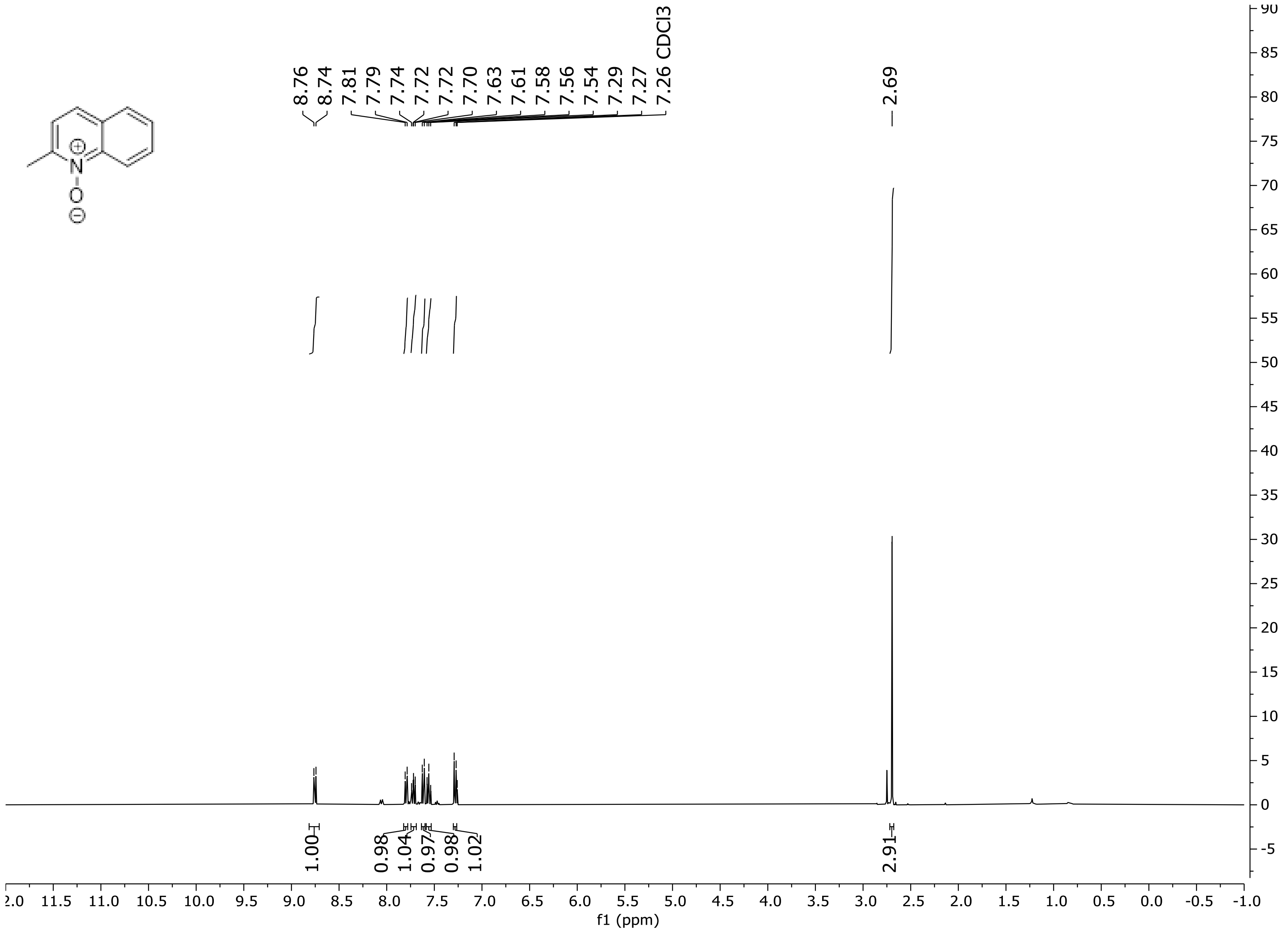

Figure S22. ^{1}H NMR (400 MHz, $CDCl_3$, 298K) of **S3**.

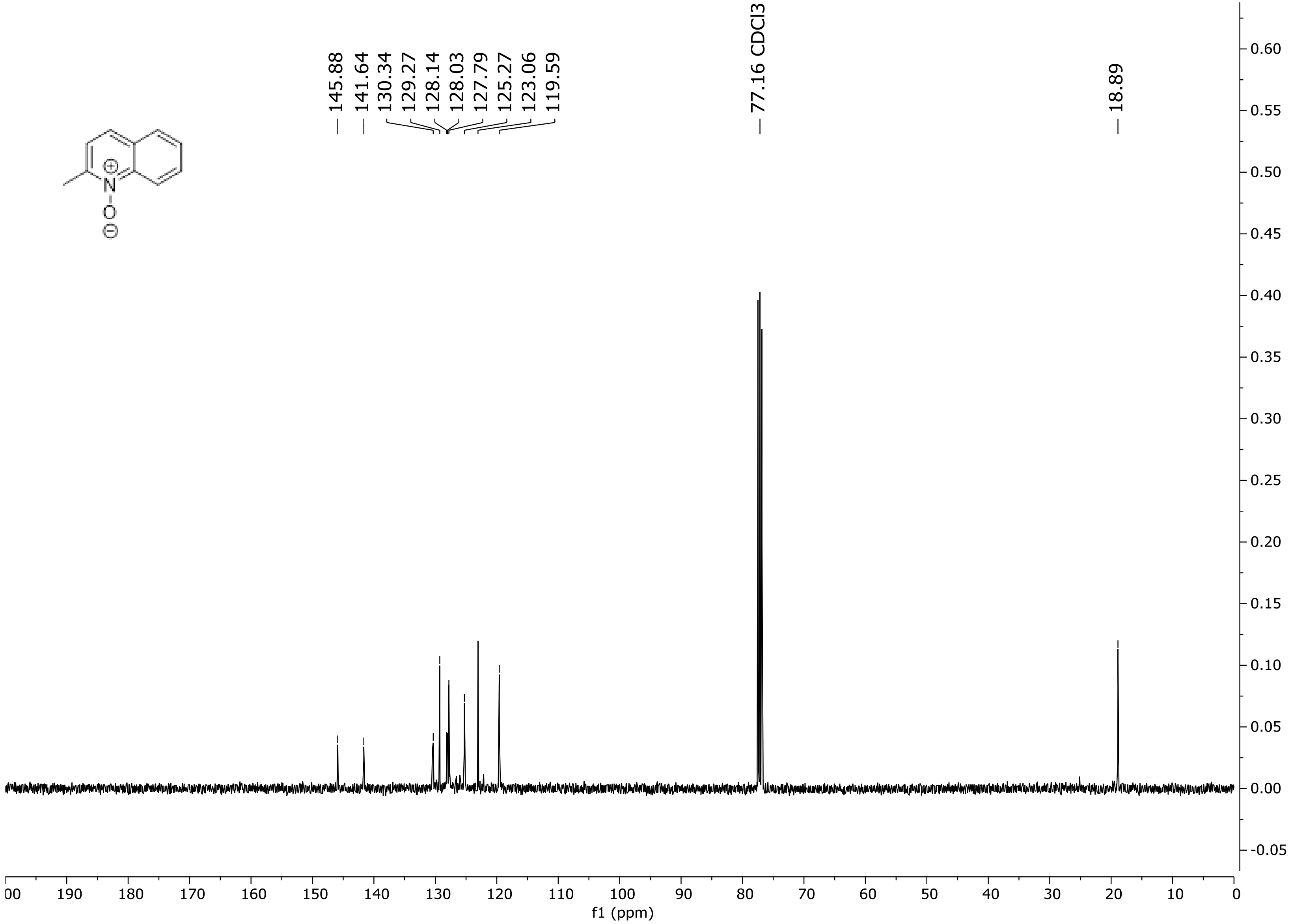

Figure S23. ^{13}C NMR (101 MHz, $CDCl_3$, 298K) of **S3**.

Quinolin-2-carbaldehyde **S4**

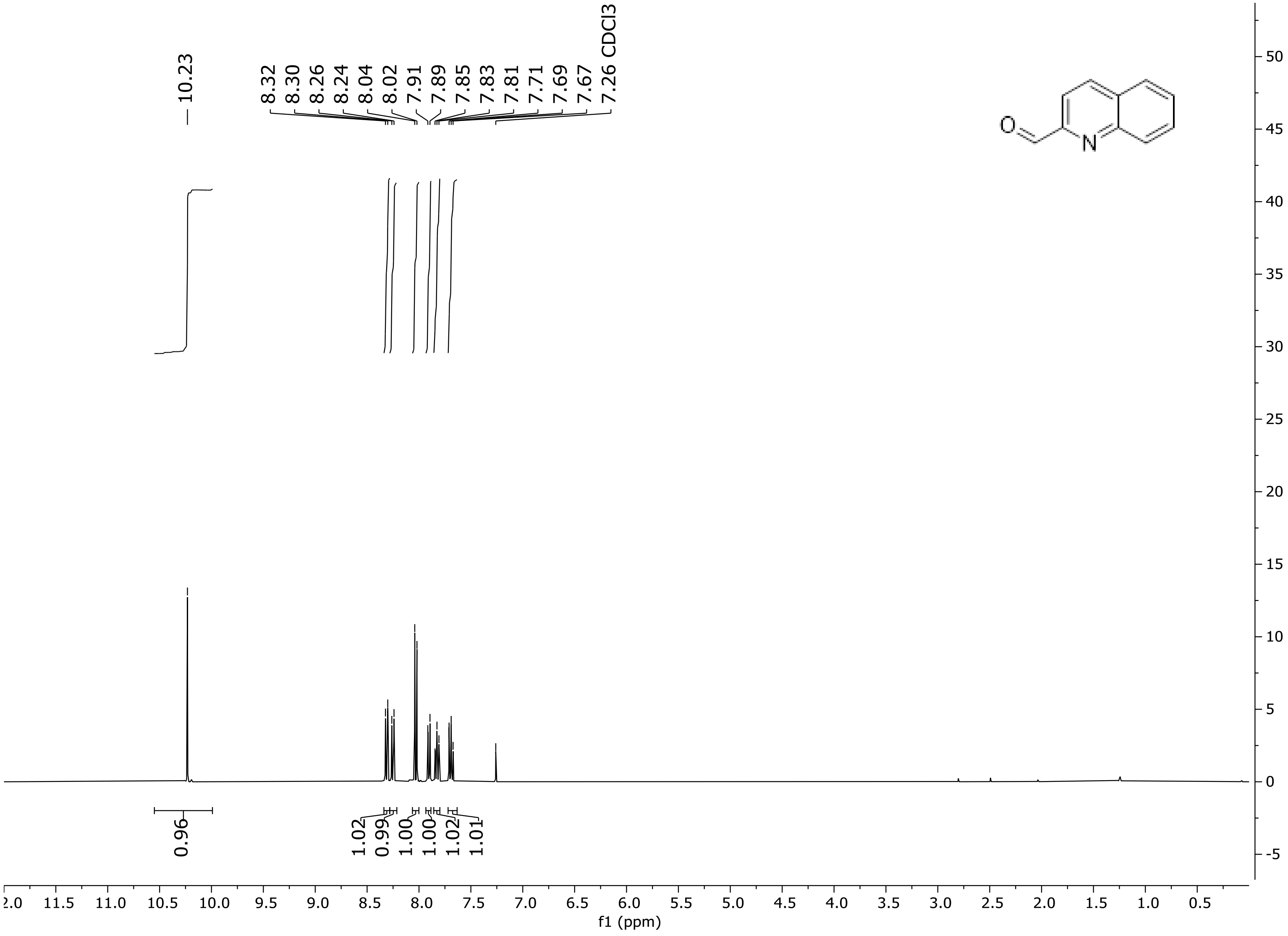

Figure S24. ^{1}H NMR (400 MHz, $CDCl_3$, 298K) of **S4**.

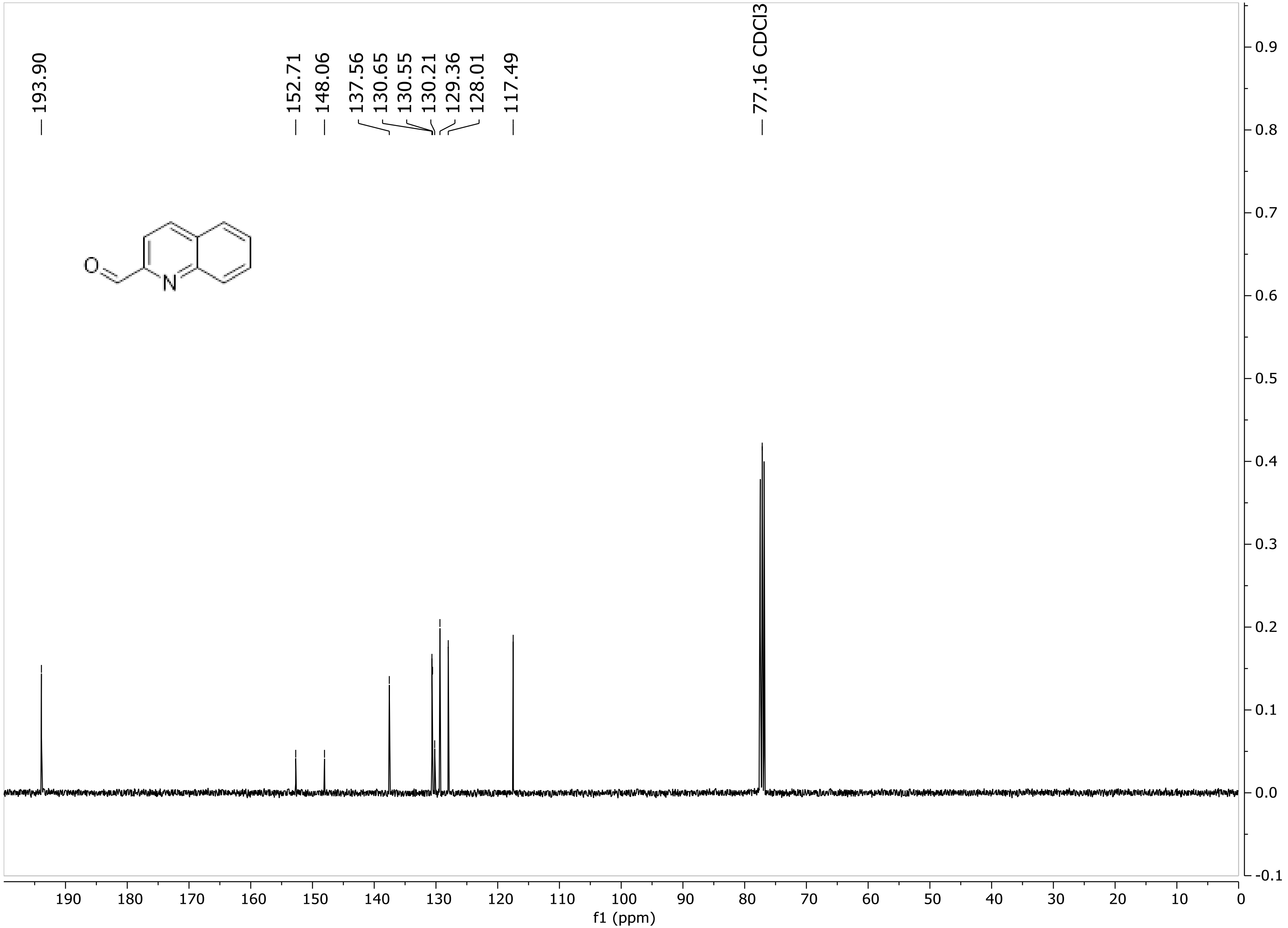

Figure S25. ^{13}C NMR (101 MHz, $CDCl_3$, 298K) of **S4**.

Quinolin-2-ylmethanol **45**

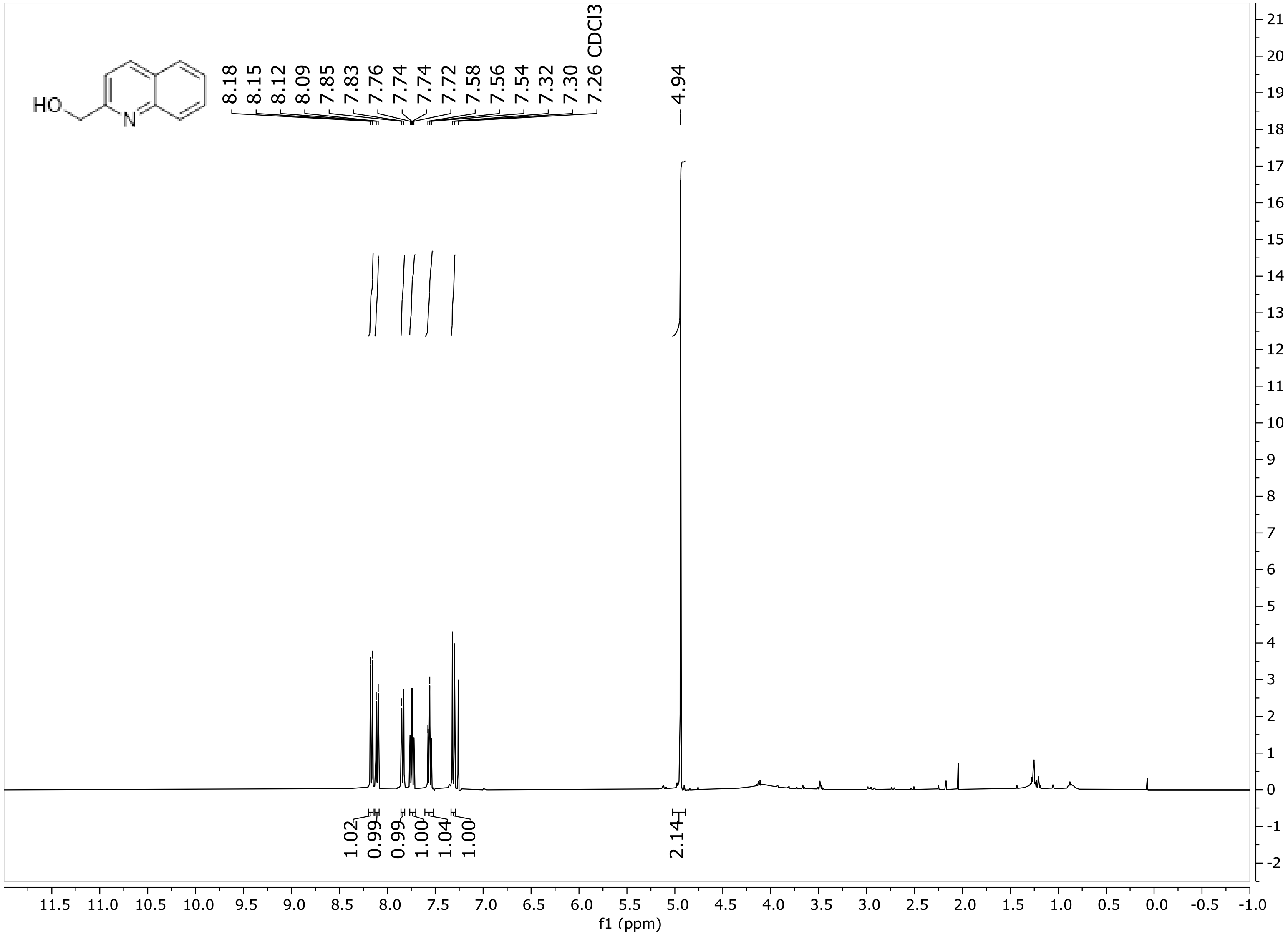

Figure S26. ^{1}H NMR (400 MHz, $CDCl_3$, 298K) of **45**.

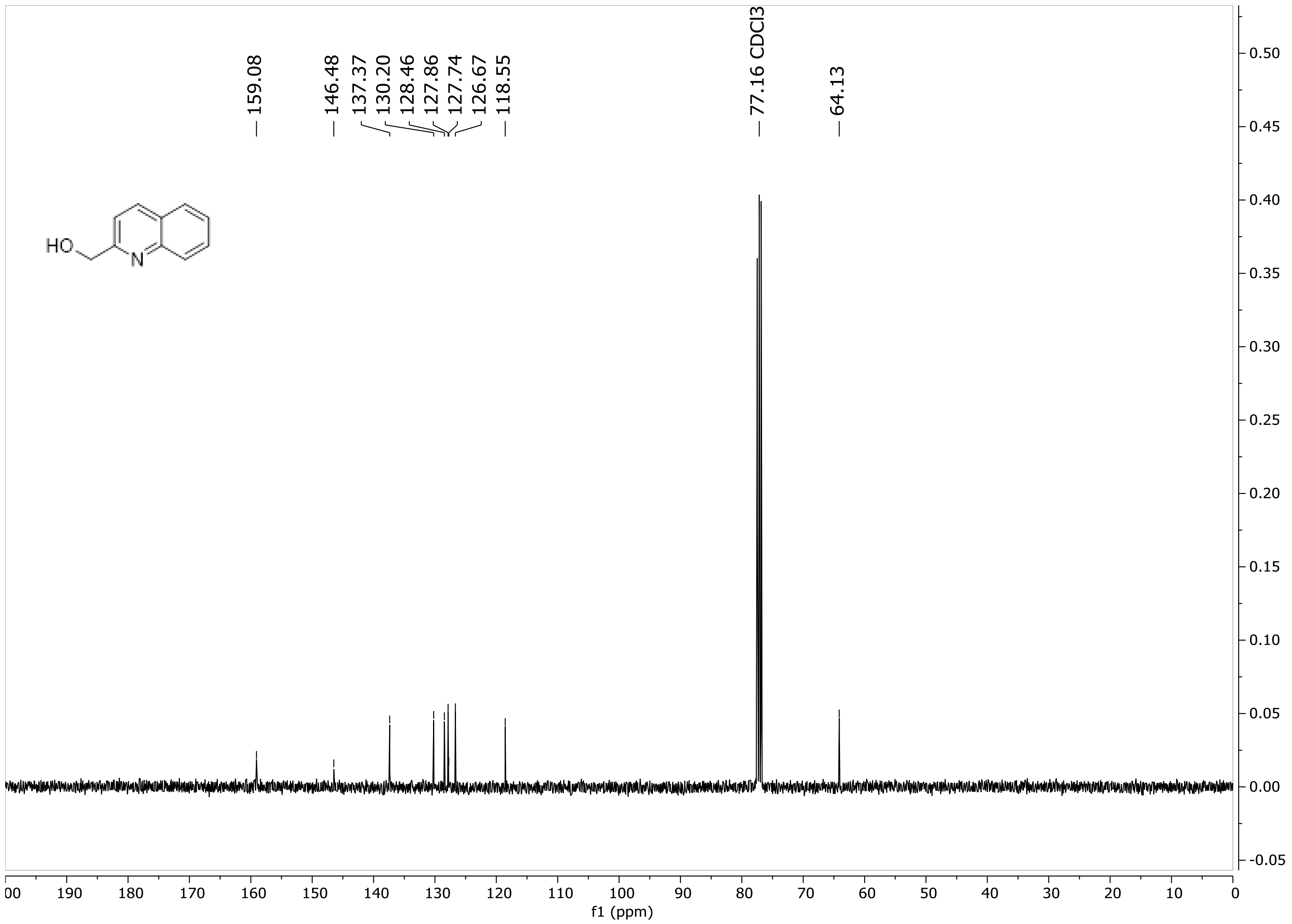

Figure S27. ^{13}C NMR (101 MHz, $CDCl_3$, 298K) of **45**.

6,7-Dihydro-5H-cyclopenta[b]pyridin-5-one **54**

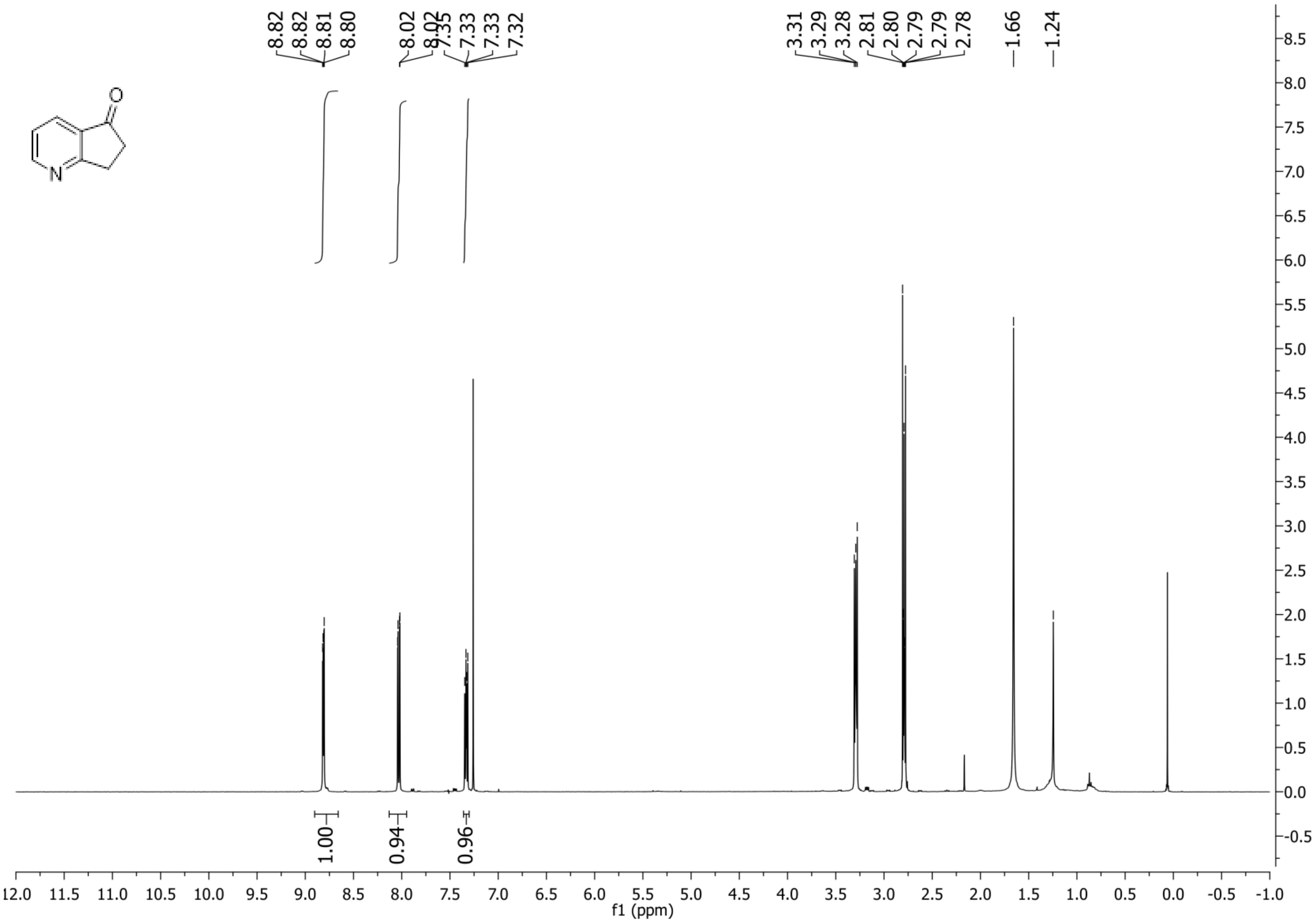

Figure S28. ^{1}H NMR (400 MHz, $CDCl_3$, 298K) of .**54**.

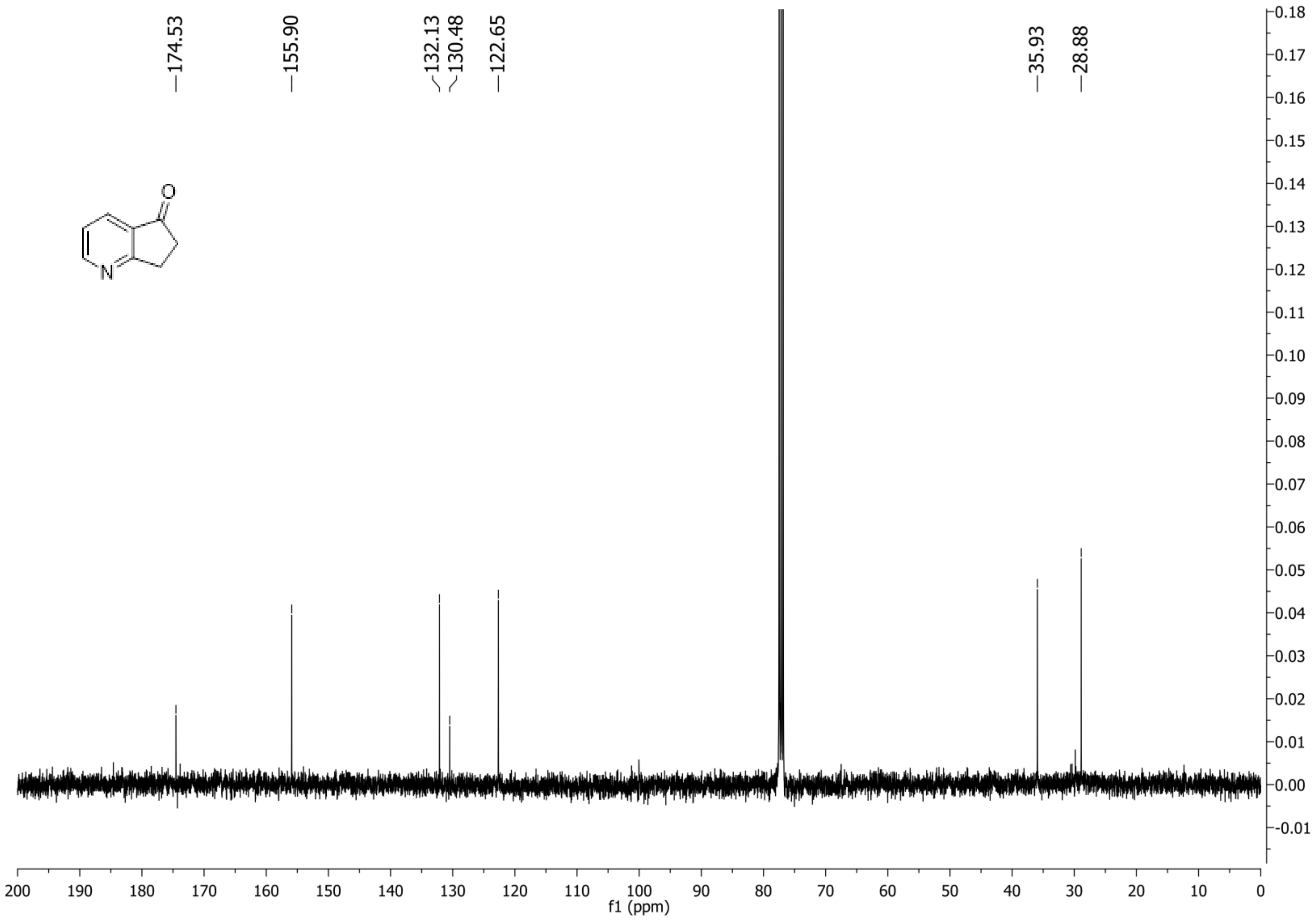

Figure S29. ^{13}C NMR (101 MHz, $CDCl_3$, 298K) of .**54**.

*(+/-)-6,7-Dihydro-5H-cyclopenta[b]pyridin-5-ol rac-***2**

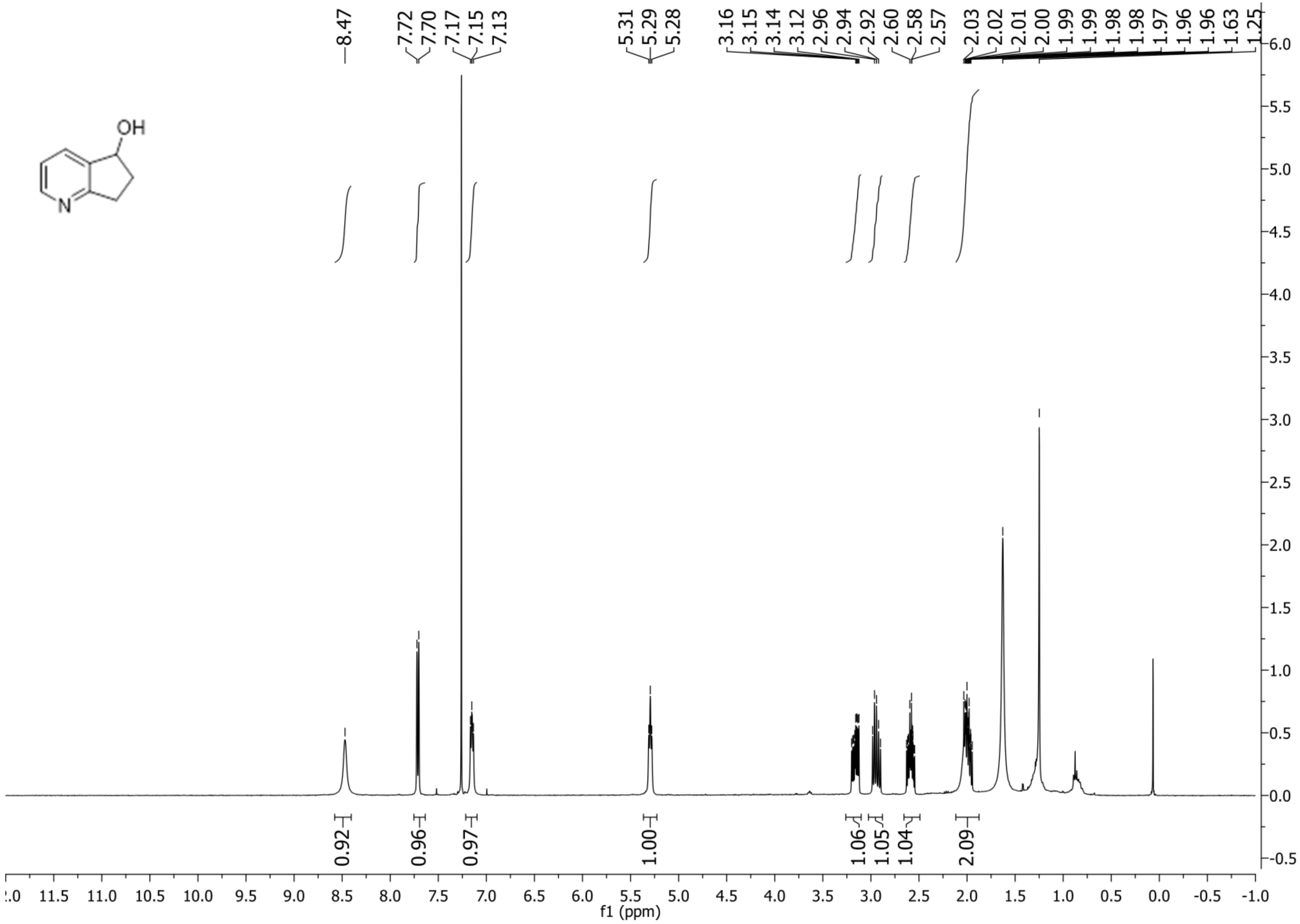

Figure S30. ^{1}H NMR (400 MHz, $CDCl_3$, 298K) of .*rac*-**2**.

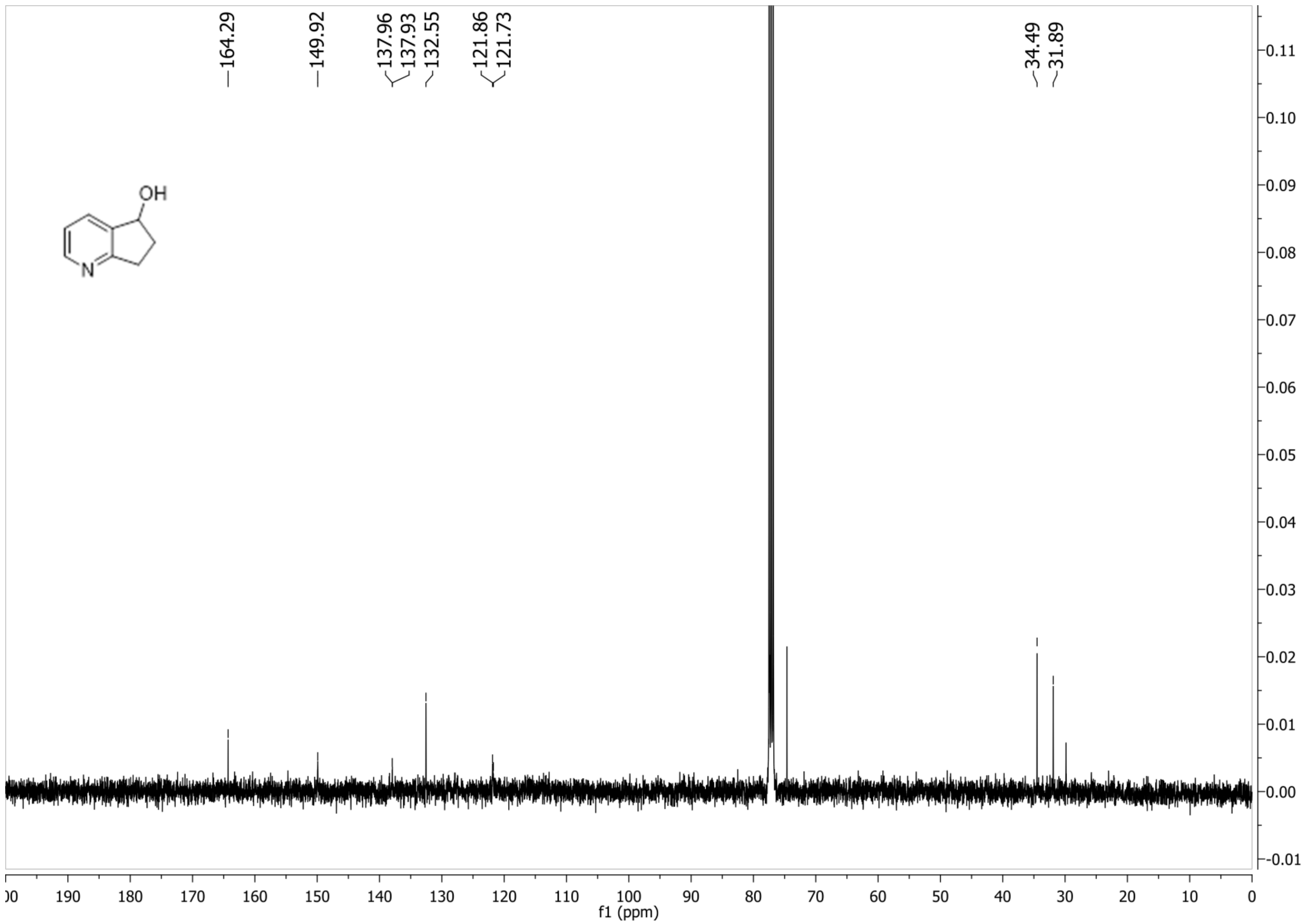

Figure S31. ^{13}C NMR (101 MHz, $CDCl_3$, 298K) of .*rac*-**2**.

2-Ethylpyridine-N-oxide **57**

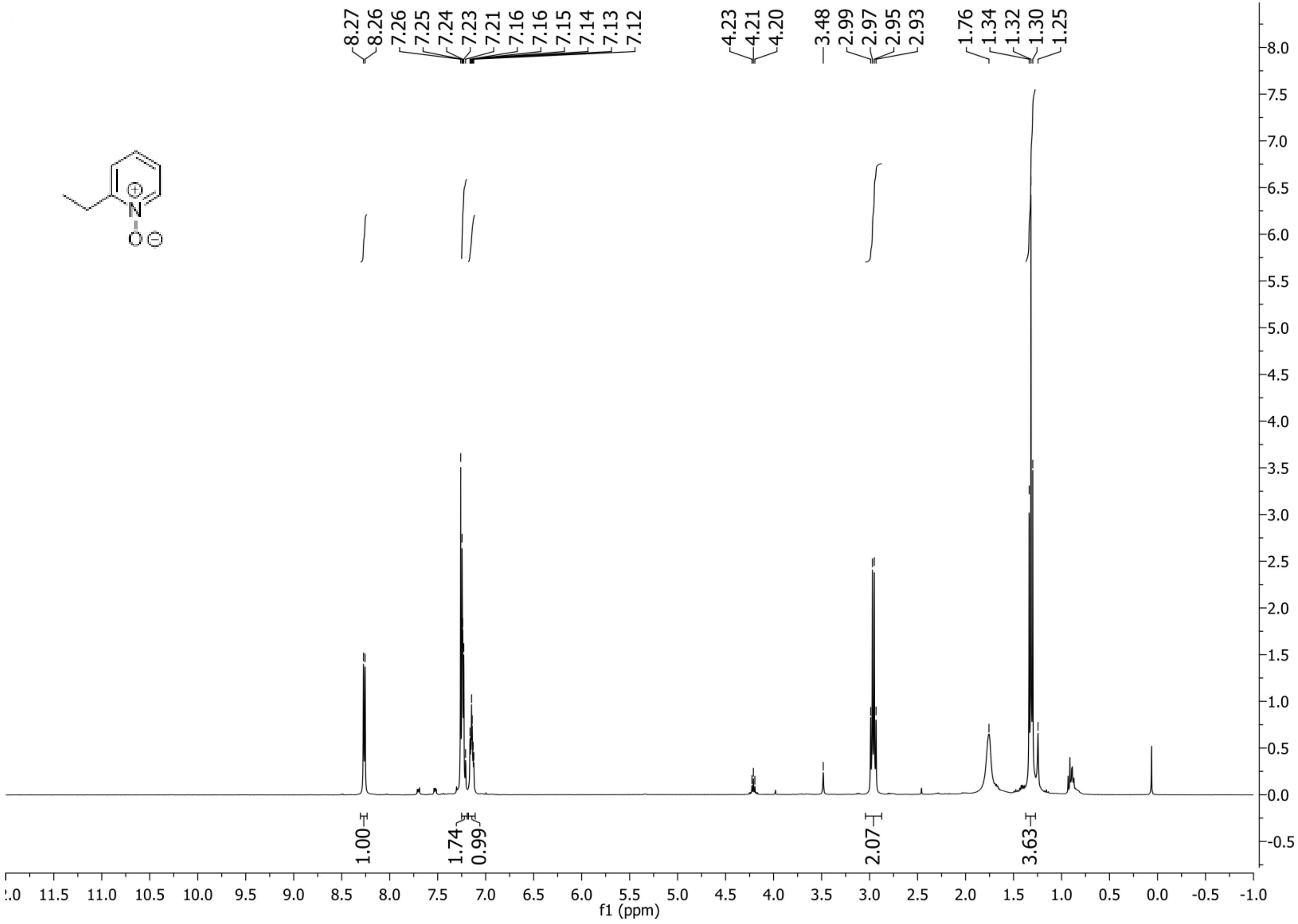

Figure S32. ^{1}H NMR (400 MHz, $CDCl_3$, 298K) of .**57**.

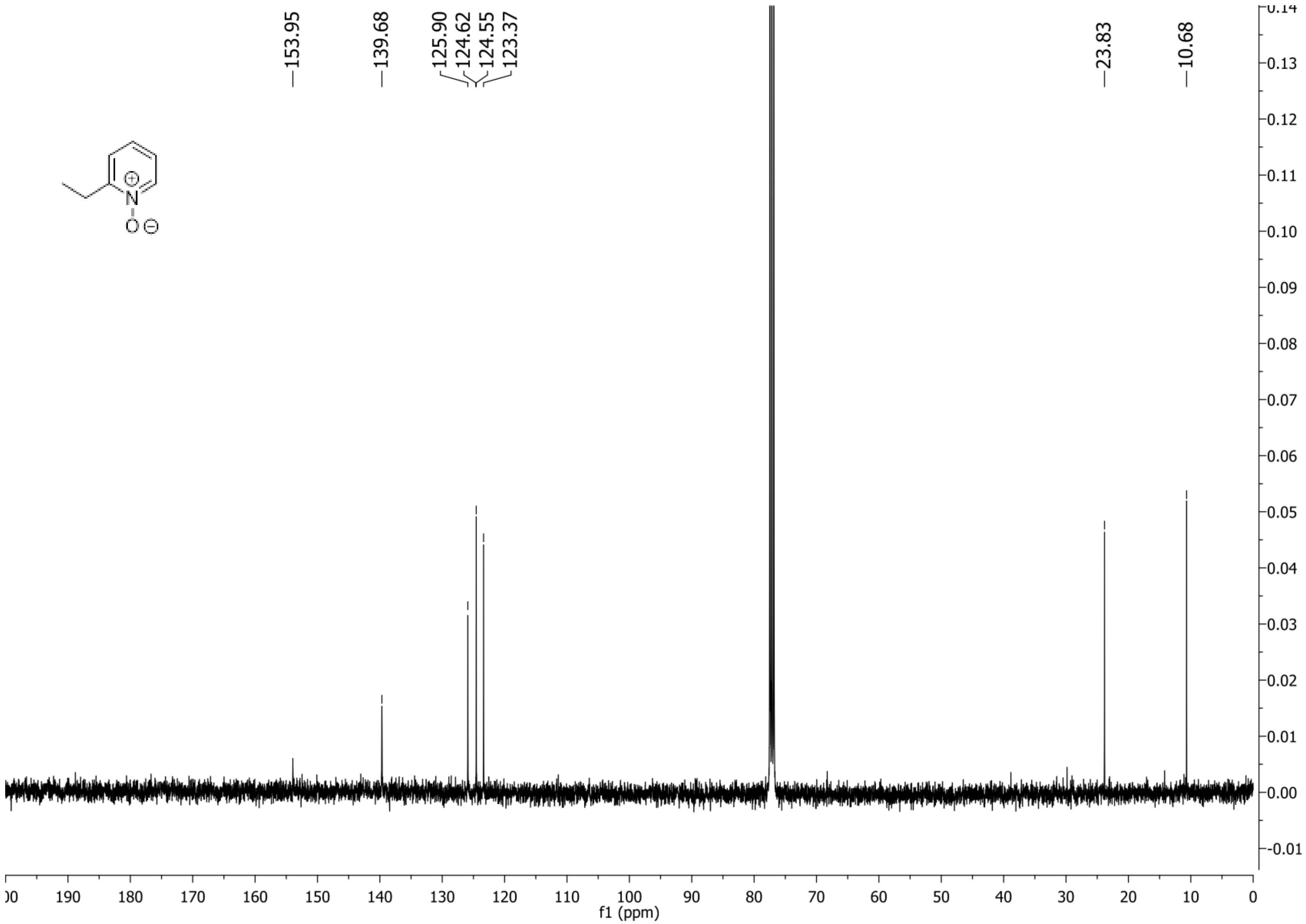

Figure S33. ^{13}C NMR (101 MHz, $CDCl_3$, 298K) of .**57**.

2-Propylpyridine-N-oxide **59**

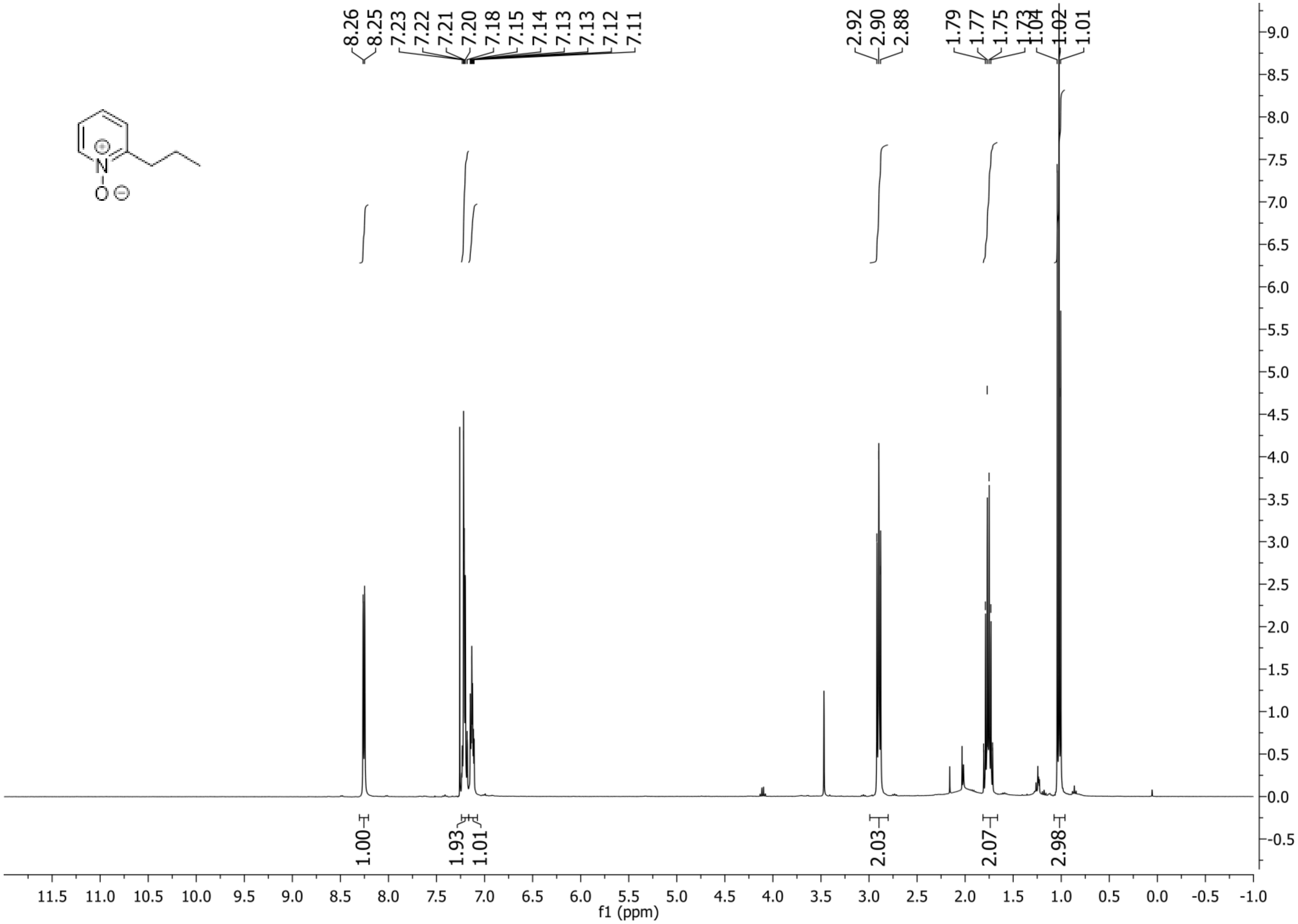

Figure S34. ^{1}H NMR (400 MHz, $CDCl_3$, 298K) of.**59**.

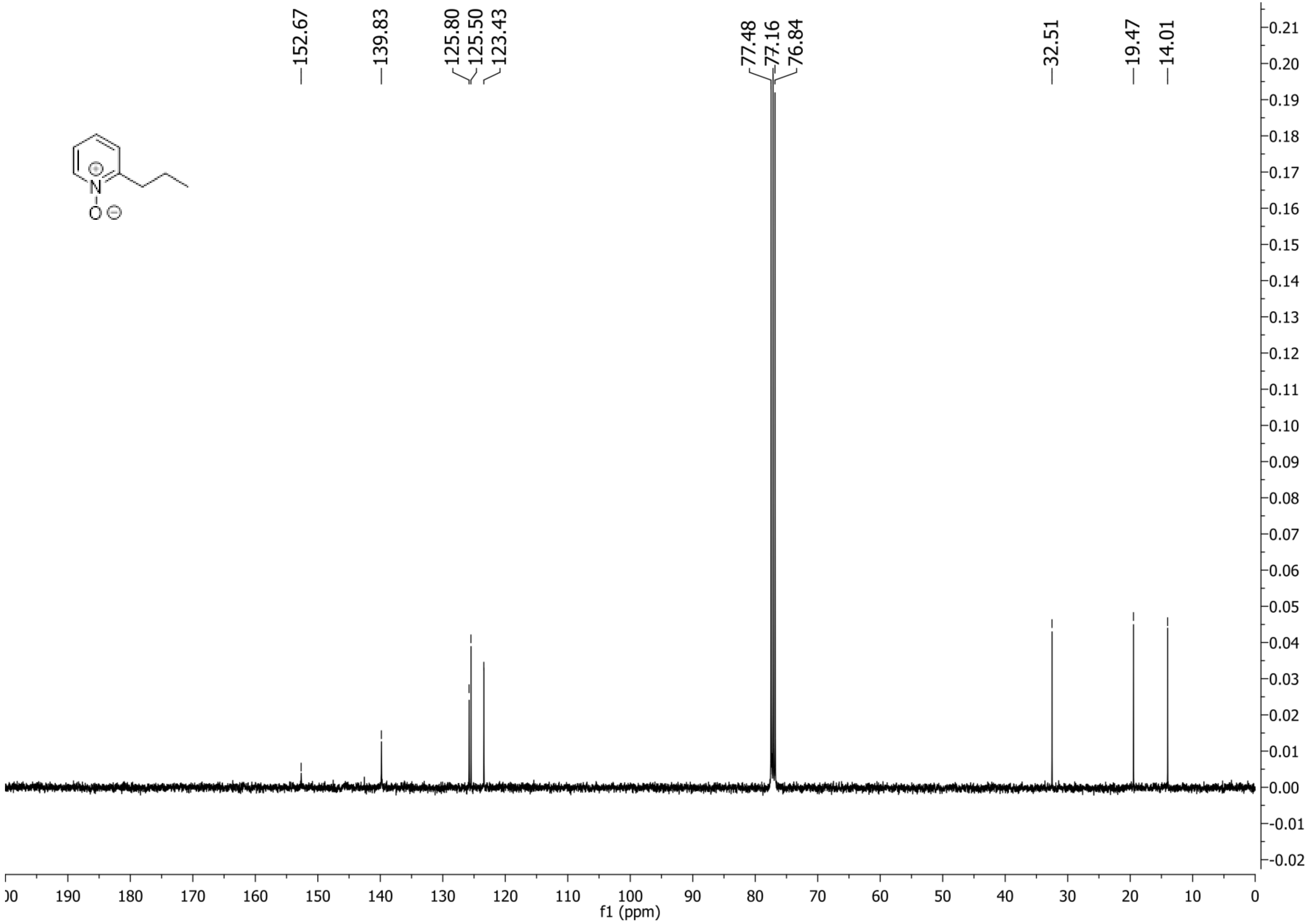

Figure S35. ^{13}C NMR (101 MHz, $CDCl_3$, 298K) of.**59**.

2-Methoxy-5-bromopyridine **S5**

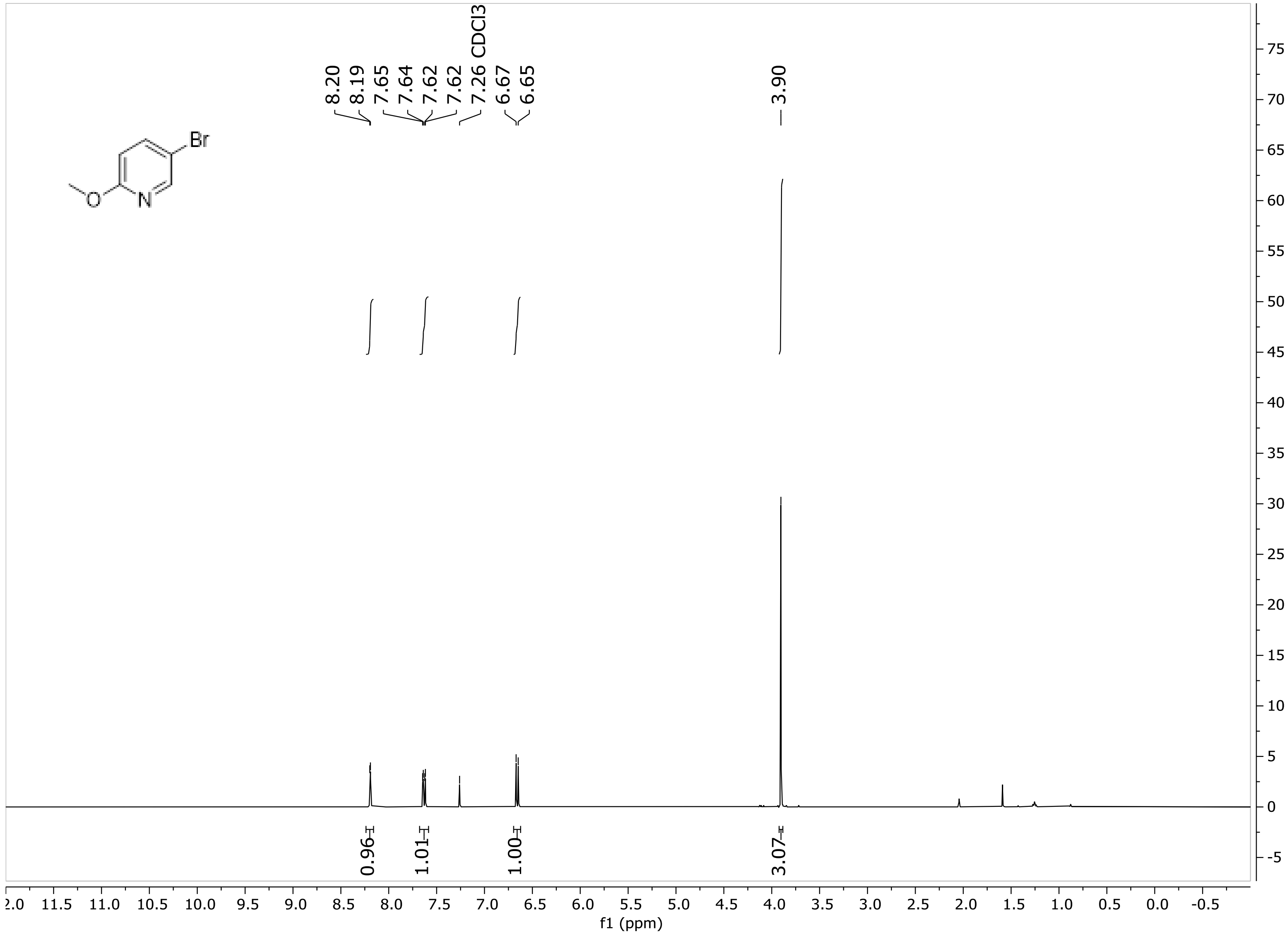

Figure S36. ^{1}H NMR (400 MHz, $CDCl_3$, 298K) of .**S5**.

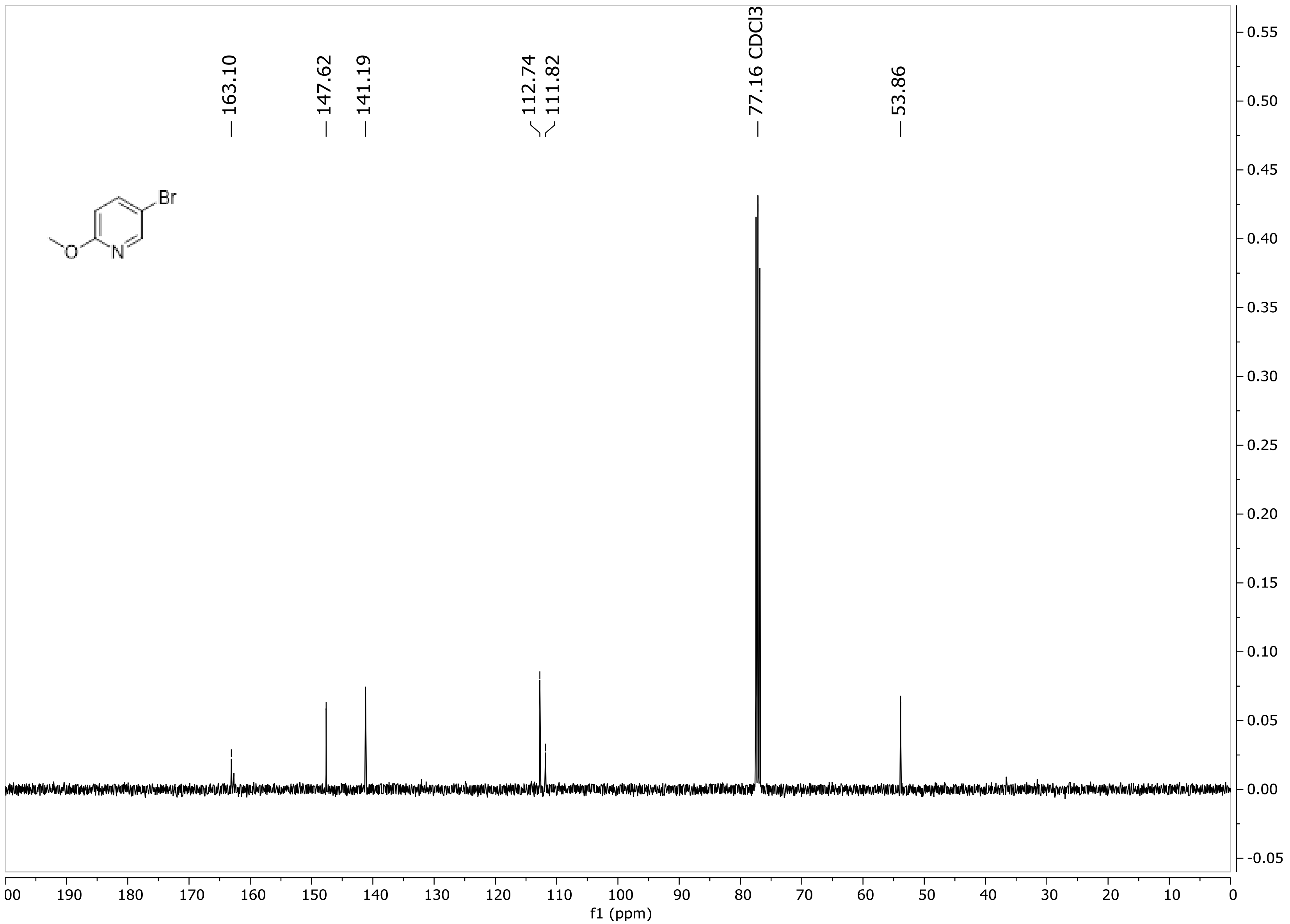

Figure S37. ^{13}C NMR (101 MHz, $CDCl_3$, 298K) of .**S5**.

2-Methoxy-5-methylpyridine **66**

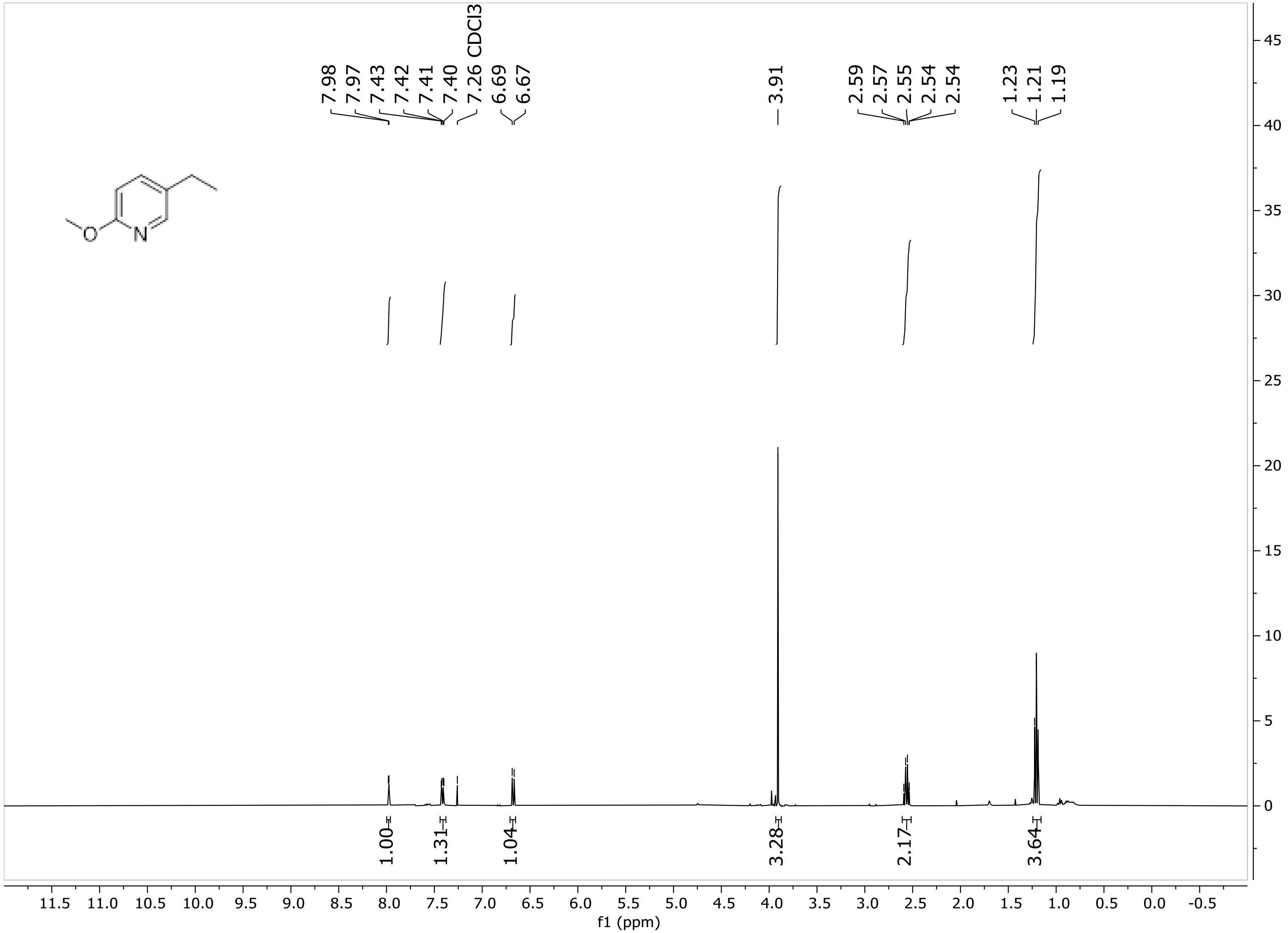

Figure S38. ^{1}H NMR (400 MHz, $CDCl_3$, 298K) of .**66**.

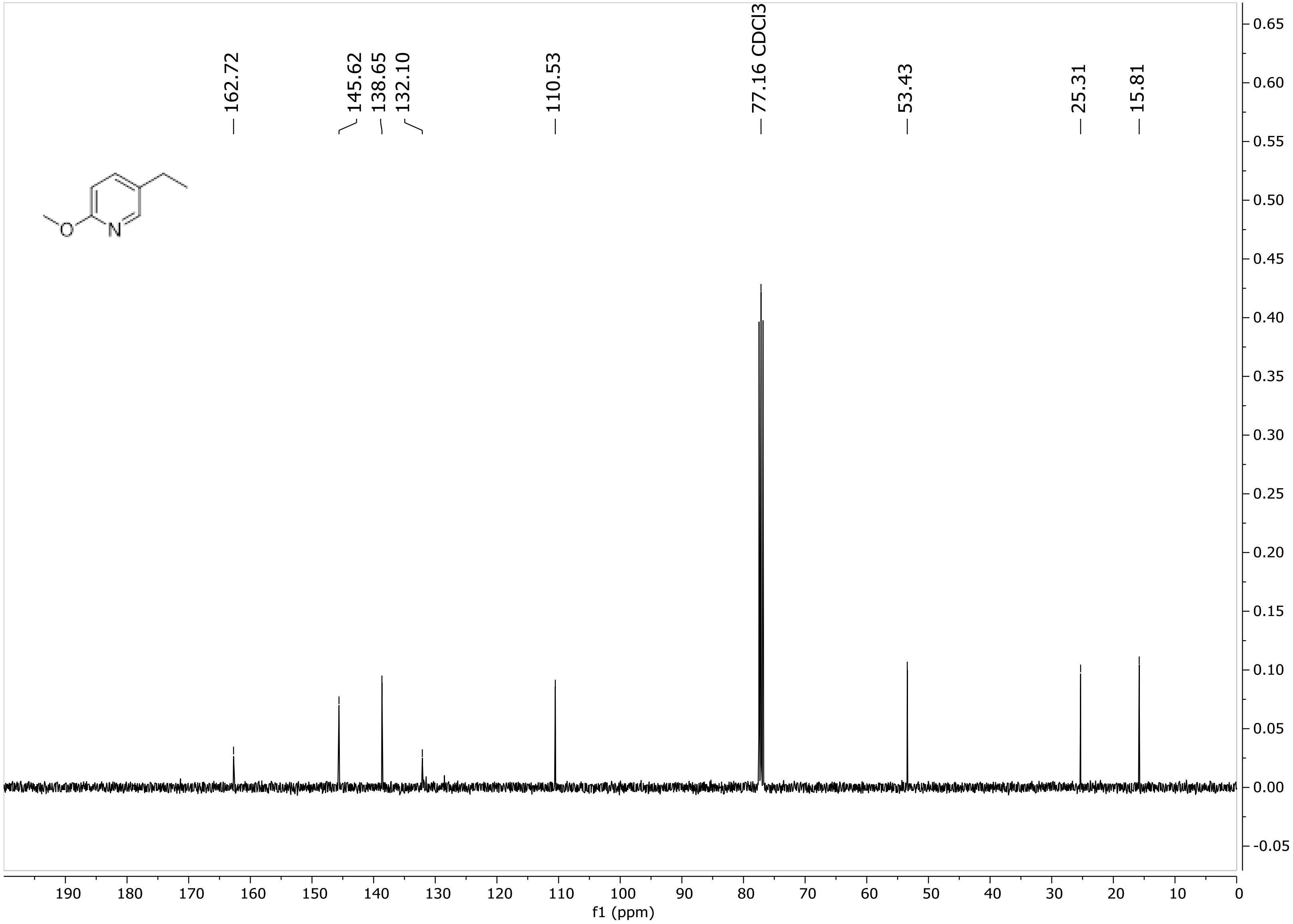

Figure S39. ^{13}C NMR (101 MHz, $CDCl_3$, 298K) of **.66**.

1-(6-Methoxypyridin-3-yl)-ethan-1-one **67b**

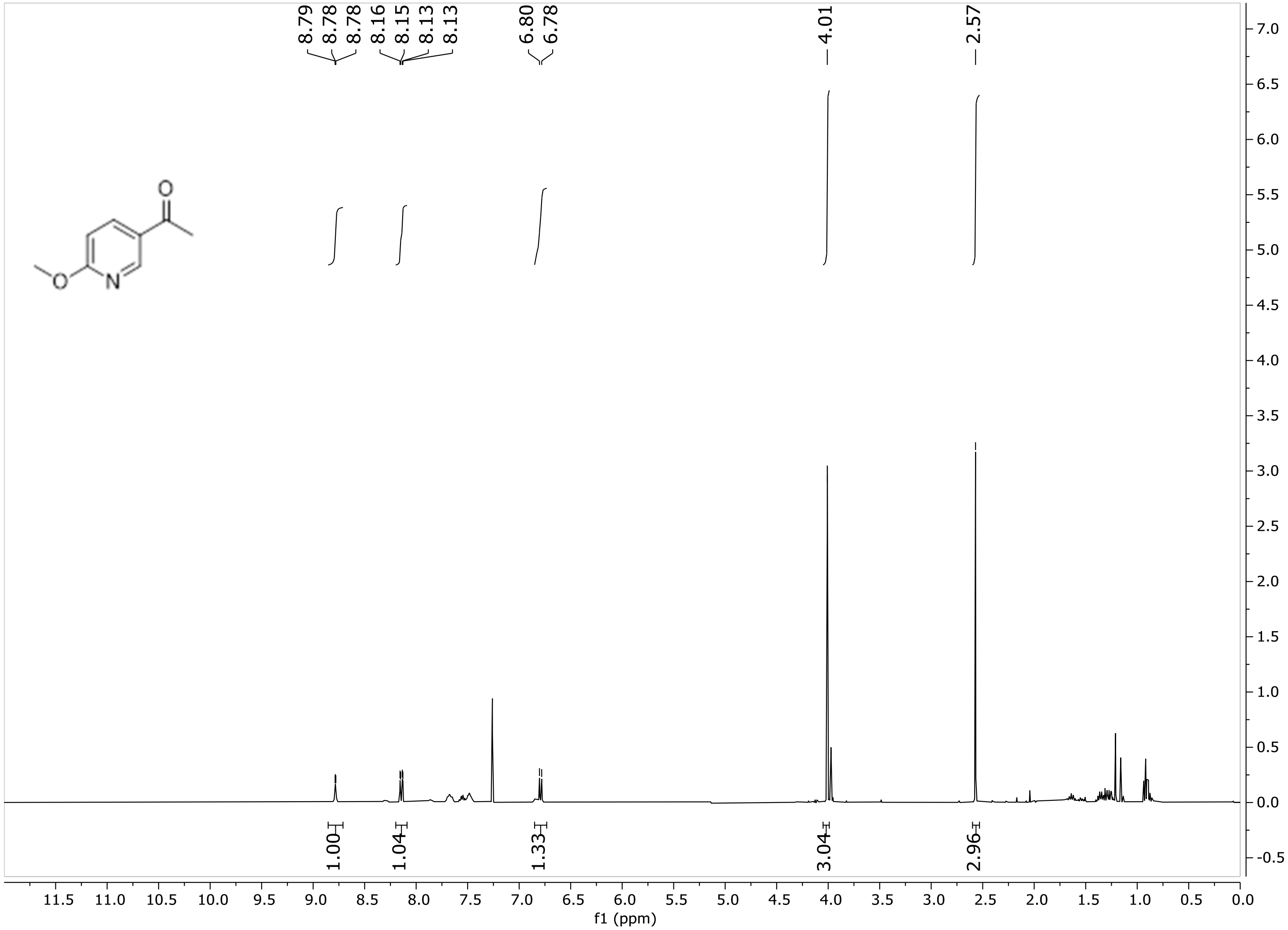

Figure S40. ^{1}H NMR (400 MHz, $CDCl_3$, 298K) of .**67b**.

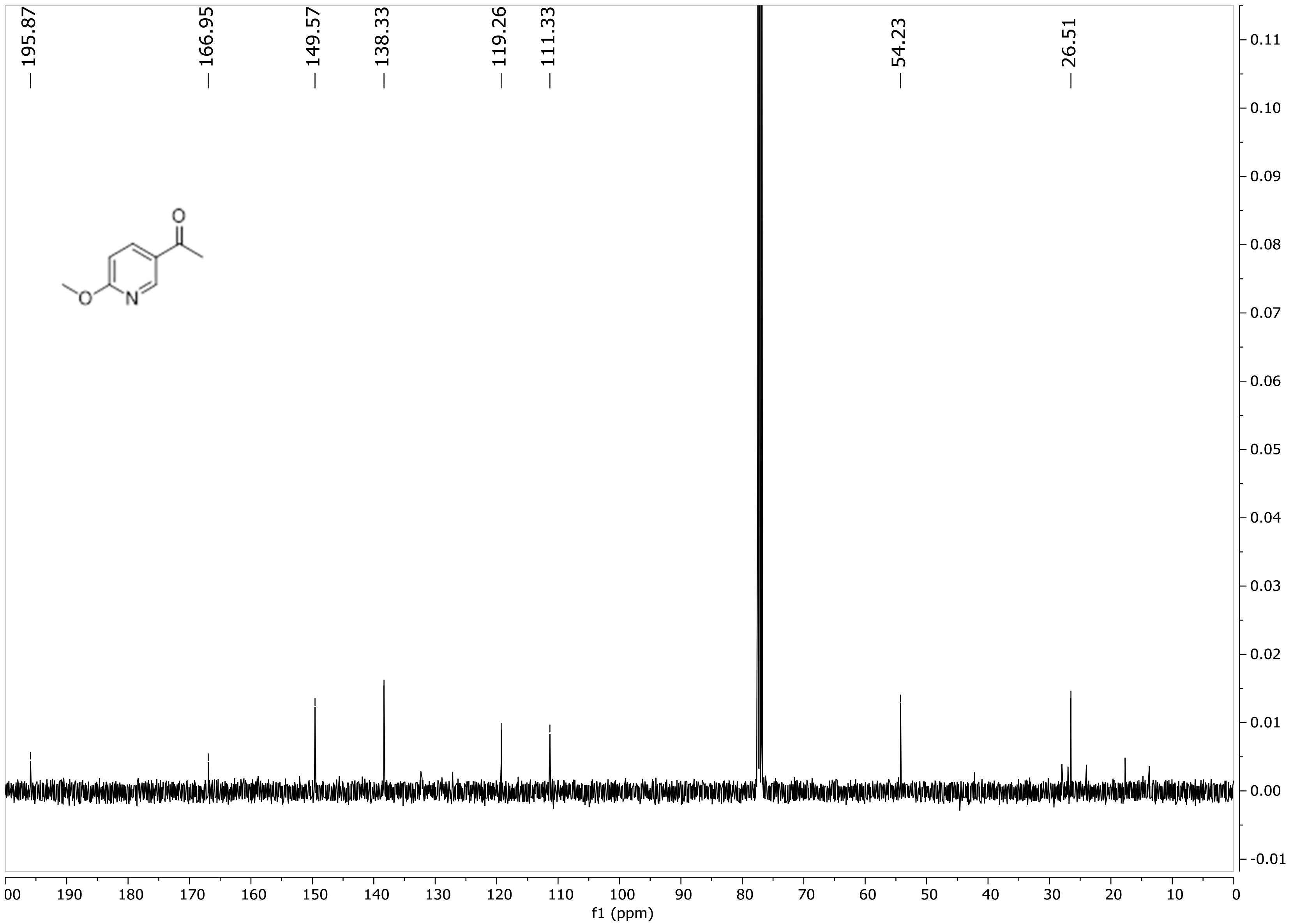

Figure S41. ^{13}C NMR (101 MHz, $CDCl_3$, 298K) of .**67b**.

*(+/-)-1-(6-Methoxypyridin-3-yl)-ethan-1-ol rac-***67a**

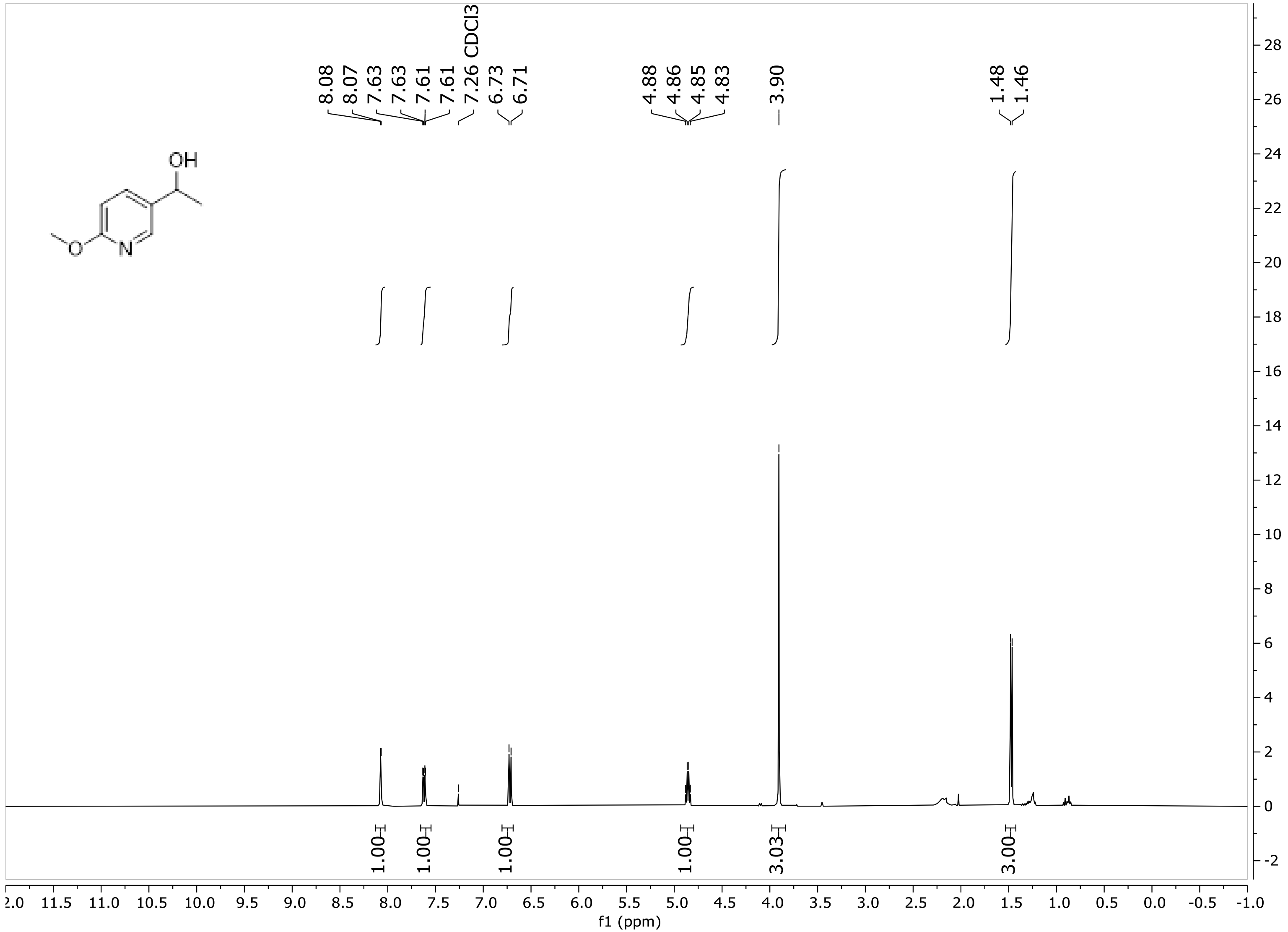

Figure S42. ^{1}H NMR (400 MHz, $CDCl_3$, 298K) of .*rac*-**67a**.

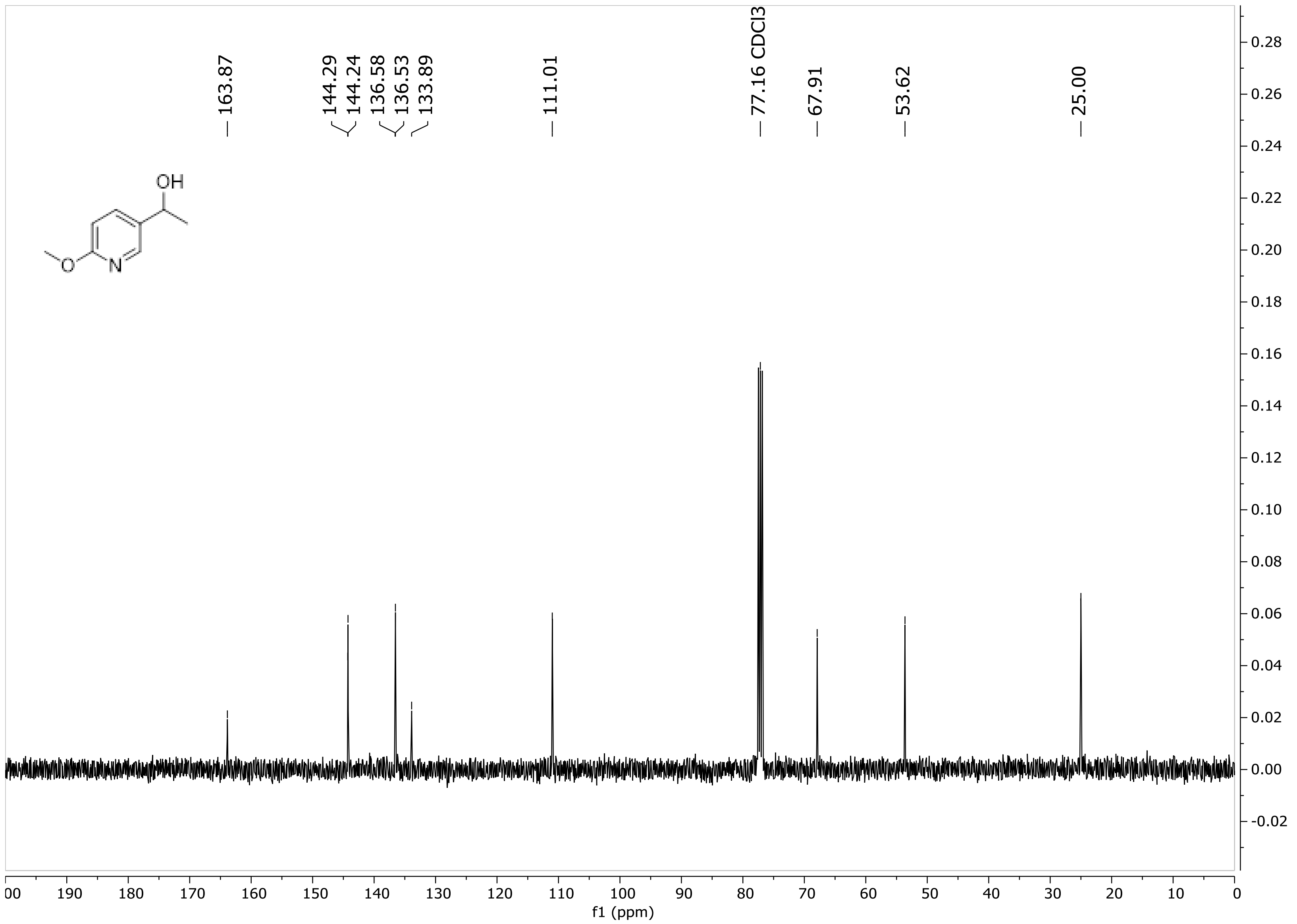

Figure S43. ^{13}C NMR (101 MHz, $CDCl_3$, 298K) of .*rac*-**67a**.

2-Chloro-5-ethylpyridine **68**

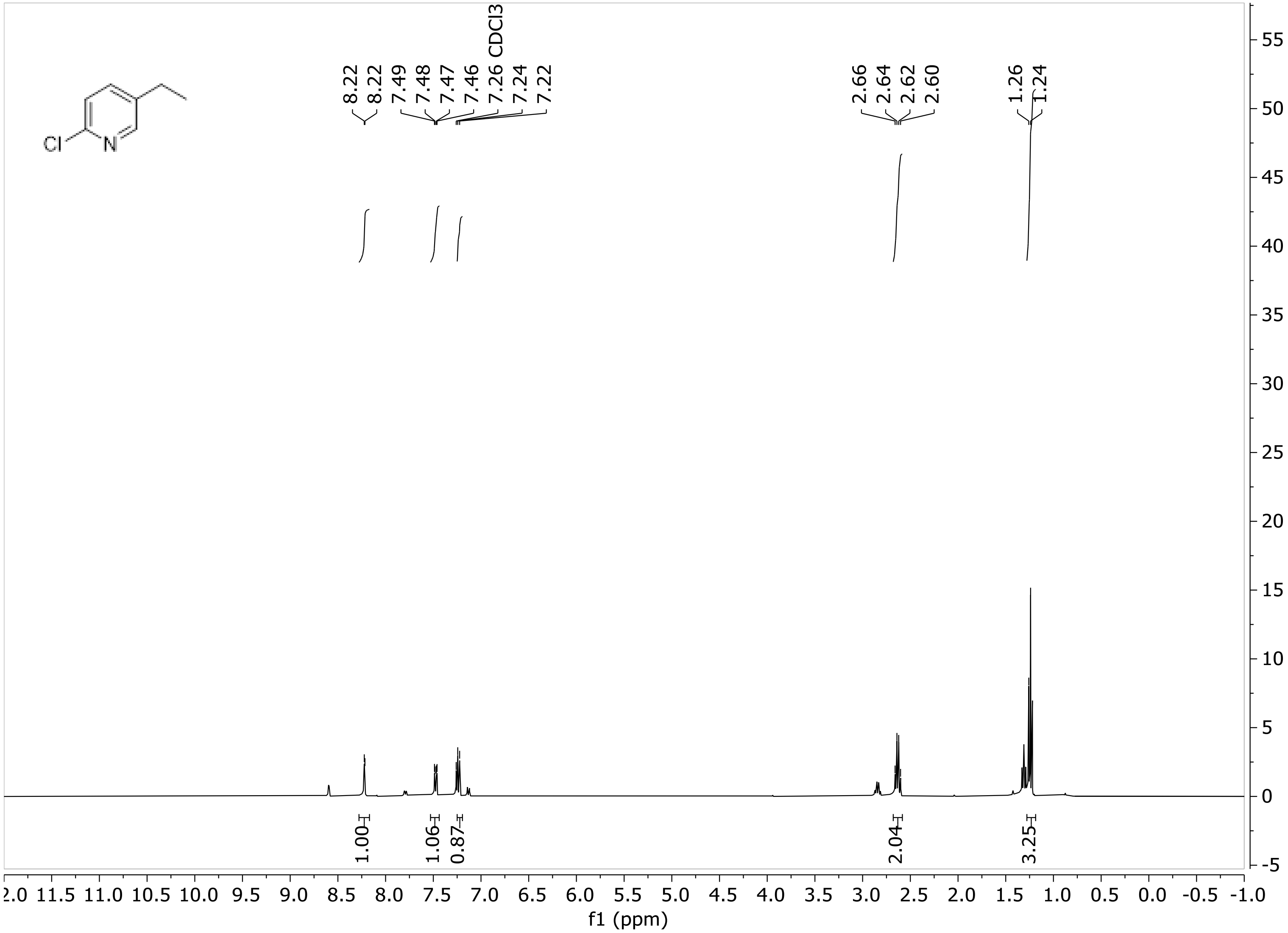

Figure S44. ^{1}H NMR (400 MHz, $CDCl_3$, 298K) of .**68**.

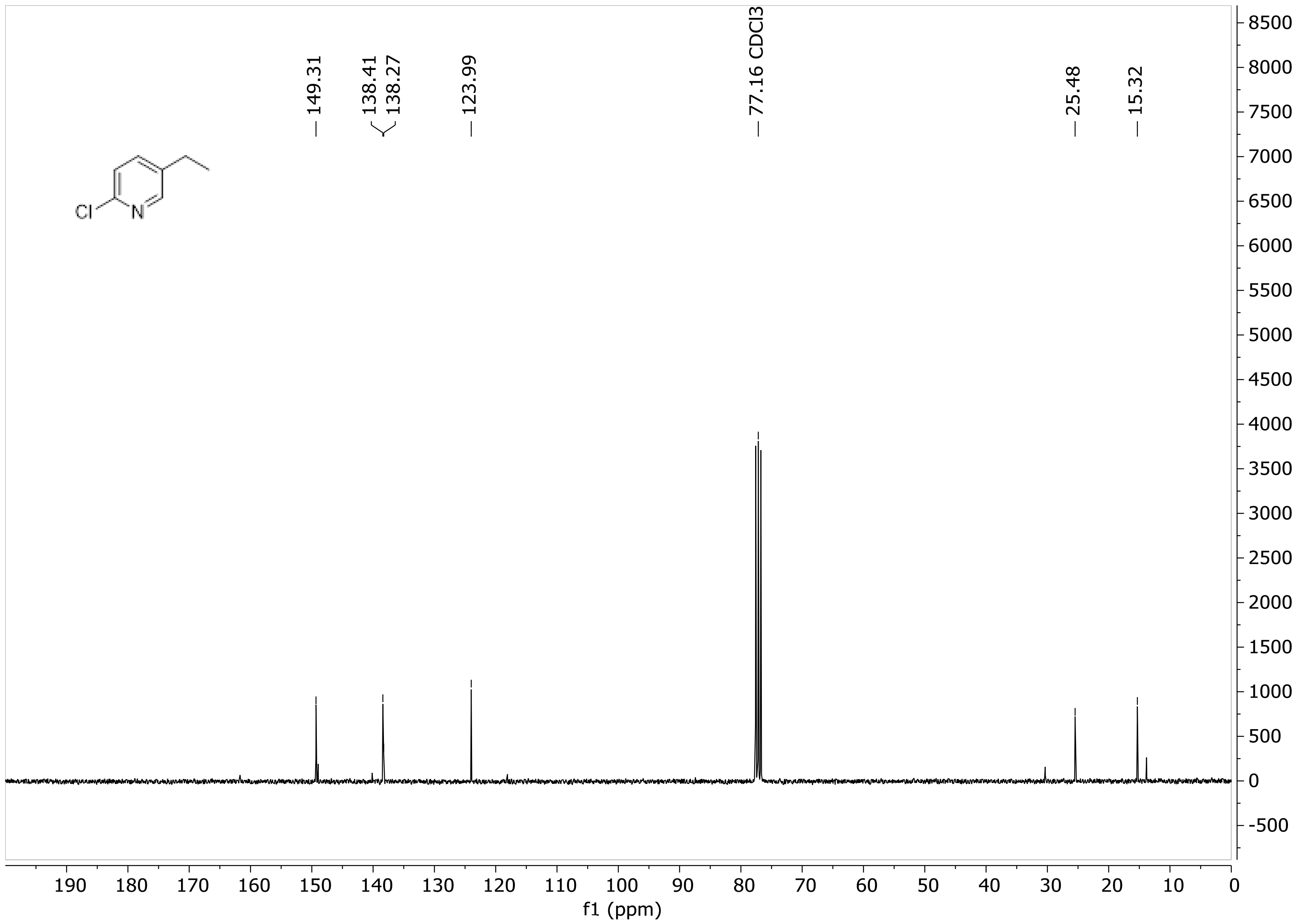

Figure S45. ^{13}C NMR (101 MHz, $CDCl_3$, 298K) of .**68**.

2-Cyano-5-bromopyridine **S6**

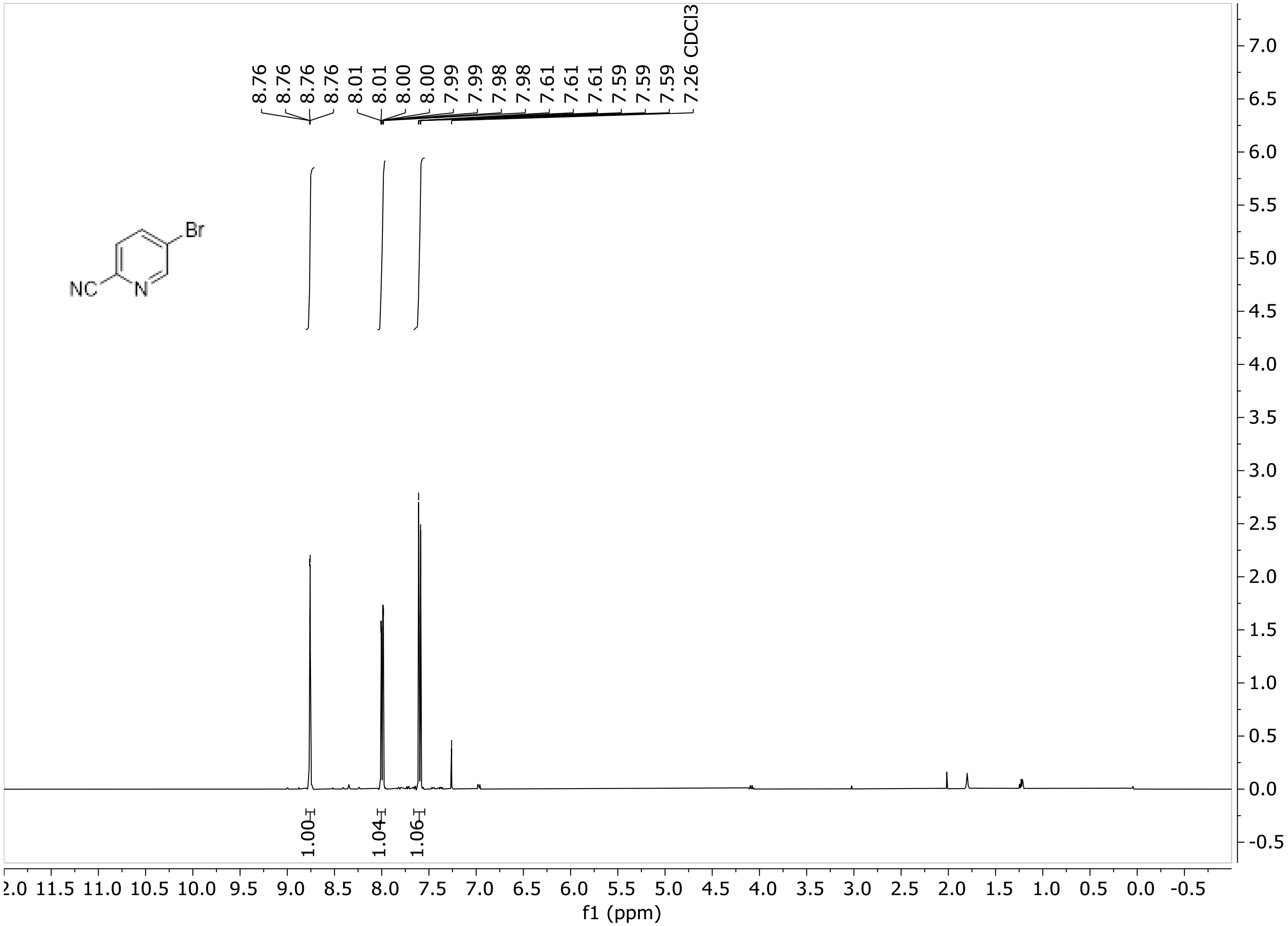

Figure S46. ^{1}H NMR (400 MHz, $CDCl_3$, 298K) of .**S6**.

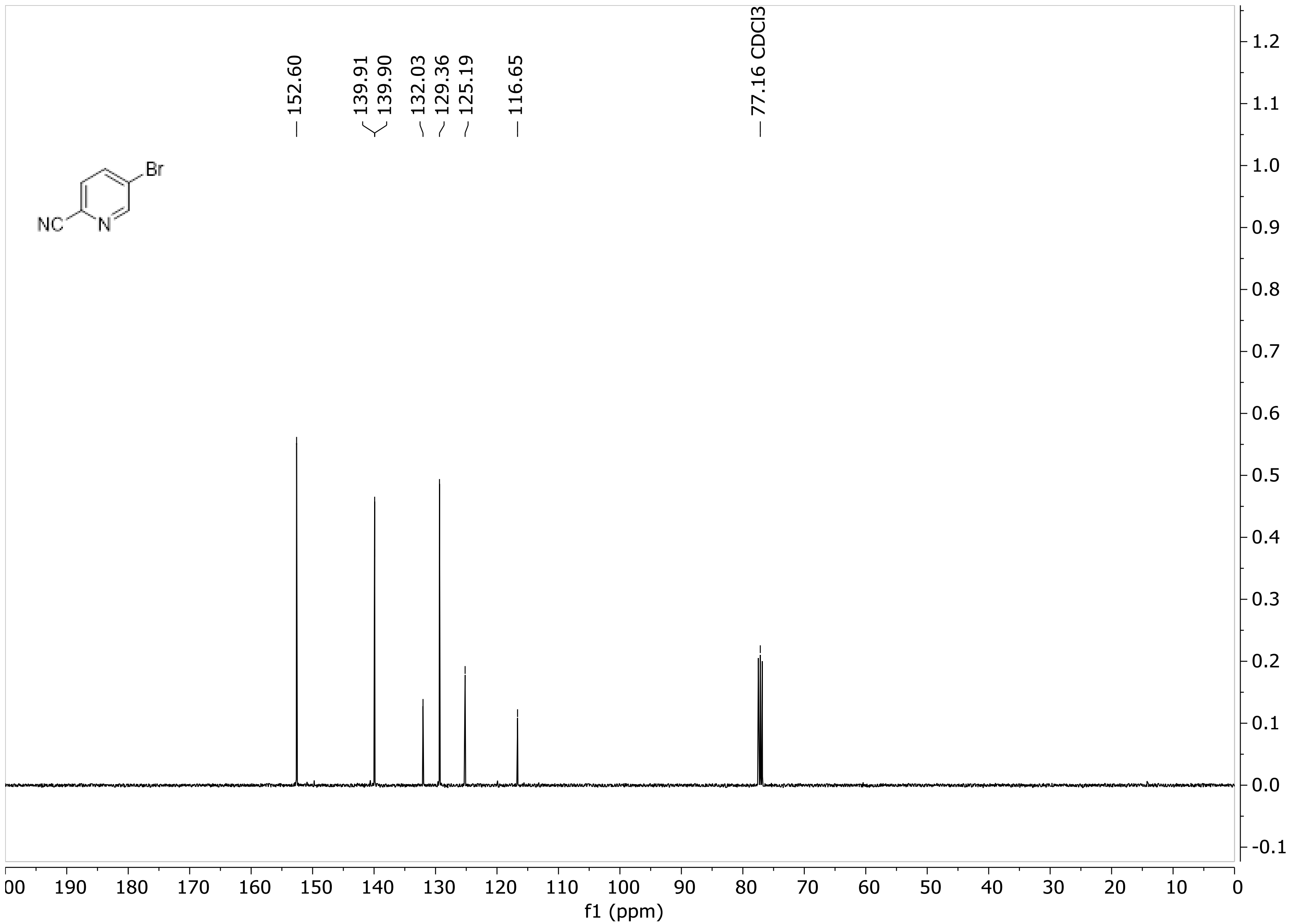

Figure S47. ^{13}C NMR (101 MHz, $CDCl_3$, 298K) of .**S6**.

2-Cyano-5-ethylpyridine **69**

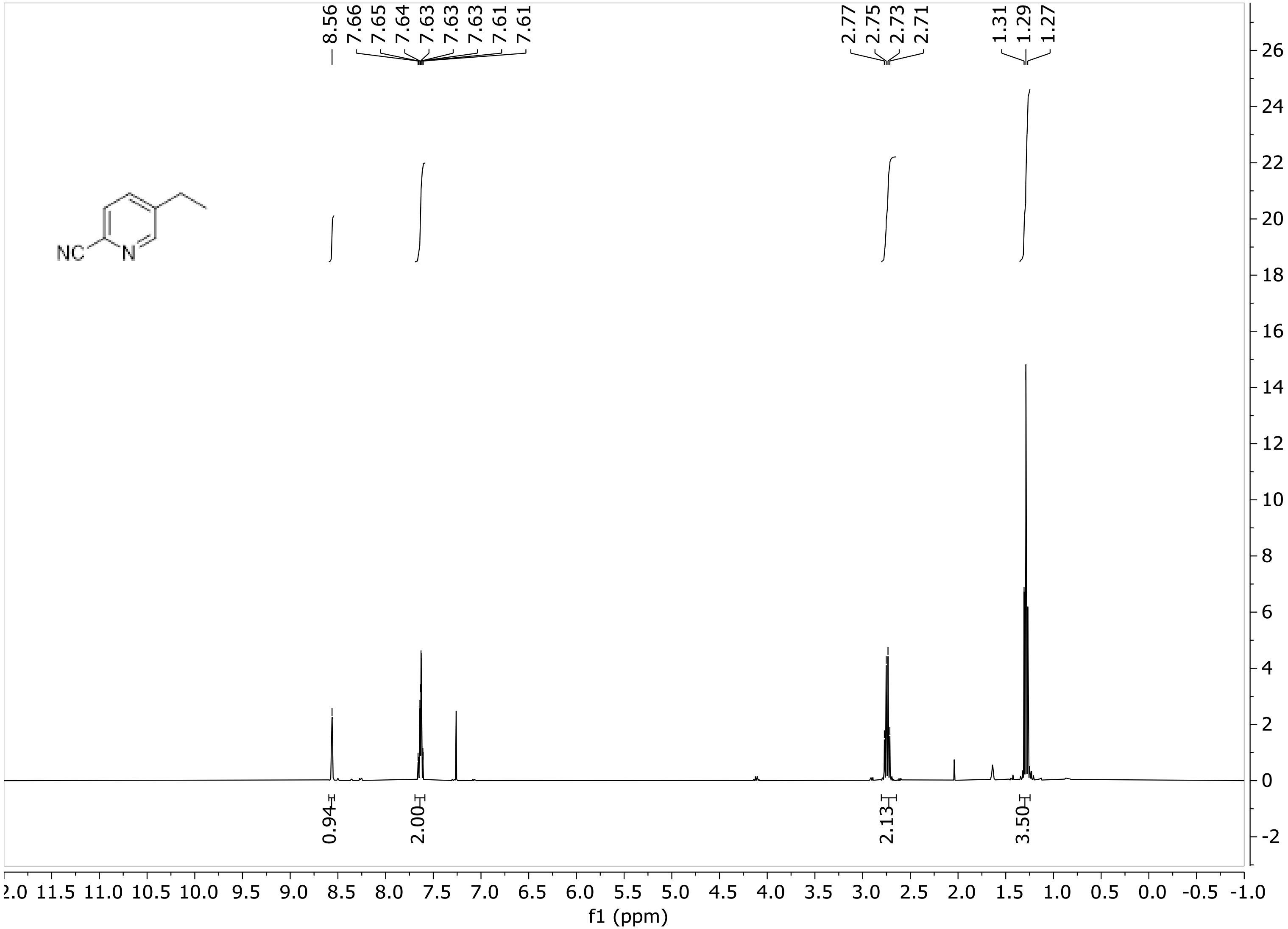

Figure S48. ^{1}H NMR (400 MHz, $CDCl_3$, 298K) of .**69**.

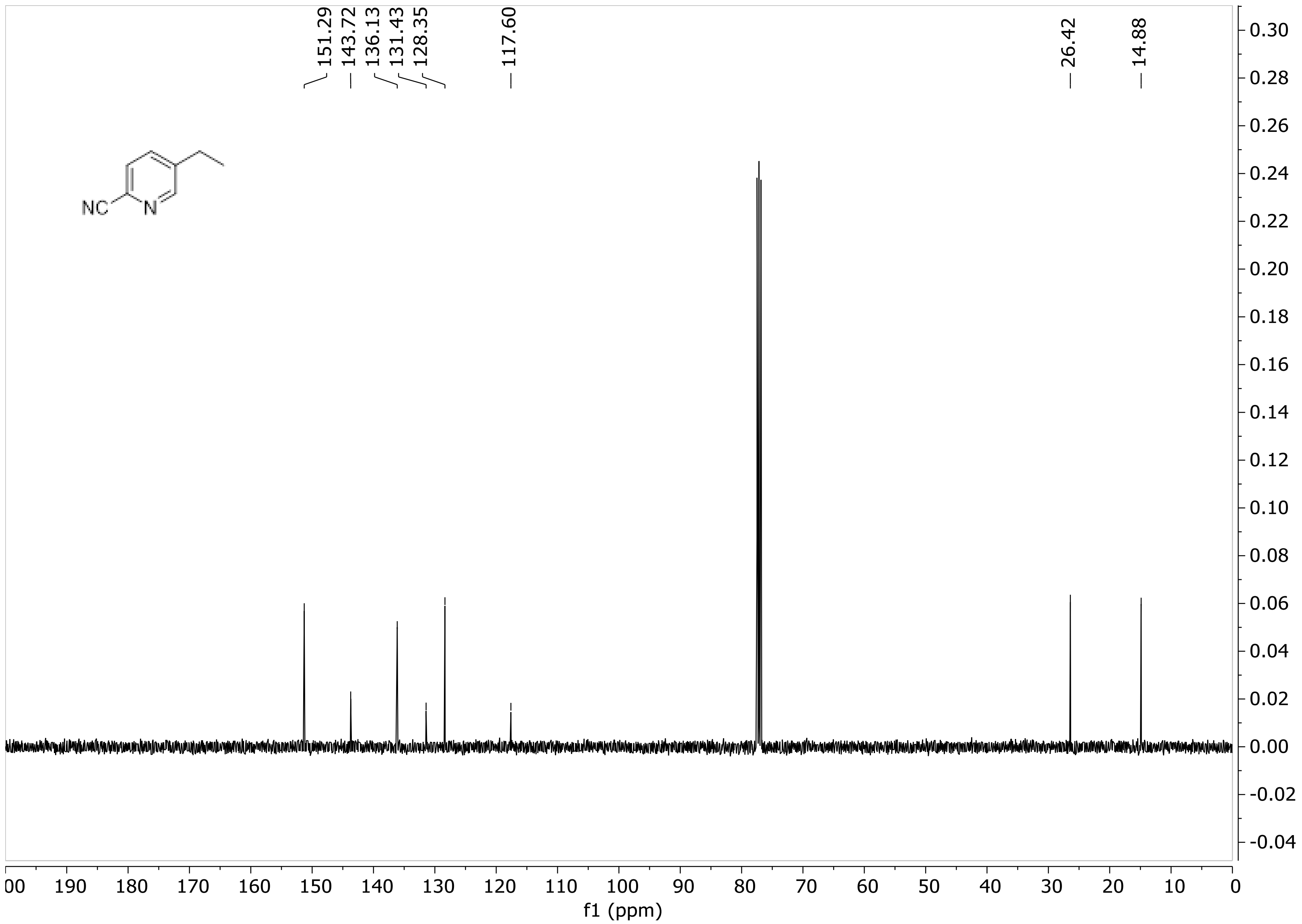

Figure S49. ^{13}C NMR (101 MHz, $CDCl_3$, 298K) of .**69**.

5-Acetylpicolinonitrile **S7**

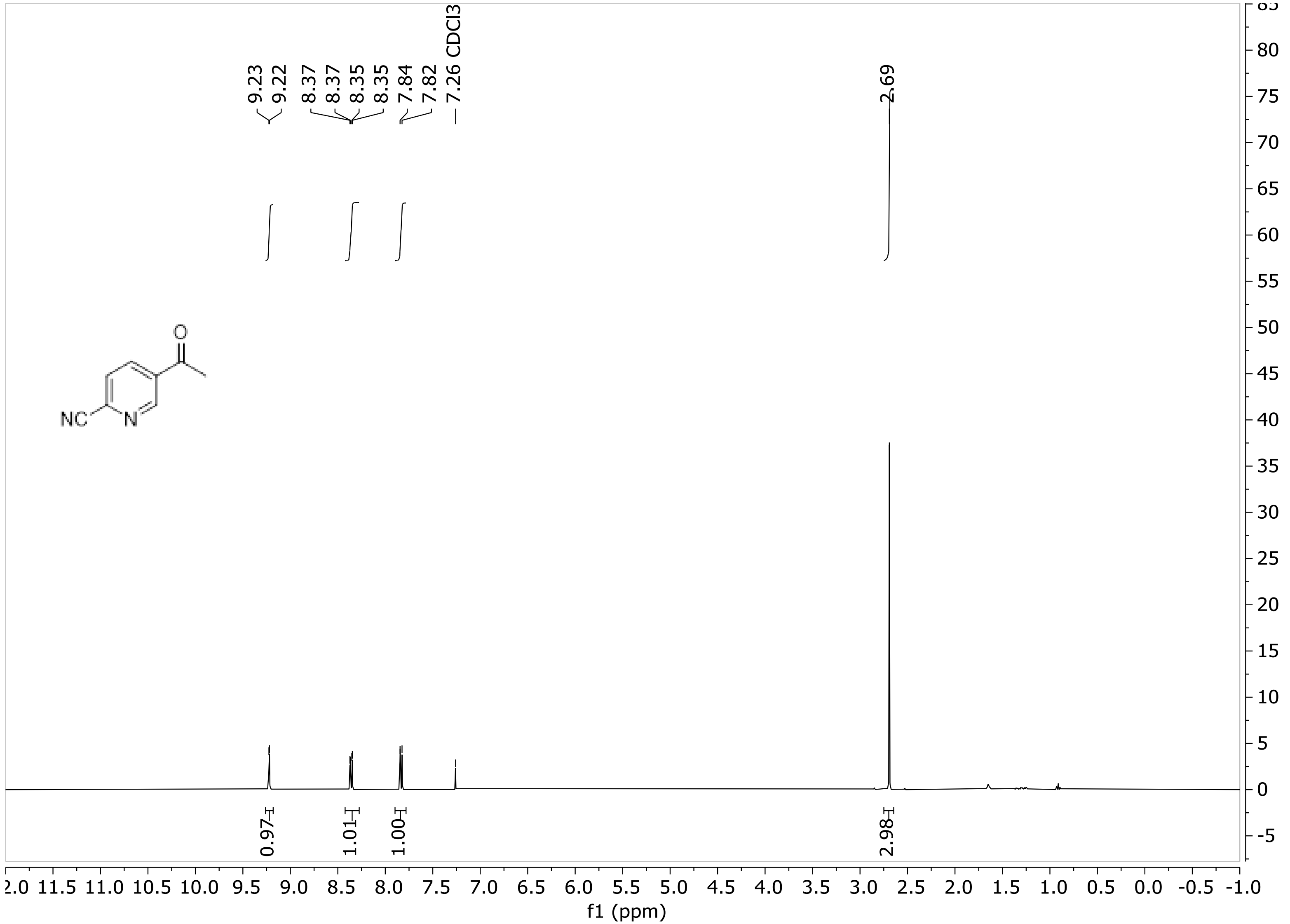

Figure S50. ^{1}H NMR (400 MHz, $CDCl_3$, 298K) of .**S7**.

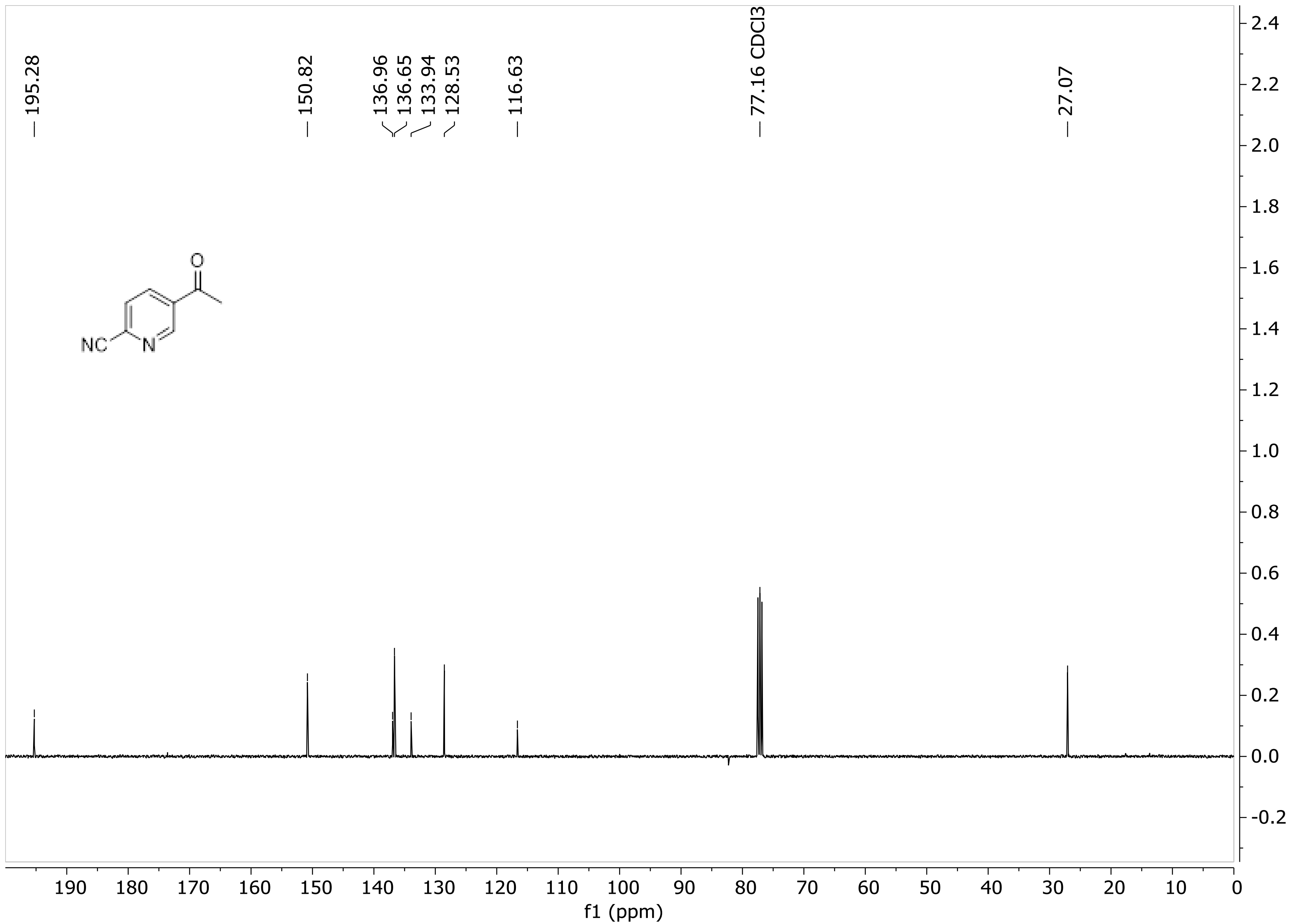

Figure S51. ^{13}C NMR (101 MHz, $CDCl_3$, 298K) of .**S7**.

*(+/-)-5-(1-Hydroxyethyl)-picolinonitrile rac-***73**

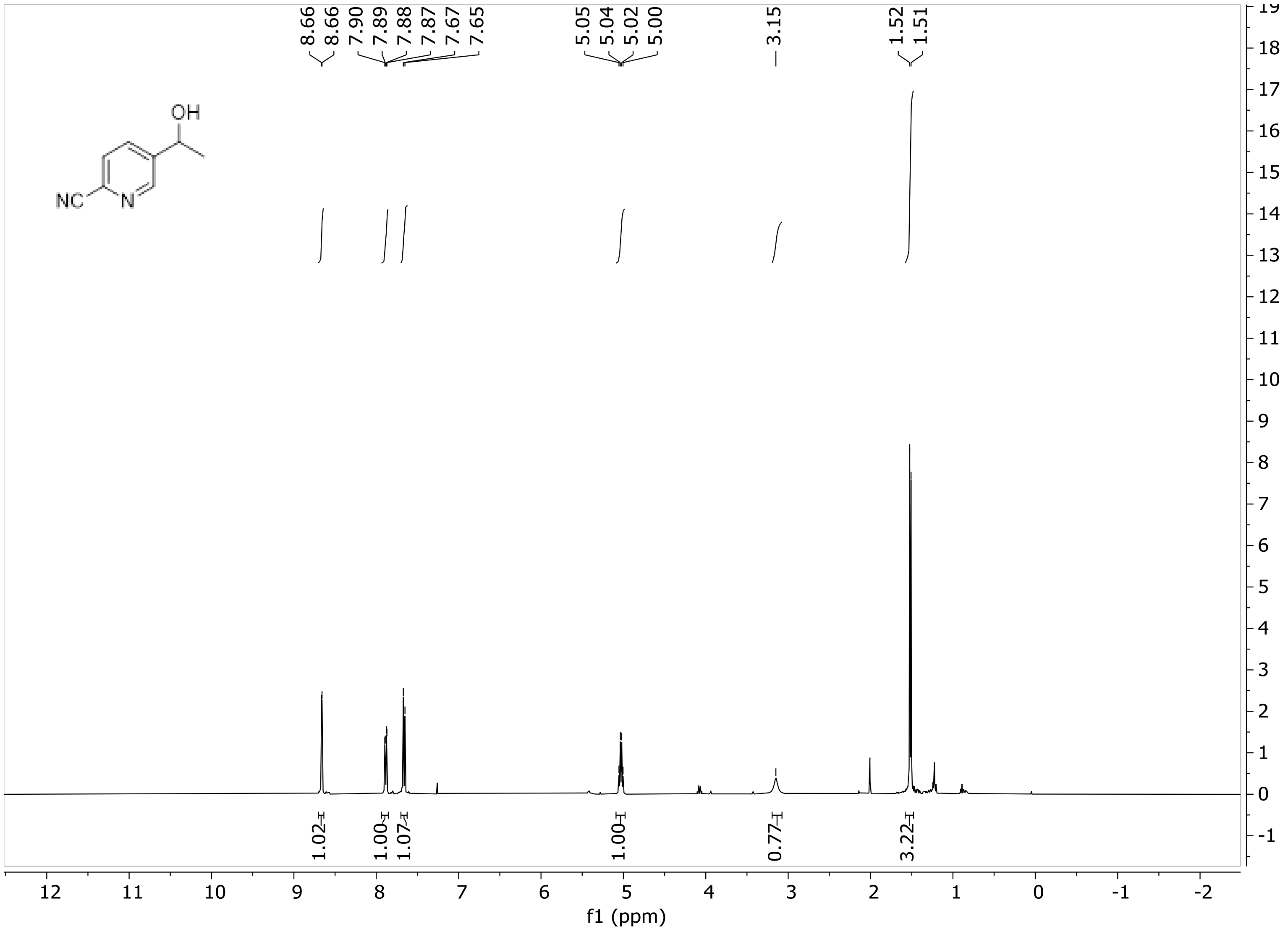

Figure S52. ^{1}H NMR (400 MHz, $CDCl_3$, 298K) of .*rac*-**73**.

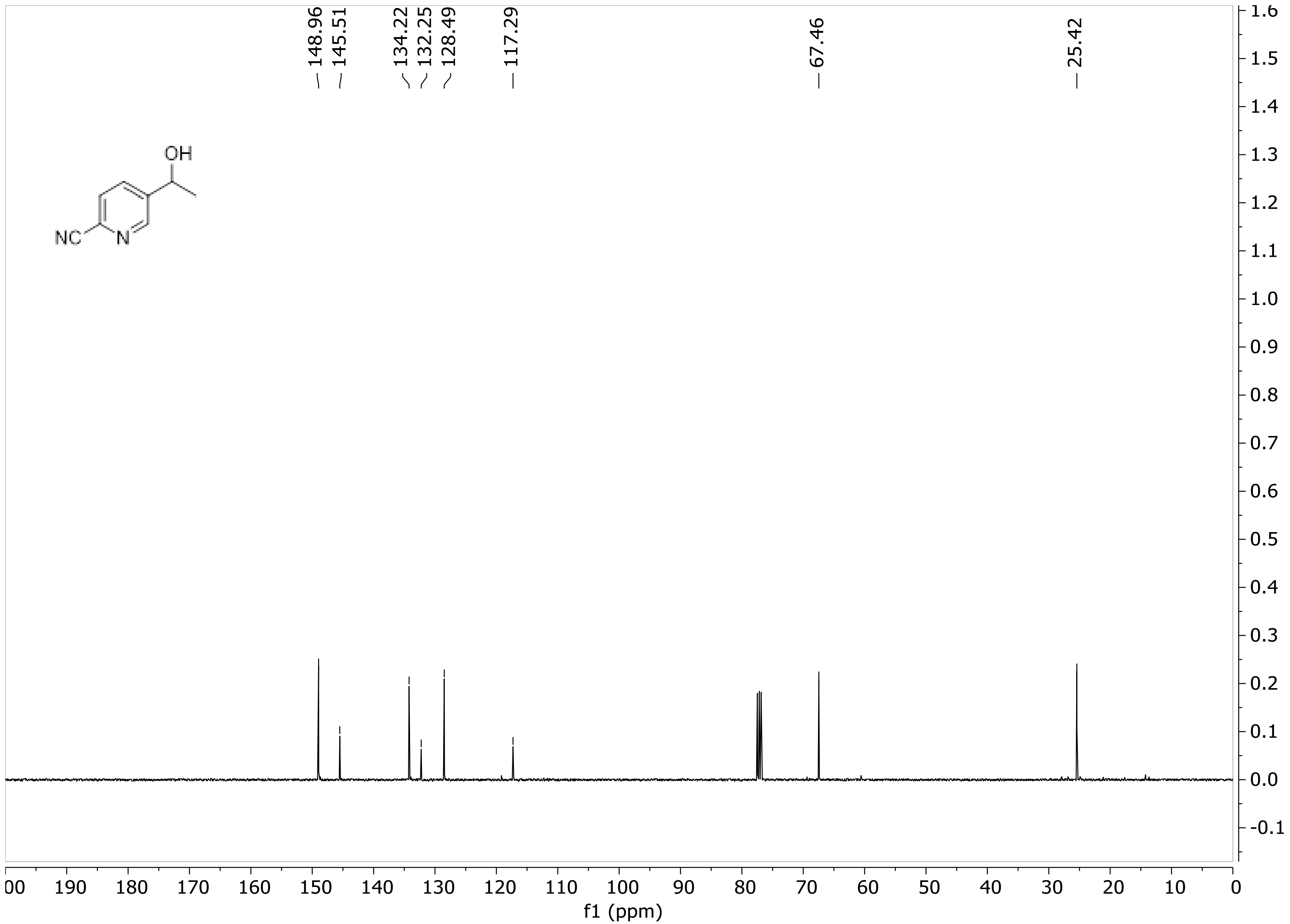

Figure S53. ^{13}C NMR (400 MHz, $CDCl_3$, 298K) of .*rac*-**73**.

5-Bromopicolinamide **S8**

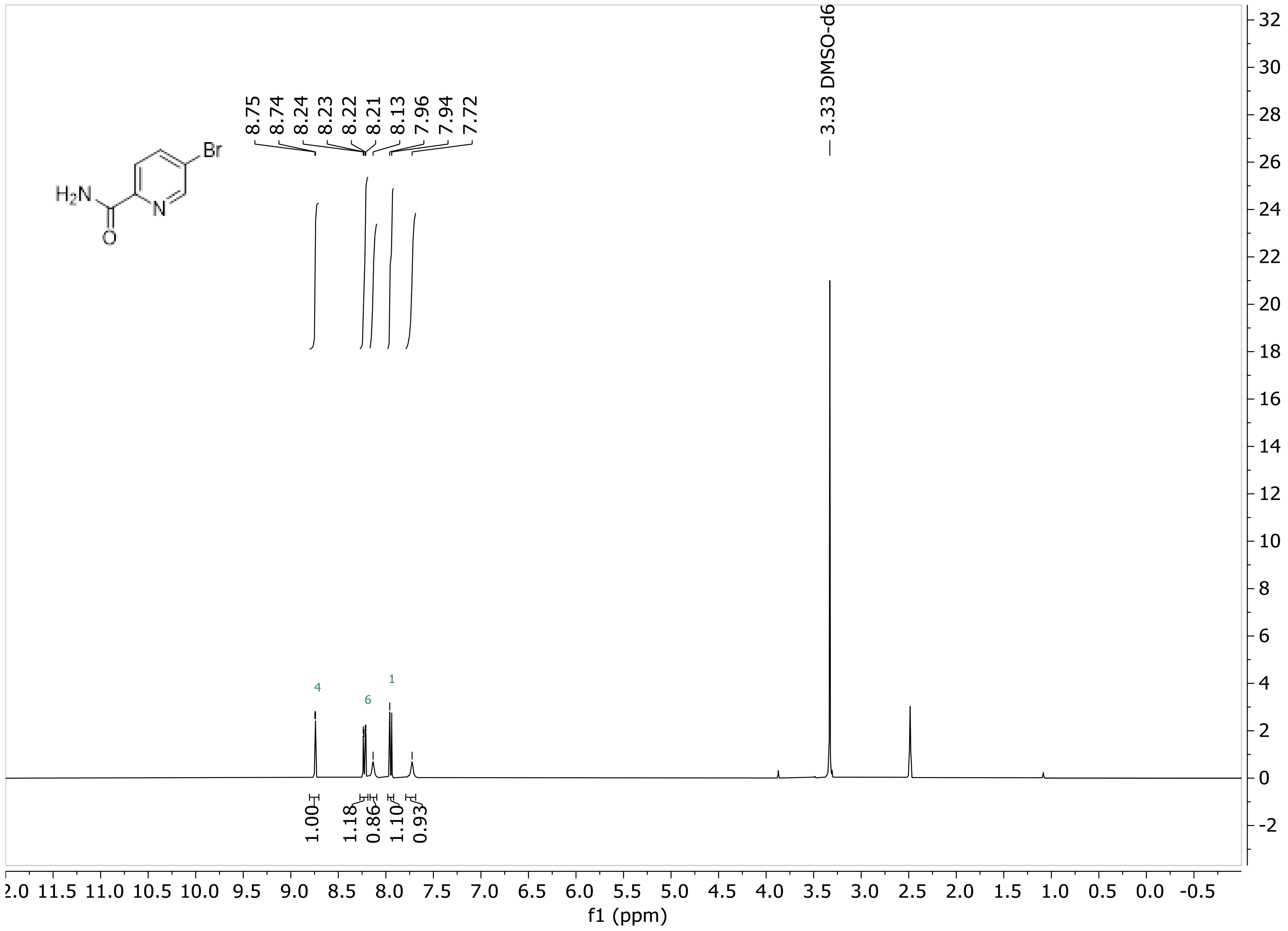

Figure S54. ^{1}H NMR (400 MHz, DMSO-d_6, 298K) of **S8**.

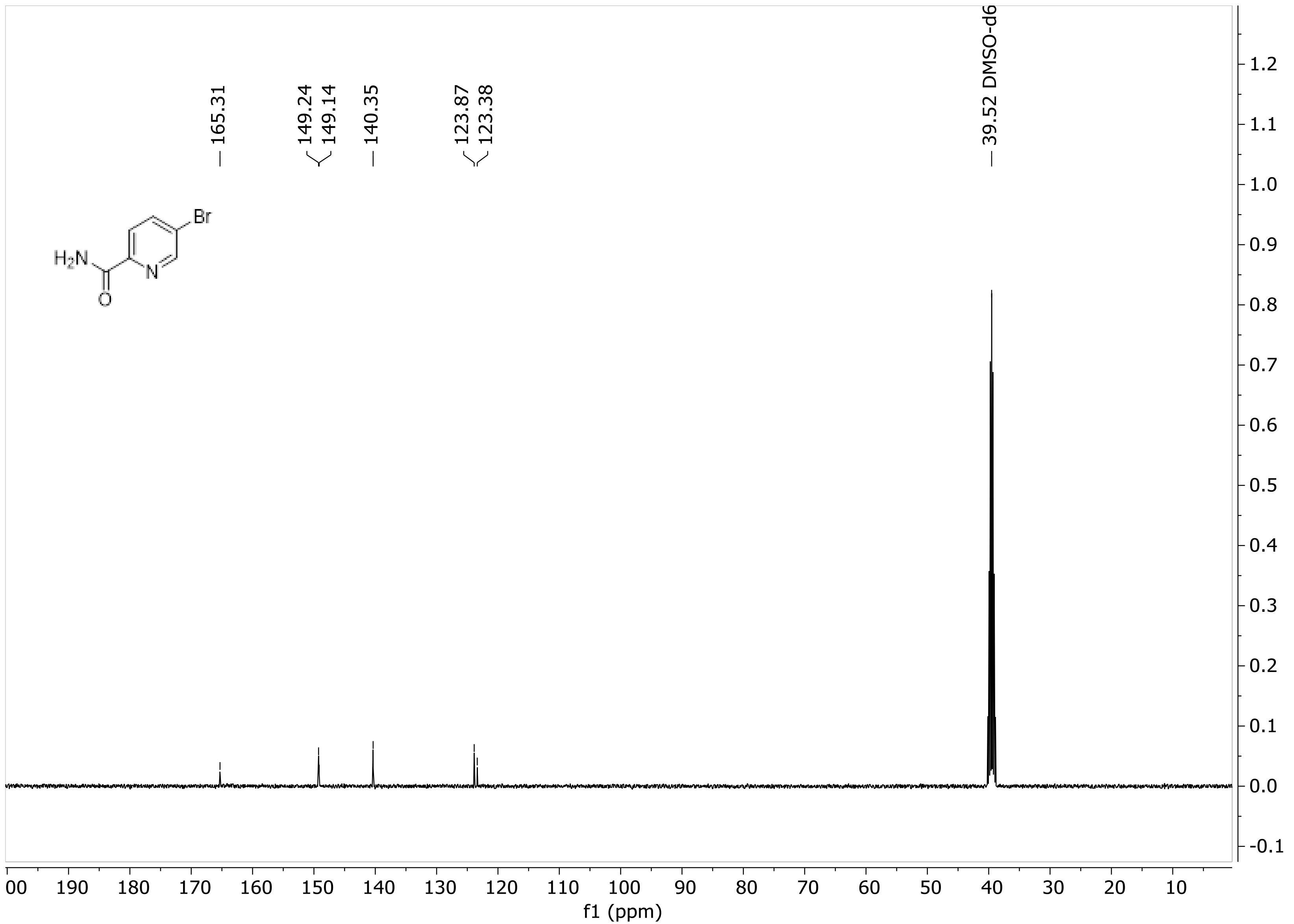

Figure S55. ^{13}C NMR (101 MHz, DMSO-d_6, 298K) of **S8**.

5-Ethylpicolinamide **70**

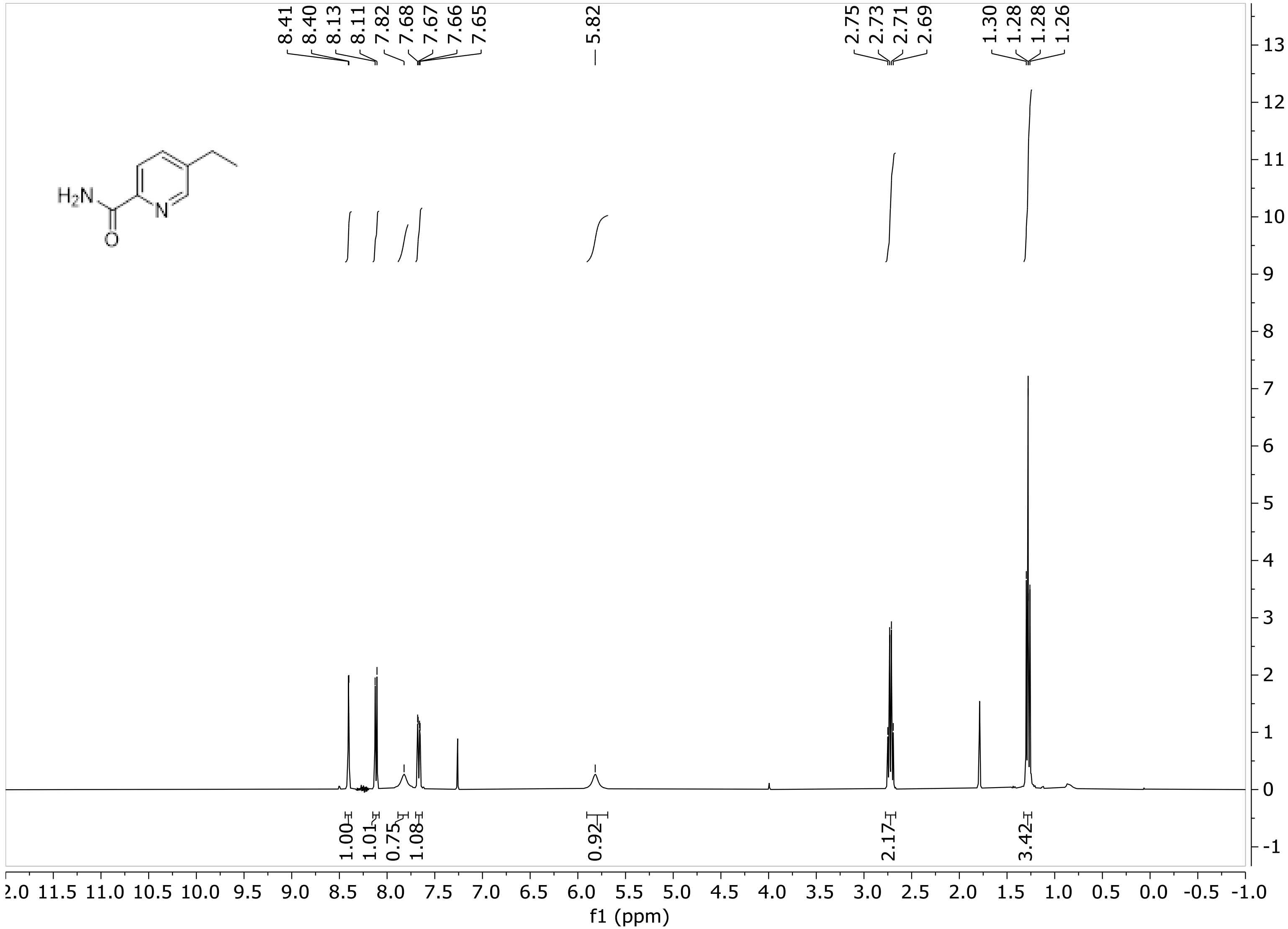

Figure S56. ^{1}H NMR (400 MHz, $CDCl_3$, 298K) of .**70**.

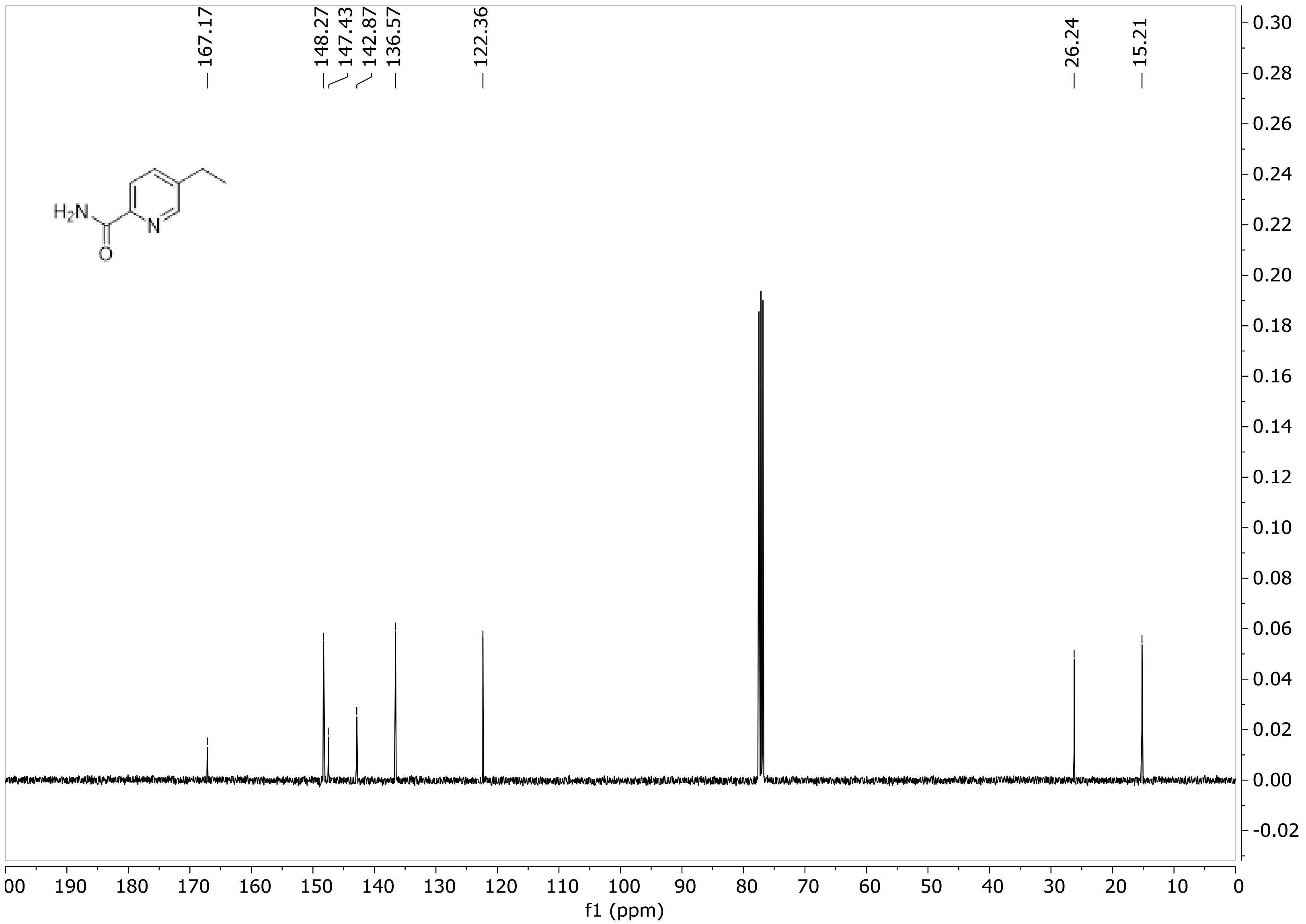

Figure S57. ^{13}C NMR (101 MHz, $CDCl_3$, 298K) of .**70**.

5-Acetylpicolinamide **S9**

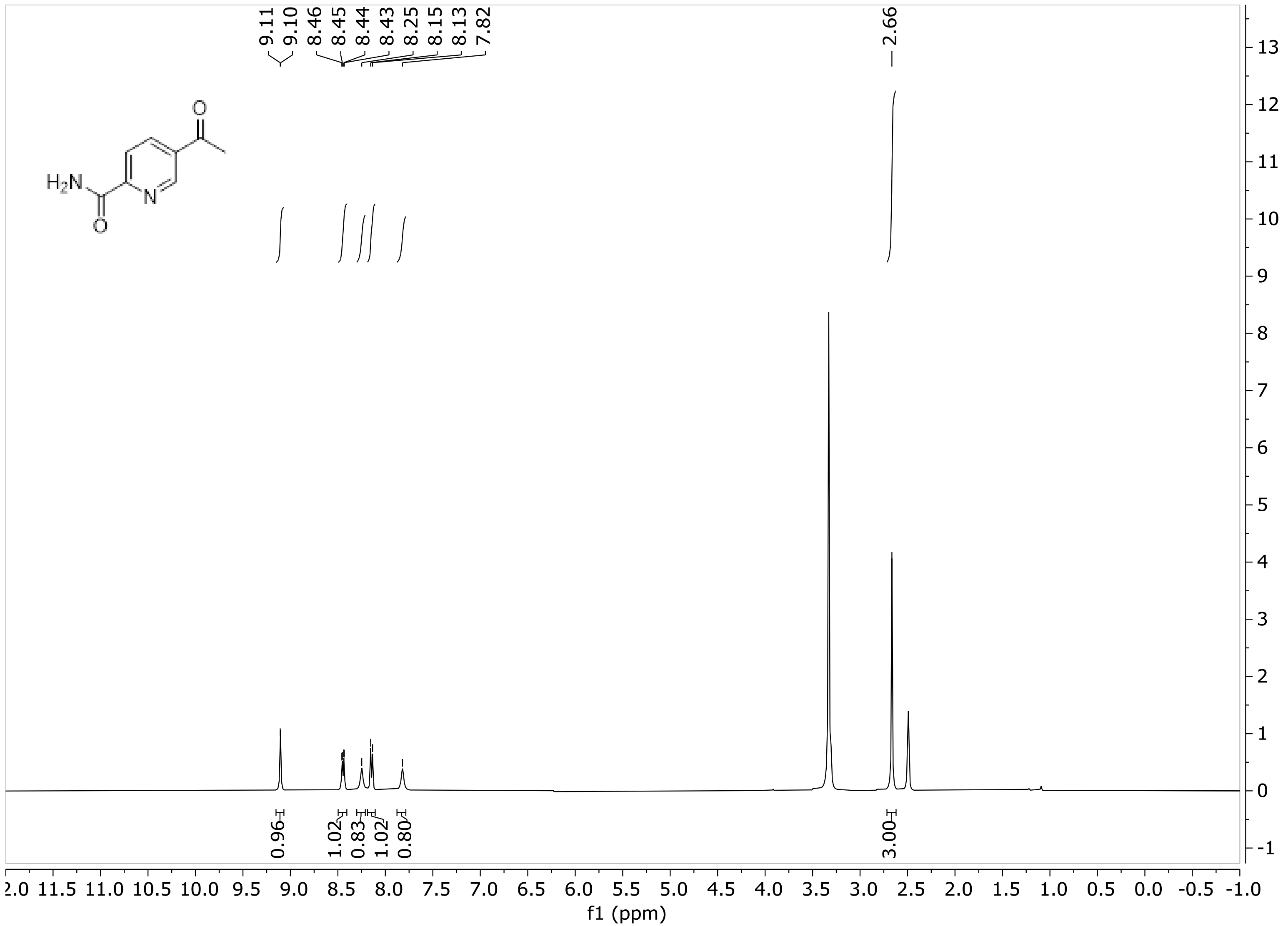

Figure S58. ^{1}H NMR (400 MHz, DMSO-d_4, 298K) of **S9**.

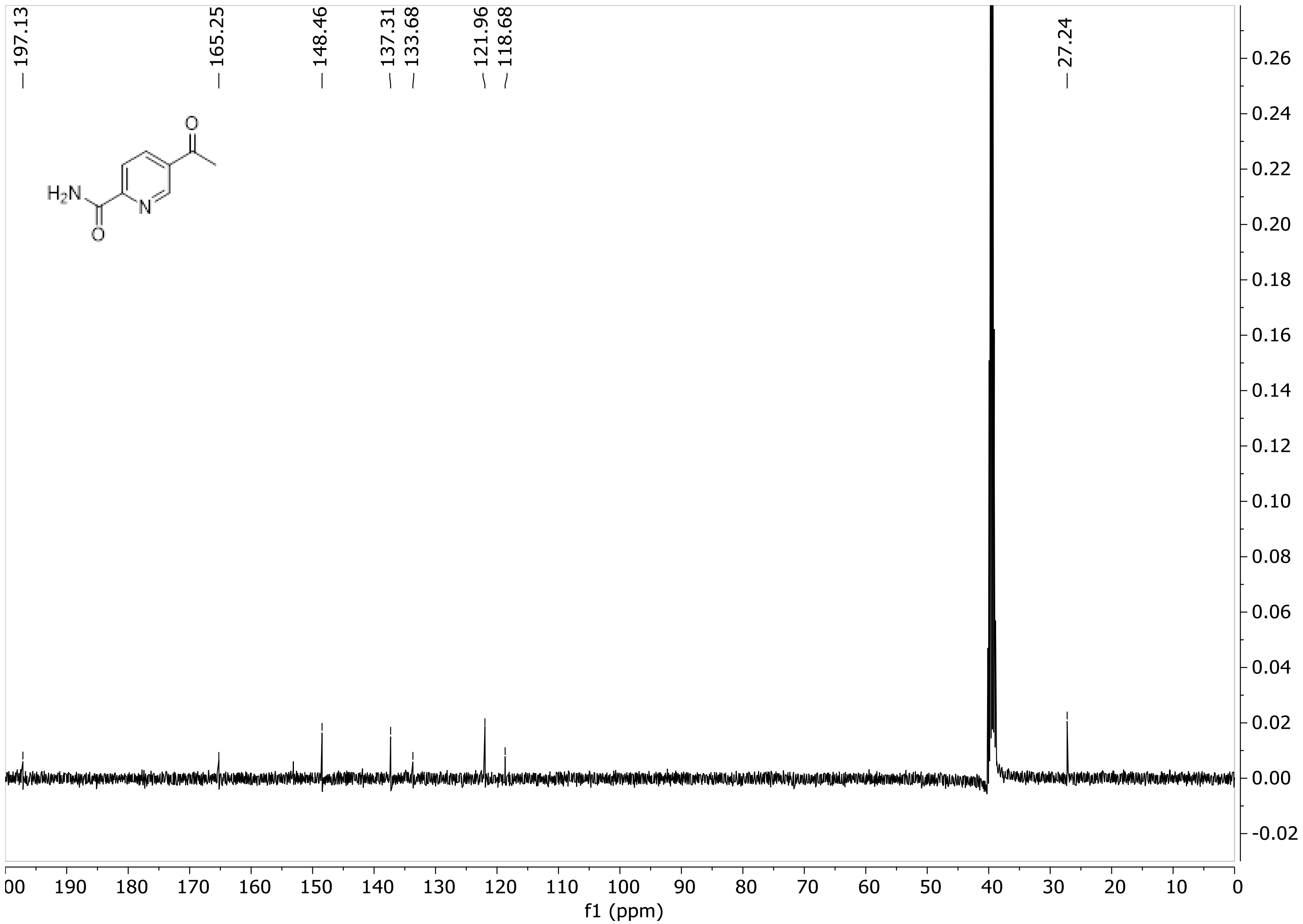

Figure S59. ^{13}C NMR (101 MHz, DMSO-d_4, 298K) of **S9**.

*(+/-)-5-(1-Hydroxyethyl)-picolinamide rac-***74**

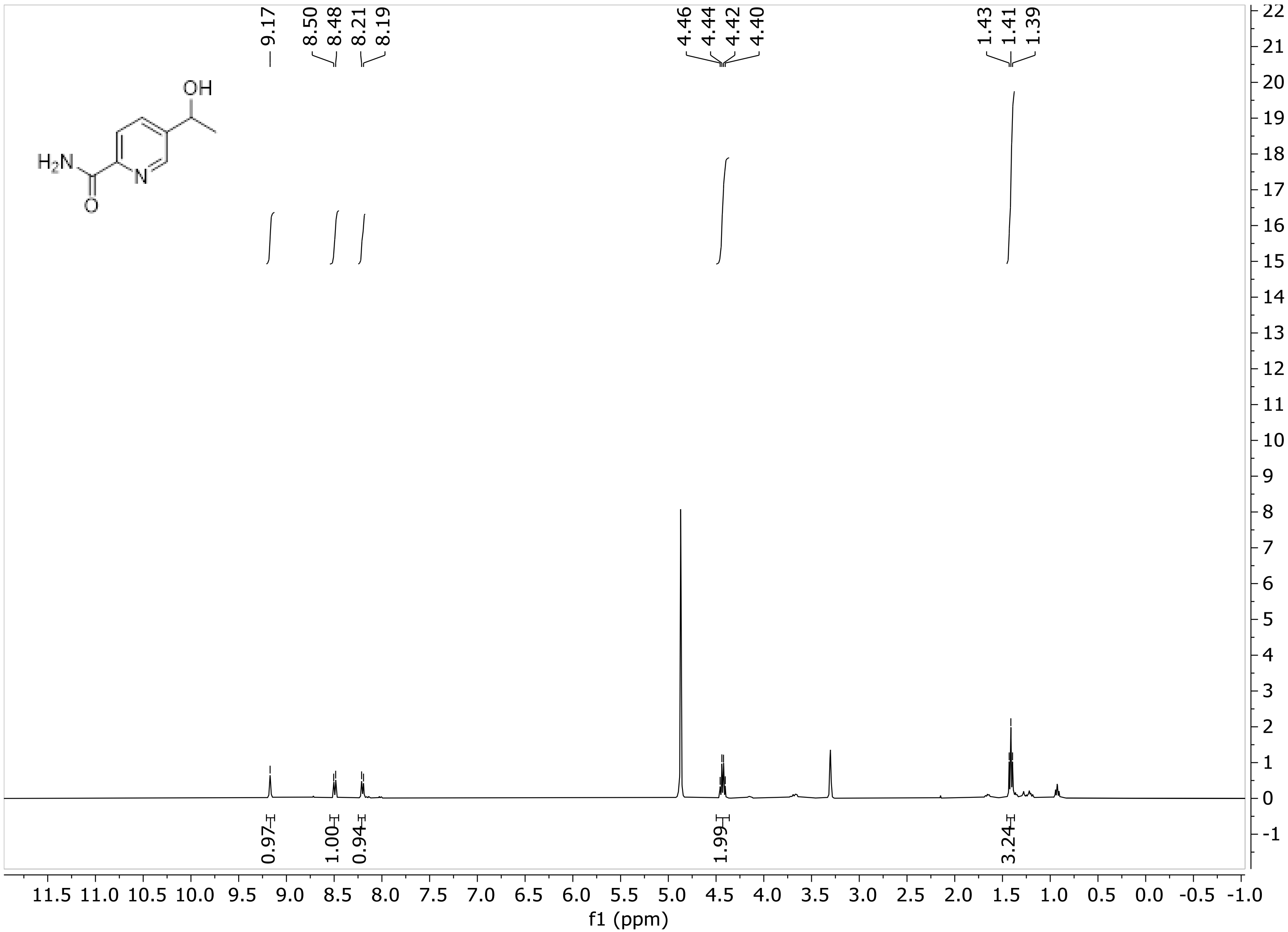

Figure S60. ^{1}H NMR (400 MHz, MeOH-d_4, 298K) of .*rac*-**74**.

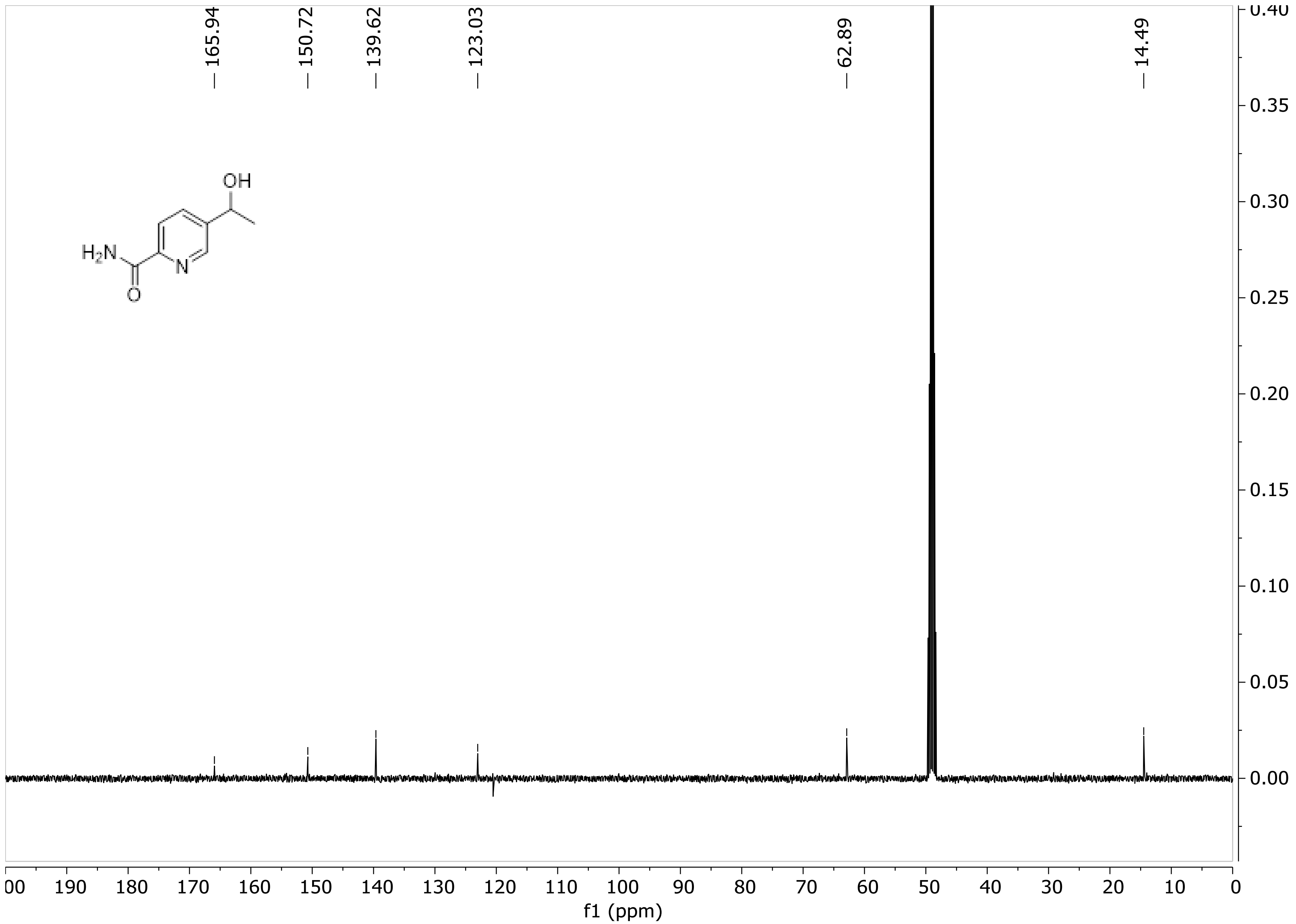

Figure 61. ^{13}C NMR (101 MHz, MeOH-d_4, 298K) of .*rac*-**74**.

5-Bromo-2-(methylthio)-pyridine **S10**

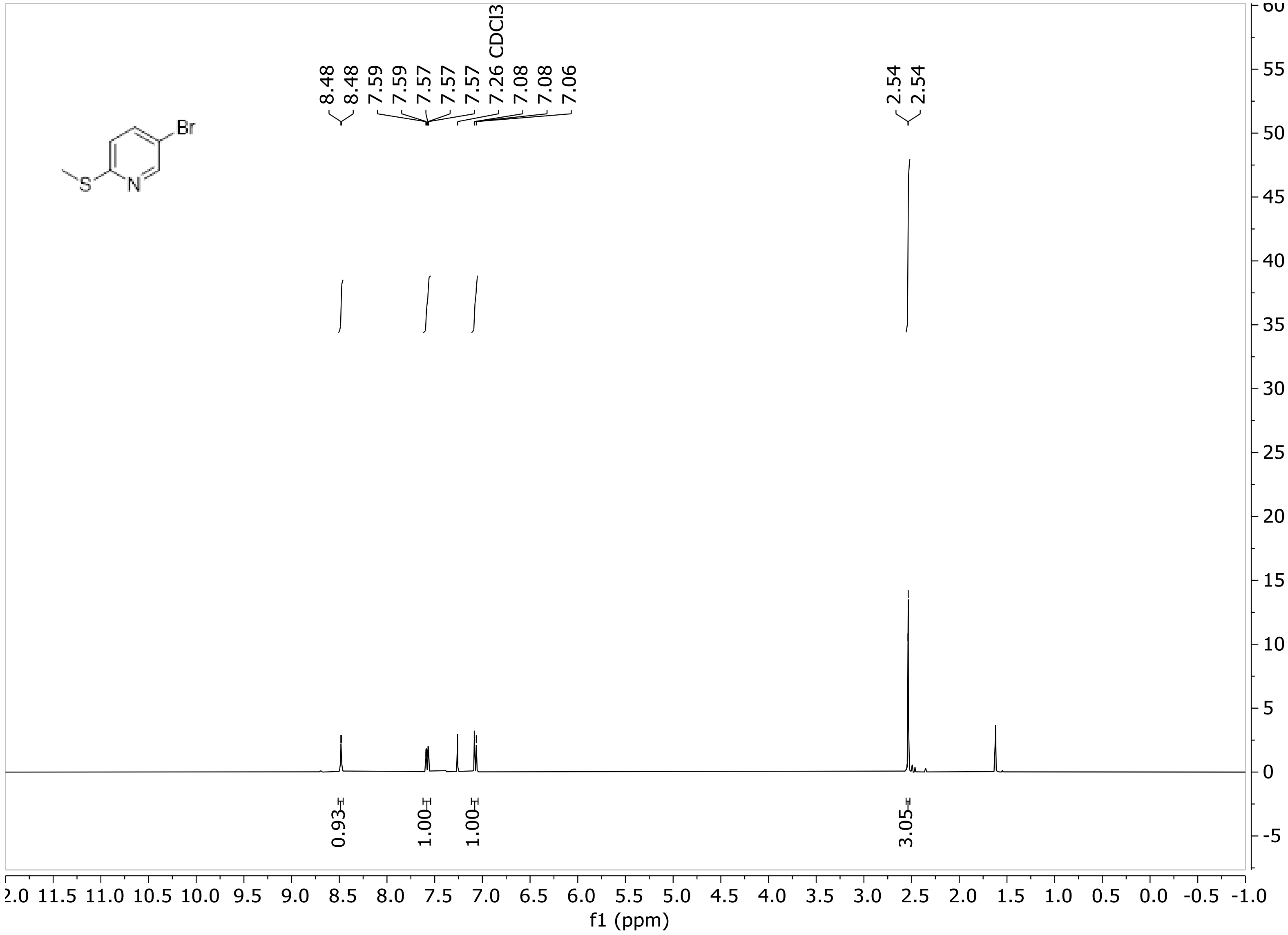

Figure S62. ^{1}H NMR (400 MHz, $CDCl_3$, 298K) of **S10**.

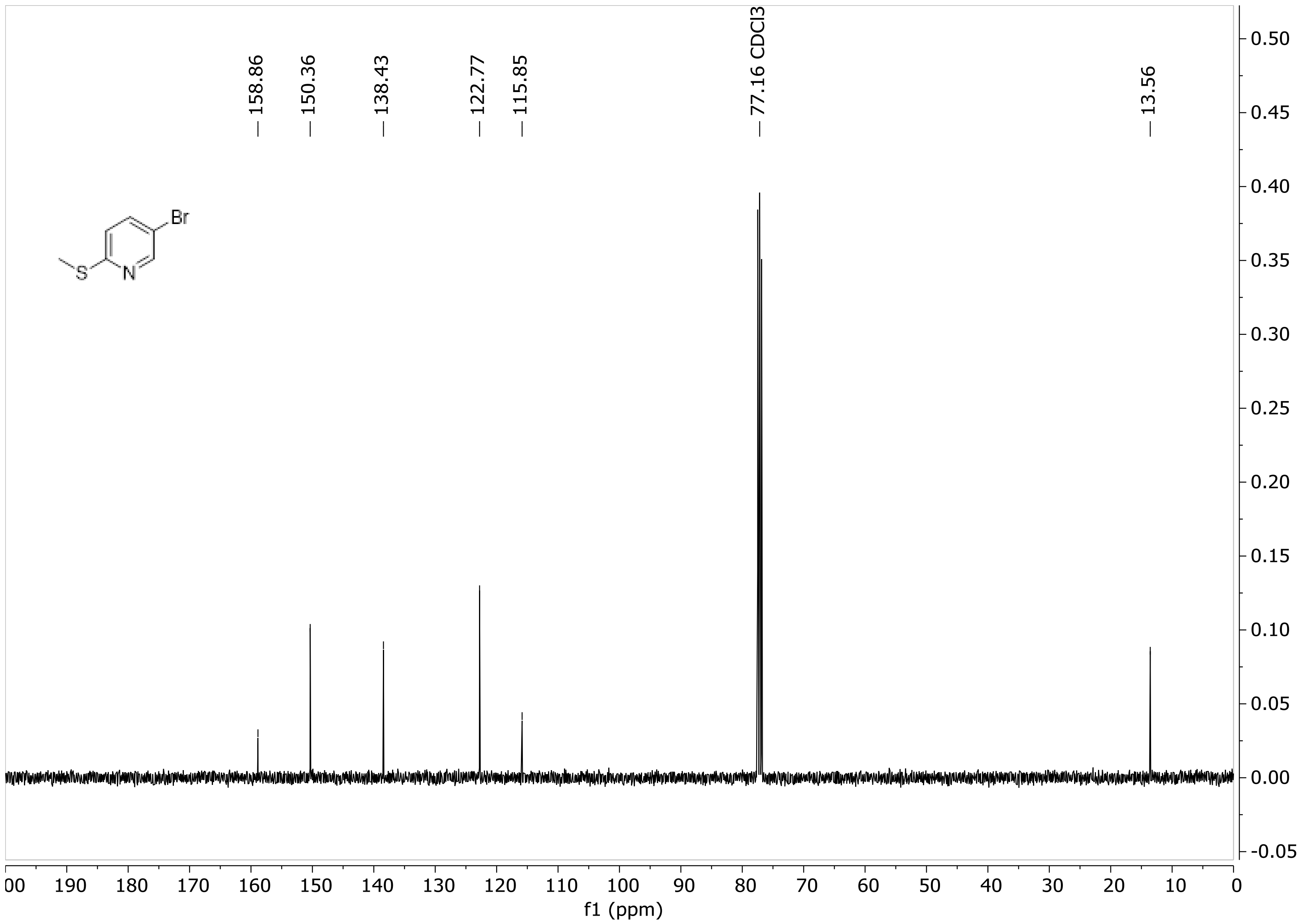

Figure S63. ^{1}H NMR (400 MHz, $CDCl_3$, 298K) of **S10**.

5-Bromo-2-(methylsulfonyl)-pyridine **S11**

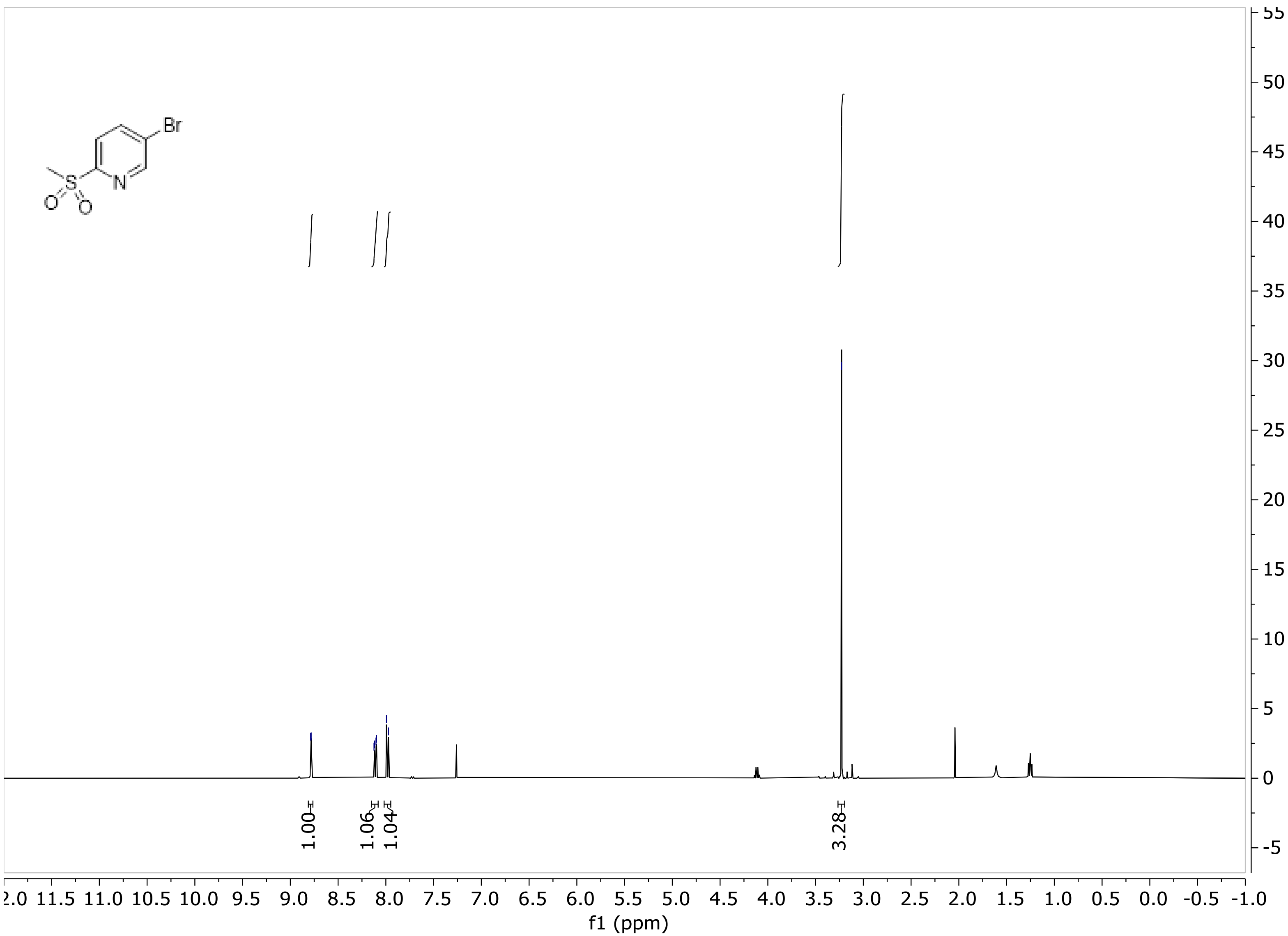

Figure S64. ^{1}H NMR (400 MHz, $CDCl_3$, 298K) of **S11**.

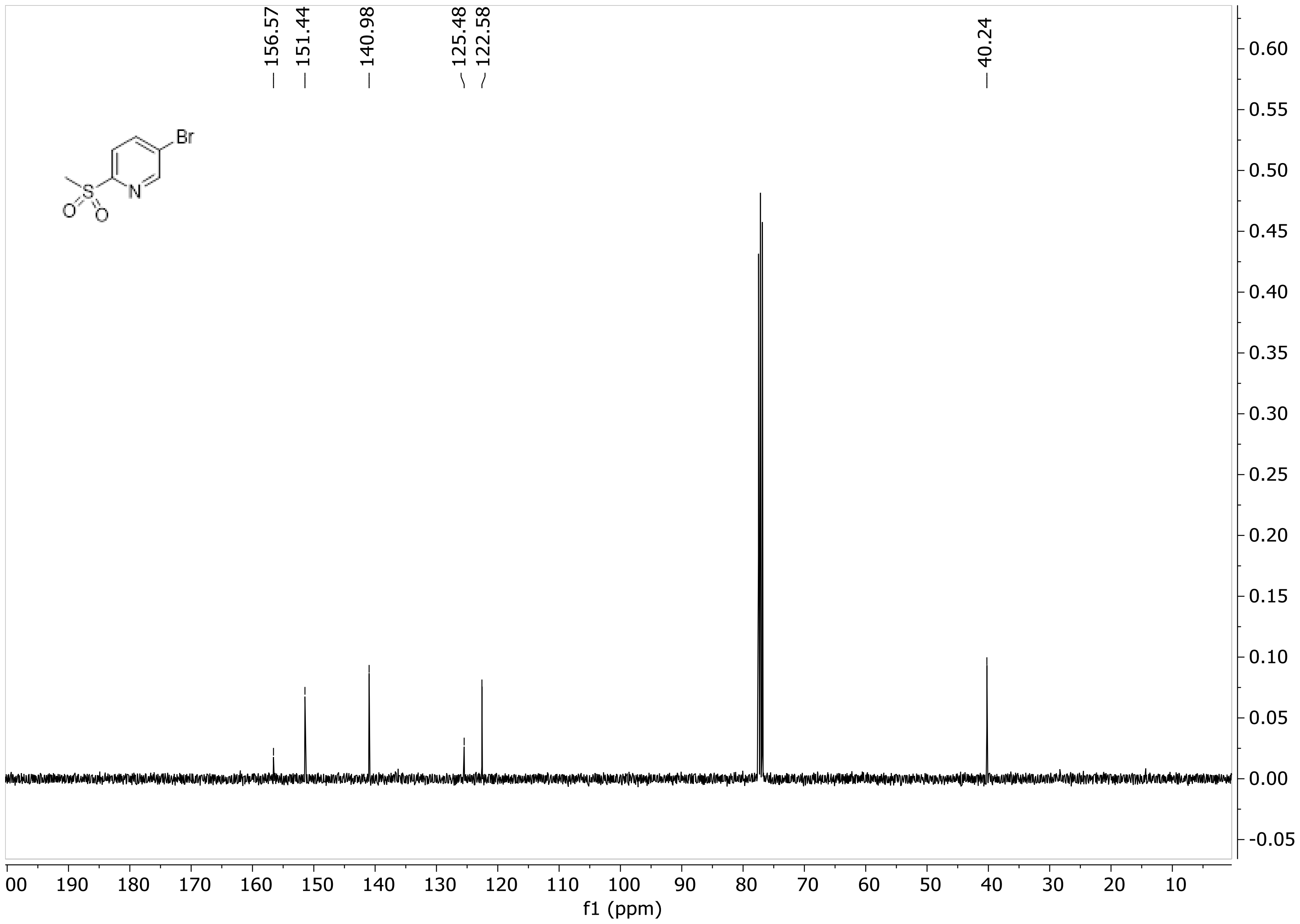

Figure S65. ^{13}C NMR (101 MHz, $CDCl_3$, 298K) of **S11**.

5-Ethyl-2-(methylsulfonyl)-pyridine **71**

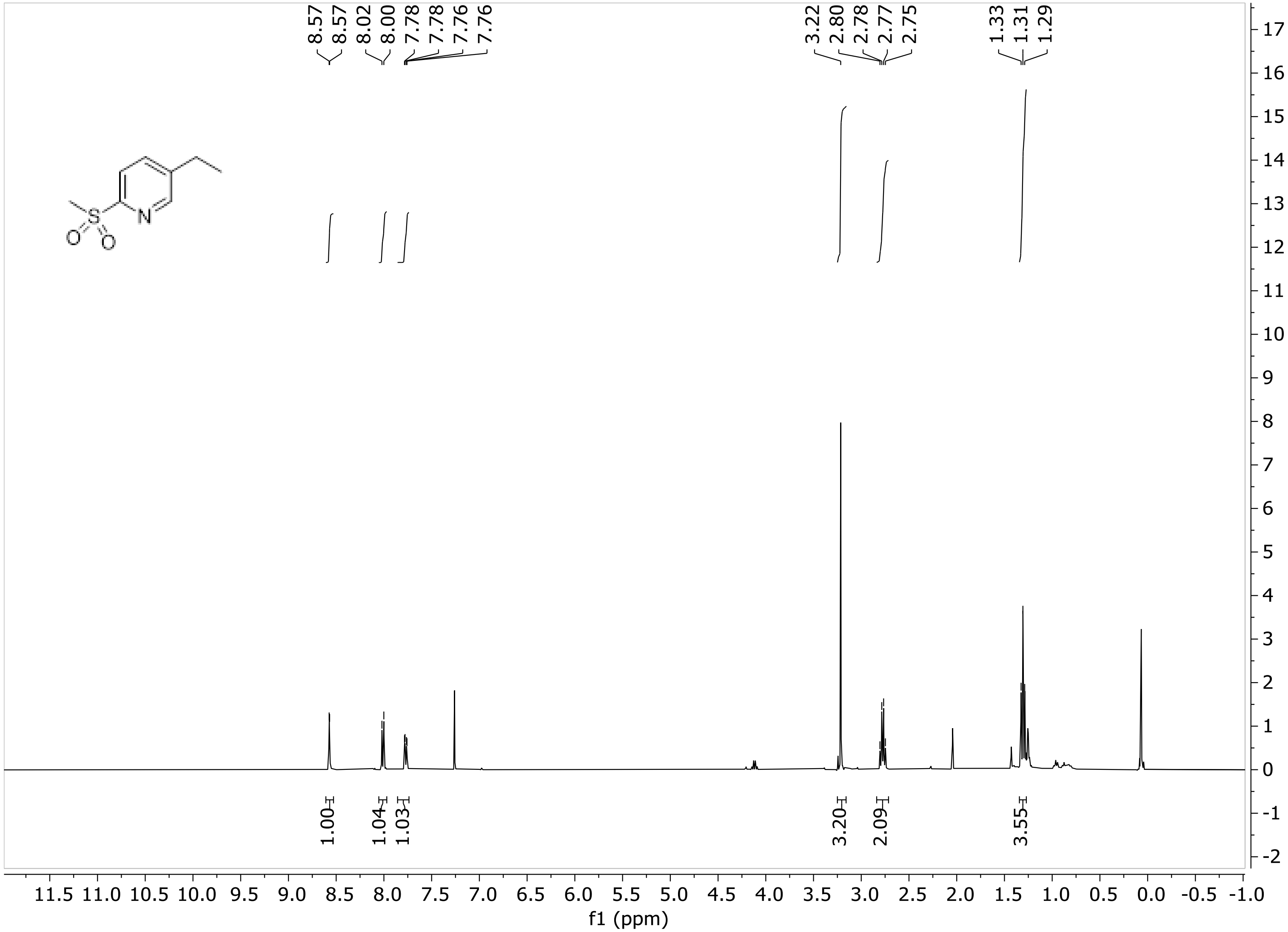

Figure S66. ^{1}H NMR (400 MHz, MeOH-d_4, 298K) of **71**.

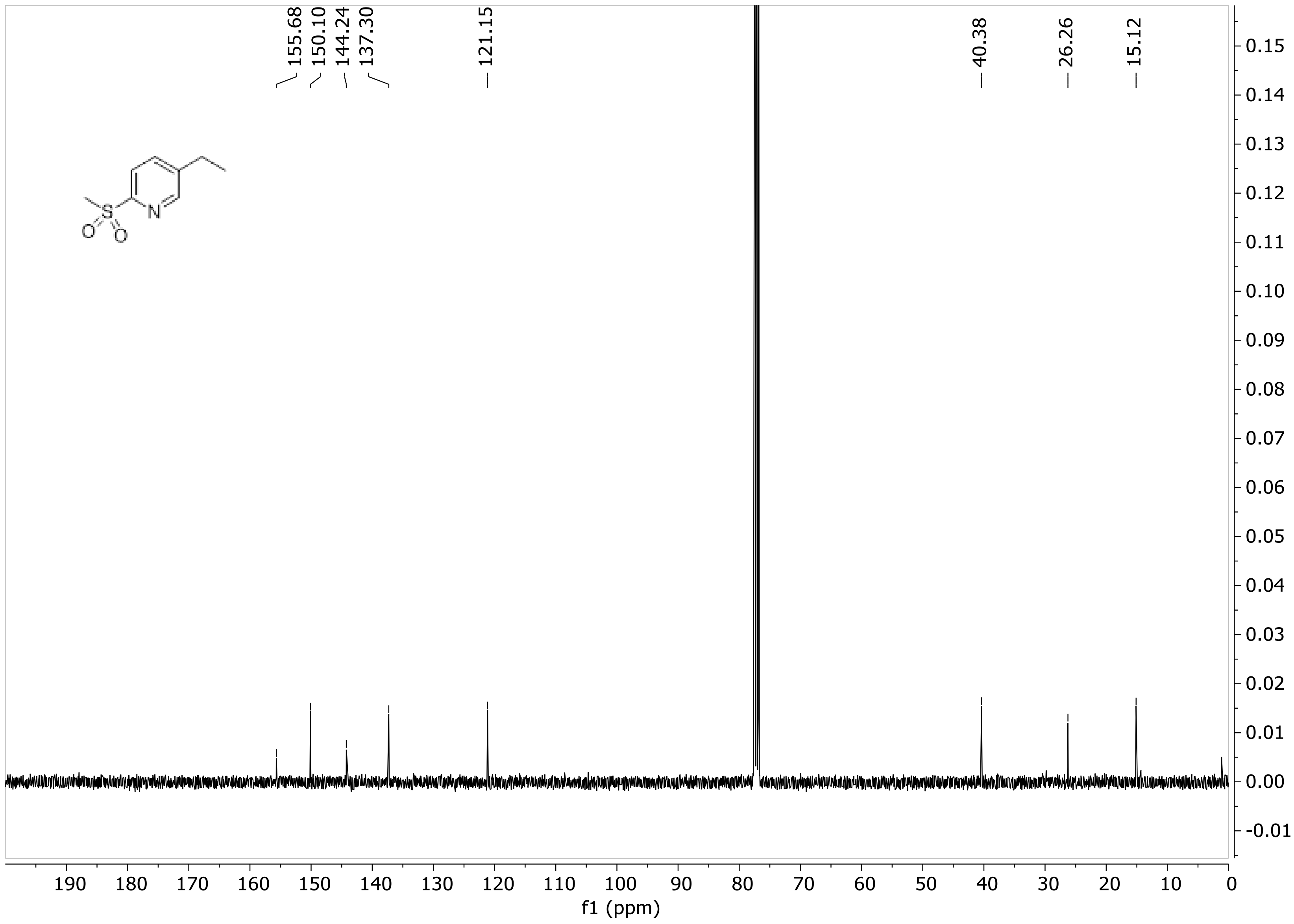

Figure S67. ^{13}C NMR (101 MHz, MeOH-d_4, 298K) of **71**.

N-methoxy-N-methylacetamide **S12**

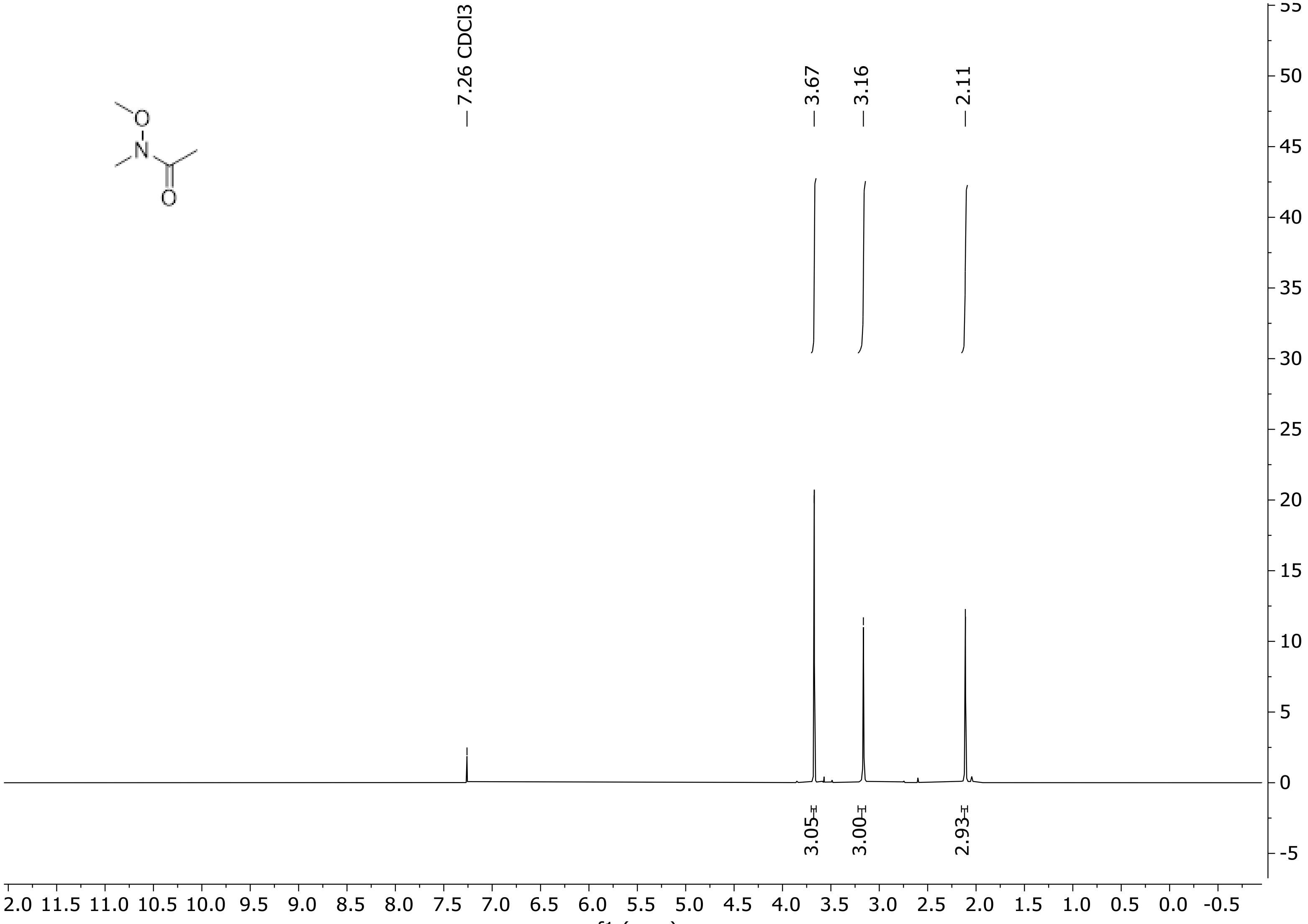

Figure S68. ^{1}H NMR (400 MHz, $CDCl_3$, 298K) of **S12**.

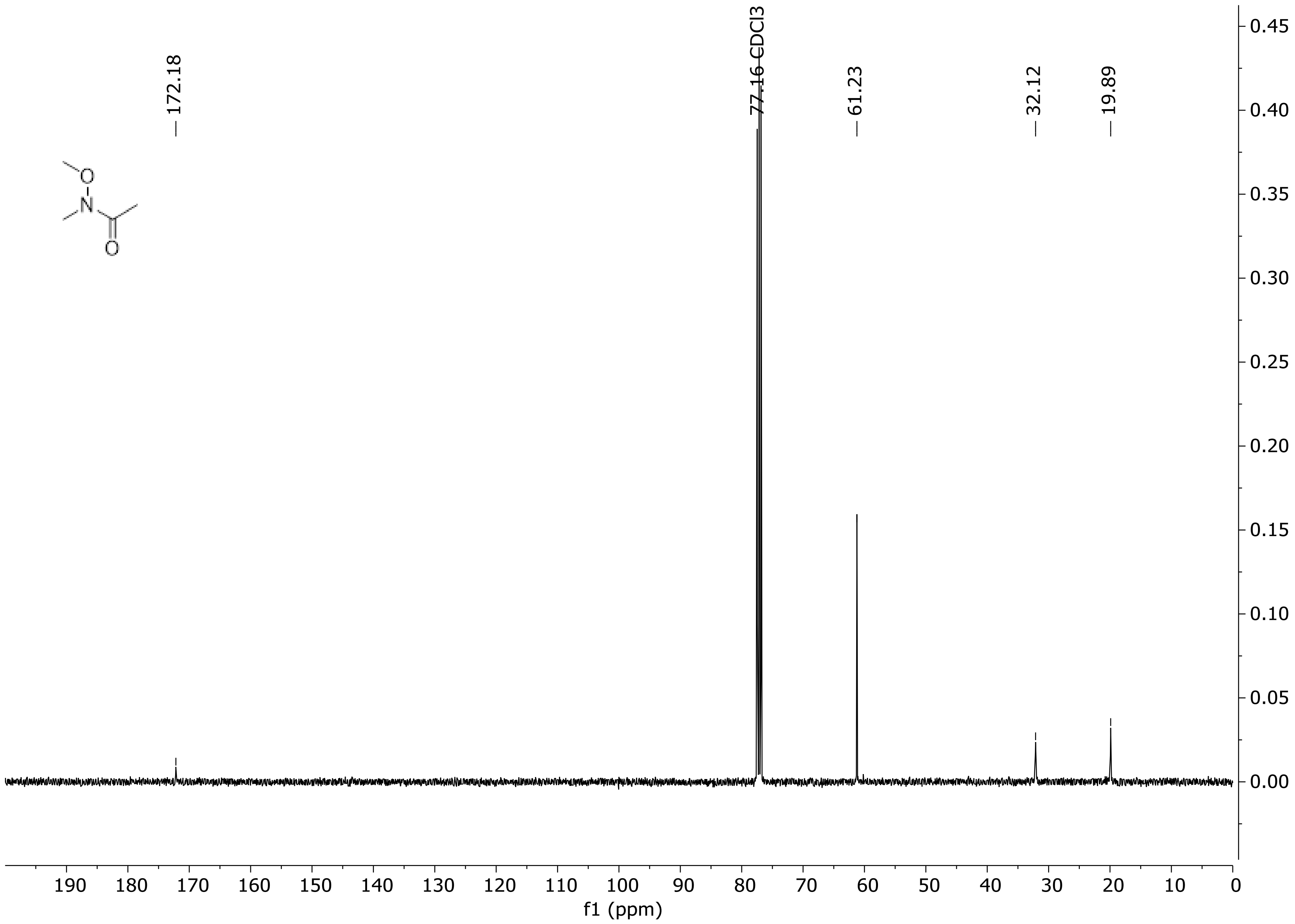

Figure S69. ^{13}C NMR (101 MHz, $CDCl_3$, 298K) of **S12**.

1-(6-Bromopyridin-3-yl)-ethan-1-one **S13**

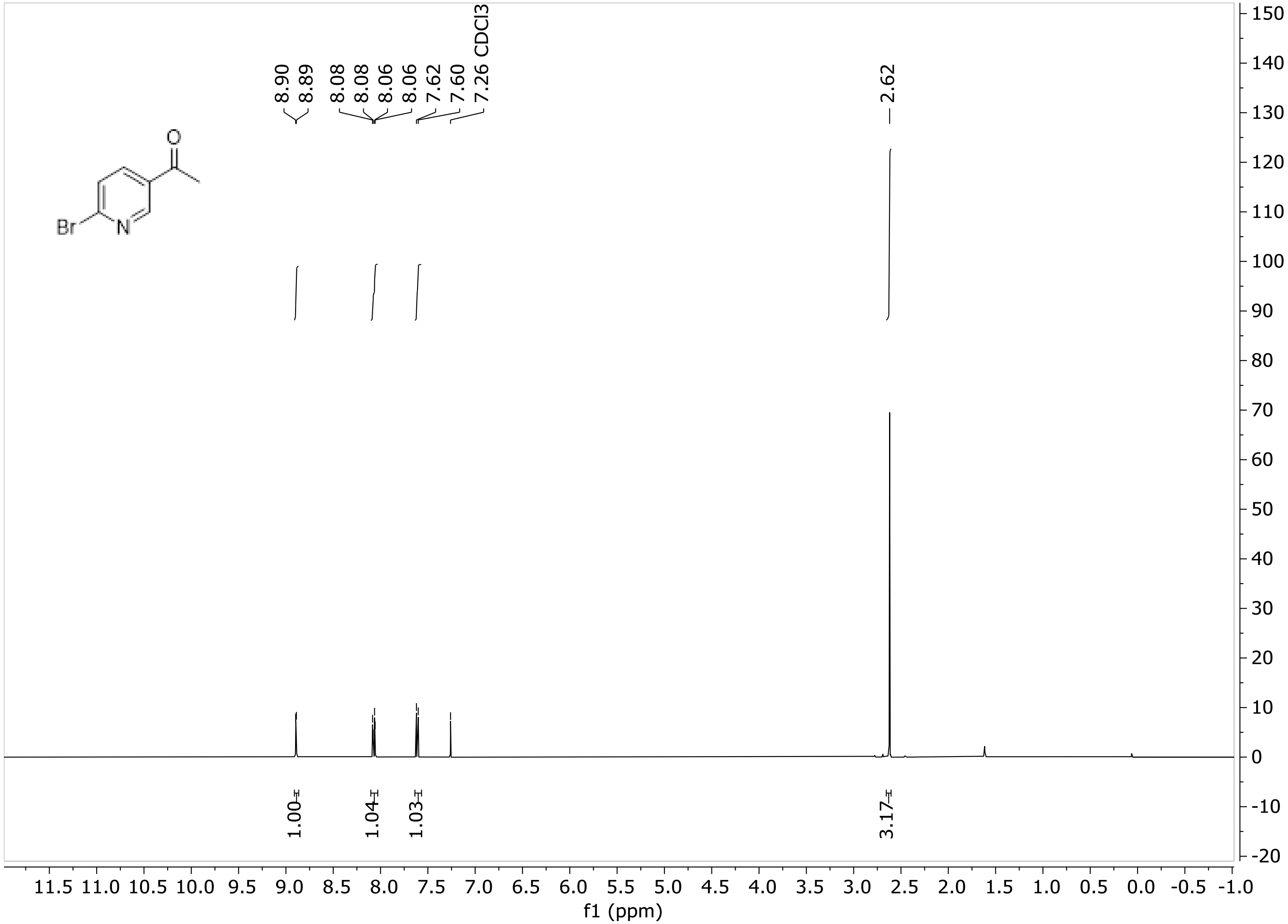

Figure S70 ^{1}H NMR (400 MHz, $CDCl_3$, 298K) of **S13**.

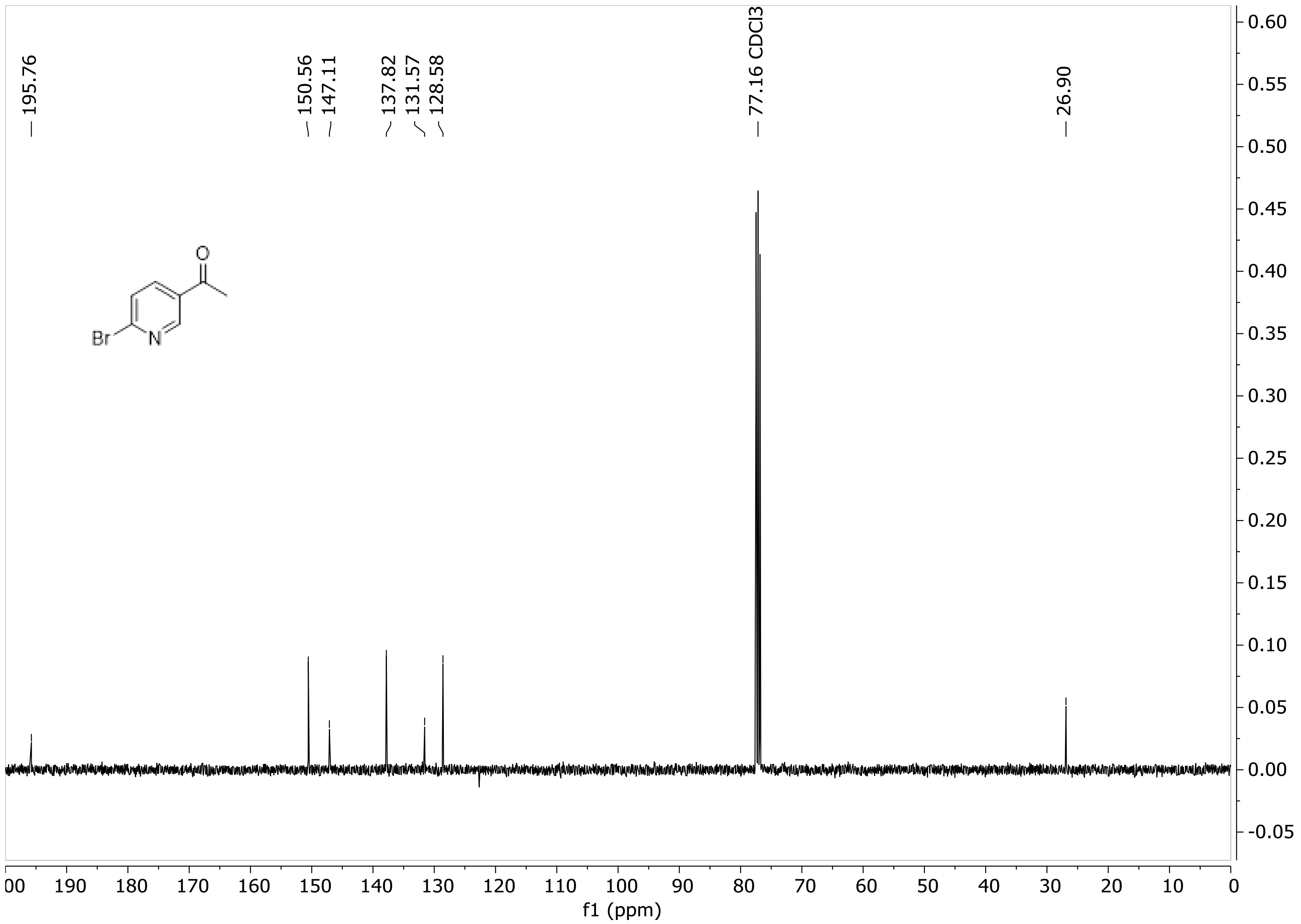

Figure S71. ^{13}C NMR (101 MHz, $CDCl_3$, 298K) of **S13**.

1-(6-(Methylsulfonyl)pyridin-3-yl)ethan-1-one **S14**

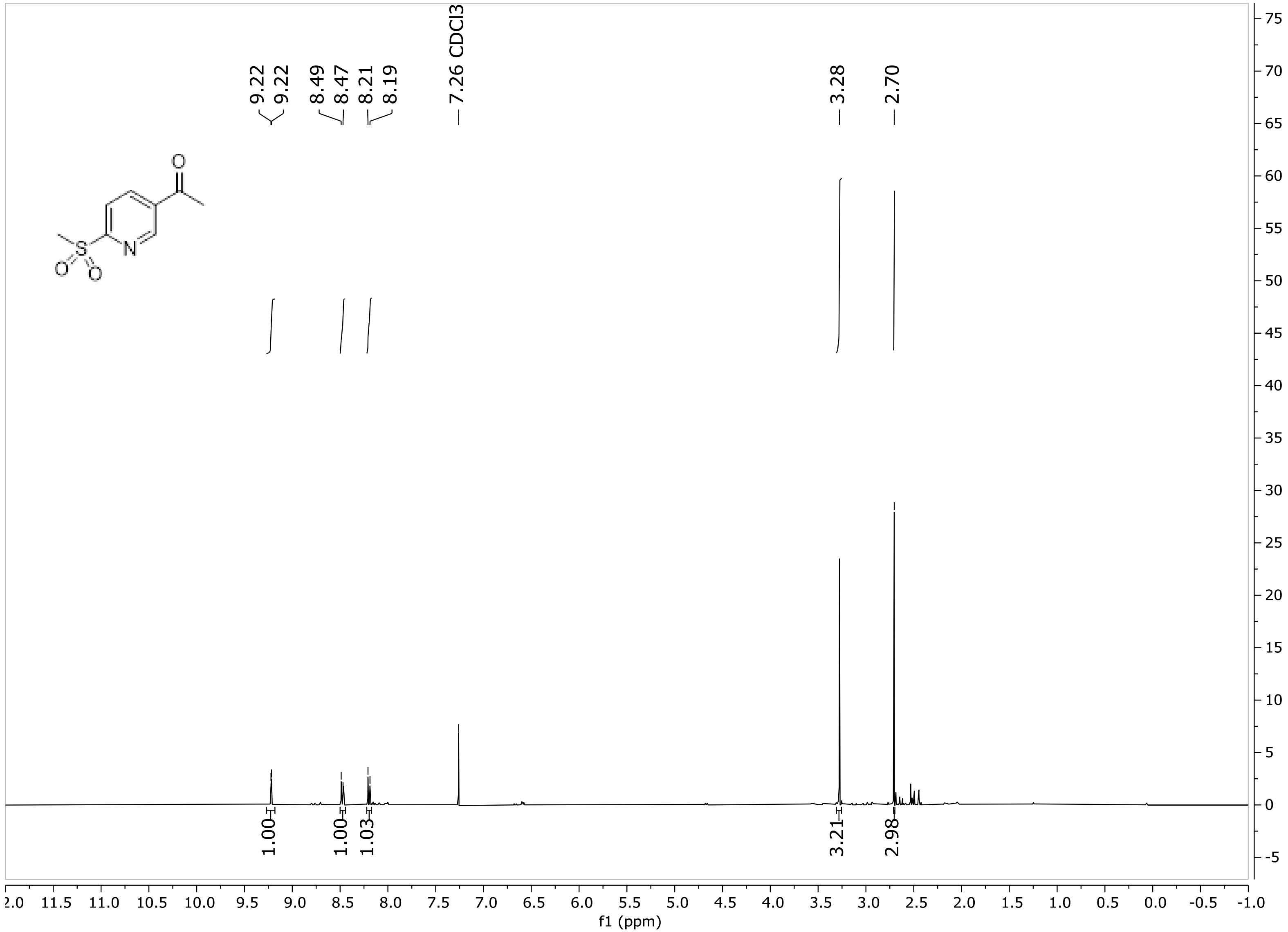

Figure S72. ¹H NMR (400 MHz, $CDCl_3$, 298K) of **S14**.

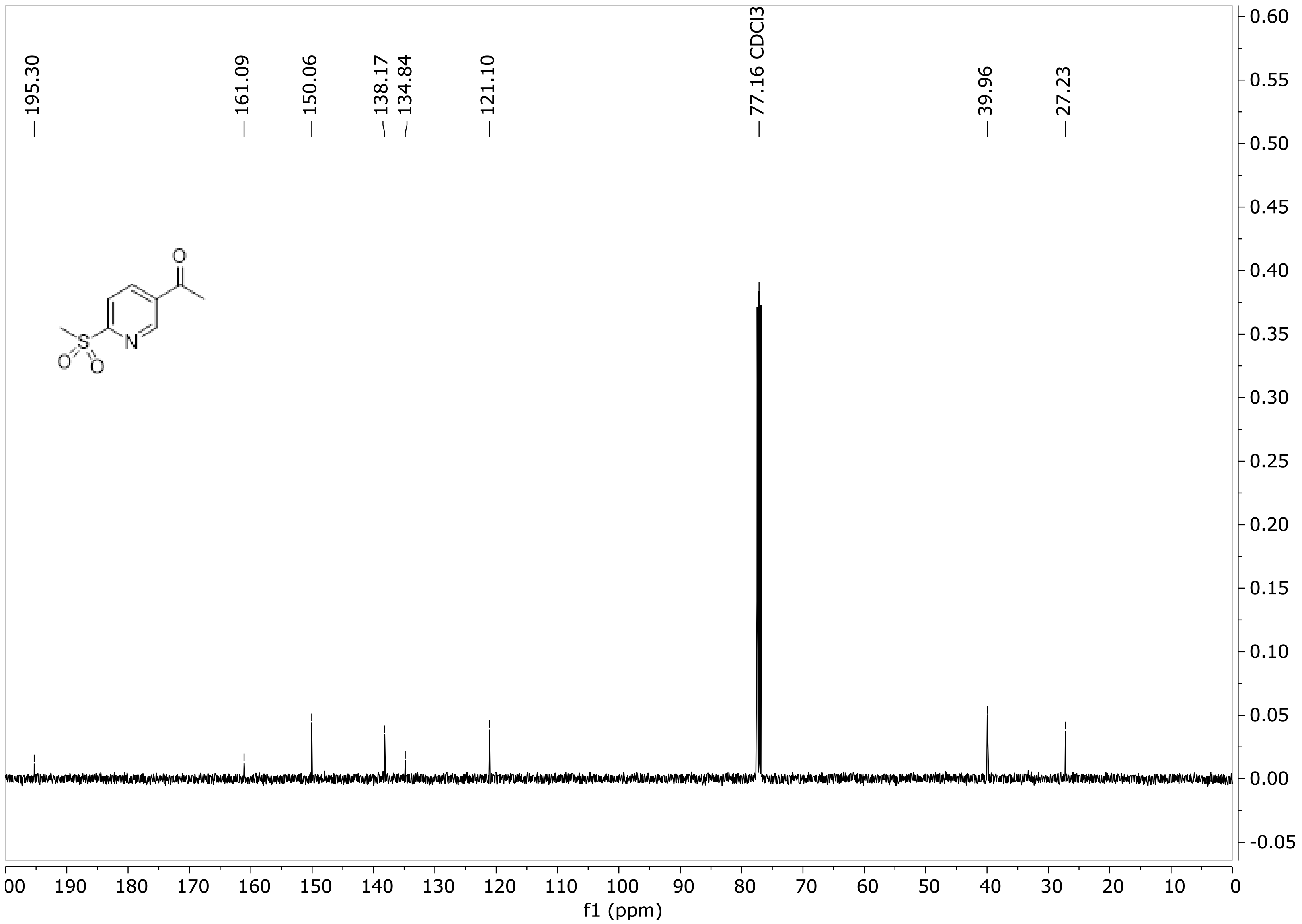

Figure S73. ^{13}C NMR (101 MHz, $CDCl_3$, 298K) of **S14**.

*(+/-)-1-(6-(Methylsulfonyl)pyridin-3-yl)-ethan-1-ol rac-***75**

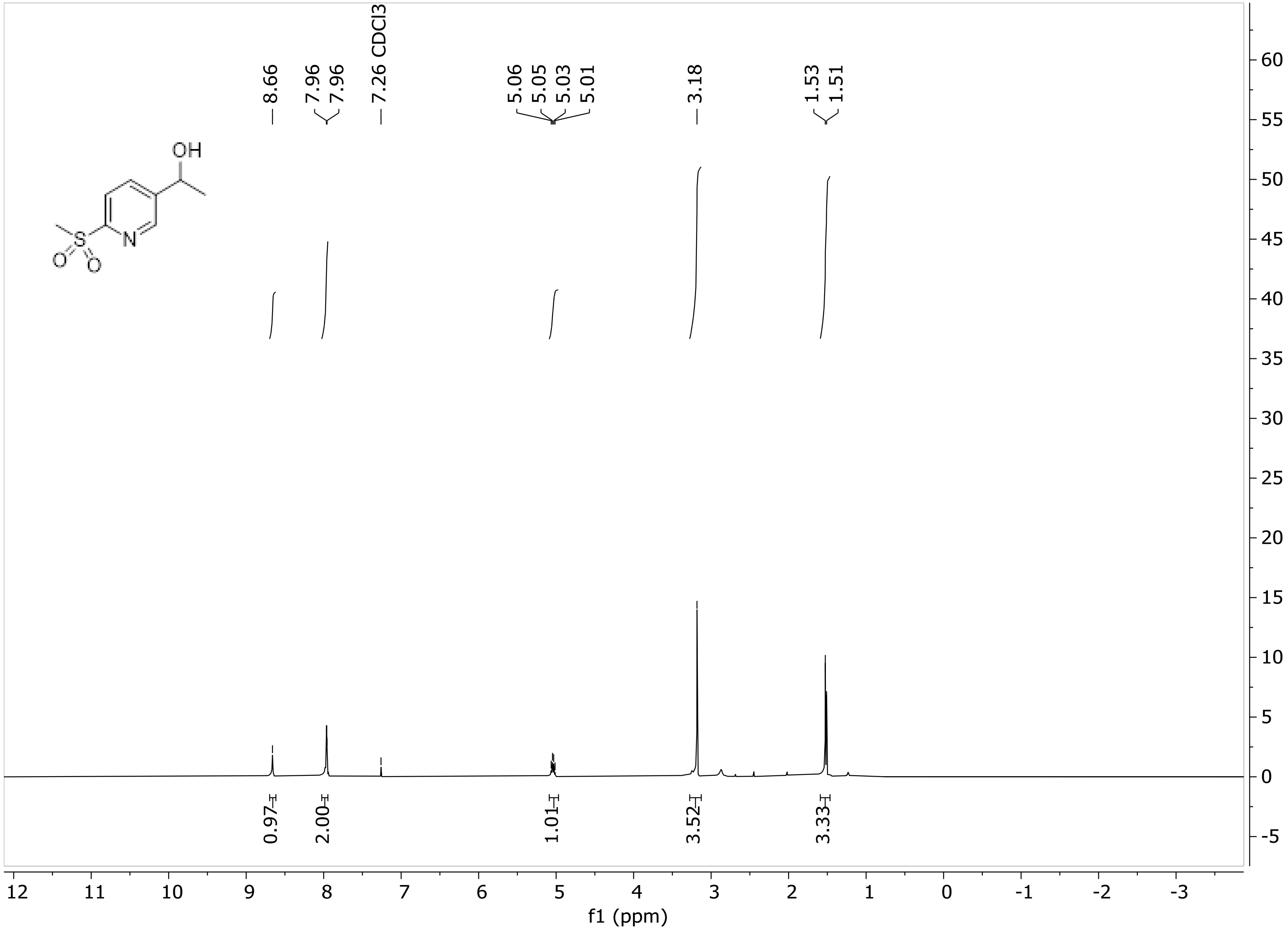

Figure S74. ^{1}H NMR (400 MHz, $CDCl_3$, 298K) of *rac*-**75**.

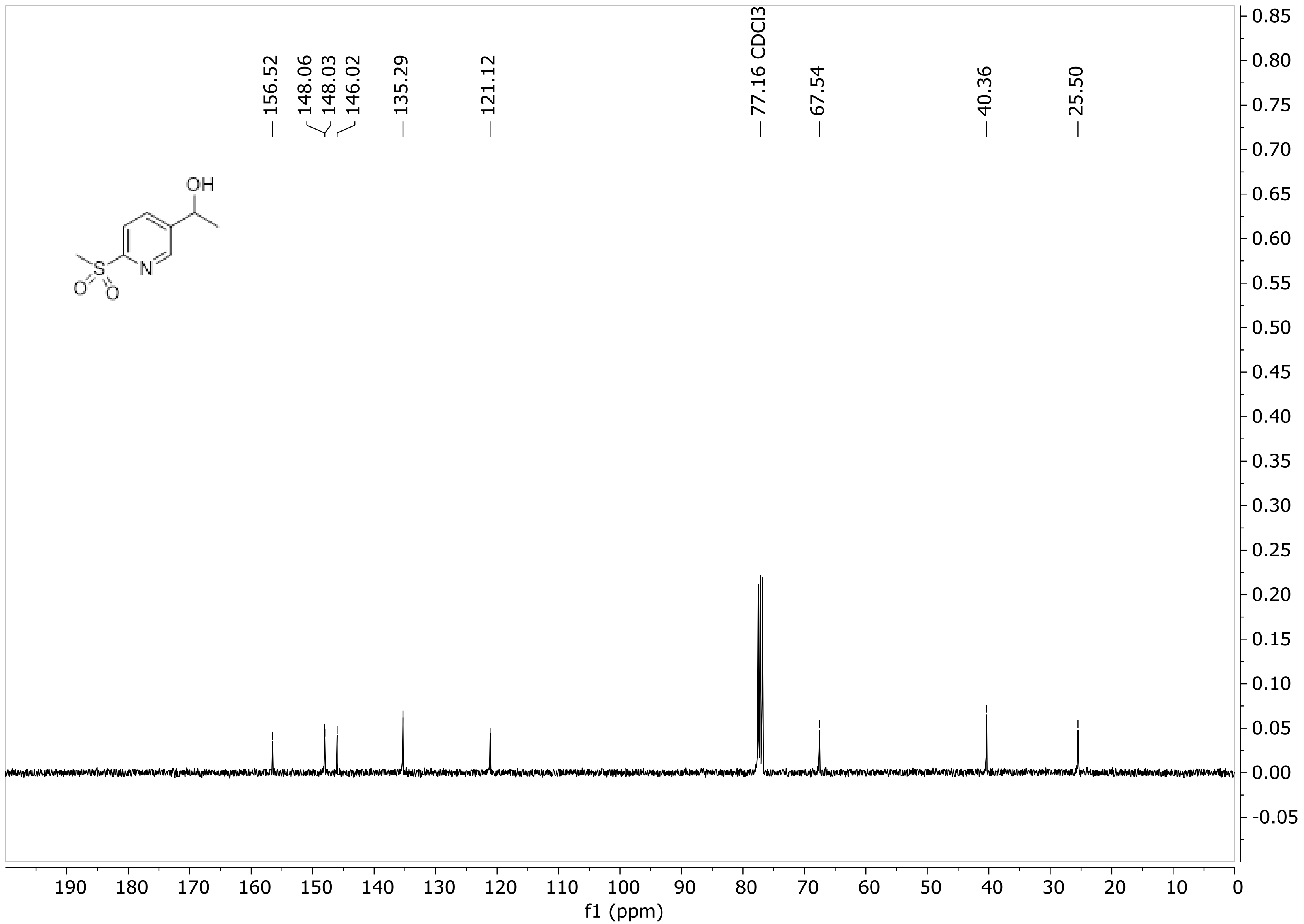

Figure S75. ^{13}C NMR (101 MHz, $CDCl_3$, 298K) of *rac*-**75**.

*5,6,7,8-tetrahydroquinolin-2(1*H*)-one* **S15**

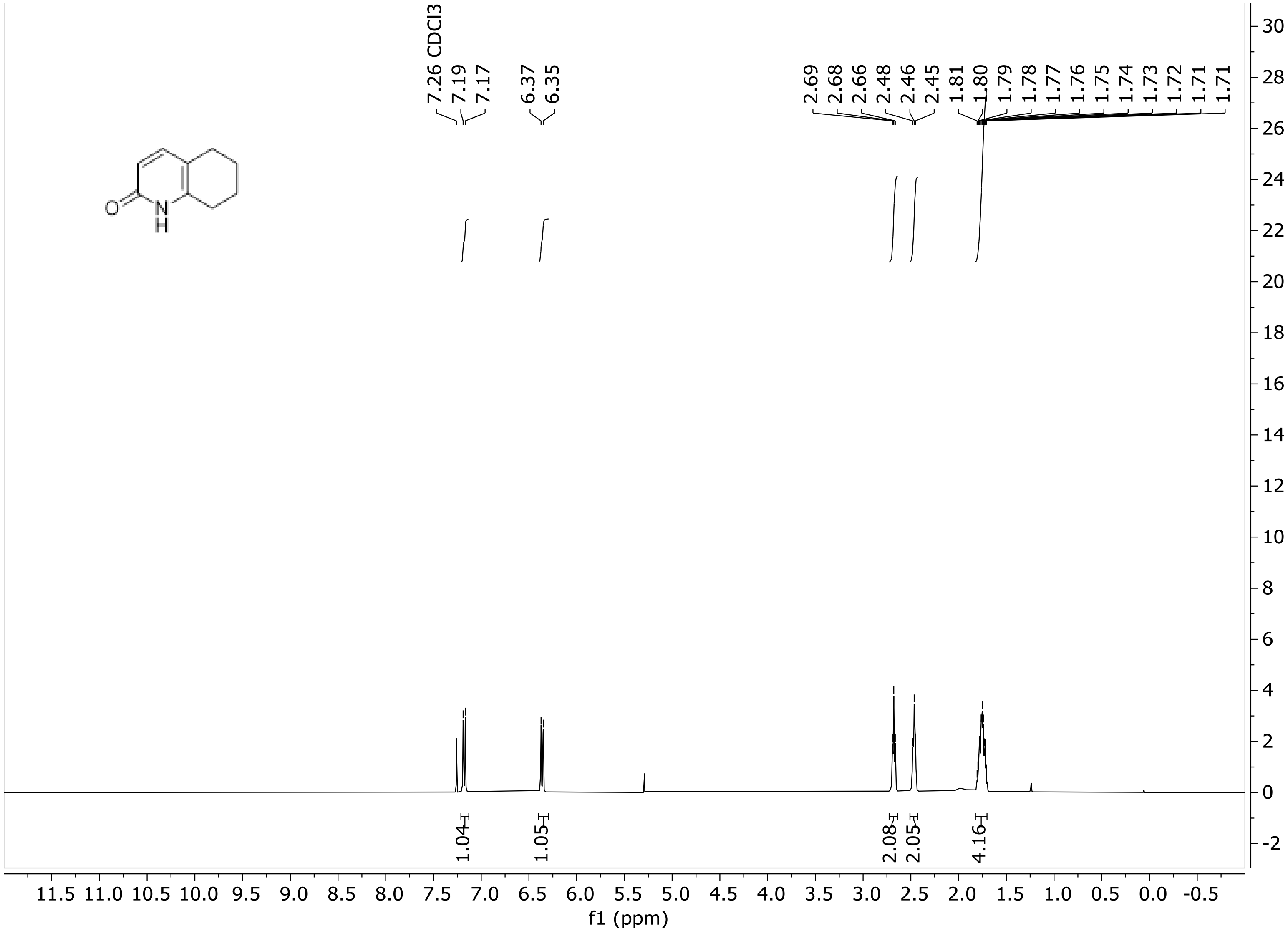

Figure S76. ^{1}H NMR (400 MHz, $CDCl_3$, 298K) of **S15**.

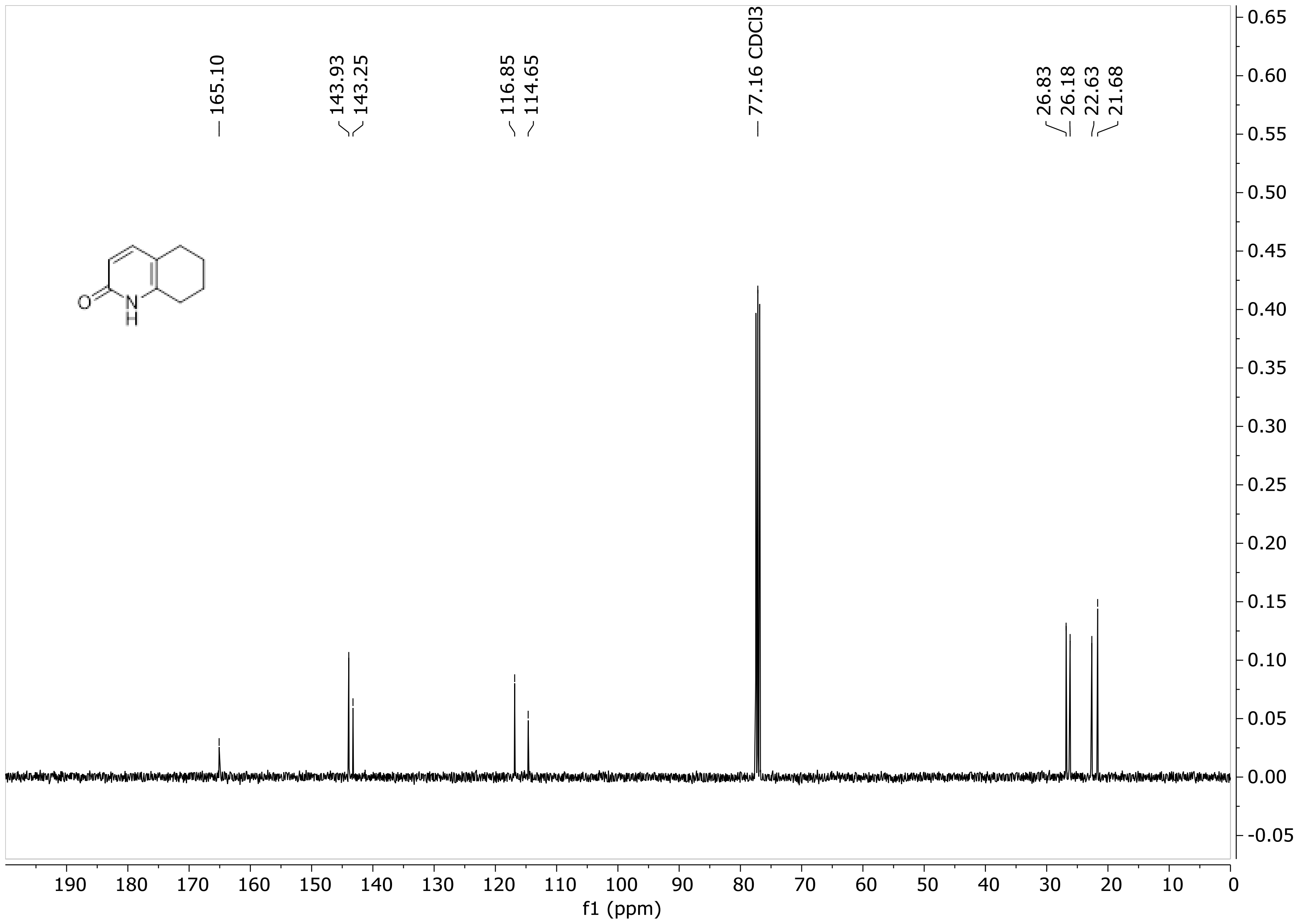

Figure S77. ^{13}C NMR (101 MHz, $CDCl_3$, 298K) of **S15**.

2-Bromo-5,6,7,8-tetrahydroquinoline **79**

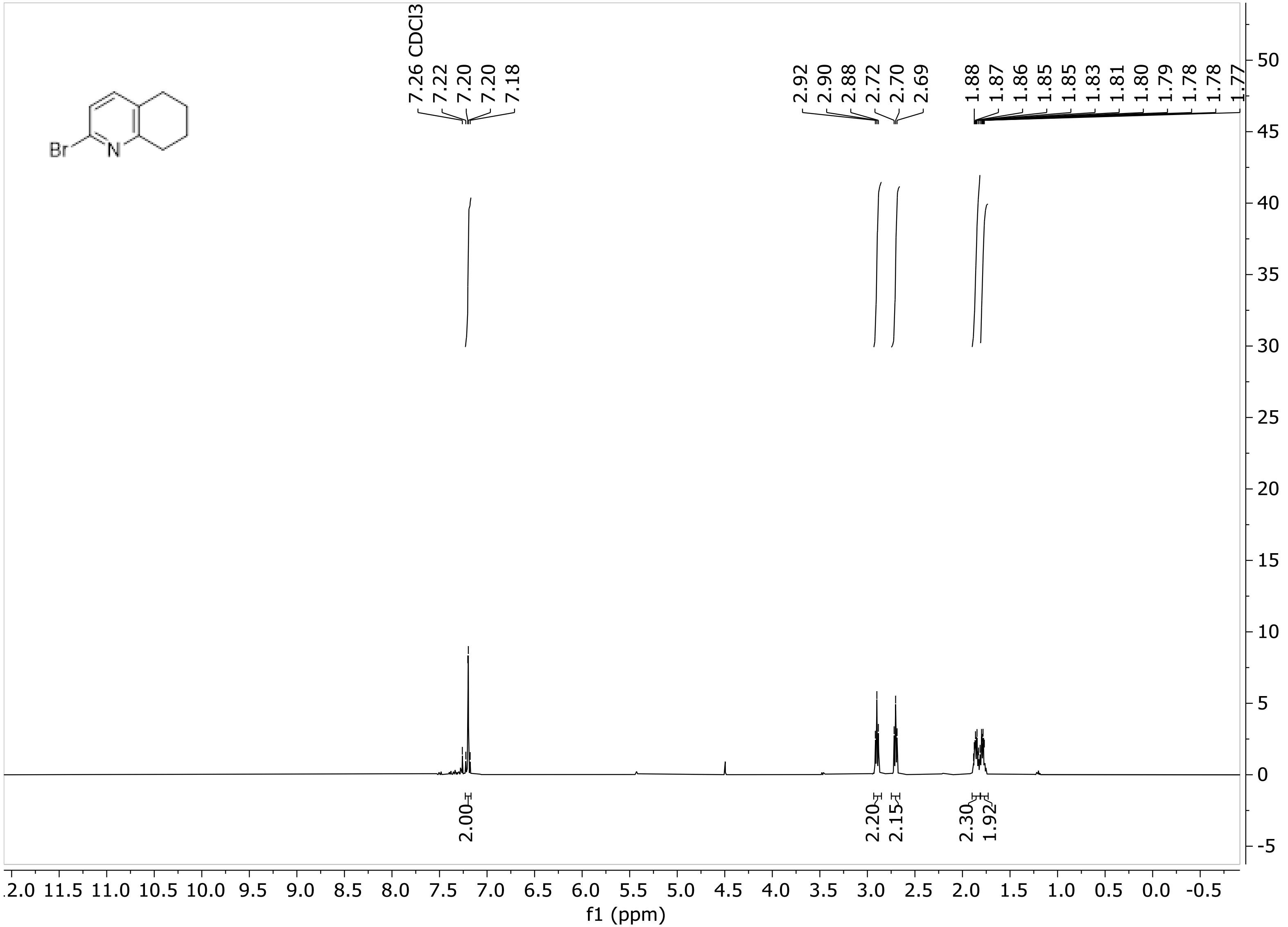

Figure S78. ^{1}H NMR (400 MHz, $CDCl_3$, 298K) of **79**.

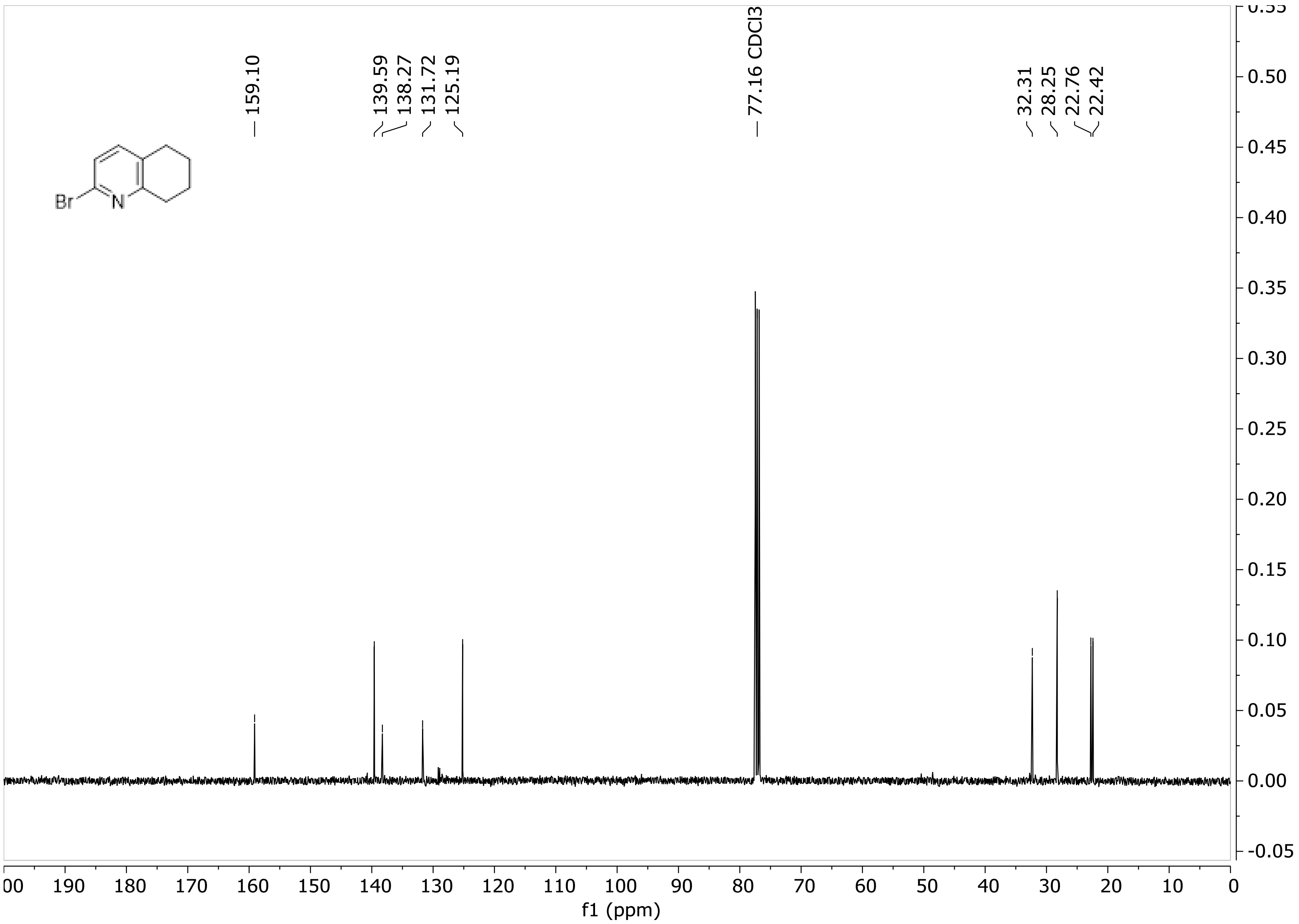

Figure S79. ^{13}C NMR (101 MHz, $CDCl_3$, 298K) of **79**.

2-Cyano-5,6,7,8-tetrahydroquinoline **81**

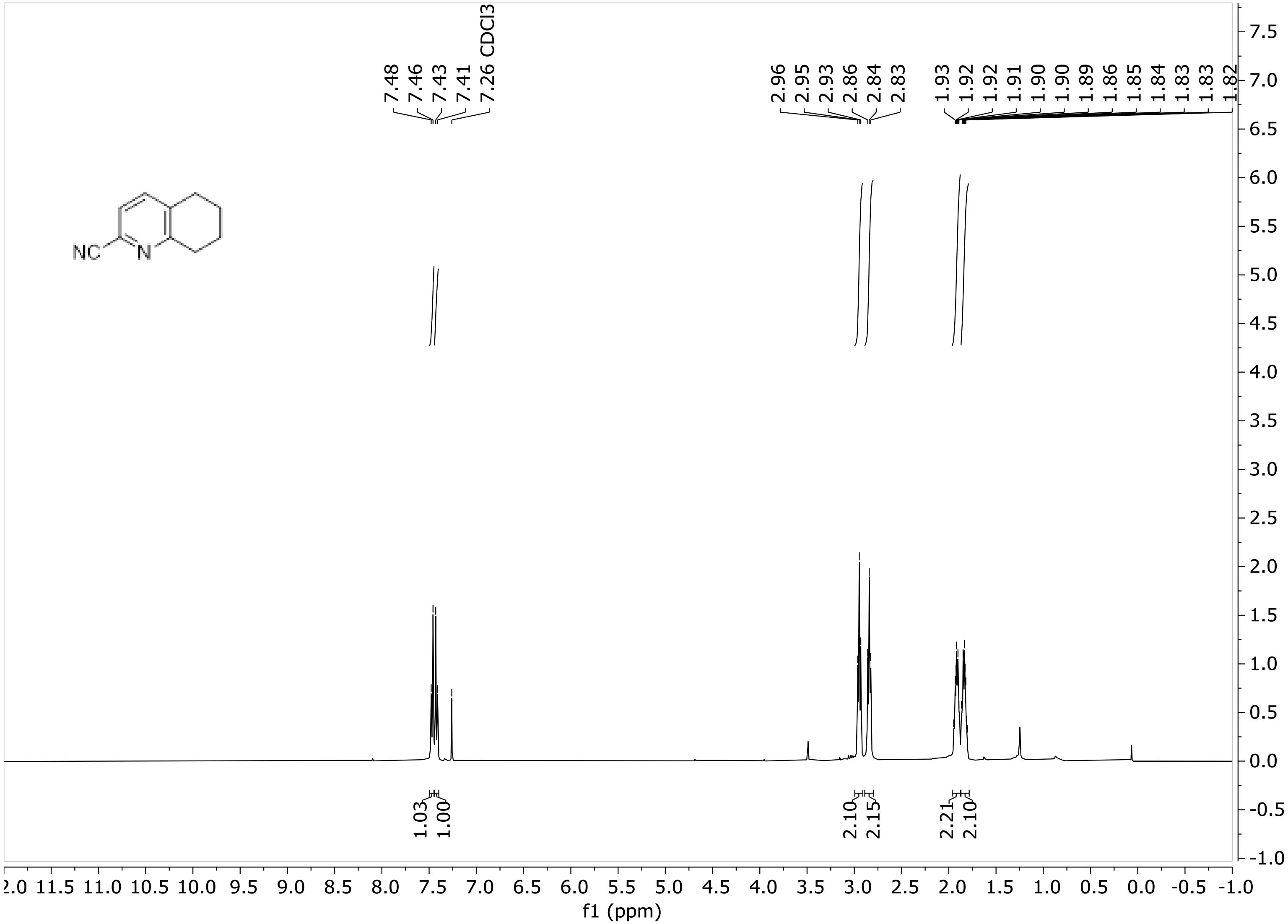

Figure S80. ^{1}H NMR (400 MHz, $CDCl_3$, 298K) of **81**.

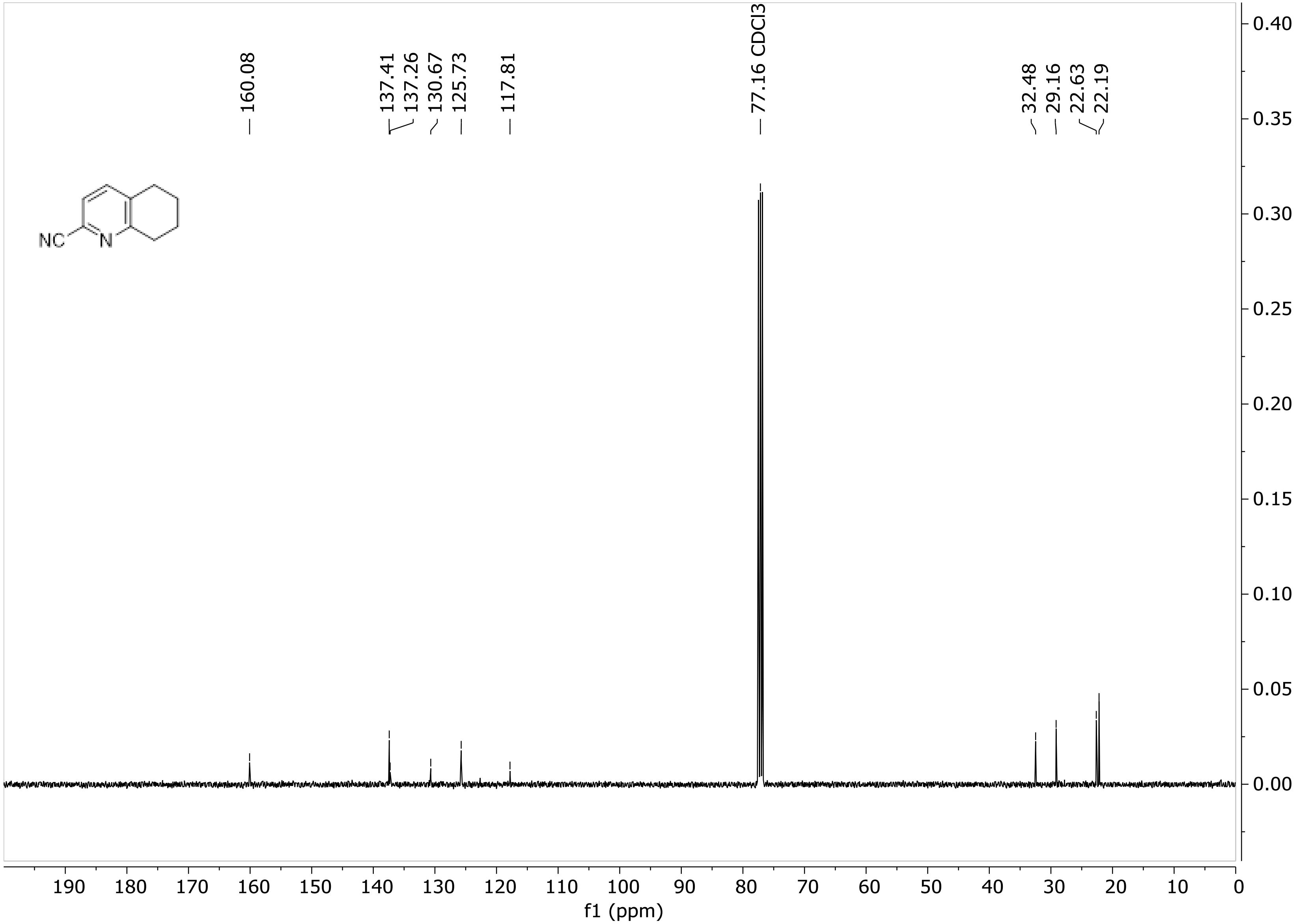

Figure S81. ^{13}C NMR (101 MHz, $CDCl_3$, 298K) of **81**.

2-(Methylsulfonyl)-5,6,7,8-tetrahydroquinoline **83**

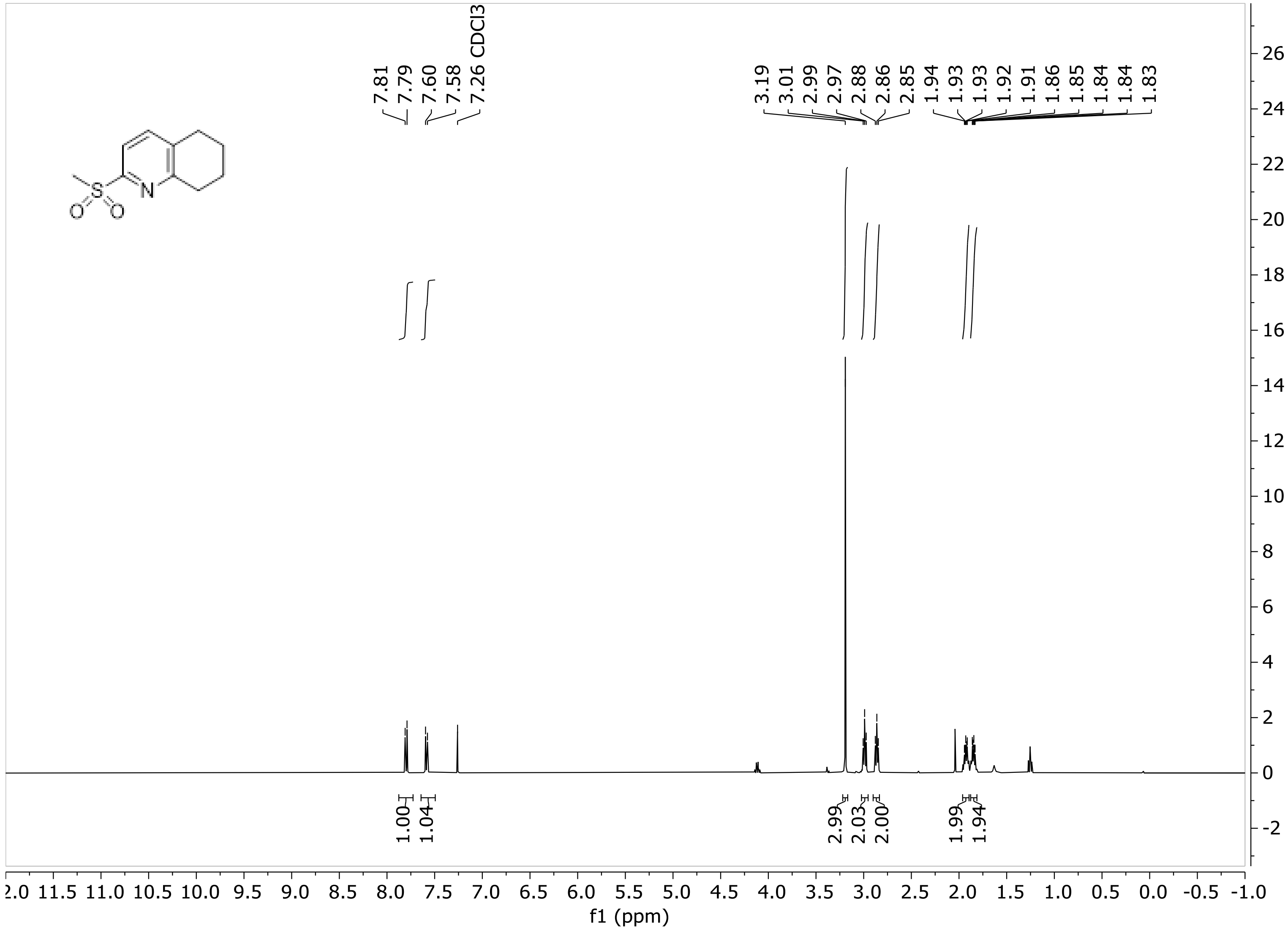

Figure S82. ^{1}H NMR (400 MHz, $CDCl_3$, 298K) of **83**.

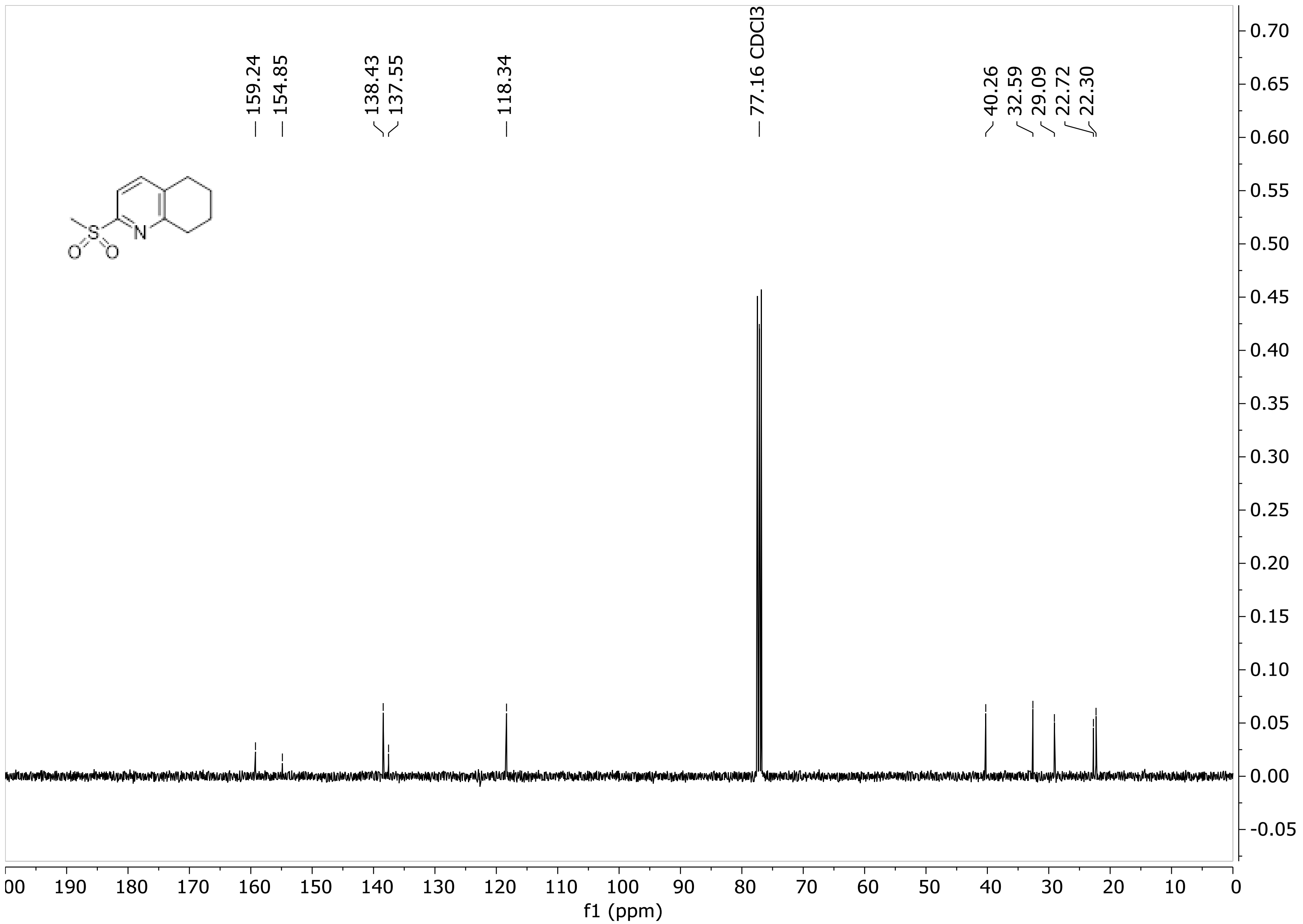

Figure S83. ^{13}C NMR (101 MHz, $CDCl_3$, 298K) of **83**.

3-(4,4,5,5-Tetramethyl-1,3,2-dioxaborolan-2-yl)-5,6,7,8-tetrahydroquinoline **S16**

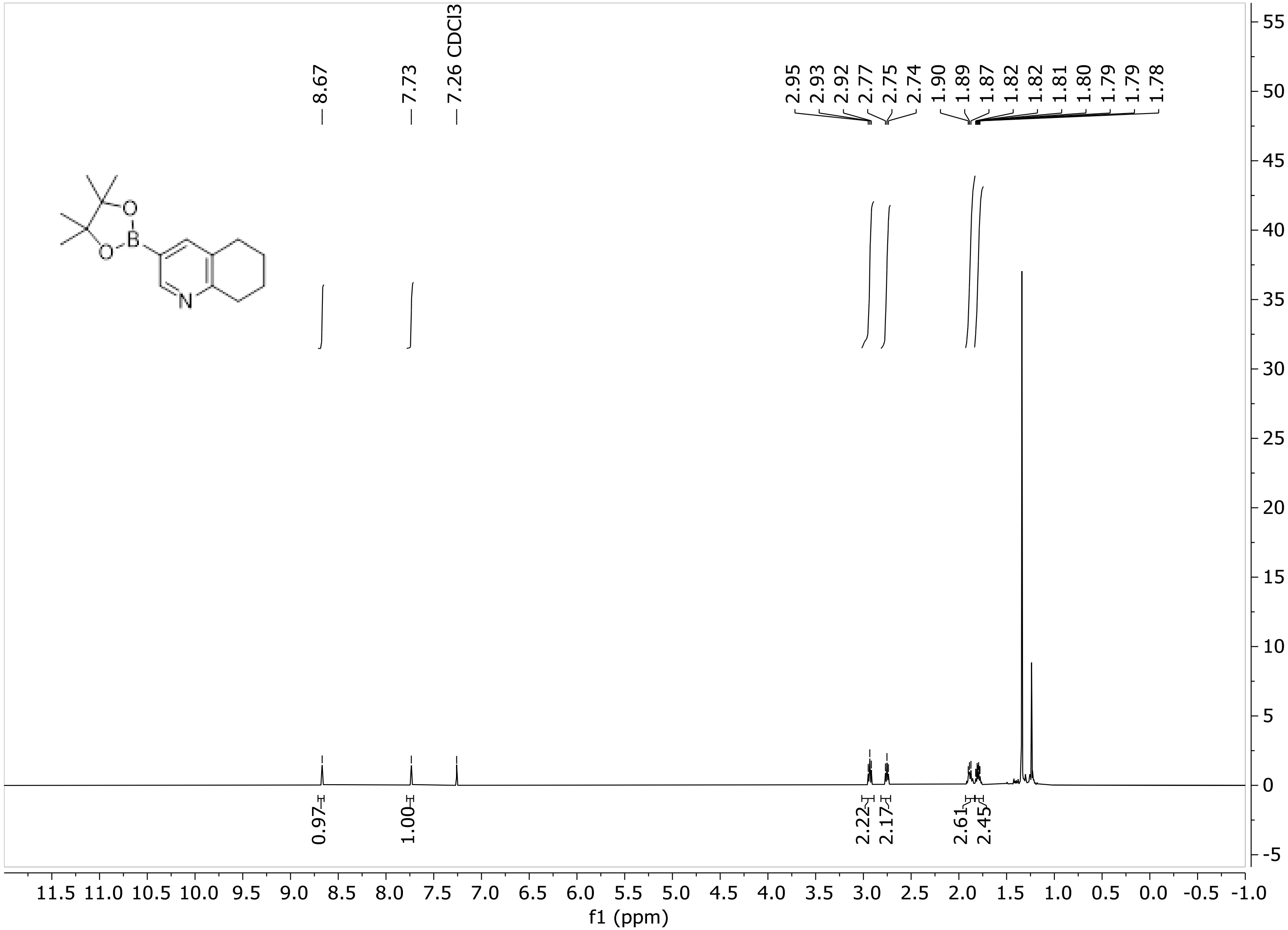

Figure S84. ^{1}H NMR (400 MHz, $CDCl_3$, 298K) of **S16**.

3-Bromo-5,6,7,8-tetrahydroquinoline **S17**

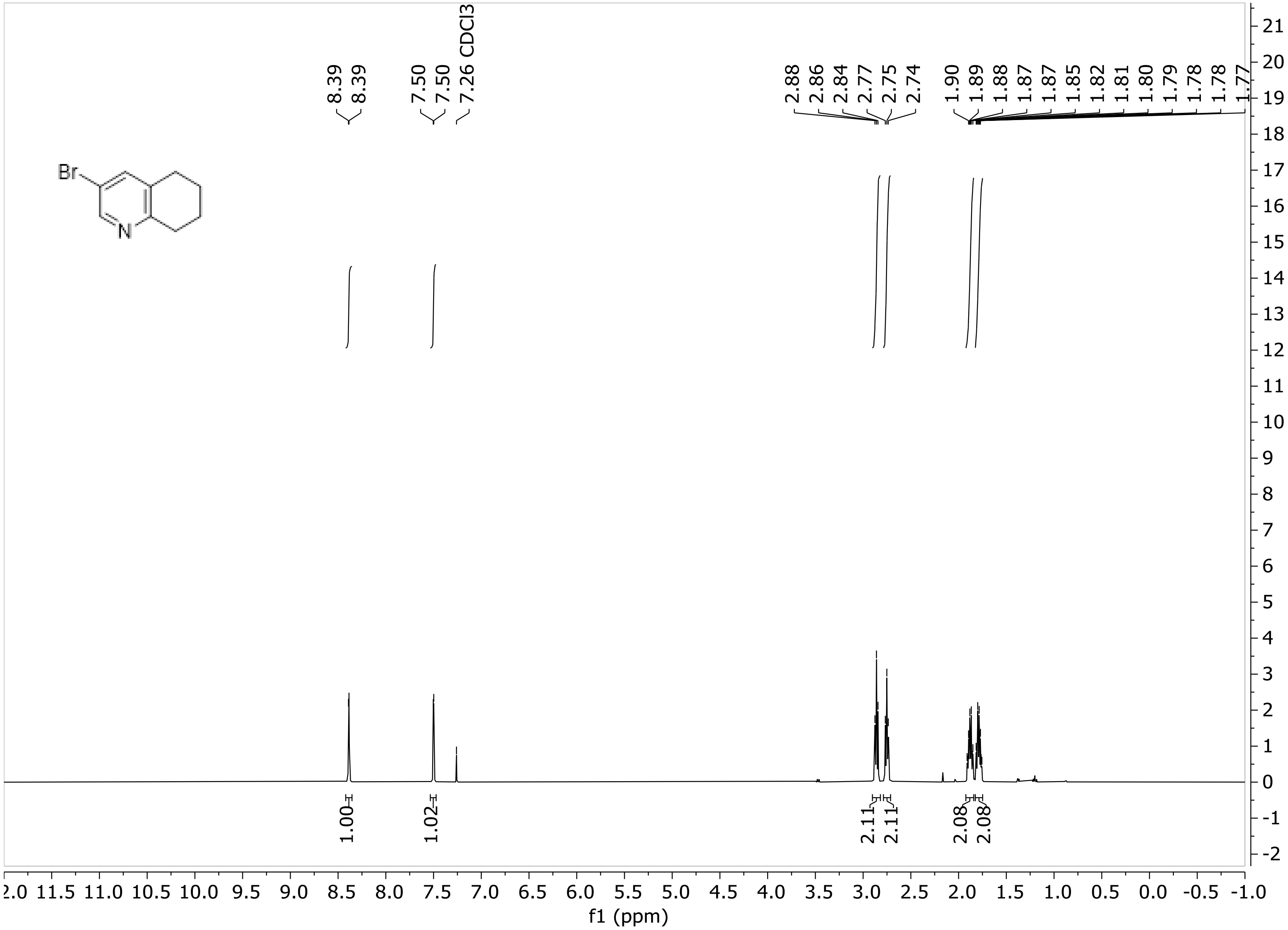

Figure S85. ^{1}H NMR (400 MHz, $CDCl_3$, 298K) of **S17**.

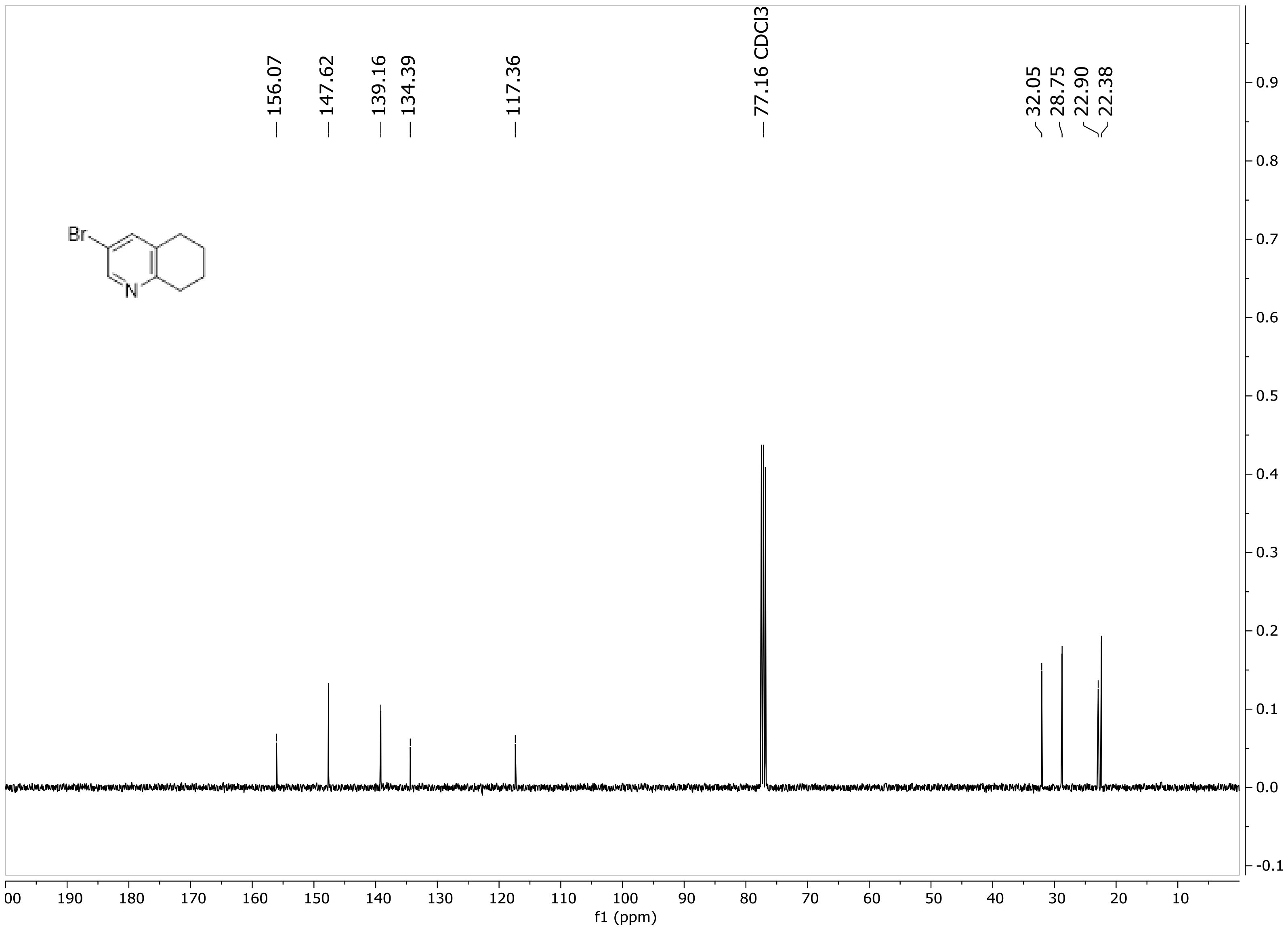

Figure S86. ^{13}C NMR (101 MHz, $CDCl_3$, 298K) of **S17**.

3-Methoxy-5,6,7,8-tetrahydroquinoline **87**

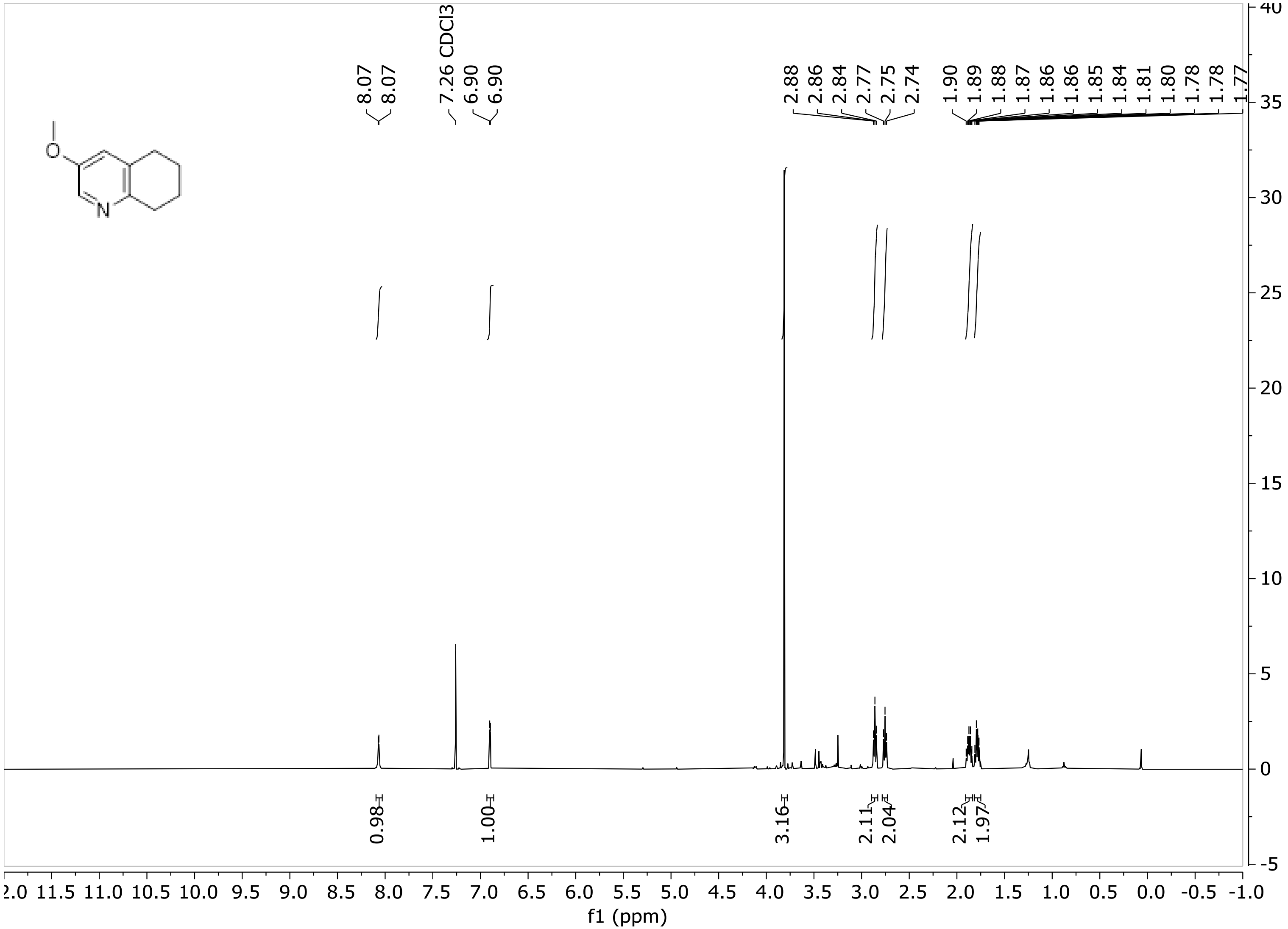

Figure S87. ^{1}H NMR (400 MHz, $CDCl_3$, 298K) of **87**.

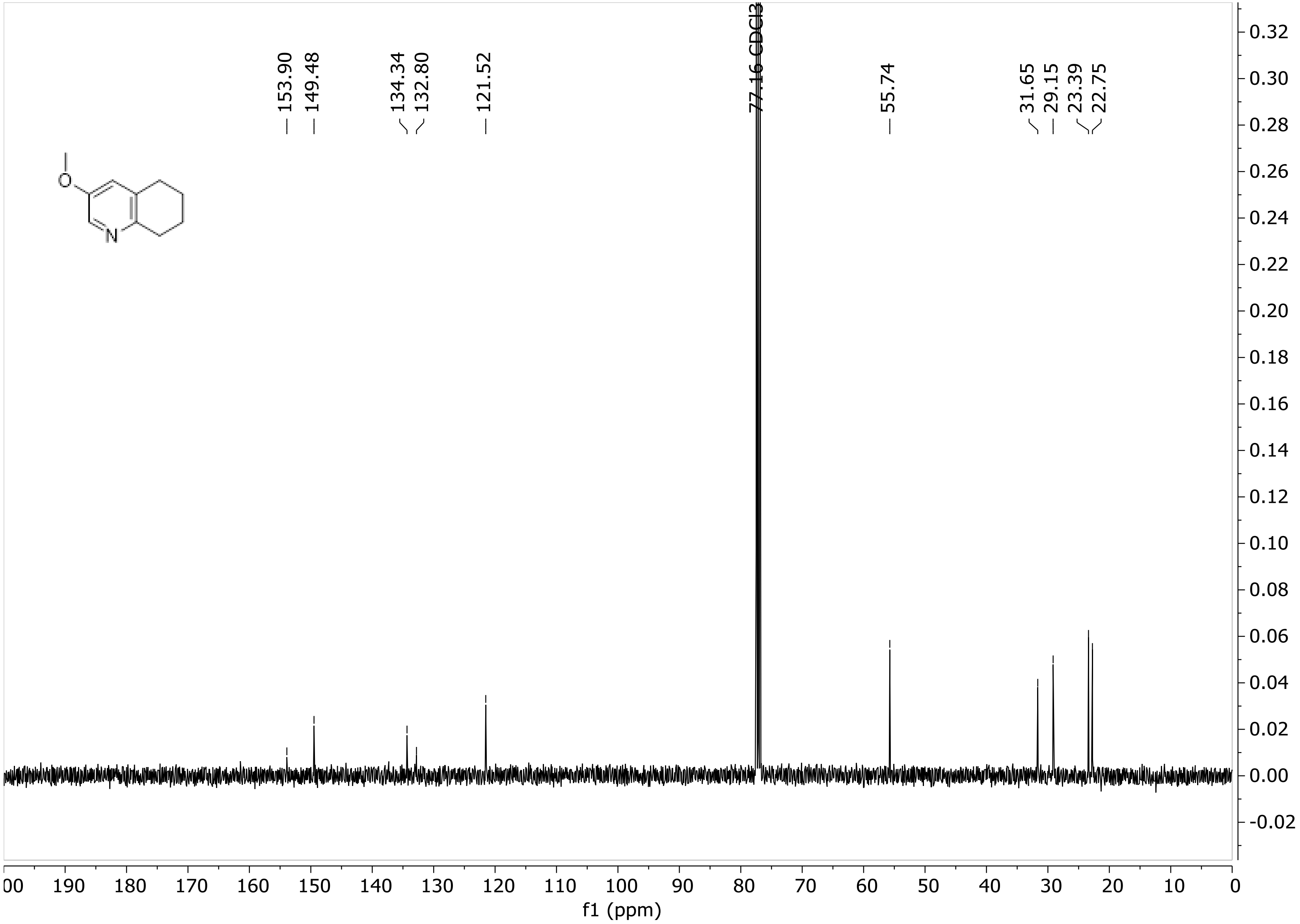

Figure S88. ^{1}H NMR (400 MHz, $CDCl_3$, 298K) of **87**.

3-(Methylsulfonyl)-5,6,7,8-tetrahydroquinoline **89**

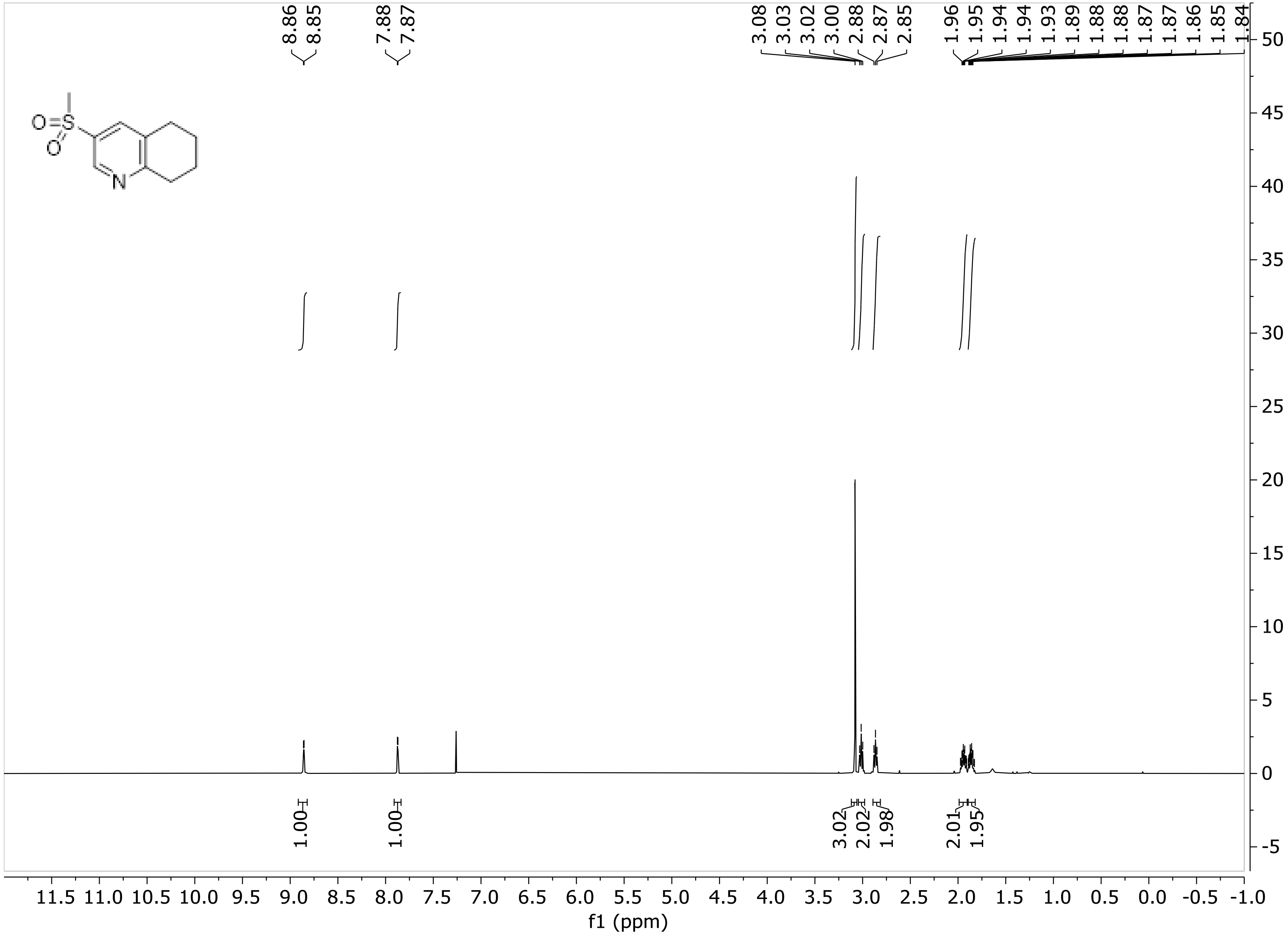

Figure S89. ^{1}H NMR (400 MHz, $CDCl_3$, 298K) of **89**.

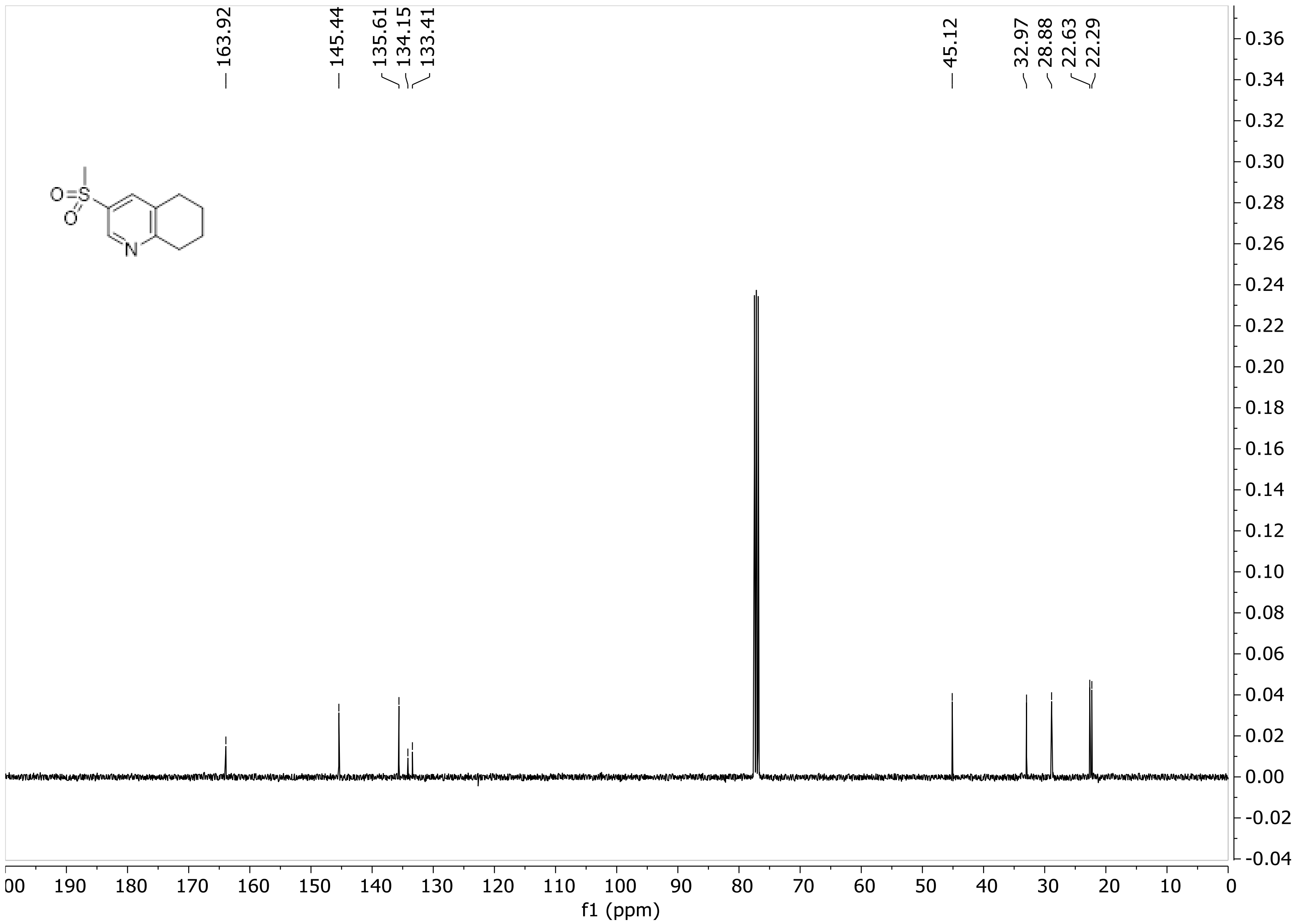

Figure S90. ^{13}C NMR (101 MHz, $CDCl_3$, 298K) of **89**.

Biocatalytically Synthesized Compounds

(-)-6,7-Dihydro-5H-cyclopenta[b]pyridin-5-ol (*R*)-**2**

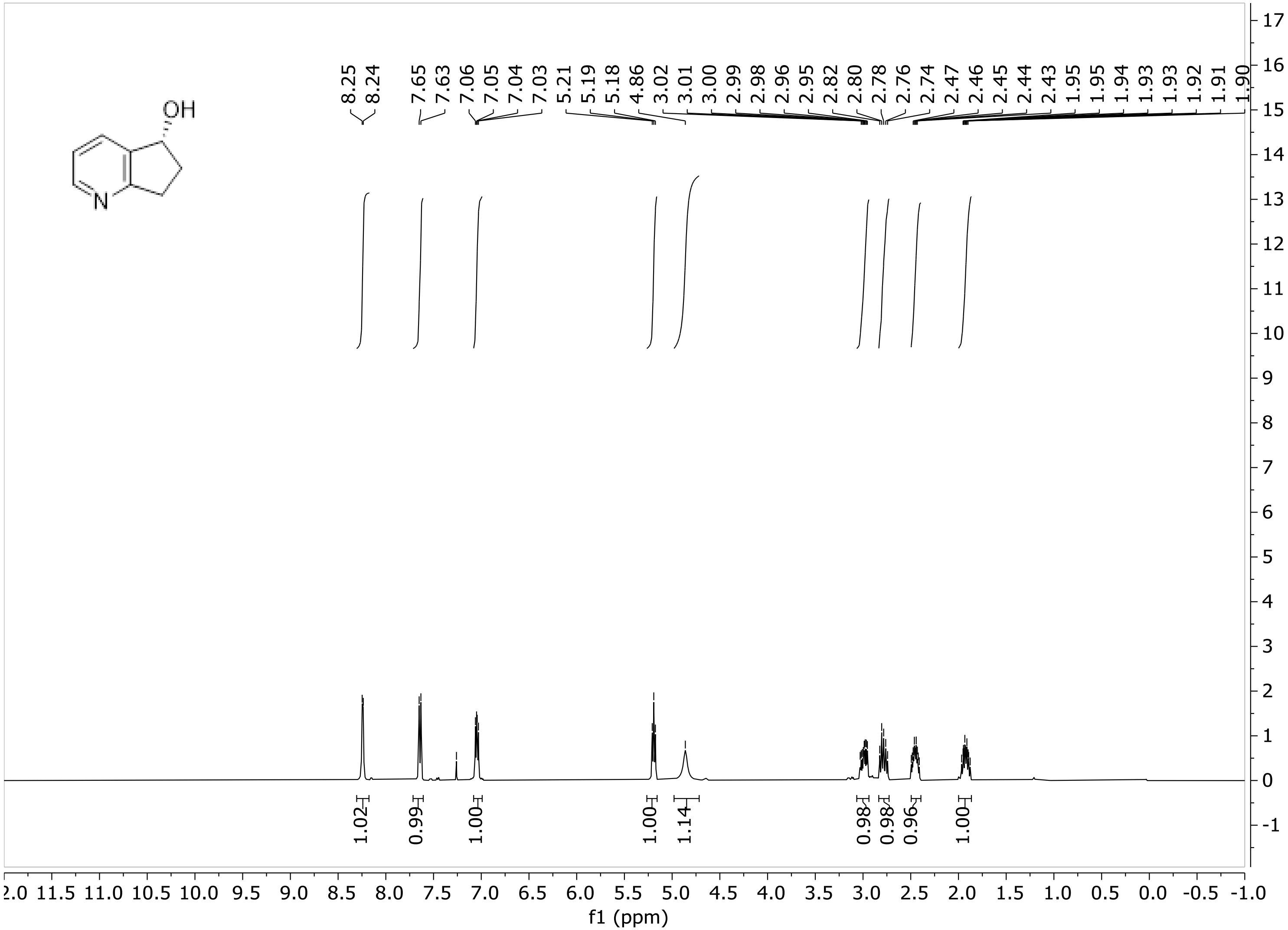

Figure S91. ^{1}H NMR (400 MHz, $CDCl_3$, 298K) of (*R*)-**2**.

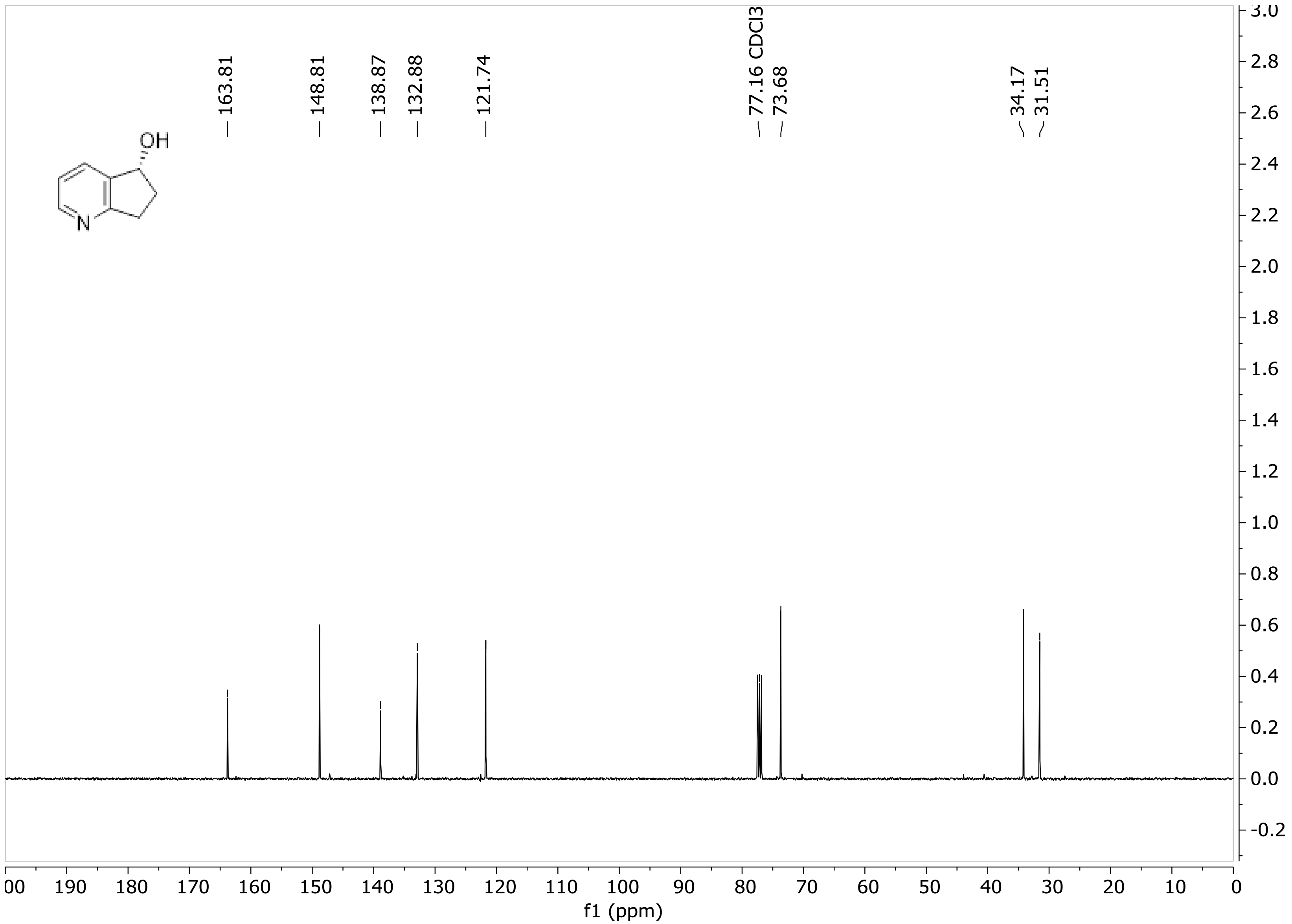

Figure S92. ^{13}C NMR (101 MHz, $CDCl_3$, 298K) of (*R*)-**2**.

6,7-Dihydro-5H-cyclopenta[b]pyridin-5-one **54**

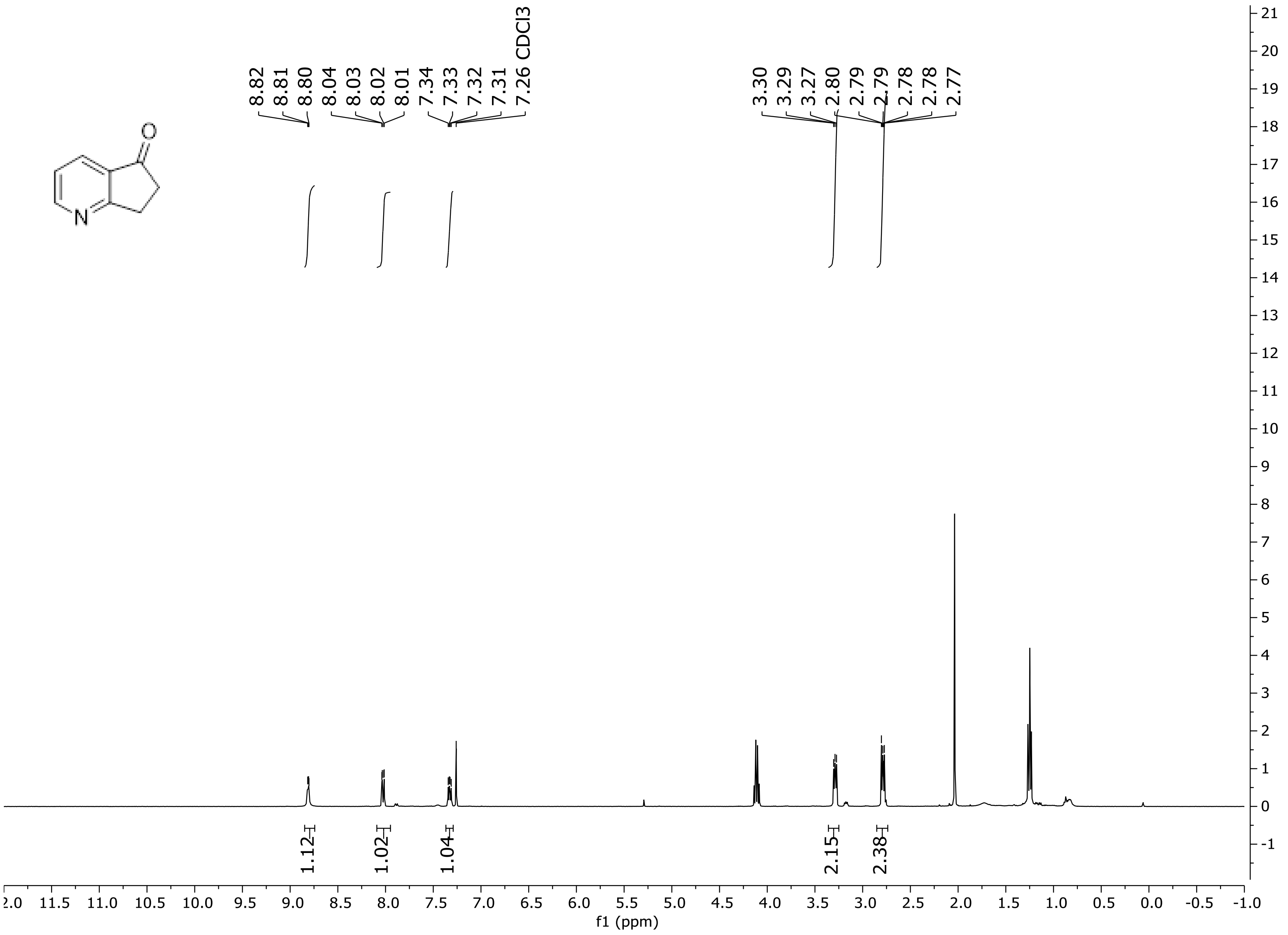

Figure S93. ^{1}H NMR (400 MHz, $CDCl_3$, 298K) of **54**.

(+)-6,7-Dihydro-5H-cyclopenta[b]pyridine-7-ol (R)-**55**

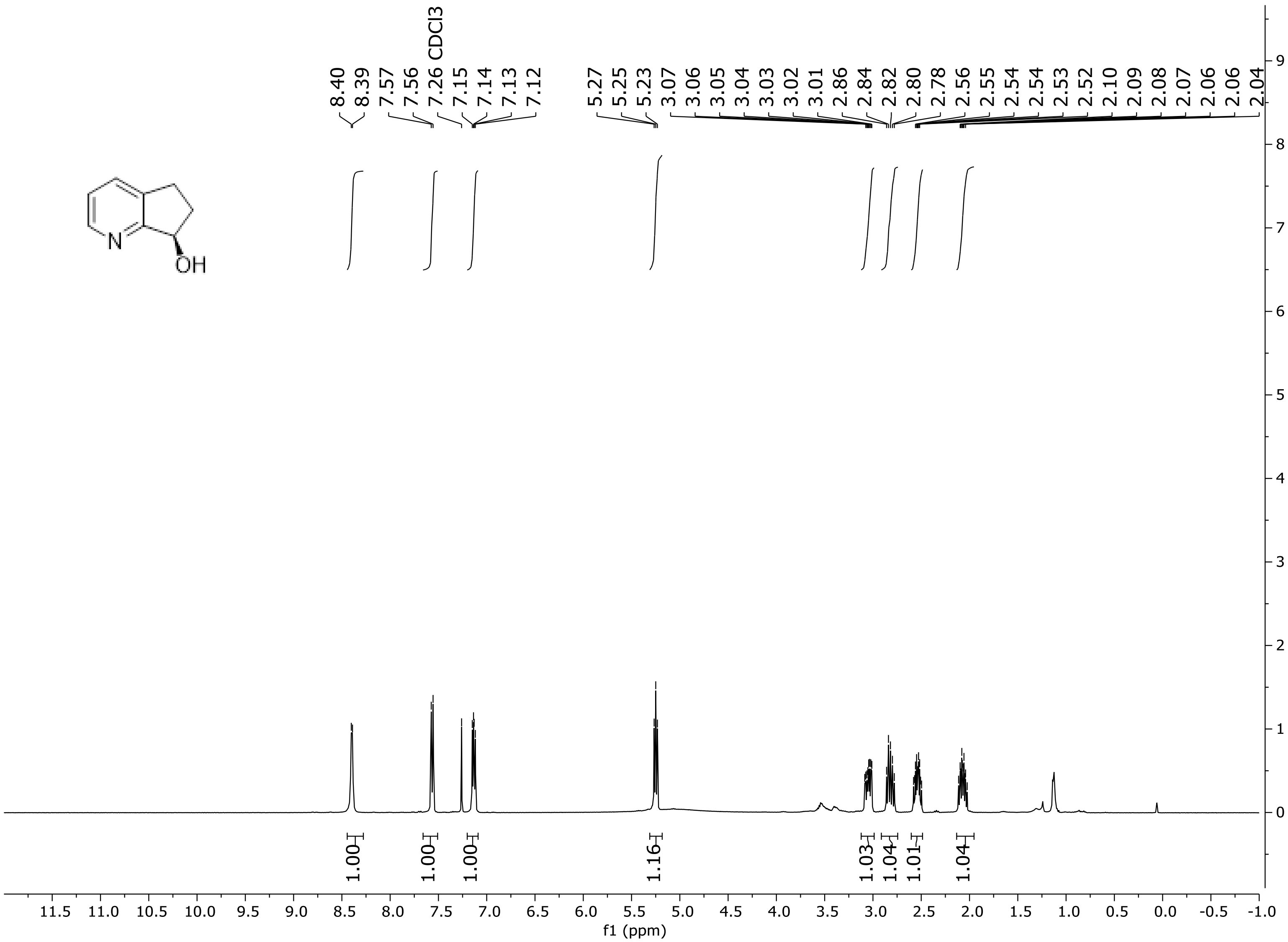

Figure S94. ^{1}H NMR (400 MHz, $CDCl_3$, 298K) of *(R)*-**55**.

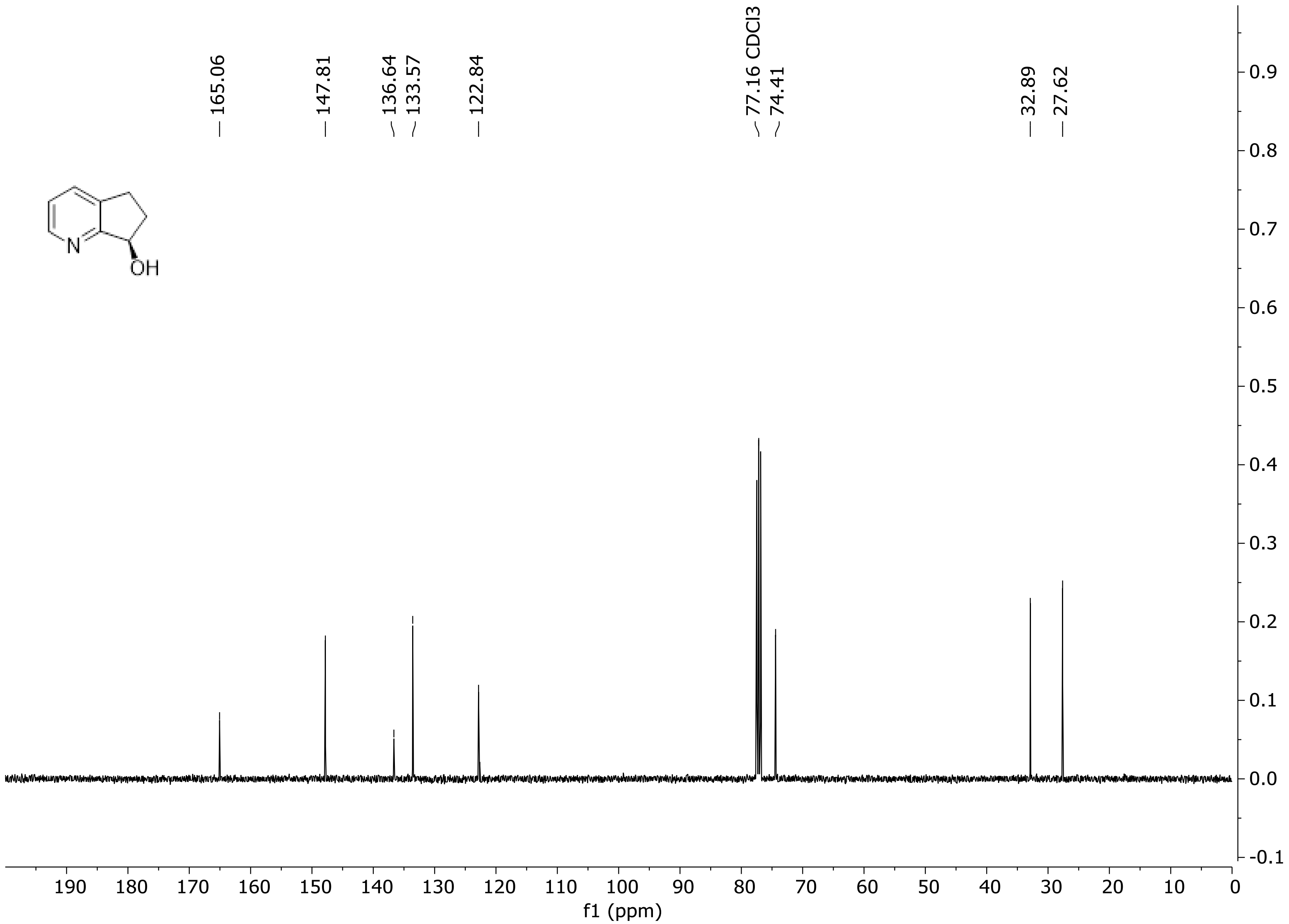

Figure S95. ^{13}C NMR (101 MHz, $CDCl_3$, 298K) of *(R)*-**55**.

(-)-5,6,7,8-Tetrahydroquinolin-5-ol (*R*)-**4**

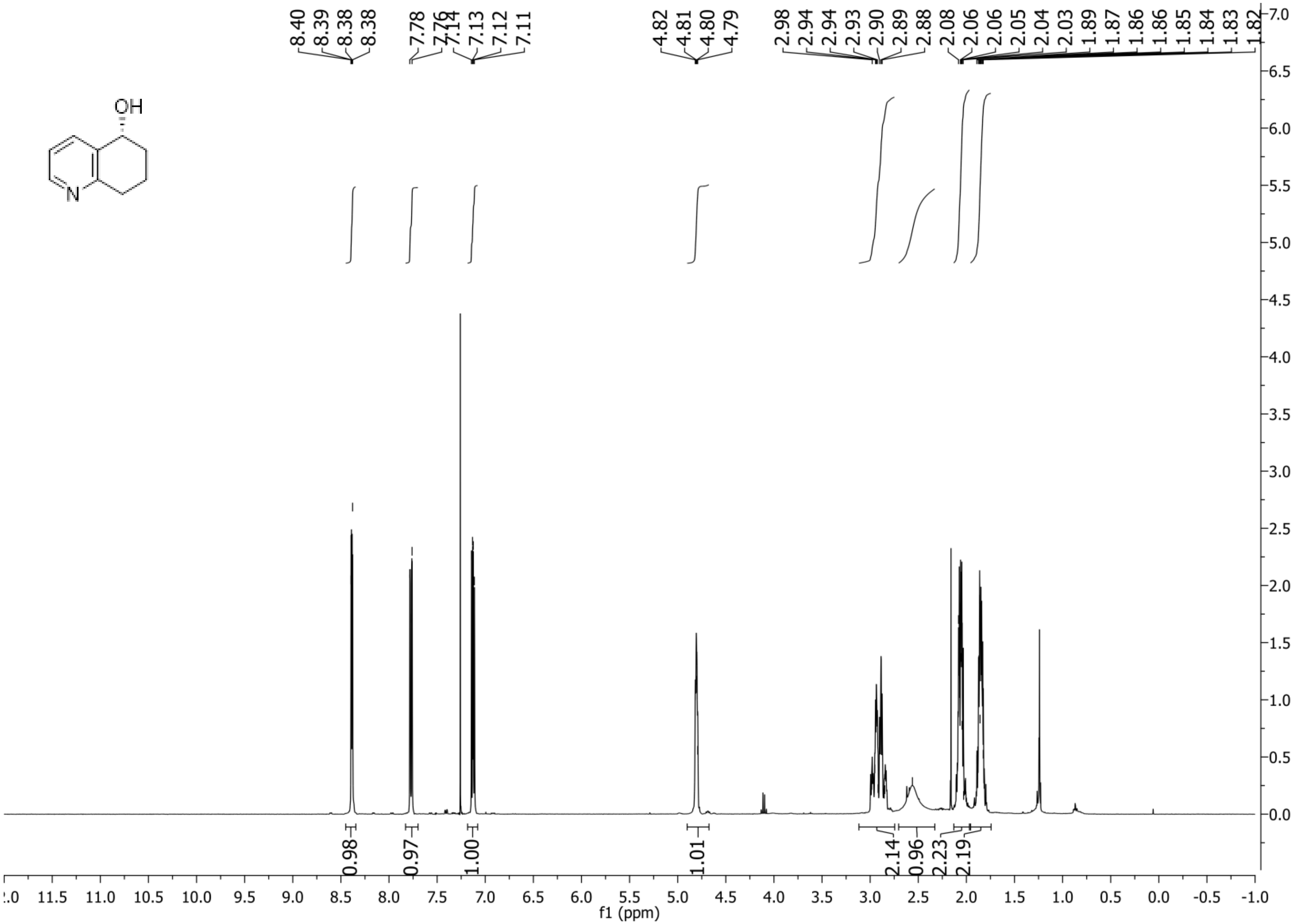

Figure S96. ^{1}H NMR (400 MHz, $CDCl_3$, 298K) of (*R*)-**4**.

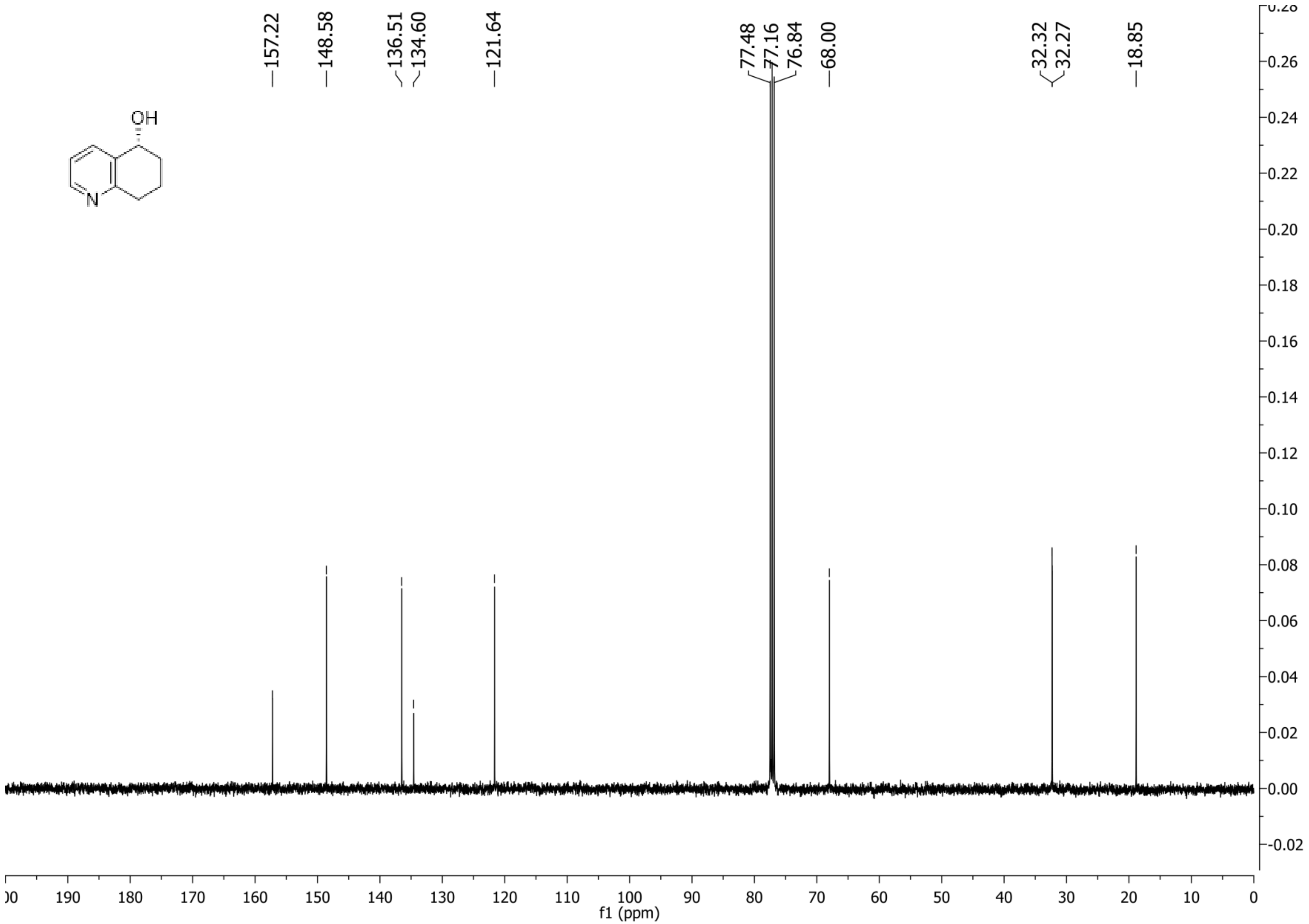

Figure S97. ^{13}C NMR (101 MHz, $CDCl_3$, 298K) of (*R*)-**4**.

3,4-dihydroisoquinolin-1(2H)-one **38**

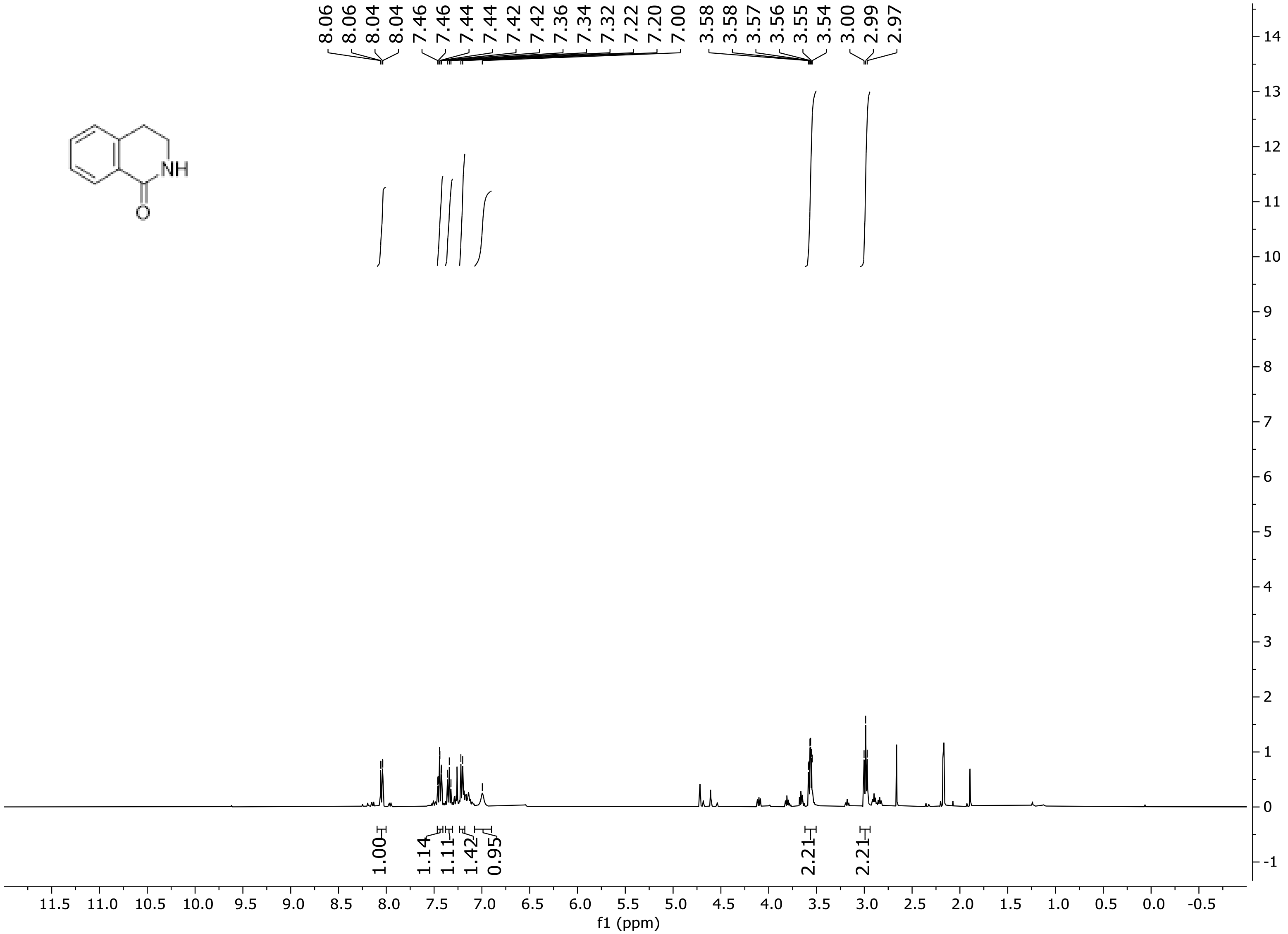

Figure S98. ^{1}H NMR (400 MHz, $CDCl_3$, 298K) of **38**.

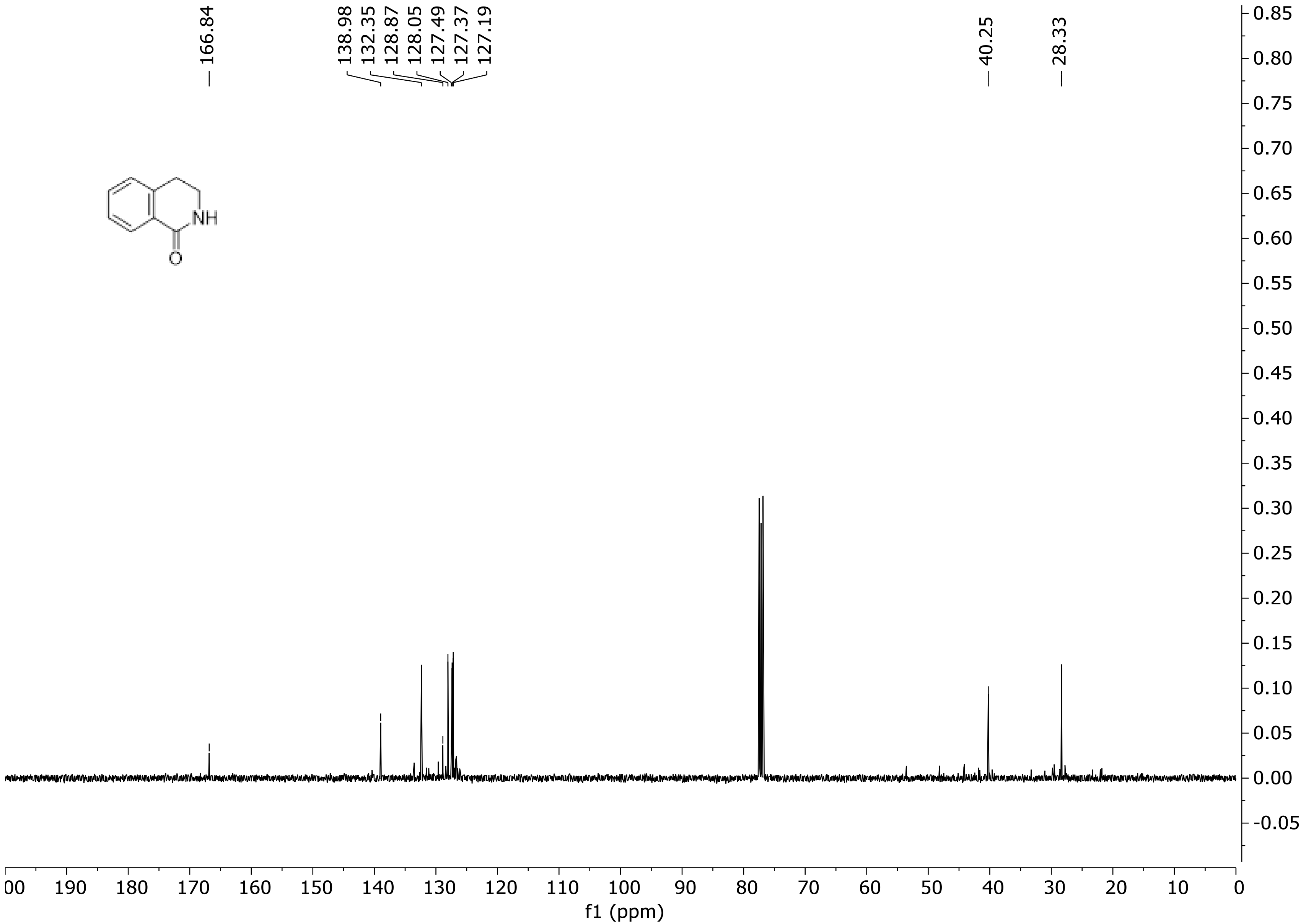

Figure S99. ^{13}C NMR (101 MHz, $CDCl_3$, 298K) of **38**.

*(E)-3,3',4,4'-tetrahydro-2*H*,2'*H*-1,1'-biisoquinolinylidene* **39**

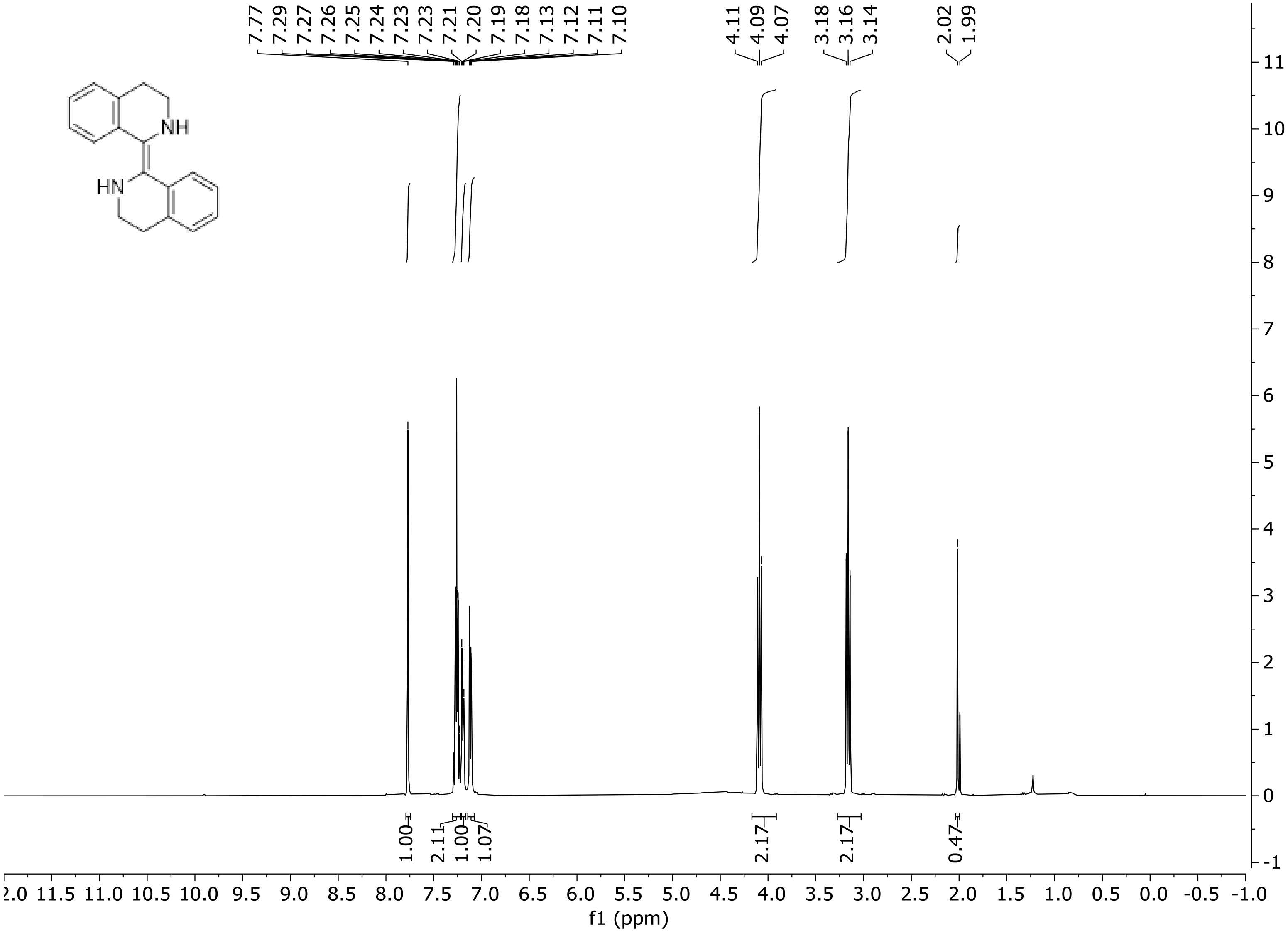

Figure S100. ^{1}H NMR (400 MHz, $CDCl_3$, 298K) of **39**.

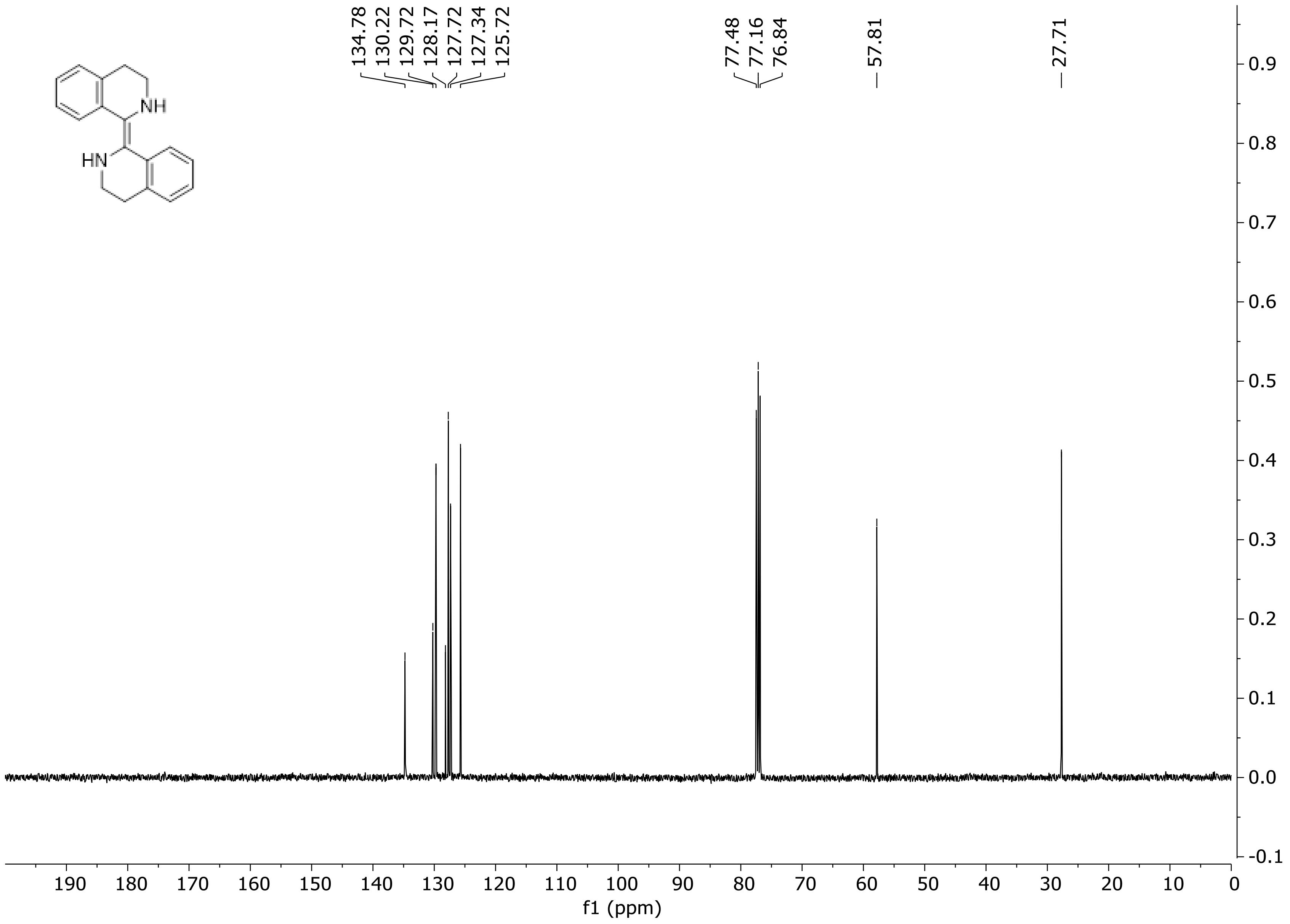

Figure S101. ^{13}C NMR (101 MHz, $CDCl_3$, 298K) of **39**.

Indolin-2-one **51**

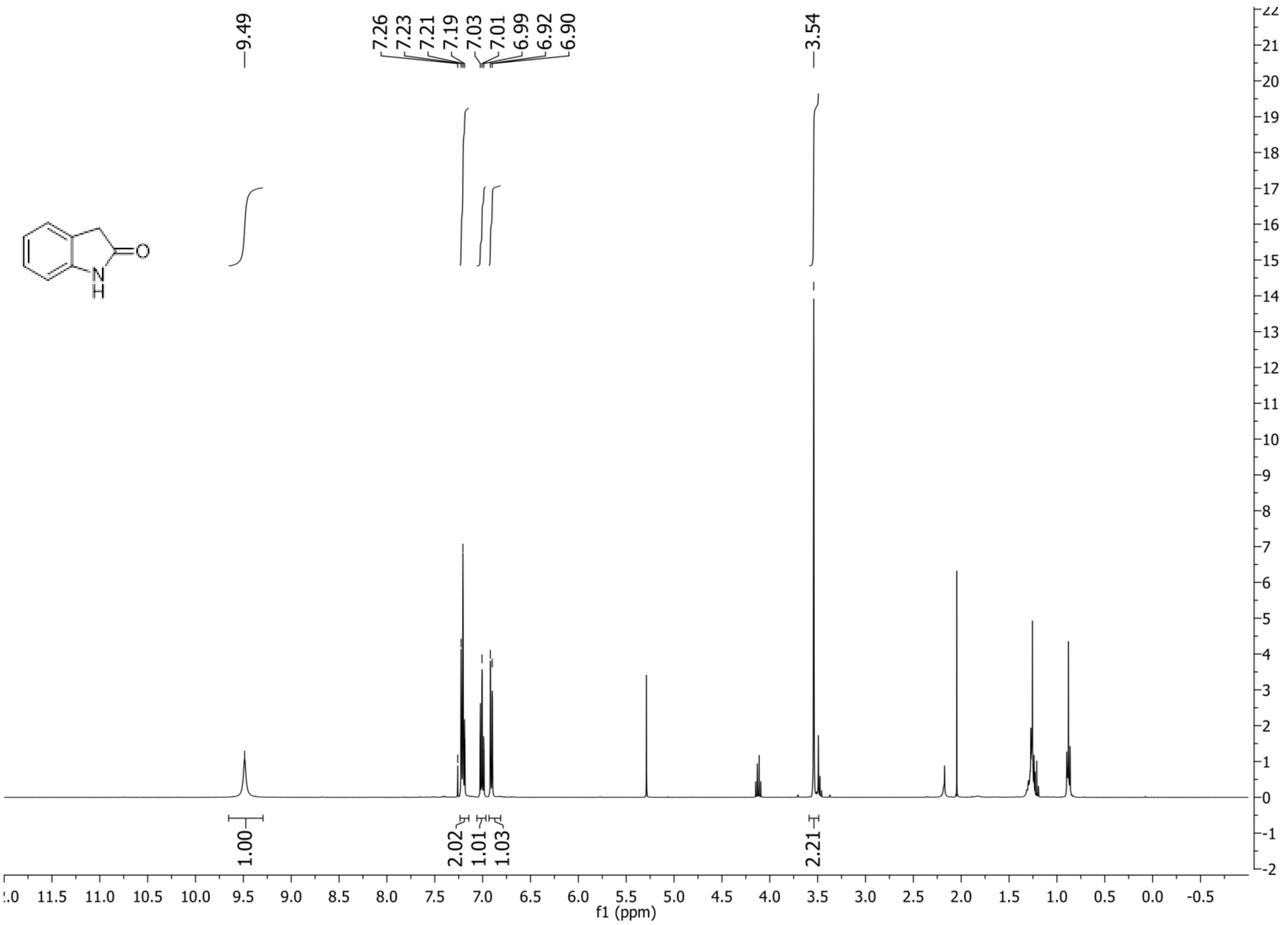

Figure S102. ^{1}H NMR (400 MHz, $CDCl_3$, 298K) of **51**.

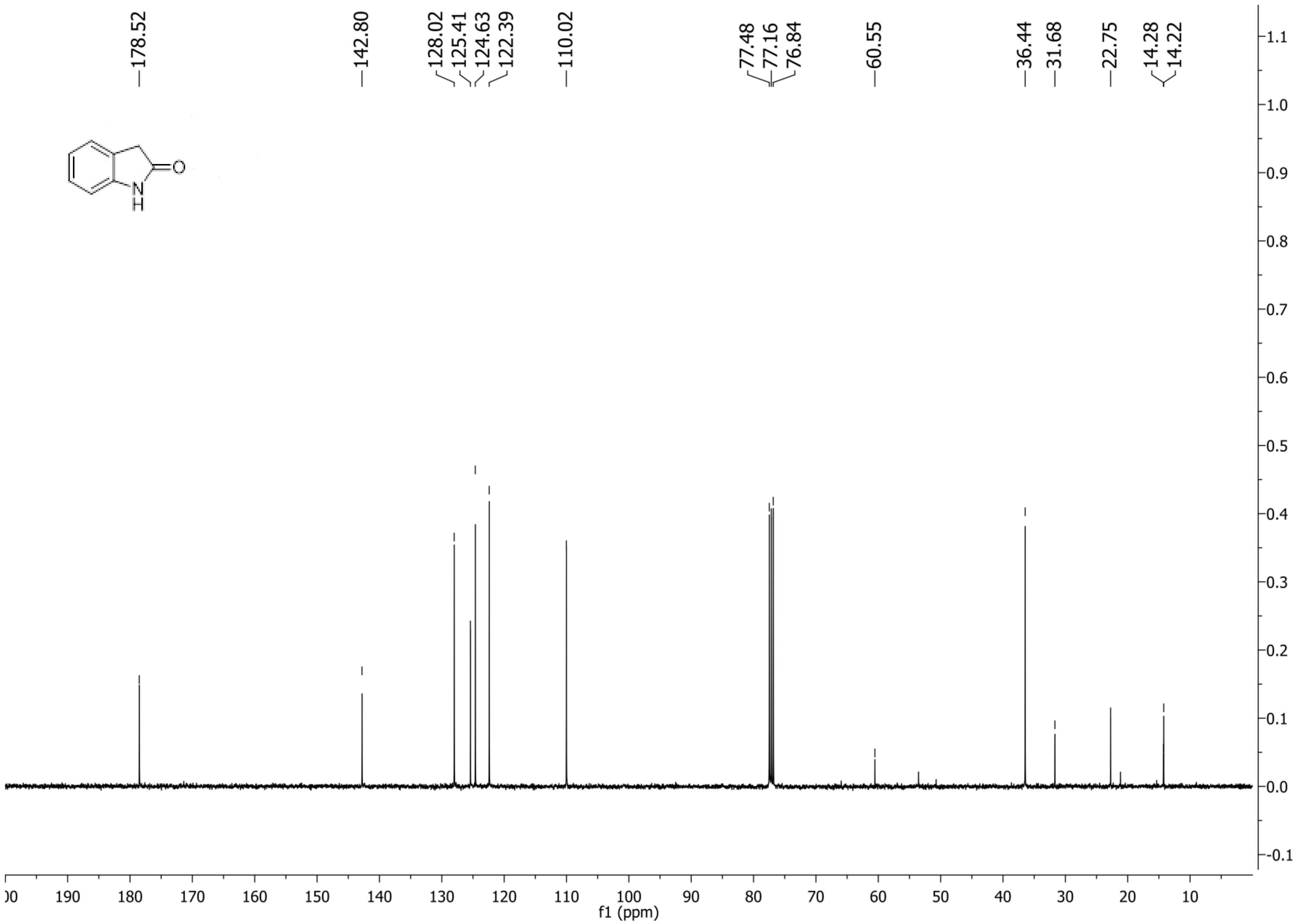

Figure S103. ^{13}C NMR (101 MHz, $CDCl_3$, 298K) of **51**.

2-Methylindolin-1-one **52**

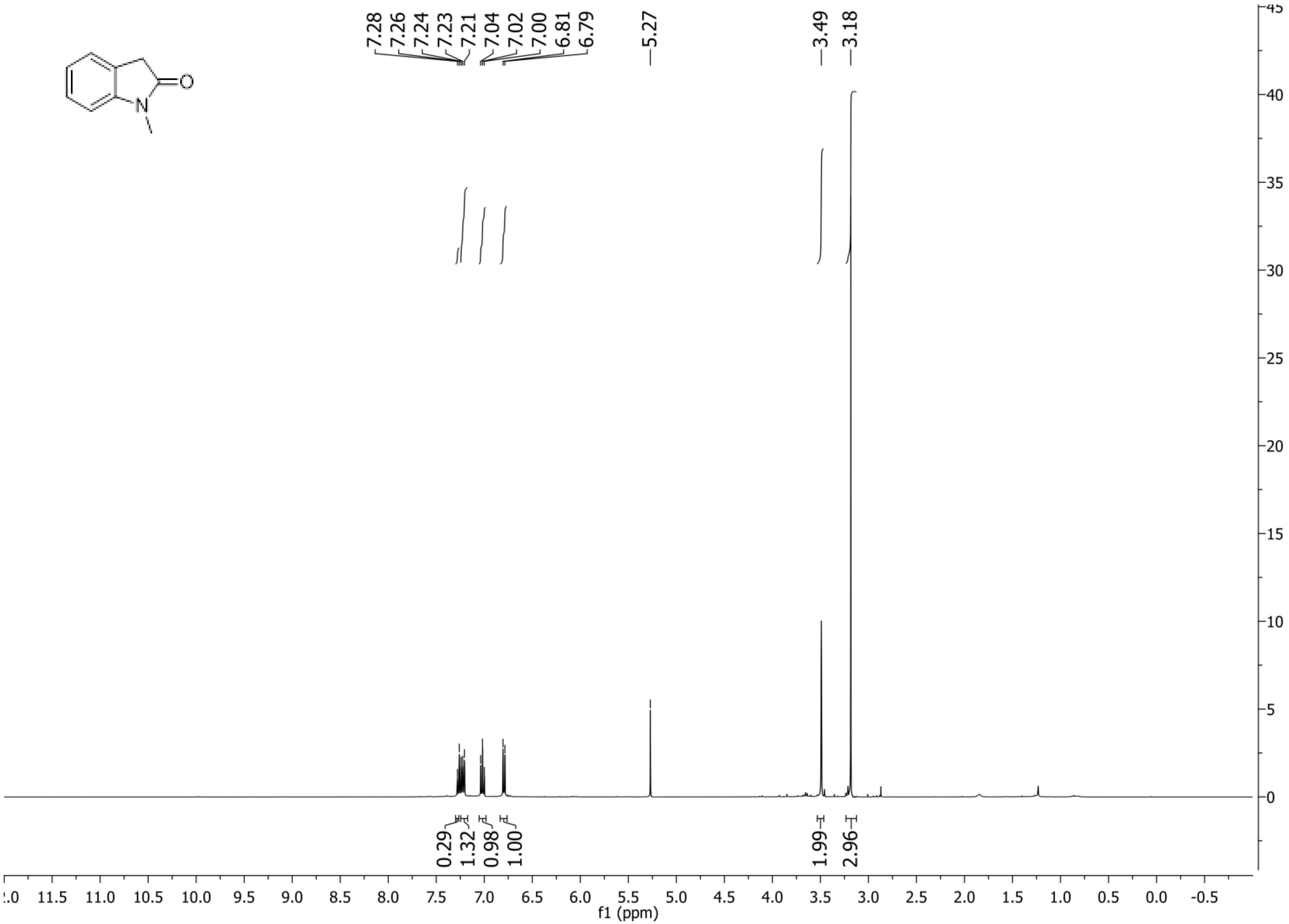

Figure S104. ^{1}H NMR (400 MHz, $CDCl_3$, 298K) of **52**.

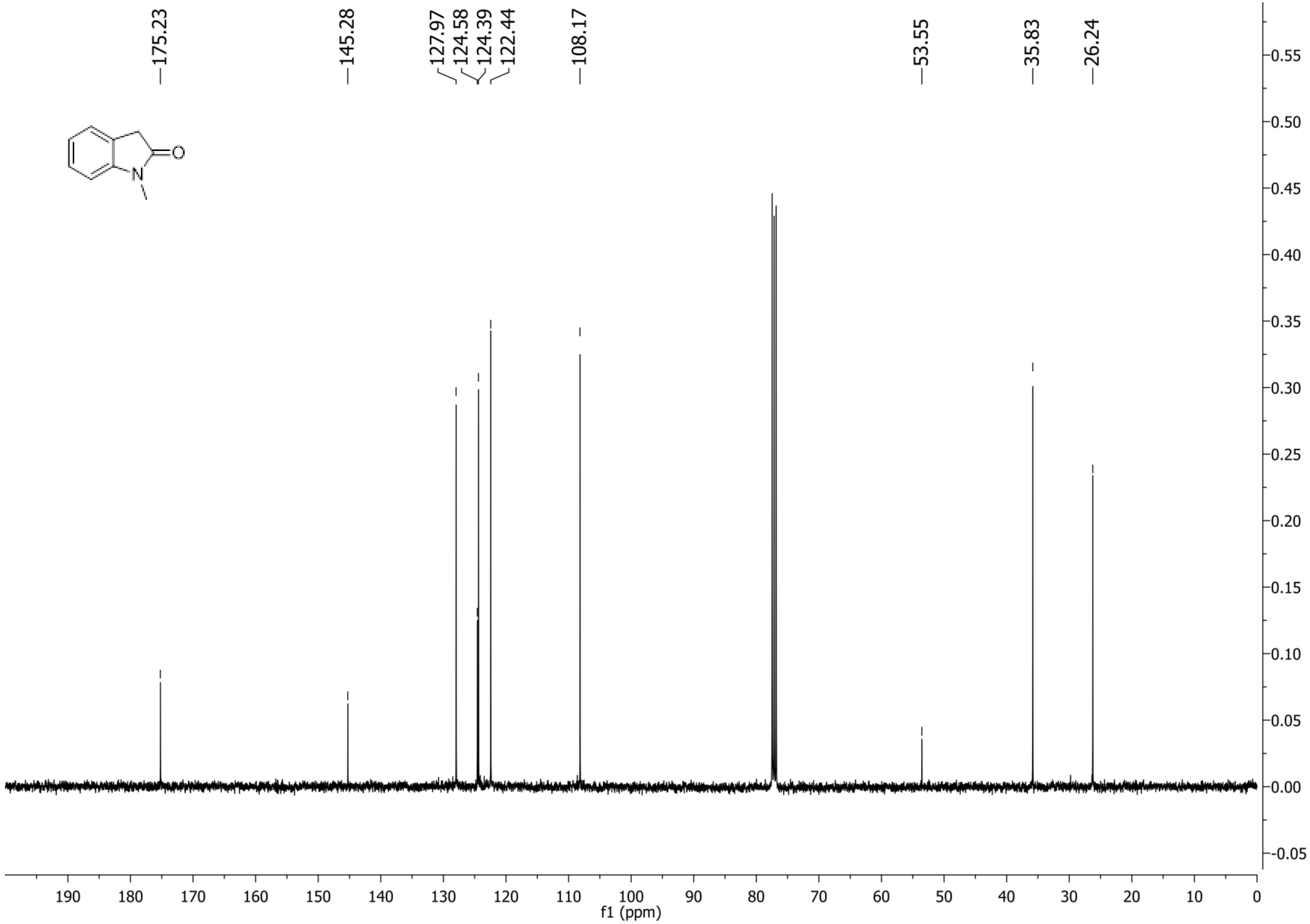

Figure S105. ^{13}C NMR (101 MHz, $CDCl_3$, 298K) of **52**.

3-Methylindolin-2-one **53**

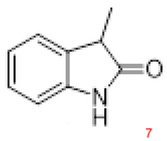

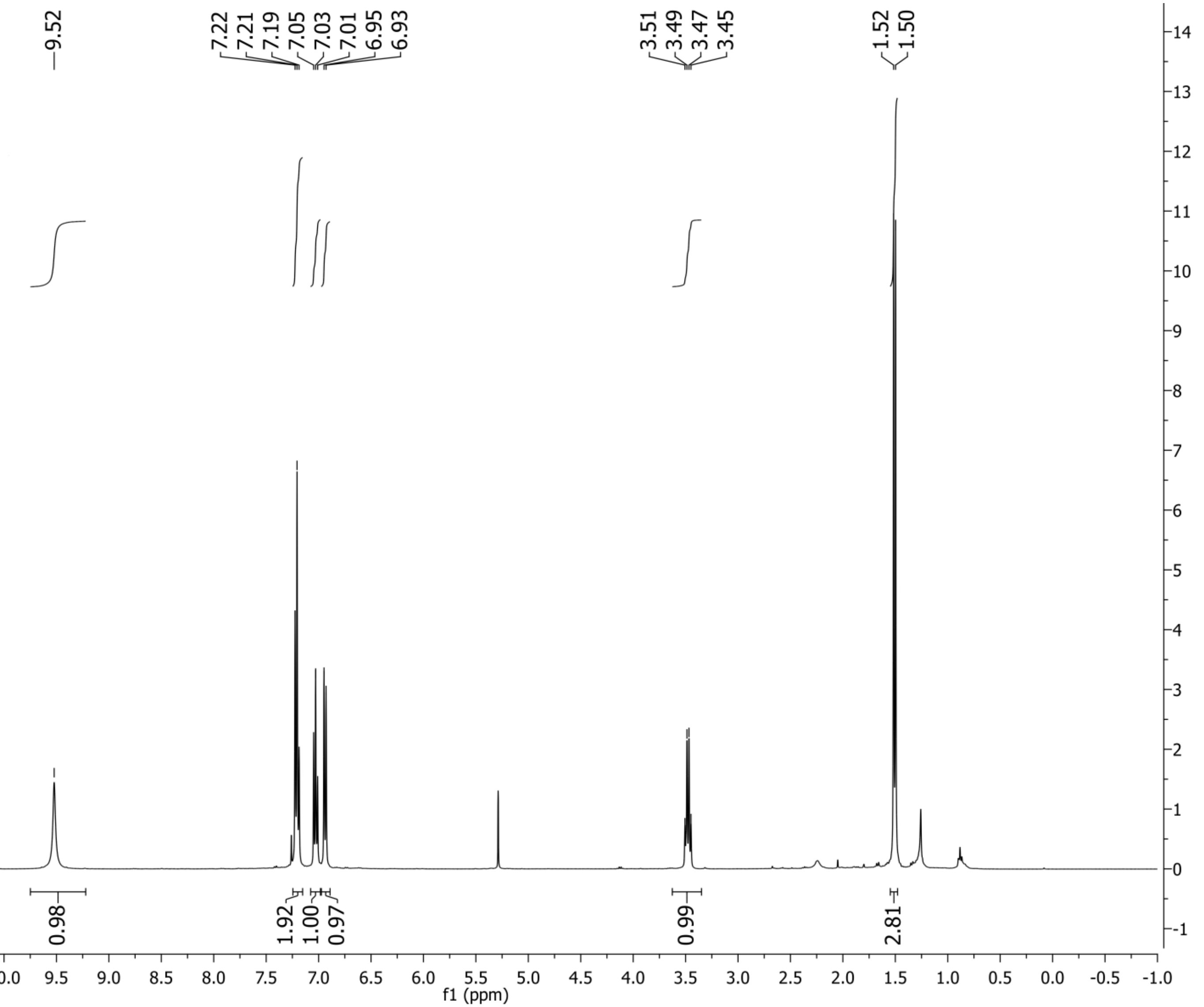

Figure S106. ^{1}H NMR (400 MHz, $CDCl_3$, 298K) of **53**.

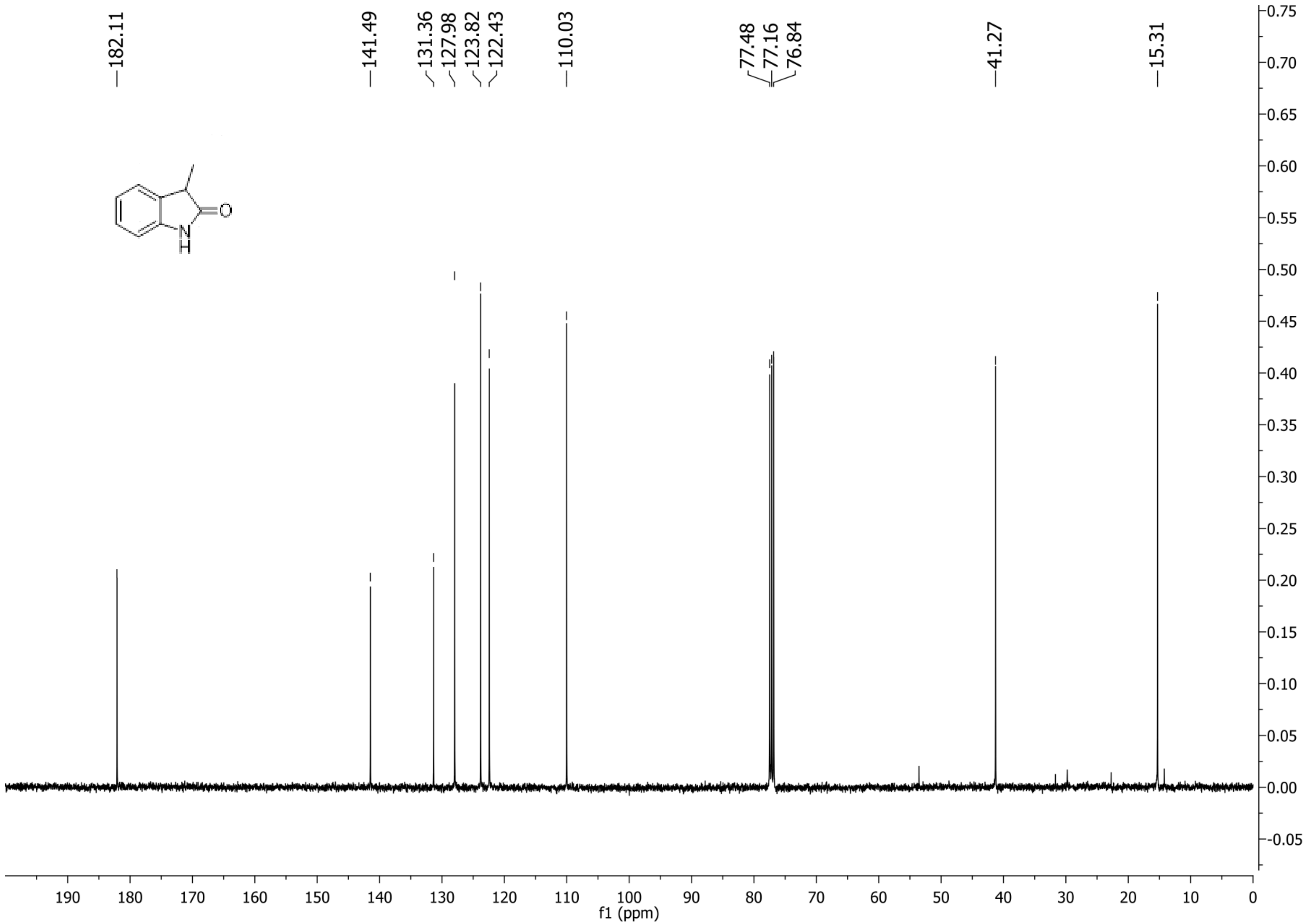

Figure S107. ^{13}C NMR (101 MHz, $CDCl_3$, 298K) of **53**.

(R)-1-(Pyridyl-3-yl)ethanol (*R*)-**20**

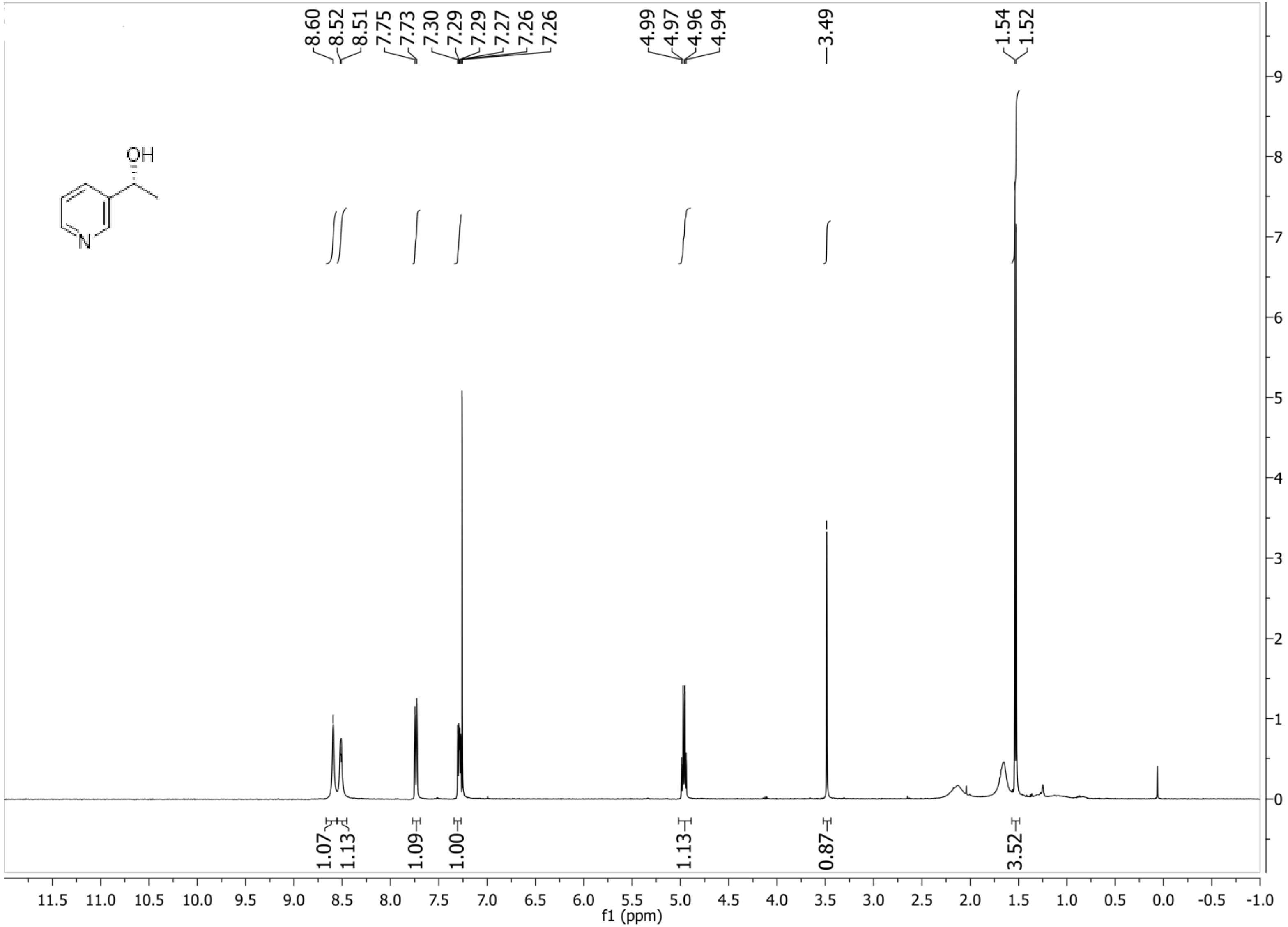

Figure S108. ^{1}H NMR (400 MHz, $CDCl_3$, 298K) of (*R*)-**20**.

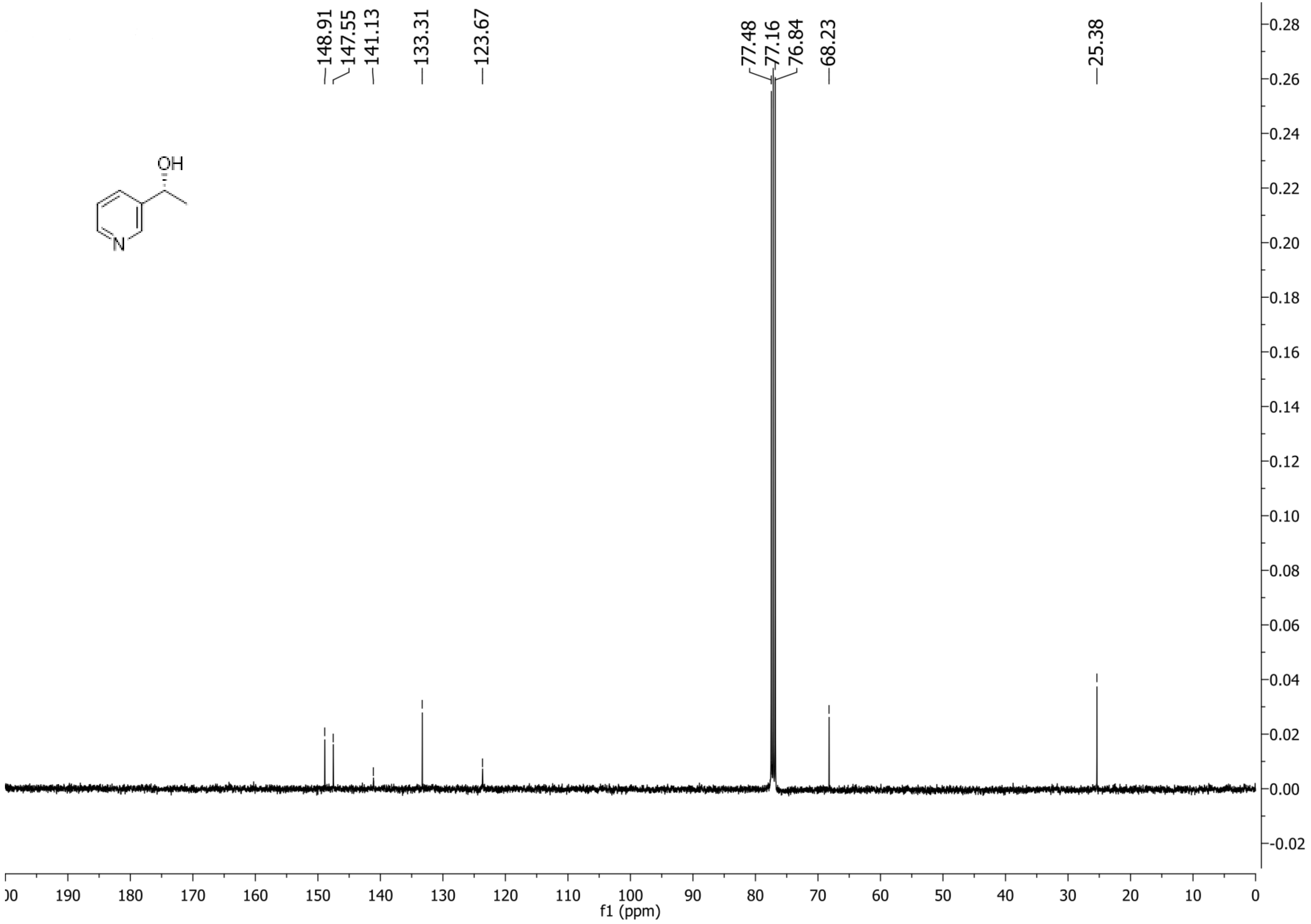

Figure S109. ^{13}C NMR (101 MHz, $CDCl_3$, 298K) of (*R*)-**20**.

3-Acetylpyridine **21**

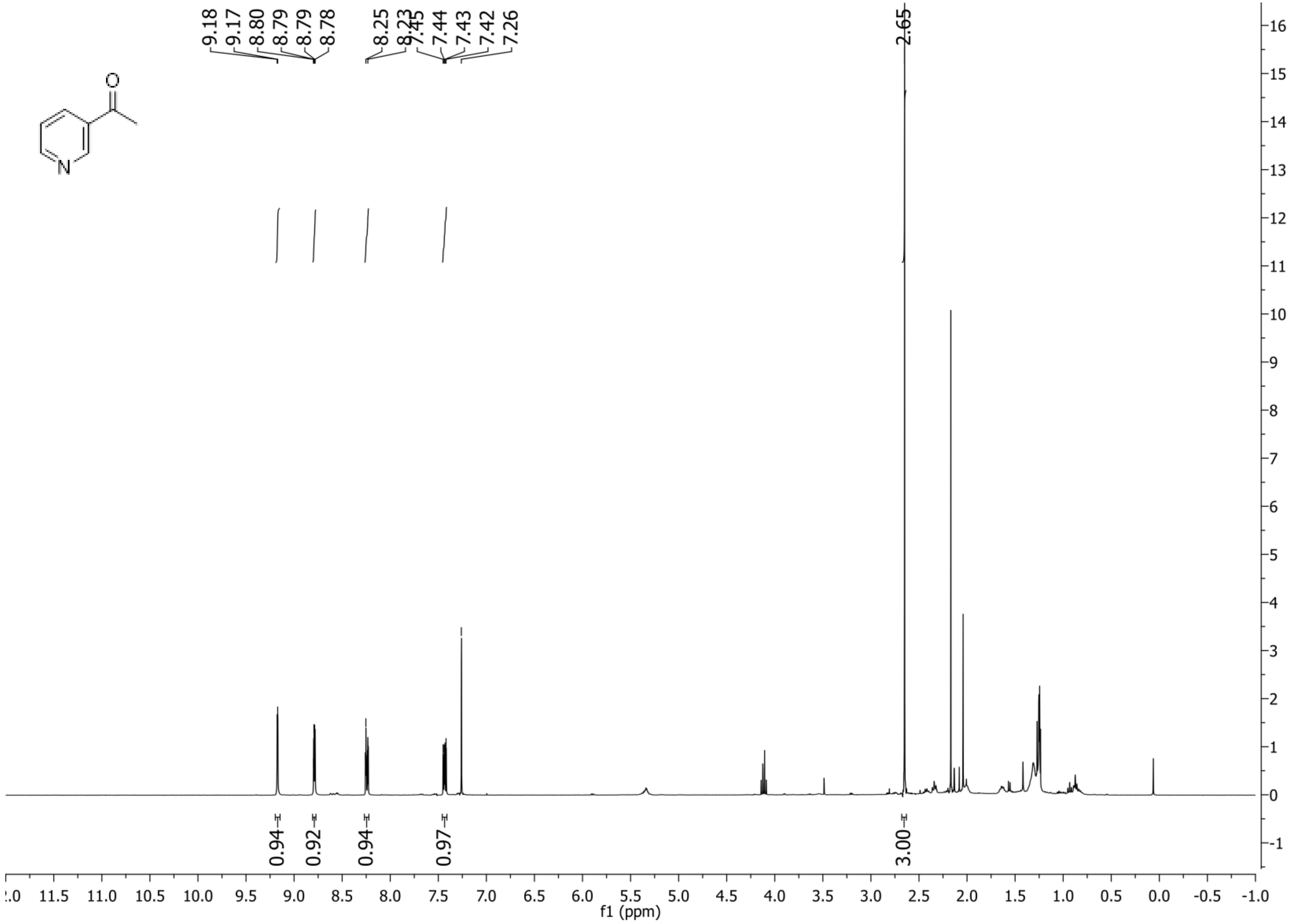

Figure S110. ^{1}H NMR (400 MHz, $CDCl_3$, 298K) of **21**.

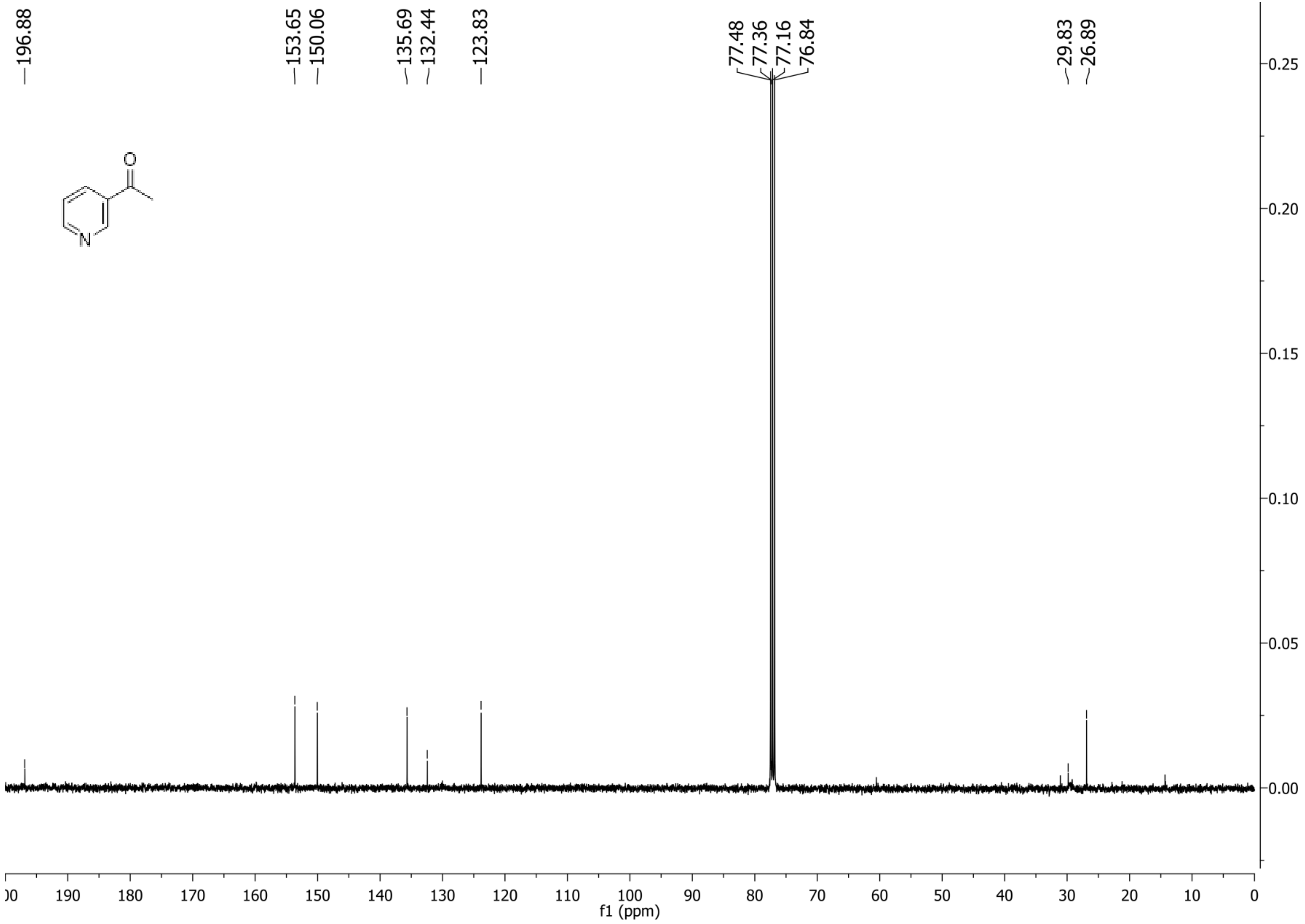

Figure S111. ^{13}C NMR (101 MHz, $CDCl_3$, 298K) of **21**.

(R)-1-(Pyridyl-2-yl)-ethan-1-ol (*R*)-**22**

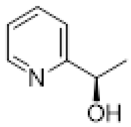

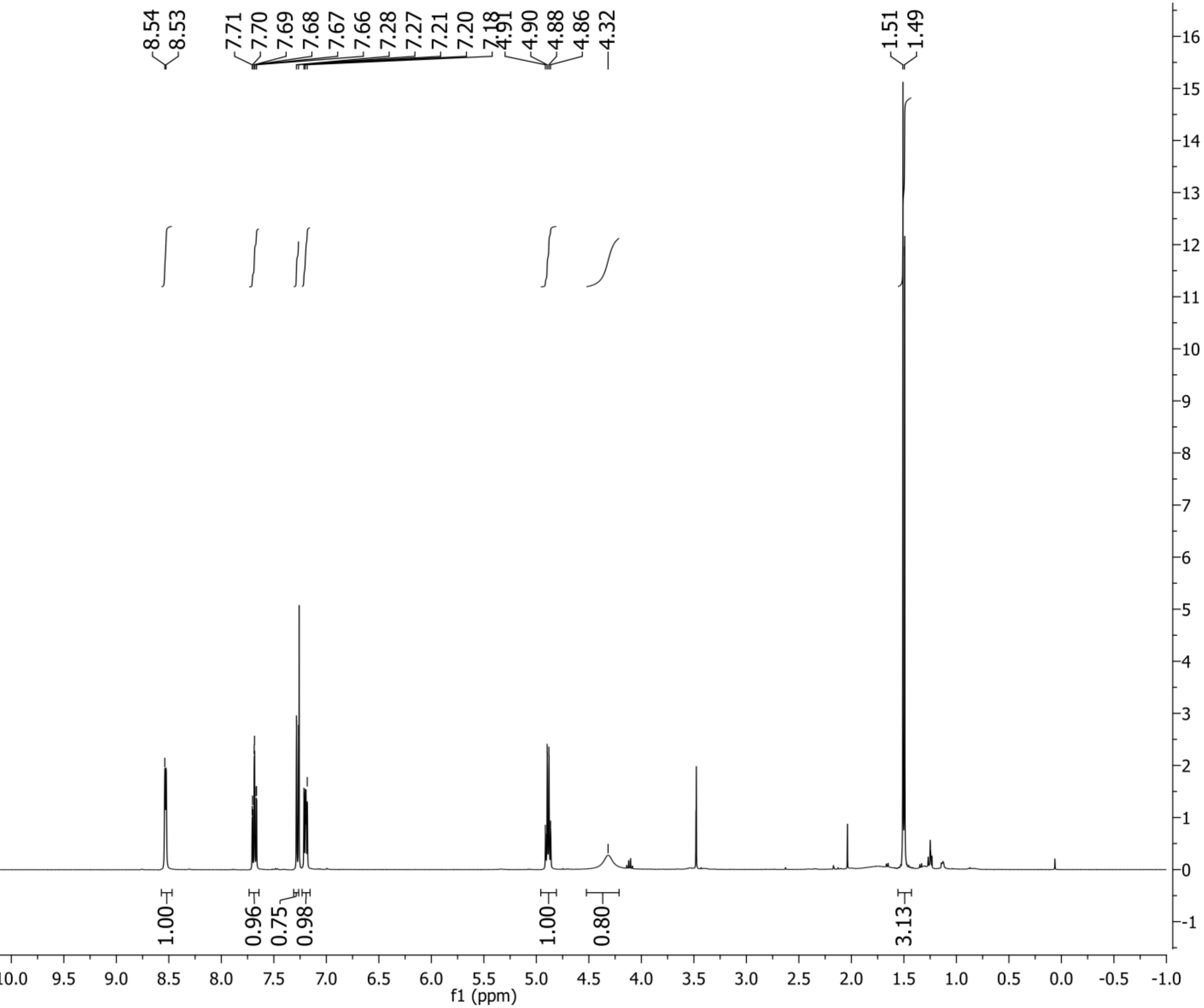

Figure S112. ^{1}H NMR (400 MHz, $CDCl_3$, 298K) of (*R*)-**22**.

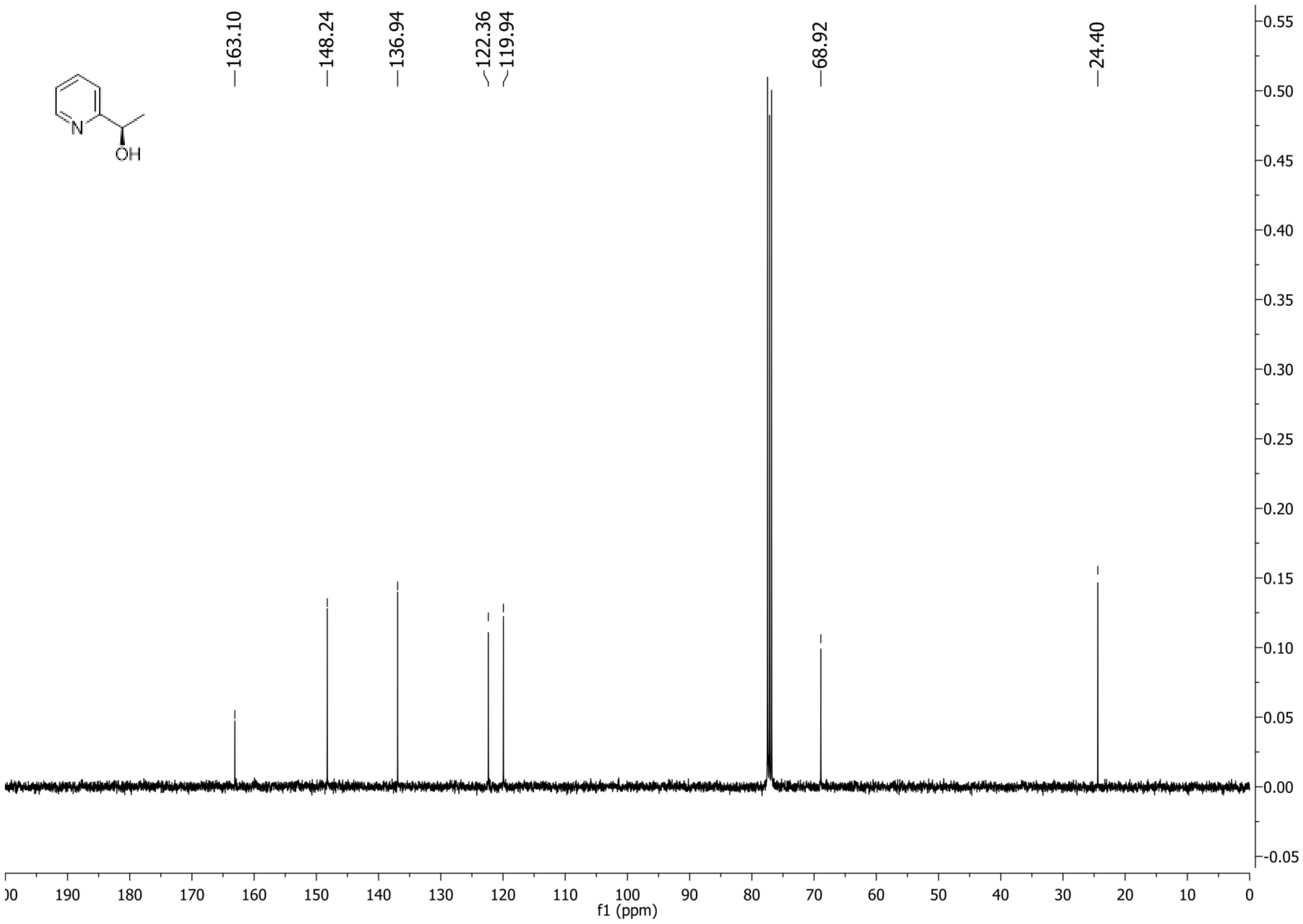

Figure S113. ^{13}C NMR (101 MHz, $CDCl_3$, 298K) of (*R*)-**22**.

2-Ethylpyridine-N-oxide **57**

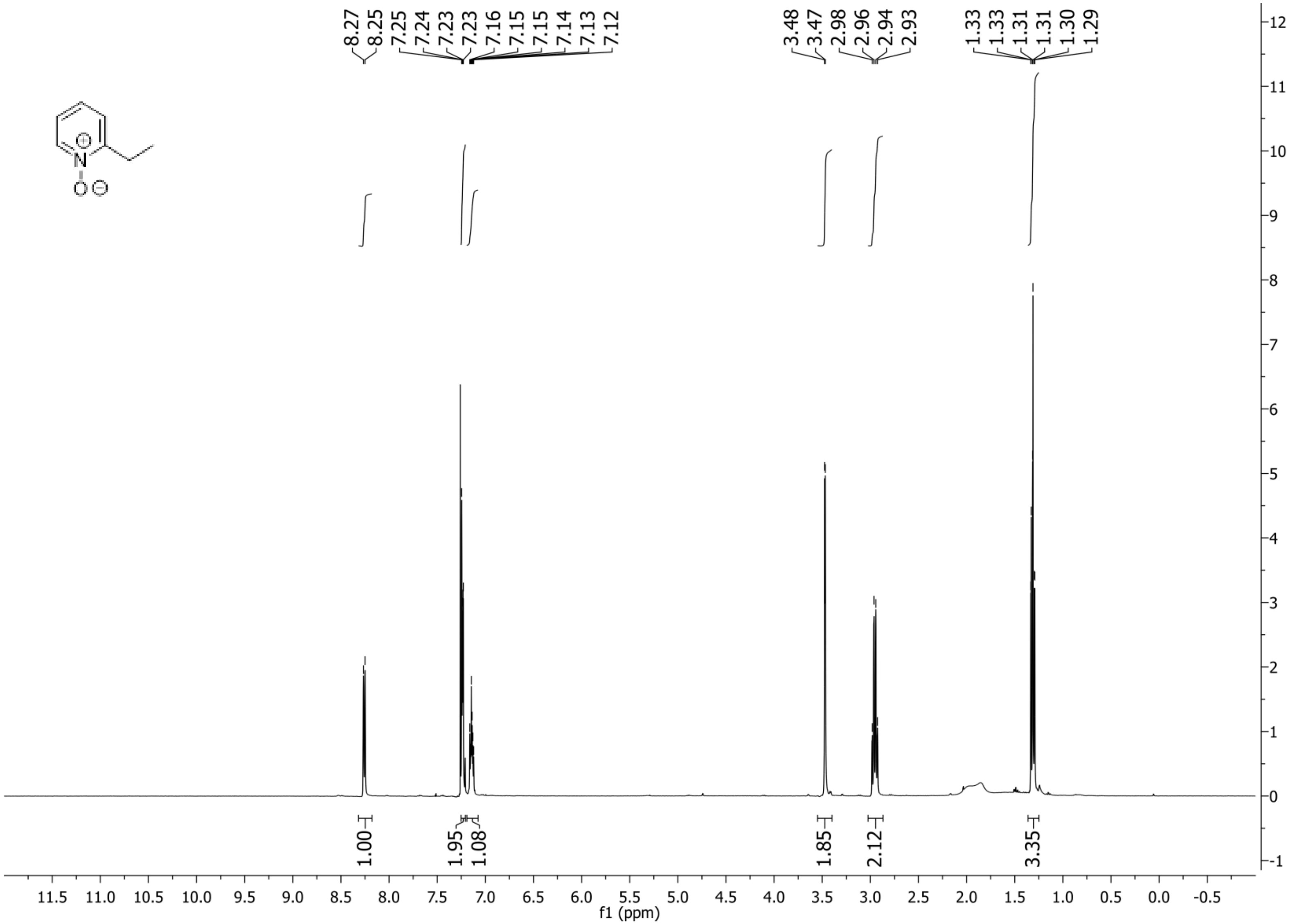

Figure S114. ^{1}H NMR (400 MHz, $CDCl_3$, 298K) of **57**.

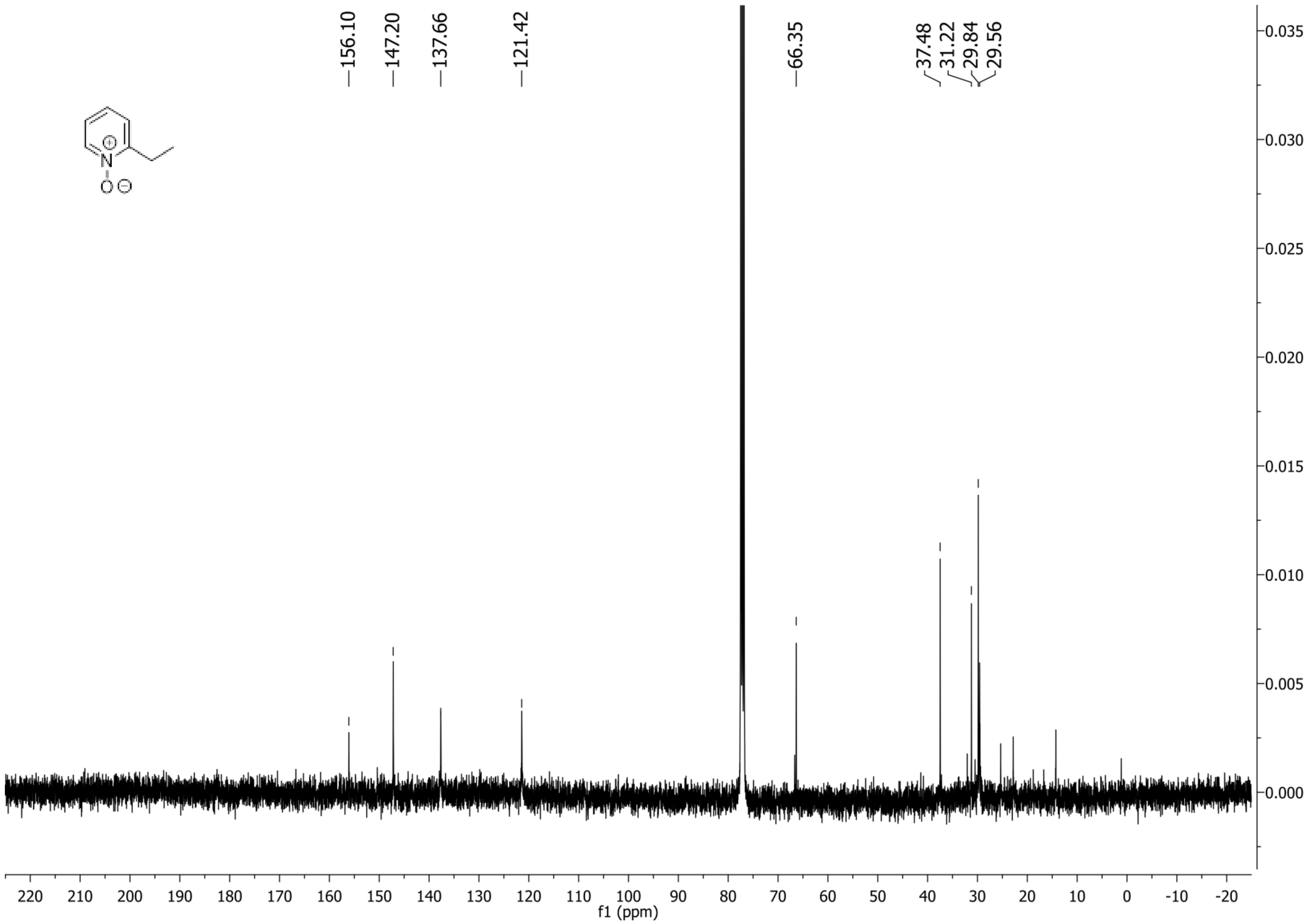

Figure S115. ^{13}C NMR (101 MHz, $CDCl_3$, 298K) of **57**.

(R)-1-(Pyridin-2-yl)-propanol (*R*)-**27**

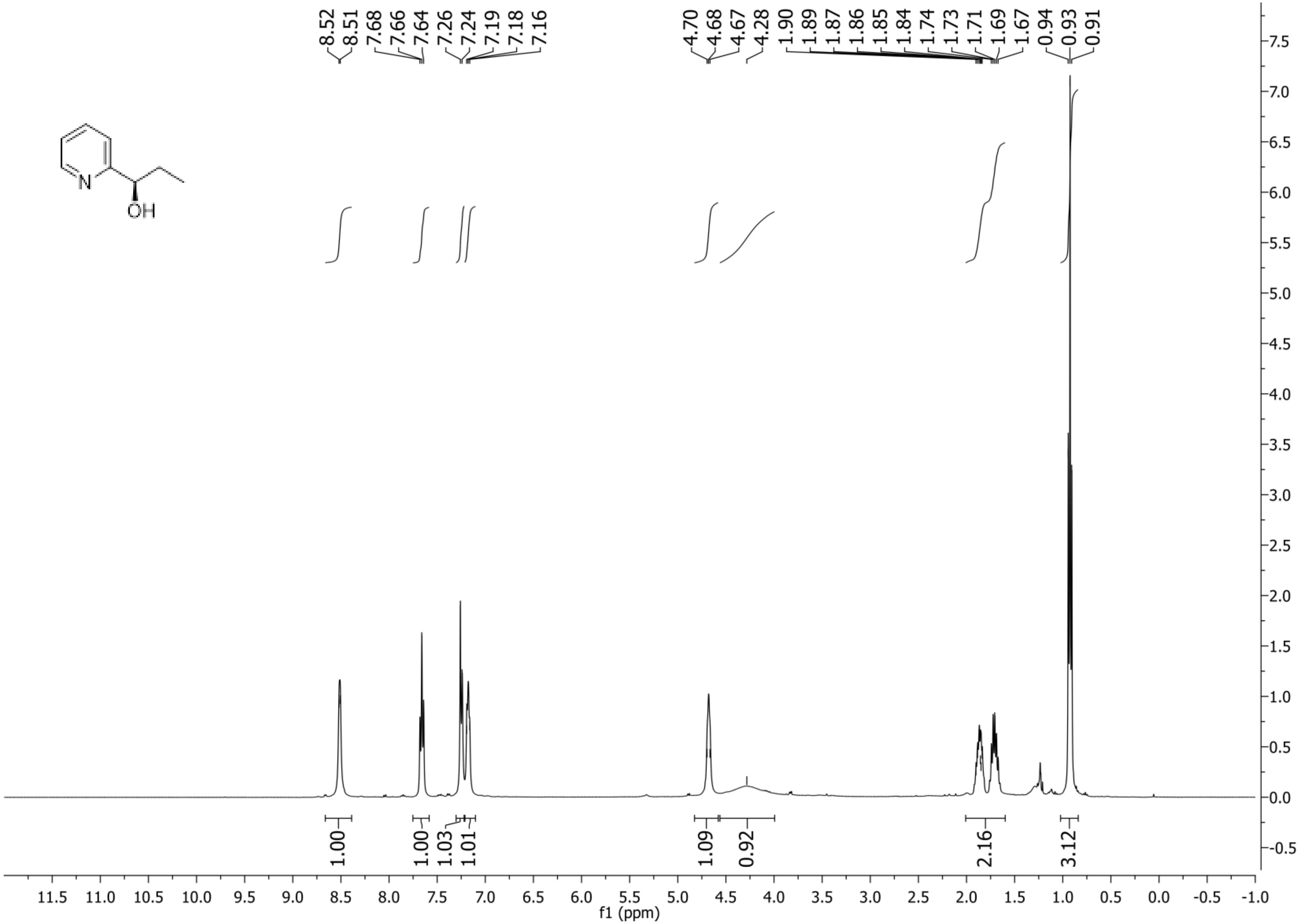

Figure S116. ^{1}H NMR (400 MHz, $CDCl_3$, 298K) of (*R*)-**27**.

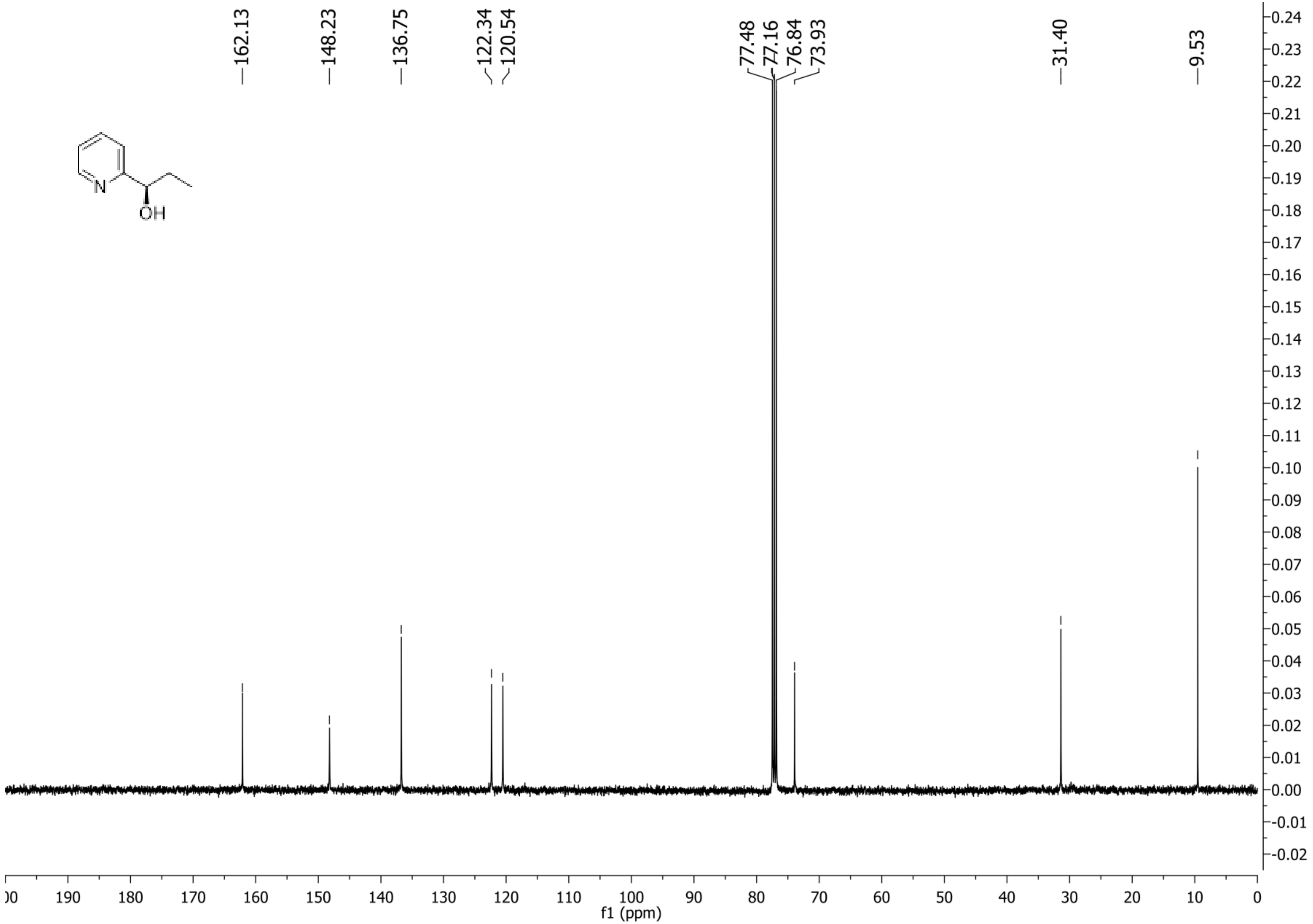

Figure S117. ^{13}C NMR (101 MHz, $CDCl_3$, 298K) of (*R*)-**27**.

1-(Pyridin-2-yl)-propan-2-ol **58**

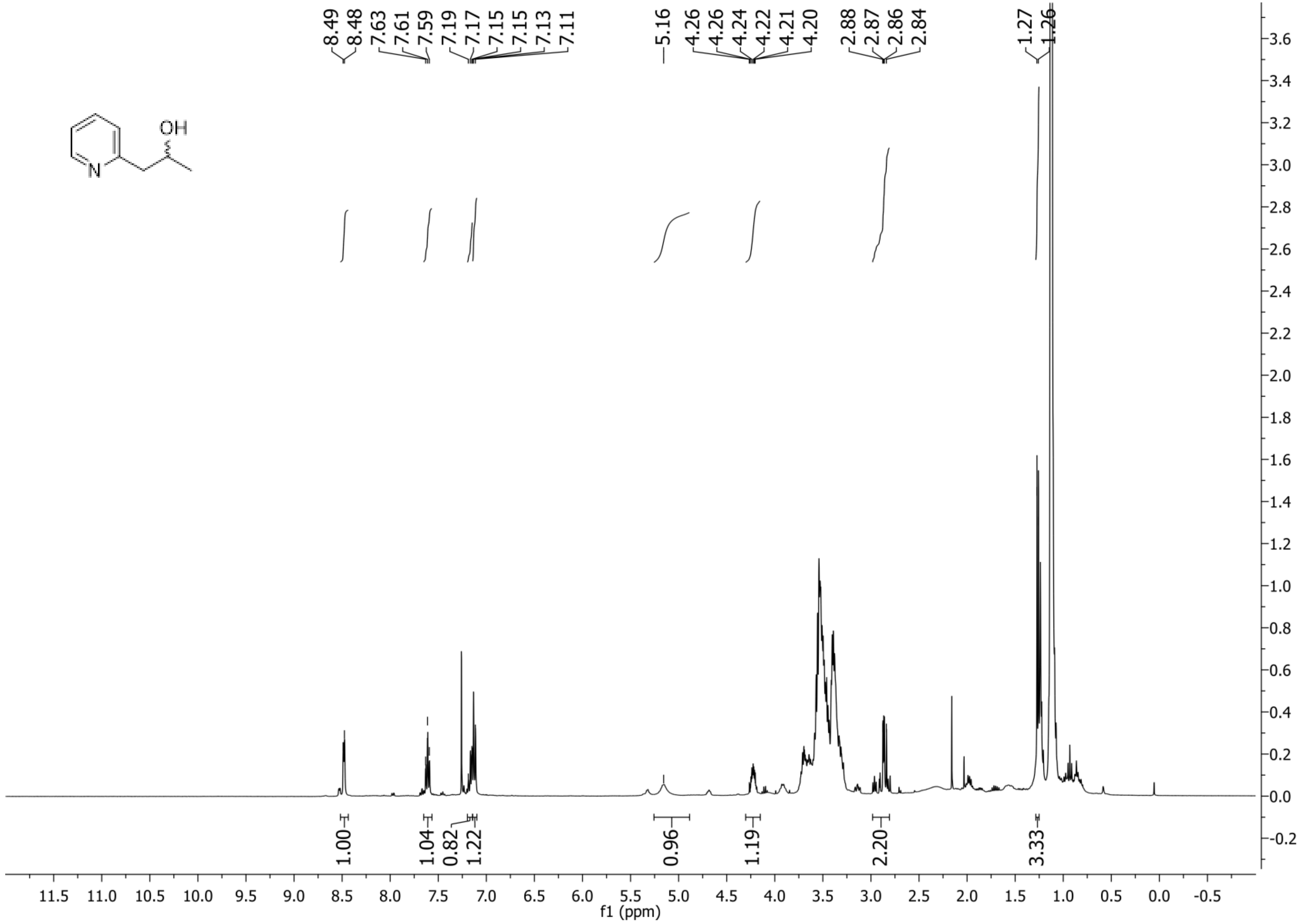

Figure S118. ^{1}H NMR (400 MHz, $CDCl_3$, 298K) of (*R*)-**27**.

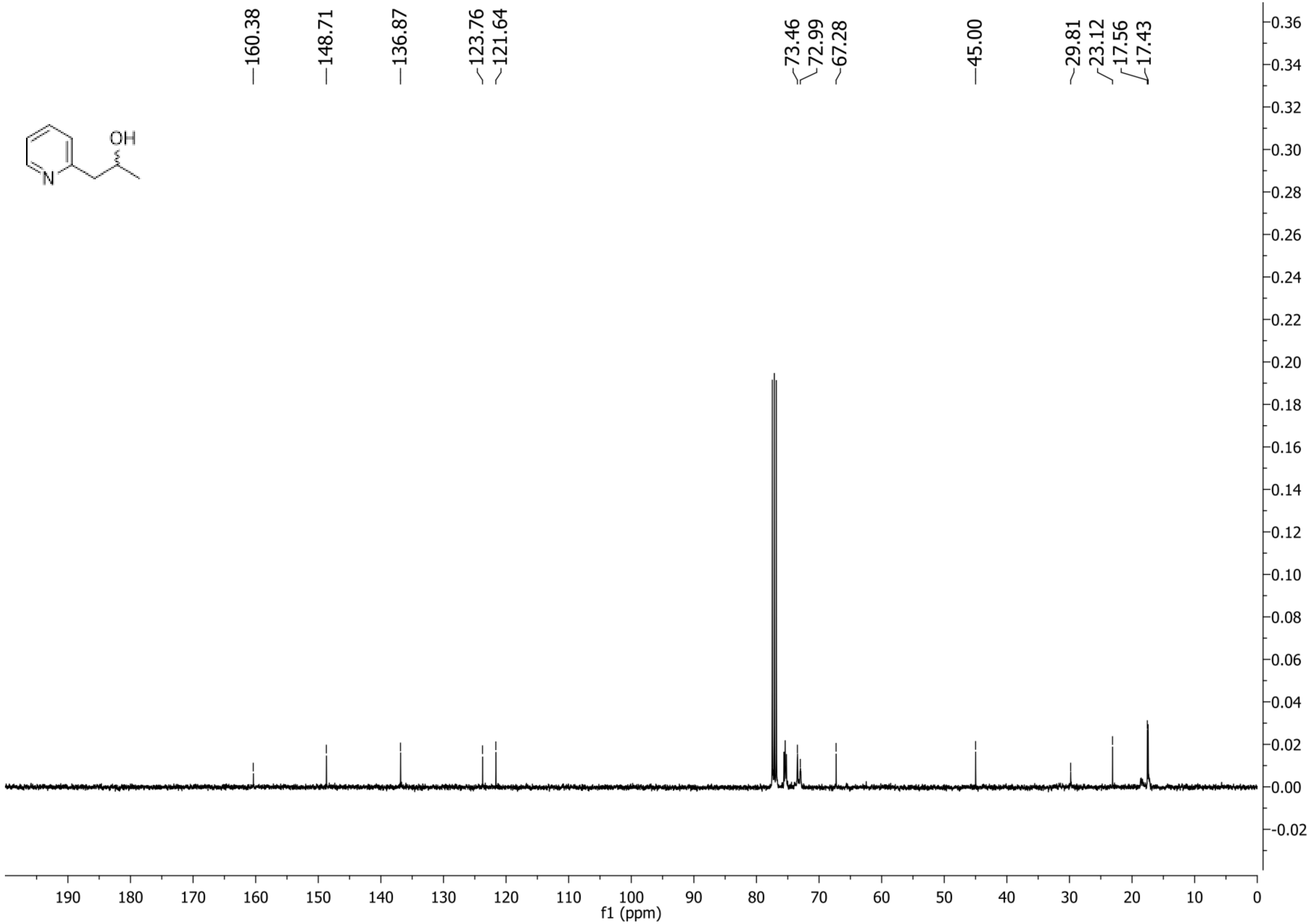

Figure S119. ^{13}C NMR (101 MHz, $CDCl_3$, 298K) of (*R*)-**27**.

2-Propylpyridine-N-oxide **59**

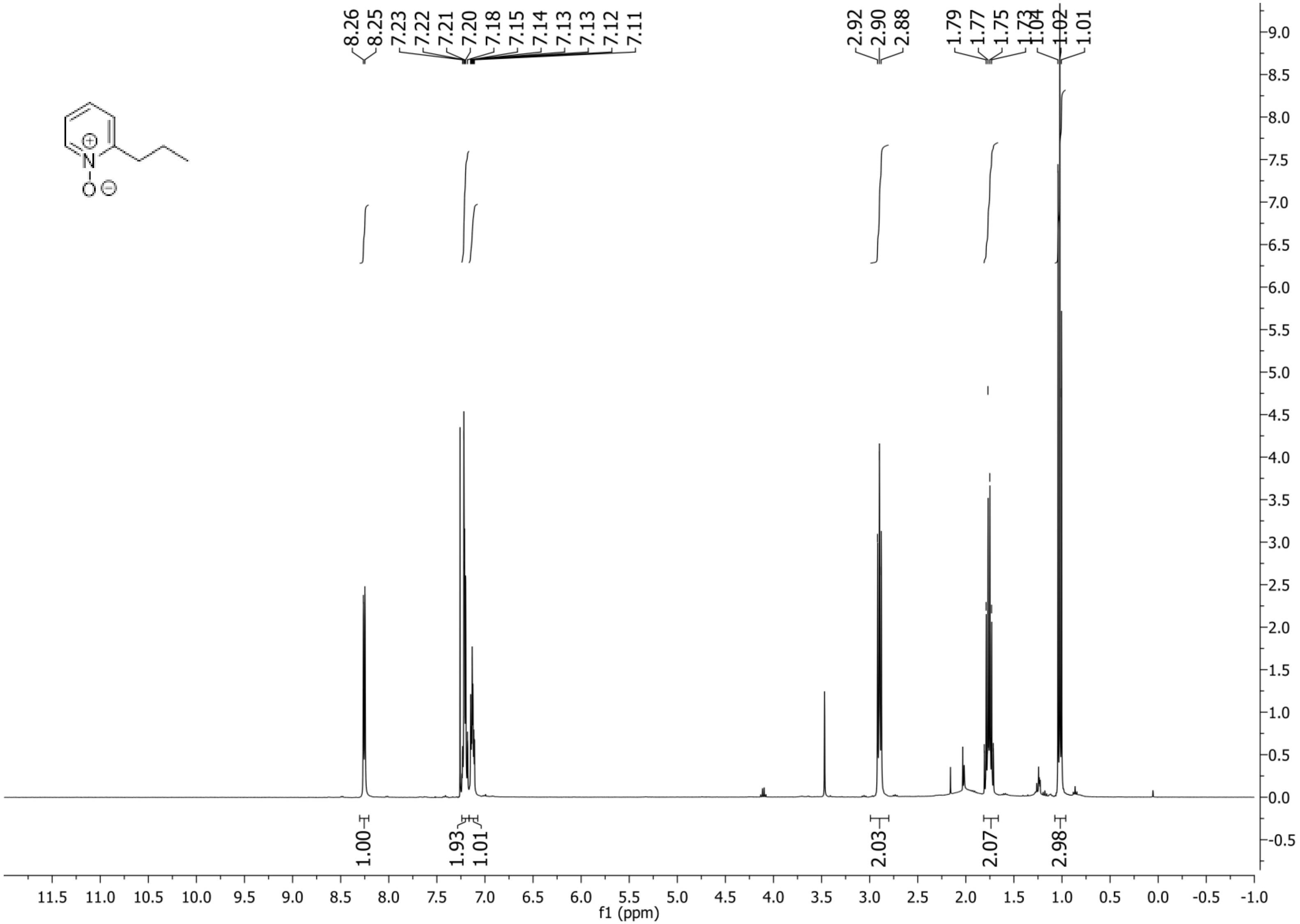

Figure S120. ^{1}H NMR (400 MHz, $CDCl_3$, 298K) of -**59**.

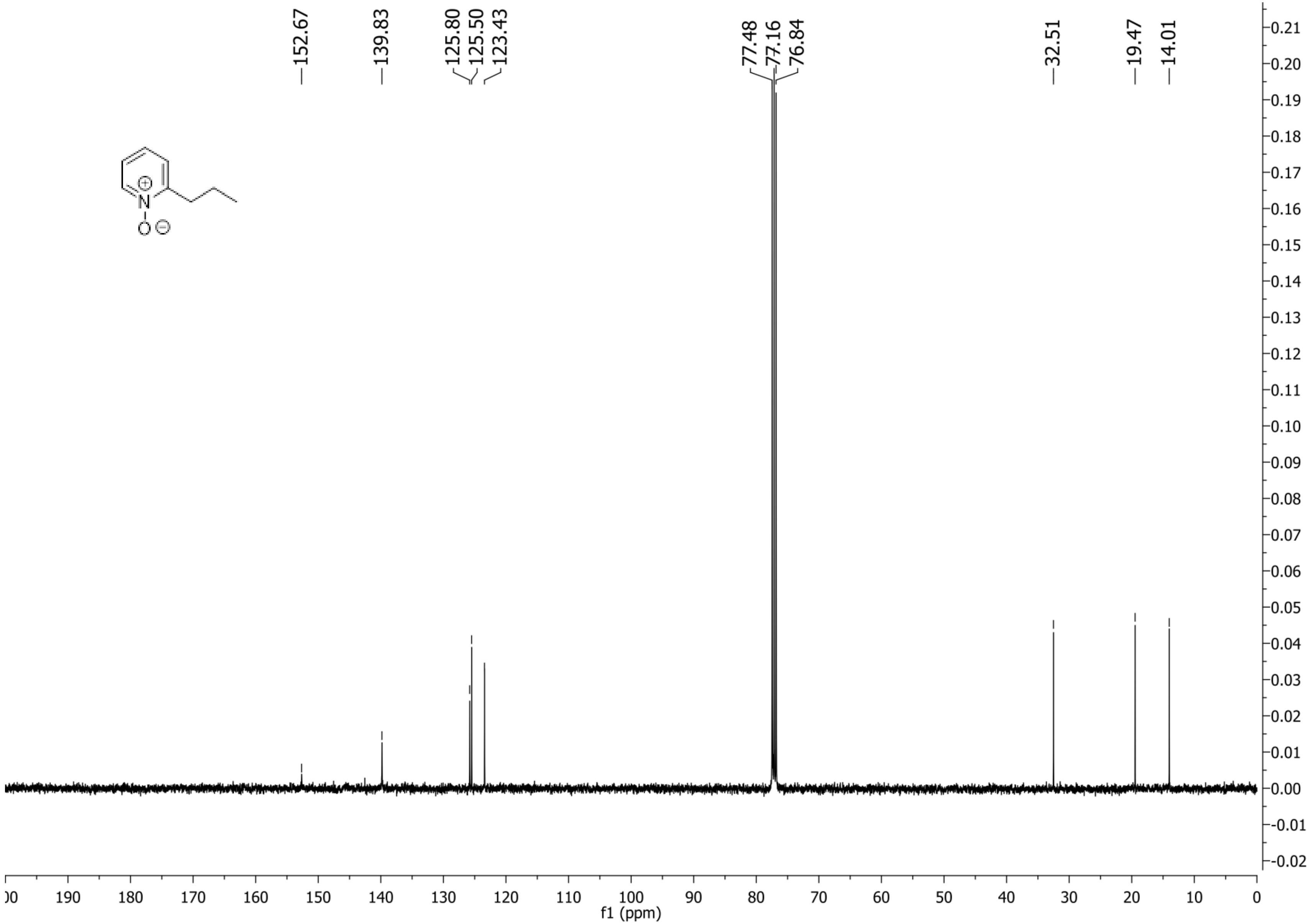

Figure S121. ^{13}C NMR (101 MHz, $CDCl_3$, 298K) of -**59**.

(6-Methoxypyridin-3-yl)-methanol **28**

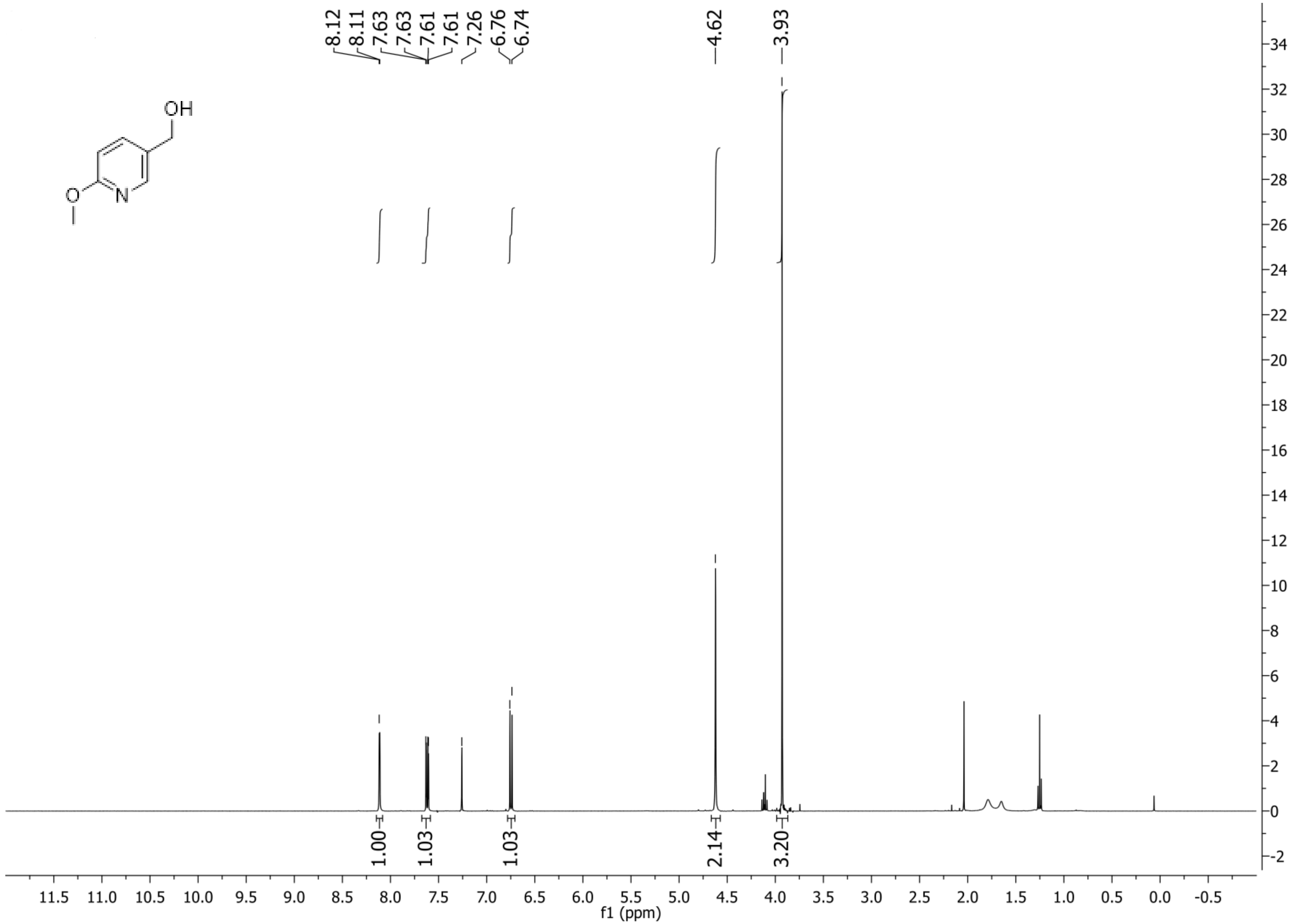

Figure S122. ^{1}H NMR (400 MHz, $CDCl_3$, 298K) of -**28**.

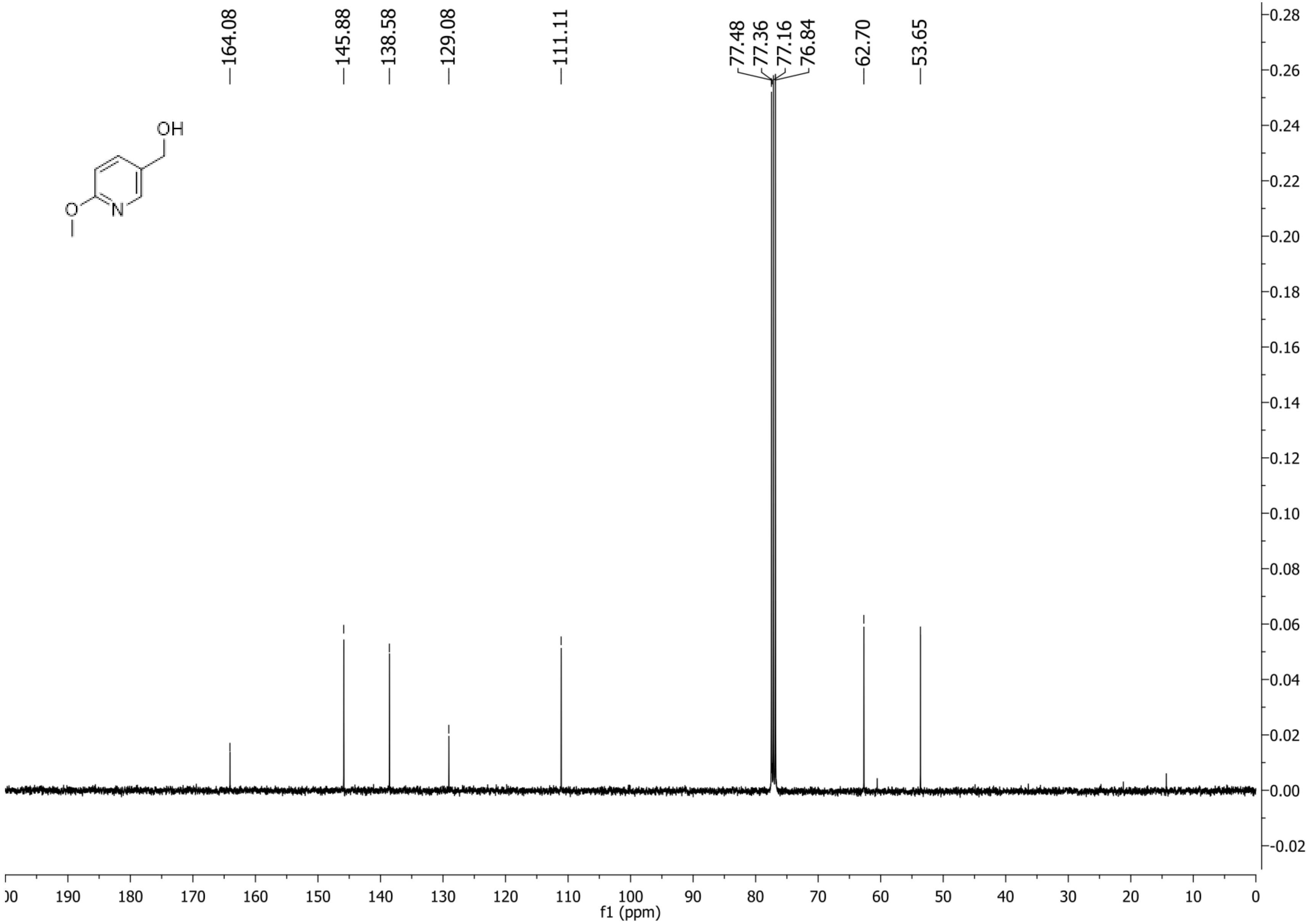

Figure S123. ^{13}C NMR (101 MHz, $CDCl_3$, 298K) of -**28**.

6-Methoxynicotinaldehyde **29**

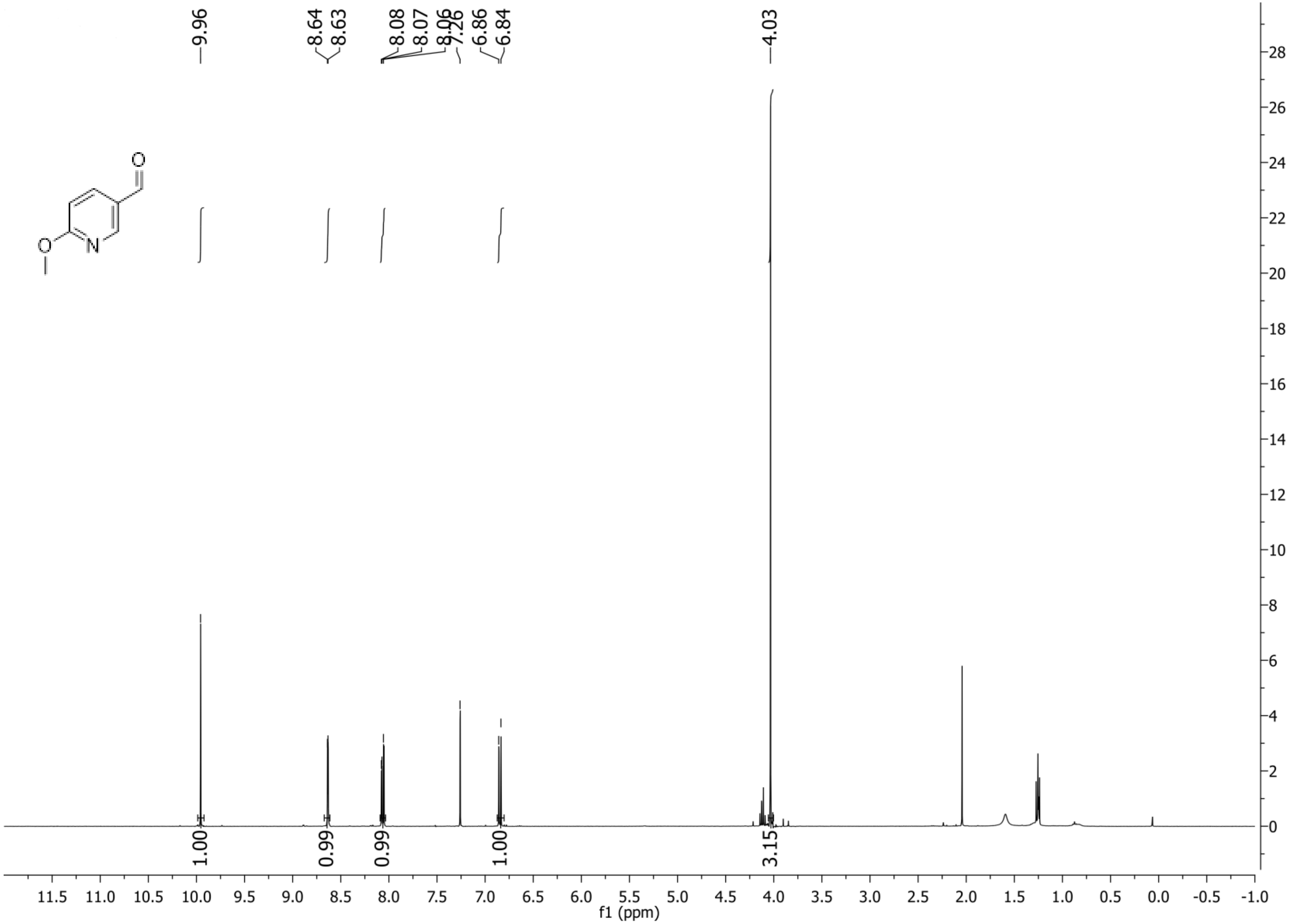

Figure S124. ^{1}H NMR (400 MHz, $CDCl_3$, 298K) of -**29**.

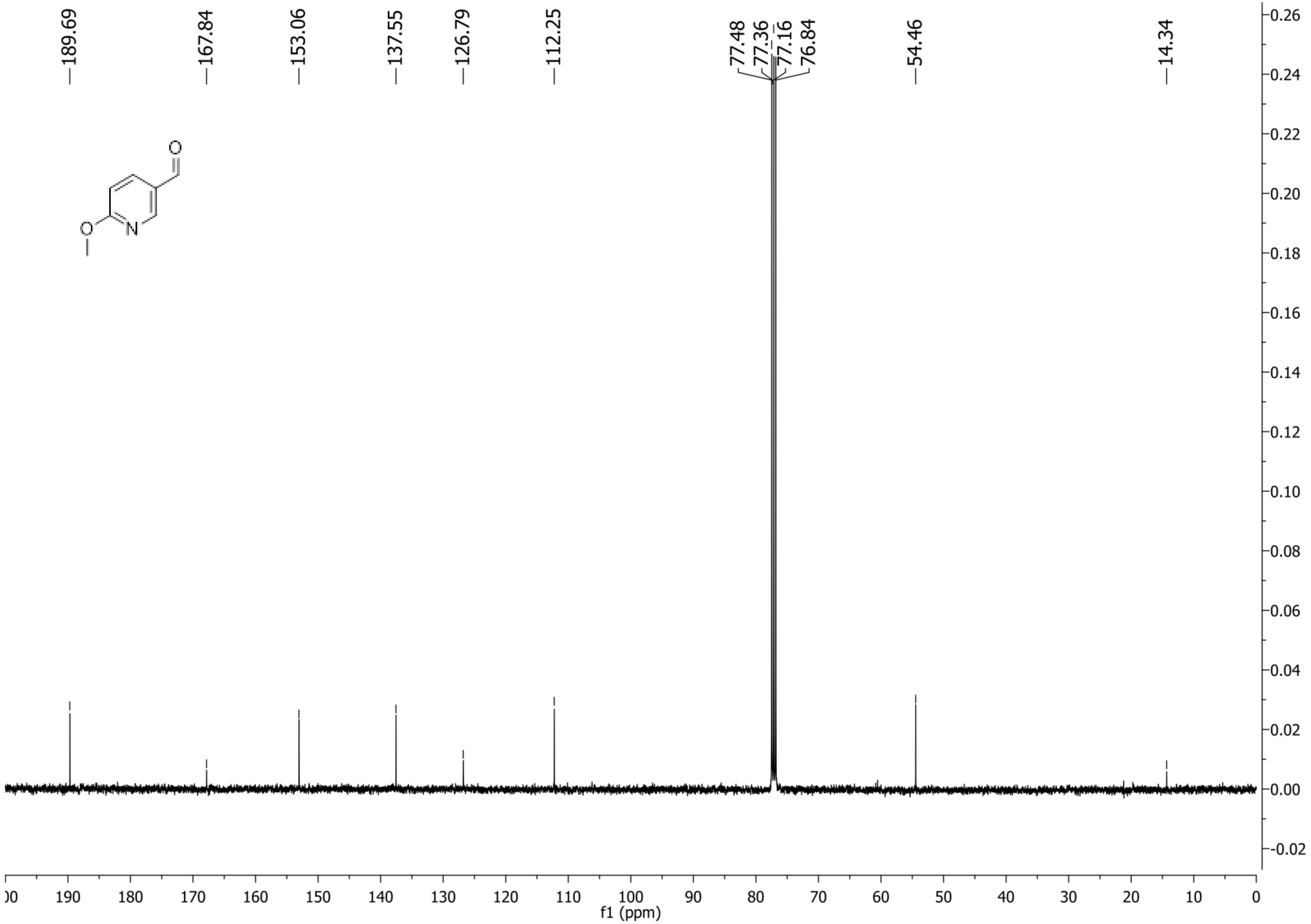

Figure S125. ^{13}C NMR (101 MHz, $CDCl_3$, 298K) of -**29**.

1-(6-Methylpyridin-3-yl)-ethan-1-ol **61a**

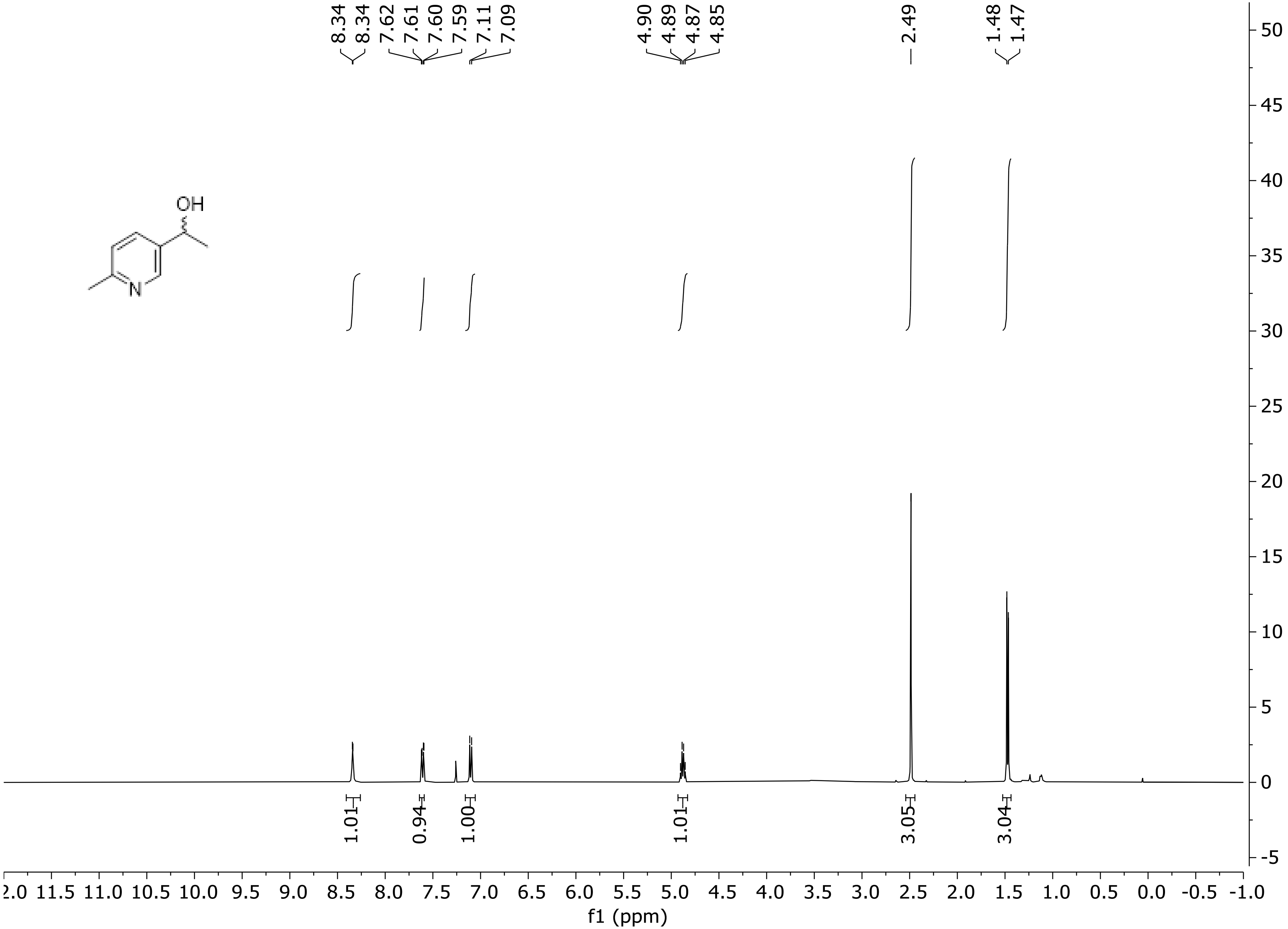

Figure S126. ^{1}H NMR (400 MHz, $CDCl_3$, 298K) of **-61a**.

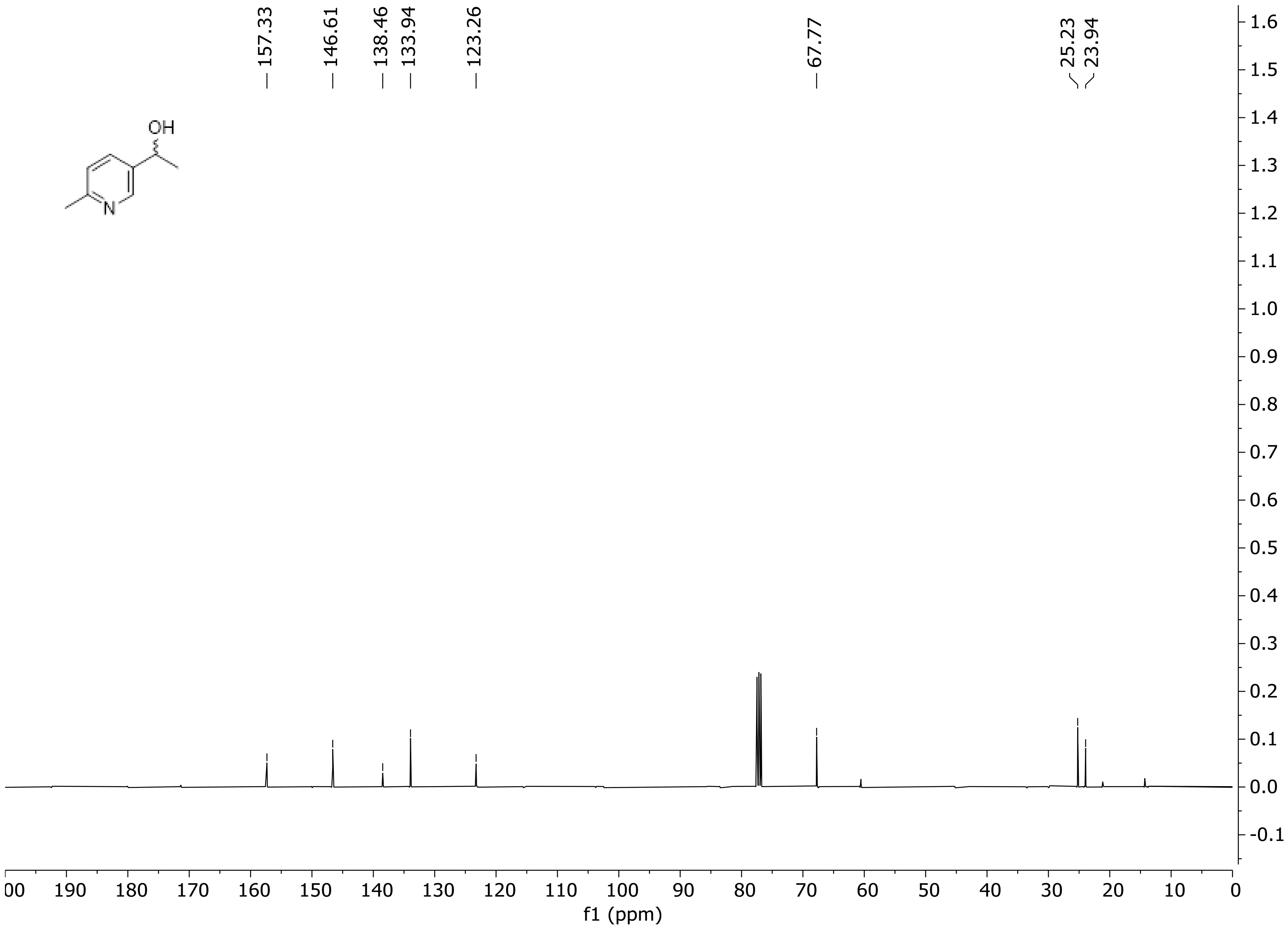

Figure S127. ^{13}C NMR (101 MHz, $CDCl_3$, 298K) of **-61a**.

Mixture of *2,5-Dimethylpyridine* **62**, *(6-Methylpyridin-3-yl)-methanol* **63a**, *6-Methylnicotinaldehyde* **63b**, *6-Methylnicotinic acid* **63c**

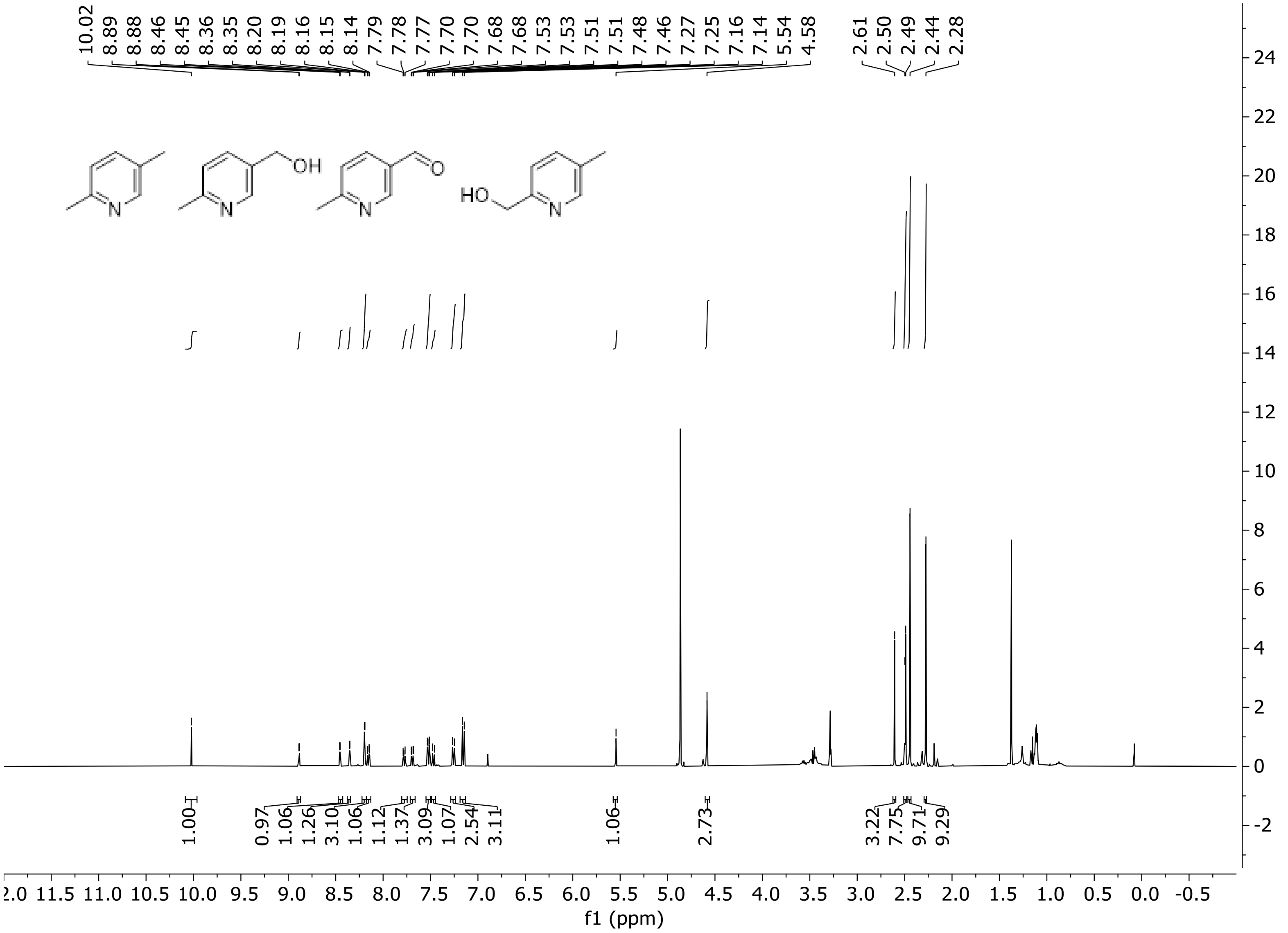

Figure S128. ^{1}H NMR (400 MHz, MeOH-d_4, 298K) of -**62**, **63a**, **63b**, **63c**.

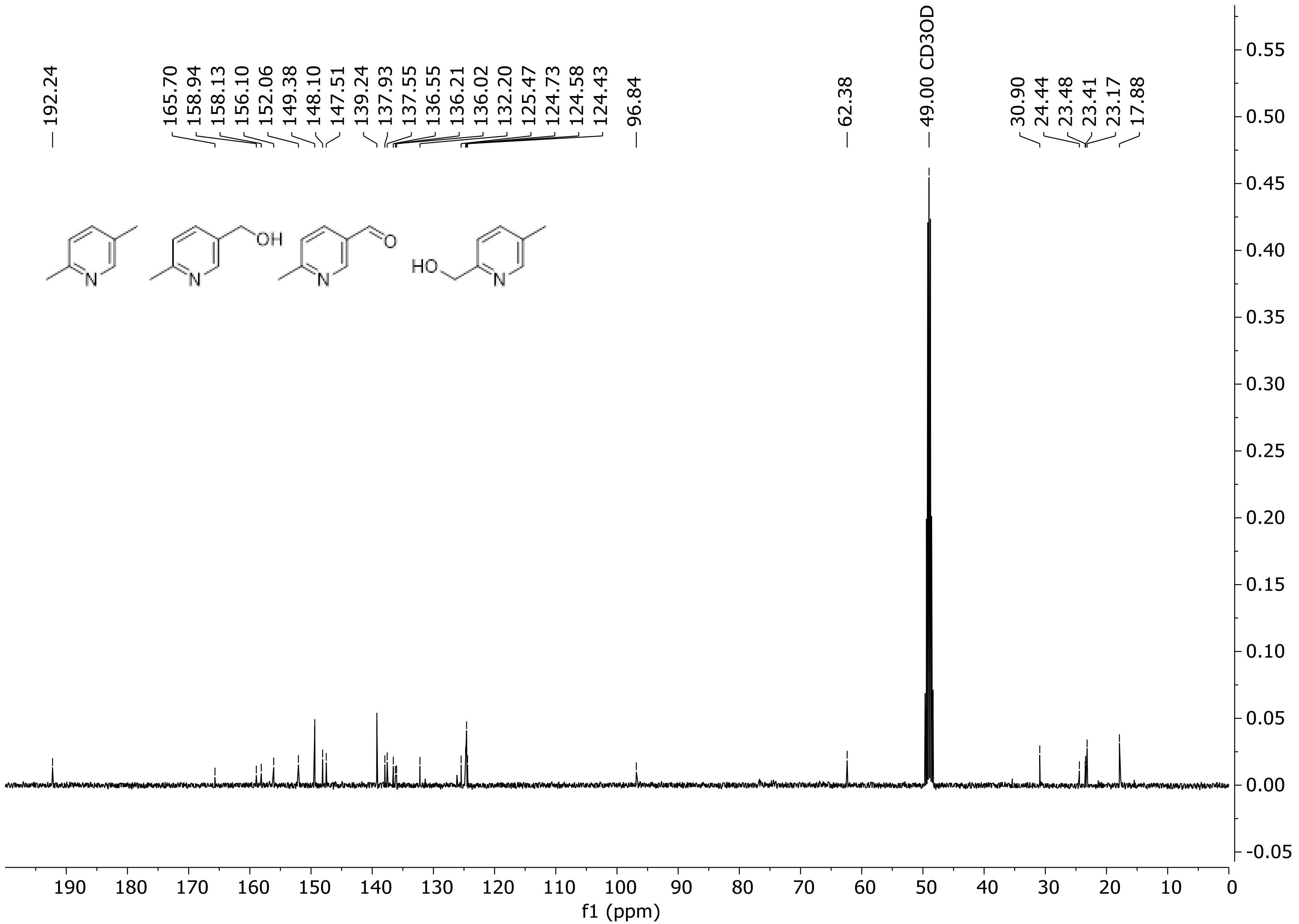

Figure S129. ^{13}C NMR (101 MHz, MeOH-d_4, 298K) of -**62**, **63a**, **63b**, **63c**.

(6-Methylpyridin-3-yl)-methanol **63a**

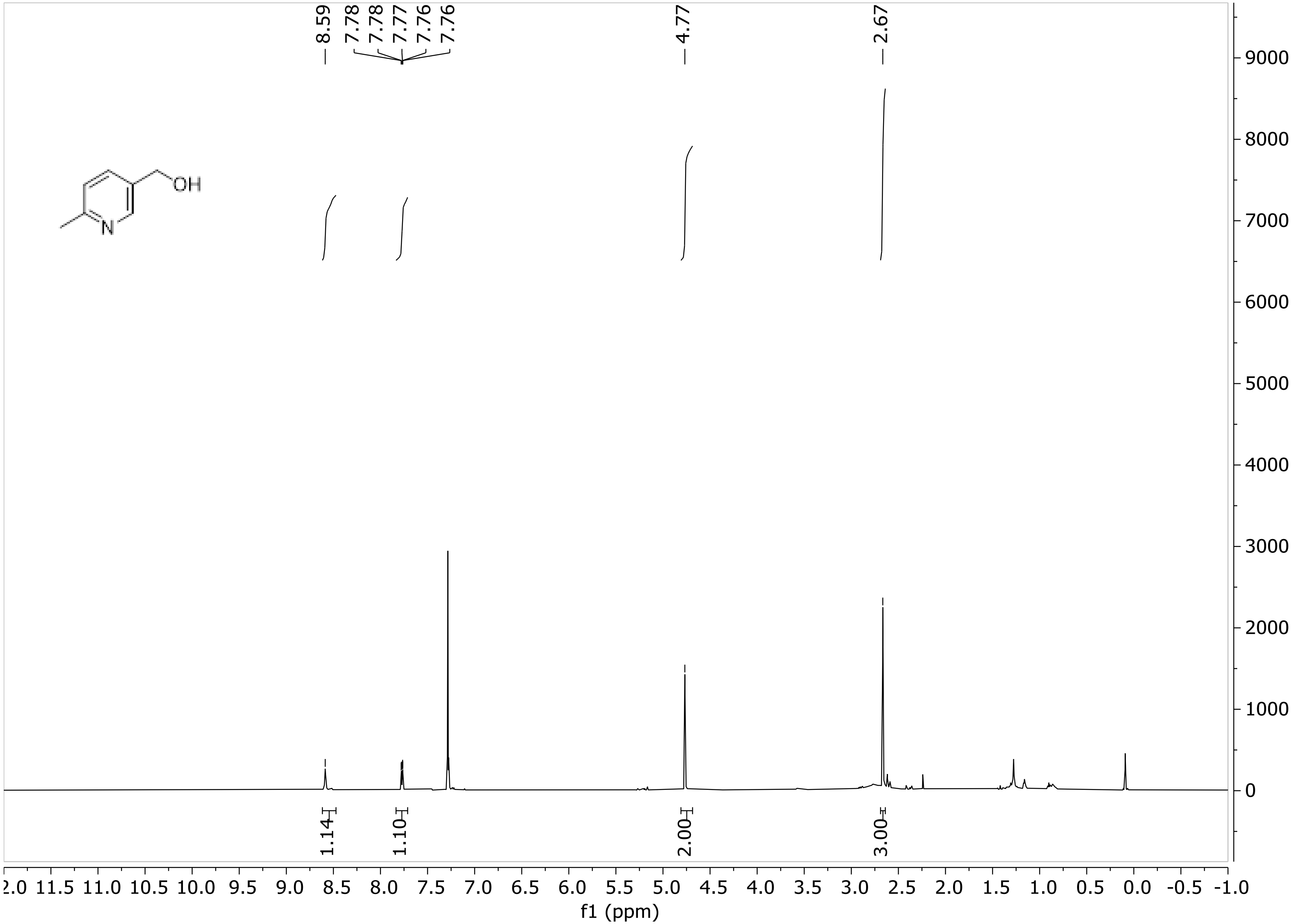

Figure S130. ^{1}H NMR (600 MHz, $CDCl_3$, 298K) of **63a**.

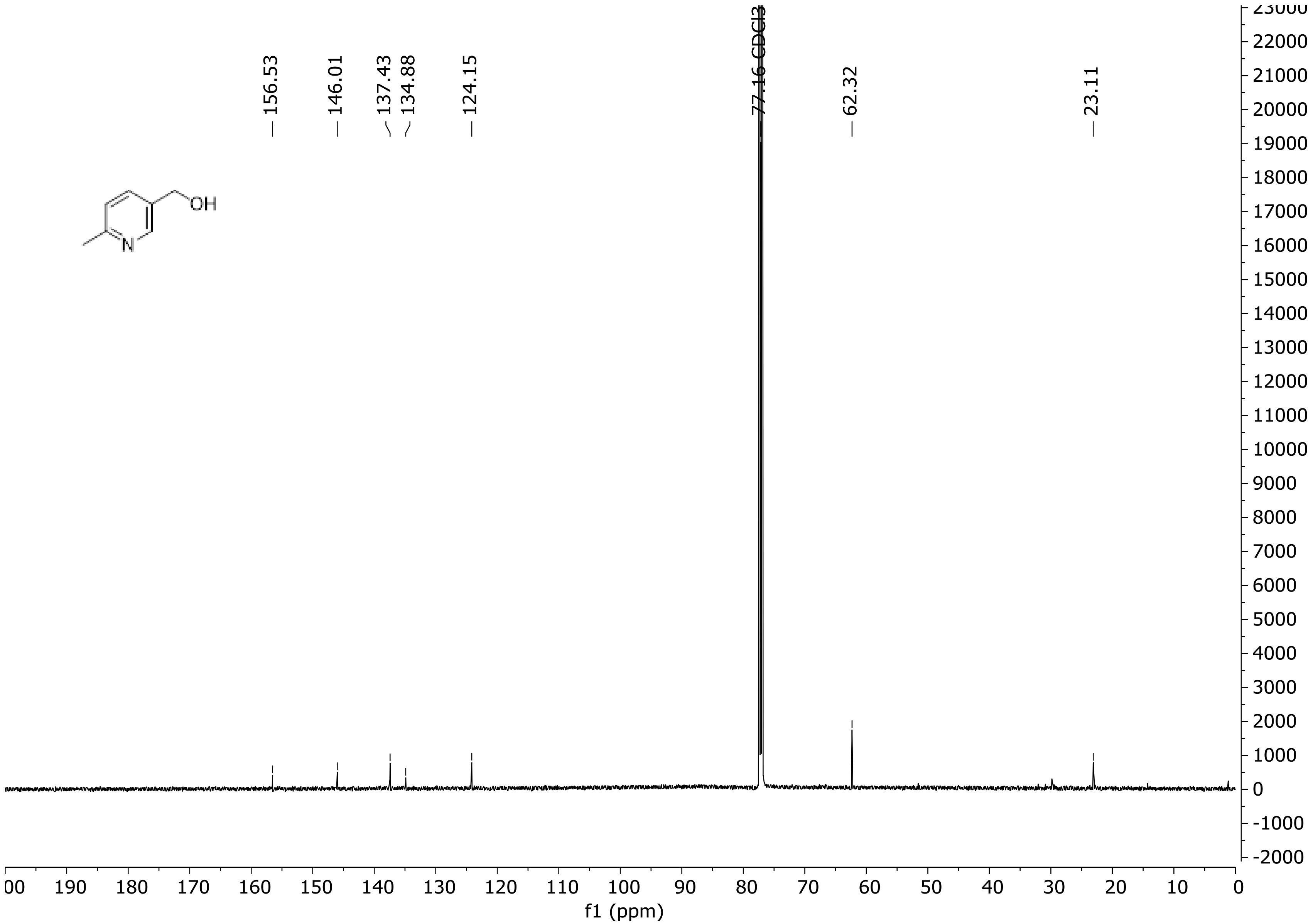

Figure S131. ^{13}C NMR (151 MHz, $CDCl_3$, 298K) of **63a**.

6-Methylnicotinaldehyde **63b**

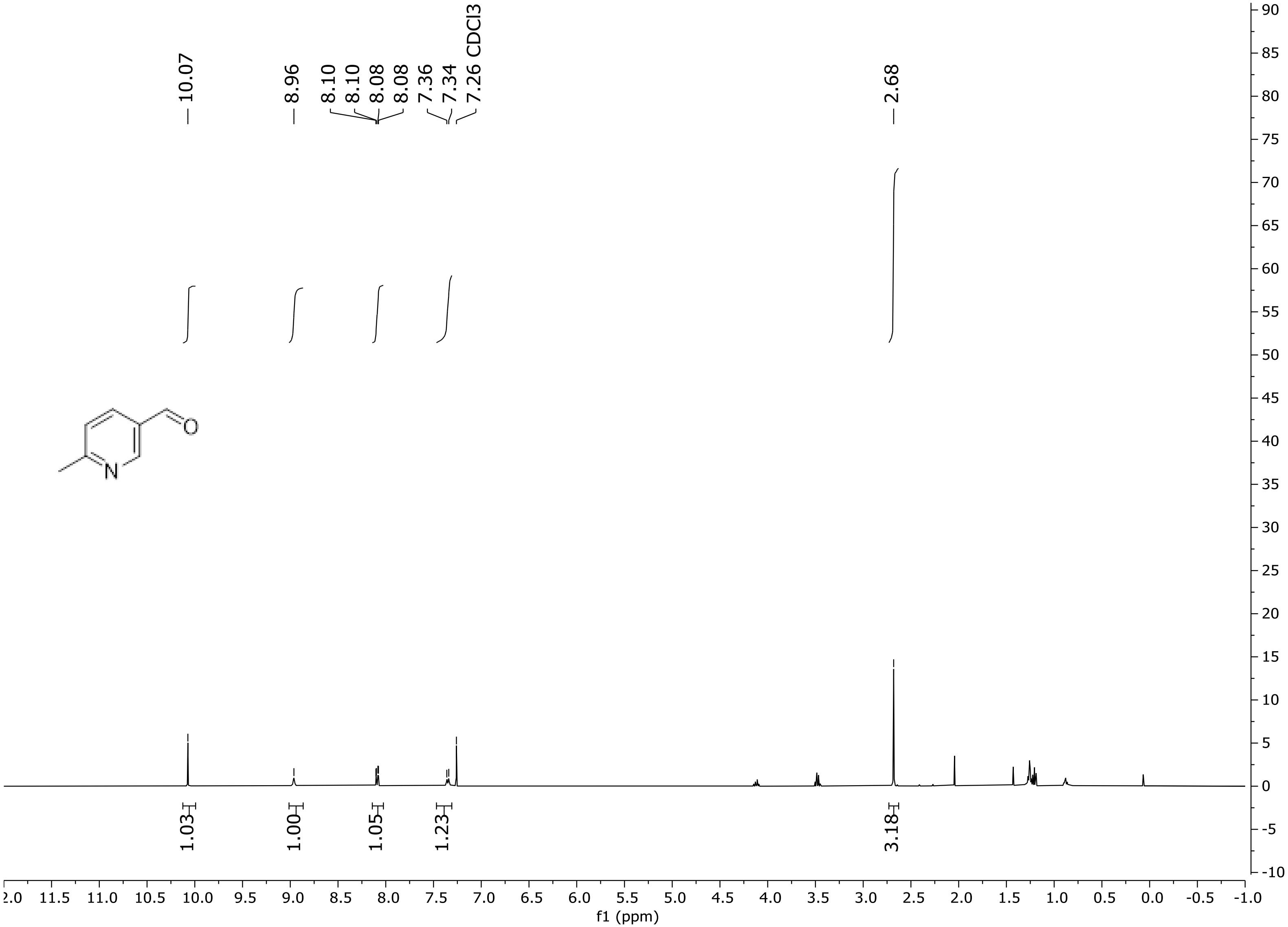

Figure S132. ^{1}H NMR (400 MHz, $CDCl_3$, 298K) of **63b**.

Mixture of *3,5-Dimethylpyridine* **64**, *(5-Methylpyridin-3-yl)-methanol* **65a** and *5-Methylnicotinaldehyde* **65b**

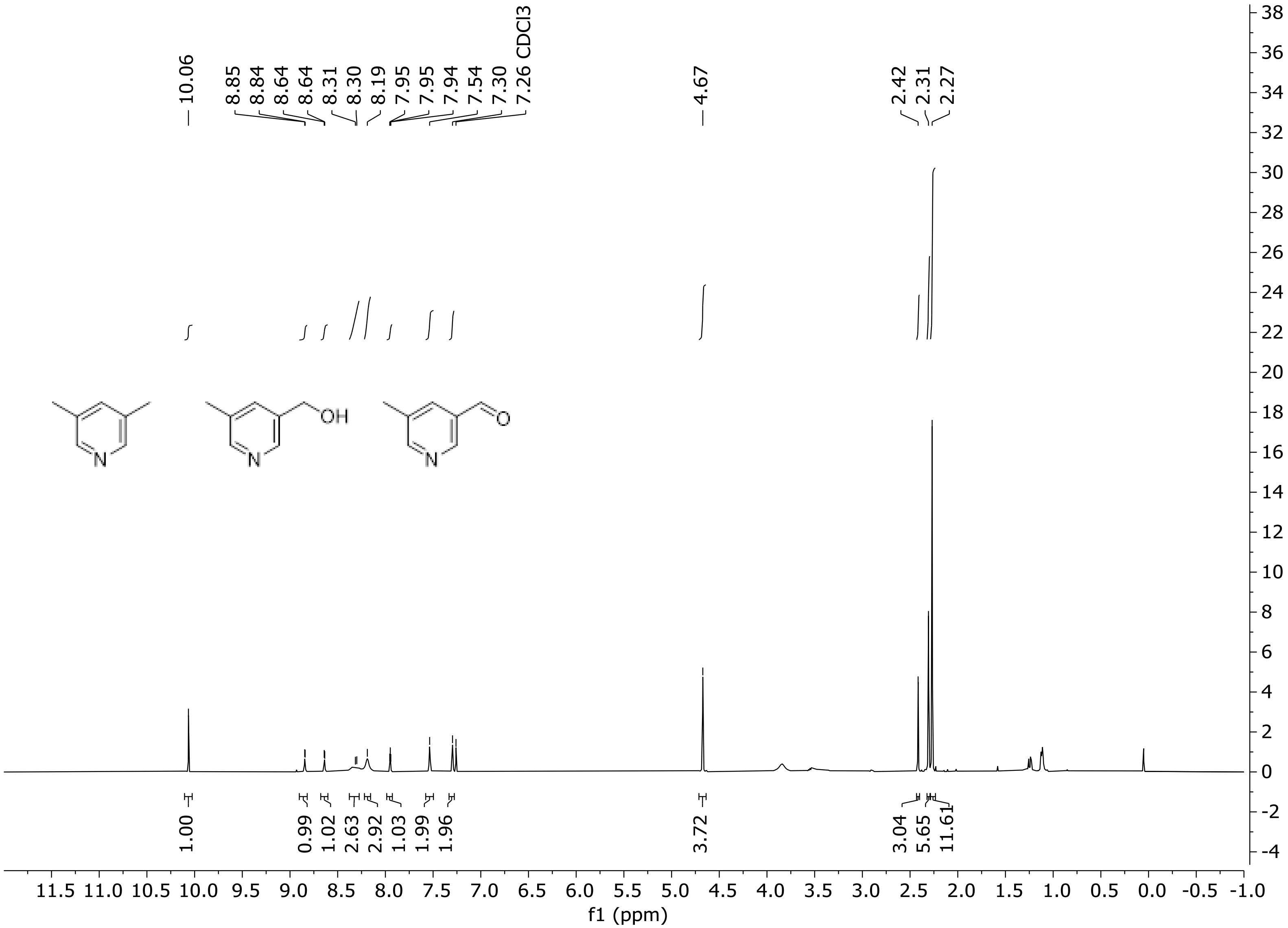

Figure S133. ^{1}H NMR (400 MHz, $CDCl_3$, 298K) of **64**, **65a**, **65b**.

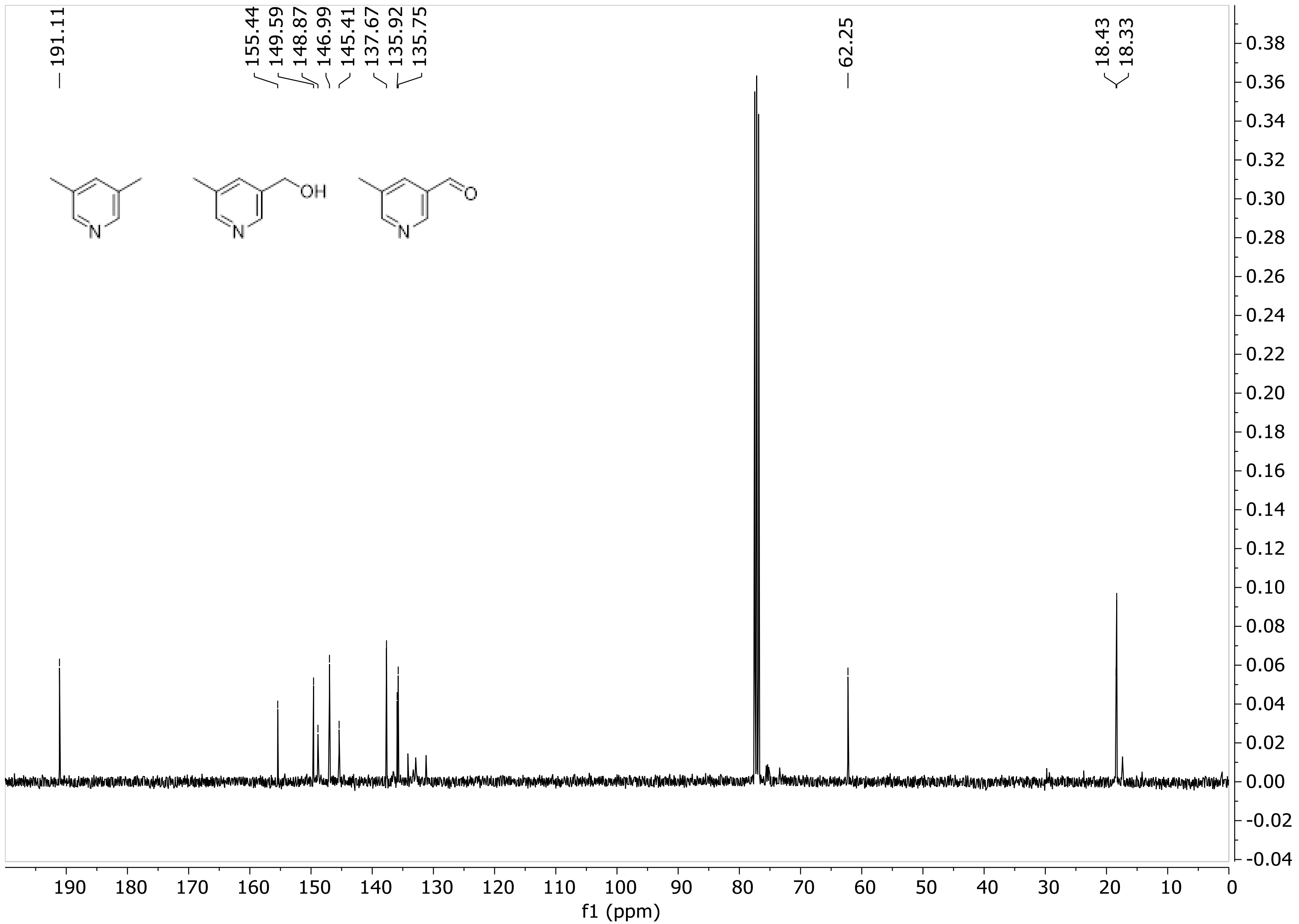

Figure S134. ^{13}C NMR (101 MHz, $CDCl_3$, 298K) of **64**, **65a**, **65b**.

(+)-1-(6-Methoxypyridin-3-yl)-ethan-1-ol (*R*)-**67a**

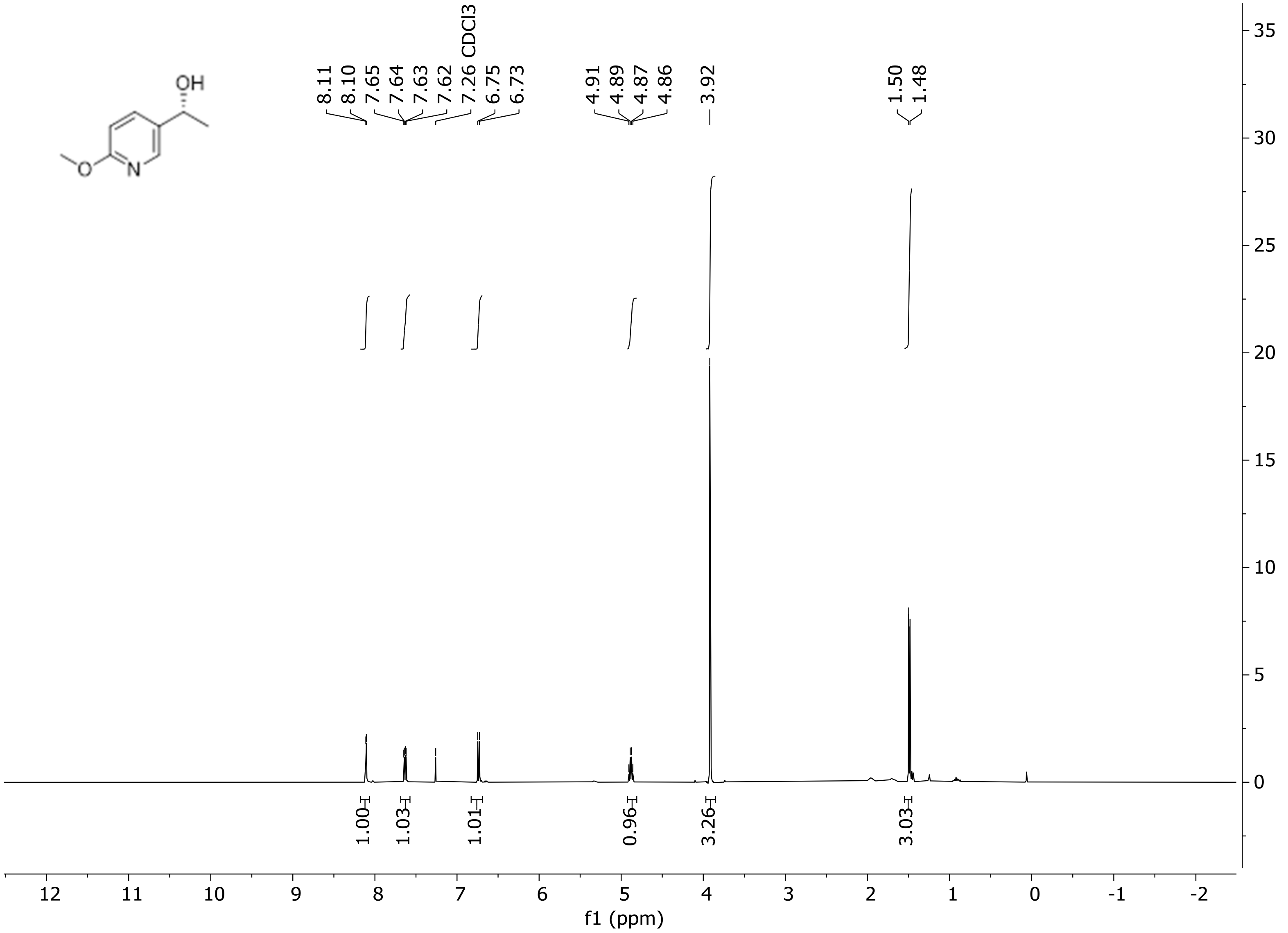

Figure S135. ^{1}H NMR (400 MHz, $CDCl_3$, 298K) of (*R*)-**67a**.

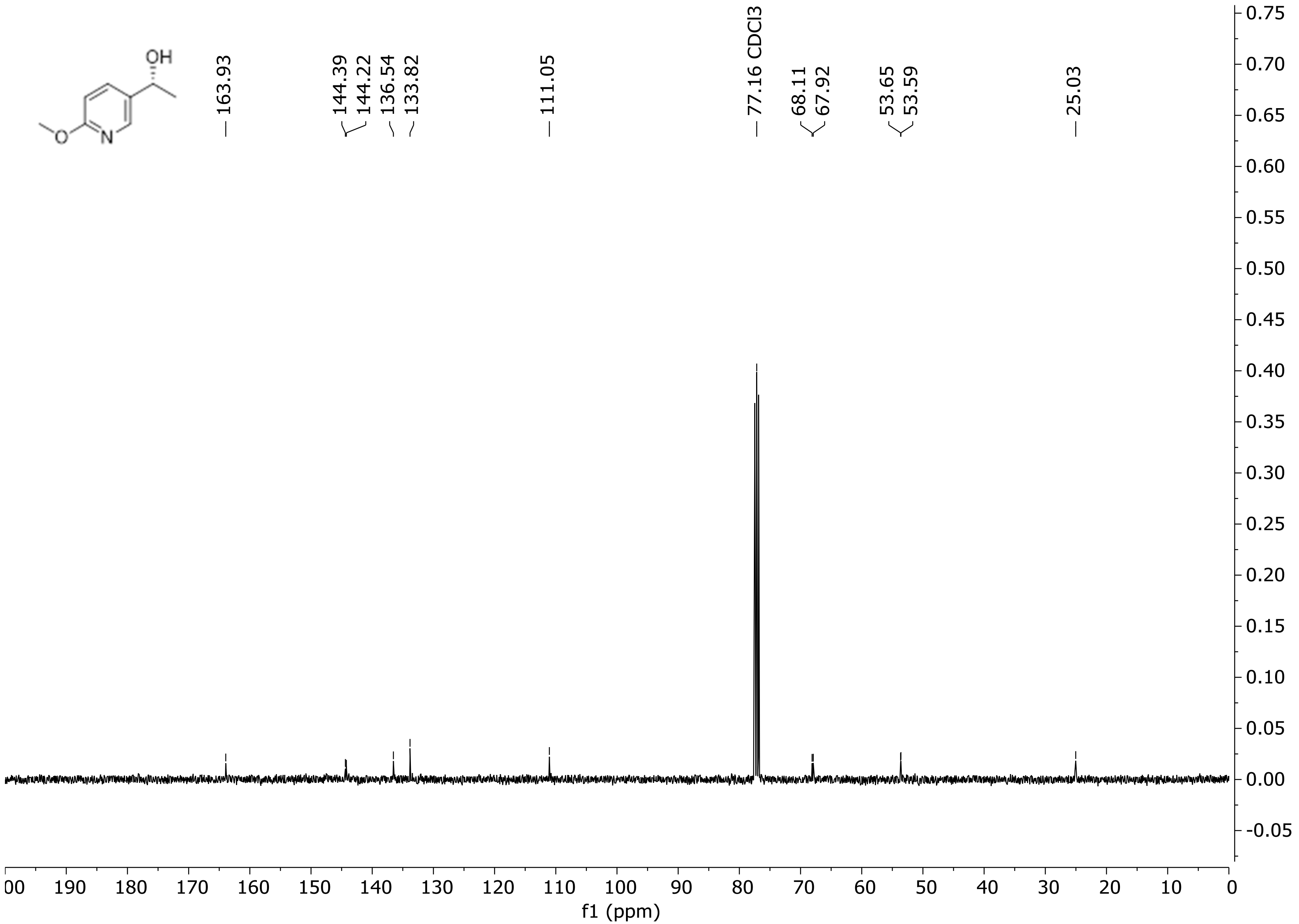

Figure S136. ^{13}C NMR (101 MHz, $CDCl_3$, 298K) of (*R*)-**67a**.

1-(6-Methoxypyridin-3-yl)-ethan-1-one **67b**

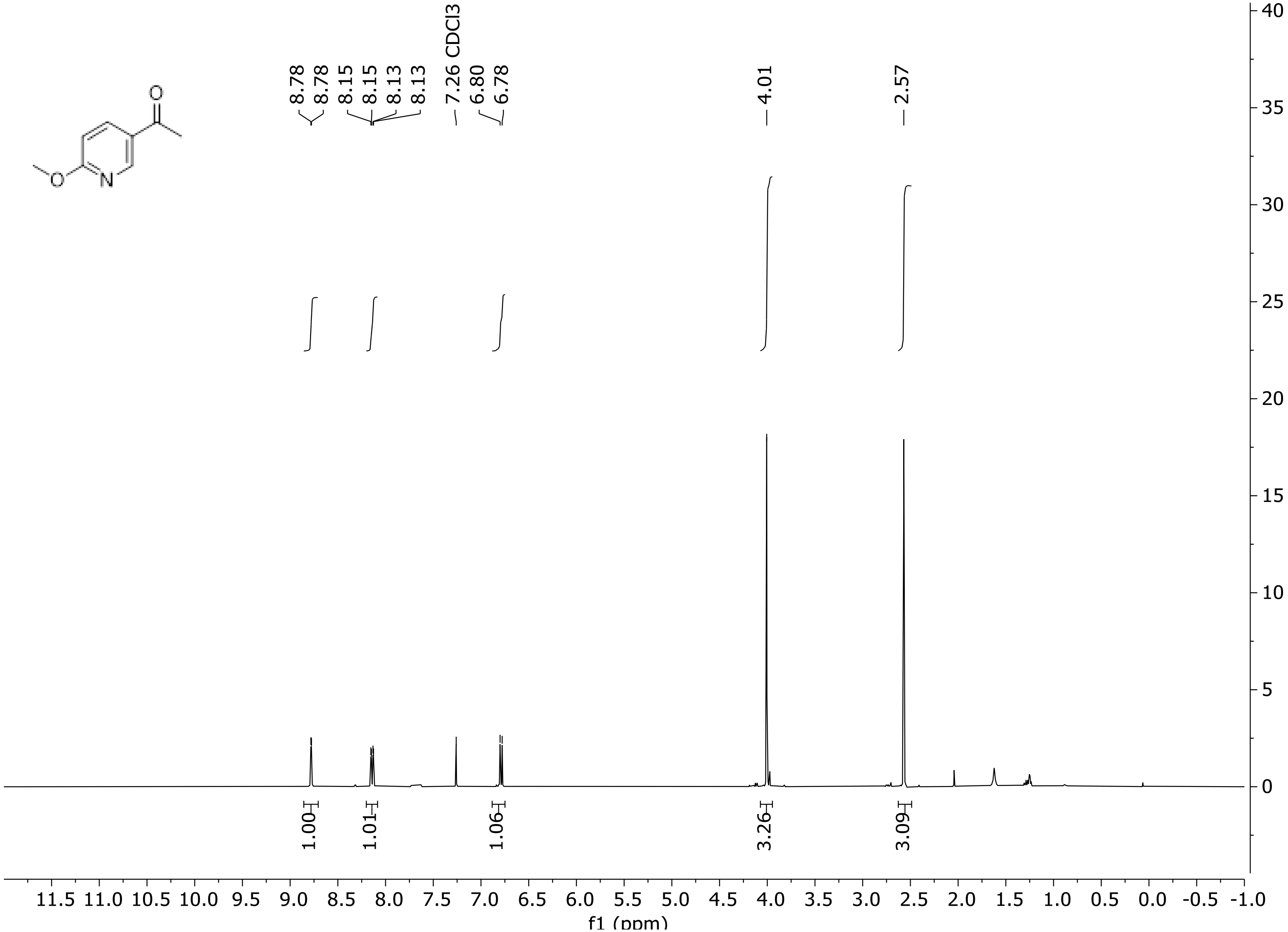

Figure S137. ^{1}H NMR (400 MHz, $CDCl_3$, 298K) of **67b**.

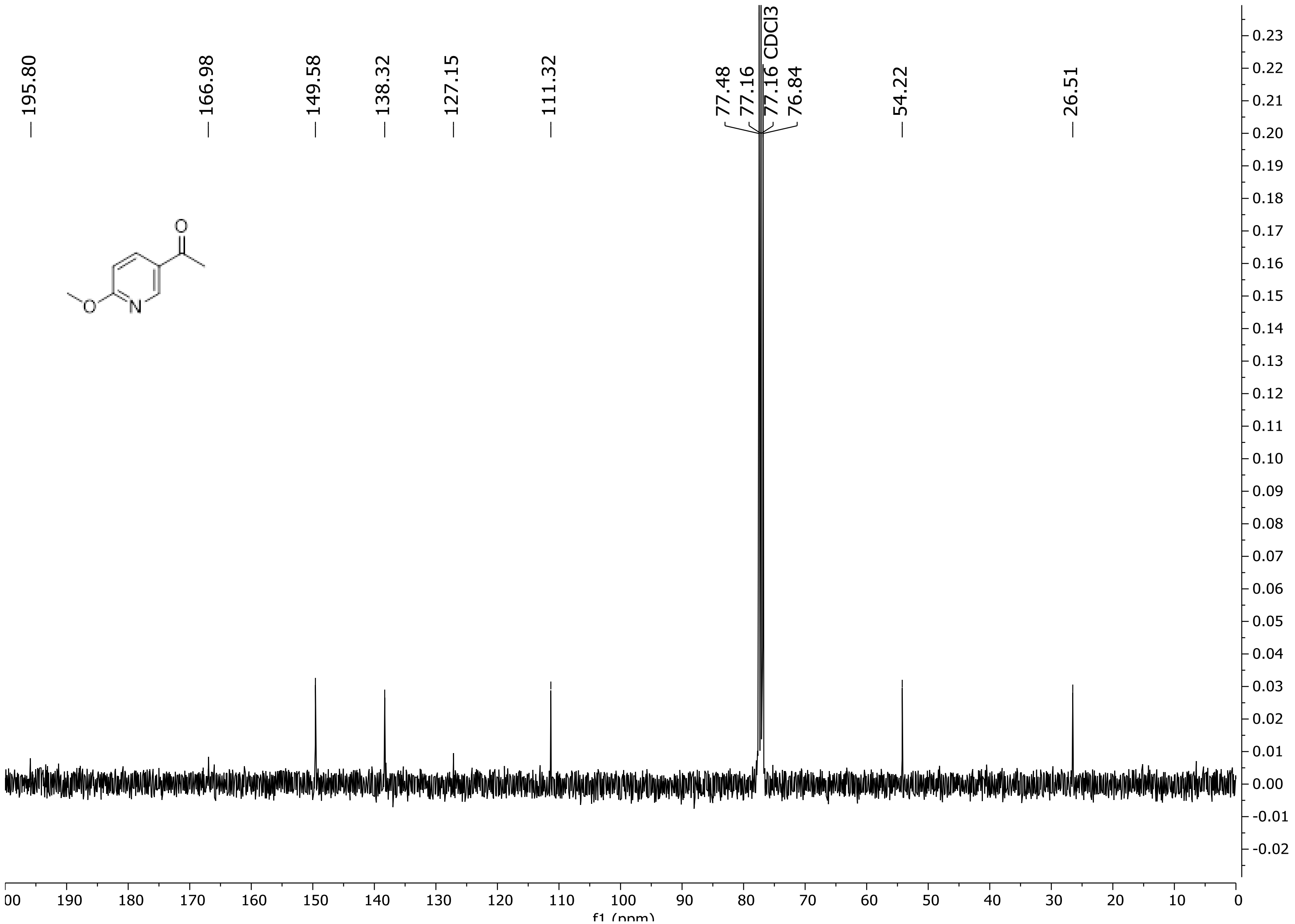

Figure S138. ^{13}C NMR (101 MHz, $CDCl_3$, 298K) of **67b**.

(+)-(6-Chloropyridin-3-yl)-ethan-1-ol (+)-**72**

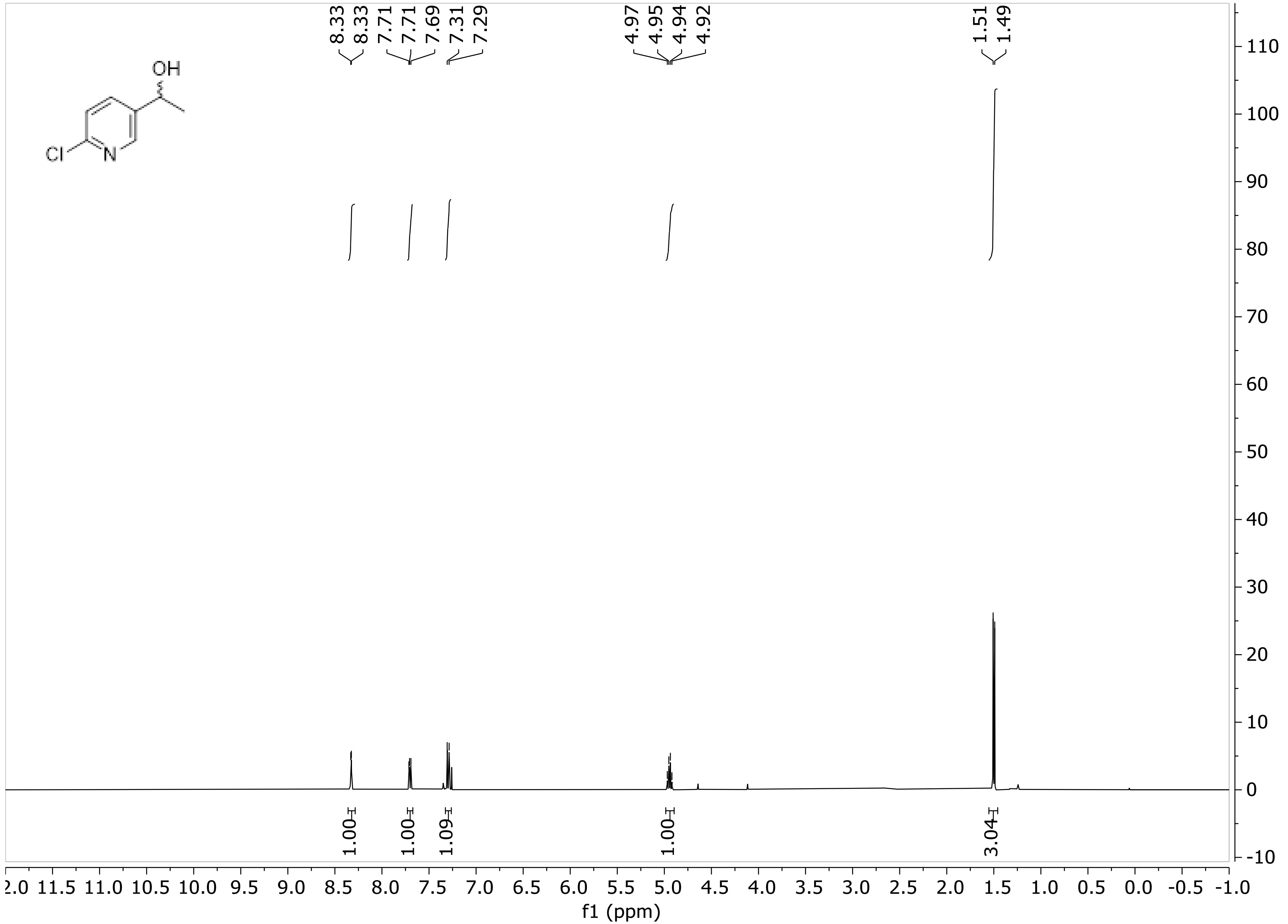

Figure S139. ^{1}H NMR (400 MHz, $CDCl_3$, 298K) of (+)-**72**.

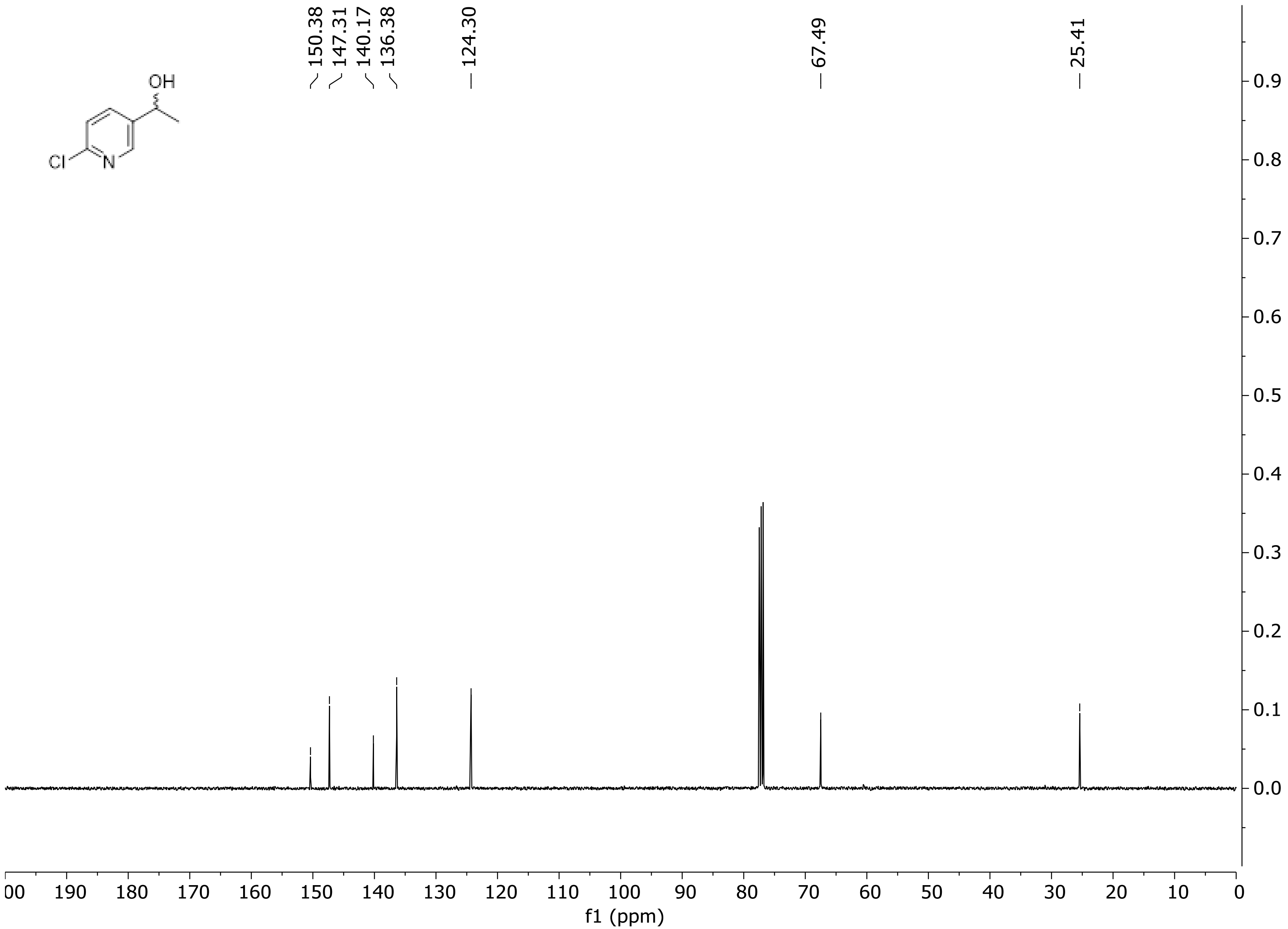

Figure S140. ^{13}C NMR (101 MHz, $CDCl_3$, 298K) of (+)-**72**.

*(+)-5-(1-Hydroxyethyl)-picolinonitrile (R)-***73**

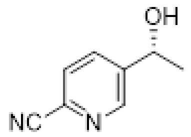

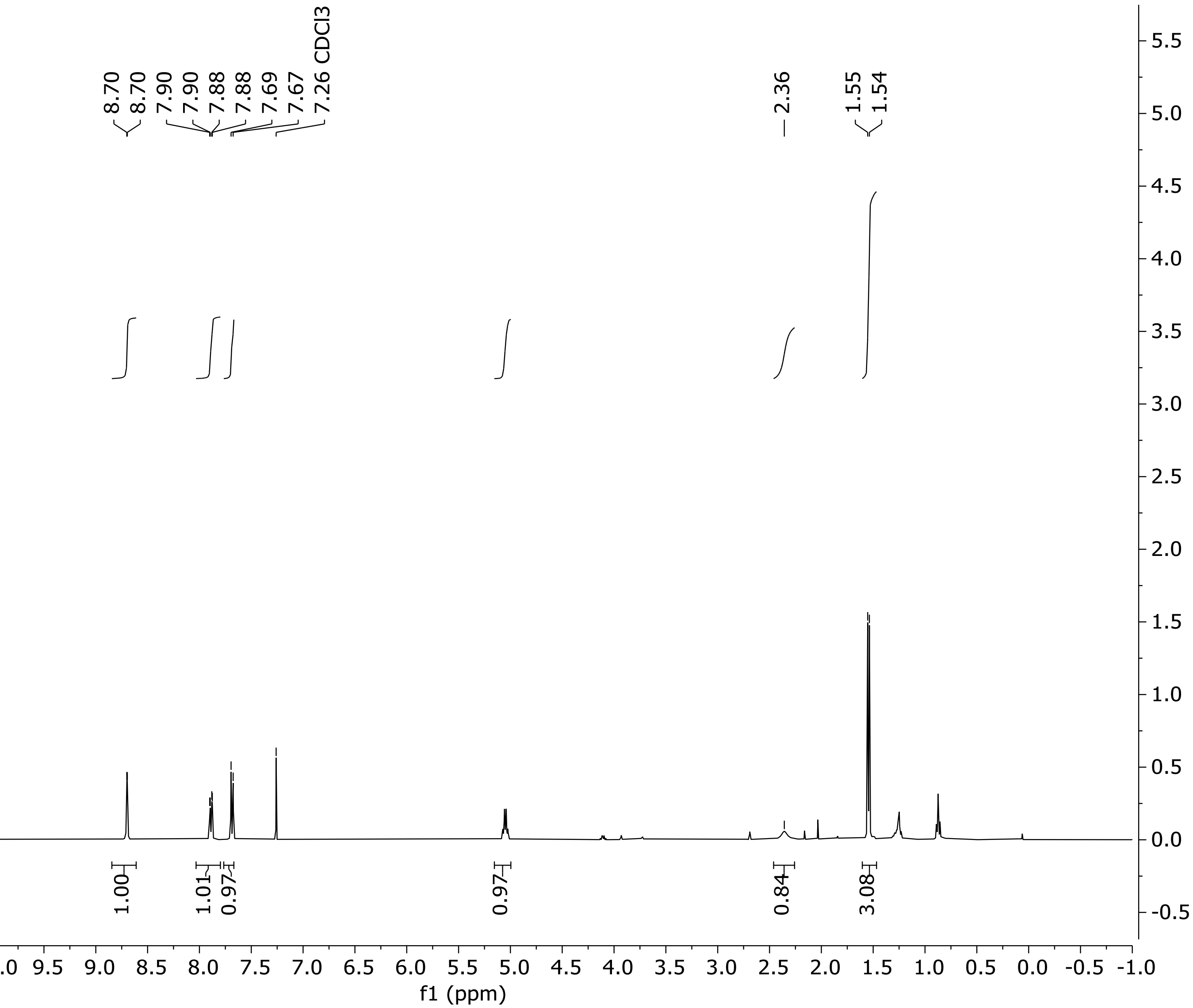

Figure S141. ^{1}H NMR (400 MHz, $CDCl_3$, 298K) of (*R*)-**73**.

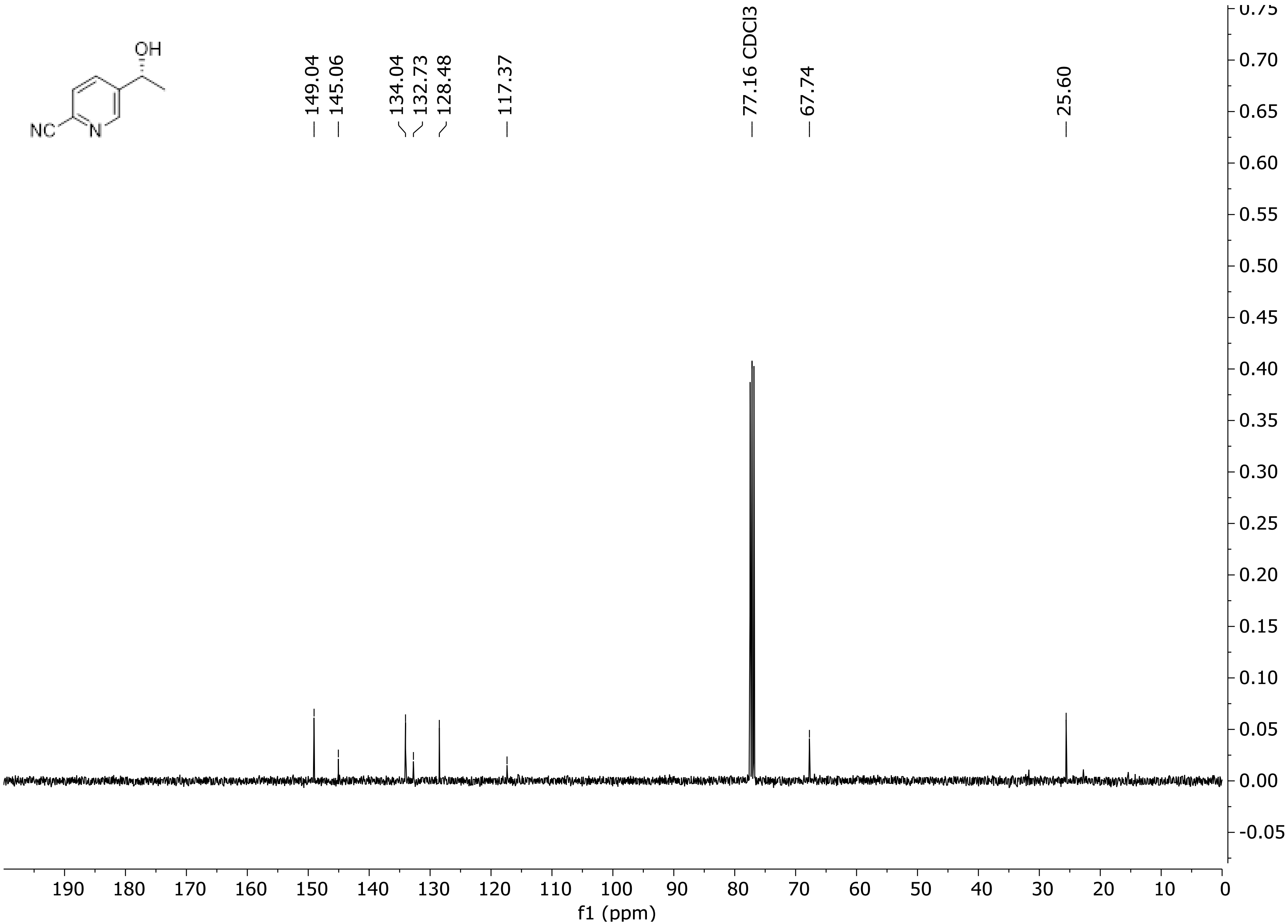

Figure S142. ^{13}C NMR (101 MHz, $CDCl_3$, 298K) of (*R*)-**73**.

(+)-5-(1-Hydroxyethyl)-picolinamide (*R*)-**74**

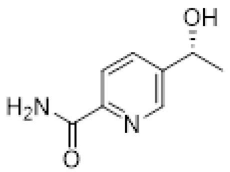

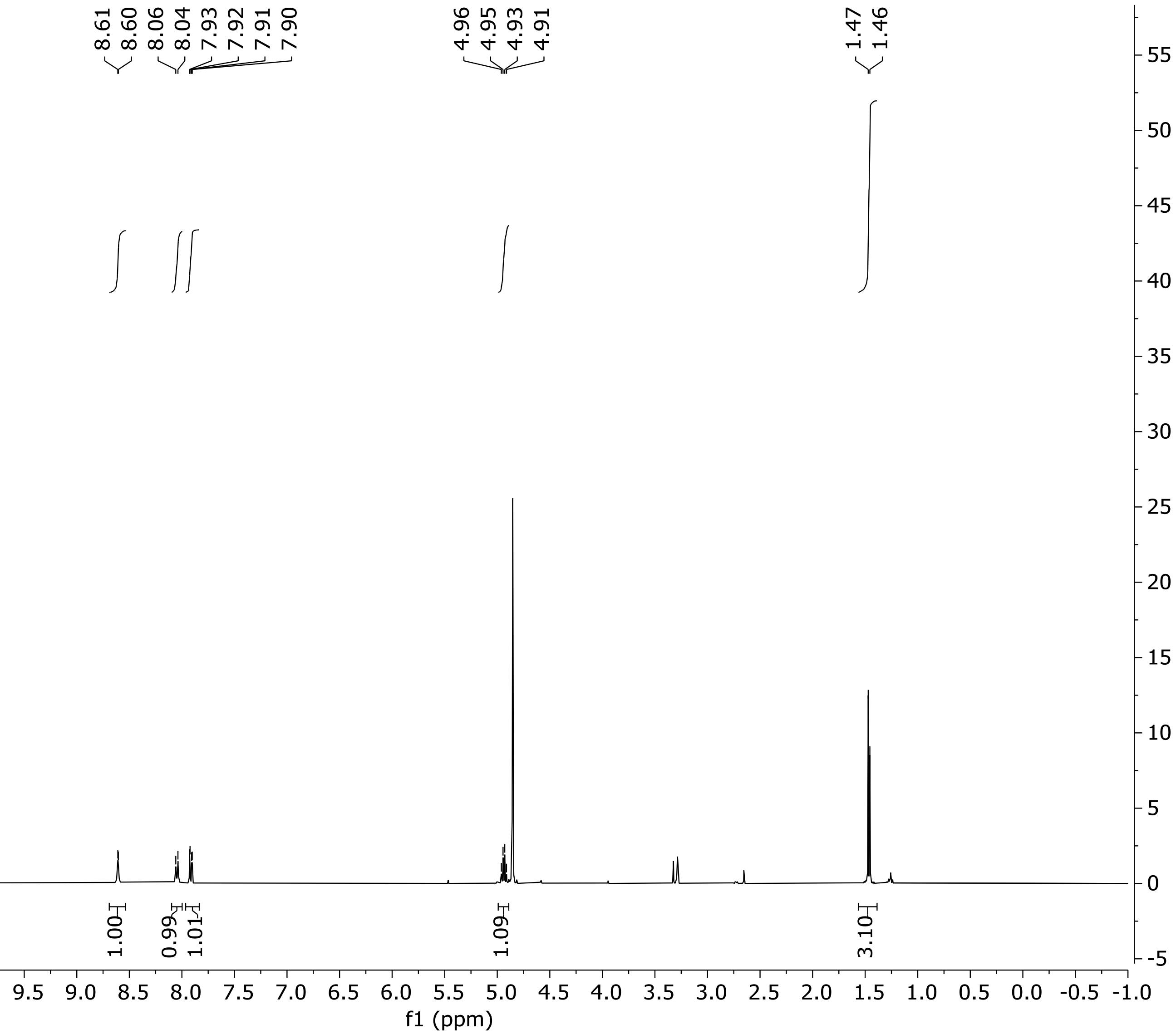

Figure S143. ^{1}H NMR (400 MHz, MeOH-d_4, 298K) of (*R*)-**74**.

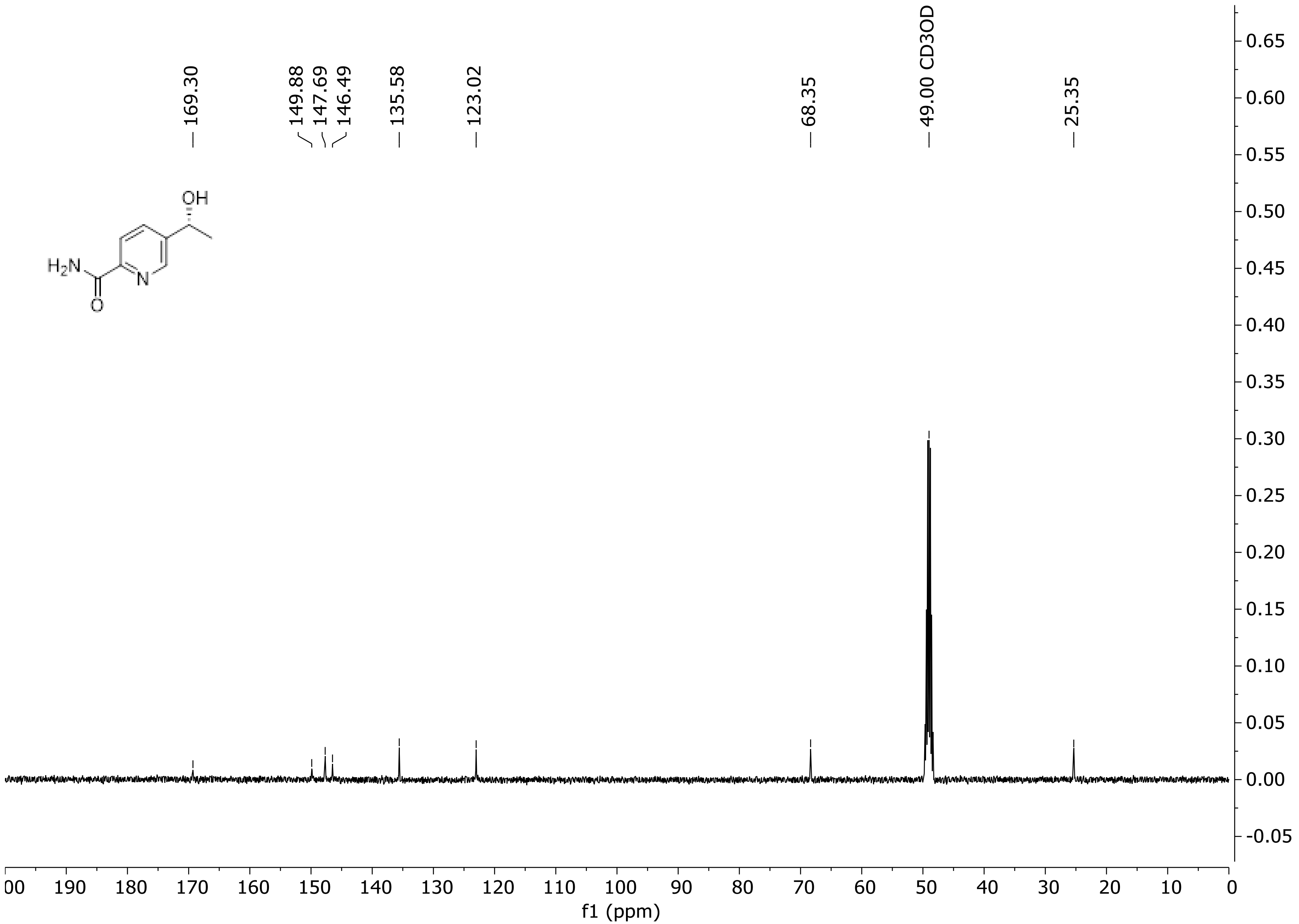

Figure S144. ^{13}C NMR (101 MHz, MeOH-d_4, 298K) of (*R*)-**74**.

*(+)-1-(6-(Methylsulfonyl)pyridin-3-yl)-ethan-1-ol (R)-***75**

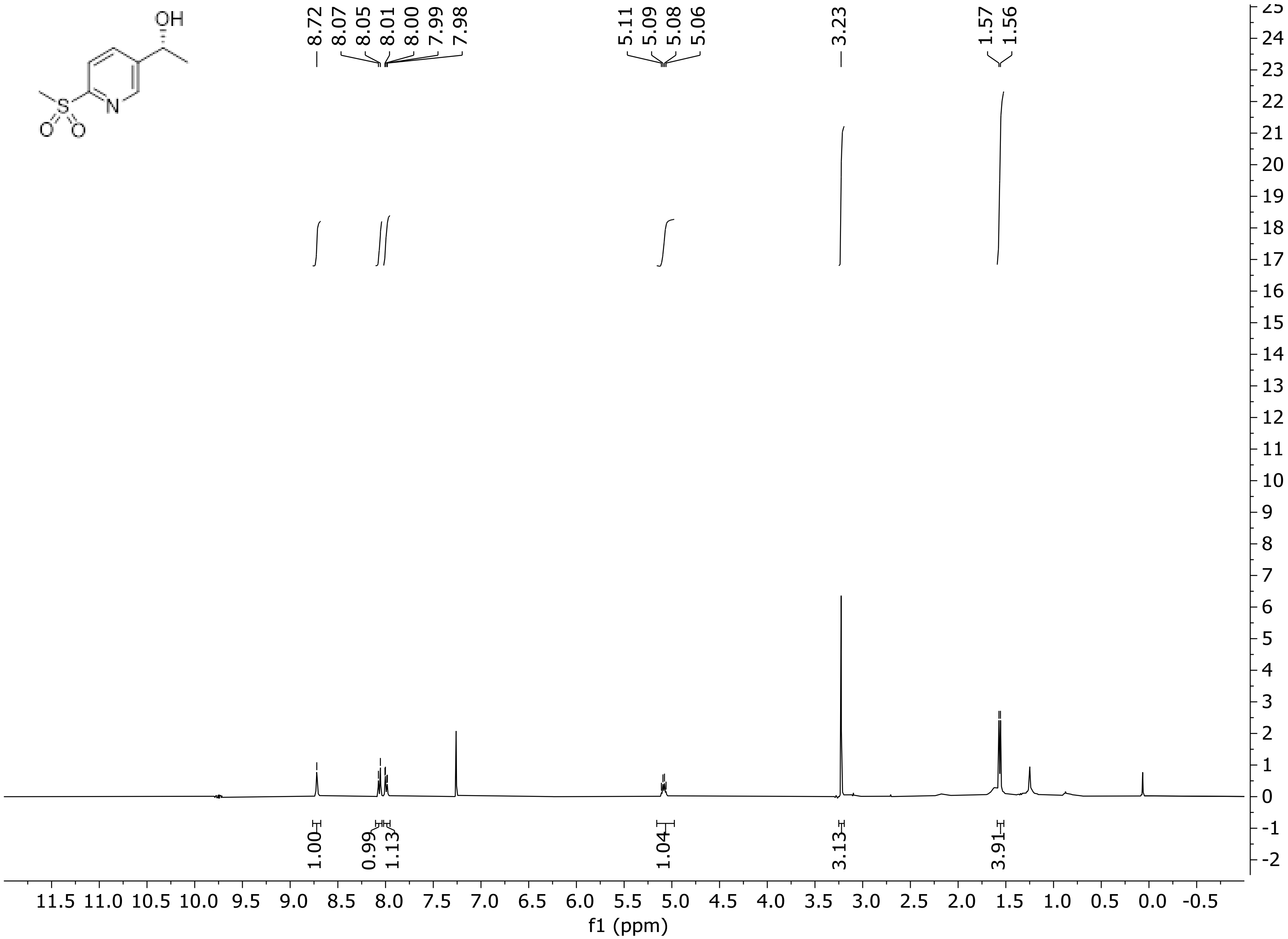

Figure S145. ^{1}H NMR (400 MHz, $CDCl_3$, 298K) of (*R*)-**75**.

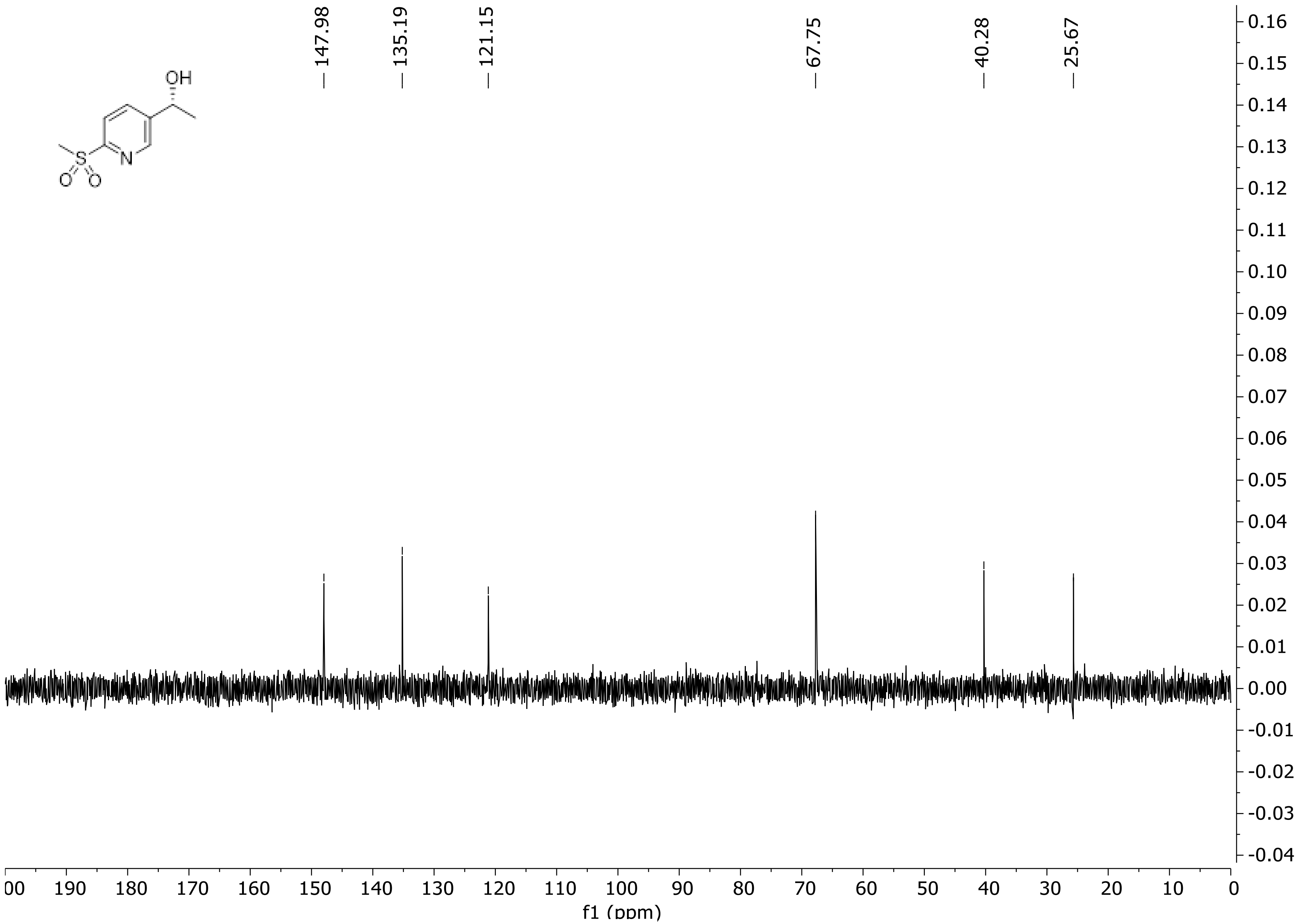

Figure S146. ^{13}C NMR (101 MHz, $CDCl_3$, 298K) of (*R*)-**75**.

5,6,7,8-Tetrahydroquinolin-6-ol **76**

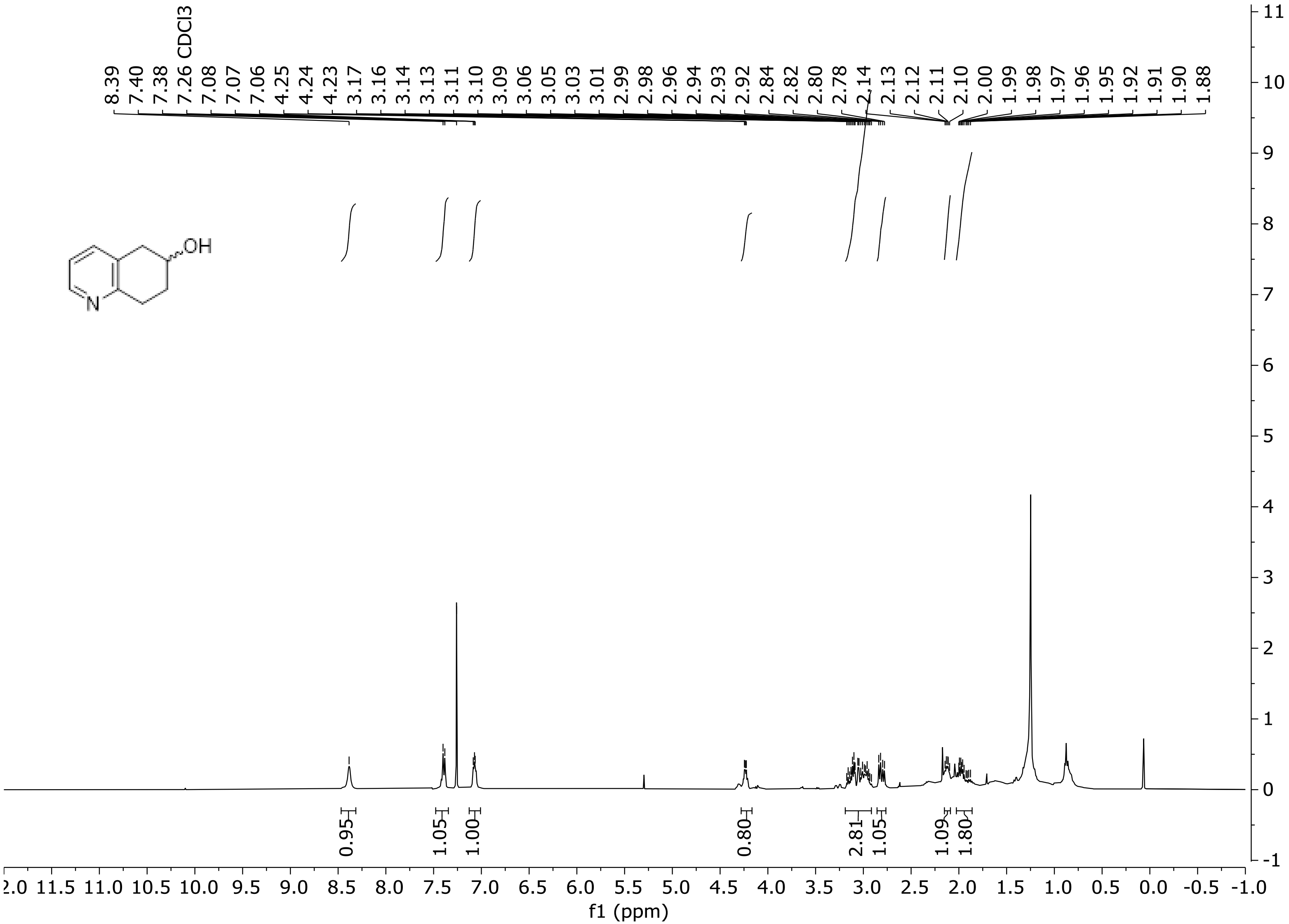

Figure S147. ^{1}H NMR (400 MHz, $CDCl_3$, 298K) of **76**.

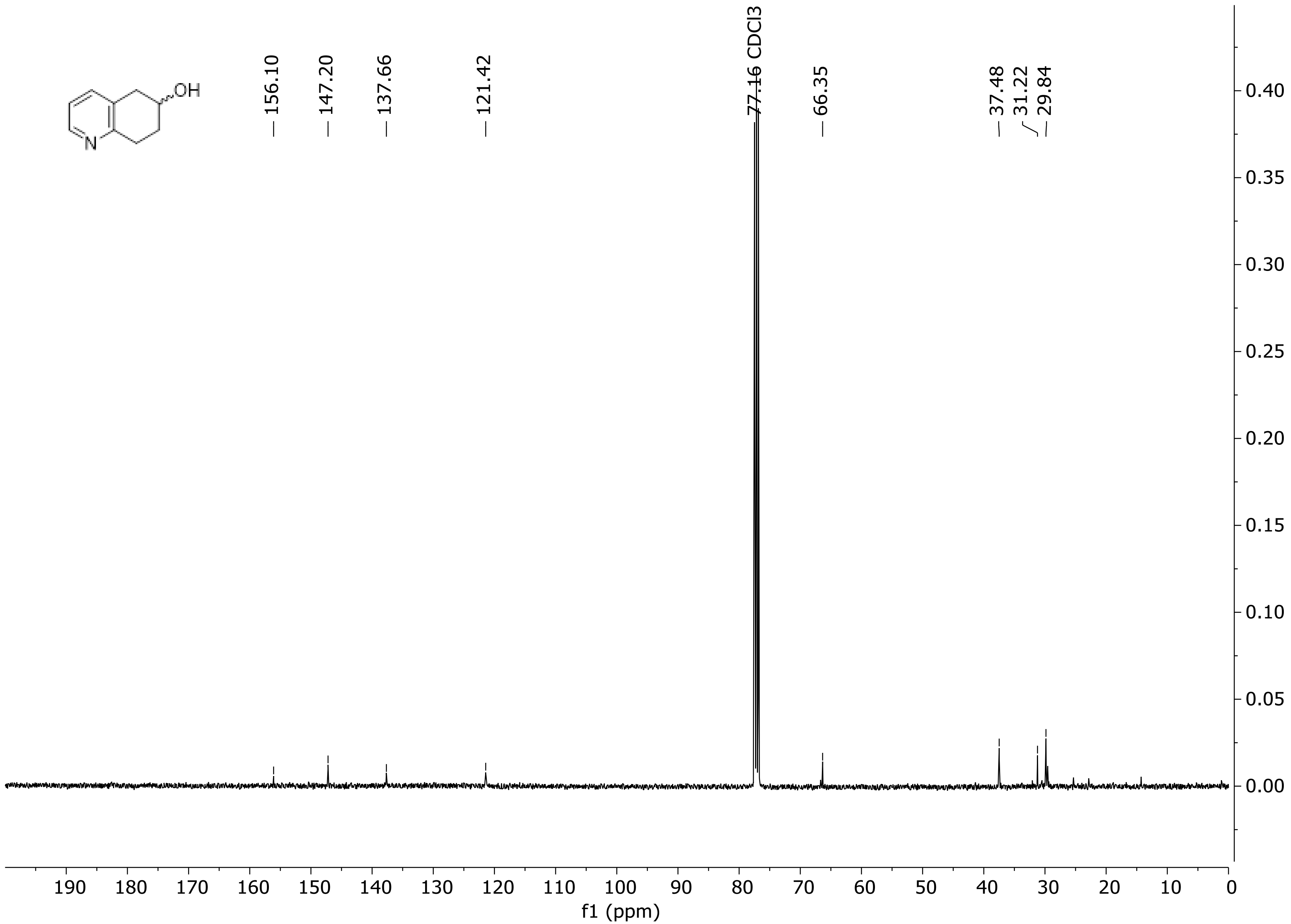

Figure S148. ^{13}C NMR (101 MHz, $CDCl_3$, 298K) of **76**.

Mixture of *5,6,7,8-tetrahydroquinolin-7-ol* **77** (major isomer) and *5,6,7,8-Tetrahydroquinolin-6-ol* **76** (minor isomer)

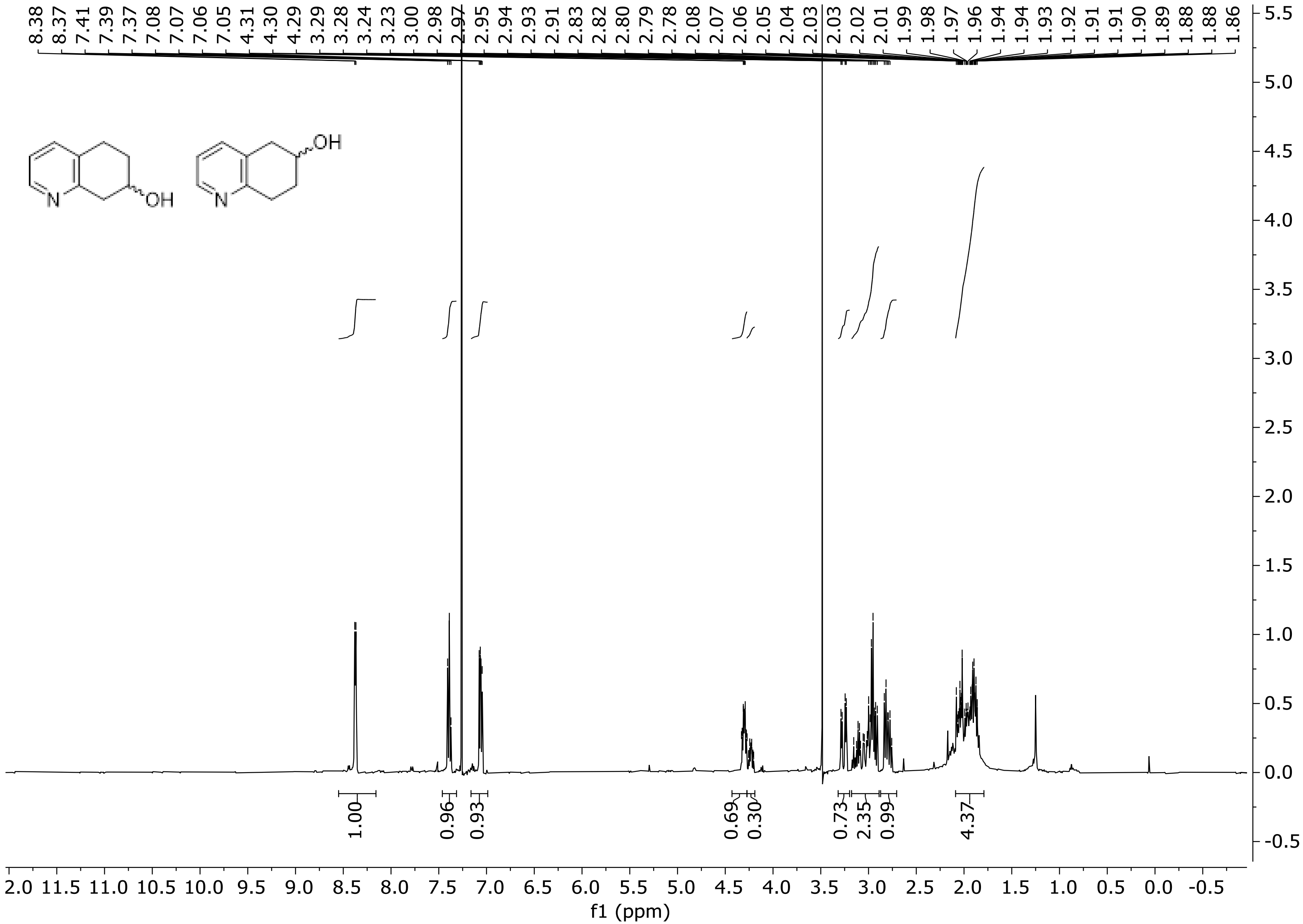

Figure S149. ^{1}H NMR (400 MHz, $CDCl_3$, 298K) of **77**.

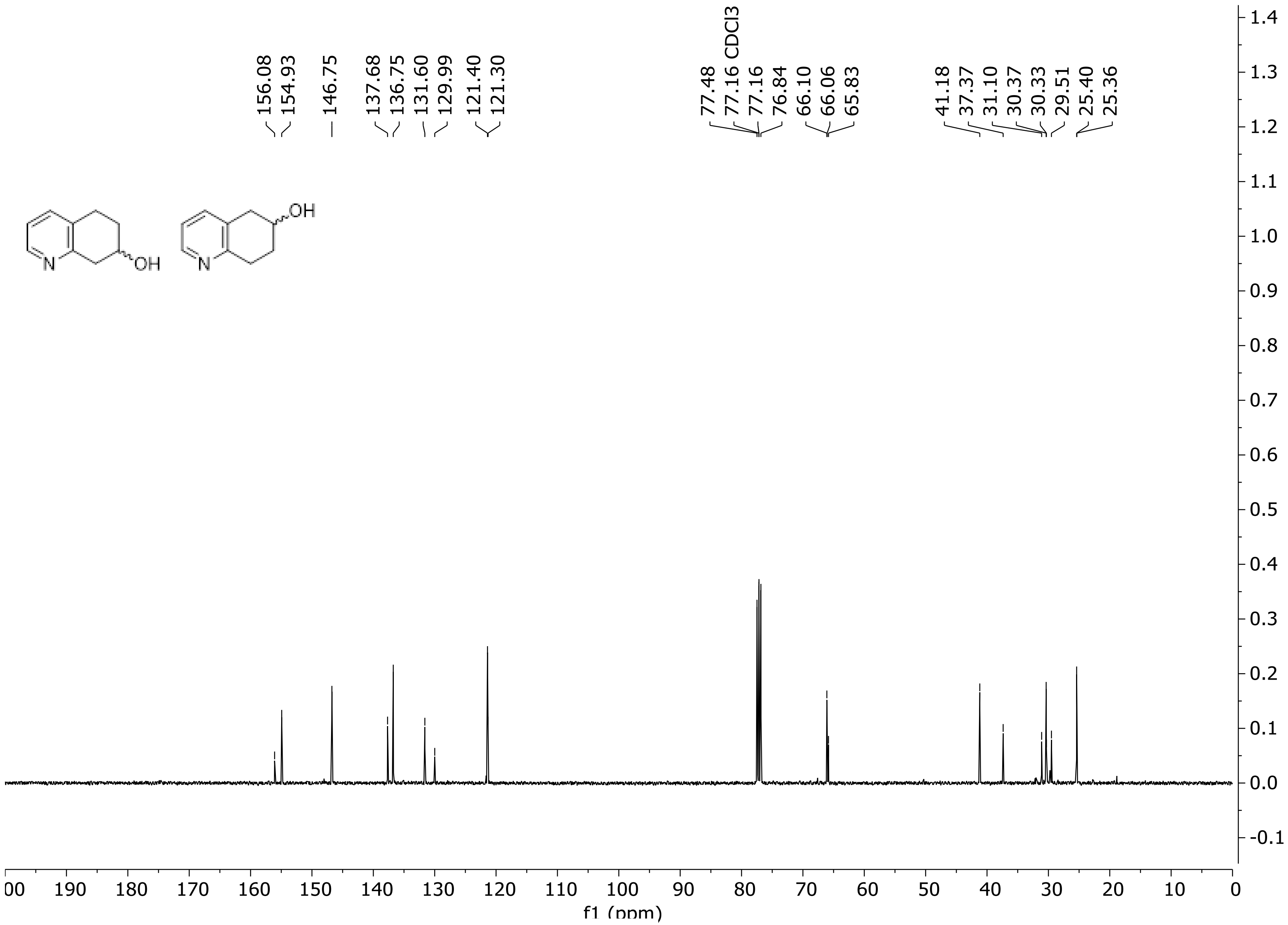

Figure S150. ^{13}C NMR (101 MHz, $CDCl_3$, 298K) of **77**.

Mixture of *5,6,7,8-Tetrahydroisoquinolin-5-ol* **37a** (major isomer) and *5,6,7,8-Tetrahydroquinolin-7-ol* **37c** (minor isomer)

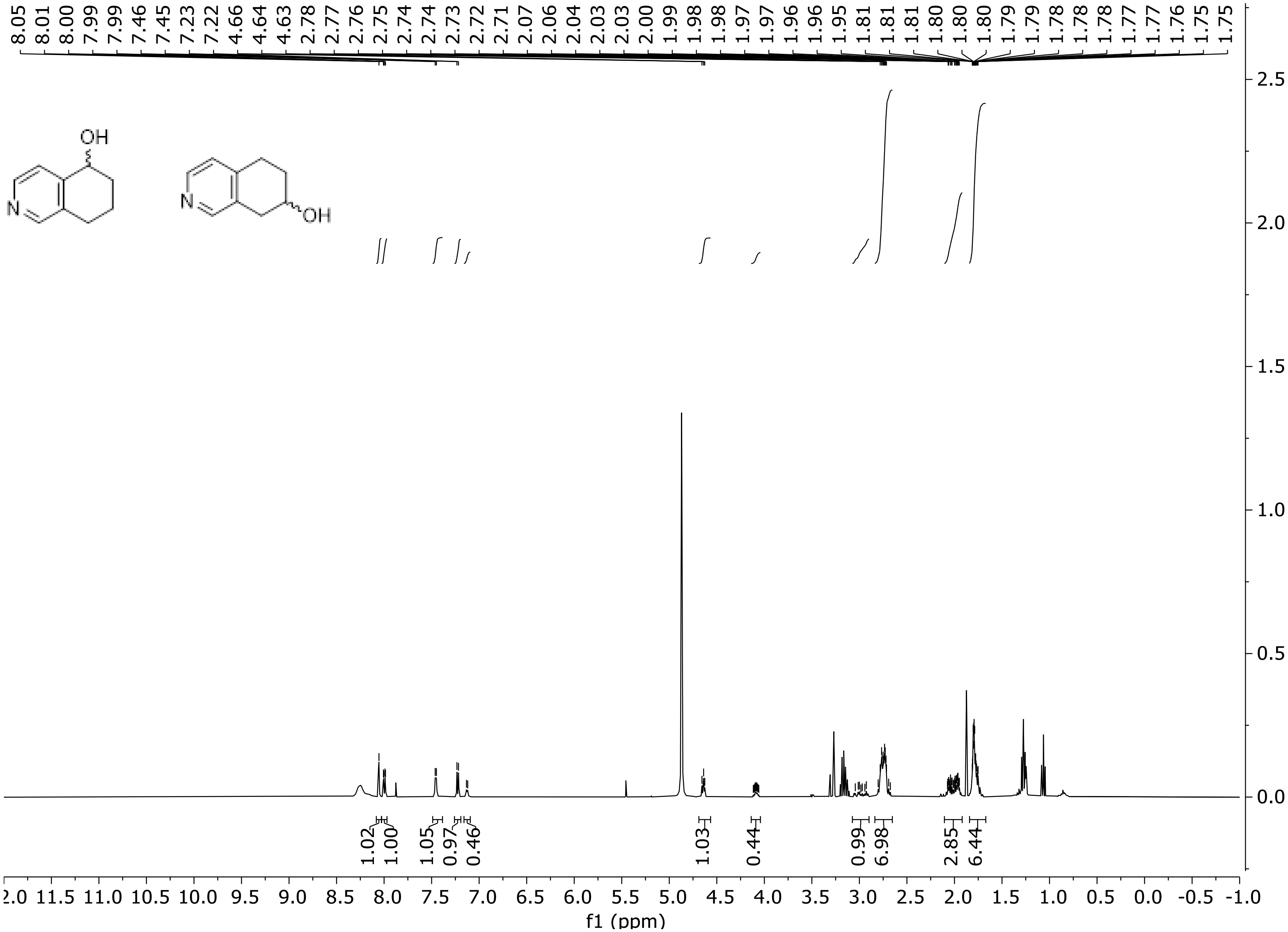

Figure S151. ^{1}H NMR (400 MHz, MeOH-d_4, 298K) of **37a**.and **37c**

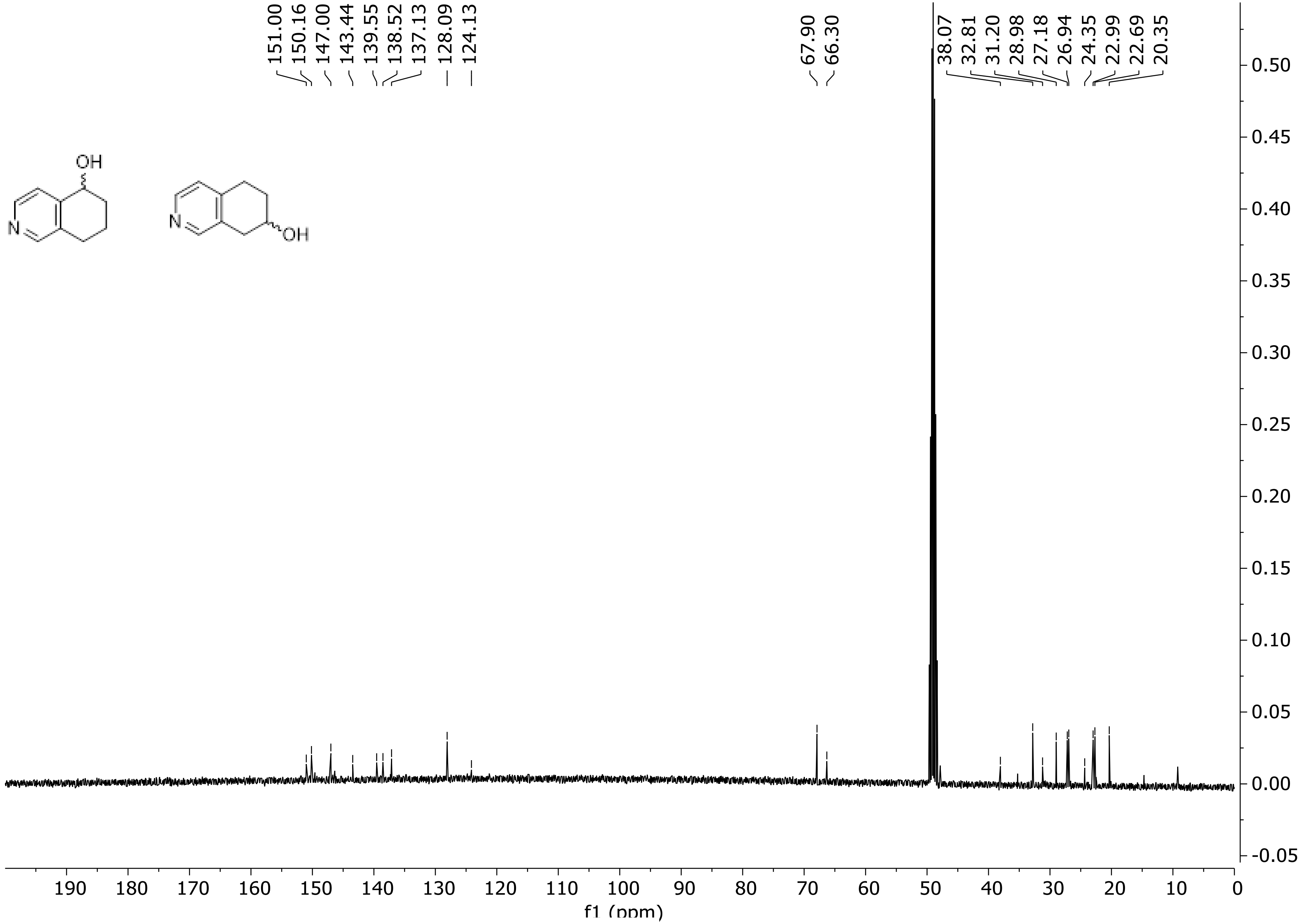

Figure S152. ^{13}C NMR (101 MHz, MeOH-d_4, 298K) of **37a**.and **37c**

Mixture of *5,6,7,8-Tetrahydroquinoline-8-ol* **37d** (major isomer) and *5,6,7,8-Terahydroquinolin-6-ol* **37b** (minor isomer)

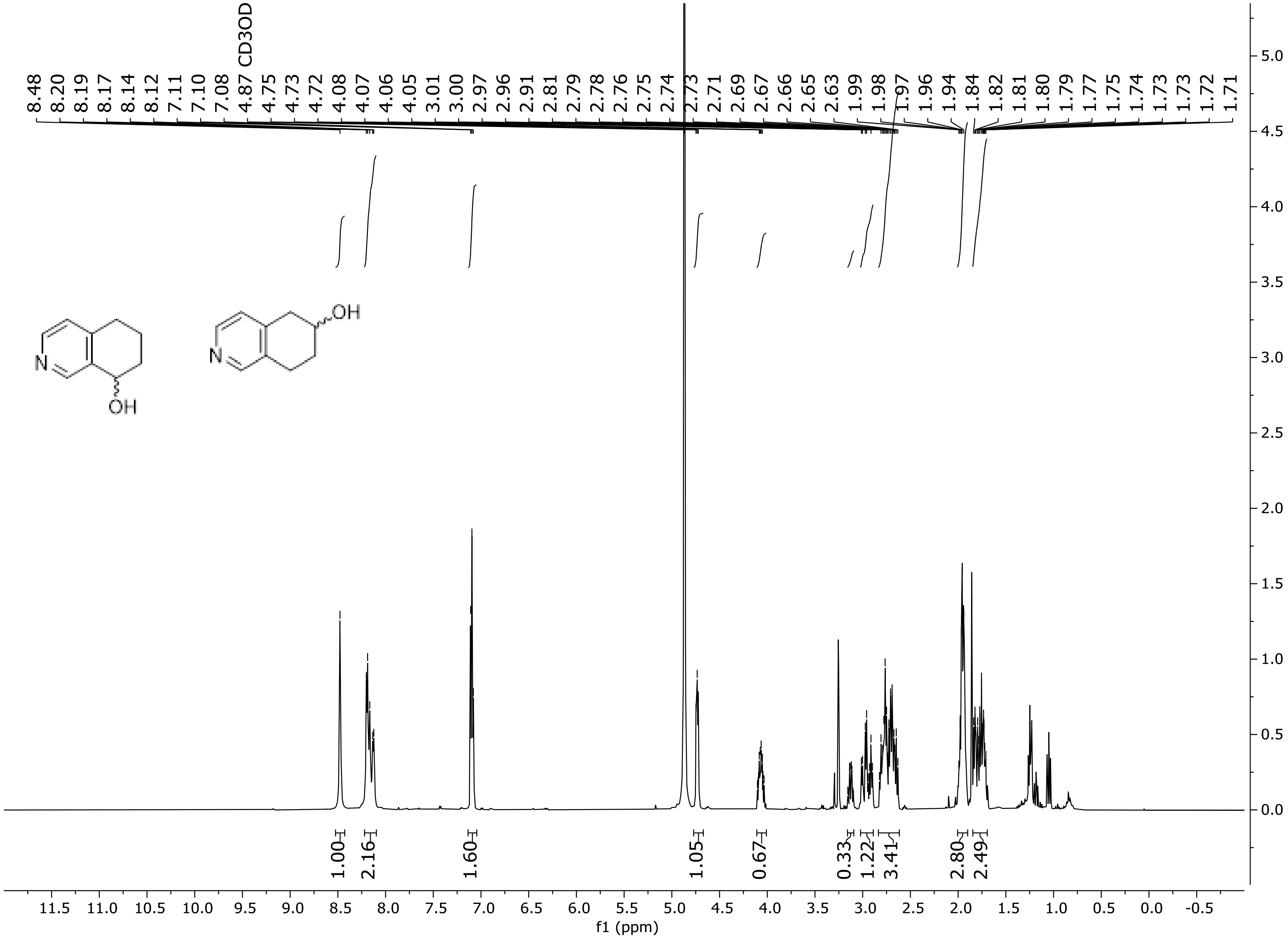

Figure S153. ^{1}H NMR (400 MHz, MeOH-d_4, 298K) of **37d**.and **37b**

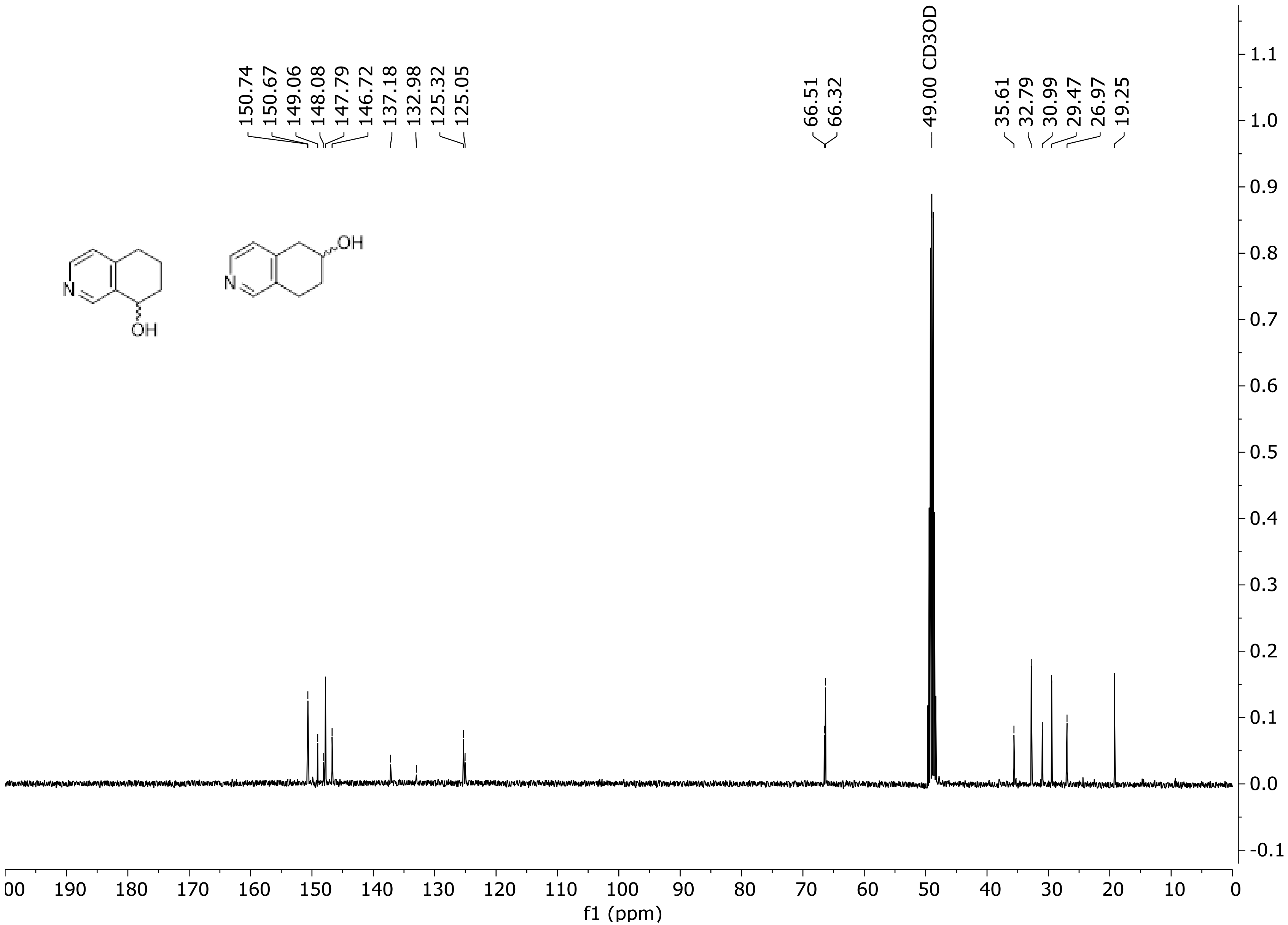

Figure S154. ^{13}C NMR (101 MHz, MeOH-d_4, 298K) of **37d**.and **37b**

1a,7b-Dihydrooxireno[2,3-f]-quinoline **41**

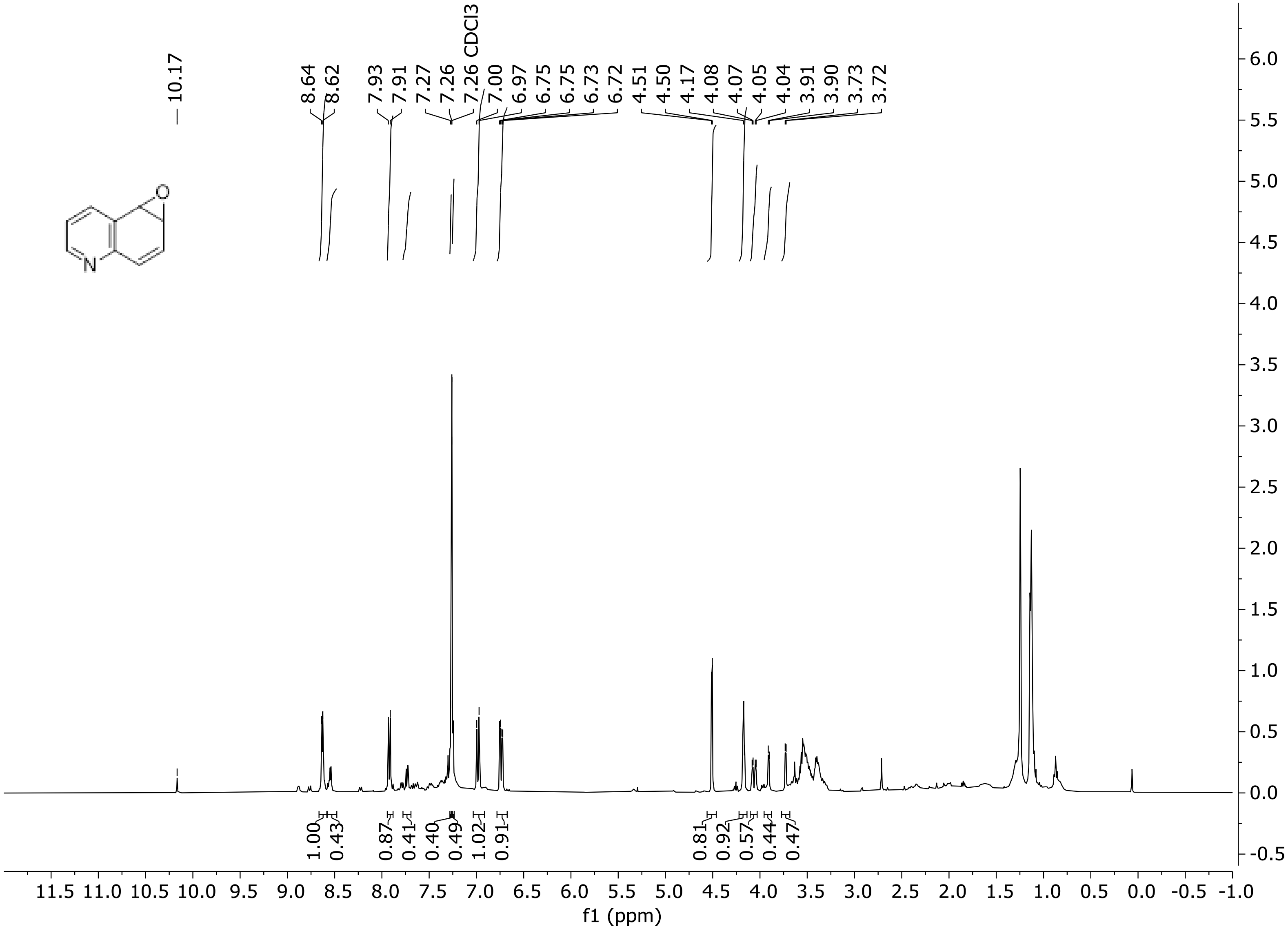

Figure S155. ^{1}H NMR (400 MHz, $CDCl_3$, 298K) of **41**.

1a,2,3,7b-Tetrahydrooxireno[2,3-h]-quinoline-2,3-diol **43**

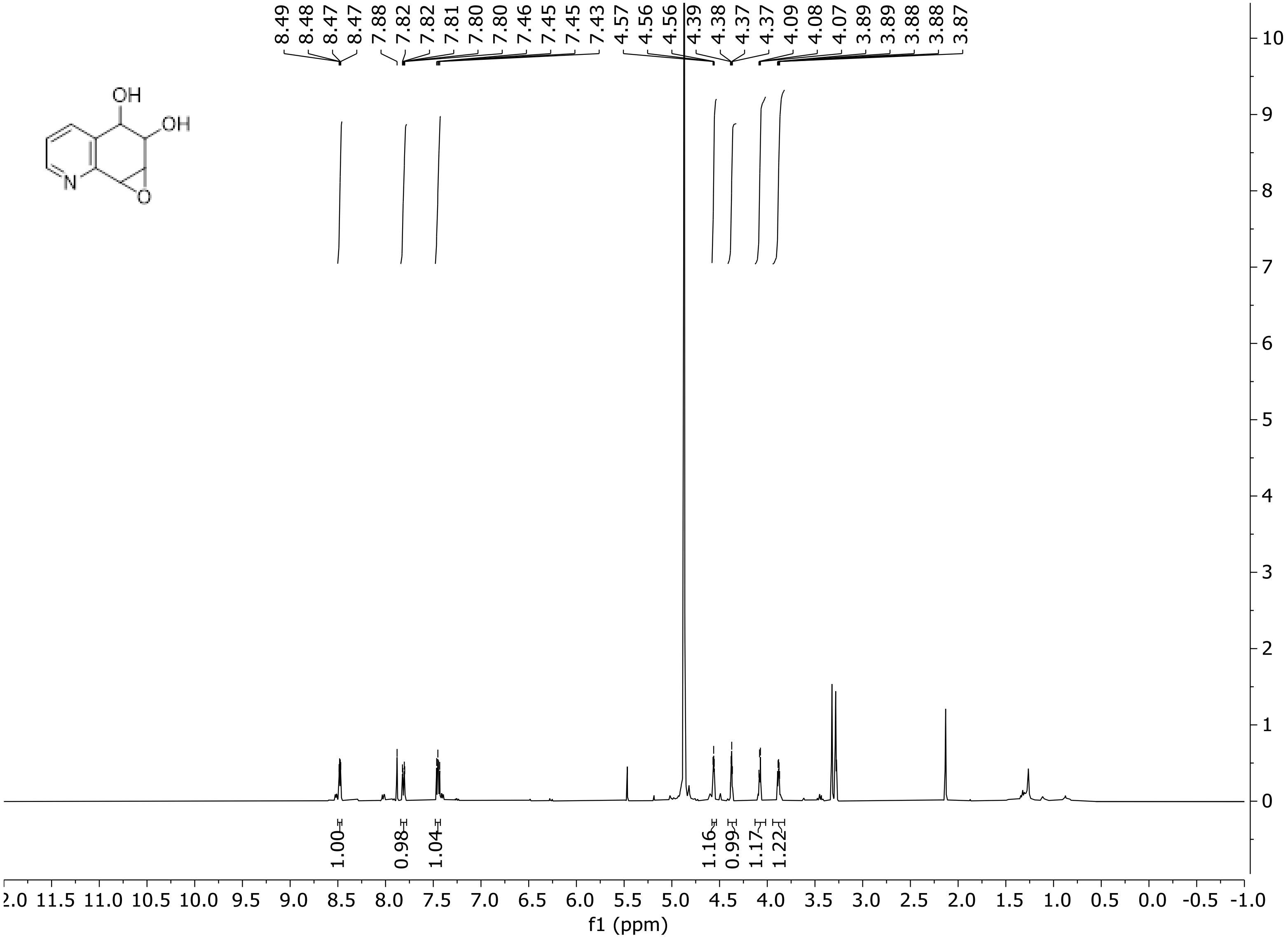

Figure S156. ^{1}H NMR (400 MHz, MeOH-d_4, 298K) of **43**.

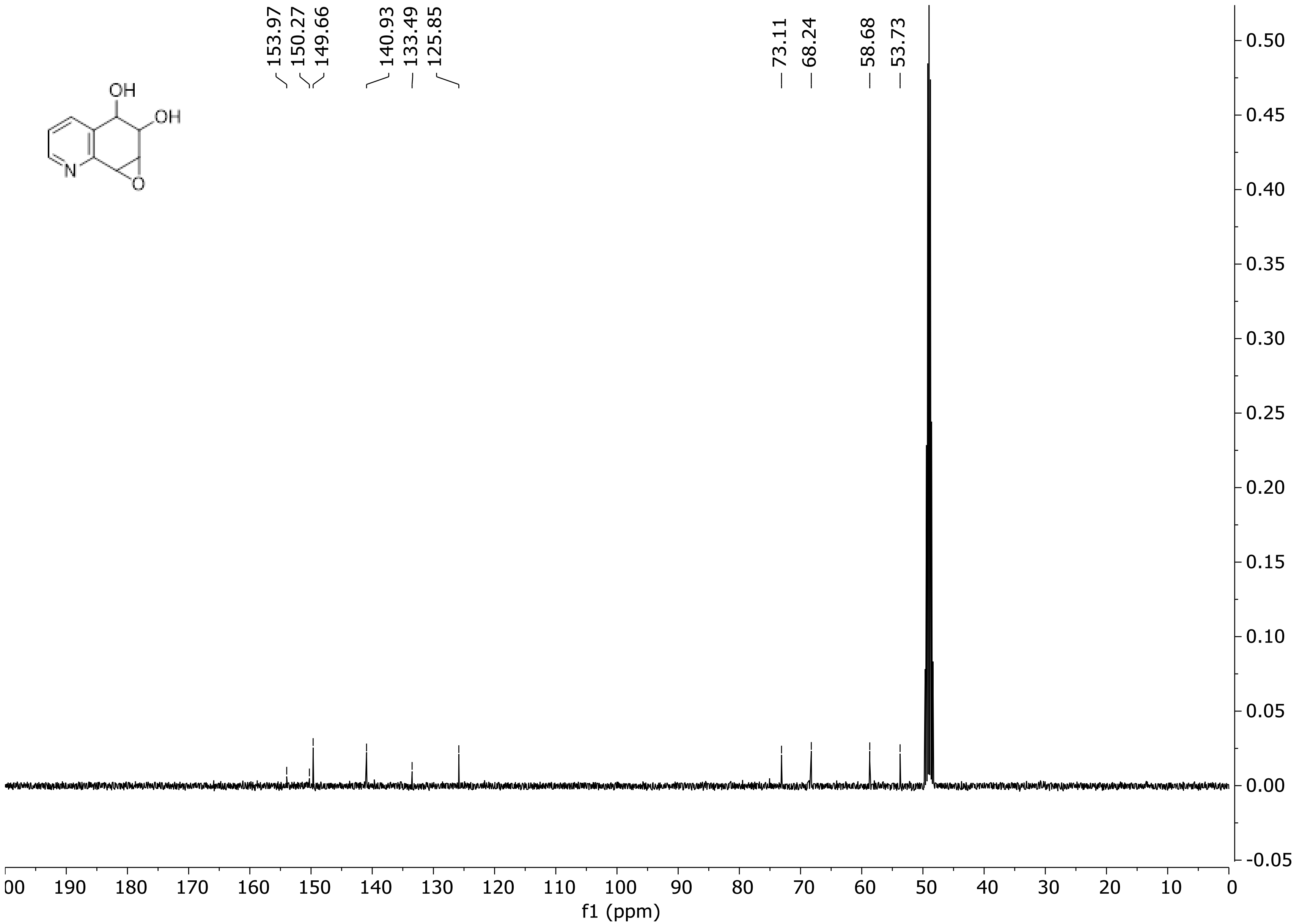

Figure S157. ^{13}C NMR (101 MHz, MeOH-d_4, 298K) of **43**.

Isoquinoline-N-oxide **44**

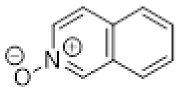

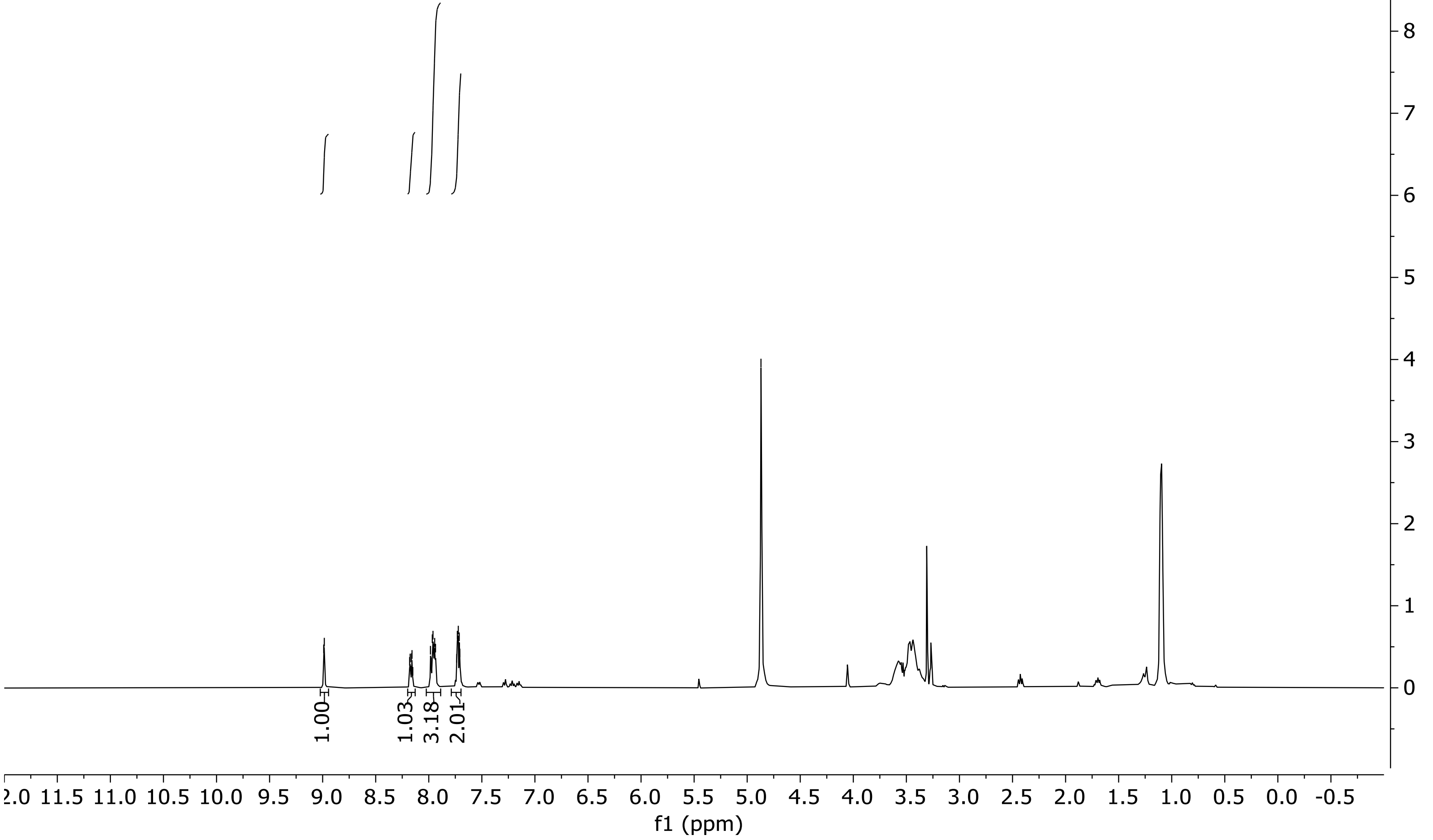

Figure S158. ^{1}H NMR (400 MHz, MeOH-d_4, 298K) of **44**.

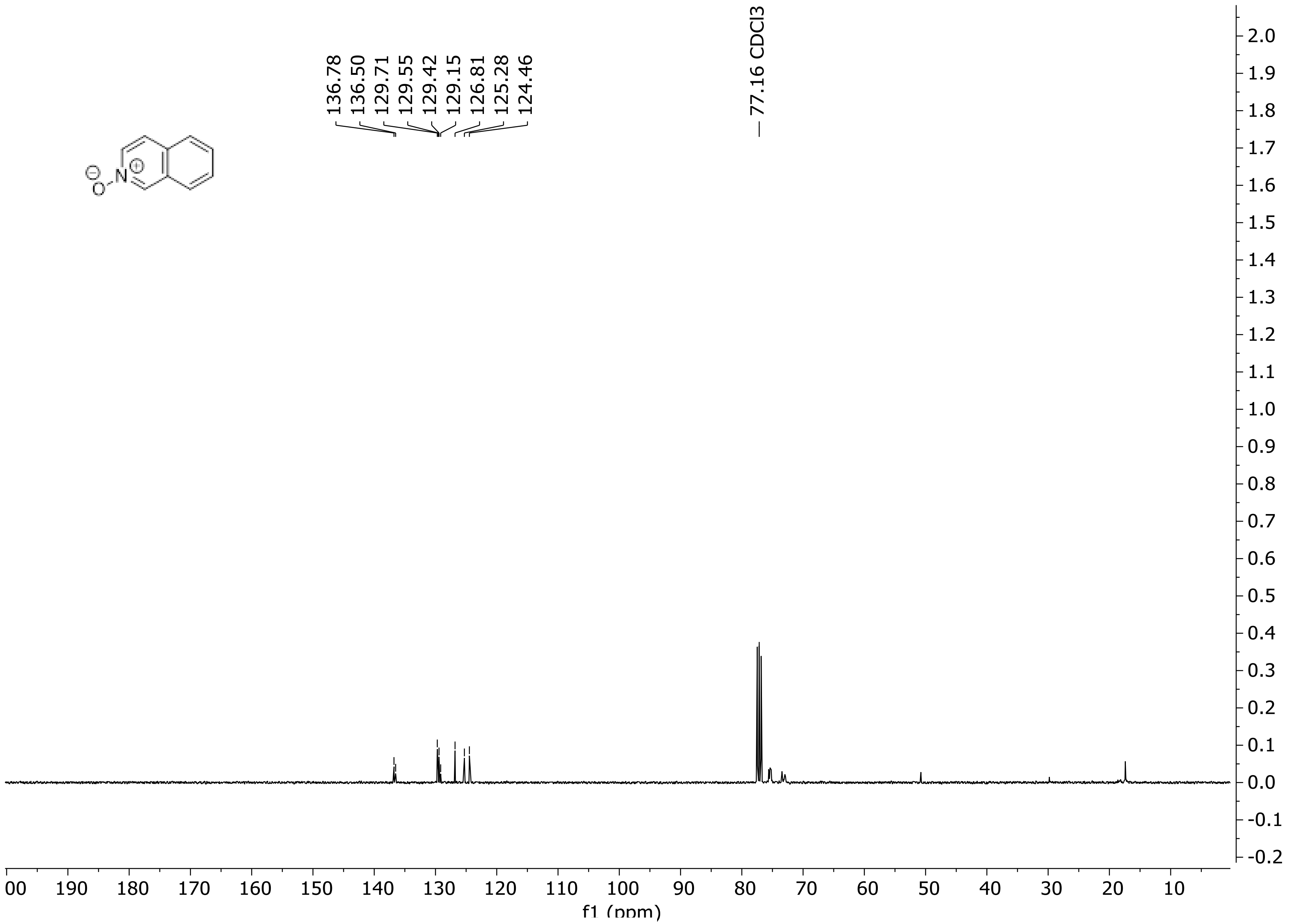

Figure S159. ^{1}C NMR (101 MHz, $CDCl_3$, 298K) of **44**.

1a,7b-Dihydrooxireno[2,3-h]-isoquinoline **78**

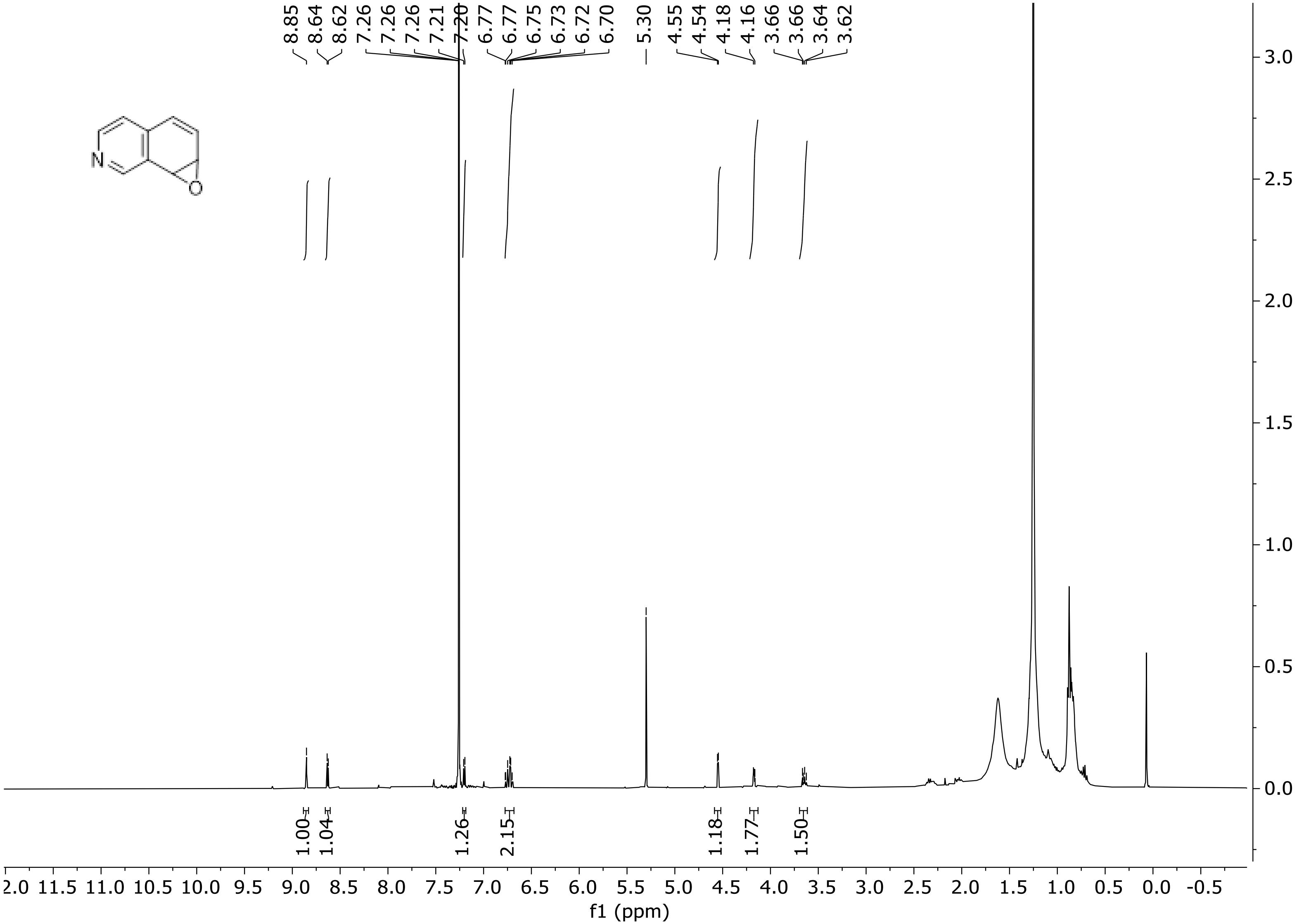

Figure S160. ^{1}H NMR (400 MHz, $CDCl_3$, 298K) of **78**.

3-Methylisoquinoline-N-oxide **46**

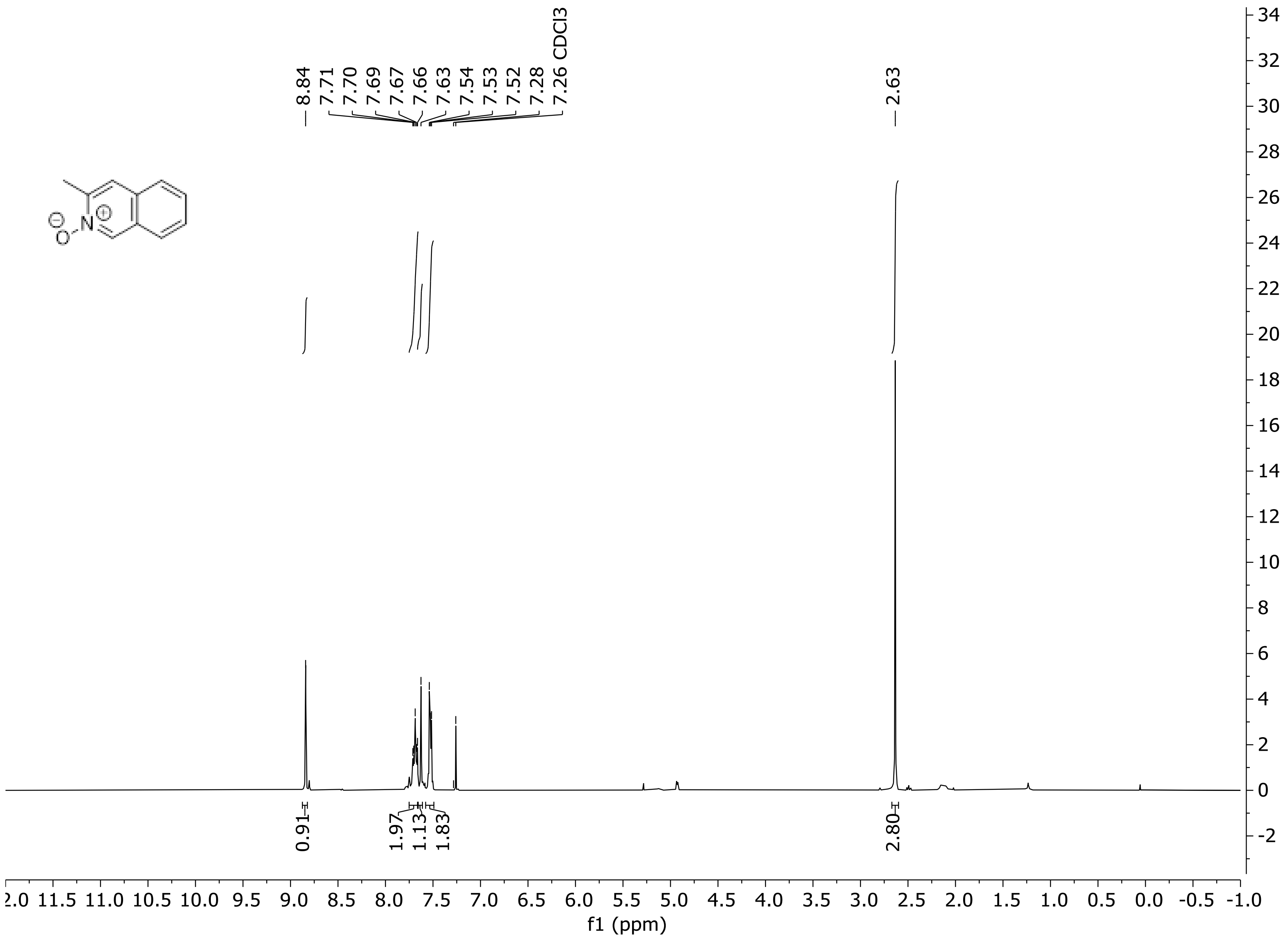

Figure S161. ^{1}H NMR (400 MHz, $CDCl_3$, 298K) of **46**.

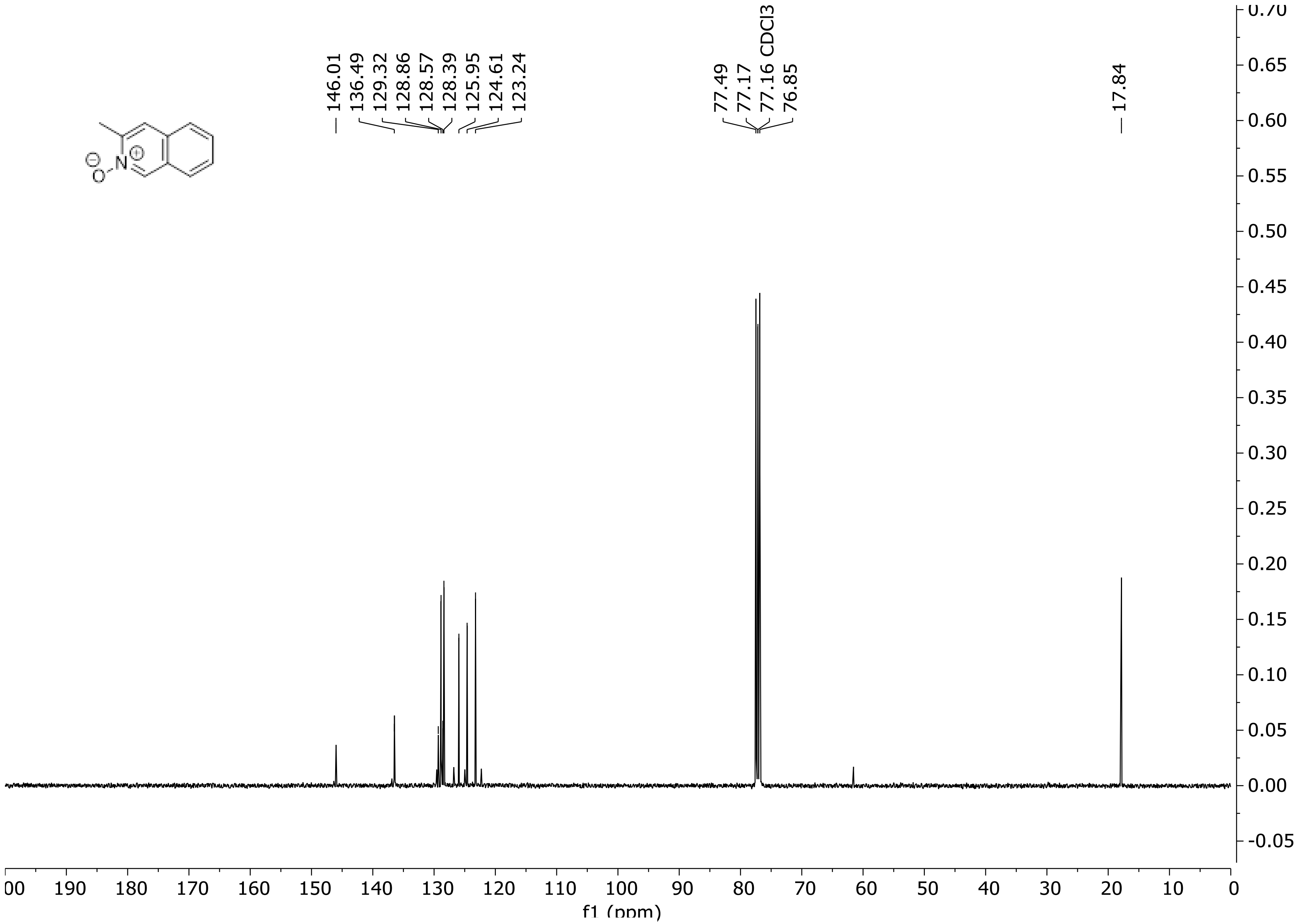

Figure S162. ^{13}C NMR (101 MHz, $CDCl_3$, 298K) of **46**.

Isoquinoline-3-carbaldehyde **47**

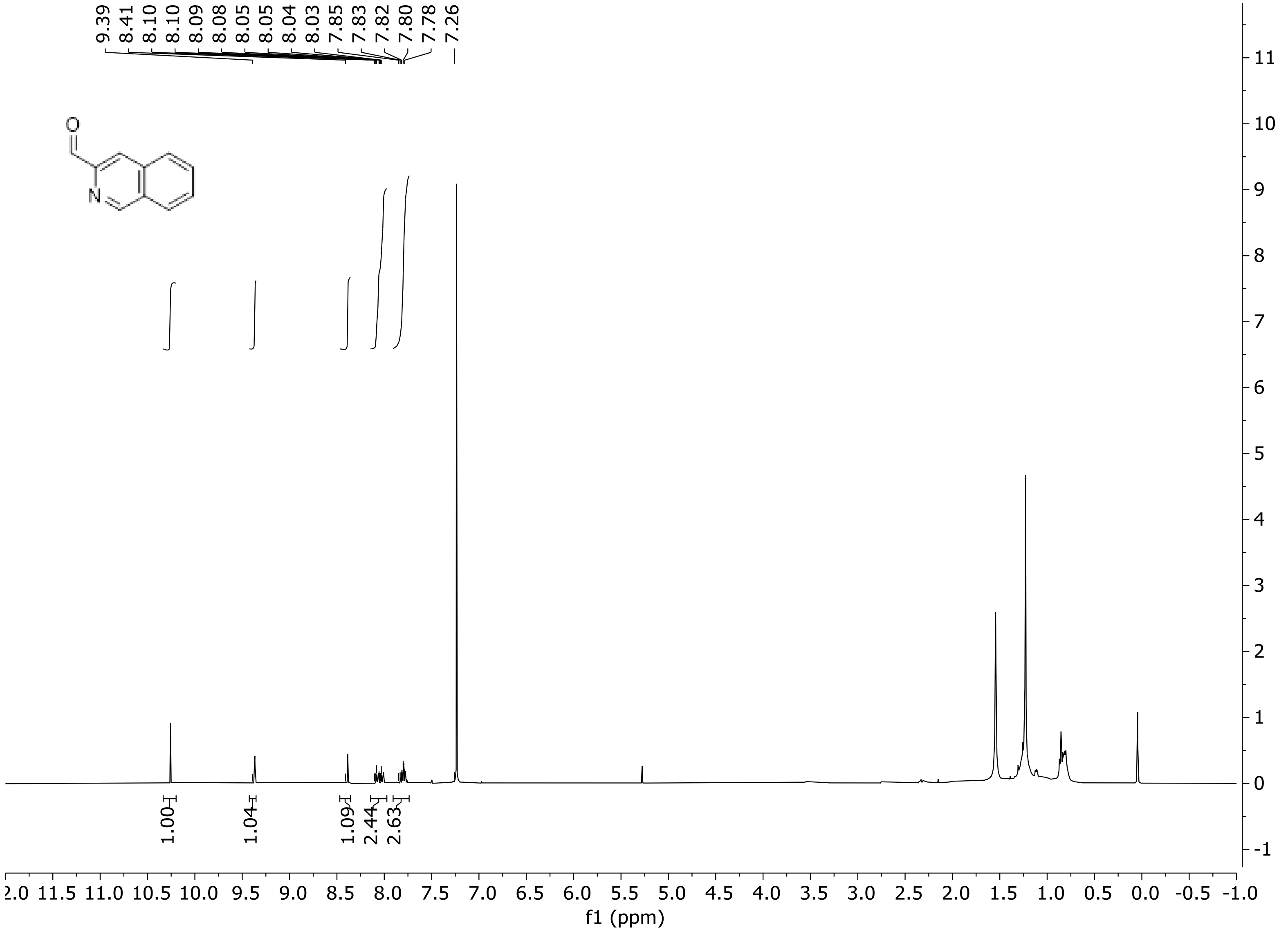

Figure S163. ^{1}H NMR (400 MHz, $CDCl_3$, 298K) of **47**.

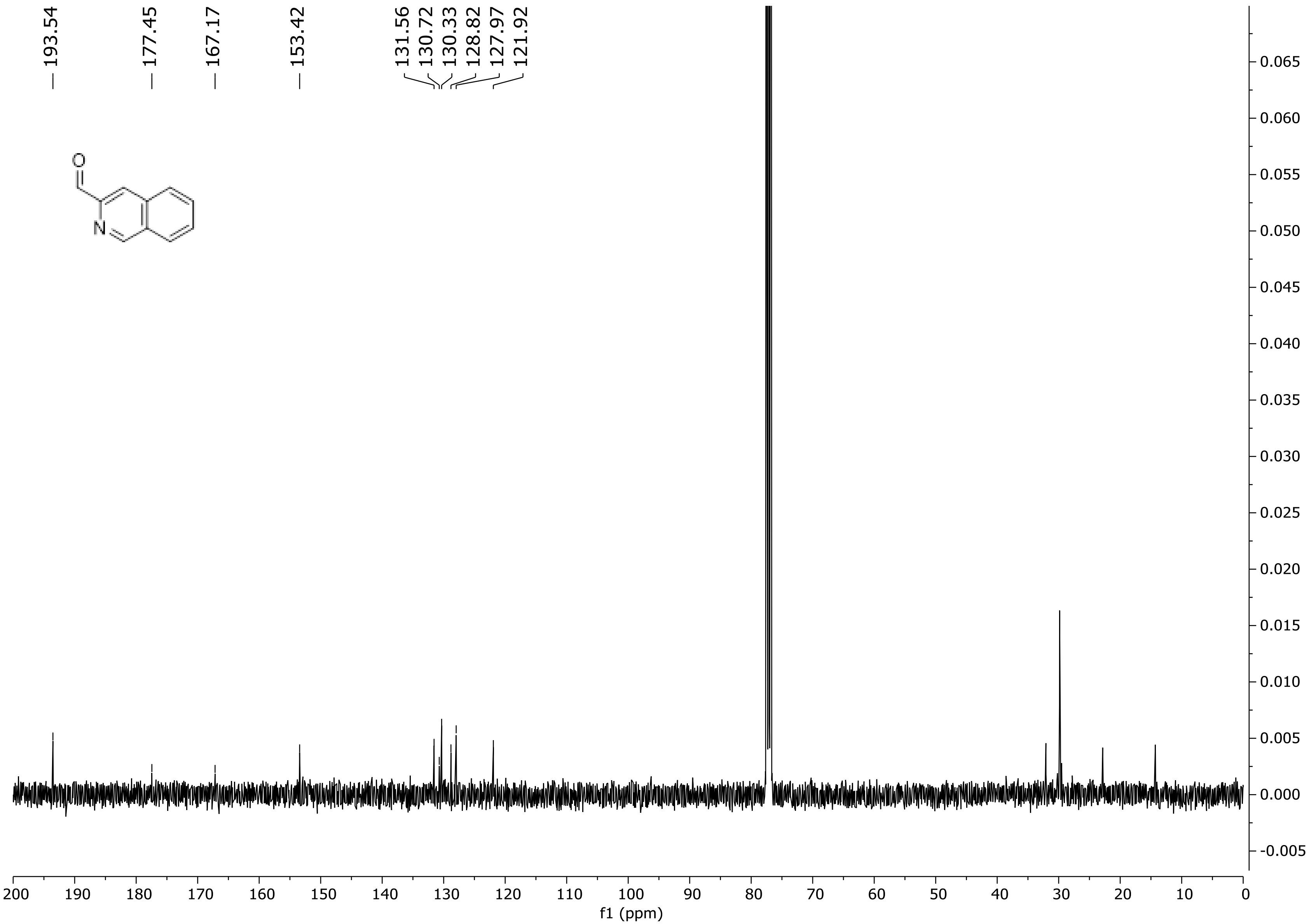

Figure S164. ^{13}C NMR (101 MHz, $CDCl_3$, 298K) of **47**.

(-)-2-Bromo-5,6,7,8-tetrahydroquinolin-5-ol **80a**

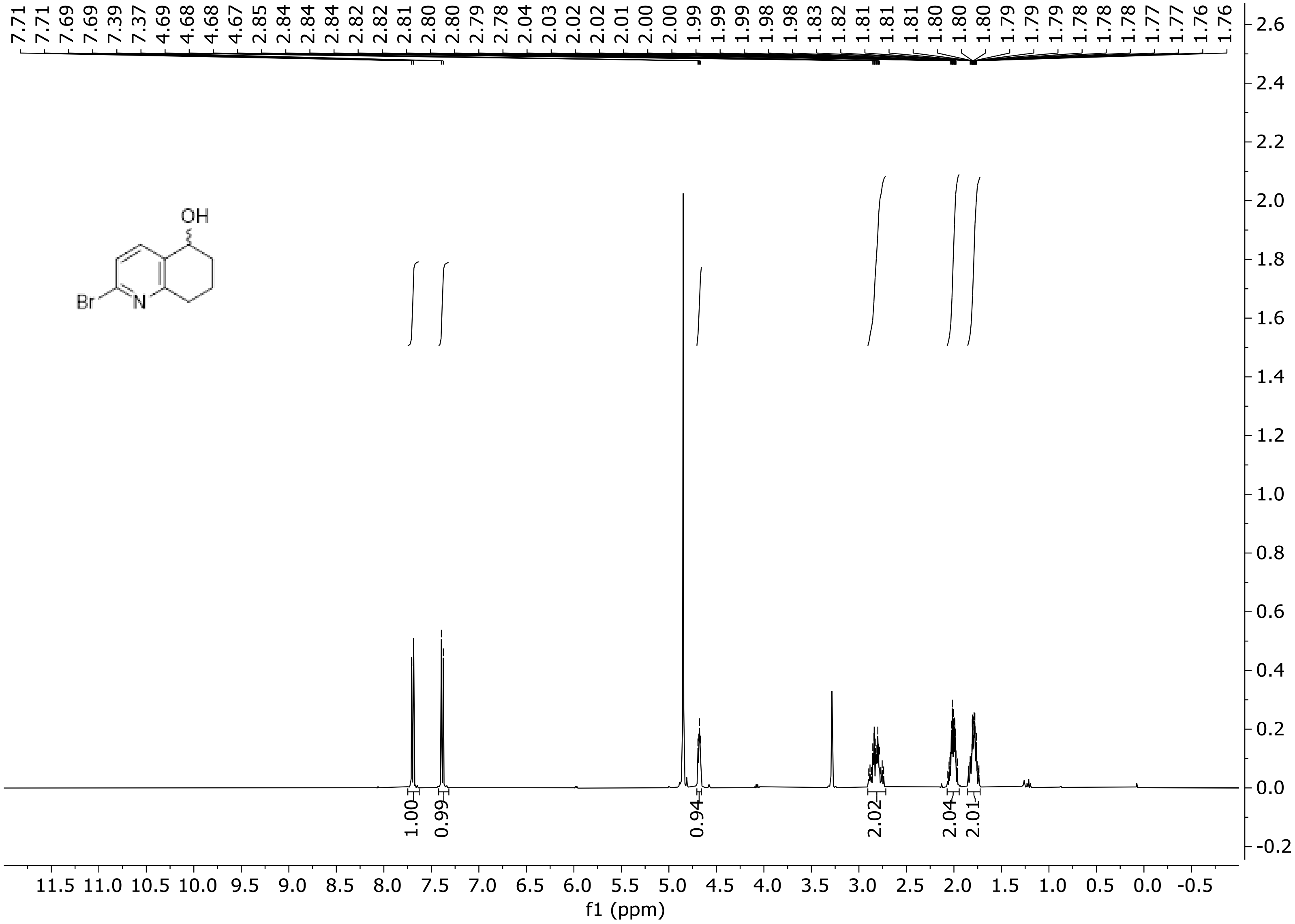

Figure S165. ^{1}H NMR (400 MHz, MeOH-d_4, 298K) of **80a**.

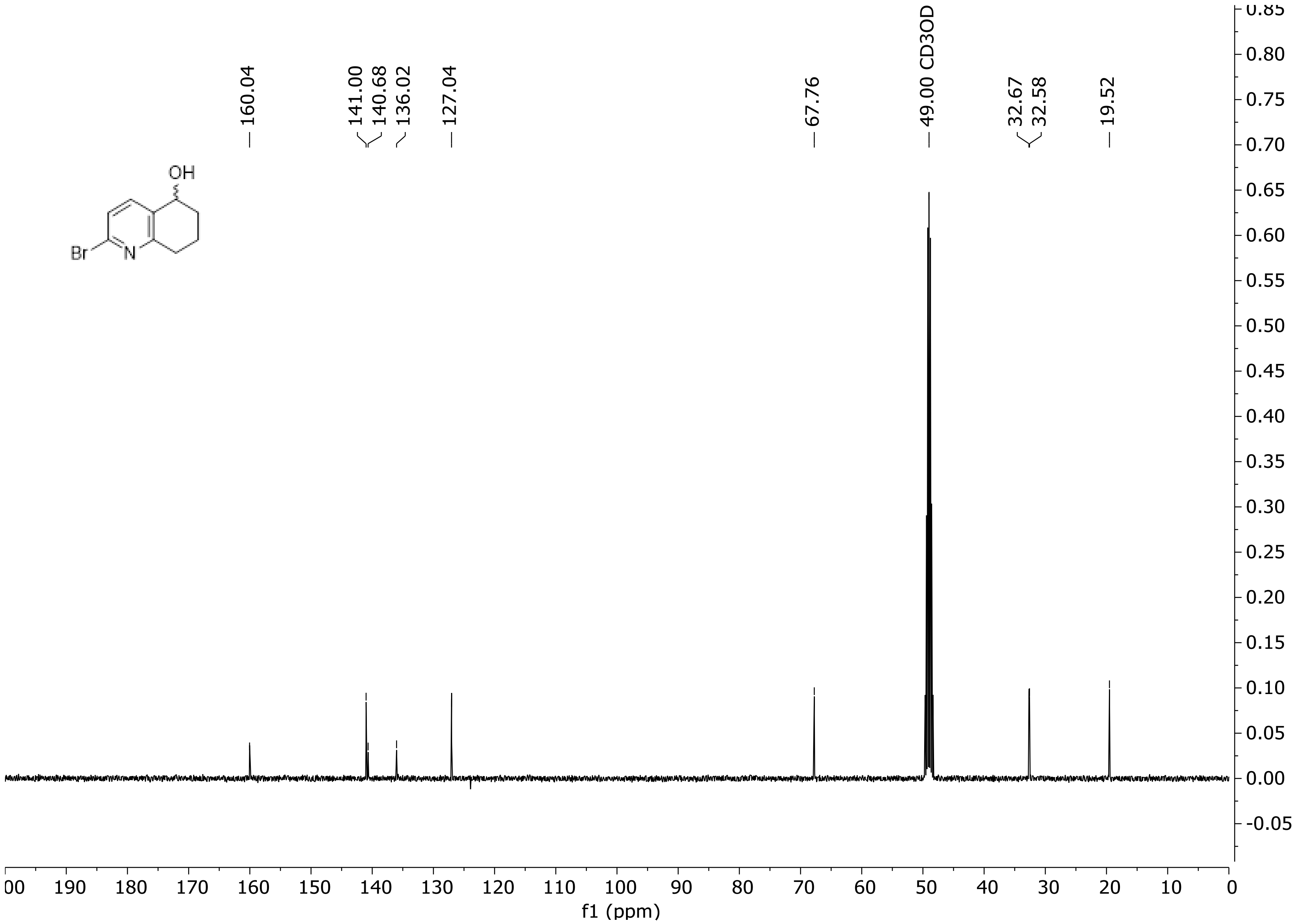

Figure S166. ^{13}C NMR (101 MHz, MeOH-d_4, 298K) of **80a**.

Mixture of *2-Bromo-5,6,7,8-tetrahydroquinolin-6-ol* **80b** (major isomer) and *2-Bromo-5,6,7,8-tetrahydroquinolin-7-ol* **80c** (minor isomer)

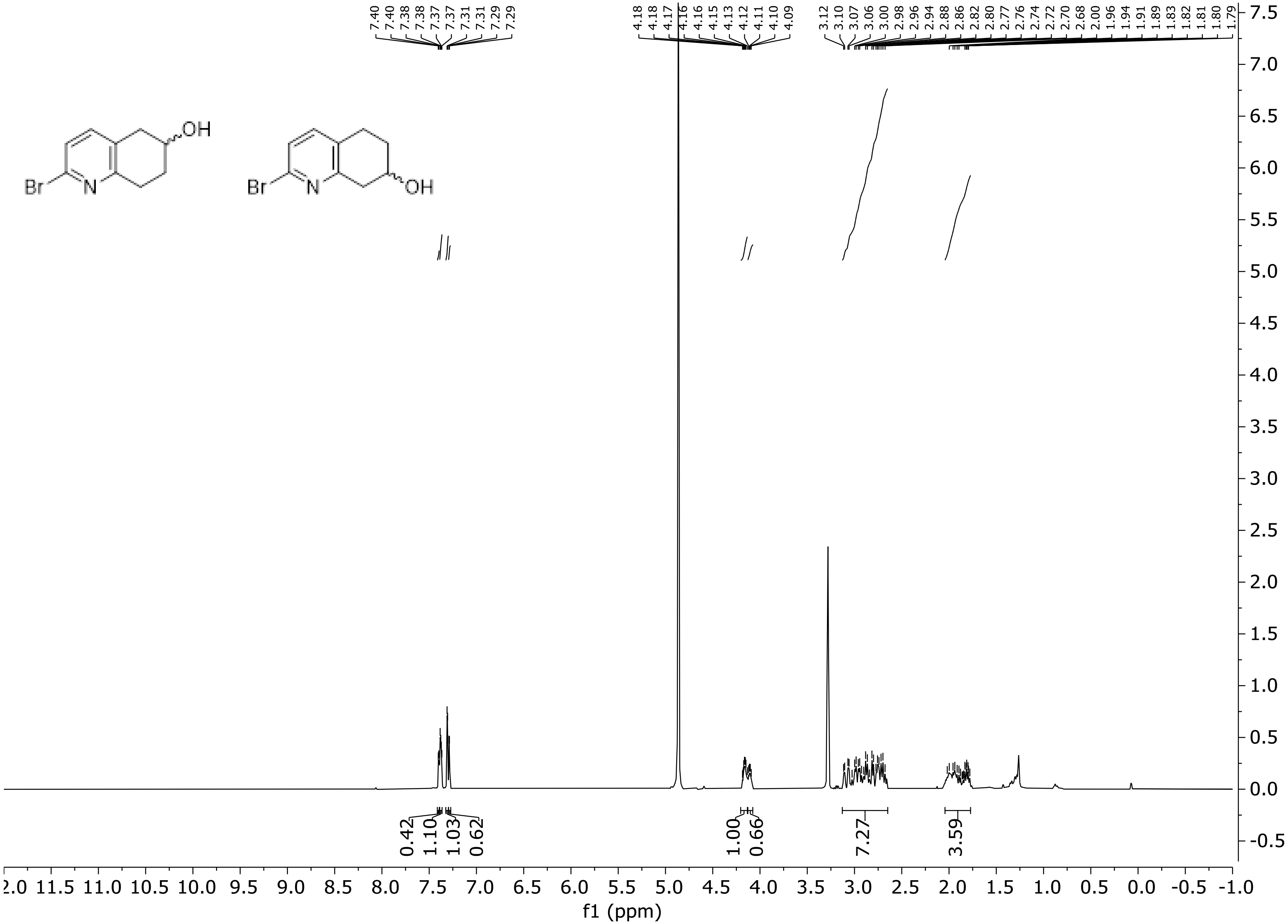

Figure S167. ^{1}H NMR (400 MHz, MeOH-d_4, 298K) of **80b** and **80c**.

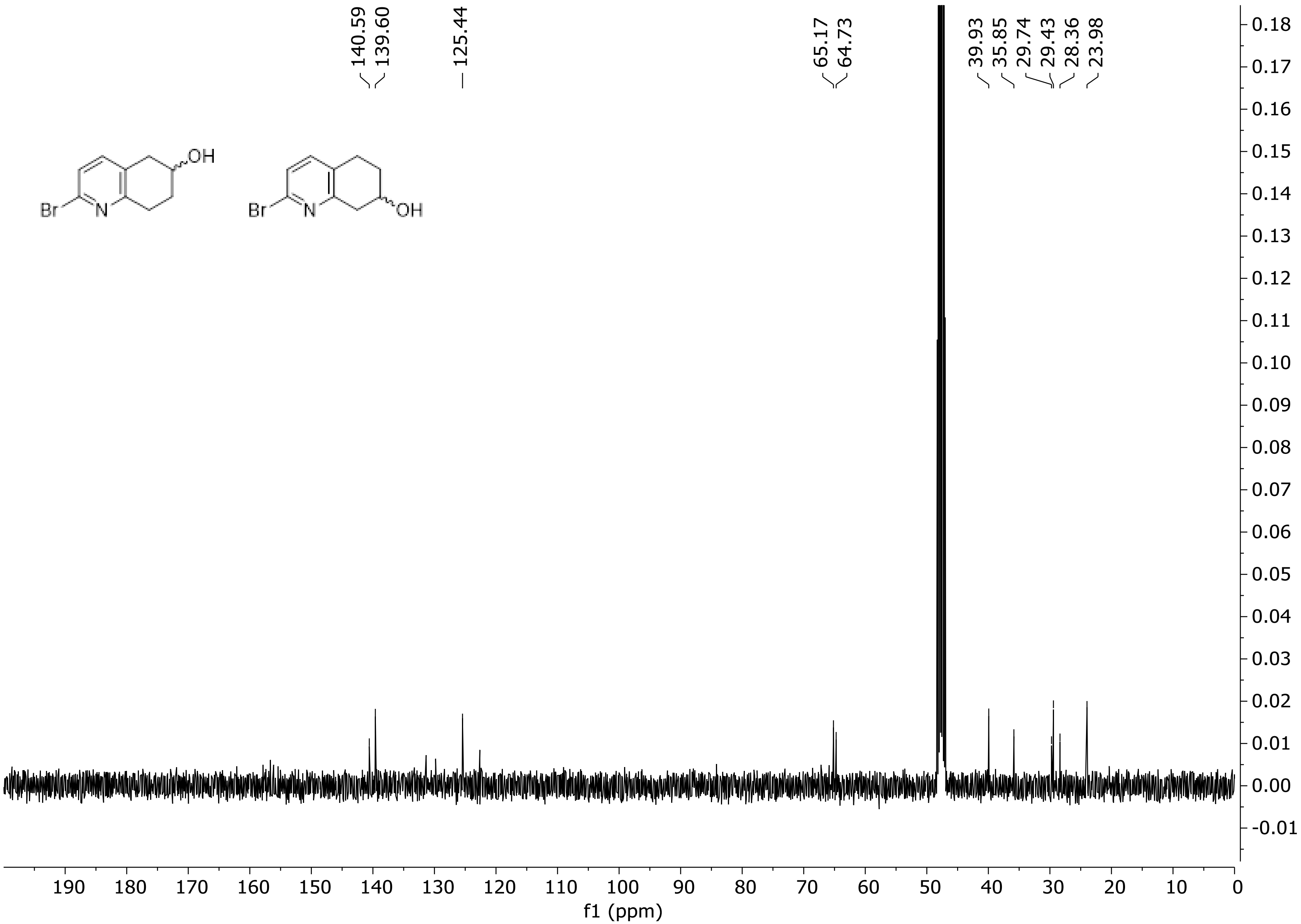

Figure S168. ^{13}C NMR (101 MHz, MeOH-d_4, 298K) of **80b** and **80c**.

(-)-5-Hydroxy-5,6,7,8-tetrahydroquinoline-2-carbonitrile **82a**

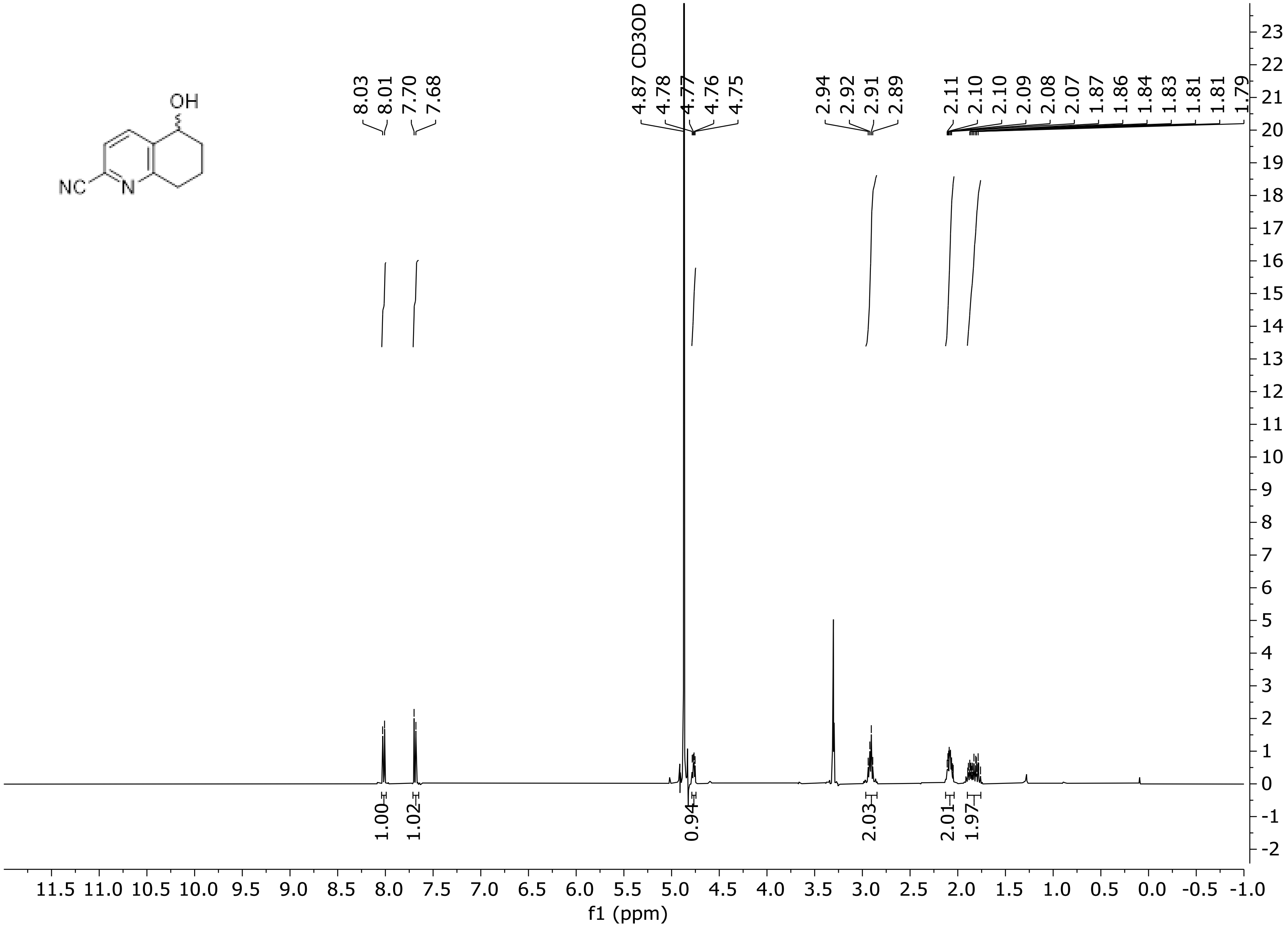

Figure S169. ^{1}H NMR (400 MHz, MeOH-d_4, 298K) of **82a**.

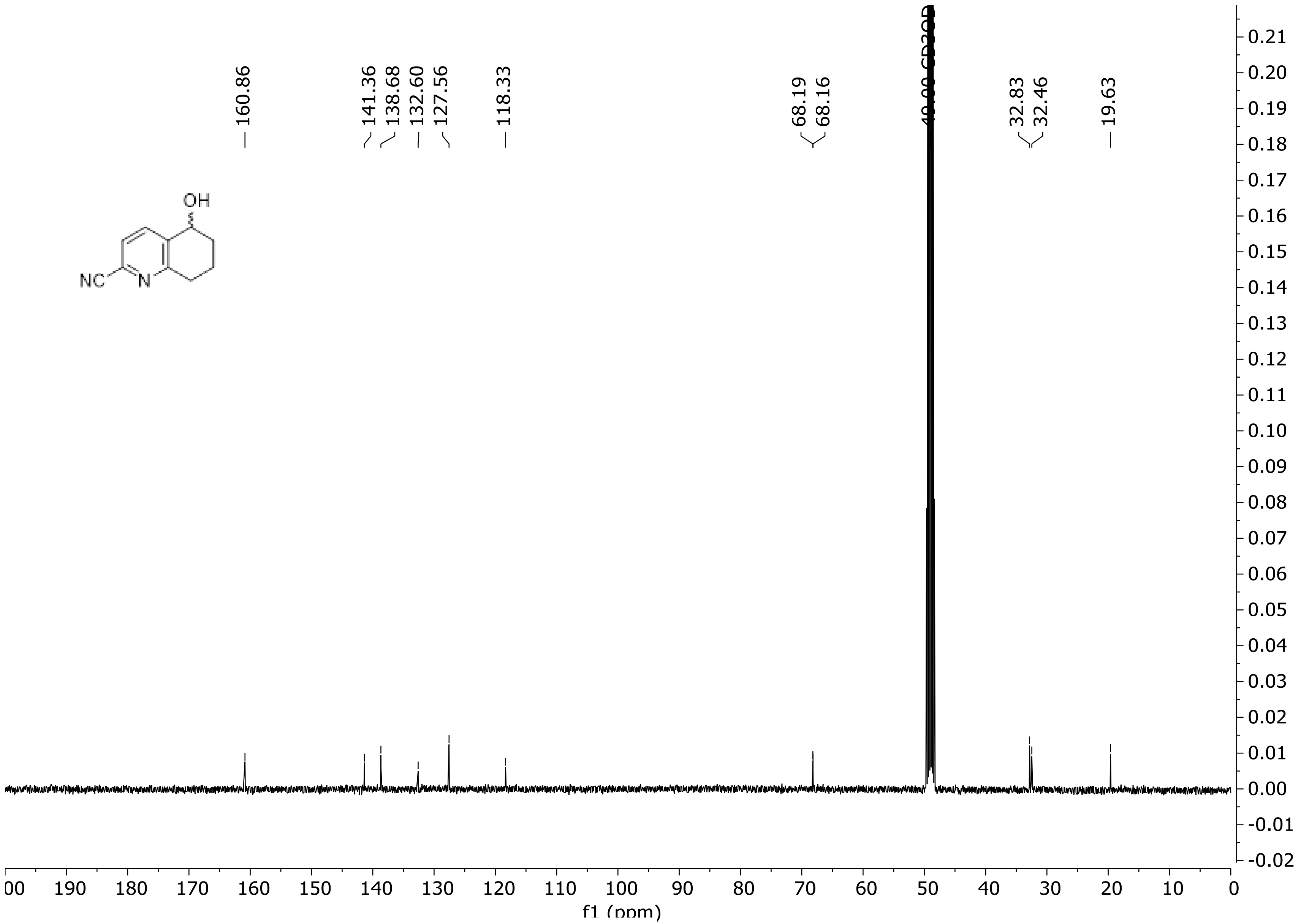

Figure S170. ^{13}C NMR (101 MHz, MeOH-d_4, 298K) of **82a**.

Mixture of *6-Hydroxy-5,6,7,8-tetrahydroquinoline-2-carbonitrile* **82b** (major isomer) and *7-Hydroxy-5,6,7,8-tetrahydroquinoline-2-carbonitrile* **82c** (minor isomer)

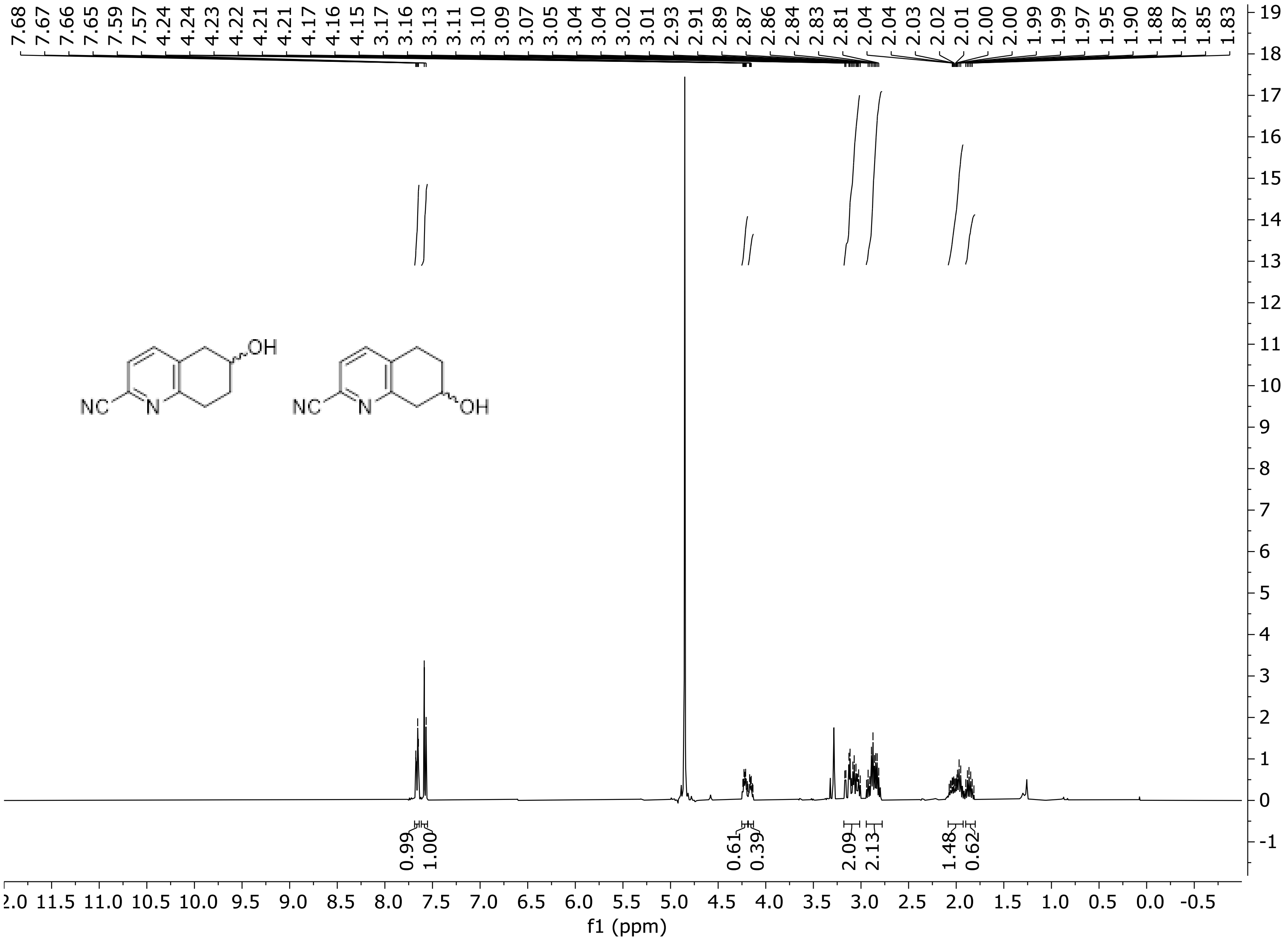

Figure S171. ^{1}H NMR (400 MHz, MeOH-d_4, 298K) of **82b** and **82c**.

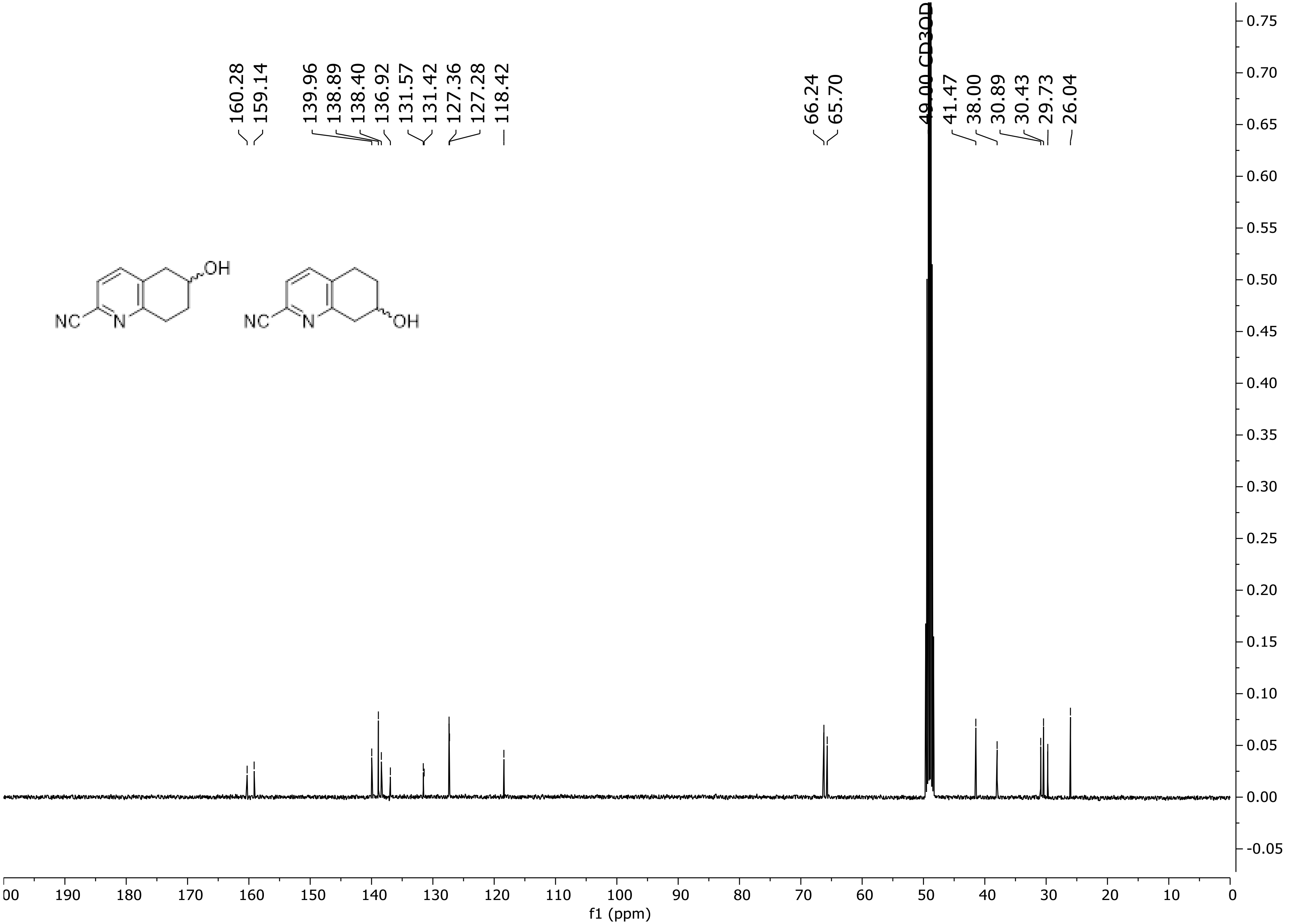

Figure S172. ^{13}C NMR (101 MHz, MeOH-d_4, 298K) of **82b** and **82c**.

5,6-Dihydroxy-5,6,7,8-tetrahydroquinoline-2-carbonitrile **82d**

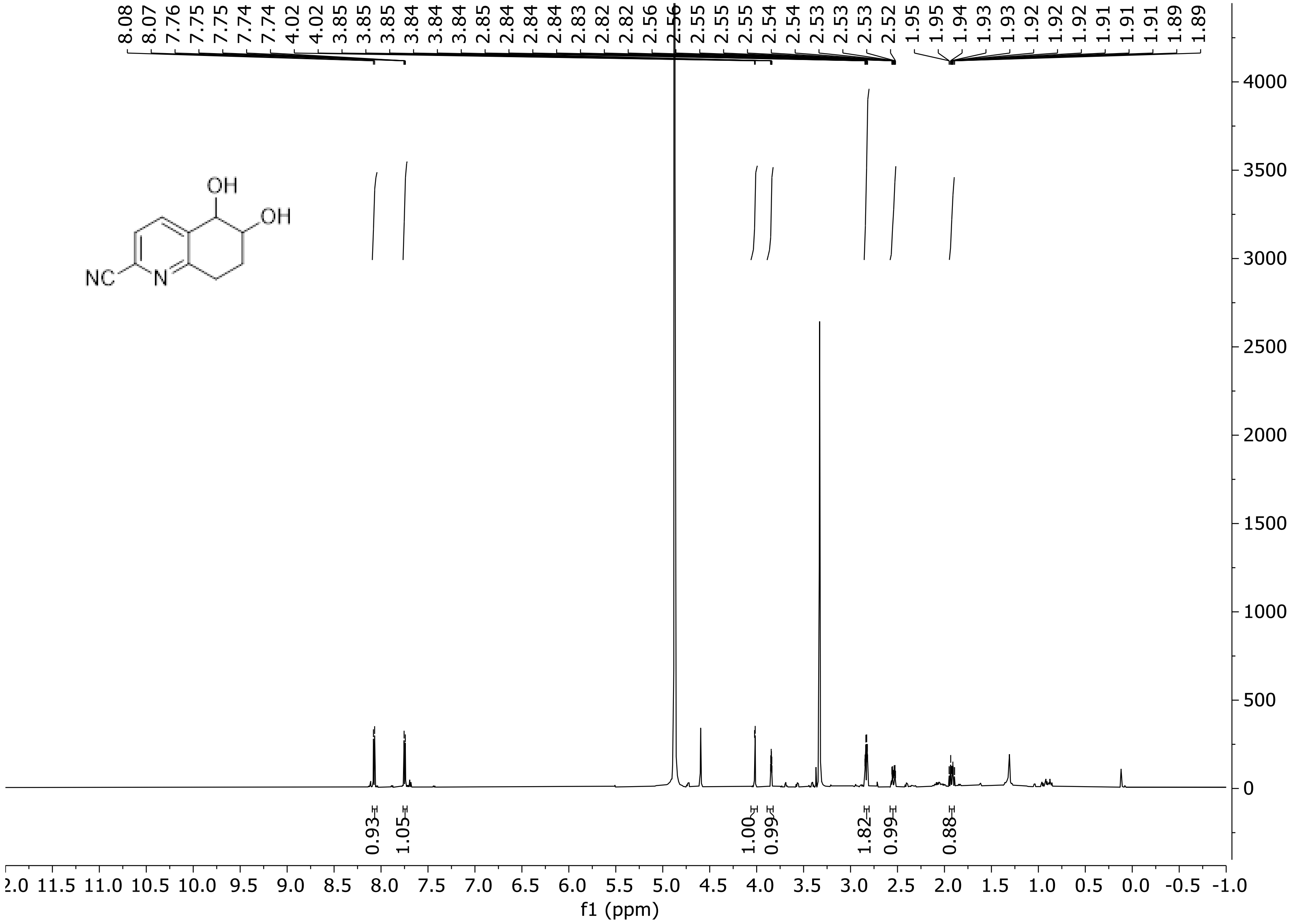

Figure S173. ^{1}H NMR (400 MHz, MeOH-d_4, 298K) of *trans*-**82d**.

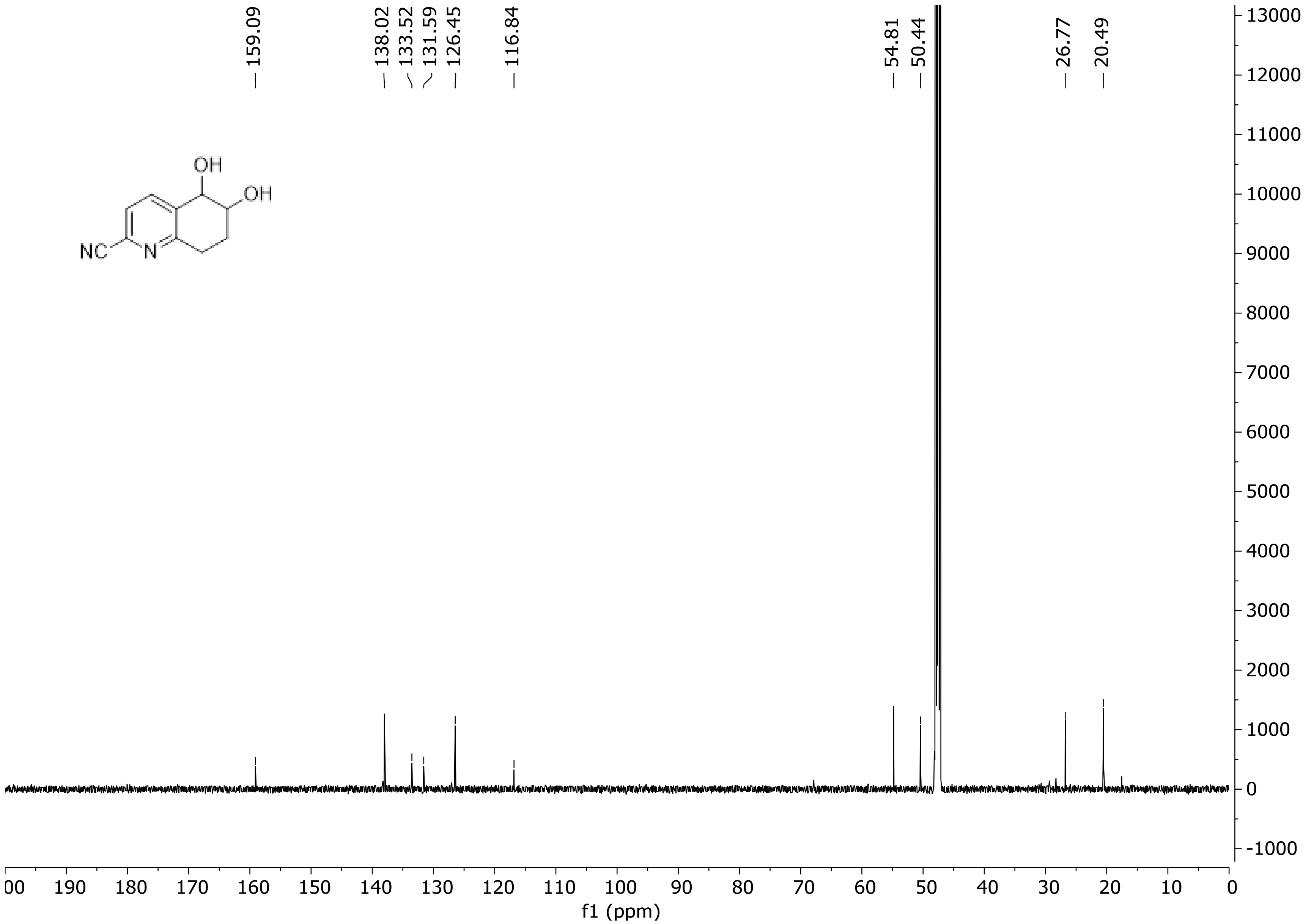

Figure S174. ^{13}C NMR (101 MHz, MeOH-d_4, 298K) of *trans*-**82d**.

(-)-2-(Methylsulfonyl)-5,6,7,8-tetrahydroquinolin-5-ol **84a**

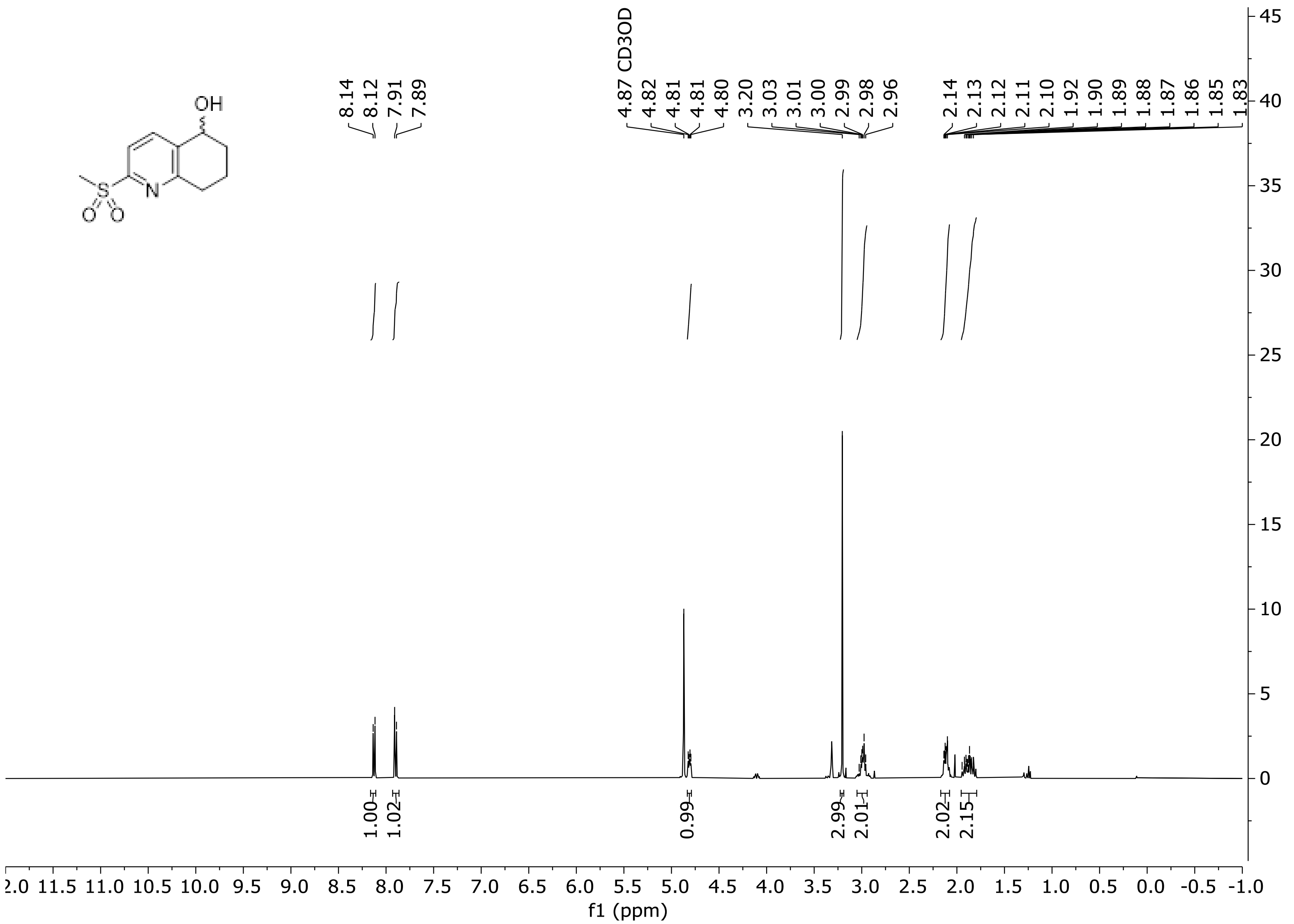

Figure S175. ^{1}H NMR (400 MHz, MeOH-d_4, 298K) of **84a**.

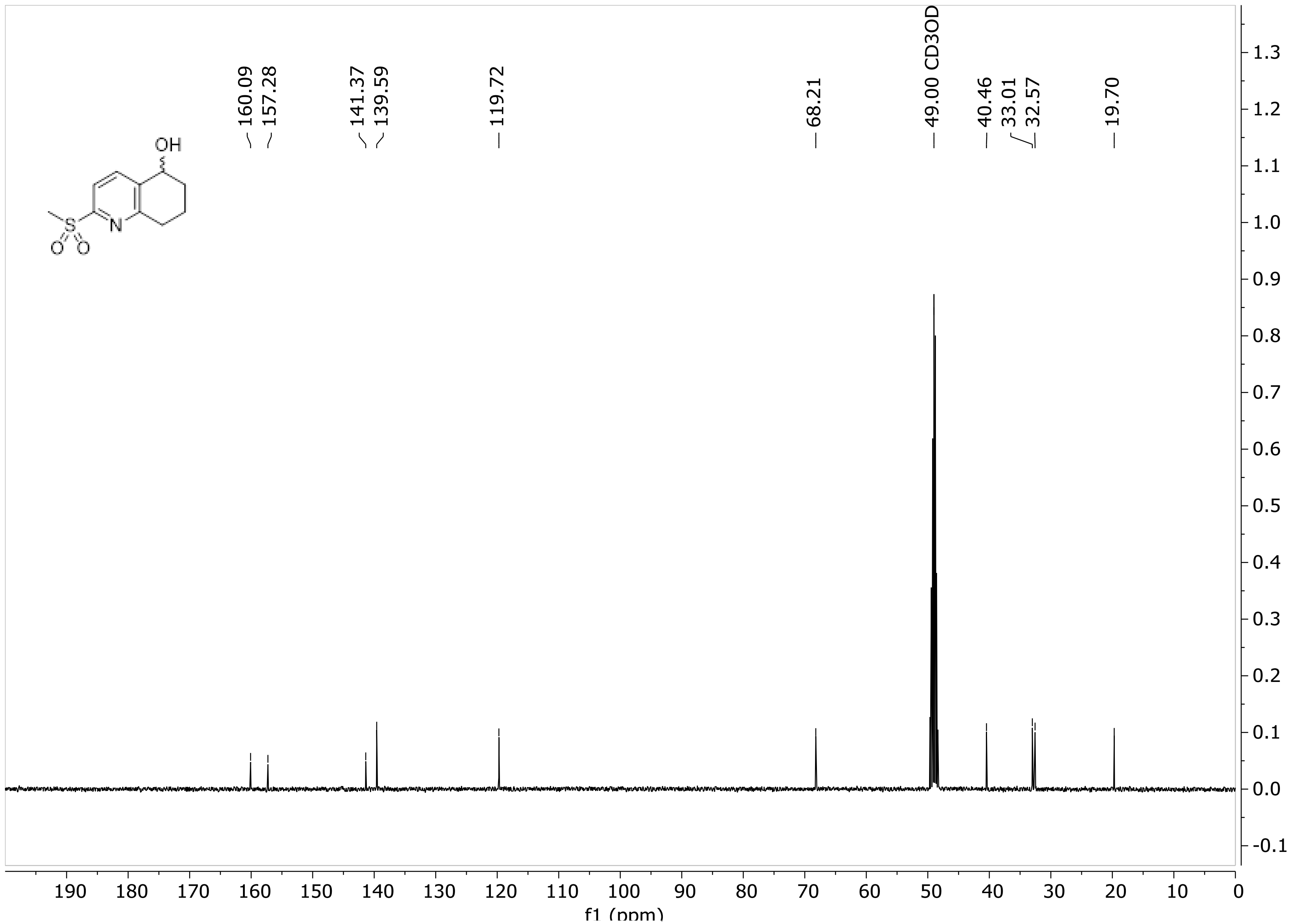

Figure S176. ^{13}C NMR (101 MHz, MeOH-d_4, 298K) of **84a**.

Mixture of *2-(Methylsulfonyl)-5,6,7,8-tetrahydroquinolin-6-ol* **84b** and *2-(Methylsulfonyl)-5,6,7,8-tetrahydroquinolin-7-ol* **84c**

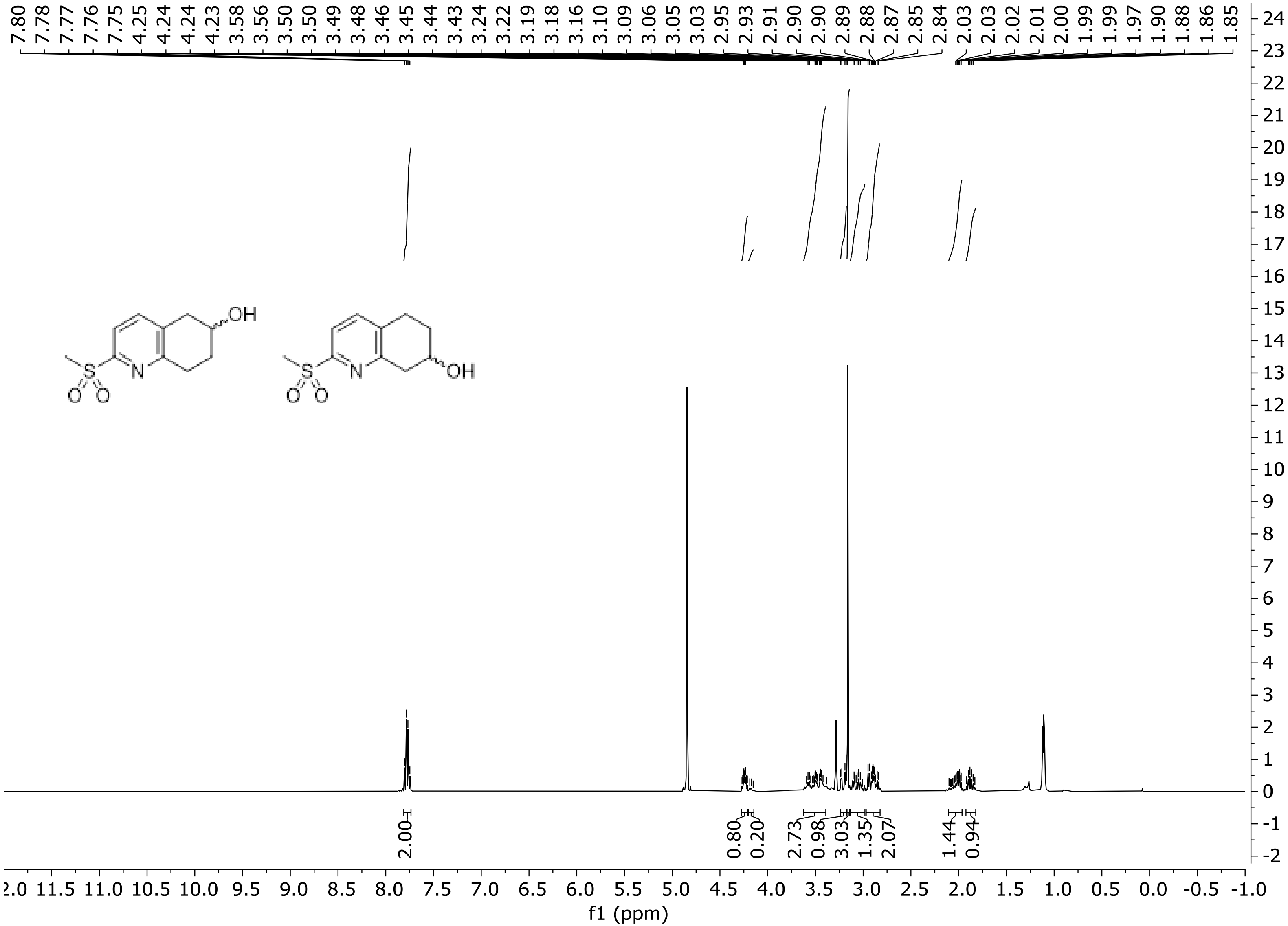

Figure S177. ^{1}H NMR (400 MHz, MeOH-d_4, 298K) of **84b** and **84c**.

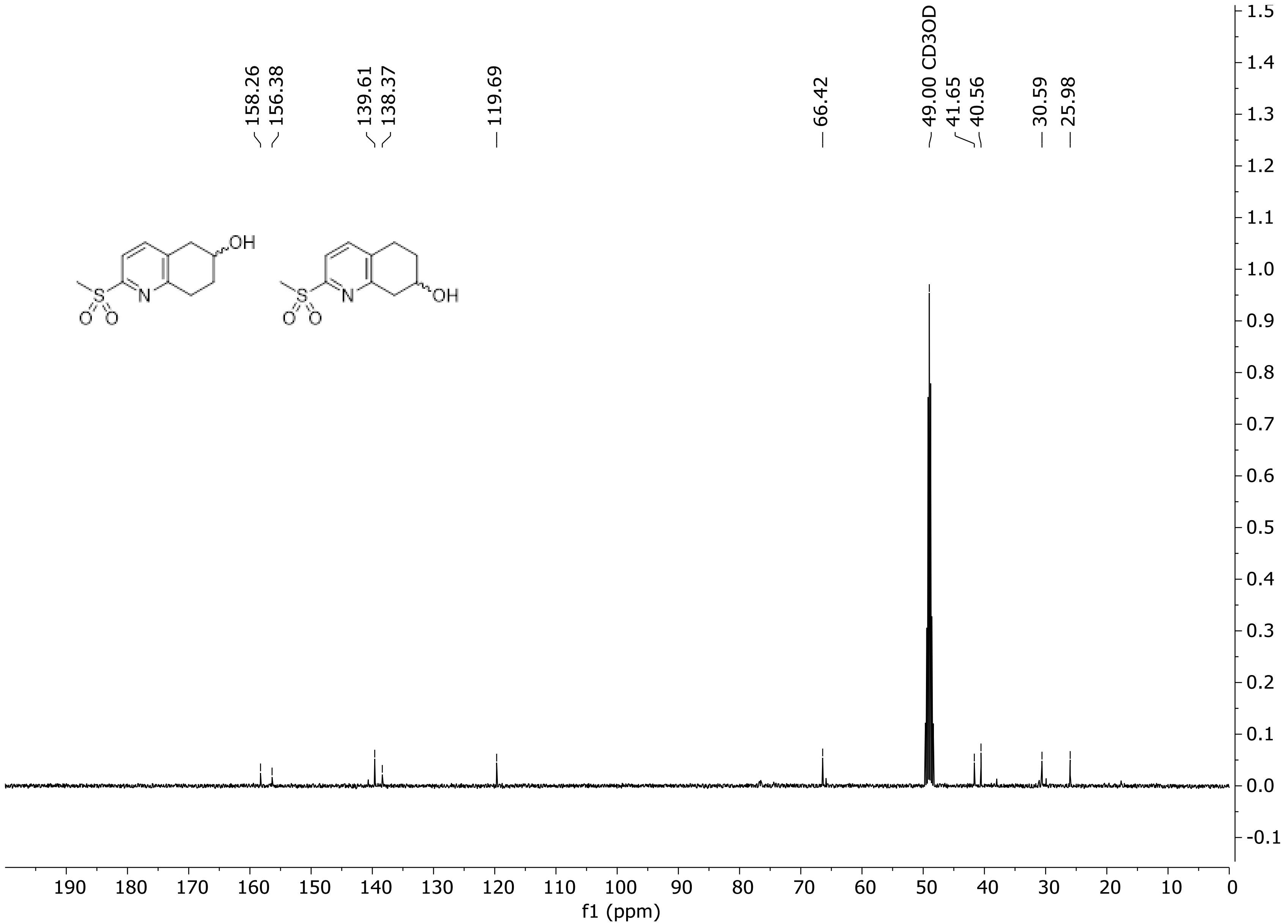

Figure S178. ^{13}C NMR (101 MHz, MeOH-d_4, 298K) of **84b** and **84c**.

(+)-2-Methoxy-5,6,7,8-tetrahydroquinolin-5-ol **86**

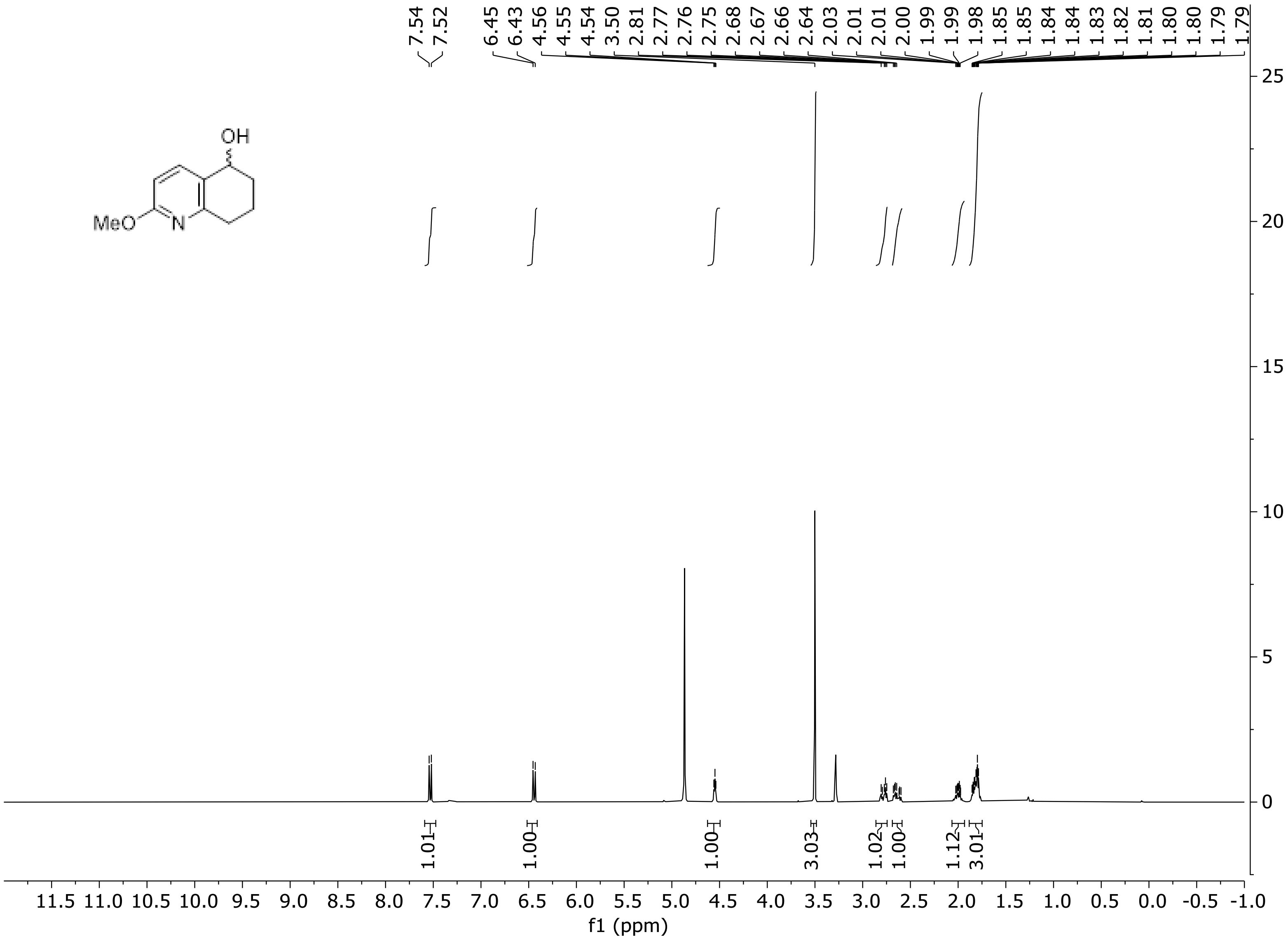

Figure S179. ^{1}H NMR (400 MHz, MeOH-d_4, 298K) of **86**.

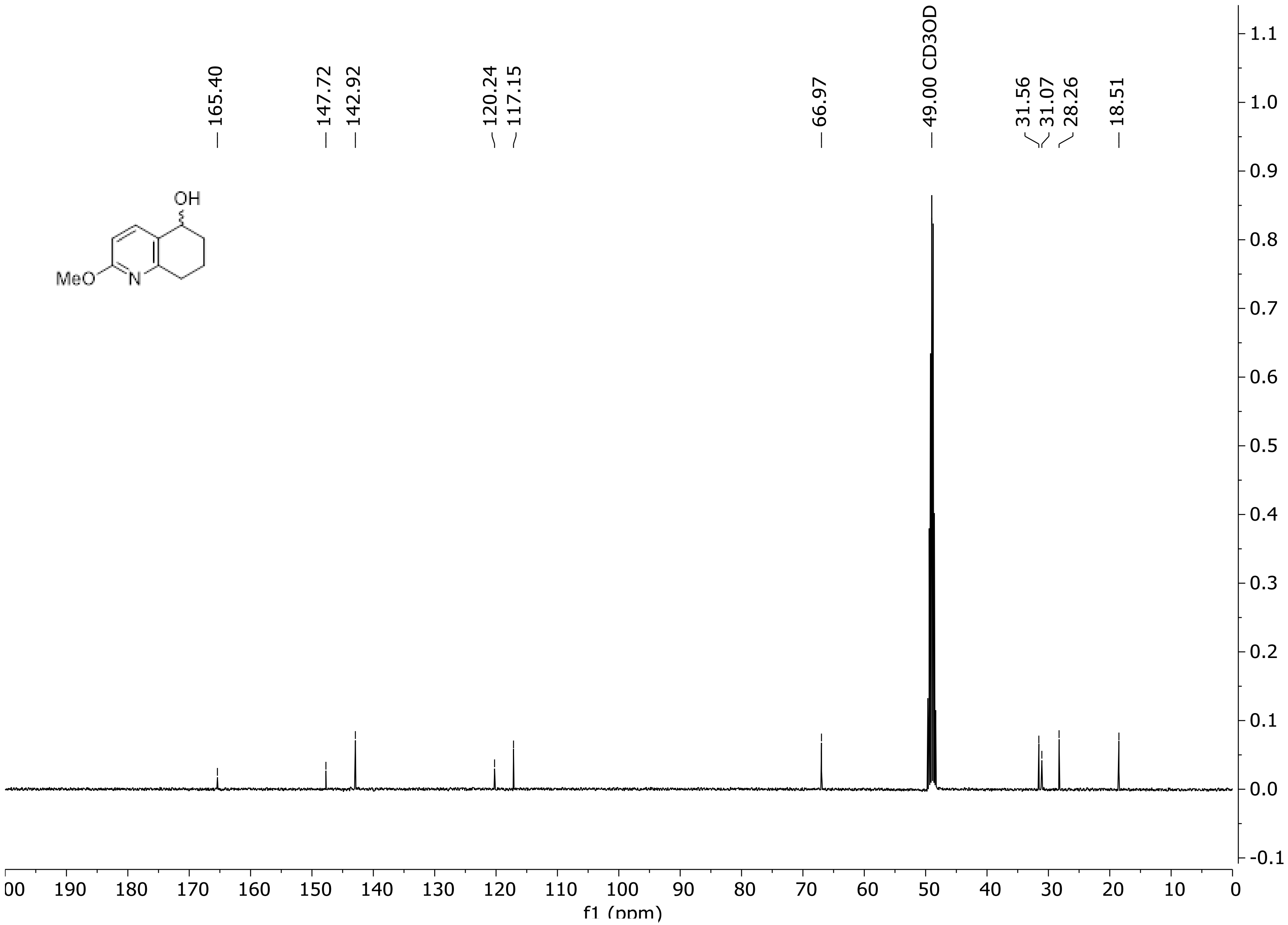

Figure S180. ^{13}C NMR (101 MHz, MeOH-d_4, 298K) of **86**.

(-)-3-Methoxy-5,6,7,8-tetrahydroquinolin-5-ol **88a**

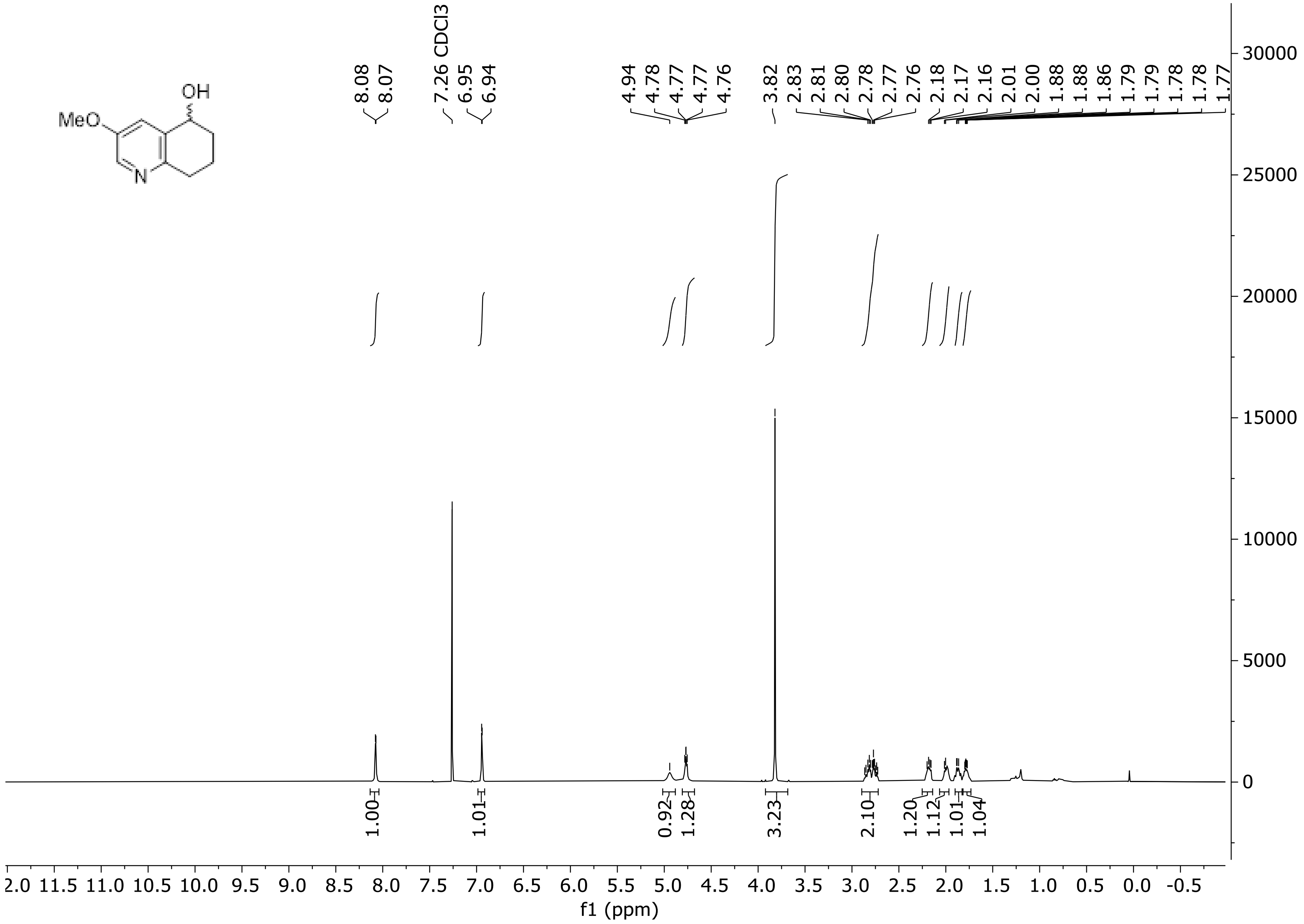

Figure S181. ^{1}H NMR (500 MHz, $CDCl_3$, 233K) of **88a**.

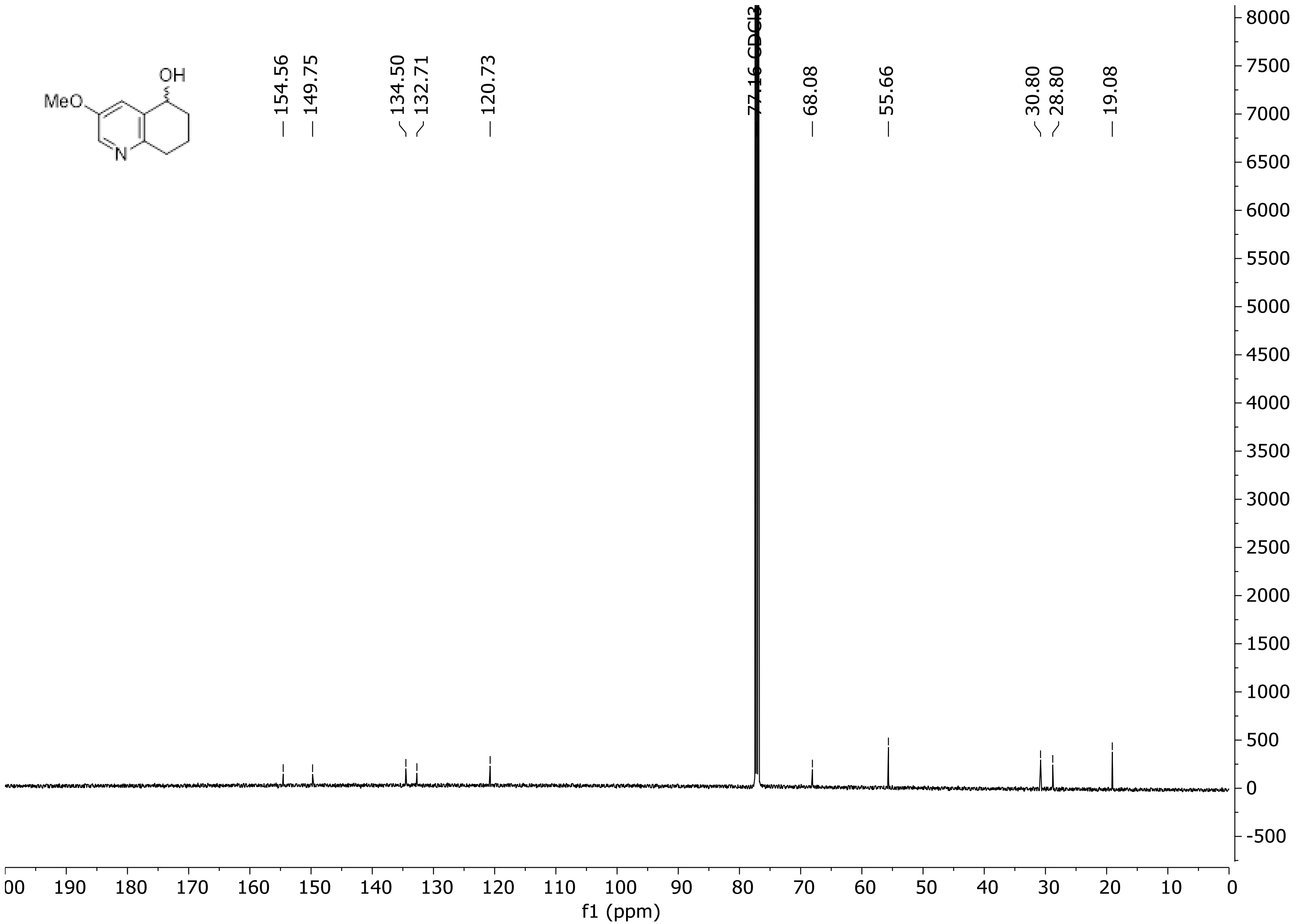

Figure S182. ^{13}C NMR (126 MHz, $CDCl_3$, 233K) of **88a**.

Mixture of *3-Methoxy-5,6,7,8-tetrahydroquinolin-6-ol* **88b** (major isomer) and *3-Methoxy-5,6,7,8-tetrahydroquinolin-7-ol* **88c** (minor isomer)

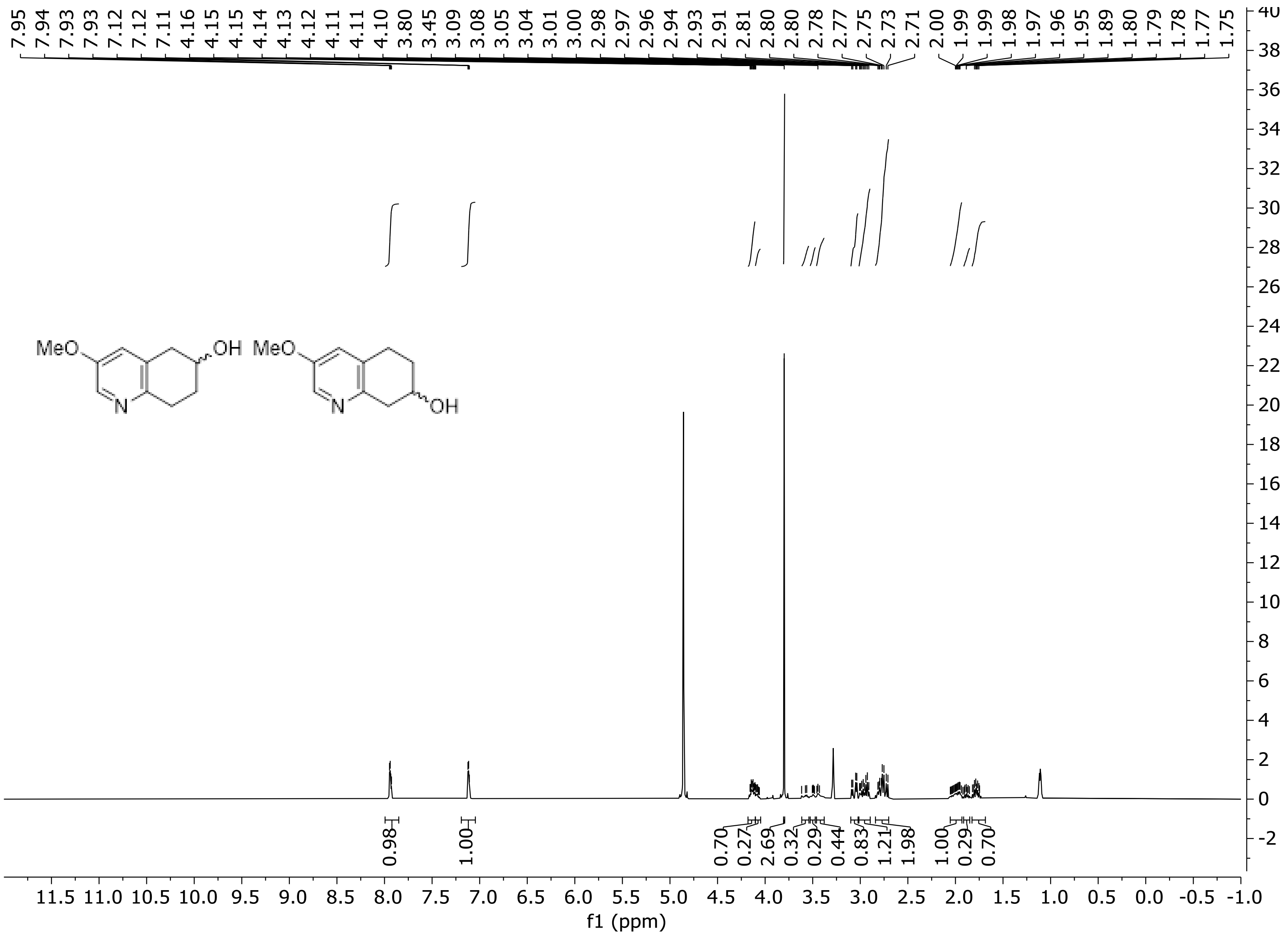

Figure S183. ^{1}H NMR (400 MHz, MeOH-d_4, 298K) of **88b** and **88c**.

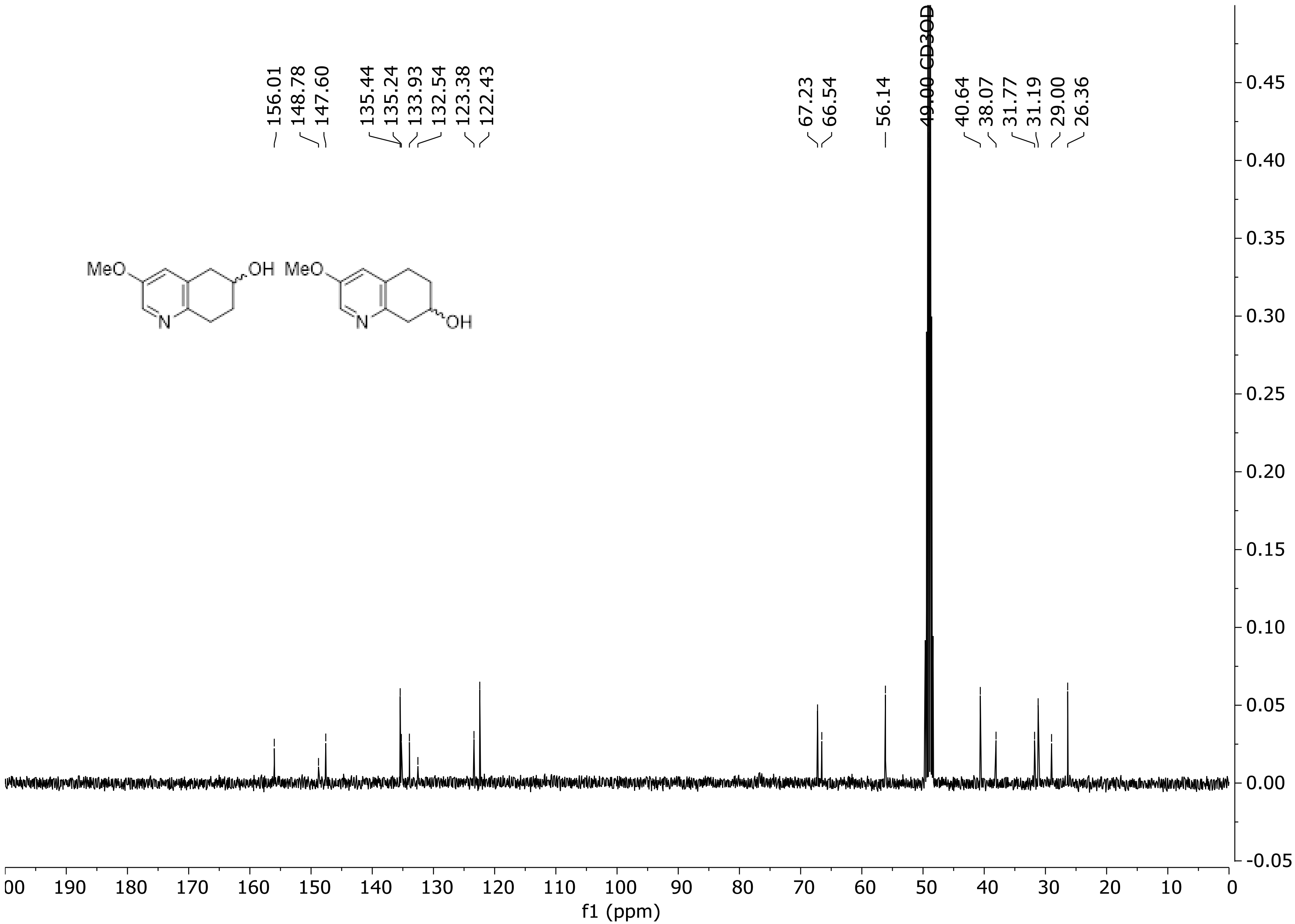

Figure S184. ^{13}C NMR (101 MHz, MeOH-d_4, 298K) of **88b** and **88c**.

Mixture of *3-(Methylsulfonyl)-5,6,7,8-tetrahydroquinolin-6-ol* **90a** (major isomer) and *3-(Methylsulfonyl)-5,6,7,8-tetrahydroquinolin-7-ol* **90b** (minor isomer)

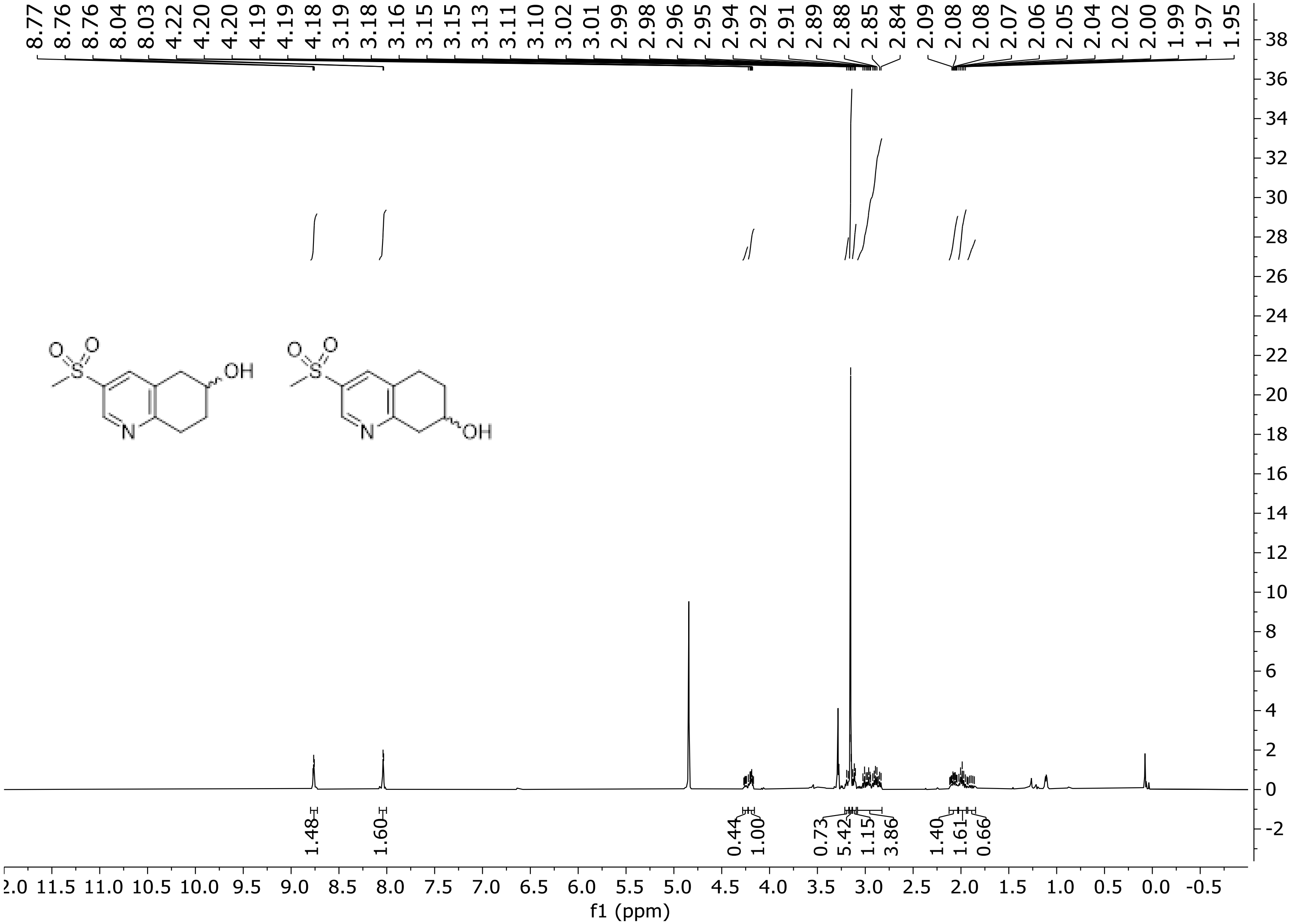

Figure S185. ^{1}H NMR (400 MHz, MeOH-d_4, 298K) of **90a** and **90b**.

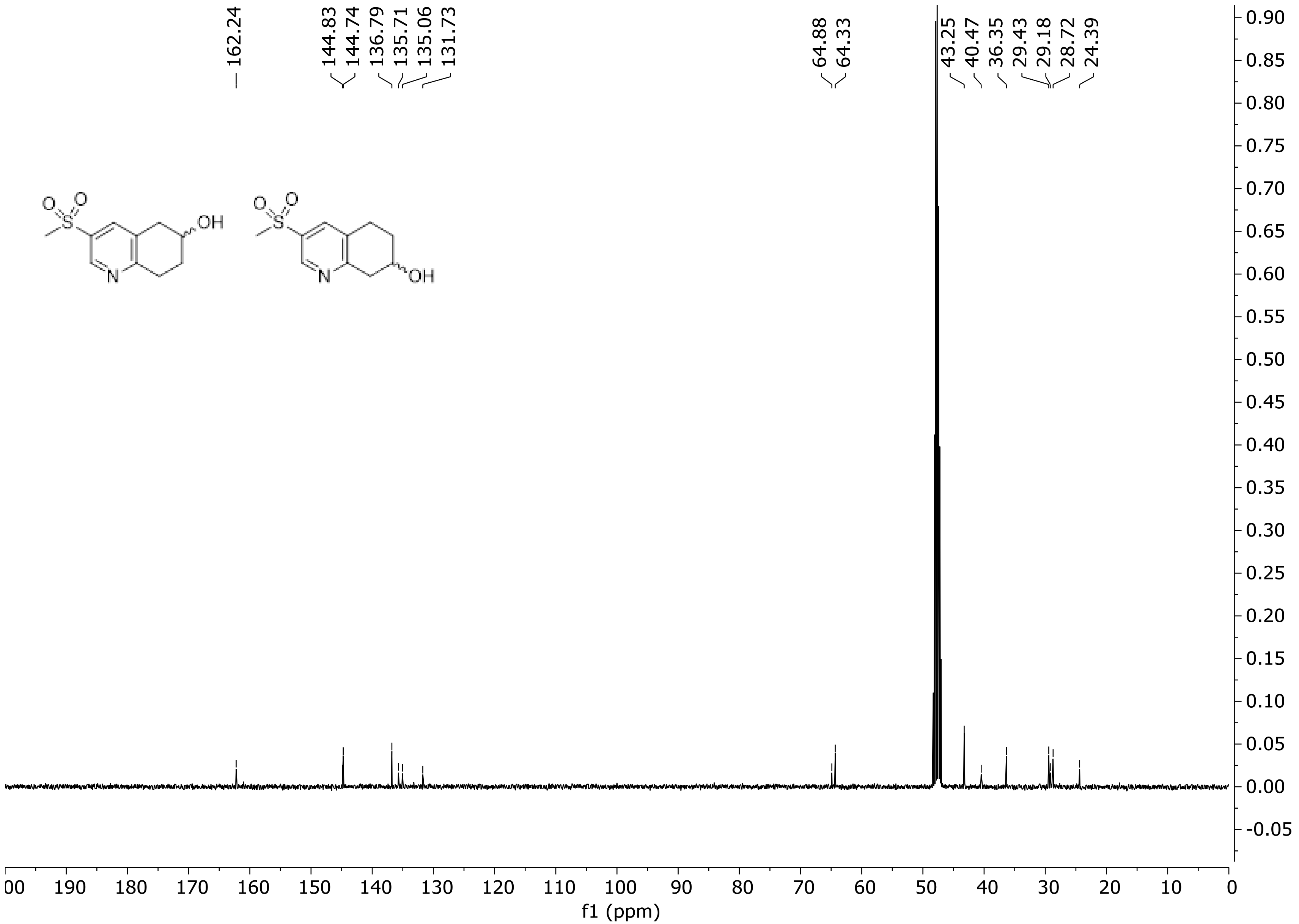

Figure S186. ^{13}C NMR (400 MHz, MeOH-d_4, 298K) of **90a** and **90b**.

(+)-3-(Methylsulfonyl)-5,6,7,8-tetrahydroquinolin-8-ol **90c**

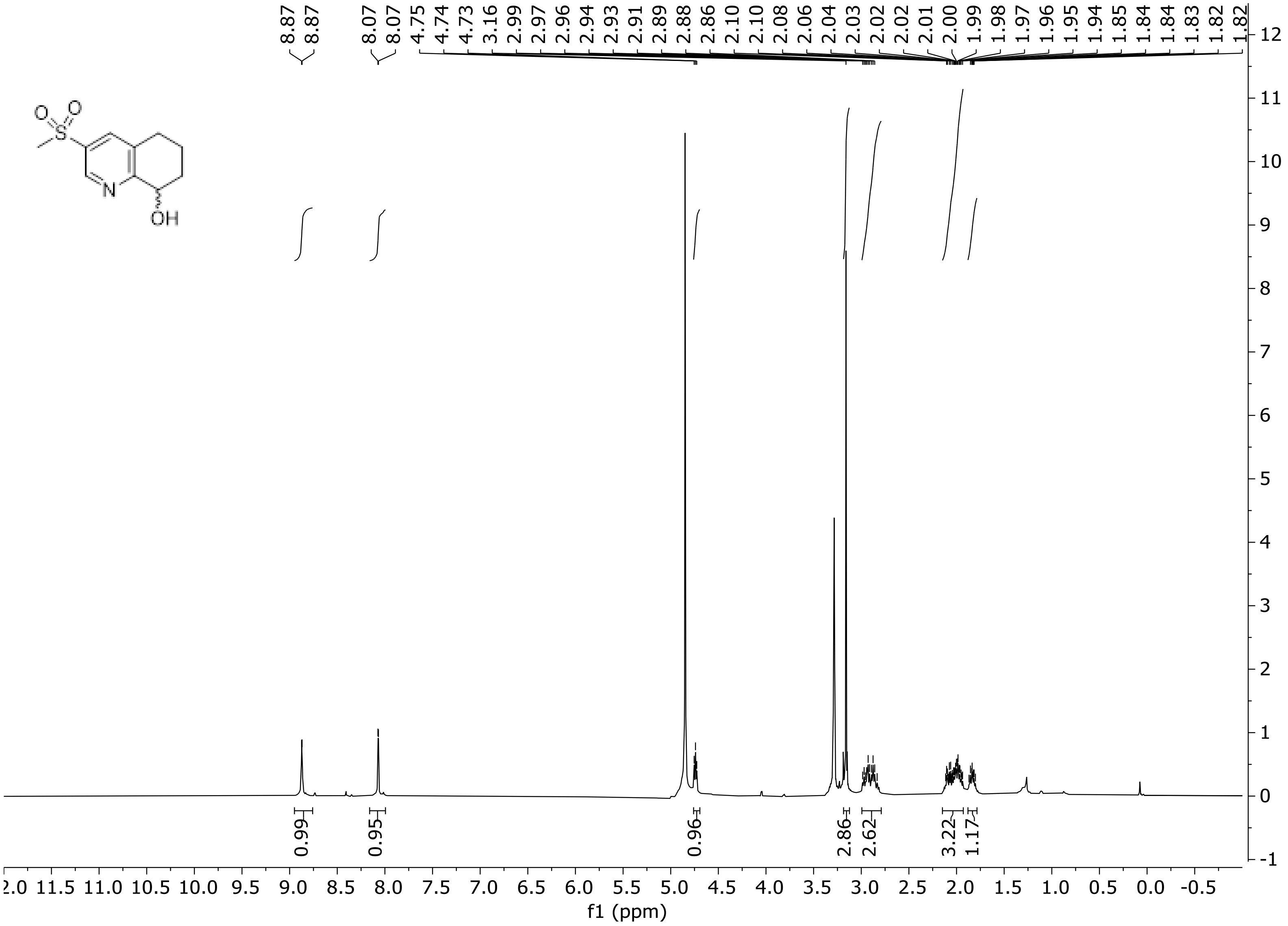

Figure S187. ^{1}H NMR (400 MHz, MeOH-d_4, 298K) of **90c**.

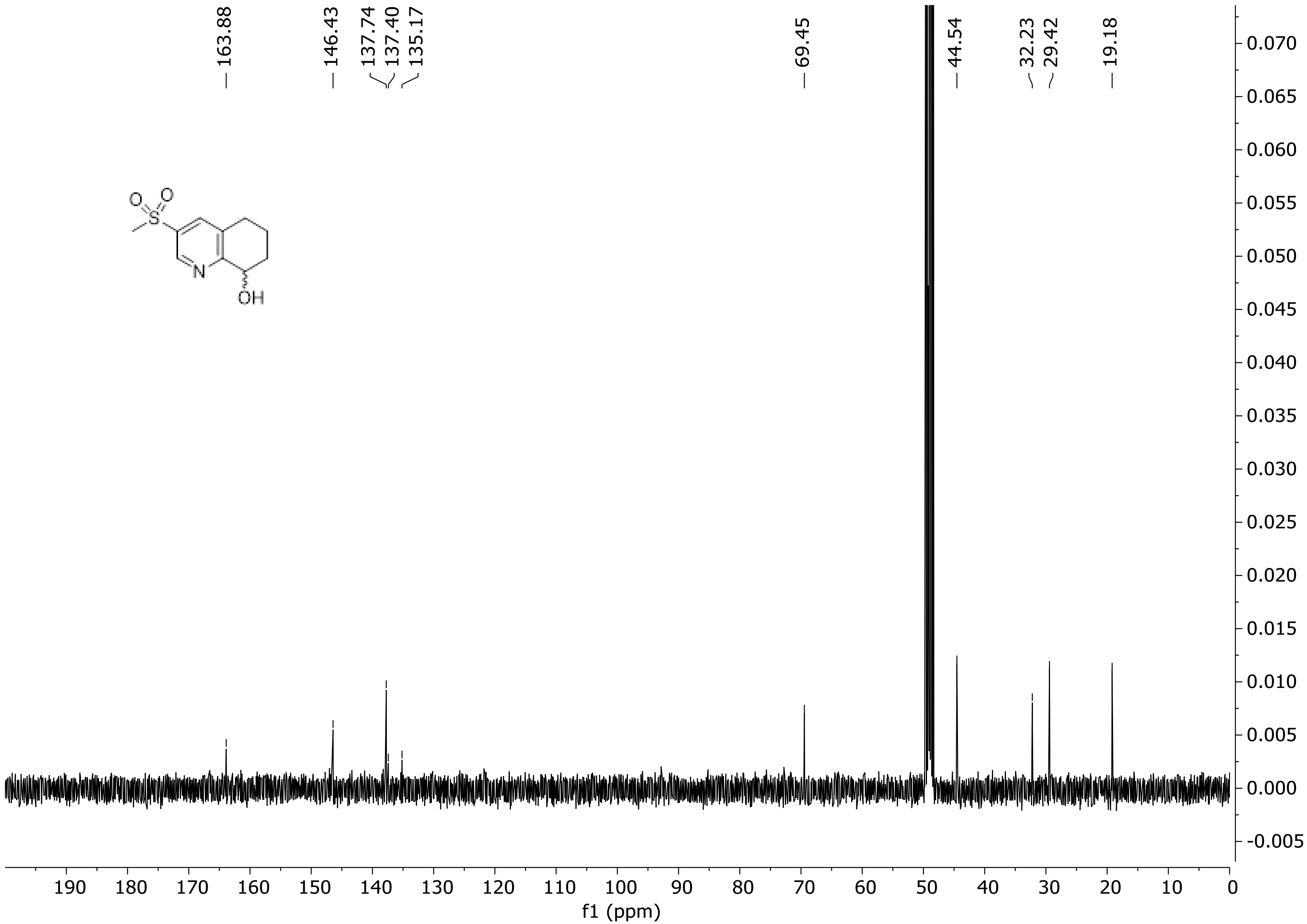

Figure S188. ^{13}C NMR (101 MHz, MeOH-d_4, 298K) of **90c**.

*(+/-)-3-Hydroxy-3-methylindolin-2-one rac-***91**

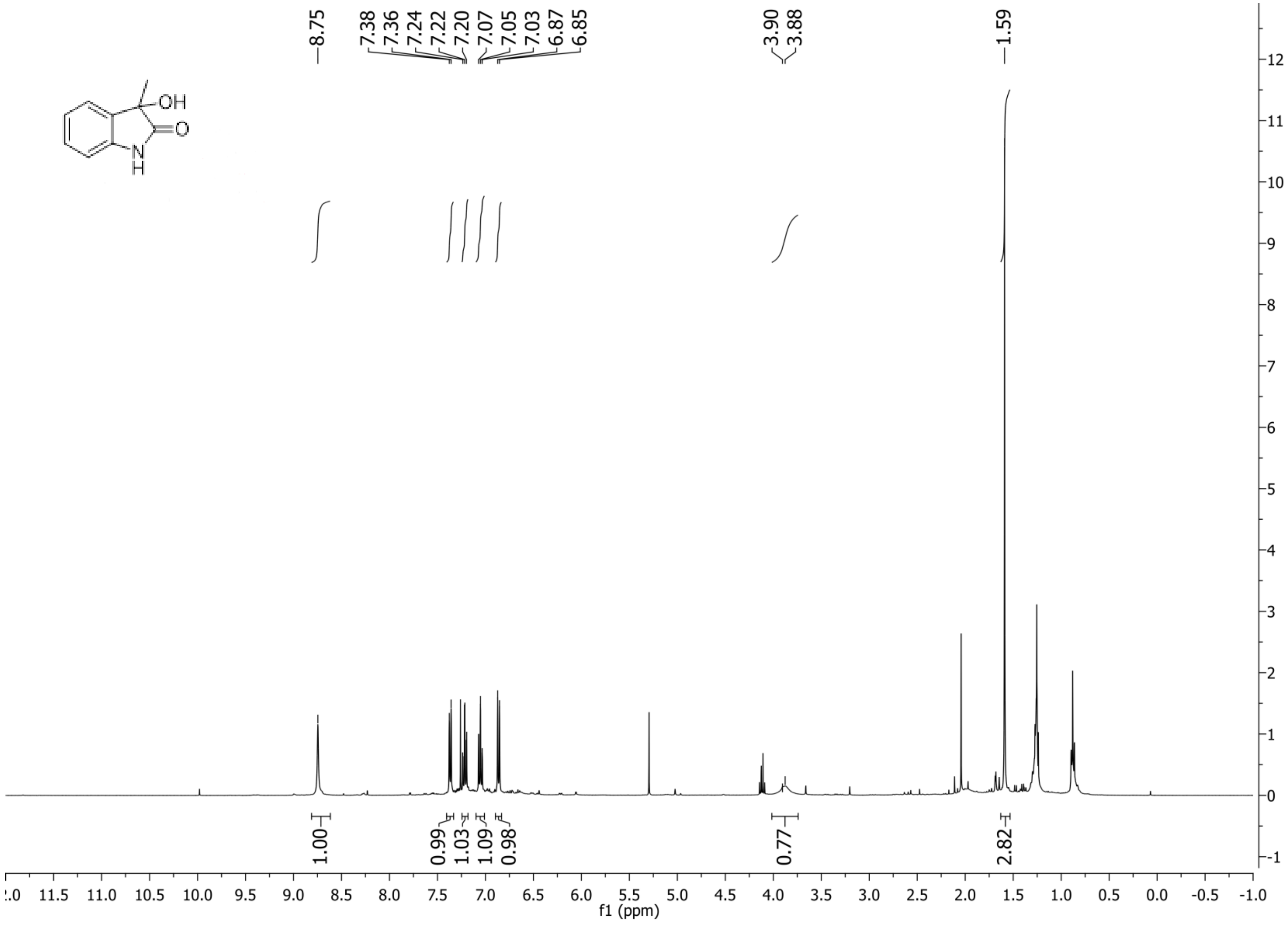

Figure S189. ^{1}H NMR (400 MHz, MeOH-d_4, 298K) of **91**.

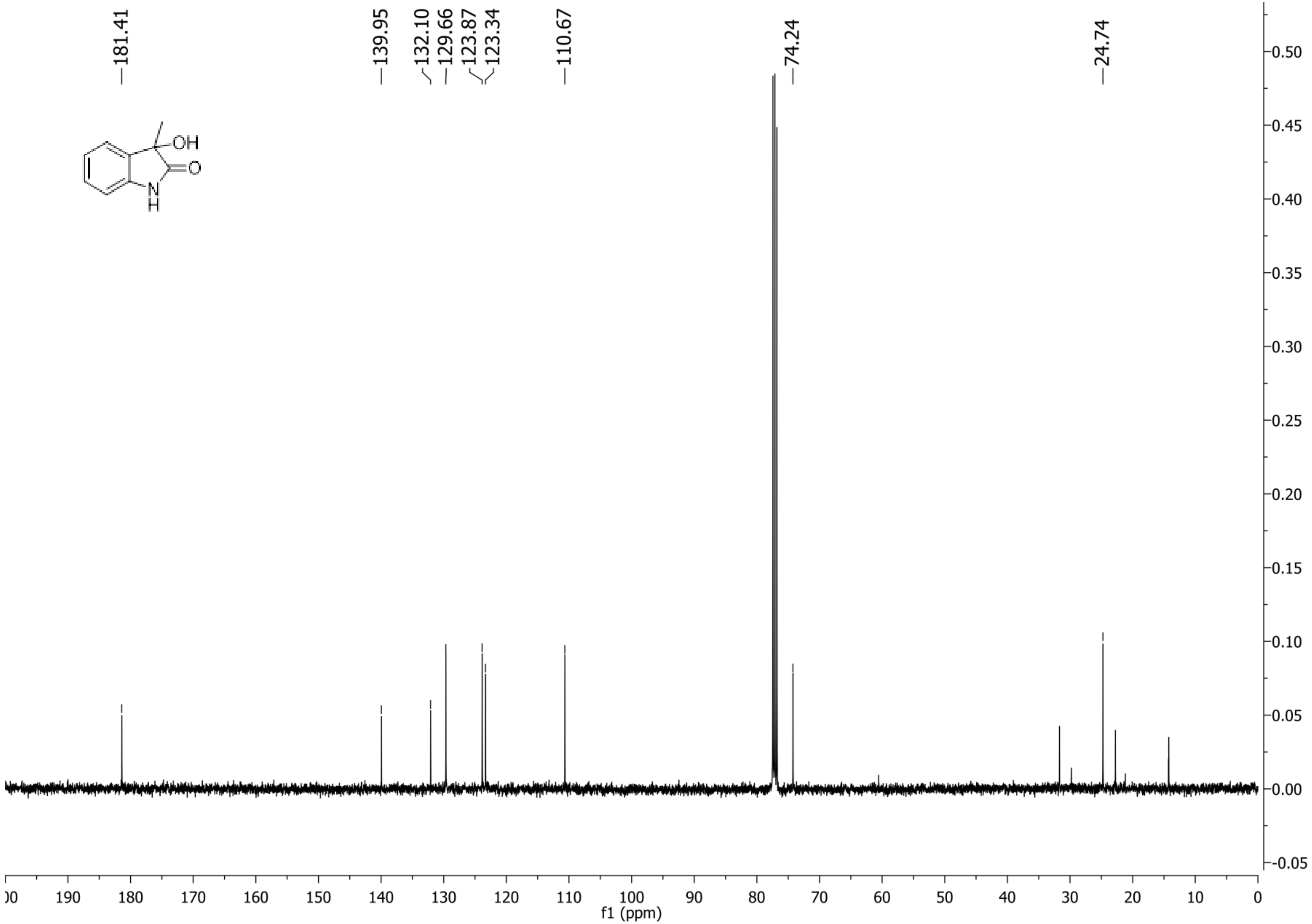

Figure S190. ^{13}C NMR (101 MHz, MeOH-d_4, 298K) of **91**.

15) References

[1] H. E. Bonfield, K. Mercer, A. Diaz-Rodriguez, G. C. Cook, B. S. J. McKay, P. Slade, G. M. Taylor, W. X. Ooi, J. D. Williams, J. P. M. Roberts, J. A. Murphy, L. Schmermund, W. Kroutil, T. Mielke, J. Cartwright, G. Grogan, L. J. Edwards, *ChemPhotoChem* **2020**, *4*, 45-51.

[2] C. Yung-Chi, W. H. Prusoff, *Biochem. Pharmacol.* **1973**, *22*, 3099–3108.

[3] G. Laudadio, S. Govaerts, Y. Wang, D. Ravelli, H. F. Koolman, M. Fagnoni, S. W. Djuric, T. Noël, *Angew. Chem. Int. Ed.* **2018**, *57*, 4078–4082.

[4] C. Pereira, S. Salgado, F. Rizzo-Aguiar, X. Garcia-Mera, J. Rodríguez-Borges, *Synlett* **2013**, *24*, 837–838.

[5] I. M. Bell, J. M. Erb, R. M. Freidinger, S. N. Gallicchio, J. P. Guare, M. T. Guidotti, R. A. Halpin, D. W. Hobbs, C. F. Homnick, M. S. Kuo, E. V. Lis, D. J. Mathre, S. R. Michelson, J. M. Pawluczyk, D. J. Pettibone, D. R. Reiss, S. Vickers, P. D. Williams, C. J. Woyden, *J. Med. Chem.* **1998**, *41*, 2146–2163.

[6] T. Fukuyama, T. Nishikawa, K. Yamada, D. Ravelli, M. Fagnoni, I. Ryu, *Org. Lett.* **2017**, *19*, 6436–6439.

[7] S. Sichler, G. Höfner, S. Rappenglück, T. Wein, K. V. Niessen, T. Seeger, F. Worek, H. Thiermann, F. F. Paintner, K. T. Wanner, *Toxicol. Lett.* **2018**, *293*, 172–183.

[8] K. Ganguli, A. Mandal, B. Sarkar, S. Kundu, *Tetrahedron* **2020**, *76*, 131439.

[9] V. Balakrishnan, V. Murugesan, B. Chindan, R. Rasappan, *Org. Lett.* **2021**, *23*, 1333–1338.

[10] F. C. Y. Chan, G. A. Potter, S. E. Barrie, B. P. Haynes, M. G. Rowlands, J. Houghton, M. Jarman, *J. Med. Chem.* **1996**, *39*, 3319–3323.

[11] D. Chen, Y. Zhang, X. Pan, F. Wang, S. Huang, *Adv. Synth. Catal.* **2018**, *360*, 3607–3612.

[12] H. Yang, N. Huo, P. Yang, H. Pei, H. Lv, X. Zhang, *Org. Lett.* **2015**, *17*, 4144–4147.

[13] A. R. Carmen, V. B. Marina, *Pyrazoloisoquinoline Derivatives*, **2007**, WO 2007/060198 A1.

[14] D. Tomohiro, Y. Hiroki, N. Takahiro, *α, β-Unsaturated Amide Compound*, **2018**, US11332455B2.

[15] K. Nienkemper, G. Kehr, S. Kehr, R. Fröhlich, G. Erker, *J. Organomet. Chem.* **2008**, *693*, 3063–3073.

[16] L. Jiang, Y. Huang, Y. Yan, Y. Xie, *Tetrahedron Lett.* **2016**, *57*, 4149–4151.

[17] J. Reutzel, T. M. Diogo, A. Geyer, *Chem. Eur. J.* **2017**, *23*, 8450–8456.

[18] S. E. Suh, S. J. Chen, M. Mandal, I. A. Guzei, C. J. Cramer, S. S. Stahl, *J. Am. Chem. Soc.* **2020**, *142*, 11388–11393.

[19] W. Bing, *Ceramide Galactosyltransferase Inhibitors for the Treatmnet of Disease*, **2017**, WO 2017/214505 A1.

[20] Z. Zhang, X. Hu, *Angew. Chem. Int. Ed.* **2021**, *60*, 22833–22838.

[21] W. Bin Im, S. H. Choi, J. Y. Park, S. H. Choi, J. Finn, S. H. Yoon, *Eur. J. Med. Chem.* **2011**, *46*, 1027–1039.

[22] J. J. Song, N. K. Yee, Z. Tan, J. Xu, S. R. Kapadia, C. H. Senanayake, *Org. Lett.* **2004**, *6*, 4905–4907.

[23] T. Sakamoto, S.-I. Kaneda, S. Nishimura, H. Yamanaka, *Chem. Pharm. Bull.* **1984**, *120*, 565–571.

[24] A. R. Katritzky, E. F. V. Scriven, S. Majumder, H. Tu, A. V. Vakulenko, N. G. Akhmedov, R. Murugan, *Synthesis.* **2005**, 993–997.

[25] R. W. Hartmann, M. Frotscher, *Arch. Pharm.* **1999**, *332*, 358–362.

[26] X. Wang, P. Rabbat, P. O'Shea, R. Tillyer, E. J. J. Grabowski, P. J. Reider, *Tetrahedron Lett.* **2000**, *41*, 4335–4338.

[27] C. Zhang, Y. Zhou, J. Huang, C. Tu, X. Zhou, G. Yin, *Org. Biomol. Chem.* **2018**, *16*, 6316–6321.

[28] J. Yang, L. Wang, Y. Lv, N. Li, Y. An, S. Gao, *Tetrahedron Lett.* **2018**, *59*, 156–159.

[29] W. J. Kerr, A. J. Morrison, M. Pazicky, T. Weber, *Org. Lett.* **2012**, *14*, 2250–2253.

[30] Y. M. Yang, W. Yan, H. W. Hu, Y. Luo, Z. Y. Tang, Z. Luo, *J. Org. Chem.* **2021**, *86*, 12344–12353.

[31] H. Jing, H. Li, J. C. Antilla, *Tetrahedron Lett.* **2020**, *61*, 152401.

[32] J. A. Steen, J. Poul, C. L. Brown, B. P. Stanley, K. Anders, *Pyrrolo-[2,1,5-CD]Indolizine Derivatives Useful in the Prevention or Treatment of Estrogen-Related Diseases or Syndromes*, **1998**, WO/1998/055482.

[33] S. Yoshinori, K. Kosuke, O. Katsunori, S. Kumi, H. Masato, Y. Shuji, *Pyridine Derivatives and Their Use as Medicaments for Treating Diseases Related to MCH Receptor*, **2006**, WO/2006/035967.

[34] S. C. Zimmerman, Z. Zeng, W. Wu, D. E. Reichert, *J. Am. Chem. Soc.* **1991**, *113*, 183–196.

[35] F. Antoni, D. Wifling, G. Bernhardt, *Eur. J. Med. Chem.* **2021**, *210*, DOI 10.1016/j.ejmech.2020.112958.

[36] L. NICOLAS, R. O. Y. PATRICK, L. YVES, *Pyridine Analogs as C5a Antagonists*, **2005**.

[37] L. Zhao, C. Tsukano, E. Kwon, Y. Takemoto, M. Hirama, *Angew. Chem. Int. Ed.* **2013**, *52*, 1722–1725.

[38] R. Kuwano, R. Ikeda, K. Hirasada, *Chem. Commun.* **2015**, *51*, 7558–7561.

[39] R. G. Alvarez, I. S. Hunter, C. J. Suckling, M. Thomas, U. Vitinius, *Tetrahedron* **2001**, *57*, 8581–8587.

[40] F. M. Piller, P. Appukkuttan, A. Gavryushin, M. Helm, P. Knochel, *Angew. Chem. Int. Ed.* **2008**, *47*, 6802–6806.

[41] H. Jing, H. Li, J. C. Antilla, *Tetrahedron Lett.* **2020**, *61*, 152401.

[42] L. E. Hanna, M. R. Harris, K. Domon, E. R. Jarvo, *Org. Lett.* **2017**, *19*, 6304–6307.

[43] A. J. Whitehouse, S. E. Thomas, K. P. Brown, A. Fanourakis, D. S. H. Chan, M. D. J. Libardo, V. Mendes, H. I. M. Boshoff, R. A. Floto, C. Abell, T. L. Blundell, A. G. Coyne, *J. Med. Chem.* **2019**, *62*, 7210–7232.

[44] R. Davenport, M. Silvi, A. Noble, Z. Hosni, N. Fey, V. K. Aggarwal, *Angew. Chemie Int. Ed.* **2020**, 1–5.

[45] H. Kurouchi, K. Kawamoto, H. Sugimoto, S. Nakamura, Y. Otani, T. Ohwada, *J. Org. Chem.* **2012**, *77*, 9313–9328.

[46] J. H. An, K. D. Kim, J. H. Lee, *J. Org. Chem.* **2021**, *86*, 2876–2894.

[47] Y. Goriya, H. Y. Kim, K. Oh, *Org. Lett.* **2016**, *18*, 5174–5177.

[48] S. P. Tanis, T. T. Parker, J. R. Colca, R. M. Fisher, R. F. Kletzein, *J. Med. Chem.* **1996**, *39*, 5053–5063.

[49] Y. Shaalan, L. Boulton, C. Jamieson, *Org. Proc. Res. Dev.* **2020**, *24*, 2745–2751.

[50] E. P. Kyba, S. T. Liu, K. Chockalingam, B. Raghava Reddy, *J. Org. Chem.* **1988**, *53*, 3513–3521.

[51] C. Ma, C. Q. Zhao, X. T. Xu, Z. M. Li, X. Y. Wang, K. Zhang, T. S. Mei, *Org. Lett.* **2019**, *21*, 2464–2467.

[52] X. B. Chen, Q. P. Hu, Q. J. Yuan, W. Ding, J. Ren, B. B. Zeng, *Tetrahedron Lett.* **2012**, *53*, 3798–3801.

[53] C. A. Arbour, B. Imperiali, *Org. Lett.* **2022**, *24*, 2170–2174.

[54] C. Bolm, M. Ewald, M. Felder, G. Schlinghoff, *Chem. Ber.* **1992**, *125*, 1169–1190.

[55] I. M. Bell, J. M. Erb, R. M. Freidinger, S. N. Gallicchio, J. P. Guare, M. T. Guidotti, R. A. Halpin, D. W. Hobbs, C. F. Homnick, M. S. Kuo, E. V. Lis, D. J. Mathre, S. R. Michelson, J. M. Pawluczyk, D. J. Pettibone, D. R. Reiss, S. Vickers, P. D. Williams, C. J. Woyden, *J. Med. Chem.* **1998**, *41*, 2146–2163.

[56] M. Hönel, F. W. Vierhapper, *J. Chem. Soc., Perkin Trans. 1* **1980**, 1933–1939.

[57] Y. Li, L. L. Wong, *Angew. Chem. Int. Ed.* **2019**, *58*, 9551–9555.

[58] K. D. Kim, J. H. Lee, *Org. Lett.* **2018**, *20*, 7712–7716.

[59] D. R. Boyd, R. J. H. Davies, L. Hamilton, J. J. McCullough, *J. Chem. Soc. Perkin Trans. 1* **1992**, 31–36.

[60] G. Bertuzzi, D. Pecorari, L. Bernardi, M. Fochi, *Chem. Commun.* **2018**, *54*, 3977–3980.

[61] G. Guanti, R. Riva, *Tetrahedron Asymmetry* **2001**, *12*, 1185–1200.

[62] P. Gandeepan, P. Rajamalli, C. H. Cheng, *Synth.* **2016**, *48*, 1872–1879.

[63] F. C. Jia, C. Xu, Z. W. Zhou, Q. Cai, Y. D. Wu, A. X. Wu, *Org. Lett.* **2016**, *18*, 5232–5235.

[64] B. Li, Y. Park, S. Chang, *J. Am. Chem. Soc.* **2014**, *136*, 1125–1131.

[65] D. B. England, G. Merey, A. Padwa, *Org. Lett.* **2007**, *9*, 3805–3807.

[66] R. Bigler, R. Huber, A. Mezzetti, **2015**, 5171–5174.

[67] A. L. Zhang, L. W. Yang, N. F. Yang, Y. L. Liu, *Tetrahedron Asymmetry* **2014**, *25*, 289–297.

[68] S. Kaiser, S. P. Smidt, A. Pfaltz, *Angew. Chem. Int. Ed.* **2006**, *45*, 5194–5197.

[69] M. W. Gribble, M. T. Pirnot, J. S. Bandar, R. Y. Liu, S. L. Buchwald, *J. Am. Chem. Soc.* **2017**, *139*, 2192–2195.

[70] S. Rodríguez, B. Qu, K. R. Fandrick, F. Buono, N. Haddad, Y. Xu, M.A. Herbage, X. Zeng, S. MA, N. Grinberg, H. Lee, Z.S. Han, N.K. Yee, C.H. Senanayake, *Adv. Synth. Catal.*, **2014**, *356*, 301-307.
